# Supplementary material for: Colora: a Snakemake workflow for complete chromosome-scale de novo genome assembly
Source: Bioinformatics. 2025 Apr 16;41(5):btaf175. doi: 10.1093/bioinformatics/btaf175 (PMC12065627; doi:10.1093/bioinformatics/btaf175)
Supplement: btaf175_Supplementary_Data [file btaf175_supplementary_data.zip › Additional_files/S17_Hi-C_contact_maps.pdf]

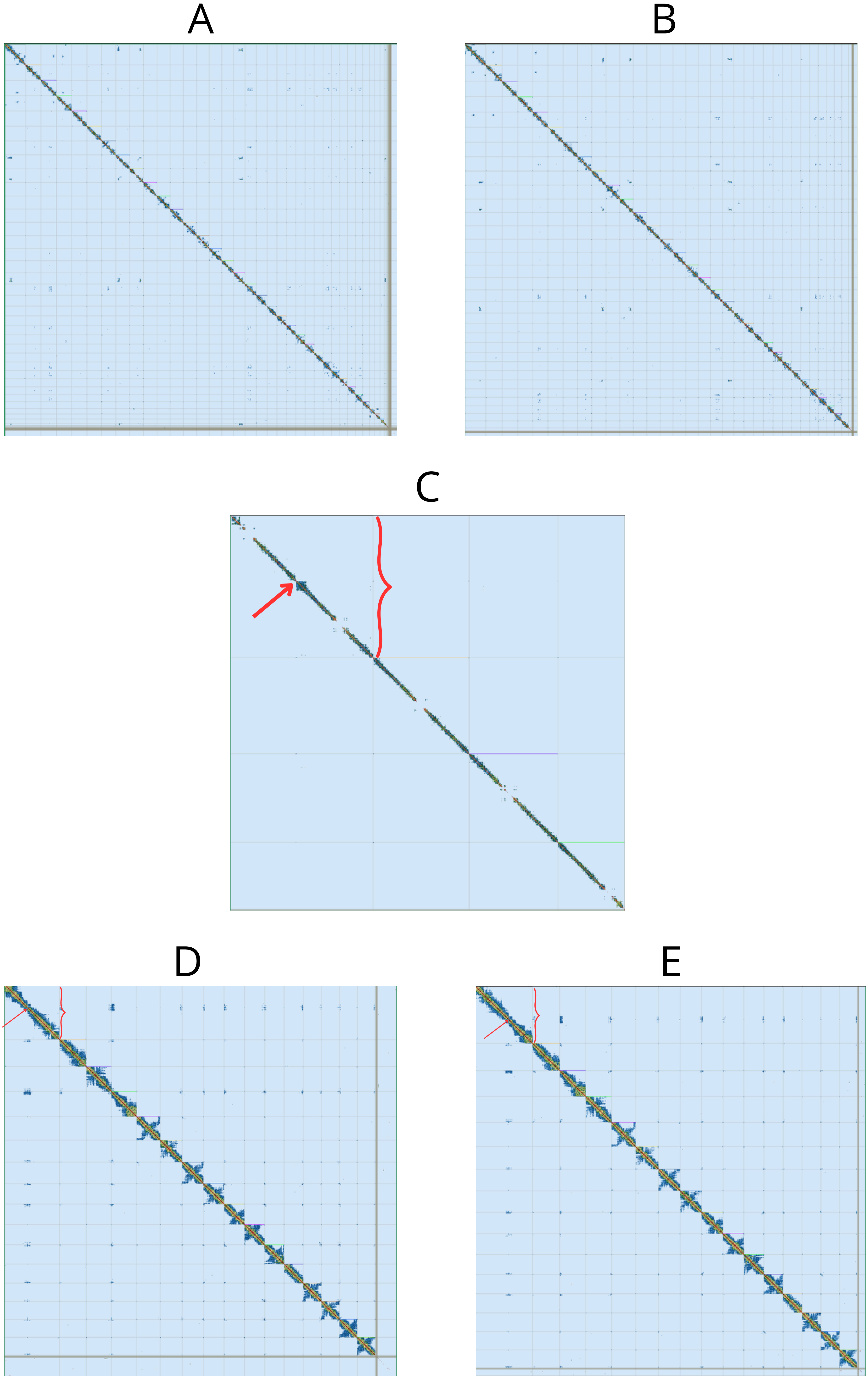

Figure S17. Hi-C contact maps for *R. irregularis* hap1 (A) and hap2 (B), *A. thaliana* (C), and *M. domestica* hap1 (D) and hap2 (E). The red curly brackets highlight the scaffolds that were incorrectly joined in *A. thaliana* and *M. domestica*, while the red arrows indicate the positions where there should have been a break between the two chromosome-scale scaffolds.
