# Supplementary material for: Colora: a Snakemake workflow for complete chromosome-scale de novo genome assembly
Source: Bioinformatics. 2025 Apr 16;41(5):btaf175. doi: 10.1093/bioinformatics/btaf175 (PMC12065627; doi:10.1093/bioinformatics/btaf175)

NanoPlot reports

Summary statistics

**General summary**	
**Mean read length**	12,812.1
**Mean read quality**	21.0
**Median read length**	13,219.0
**Median read quality**	30.6
**Number of reads**	2,341,873.0
**Read length N50**	13,983.0
**STDEV read length**	4,115.0
**Total bases**	30,004,306,961.0
**Number, percentage and megabases of reads above quality cutoffs**	
**>Q5**	2341873 (100.0%) 30004.3Mb
**>Q7**	2340830 (100.0%) 29973.2Mb
**>Q10**	2327686 (99.4%) 29711.5Mb
**>Q12**	2303673 (98.4%) 29357.2Mb
**>Q15**	2206465 (94.2%) 28008.5Mb
**Top 5 highest mean basecall quality scores and their read lengths**	
**1**	93.0 (5864)
**2**	93.0 (1928)
**3**	93.0 (3567)
**4**	93.0 (2927)
**5**	93.0 (1736)
**Top 5 longest reads and their mean basecall quality score**	
**1**	132789 (7.1)
**2**	128512 (6.1)
**3**	123726 (9.0)
**4**	119378 (6.3)
**5**	116180 (6.7)

Plots

Weighted histogram of read lengths -

Weighted histogram of read lengths

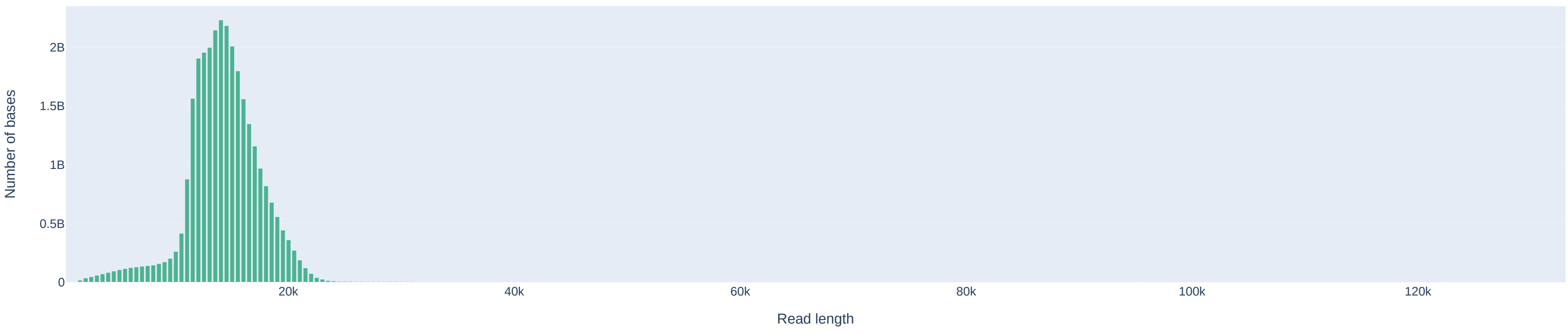

Weighted histogram of read lengths after log transformation -

Weighted histogram of read lengths after log transformation

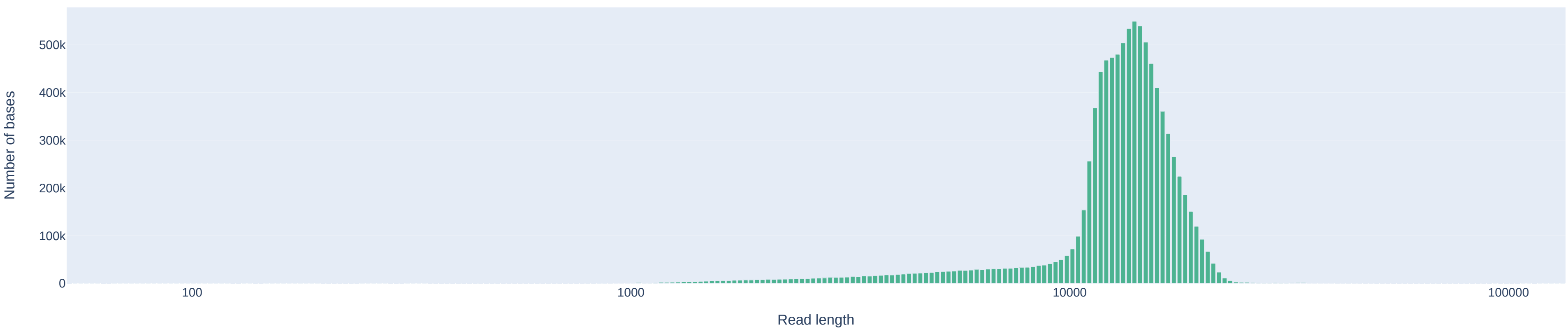

Non weighted histogram of read lengths -

Non weighted histogram of read lengths

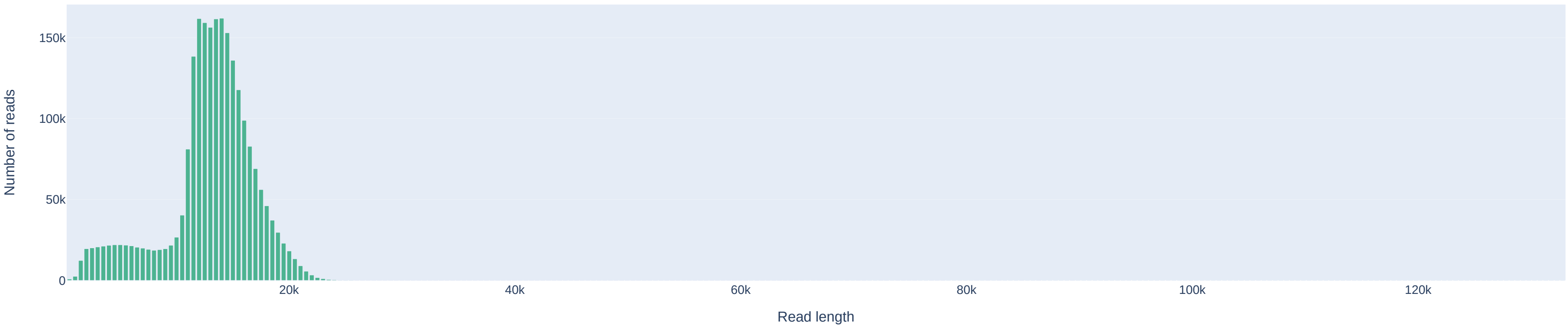

Non weighted histogram of read lengths after log transformation

Non weighted histogram of read lengths after log transformation

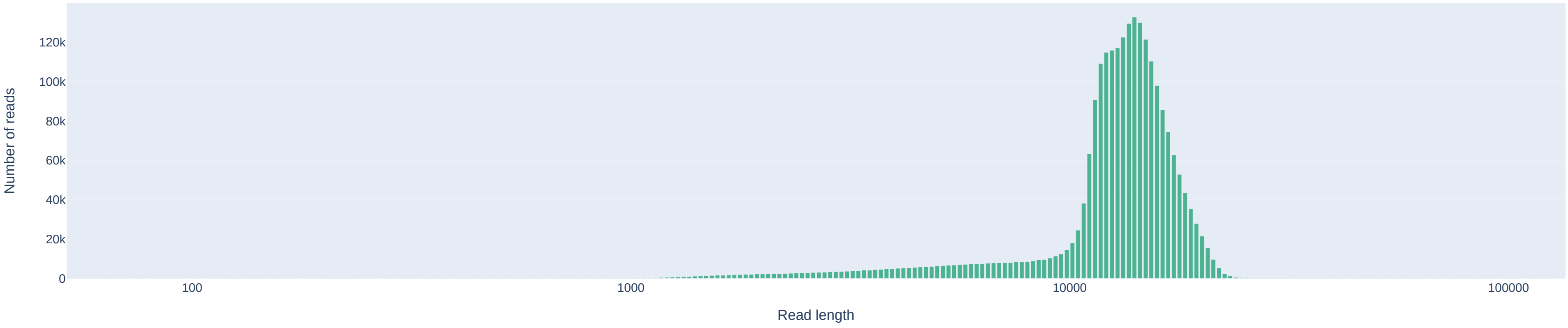

Yield by length

Yield by length

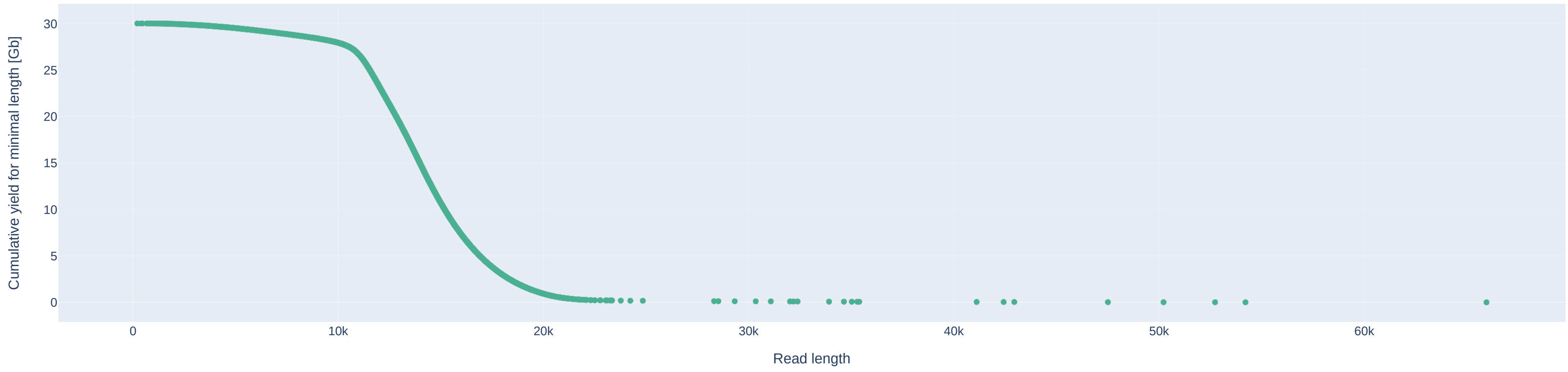

Read lengths vs Average read quality plot using dots

Read lengths vs Average read quality plot using dots

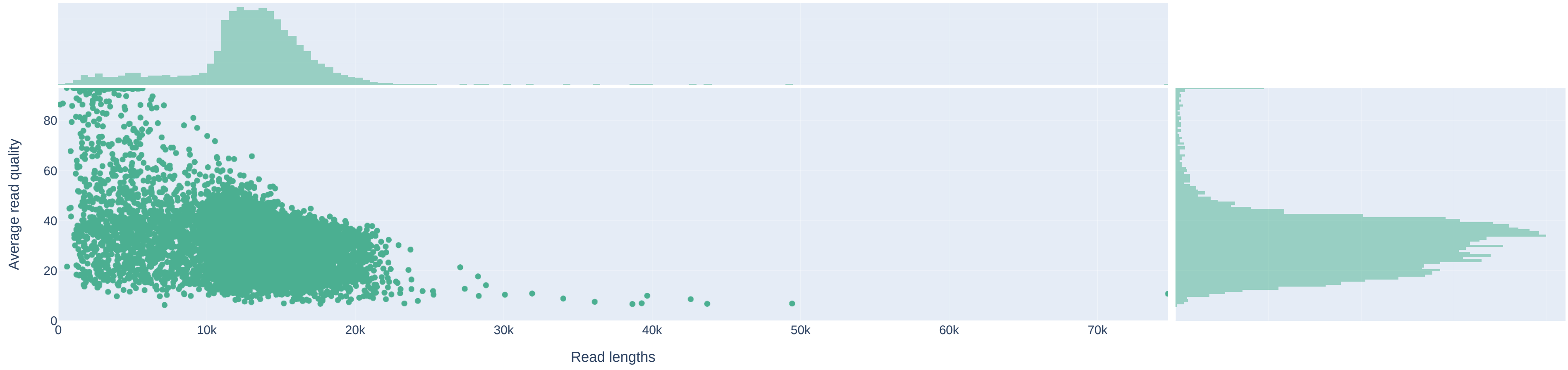

Read lengths vs Average read quality plot using dots after log transformation of read lengths

Read lengths vs Average read quality plot using dots after log transformation of read lengths

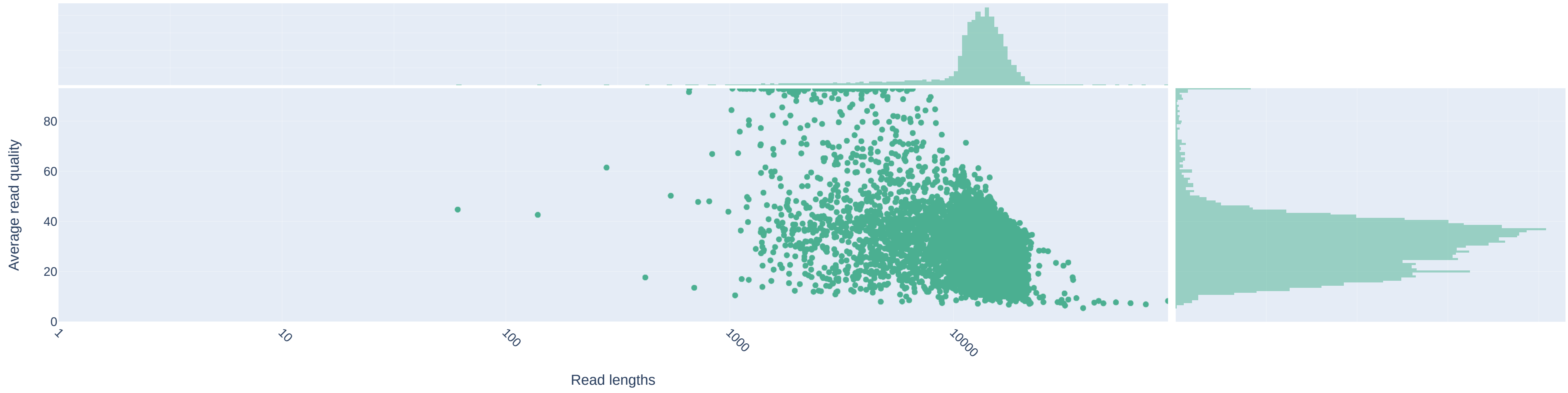

Supplement: btaf175_Supplementary_Data [file btaf175_supplementary_data.zip › Additional_files/S4_NanoPlot_R.irregularis.pdf]
