# Supplementary material for: Colora: a Snakemake workflow for complete chromosome-scale de novo genome assembly
Source: Bioinformatics. 2025 Apr 16;41(5):btaf175. doi: 10.1093/bioinformatics/btaf175 (PMC12065627; doi:10.1093/bioinformatics/btaf175)

Summary Statistics | Plots | Report issue on Github

NanoPlot reports

Summary statistics

General summary	
**Mean read length**	15,094.4
**Mean read quality**	27.1
**Median read length**	14,936.0
**Median read quality**	31.4
**Number of reads**	1,517,433.0
**Read length N50**	15,424.0
**STDEV read length**	2,661.7
**Total bases**	22,904,700,074.0
**Number, percentage and megabases of reads above quality cutoffs**	
**>Q5**	1517433 (100.0%) 22904.7Mb
**>Q7**	1517433 (100.0%) 22904.7Mb
**>Q10**	1517433 (100.0%) 22904.7Mb
**>Q12**	1517433 (100.0%) 22904.7Mb
**>Q15**	1517433 (100.0%) 22904.7Mb
**Top 5 highest mean basecall quality scores and their read lengths**	
**1**	93.0 (89)
**2**	93.0 (1825)
**3**	93.0 (3415)
**4**	93.0 (1581)
**5**	93.0 (52)
**Top 5 longest reads and their mean basecall quality score**	
**1**	45035 (22.6)
**2**	43917 (21.8)
**3**	43745 (20.5)
**4**	43682 (23.6)
**5**	43626 (22.0)

Plots

Weighted histogram of read lengths -

Weighted histogram of read lengths

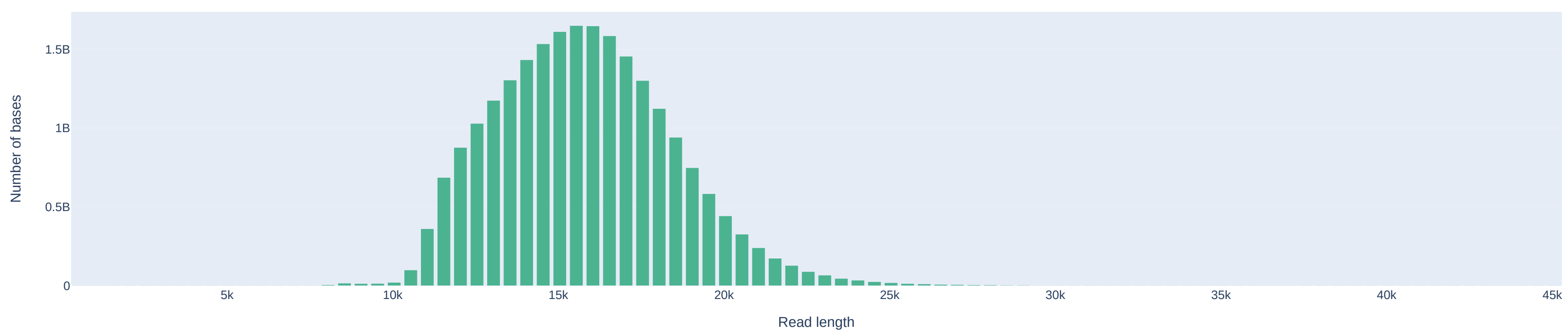

Weighted histogram of read lengths after log transformation -

Weighted histogram of read lengths after log transformation

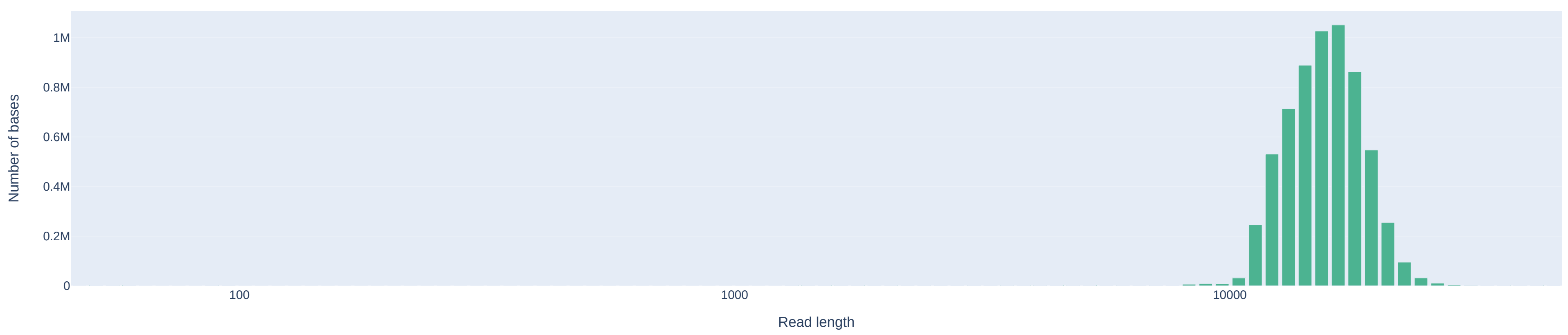

Non weighted histogram of read lengths -

Non weighted histogram of read lengths

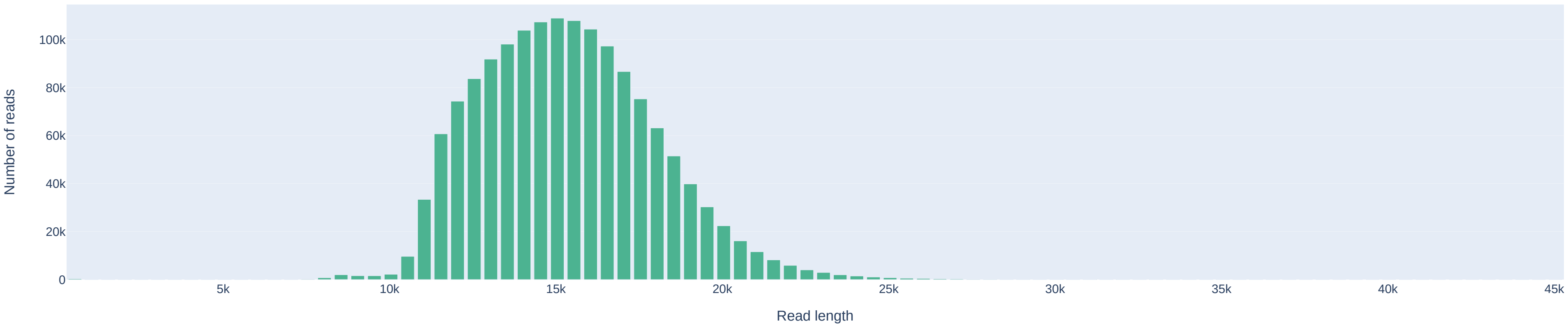

Non weighted histogram of read lengths after log transformation

-

Non weighted histogram of read lengths after log transformation

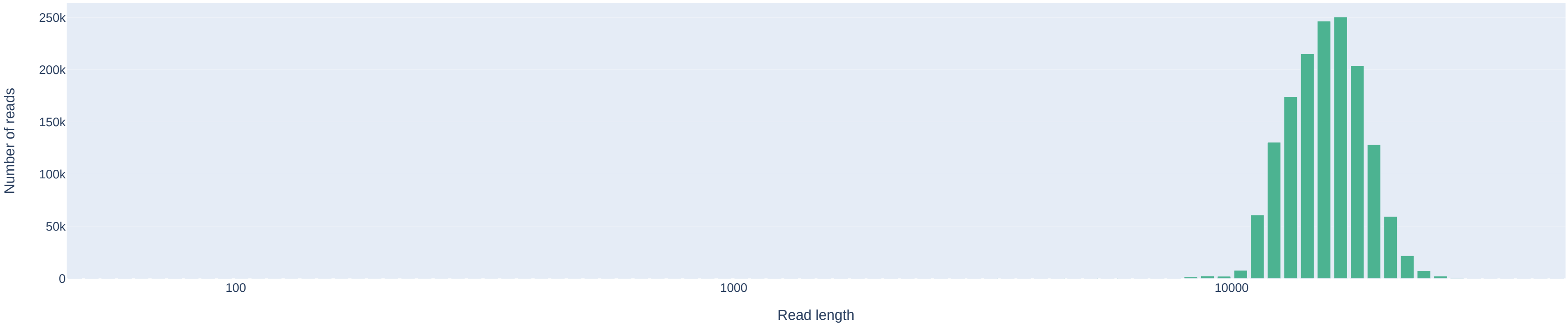

Yield by length

-

Yield by length

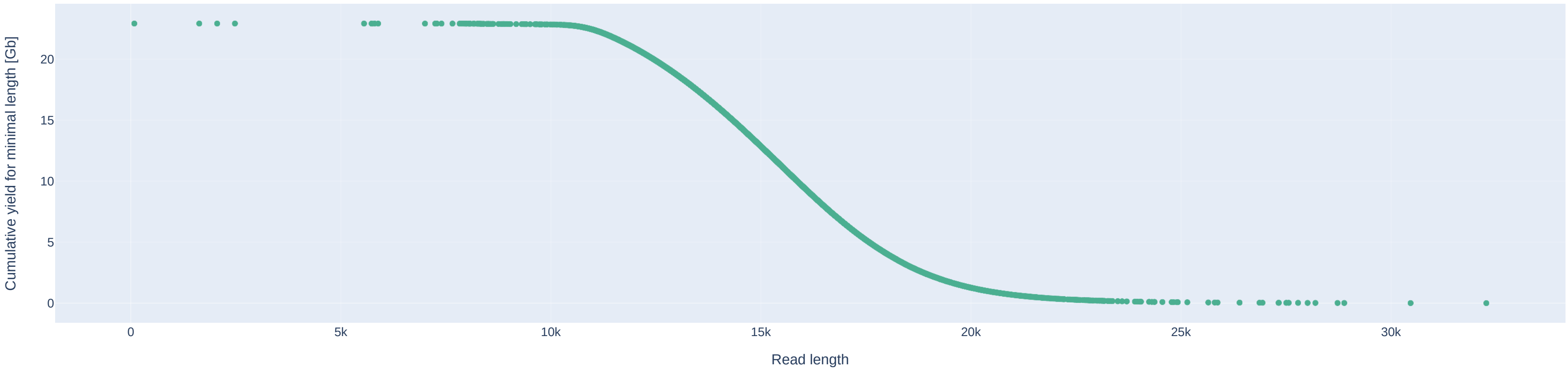

Read lengths vs Average read quality plot using dots

-

Read lengths vs Average read quality plot using dots

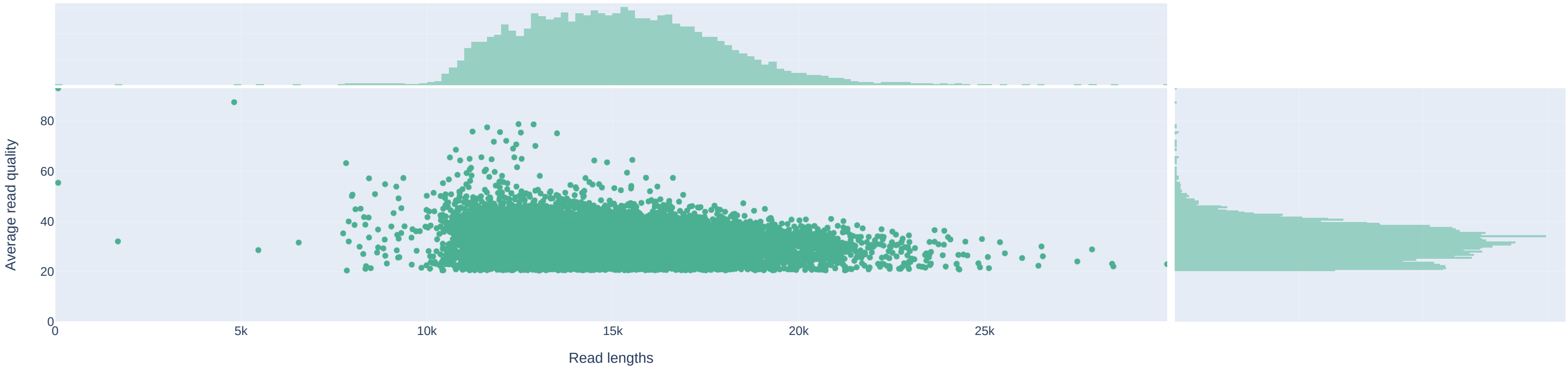

Read lengths vs Average read quality plot using dots after log transformation of read lengths

-

Read lengths vs Average read quality plot using dots after log transformation of read lengths

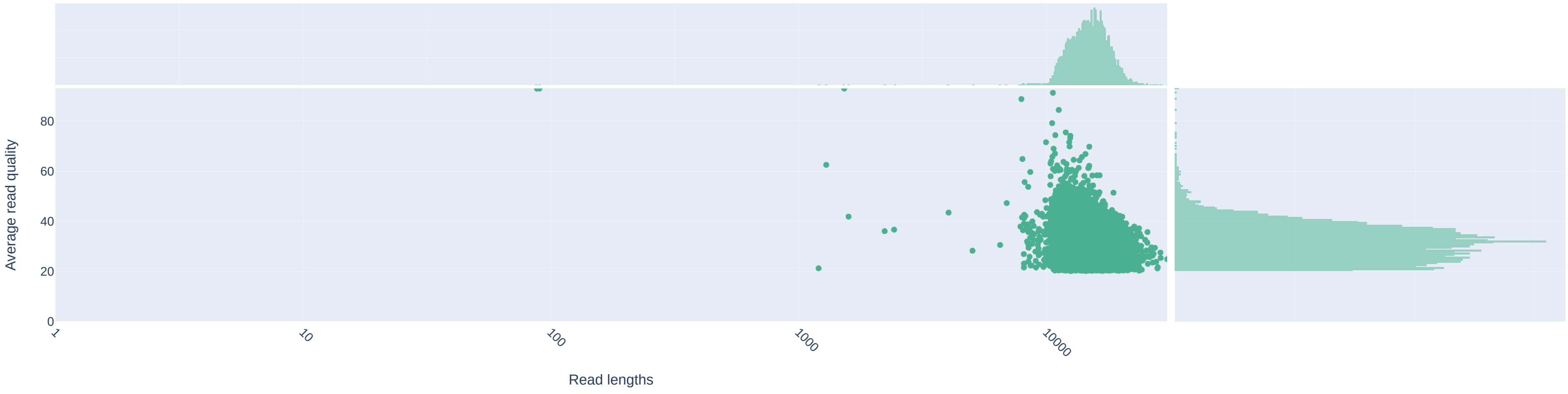

Supplement: btaf175_Supplementary_Data [file btaf175_supplementary_data.zip › Additional_files/S5_NanoPlot_A.thaliana.pdf]
