# Supplementary material for: Colora: a Snakemake workflow for complete chromosome-scale de novo genome assembly
Source: Bioinformatics. 2025 Apr 16;41(5):btaf175. doi: 10.1093/bioinformatics/btaf175 (PMC12065627; doi:10.1093/bioinformatics/btaf175)

NanoPlot reports

Summary statistics

General summary	
Mean read length	18,541.3
Mean read quality	11.1
Median read length	7,818.0
Median read quality	11.2
Number of reads	3,064,191.0
Read length N50	46,452.0
STDEV read length	26,536.0
Total bases	56,814,196,989.0
Number, percentage and megabases of reads above quality cutoffs	
>Q5	3064191 (100.0%) 56814.2Mb
>Q7	3064123 (100.0%) 56814.2Mb
>Q10	2168595 (70.8%) 40456.1Mb
>Q12	1055916 (34.5%) 19383.7Mb
>Q15	6640 (0.2%) 12.3Mb
Top 5 highest mean basecall quality scores and their read lengths	
1	21.0 (1)
2	19.0 (1)
3	19.0 (1)
4	19.0 (1)
5	18.9 (358)
Top 5 longest reads and their mean basecall quality score	
1	495032 (12.4)
2	457760 (8.7)
3	439434 (9.1)
4	438143 (8.7)
5	431286 (9.7)

Plots

Weighted histogram of read lengths -

Weighted histogram of read lengths

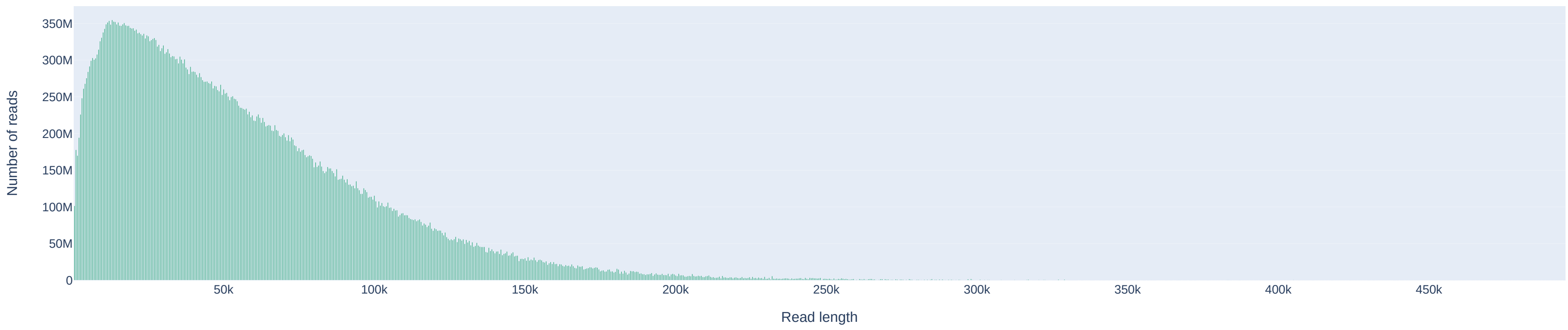

Weighted histogram of read lengths after log transformation -

Weighted histogram of read lengths after log transformation

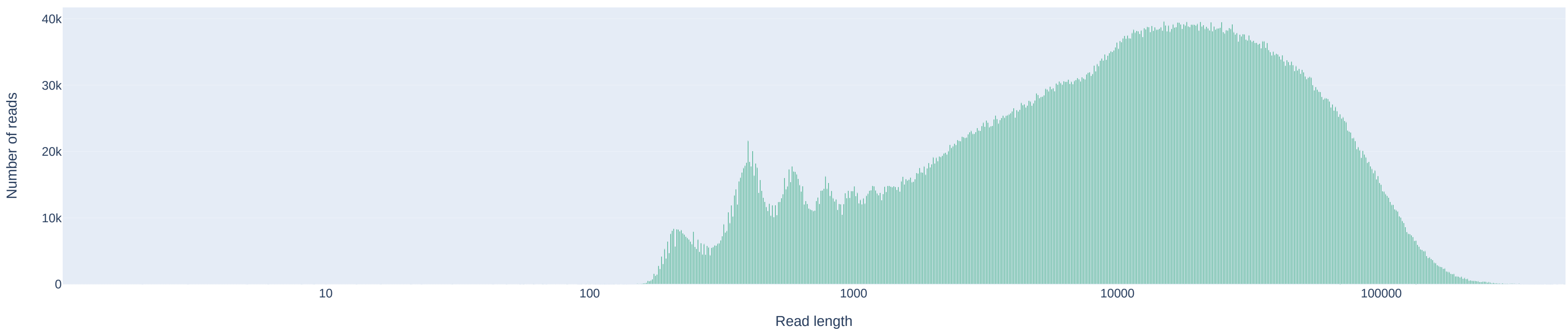

Non weighted histogram of read lengths -

Non weighted histogram of read lengths

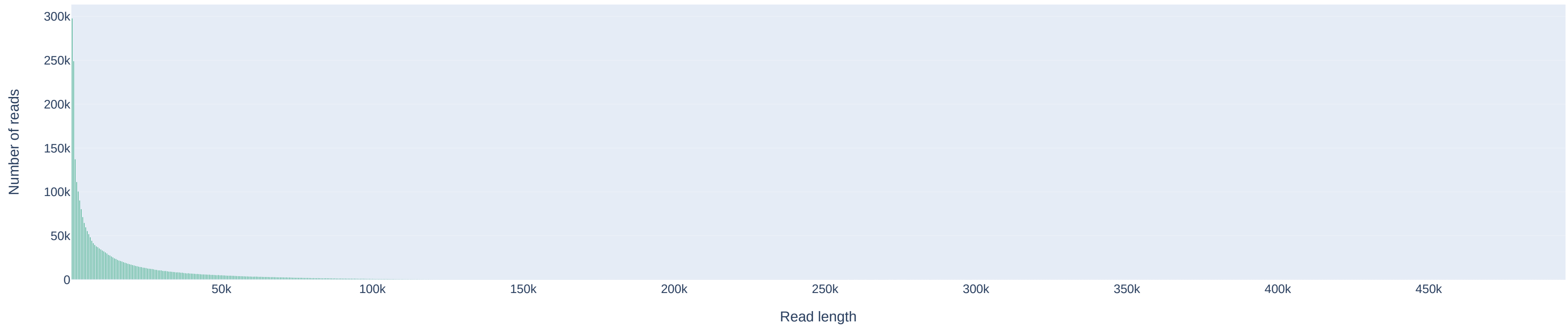

Non weighted histogram of read lengths after log transformation

Non weighted histogram of read lengths after log transformation

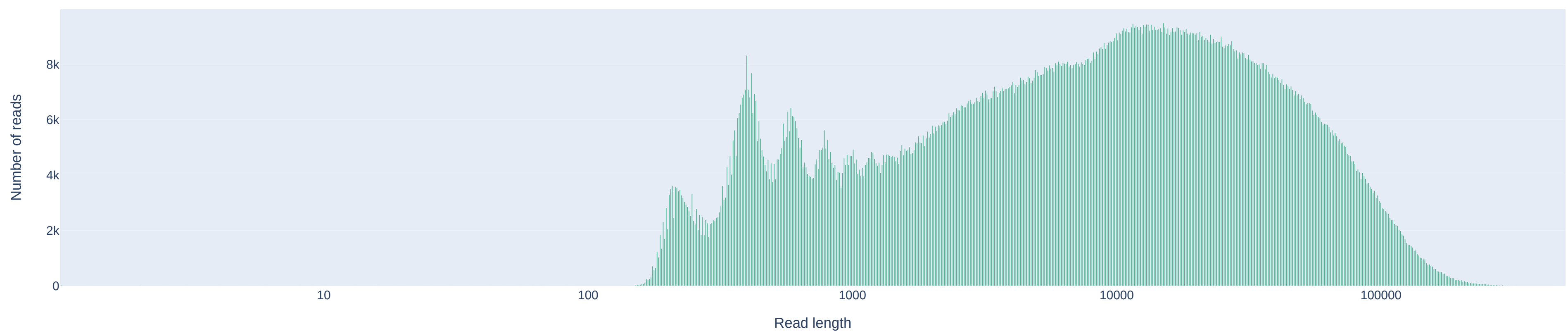

Yield by length

Yield by length

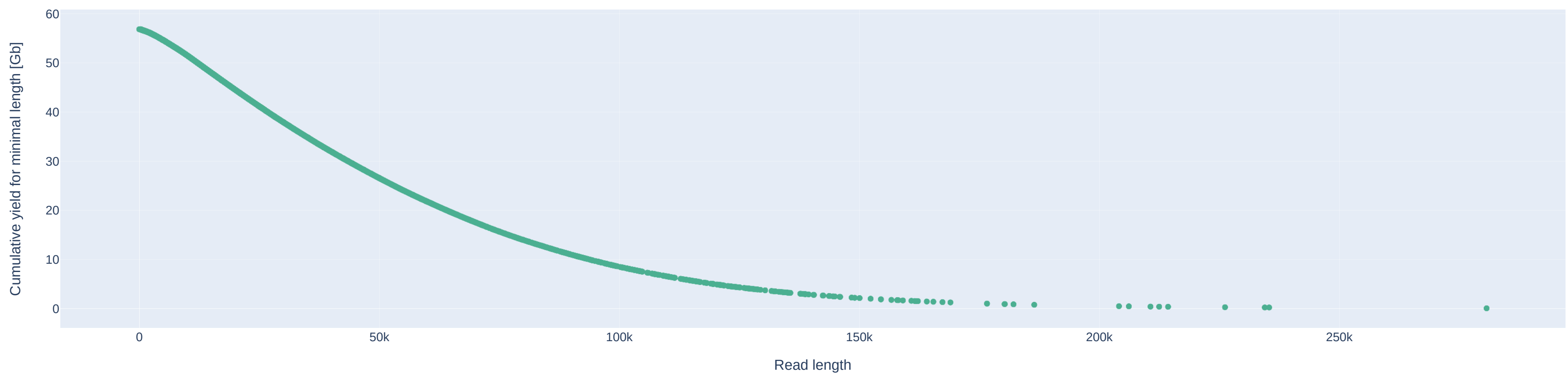

Read lengths vs Average read quality plot using dots

Read lengths vs Average read quality plot using dots

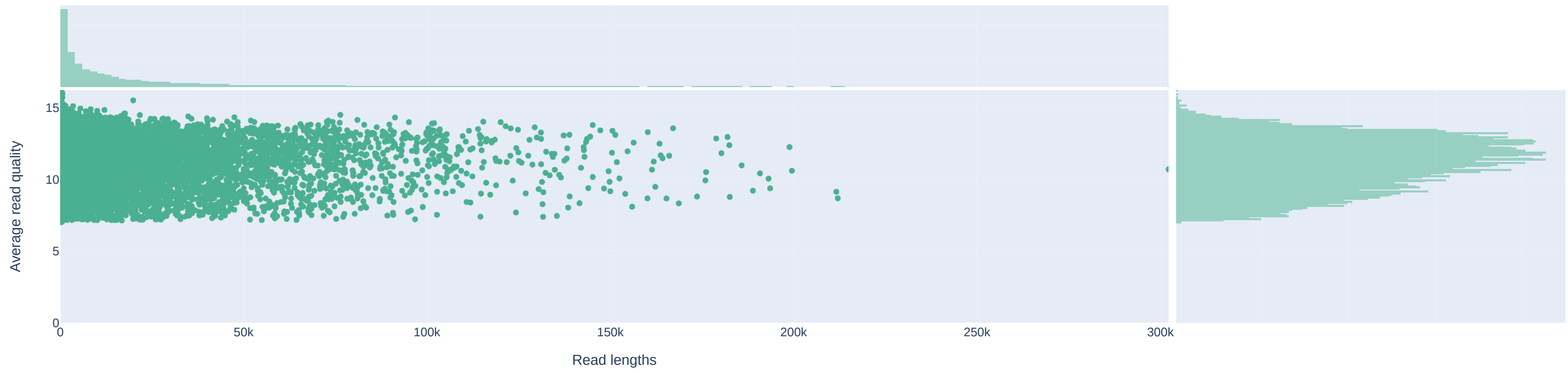

Supplement: btaf175_Supplementary_Data [file btaf175_supplementary_data.zip › Additional_files/S10_NanoPlot_A.thaliana_ONT.pdf]
