# Supplementary material for: Colora: a Snakemake workflow for complete chromosome-scale de novo genome assembly
Source: Bioinformatics. 2025 Apr 16;41(5):btaf175. doi: 10.1093/bioinformatics/btaf175 (PMC12065627; doi:10.1093/bioinformatics/btaf175)
Supplement: btaf175_Supplementary_Data [file btaf175_supplementary_data.zip › Additional_files/S8_fastp_A.thaliana.pdf]

fastp report

Summary

General

**fastp version:**	0.23.4 (https://github.com/OpenGene/fastp)
**sequencing:**	paired end (150 cycles + 150 cycles)
**mean length before filtering:**	150bp, 150bp
**mean length after filtering:**	148bp, 148bp
**duplication rate:**	9.464361%
**Insert size peak:**	269

Before filtering

**total reads:**	140.957500 M
**total bases:**	21.143625 G
**Q20 bases:**	20.447749 G (96.708815%)
**Q30 bases:**	19.262000 G (91.100747%)
**GC content:**	39.431368%

After filtering

**total reads:**	140.120386 M
**total bases:**	20.871882 G
**Q20 bases:**	20.234323 G (96.945371%)
**Q30 bases:**	19.079098 G (91.410531%)
**GC content:**	39.356446%

Filtering result

**reads passed filters:**	140.120386 M (99.406123%)
**reads with low quality:**	822.994000 K (0.583860%)
**reads with too many N:**	360 (0.000255%)
**reads too short:**	13.760000 K (0.009762%)

Adapters

Adapter or bad ligation of read1

The input has little adapter percentage (~0.663794%), probably it's trimmed before.

Sequence	Occurrences
A	44602
AG	39964
AGA	38018
AGAT	37142
AGATC	34863
AGATCG	34099
AGATCGG	33010
AGATCGGA	32658
AGATCGGAA	31158
AGATCGGAAG	31217
AGATCGGAAGA	30159
AGATCGGAAGAG	29458
AGATCGGAAGAGC	28073
AGATCGGAAGAGCA	27423
AGATCGGAAGAGCAC	26519
AGATCGGAAGAGCACA	25496
AGATCGGAAGAGCACAC	24565

AGATCGGAAGAGCACACG	24403
AGATCGGAAGAGCACACGT	23364
AGATCGGAAGAGCACACGTC	23233
AGATCGGAAGAGCACACGTCT	23077
AGATCGGAAGAGCACACGTCTG	22236
AGATCGGAAGAGCACACGTCTGA	21548
AGATCGGAAGAGCACACGTCTGAACTCCAGTCACGAGATTCCATCTCGTATGCCGTCTTCTGCTTGAAAA	29027
other adapter sequences	1419410

Adapter or bad ligation of read2

The input has little adapter percentage (~0.674501%), probably it's trimmed before.

Sequence	Occurrences
A	45050
AG	40601
AGA	38484
AGAT	37579
AGATC	35307
AGATCG	34507
AGATCGG	33528
AGATCGGA	33053
AGATCGGAA	31476
AGATCGGAAG	31588
AGATCGGAAGA	30418
AGATCGGAAGAG	29773
AGATCGGAAGAGC	28051
AGATCGGAAGAGCG	27669
AGATCGGAAGAGCGT	26690
AGATCGGAAGAGCGTC	25571
AGATCGGAAGAGCGTCG	24541
AGATCGGAAGAGCGTCGT	24065
AGATCGGAAGAGCGTCGTG	23189
AGATCGGAAGAGCGTCGTGT	22876
AGATCGGAAGAGCGTCGTGTA	22524
AGATCGGAAGAGCGTCGTGTAG	21782
AGATCGGAAGAGCGTCGTGTAGGGAAAGAGTGTTCAGAGCCGTGTAGATCTCGGTGGTCGCCGTATCATTAAAAA	25870
other adapter sequences	1441969

Insert size estimation

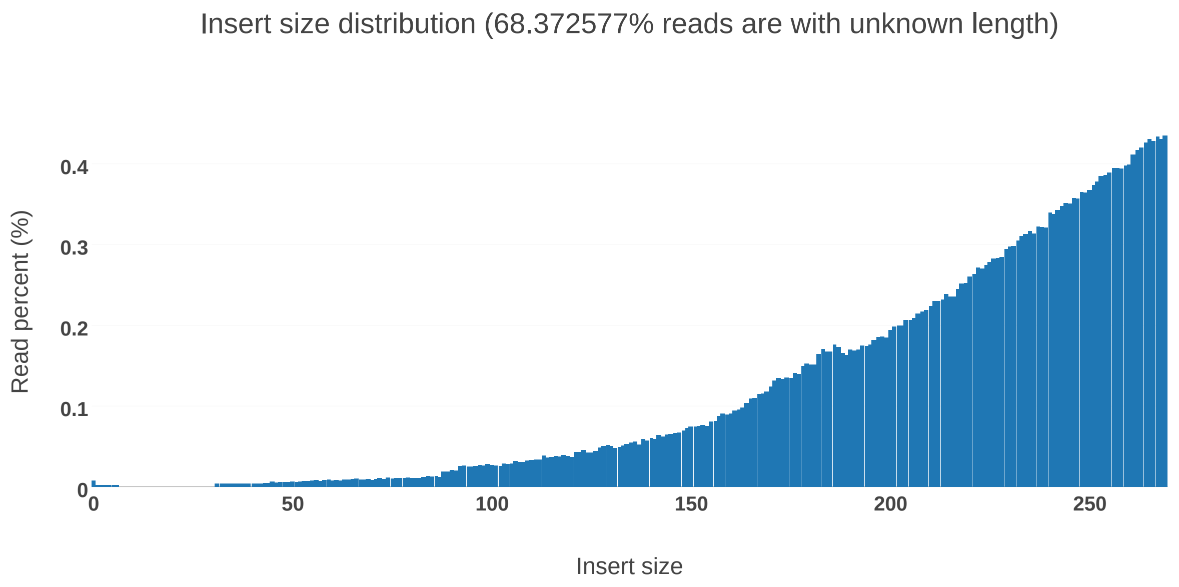

This estimation is based on paired-end overlap analysis, and there are 68.372577% reads found not overlapped.
The nonoverlapped read pairs may have insert size <30 or >270, or contain too much sequencing errors to be detected as overlapped.

Before filtering

Before filtering: read1: quality

Value of each position will be shown on mouse over.

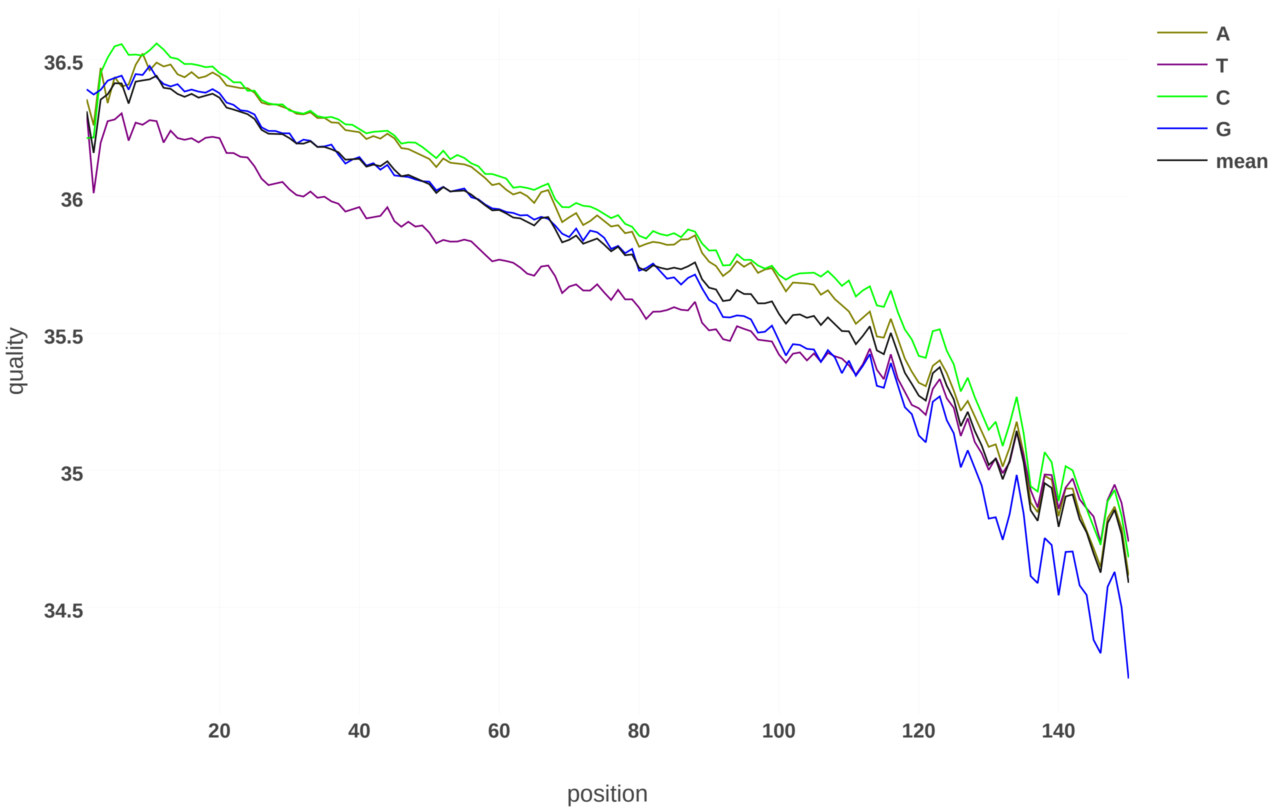

Before filtering: read1: base contents

Value of each position will be shown on mouse over.

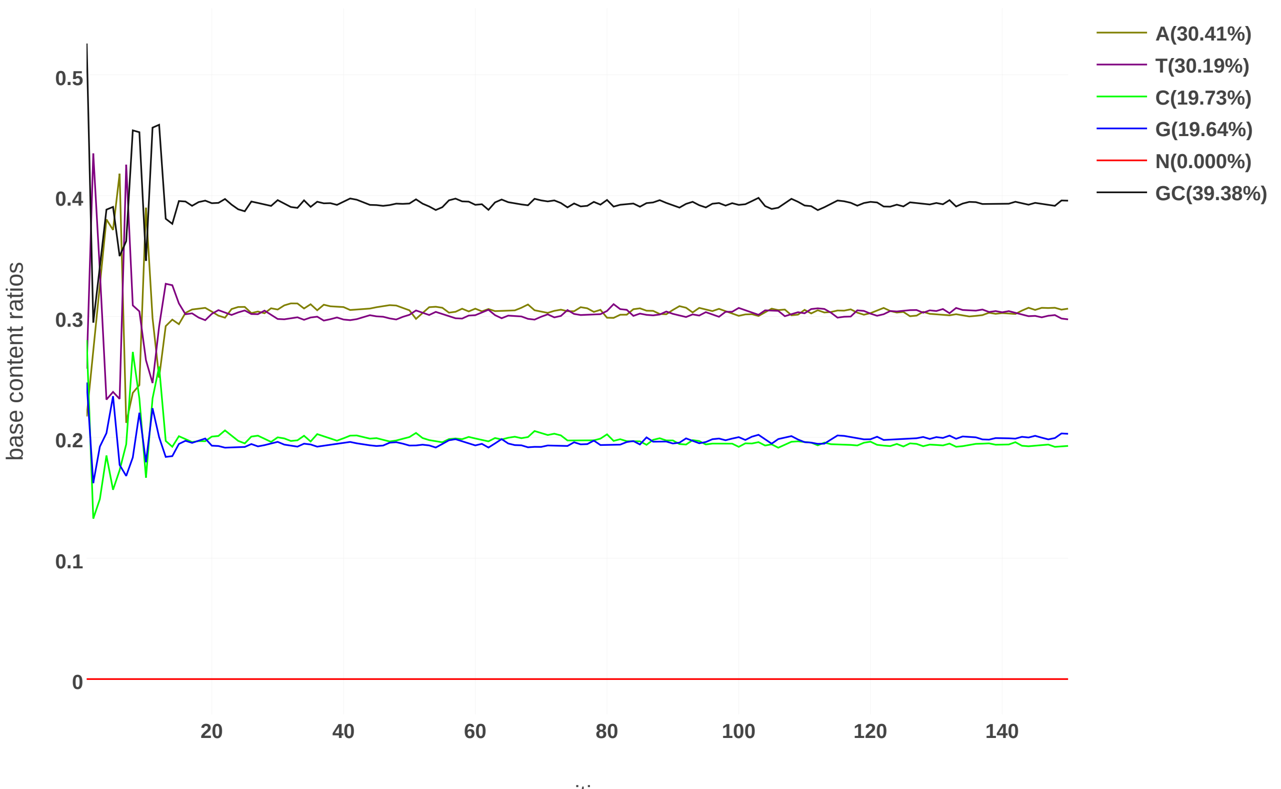

position

Before filtering: read1: KMER counting

Darker background means larger counts. The count will be shown on mouse over.

	AA	AT	AC	AG	TA	TT	TC	TG	CA	CT	CC	CG	GA	GT	GC	GG
AAA	AAAAA	AAAAT	AAAAC	AAAAG	AAATA	AAATT	AAATC	AAATG	AAACA	AAACT	AAACC	AAACG	AAAGA	AAAGT	AAAGC	AAAGG
AAT	AATAA	AATAT	AATAC	AATAG	AATTA	AATTT	AATTC	AATTG	AATCA	AATCT	AATCC	AATCG	AATGA	AATGT	AATGC	AATGG
AAC	AACAA	AACAT	AACAC	AACAG	AACTA	AACTT	AACTC	AACTG	AACCA	AACCT	AACCC	AACCG	AACGA	AACGT	AACGC	AACGG
AAG	AAGAA	AAGAT	AAGAC	AAGAG	AAGTA	AAGTT	AAGTC	AAGTG	AAGCA	AAGCT	AAGCC	AAGCG	AAGGA	AAGGT	AAGGC	AAGGG
ATA	ATAAA	ATAAT	ATAAC	ATAAG	ATATA	ATATT	ATATC	ATATG	ATACA	ATACT	ATACC	ATACG	ATAGA	ATAGT	ATAGC	ATAGG
ATT	ATTAA	ATTAT	ATTAC	ATTAG	ATTTA	ATTTT	ATTTC	ATTTG	ATTCA	ATTCT	ATTCC	ATTCG	ATTGA	ATTGT	ATTGC	ATTGG
ATC	ATCAA	ATCAT	ATCAC	ATCAG	ATCTA	ATCTT	ATCTC	ATCTG	ATCCA	ATCCT	ATCCC	ATCCG	ATCGA	ATCGT	ATCGC	ATCGG
ATG	ATGAA	ATGAT	ATGAC	ATGAG	ATGTA	ATGTT	ATGTC	ATGTG	ATGCA	ATGCT	ATGCC	ATGCG	ATGGA	ATGGT	ATGGC	ATGGG
ACA	ACAAA	ACAAT	ACAAC	ACAAG	ACATA	ACATT	ACATC	ACATG	ACACA	ACACT	ACACC	ACACG	ACAGA	ACAGT	ACAGC	ACAGG
ACT	ACTAA	ACTAT	ACTAC	ACTAG	ACTTA	ACTTT	ACTTC	ACTTG	ACTCA	ACTCT	ACTCC	ACTCG	ACTGA	ACTGT	ACTGC	ACTGG
ACC	ACCAA	ACCAT	ACCAC	ACCAG	ACCTA	ACCTT	ACCTC	ACCTG	ACCCA	ACCCT	ACCCC	ACCCG	ACCGA	ACCGT	ACCGC	ACCGG
ACG	ACGAA	ACGAT	ACGAC	ACGAG	ACGTA	ACGTT	ACGTC	ACGTG	ACGCA	ACGCT	ACGCC	ACGCG	ACGGA	ACGGT	ACGGC	ACGGG
AGA	AGAAA	AGAAT	AGAAC	AGAAG	AGATA	AGATT	AGATC	AGATG	AGACA	AGACT	AGACC	AGACG	AGAGA	AGAGT	AGAGC	AGAGG
AGT	AGTAA	AGTAT	AGTAC	AGTAG	AGTTA	AGTTT	AGTTC	AGTTG	AGTCA	AGTCT	AGTCC	AGTCG	AGTGA	AGTGT	AGTGC	AGTGG
AGC	AGCAA	AGCAT	AGCAC	AGCAG	AGCTA	AGCTT	AGCTC	AGCTG	AGCCA	AGCCT	AGCCC	AGCCG	AGCGA	AGCGT	AGCGC	AGCGG
AGG	AGGAA	AGGAT	AGGAC	AGGAG	AGGTA	AGGTT	AGGTC	AGGTG	AGGCA	AGGCT	AGGCC	AGGCG	AGGGA	AGGGT	AGGGC	AGGGG
TAA	TAAAA	TAAAT	TAAAC	TAAAG	TAATA	TAATT	TAATC	TAATG	TAACA	TAACT	TAACC	TAACG	TAAGA	TAAGT	TAAGC	TAAGG
TAT	TATAA	TATAT	TATAC	TATAG	TATTA	TATTT	TATTC	TATTG	TATCA	TATCT	TATCC	TATCG	TATGA	TATGT	TATGC	TATGG
TAC	TACAA	TACAT	TACAC	TACAG	TACTA	TACTT	TACTC	TACTG	TACCA	TACCT	TACCC	TACCG	TACGA	TACGT	TACGC	TACGG
TAG	TAGAA	TAGAT	TAGAC	TAGAG	TAGTA	TAGTT	TAGTC	TAGTG	TAGCA	TAGCT	TAGCC	TAGCG	TAGGA	TAGGT	TAGGC	TAGGG
TTA	TTAAA	TTAAT	TTAAC	TTAAG	TTATA	TTATT	TTATC	TTATG	TTACA	TTACT	TTACC	TTACG	TTAGA	TTAGT	TTAGC	TTAGG
TTT	TTTAA	TTTAT	TTTAC	TTTAG	TTTTA	TTTTT	TTTTC	TTTTG	TTTCA	TTTCT	TTTCC	TTTCG	TTTGA	TTTGT	TTTGC	TTTGG
TTC	TTCAA	TTCAT	TTCAC	TTCAG	TTCTA	TTCTT	TTCTC	TTCTG	TTCCA	TTCCT	TTCCC	TTCCG	TTCGA	TTCGT	TTCGC	TTCGG
TTG	TTGAA	TTGAT	TTGAC	TTGAG	TTGTA	TTGTT	TTGTC	TTGTG	TTGCA	TTGCT	TTGCC	TTGCG	TTGGA	TTGGT	TTGGC	TTGGG
TCA	TCAAA	TCAAT	TCAAC	TCAAG	TCATA	TCATT	TCATC	TCATG	TCACA	TCACT	TCACC	TCACG	TCAGA	TCAGT	TCAGC	TCAGG
TCT	TCTAA	TCTAT	TCTAC	TCTAG	TCTTA	TCTTT	TCTTC	TCTTG	TCTCA	TCTCT	TCTCC	TCTCG	TCTGA	TCTGT	TCTGC	TCTGG
TCC	TCCAA	TCCAT	TCCAC	TCCAG	TCCTA	TCCTT	TCCTC	TCCTG	TCCCA	TCCCT	TCCCC	TCCCG	TCCGA	TCCGT	TCCGC	TCCGG
TCG	TCGAA	TCGAT	TCGAC	TCGAG	TCGTA	TCGTT	TCGTC	TCGTG	TCGCA	TCGCT	TCGCC	TCGCG	TCGGA	TCGGT	TCGGC	TCGGG
TGA	TGAAA	TGAAT	TGAAC	TGAAG	TGATA	TGATT	TGATC	TGATG	TGACA	TGACT	TGACC	TGACG	TGAGA	TGAGT	TGAGC	TGAGG
TGT	TGTAA	TGTAT	TGTAC	TGTAG	TGTTA	TGTTT	TGTTC	TGTTG	TGTCA	TGTCT	TGTCC	TGTCG	TGTGA	TGTGT	TGTGC	TGTGG
TGC	TGCAA	TGCAT	TGCAC	TGCAG	TGCTA	TGCTT	TGCTC	TGCTG	TGCCA	TGCCT	TGCCC	TGCCG	TGCGA	TGCGT	TGCGC	TGCGG
TGG	TGGAA	TGGAT	TGGAC	TGGAG	TGGTA	TGGTT	TGGTC	TGGTG	TGGCA	TGGCT	TGGCC	TGGCG	TGGGA	TGGGT	TGGGC	TGGGG
CAA	CAAAA	CAAAT	CAAAC	CAAAG	CAATA	CAATT	CAATC	CAATG	CAACA	CAACT	CAACC	CAACG	CAAGA	CAAGT	CAAGC	CAAGG
CAT	CATAA	CATAT	CATAC	CATAG	CATTA	CATTT	CATTC	CATTG	CATCA	CATCT	CATCC	CATCG	CATGA	CATGT	CATGC	CATGG
CAC	CACAA	CACAT	CACAC	CACAG	CACTA	CACTT	CACTC	CACTG	CACCA	CACCT	CACCC	CACCG	CACGA	CACGT	CACGC	CACGG
CAG	CAGAA	CAGAT	CAGAC	CAGAG	CAGTA	CAGTT	CAGTC	CAGTG	CAGCA	CAGCT	CAGCC	CAGCG	CAGGA	CAGGT	CAGGC	CAGGG
CTA	CTAAA	CTAAT	CTAAC	CTAAG	CTATA	CTATT	CTATC	CTATG	CTACA	CTACT	CTACC	CTACG	CTAGA	CTAGT	CTAGC	CTAGG
CTT	CTTAA	CTTAT	CTTAC	CTTAG	CTTTA	CTTTT	CTTTC	CTTTG	CTTCA	CTTCT	CTTCC	CTTCG	CTTGA	CTTGT	CTTGC	CTTGG
CTC	CTCAA	CTCAT	CTCAC	CTCAG	CTCTA	CTCTT	CTCTC	CTCTG	CTCCA	CTCCT	CTCCC	CTCCG	CTCGA	CTCGT	CTCGC	CTCGG
CTG	CTGAA	CTGAT	CTGAC	CTGAG	CTGTA	CTGTT	CTGTC	CTGTG	CTGCA	CTGCT	CTGCC	CTGCG	CTGGA	CTGGT	CTGGC	CTGGG
CCA	CCAAA	CCAAT	CCAAC	CCAAG	CCATA	CCATT	CCATC	CCATG	CCACA	CCACT	CCACC	CCACG	CCAGA	CCAGT	CCAGC	CCAGG
CCT	CCTAA	CCTAT	CCTAC	CCTAG	CCTTA	CCTTT	CCTTC	CCTTG	CCTCA	CCTCT	CCTCC	CCTCG	CCTGA	CCTGT	CCTGC	CCTGG
CCC	CCCAA	CCCAT	CCCAC	CCCAG	CCCTA	CCCTT	CCCTC	CCCTG	CCCCA	CCCCT	CCCCC	CCCCG	CCCGA	CCCGT	CCCGC	CCCGG
CCG	CCGAA	CCGAT	CCGAC	CCGAG	CCGTA	CCGTT	CCGTC	CCGTG	CCGCA	CCGCT	CCGCC	CCGCG	CCGGA	CCGGT	CCGGC	CCGGG
CGA	CGAAA	CGAAT	CGAAC	CGAAG	CGATA	CGATT	CGATC	CGATG	CGACA	CGACT	CGACC	CGACG	CGAGA	CGAGT	CGAGC	CGAGG
CGT	CGTAA	CGTAT	CGTAC	CGTAG	CGTTA	CGTTT	CGTTC	CGTTG	CGTCA	CGTCT	CGTCC	CGTCG	CGTGA	CGTGT	CGTGC	CGTGG
CGC	CGCAA	CGCAT	CGCAC	CGCAG	CGCTA	CGCTT	CGCTC	CGCTG	CGCCA	CGCCT	CGCCC	CGCCG	CGCGA	CGCGT	CGCGC	CGCGG
CGG	CGGAA	CGGAT	CGGAC	CGGAG	CGGTA	CGGTT	CGGTC	CGGTG	CGGCA	CGGCT	CGGCC	CGGCG	CGGGA	CGGGT	CGGGC	CGGGG
GAA	GAAAA	GAAAT	GAAAC	GAAAG	GAATA	GAATT	GAATC	GAATG	GAACA	GAACT	GAACC	GAACG	GAAGA	GAAGT	GAAGC	GAAGG
GAT	GATAA	GATAT	GATAC	GATAG	GATTA	GATTT	GATTC	GATTG	GATCA	GATCT	GATCC	GATCG	GATGA	GATGT	GATGC	GATGG
GAC	GACAA	GACAT	GACAC	GACAG	GACTA	GACTT	GACTC	GACTG	GACCA	GACCT	GACCC	GACCG	GACGA	GACGT	GACGC	GACGG
GAG	GAGAA	GAGAT	GAGAC	GAGAG	GAGTA	GAGTT	GAGTC	GAGTG	GAGCA	GAGCT	GAGCC	GAGCG	GAGGA	GAGGT	GAGGC	GAGGG
GTA	GTAAA	GTAAT	GTAAC	GTAAG	GTATA	GTATT	GTATC	GTATG	GTACA	GTACT	GTACC	GTACG	GTAGA	GTAGT	GTAGC	GTAGG
GTT	GTTAA	GTTAT	GTTAC	GTTAG	GTTTA	GTTTT	GTTTC	GTTTG	GTTCA	GTTCT	GTTCC	GTTCG	GTTGA	GTTGT	GTTGC	GTTGG
GTC	GTCAA	GTCAT	GTCAC	GTCAG	GTCTA	GTCTT	GTCTC	GTCTG	GTCCA	GTCCT	GTCCC	GTCCG	GTCGA	GTCGT	GTCGC	GTCGG
GTG	GTGAA	GTGAT	GTGAC	GTGAG	GTGTA	GTGTT	GTGTC	GTGTG	GTGCA	GTGCT	GTGCC	GTGCG	GTGGA	GTGGT	GTGGC	GTGGG
GCA	GCAAA	GCAAT	GCAAC	GCAAG	GCATA	GCATT	GCATC	GCATG	GCACA	GCACT	GCACC	GCACG	GCAGA	GCAGT	GCAGC	GCAGG
GCT	GCTAA	GCTAT	GCTAC	GCTAG	GCTTA	GCTTT	GCTTC	GCTTG	GCTCA	GCTCT	GCTCC	GCTCG	GCTGA	GCTGT	GCTGC	GCTGG
GCC	GCCAA	GCCAT	GCCAC	GCCAG	GCCTA	GCCTT	GCCTC	GCCTG	GCCCA	GCCCT	GCCCC	GCCCG	GCCGA	GCCGT	GCCGC	GCCGG
GCG	GCGAA	GCGAT	GCGAC	GCGAG	GCGTA	GCGTT	GCGTC	GCGTG	GCGCA	GCGCT	GCGCC	GCGCG	GCGGA	GCGGT	GCGGC	GCGGG
GGA	GGAAA	GGAAT	GGAAC	GGAAG	GGATA	GGATT	GGATC	GGATG	GGACA	GGACT	GGACC	GGACG	GGAGA	GGAGT	GGAGC	GGAGG
GGT	GGTAA	GGTAT	GGTAC	GGTAG	GGTTA	GGTTT	GGTTC	GGTTG	GGTCA	GGTCT	GGTCC	GGTCG	GGTGA	GGTGT	GGTGC	GGTGG
GGC	GGCAA	GGCAT	GGCAC	GGCAG	GGCTA	GGCTT	GGCTC	GGCTG	GGCCA	GGCCT	GGCCC	GGCCG	GGCGA	GGCGT	GGCGC	GGCGG
GGG	GGGAA	GGGAT	GGGAC	GGGAG	GGGTA	GGGTT	GGGTC	GGGTG	GGGCA	GGGCT	GGGCC	GGGCG	GGGGA	GGGGT	GGGGC	GGGGG

Before filtering: read1: overrepresented sequences

Sampling rate: 1 / 20

overrepresented sequence	count (% of bases)	distribution: cycle 1 ~ cycle 150
AAAAAAAAAA	59956 (0.113426%)	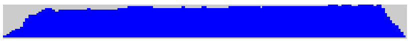
AAAAAGCAACGTCGCTATGAACGCTTGGCTGCCACAAGCCAGTTATCCCTGTGGTAACTTTTCTGACACCTCTAGCTTCAAATTCCGAAGGTCTAAAGGA	339 (0.006413%)	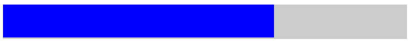
AAAAGATAACGCAGGTGTCCTAAGATGAGCTCAACGAGAACAGAAATCTCGTGTGGAACAAAAGGGTAAAAGCTCGTTTGATTCTGATTTTCAGTACGAA	12 (0.000227%)	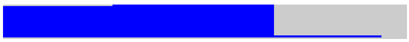
AAAAGCAACGTCGCTATGAACGCTTGGCTGCCACAAGCCAGTTATCCCTGTGGTAACTTTTCTGACACCTCTAGCTTCAAATTCCGAAGGTCTAAAGGAT	33 (0.000624%)	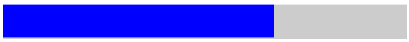
AAAAGCCTAAGTAGTGTTTCCTTGTTAGAAGACACAAAGCCAAAGACTCATATGGACTTTGGCTACACCATGAAAGCTTTGAGAAGCAAGAAGAAGGTTG	25 (0.000473%)	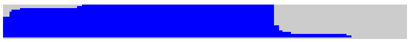
AAAATCAGAATCAAACGAGCTTTTACCCTTTTGTTCCACACGAGATTTCTGTTCTCGTTGAGCTCATCTTAGGACACCTGCGTTATCTTTTAACAGATGT	3 (0.000057%)	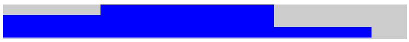
AAACCGCAACCGGATCTTAAAGGCGTAAGAATTGTATCCTTGTTAGAAGACACAAAGCCAAAGACTCATATGGACTTTGGCTACACCATGAAAGCTTTGA	74 (0.001400%)	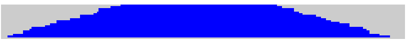
AAACGAGCTTTTACCCTTTTGTTCCACACGAGATTTCTGTTCTCGTTGAGCTCATCTTAGGACACCTGCGTTATCTTTTAACAGATGTGCCGCCCCAGCC	4 (0.000076%)	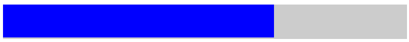
AAACTGCGAATGGCTCATTAAATCAGTTATAGTTTGTTTGATGGTAACTACTACTCGGATAACCGTAGTAATTCTAGAGCTAATACGTGCAACAAACCCC	4 (0.000076%)	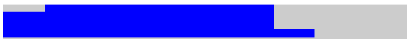
AAAGAAGACCCTGTTGAGCTTGACTCTAGTCCGACTTTGTGAAATGACTTGAGAGGTGTAGGATAAGTGGGAGCTTCGGCGCAAGTGAAATACCACTACT	15 (0.000284%)	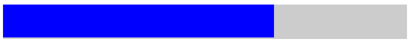
AAAGACTCATATGGACTTTGGCTACACCATGAAAGCTTTGAGAAGCAAGAAGAAGGTTGGTTAGTGTTTTGGAGTCGAATATGACTTGATGTCATGTGTA	90 (0.001703%)	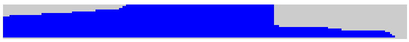
AAAGAGCCCGGTATTGTTATTTATTGTCACTACCTCCCCGTGTCAGGATTGGGTAATTTGCGCGCCTGCTGCCTTCCTTGGATGTGGTAGCCGTTTCTCA	6 (0.000114%)	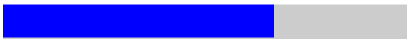
AAAGATTAAGCCATGCATGTGTAAGTATGAACGAATTCAGACTGTGAAACTGCGAATGGCTCATTAAATCAGTTATAGTTTGTTTGATGGTAACTACTAC	18 (0.000341%)	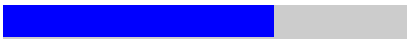
AAAGCAACGTCGCTATGAACGCTTGGCTGCCACAAGCCAGTTATCCCTGTGGTAACTTTTCTGACACCTCTAGCTTCAAATTCCGAAGGTCTAAAGGATC	269 (0.005089%)	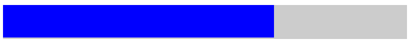
AAAGCCAAAGACTCATATGGACTTTGGCTACACCATGAAAGCTTTGAGAAGCAAGAAGAAGGTTGGTTAGTGTTTGGAGTCGAATATGACTTGATGTCA	314 (0.005940%)	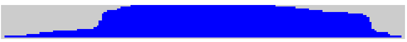
AAAGCCTAAGTAGTGTTTCCTTGTTAGAAGACACAAAGCCAAAGACTCATATGGACTTTGGCTACACCATGAAAGCTTTGAGAAGCAAGAAGAAGGTTGG	8 (0.000151%)	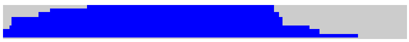

AAAGCTTTCATGGTGTAGCCAAAGTCCATATGAGTCTTTGGCTTTGTGTCTTCTAACAAGGAAACACTACTTAGGCTTATAAGATGCGGTTGCGGTTTAA	8 (0.000151%)
AAAGGCGTAAGAATTGTATCCTTGTTAAAAGACACAAAGCCAAAGACTCATATGGACTTTGGCTACACCATGAAAGCTTTGAGAAGCAAGAAGAAGGTTG	13 (0.000246%)
AAAGGCGTAAGAATTGTATCCTTGTTAGAAGACACAAAGCCAAAGACTCATATGGACTTTGGCTACACCATGAAAGCTTTGAGAAGCAAGAAGAAGGTTG	27 (0.000511%)
AAATCAGTTATAGTTTGTTTGATGGTAACTACTACTCGGATAACCGTAGTAATTCTAGAGCTAATACGTGCAACAAACCCCGACTTATGGAAGGGACGCA	3 (0.000057%)
AAATCCGCTAAGGAGTGTGTAACAACTCACCTGCCGAATCAACTAGCCCCGAAAATGGATGGCGCTTAAGCGCGCGACCTATACCCGGCCGTCGGGGCAA	9 (0.000170%)
AAATCCTATGATGTTATCCCATGCTAATGTATCCAGAGCGTAGGCTTGCTTTGAGCACTCTAATTTCTTCAAAGTAACAGCGCCGGAGGCACGACCCGGC	32 (0.000605%)
AAATCCTATTTTCTGGTAAATTTTCATAATTTTTTGACACCTCTAGCTAGGTCATTTGACCTGATACAACATCGGATTTTCATGGTCTAGTTGGGGCTCC	5 (0.000095%)
AAATTTTCATAATTTTTTGACACCTCTAGCTAGGTCATTTGACCTGATACAACATCGGATTTTCATGGTCTAGTTGGGGCTCCGTGGGCATATTTGATGC	8 (0.000151%)
AACAGGGACAGTCGGGGGCATTCGTATTTCATAGTCAGAGGTGAAATTCTTGGATTTATGAAAGACGAACAACTGCGAAAGCATTTGCCAAGGATGTTTT	19 (0.000359%)
AACAGGTCTGTGATGCCCTTAGATGTTCTGGGCCGCACGCGCGCTACACTGATGTATTCAACGAGTTCACACCTTGGCCGACAGGCCCGGGTAATCTTTG	9 (0.000170%)
AACCGCAACCGGATCTTAAAGGCGTAAGAATTGTATCCTTGTTAGAAGACACAAAGCCAAAGACTCATATGGACTTTGGCTACACCATGAAAGCTTTGAG	3 (0.000057%)
AACCTAGGCGAGACAAGGGTTCACATTTCGTTCATCACCCTTGGCCGGCTATCGAACAGCCGGACTCCCATCAAAAGATGGTTGCCAAGAACATCTTCGT	3 (0.000057%)
AACGAAGCACGCCCATCCAACCTAGGCGAGACAAGGGTTCACATTTCGTTCATCACCCTTGGCCGGCTATCGAACAGCCGGACTCCCATCAAAAGATGGT	19 (0.000359%)
AACGAAGCACGCCCATCCAACCTAGGCGAGACAAGGGTTCACATTTCGTTCATCACCCTTGGCCGGCTTTCGAACAGCCGGACTCCCATCAAAAGATGGT	21 (0.000397%)
AACGCCTCGAAGAACTAATGGCAGCCCACGCAAGGCAAGCCCATTCTCCTCGACGATTCAGCAGTTTTTGTCCGAGAACTGCTGAGAAAACTCGGAAAAA	11 (0.000208%)
AACGGCGTGCCTCGGCATCAGCGTGCTCCGGGCGTCGGCCTGTGGGCTCCCCATTCGACCCGTCTTGAAACACGGACCAAGGAGTCTGACATGTGTGCGA	3 (0.000057%)
AACGGGCAGAGCCCGCGTCGACCTTTTATCTAATAAATGCGTCCCTTCCATAAGTCGGGGTTTGTTGCACGTATTAGCTCTAGAATTACTACGGTTATCC	13 (0.000246%)
AAGAAGACCCTGTTGAGCTTGACTCTAGTCCGACTTTGTGAAATGACTTGAGAGGTGTAGGATAAGTGGGAGCTTCGGCGCAAGTGAAATACCACTACTT	7 (0.000132%)
AAGAATTGTATCCTTGTTAGAAGACACAAAGCCAAAGACTCATATGGACTTTGGCTACACCATGAAAGCTTTGAGAAGCAAGAAGAAGGTTGGTTAGTGT	17 (0.000322%)
AAGACACAAAGCCAAAGACTCATATGGACTTTGGCTACACCATGAAAGCTTTGAGAAGCAAGAAGAAGGTTGGTTAGTGTTTTGGAGTCGAATATGACTT	658 (0.012448%)
AAGACCCTGTTGAGCTTGACTCTAGTCCGACTTTGTGAAATGACTTGAGAGGTGTAGGATAAGTGGGAGCTTCGGCGCAAGTGAAATACCACTACTTTTA	3 (0.000057%)
AAGACTCATATGGACTTTGGCTACACCATGAAAGCTTTGAGAAGCAAGAAGAAGGTTGGTTAGTGTTTTGGAGTCGAATATGACTTGATGTCATGTGTAT	23 (0.000435%)
AAGAGCACACGTCTGAACTCCAGTCACGAGATTCCATCTC	8 (0.000061%)
AAGAGCCCGGTATTGTTATTTATTGTCACTACCTCCCCGTGTCAGGATTGGGTAATTTGCGCGCCTGCTGCCTTCCTTGGATGTGGTAGCCGTTTCTCAG	7 (0.000132%)
AAGATCGATC	202437 (0.382975%)
AAGCAACGTCGCTATGAACGCTTGGCTGCCACAAGCCAGTTATCCCTGTGGTAACTTTTCTGACACCTCTAGCTTCAAATTCCGAAGGTCTAAAGGATCG	138 (0.002611%)
AAGCACGCCCATCCAACCTAGGCGAGACAAGGGTTCACATTTCGTTCATCACCCTTGGCCGGCTTTCGAACAGCCGGACTCCCATCAAAAGATGGTTGCC	3 (0.000057%)
AAGCCAAAGACTCATATGGACTTTGGCTACACCATGAAAGCTTTGAGAAGCAAGAAGAAGGTTGGTTAGTGTTTTGGAGTCGAATATGACTTGATGTCAT	11 (0.000208%)
AAGCCATGCATGTGTAAGTATGAACGAATTCAGACTGTGAAACTGCGAATGGCTCATTAAATCAGTTATAGTTTGTTTGATGGTAACTACTACTCGGATA	3 (0.000057%)
AAGCCTAAGTAGTGTTTCCTTGTTAGAAGACACAAAGCCAAAGACTCATATGGACTTTGGCTACACCATGAAAGCTTTGAGAAGCAAGAAGAAGGTTGGT	1202 (0.022740%)
AAGCGCGCGACCTATACCCGGCCGTCGGGGCAAGAGCCAGGCCTCGATGAGTAGGAGGGCGCGGCGGTCGCTGCAAAACCTAGGGCGCGAGCCCGGGCGG	12 (0.000227%)
AAGCTTTCATGGTGTAGCCAAAGTCCATATGAGTCTTTGGCTTTGTGTCTTCTAACAAGGAAACACTACTTAGGCTTATAAGATGCGGTTGCGGTTTAAG	4 (0.000076%)
AAGGATACTAAATCCTATTTTCTGGTAAATTTTCATAATTTTTTGACACCTCTAGCTAGGTCATTTGACCTGATACAACATCGGATTTTCATGGTCTAGT	9 (0.000170%)
AAGGCGTAAGAATTGTATCCTTGTTAAAAGACACAAAGCCAAAGACTCATATGGACTTTGGCTACACCATGAAAGCTTTGAGAAGCAAGAAGAAGGTTGG	7 (0.000132%)
AAGGCGTAAGAATTGTATCCTTGTTAGAAGACACAAAGCCAAAGACTCATATGGACTTTGGCTACACCATGAAAGCTTTGAGAAGCAAGAAGAAGGTTGG	10 (0.000189%)
AAGGGATTTAGATTGTACTCATTCCAATTACCAGACTCGAAAGAGCCCGGTATTGTTATTTATTGTCACTACCTCCCCGTGTCAGGATTGGGTAATTTGC	57 (0.001078%)
AAGTAGTGTTTCCTTGTTAGAAGACACAAAGCCAAAGACTCATATGGACTTTGGCTACACCATGAAAGCTTTGAGAAGCAAGAAGAAGGTTGGTTAGTGT	18 (0.000341%)
AAGTCATATTCGACTCCAAAACACTAACCAACCTTCTTCTTGCTTCTCAAAGCTTTCATGGTGTAGCCAAAGTCCATATGAGTCTTTGGCTTTGTGTCTT	136 (0.002573%)
AAGTCGAAATCCGCTAAGGAGTGTGTAACAACTCACCTGCCGAATCAACTAGCCCCGAAAATGGATGGCGCTTAAGCGCGCGACCTATACCCGGCCGTCG	7 (0.000132%)
AAGTCTGGTGCCAGCAGCCGCGGTAATTCCAGCTCCAATAGCGTATATTTAAGTTGTTGCAGTTAAAAAGCTCGTAGTTGAACCTTGGGATGGGTCGGCC	3 (0.000057%)
AAGTTCACCACCGCATGTCGGTACGCTCCAGGCGTCCTTGGCTCGGATTTAGGCCAACCGCGTGCGGTAACACACGGGAGACCAGCTTCCGTCCCGCATC	23 (0.000435%)
AAGTTCTTATACTCAATCATACACATGACATCAAGTCATATTCGACTCCAAAACACTAACCAACCTTCTTCTTGCTTCTCAAAGCTTTCATGGTGTAGCC	35 (0.000662%)
AAGTTGTTATACTCAATCATACACATGACATCAAGTCATA	116 (0.000878%)

AATAACAGGTCTGTGATGCCCTTAGATGTTCTGGGCCGCACGCGCGCTACACTGATGTATTCAACGAGTTCACACCTTGGCCGACAGGCCCGGGTAATCT	20 (0.000378%)	
AATATGACTTGATGTCATGTGTATGATTGAGTATAAGAAC	1390 (0.010519%)	
AATCAAACGAGCTTTTACCCTTTTGTTCCACACGAGATTTCTGTTCTCGTTGAGCTCATCTTAGGACACCTGCGTTATCTTTTAACAGATGTGCCGCCCC	32 (0.000605%)	
AATCAGAATCAAACGAGCTTTTACCCTTTTGTTCCACACGAGATTTCTGTTCTCGTTGAGCTCATCTTAGGACACCTGCGTTATCTTTTAACAGATGTGC	4 (0.000076%)	
AATCAGTTATAGTTTGTTTGATGGTAACTACTACTCGGATAACCGTAGTAATTCTAGAGCTAATACGTGCAACAAACCCCGACTTATGGAAGGGACGCAT	8 (0.000151%)	
AATCATACACATGACAACAAGTCATATTCGACTCCAAAACACTAACCAACCTTCTTCTTGCTTCTCAAAGCTTTCATGGTGTAGCCAAAGTCCATATGAG	21 (0.000397%)	
AATCATACACATGACATCAAGTCATATTCGACTCCAAAACACTAACCAACCTTCTTCTTGCTTCTCAAAGCTTTCATGGTGTAGCCAAAGTCCATATGAG	63 (0.001192%)	
AATCATCAGAGCAACGGGCAGAGCCCGCGTCGACCTTTTATCTAATAAATGCGTCCCTTCCATAAGTCGGGGTTTGTTGCACGTATTAGCTCTAGAATTA	51 (0.000965%)	
AATCCGCTAAGGAGTGTGTAACAACTCACCTGCCGAATCAACTAGCCCCGAAAATGGATGGCGCTTAAGCGCGCGACCTATACCCGGCCGTCGGGGCAAG	14 (0.000265%)	
AATCCTATGATGTTATCCCATGCTAATGTATCCAGAGCGTAGGCTTGCTTTGAGCACTCTAATTTCTTCAAAGTAACAGCGCCGGAGGCACGACCCGGCC	42 (0.000795%)	
AATCCTTGTTAGTTTCTTTTCCTCCGCTTATTGATATGCTTAAACTCAGCGGGTAATCCCGCCTGACCTGGGGTCGCTATATGGACTTTGGGTCATCTAC	8 (0.000151%)	
AATCGGTAGGAGCGACGGGCGGTGTGTACAAAGGGCAGGGACGTAGTCAACGCGAGCTGATGACTCGCGCTTACTAGGAATTCCTCGTTGAAGACCAACA	71 (0.001343%)	
AATGATTAACAGGGACAGTCGGGGGCATTCGTATTTCATAGTCAGAGGTGAAATTCTTGGATTTATGAAAGACGAACAACTGCGAAAGCATTTGCCAAGG	93 (0.001759%)	
AATGGAAAGAGAGAAAGGACGAGGTCTTGACCGTCATCTTTTGCCCGAAGGACGGATGAGCTTTGGCGGGACTGAATCACTTCGAGTCACCGTCGACAAC	4 (0.000076%)	
AATGTATCCAGAGCGTAGGCTTGCTTTGAGCACTCTAATTTCTTCAAAGTAACAGCGCCGGAGGCACGACCCGGCCAATTAAGACCAGGAGCGTATCGCC	7 (0.000132%)	
AATTACCAGACTCGAAAGAGCCCGGTATTGTTATTTATTGTCACTACCTCCCCGTGTCAGGATTGGGTAATTTGCGCGCCTGCTGCCTTCCTTGGATGTG	23 (0.000435%)	
AATTAGGGTTCGATTCCGGAGAGGGAGCCTGAGAAACGGCTACCACATCCAAGGAAGGCAGCAGGCGCGCAAATTACCCAATCCTGACACGGGGAGGTAG	27 (0.000511%)	
AATTCCAGCTCCAATAGCGTATATTTAAGTTGTTGCAGTTAAAAAGCTCGTAGTTGAACCTTGGGATGGGTCGGCCGGTCCGCCTTTGGTGTGCATTGGT	12 (0.000227%)	
AATTCCCCGCCACATCCTCTCAAACGCAATGGAAAGAGAGAAAGGACGAGGTCTTGACCGTCATCTTTTGCCCGAAGGACGGATGAGCTTTGGCGGGACT	11 (0.000208%)	
AATTGTATCCTTGTTAGAAGACACAAAGCCAAAGACTCATATGGACTTTGGCTACACCATGAAAGCTTTGAGAAGCAAGAAGAAGGTTGGTTAGTGTTTT	4 (0.000076%)	
AATTGTTGGTCTTCAACGAGGAATTCCTAGTAAGCGCGAGTCATCAGCTCGCGTTGACTACGTCCCTGCCCTTTGTACACACCGCCCGTCGCTCCTACCG	1353 (0.025596%)	
AATTTTCATAATTTTTTGACACCTCTAGCTAGGTCATTTGACCTGATACAACATCGGATTTTCATGGTCTAGTTGGGGCTCCGTGGGCATATTTGATGCA	8 (0.000151%)	
ACAAAGCCAAAGACTCATATGGACTTTGGCTACACCATGAAAGCTTTGAGAAGCAAGAAGAAGGTTGGTTAGTGTTTTGGAGTCGAATATGACTTGATGT	167 (0.003159%)	
ACACAAAGCCAAAGACTCATATGGACTTTGGCTACACCATGAAAGCTTTGAGAAGCAAGAAGAAGGTTGGTTAGTGTTTTGGAGTCGAATATGACTTGAT	185 (0.003500%)	
ACACATGACATCAAGTCATATTCGACTCCAAAACACTAACCAACCTTCTTCTTGCTTCTCAAAGCTTTCATGGTGTAGCCAAAGTCCATATGAGTCTTTG	19 (0.000359%)	
ACACCATGAAAGCTTTGAGAAGCAAGAAGAAGGTTGGTTAGTGTTTTGGAGTCGAATATGACTTGATGTCATGTGTATGATTGAGTATAACAACTTAAAC	7 (0.000132%)	
ACACGTCTGAACTCCAGTCACGAGATTCCATCTCGTATGC	19 (0.000144%)	
ACATCAAGTCATATTCGACTCCAAAACACTAACCAACCTTCTTCTTGCTTCTCAAAGCTTTCATGGTGTAGCCAAAGTCCATATGAGTCTTTGGCTTTGT	58 (0.001097%)	
ACATCTGTTAAAAGATAACGCAGGTGTCCTAAGATGAGCTCAACGAGAACAGAAATCTCGTGTGGAACAAAAGGGTAAAAGCTCGTTTGATTCTGATTTT	4 (0.000076%)	
ACATGACATCAAGTCATATTCGACTCCAAAACACTAACCAACCTTCTTCTTGCTTCTCAAAGCTTTCATGGTGTAGCCAAAGTCCATATGAGTCTTTGGC	70 (0.001324%)	
ACATTGTCAGGTGGGGAGTTTGGCTGGGGCGGCACATCTGTTAAAAGATAACGCAGGTGTCCTAAGATGAGCTCAACGAGAACAGAAATCTCGTGTGGAA	8 (0.000151%)	
ACCACCGCATGTCGGTACGCTCCAGGCGTCCTTGGCTCGGATTTAGGCCAACCGCGTGCGGTAACACACGGGAGACCAGCTTCCGTCCCGCATCAGCAAA	12 (0.000227%)	
ACCAGACTCGAAAGAGCCCGGTATTGTTATTTATTGTCACTACCTCCCCGTGTCAGGATTGGGTAATTTGCGCGCCTGCTGCCTTCCTTGGATGTGGTAG	6 (0.000114%)	
ACCAGAGGCTGTTCACCTTGGAGACCTGATGCGGTTATGAGTACGACCGGGCGTGAGCGGCACTCGGTCCTCCGGATTTTCAAGGGCCGCCGGGGGCGCA	10 (0.000189%)	
ACCATCCTTTGCTGATGCGGGACGGAAGCTGGTCTCCCGTGTGTTACCGCACGCGGTTGGCCTAAATCCGAGCCAAGGACGCCTGGAGCGTACCGACATG	6 (0.000114%)	
ACCCGGCCGTCGGGGCAAGAGCCAGGCCTCGATGAGTAGGAGGGCGCGGCGGTCGCTGCAAAACCTAGGGCGCGAGCCCGGGCGGAGCGGCCGTCGGTGC	3 (0.000057%)	
ACCCGTTGACTCGCACACATGTCAGACTCCTTGGTCCGTGTTTCAAGACGGGTCGAATGGGGAGCCCACAGGCCGACGCCCGGAGCACGCTGATGCCGAG	4 (0.000076%)	
ACCCTGTTGAGCTTGACTCTAGTCCGACTTTGTGAAATGACTTGAGAGGTGTAGGATAAGTGGGAGCTTCGGCGCAAGTGAAATACCACTACTTTTAACG	5 (0.000095%)	
ACCGCATGTCGGTACGCTCCAGGCGTCCTTGGCTCGGATTTAGGCCAACCGCGTGCGGTAACACACGGGAGACCAGCTTCCGTCCCGCATCAGCAAAGGA	6 (0.000114%)	
ACCGGATTGCTCCGTTCCGCATCCGACCAGGACGCATCGCCGGCCCCCATCCGCTTCCCTCCCGACAATTTCAAGCACTCTTTGACTCTCTTTTCAAAGT	15 (0.000284%)	
ACCTAGGCGAGACAAGGGTTCACATTTCGTTCATCACCCTTGGCCGGCTATCGAACAGCCGGACTCCCATCAAAAGATGGTTGCCAAGAACATCTTCGTT	5 (0.000095%)	
ACCTAGGCGAGACAAGGGTTCACATTTCGTTCATCACCCTTGGCCGGCTTTCGAACAGCCGGACTCCCATCAAAAGATGGTTGCCAAGAACATCTTCGTT	3 (0.000057%)	
ACGAAGCACGCCCATCCAACCTAGGCGAGACAAGGGTTCACATTTCGTTCATCACCCTTGGCCGGCTATCGAACAGCCGGACTCCCATCAAAAGATGGTT	5 (0.000095%)	

ACGAAGCACGCCCATCCAACCTAGGCGAGACAAGGGTTCACATTTCGTTCATCACCCTTGGCCGGCTTTCGAACAGCCGGACTCCCATCAAAAGATGGTT	8 (0.000151%)
ACGAGGAATTCCTAGTAAGCGCGAGTCATCAGCTCGCGTTGACTACGTCCCTGCCCTTTGTACACACCGCCCGTCGCTCCTACCGATTGAATGATCGATC	4 (0.000076%)
ACGCGCCTAACGGCGTGCCTCGGCATCAGCGTGCTCCGGGCGTCGGCCTGTGGGCTCCCCATTCGACCCGTCTTGAAACACGGACCAAGGAGTCTGACAT	14 (0.000265%)
ACGCTTTCACGGTTCGTATTCGTACTGAAAATCAGAATCAAACGAGCTTTTACCCTTTTGTTCCACACGAGATTTCTGTTCTCGTTGAGCTCATCTTAGG	7 (0.000132%)
ACGGCAATTCCCCGCCACATCCTCTCAAACGCAATGGAAAGAGAGAAAGGACGAGGTCTTGACCGTCATCTTTTGCCCGAAGGACGGATGAGCTTTGGCG	7 (0.000132%)
ACGGCGTGCCTCGGCATCAGCGTGCTCCGGGCGTCGGCCTGTGGGCTCCCCATTCGACCCGTCTTGAAACACGGACCAAGGAGTCTGACATGTGTGCGAG	4 (0.000076%)
ACGGGCAGAGCCCGCGTCGACCTTTTATCTAATAAATGCGTCCCTTCCATAAGTCGGGGTTTGTTGCACGTATTAGCTCTAGAATTACTACGGTTATCCG	3 (0.000057%)
ACGGGCGGTGTGTACAAAGGGCAGGGACGTAGTCAACGCGAGCTGATGACTCGCGCTTACTAGGAATTCCTCGTTGAAGACCAACAATTGCAATGATCGA	3 (0.000057%)
ACGTCTGAACTCCAGTCACGAGATTCCATCTCGTATGCCG	18 (0.000136%)
ACTAAATCCTATTTTCTGGTAAATTTTCATAATTTTTTGACACCTCTAGCTAGGTCATTTGACCTGATACAACATCGGATTTTCATGGTCTAGTTGGGGC	24 (0.000454%)
ACTCAATCATACACATGACAACAAGTCATATTCGACTCCAAAACACTAACCAACCTTCTTCTTGCTTCTCAAAGCTTTCATGGTGTAGCCAAAGTCCATA	8 (0.000151%)
ACTCAATCATACACATGACATCAAGTCATATTCGACTCCAAAACACTAACCAACCTTCTTCTTGCTTCTCAAAGCTTTCATGGTGTAGCCAAAGTCCATA	26 (0.000492%)
ACTCATATGGACTTTGGCTACACCATGAAAGCTTTGAGAAGCAAGAAGAAGGTTGGTTAGTGTTTTGGAGTCGAATATGACTTGATGTCATGTGTATGAT	88 (0.001665%)
ACTCATTCCAATTACCAGACTCGAAAGAGCCCGGTATTGTTATTTATTGTCACTACCTCCCCGTGTCAGGATTGGGTAATTTGCGCGCCTGCTGCCTTCC	30 (0.000568%)
ACTCCAAAACACTAACCAACCTTCTTCTTGCTTCTCAAAGCTTTCATGGTGTAGCCAAAGTCCATATGAGTCTTTGGCTTTGTGTCTTCTAACAAGGAAA	203 (0.003840%)
ACTCGAAAGAGCCCGGTATTGTTATTTATTGTCACTACCTCCCCGTGTCAGGATTGGGTAATTTGCGCGCCTGCTGCCTTCCTTGGATGTGGTAGCCGTT	4 (0.000076%)
ACTGAAAATCAGAATCAAACGAGCTTTTACCCTTTTGTTCCACACGAGATTTCTGTTCTCGTTGAGCTCATCTTAGGACACCTGCGTTATCTTTTAACAG	5 (0.000095%)
ACTTCCCTTGCCTACATTGTTCCATCGACCAGAGGCTGTTCACCTTGGAGACCTGATGCGGTTATGAGTACGACCGGGCGTGAGCGGCACTCGGTCCTCC	13 (0.000246%)
ACTTGATGTCATGTGTATGATTGAGTATAAGAACTTAAAC	358 (0.002709%)
ACTTTGTGAAATGACTTGAGAGGTGTAGGATAAGTGGGAGCTTCGGCGCAAGTGAAATACCACTACTTTTAACGTTATTTTACTTACTCCGTGAATCGGA	5 (0.000095%)
AGAAGACACAAAGCCAAAGACTCATATGGACTTTGGCTACACCATGAAAGCTTTGAGAAGCAAGAAGAAGGTTGGTTAGTGTTTTGGAGTCGAATATGAC	81 (0.001532%)
AGAAGACCCTGTTGAGCTTGACTCTAGTCCGACTTTGTGAAATGACTTGAGAGGTGTAGGATAAGTGGGAGCTTCGGCGCAAGTGAAATACCACTACTTT	51 (0.000965%)
AGAATTGTATCCTTGTTAGAAGACACAAAGCCAAAGACTCATATGGACTTTGGCTACACCATGAAAGCTTTGAGAAGCAAGAAGAAGGTTGGTTAGTGTT	6 (0.000114%)
AGACAAGGGTTCACATTTCGTTCATCACCCTTGGCCGGCTATCGAACAGCCGGACTCCCATCAAAAGATGGTTGCCAAGAACATCTTCGTTACGGTTTGC	3 (0.000057%)
AGACACAAAGCCAAAGACTCATATGGACTTTGGCTACACCATGAAAGCTTTGAGAAGCAAGAAGAAGGTTGGTTAGTGTTTTGGAGTCGAATATGACTTG	39 (0.000738%)
AGACCCTGTTGAGCTTGACTCTAGTCCGACTTTGTGAAATGACTTGAGAGGTGTAGGATAAGTGGGAGCTTCGGCGCAAGTGAAATACCACTACTTTTAA	6 (0.000114%)
AGACTCATATGGACTTTGGCTACACCATGAAAGCTTTGAGAAGCAAGAAGAAGGTTGGTTAGTGTTTTGGAGTCGAATATGACTTGATGTCATGTGTATG	15 (0.000284%)
AGACTCGAAAGAGCCCGGTATTGTTATTTATTGTCACTACCTCCCCGTGTCAGGATTGGGTAATTTGCGCGCCTGCTGCCTTCCTTGGATGTGGTAGCCG	5 (0.000095%)
AGAGCAACGGGCAGAGCCCGCGTCGACCTTTTATCTAATAAATGCGTCCCTTCCATAAGTCGGGGTTTGTTGCACGTATTAGCTCTAGAATTACTACGGT	33 (0.000624%)
AGAGCACACGTCTGAACTCCAGTCACGAGATTCCATCTCG	15 (0.000114%)
AGAGCCCGCGTCGACCTTTTATCTAATAAATGCGTCCCTTCCATAAGTCGGGGTTTGTTGCACGTATTAGCTCTAGAATTACTACGGTTATCCGAGTAGT	7 (0.000132%)
AGAGCCCGGTATTGTTATTTATTGTCACTACCTCCCCGTGTCAGGATTGGGTAATTTGCGCGCCTGCTGCCTTCCTTGGATGTGGTAGCCGTTTCTCAGG	3 (0.000057%)
AGAGCGTAGGCTTGCTTTGAGCACTCTAATTTCTTCAAAGTAACAGCGCCGGAGGCACGACCCGGCCAATTAAGACCAGGAGCGTATCGCCGACCGAAGG	14 (0.000265%)
AGATCGGAAGAGCACACGTCTGAACTCCAGTCACGAGATT	20073 (0.151898%)
AGATGTTCTGGGCCGCACGCGCGCTACACTGATGTATTCAACGAGTTCACACCTTGGCCGACAGGCCCGGGTAATCTTTGAAATTTCATCGTGATGGGGA	8 (0.000151%)
AGATTAAGCCATGCATGTGTAAGTATGAACGAATTCAGACTGTGAAACTGCGAATGGCTCATTAAATCAGTTATAGTTTGTTTGATGGTAACTACTACTC	4 (0.000076%)
AGATTGTACTCATTCCAATTACCAGACTCGAAAGAGCCCGGTATTGTTATTTATTGTCACTACCTCCCCGTGTCAGGATTGGGTAATTTGCGCGCCTGCT	12 (0.000227%)
AGCAACGGGCAGAGCCCGCGTCGACCTTTTATCTAATAAATGCGTCCCTTCCATAAGTCGGGGTTTGTTGCACGTATTAGCTCTAGAATTACTACGGTTA	4 (0.000076%)
AGCAACGTCGCTATGAACGCTTGGCTGCCACAAGCCAGTTATCCCTGTGGTAACTTTTCTGACACCTCTAGCTTCAAATTCCGAAGGTCTAAAGGATCGA	32 (0.000605%)
AGCACACGTCTGAACTCCAGTCACGAGATTCCATCTCGTA	8 (0.000061%)
AGCACGCCCATCCAACCTAGGCGAGACAAGGGTTCACATTTCGTTCATCACCCTTGGCCGGCTATCGAACAGCCGGACTCCCATCAAAAGATGGTTGCCA	12 (0.000227%)
AGCACGCCCATCCAACCTAGGCGAGACAAGGGTTCACATTTCGTTCATCACCCTTGGCCGGCTTTCGAACAGCCGGACTCCCATCAAAAGATGGTTGCCA	16 (0.000303%)
AGCACGCGCCTAACGGCGTGCCTCGGCATCAGCGTGCTCCGGGCGTCGGCCTGTGGGCTCCCCATTCGACCCGTCTTGAAACACGGACCAAGGAGTCTGA	31 (0.000586%)

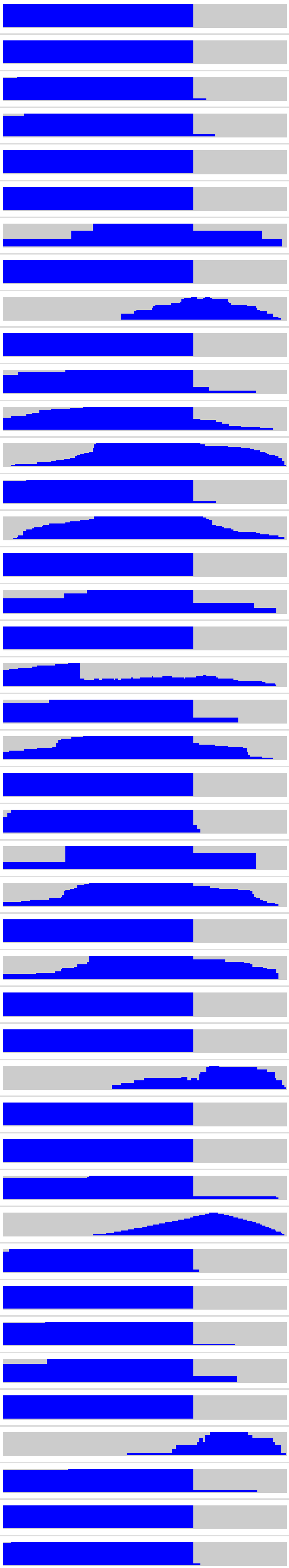

AGCAGCCGCGGTAATTCCAGCTCCAATAGCGTATATTTAAGTTGTTGCAGTTAAAAAGCTCGTAGTTGAACCTTGGGATGGGTCGGCCGGTCCGCCTTTG	7 (0.000132%)	
AGCCAAAGACTCATATGGACTTTGGCTACACCATGAAAGCTTTGAGAAGCAAGAAGAAGGTTGGTTAGTGTTTTGGAGTCGAATATGACTTGATGTCATG	10 (0.000189%)	
AGCCCGCGTCGACCTTTTATCTAATAAATGCGTCCCTTCCATAAGTCGGGGTTTGTTGCACGTATTAGCTCTAGAATTACTACGGTTATCCGAGTAGTAG	3 (0.000057%)	
AGCCGCGGTAATTCCAGCTCCAATAGCGTATATTTAAGTTGTTGCAGTTAAAAAGCTCGTAGTTGAACCTTGGGATGGGTCGGCCGGTCCGCCTTTGGTG	9 (0.000170%)	
AGCCTAAGTAGTGTTTCCTTGTTAGAAGACACAAAGCCAAAGACTCATATGGACTTTGGCTACACCATGAAAGCTTTGAGAAGCAAGAAGAAGGTTGGTT	34 (0.000643%)	
AGCGACGGGCGGTGTGTACAAAGGGCAGGGACGTAGTCAACGCGAGCTGATGACTCGCGCTTACTAGGAATTCCTCGTTGAAGACCAACAATTGCAATGA	5 (0.000095%)	
AGCGAGGTGTGAGTGTCGCCCATGGGCATCGACACCTTGCGGCTAGGAACTGGAACGAGACGGGTGGCAAAGATTTCGAGTAGCACTTCATACTACCGTG	6 (0.000114%)	
AGCGTAGGCTTGCTTTGAGCACTCTAATTTCTTCAAAGTAACAGCGCCGGAGGCACGACCCGGCCAATTAAGACCAGGAGCGTATCGCCGACCGAAGGGA	6 (0.000114%)	
AGGACATTGTCAGGTGGGGAGTTTGGCTGGGGCGGCACATCTGTTAAAAGATAACGCAGGTGTCCTAAGATGAGCTCAACGAGAACAGAAATCTCGTGTG	188 (0.003557%)	
AGGAGCGACGGGCGGTGTGTACAAAGGGCAGGGACGTAGTCAACGCGAGCTGATGACTCGCGCTTACTAGGAATTCCTCGTTGAAGACCAACAATTGCAA	17 (0.000322%)	
AGGATACTAAATCCTATTTTCTGGTAAATTTTCATAATTTTTTGACACCTCTAGCTAGGTCATTTGACCTGATACAACATCGGATTTTCATGGTCTAGTT	4 (0.000076%)	
AGGCCACGCTTTCACGGTTCGTATTCGTACTGAAAATCAGAATCAAACGAGCTTTTACCCTTTTGTTCCACACGAGATTTCTGTTCTCGTTGAGCTCATC	15 (0.000284%)	
AGGCGAGACAAGGGTTCACATTTCGTTCATCACCCTTGGCCGGCTTTCGAACAGCCGGACTCCCATCAAAAGATGGTTGCCAAGAACATCTTCGTTACGG	7 (0.000132%)	
AGGCGTAAGAATTGTATCCTTGTTAAAAGACACAAAGCCAAAGACTCATATGGACTTTGGCTACACCATGAAAGCTTTGAGAAGCAAGAAGAAGGTTGGT	6 (0.000114%)	
AGGCGTAAGAATTGTATCCTTGTTAGAAGACACAAAGCCAAAGACTCATATGGACTTTGGCTACACCATGAAAGCTTTGAGAAGCAAGAAGAAGGTTGGT	3 (0.000057%)	
AGGCTGTCCCGAGTGTGAGCGAGGTGTGAGTGTCGCCCATGGGCATCGACACCTTGCGGCTAGGAACTGGAACGAGACGGGTAGCAAAGATTTCGAGTAG	16 (0.000303%)	
AGGCTGTCCCGAGTGTGAGCGAGGTGTGAGTGTCGCCCATGGGCATCGACACCTTGCGGCTAGGAACTGGAACGAGACGGGTGGCAAAGATTTCGAGTAG	11 (0.000208%)	
AGGGAATCCTTGTTAGTTTCTTTTCCTCCGCTTATTGATATGCTTAAACTCAGCGGGTAATCCCGCCTGACCTGGGGTCGCTATATGGACTTTGGGTCAT	227 (0.004294%)	
AGGGACAGTCGGGGGCATTCGTATTTCATAGTCAGAGGTGAAATTCTTGGATTTATGAAAGACGAACAACTGCGAAAGCATTTGCCAAGGATGTTTTCAT	5 (0.000095%)	
AGGGATTTAGATTGTACTCATTCCAATTACCAGACTCGAAAGAGCCCGGTATTGTTATTTATTGTCACTACCTCCCCGTGTCAGGATTGGGTAATTTGCG	10 (0.000189%)	
AGGGCAAGTCTGGTGCCAGCAGCCGCGGTAATTCCAGCTCCAATAGCGTATATTTAAGTTGTTGCAGTTAAAAAGCTCGTAGTTGAACCTTGGGATGGGT	25 (0.000473%)	
AGGTCTGTGATGCCCTTAGATGTTCTGGGCCGCACGCGCGCTACACTGATGTATTCAACGAGTTCACACCTTGGCCGACAGGCCCGGGTAATCTTTGAAA	6 (0.000114%)	
AGTAGTCATATGCTTGTCTCAAAGATTAAGCCATGCATGTGTAAGTATGAACGAATTCAGACTGTGAAACTGCGAATGGCTCATTAAATCAGTTATAGTT	73 (0.001381%)	
AGTAGTGTTTCCTTGTTAGAAGACACAAAGCCAAAGACTCATATGGACTTTGGCTACACCATGAAAGCTTTGAGAAGCAAGAAGAAGGTTGGTTAGTGTT	27 (0.000511%)	
AGTCAGAGGTGAAATTCTTGGATTTATGAAAGACGAACAACTGCGAAAGCATTTGCCAAGGATGTTTTCATTAATCAAGAACGAAAGTTGGGGGCTCGAA	9 (0.000170%)	
AGTCATATGCTTGTCTCAAAGATTAAGCCATGCATGTGTAAGTATGAACGAATTCAGACTGTGAAACTGCGAATGGCTCATTAAATCAGTTATAGTTTGT	11 (0.000208%)	
AGTCATATTCGACTCCAAAACACTAACCAACCTTCTTCTTGCTTCTCAAAGCTTTCATGGTGTAGCCAAAGTCCATATGAGTCTTTGGCTTTGTGTCTTC	6 (0.000114%)	
AGTCGAAATCCGCTAAGGAGTGTGTAACAACTCACCTGCCGAATCAACTAGCCCCGAAAATGGATGGCGCTTAAGCGCGCGACCTATACCCGGCCGTCGG	9 (0.000170%)	
AGTCGGGGGCATTCGTATTTCATAGTCAGAGGTGAAATTCTTGGATTTATGAAAGACGAACAACTGCGAAAGCATTTGCCAAGGATGTTTTCATTAATCA	8 (0.000151%)	
AGTGTATCCTTGTTAGAAGACACAAAGCCAAAGACTCATATGGACTTTGGCTACACCATGAAAGCTTTGAGAAGCAAGAAGAAGGTTGGTTAGTGTTTTG	768 (0.014529%)	
AGTGTGAGCGAGGTGTGAGTGTCGCCCATGGGCATCGACACCTTGCGGCTAGGAACTGGAACGAGACGGGTGGCAAAGATTTCGAGTAGCACTTCATACT	4 (0.000076%)	
AGTGTTTCCTTGTTAGAAGACACAAAGCCAAAGACTCATATGGACTTTGGCTACACCATGAAAGCTTTGAGAAGCAAGAAGAAGGTTGGTTAGTGTTTTG	12 (0.000227%)	
AGTTATCATGAATCATCAGAGCAACGGGCAGAGCCCGCGTCGACCTTTTATCTAATAAATGCGTCCCTTCCATAAGTCGGGGTTTGTTGCACGTATTAGC	12 (0.000227%)	
AGTTCACCACCGCATGTCGGTACGCTCCAGGCGTCCTTGGCTCGGATTTAGGCCAACCGCGTGCGGTAACACACGGGAGACCAGCTTCCGTCCCGCATCA	6 (0.000114%)	
AGTTCTTATACTCAATCATACACATGACATCAAGTCATATTCGACTCCAAAACACTAACCAACCTTCTTCTTGCTTCTCAAAGCTTTCATGGTGTAGCCA	30 (0.000568%)	
AGTTGTTATACTCAATCATACACATGACATCAAGTCATAT	98 (0.000742%)	
AGTTTCTTTTCCTCCGCTTATTGATATGCTTAAACTCAGCGGGTAATCCCGCCTGACCTGGGGTCGCTATATGGACTTTGGGTCATCTACAGCTTCCGGA	4 (0.000076%)	
ATAACAGGTCTGTGATGCCCTTAGATGTTCTGGGCCGCACGCGCGCTACACTGATGTATTCAACGAGTTCACACCTTGGCCGACAGGCCCGGGTAATCTT	27 (0.000511%)	
ATAAGGATACTAAATCCTATTTTCTGGTAAATTTTCATAATTTTTTGACACCTCTAGCTAGGTCATTTGACCTGATACAACATCGGATTTTCATGGTCTA	13 (0.000246%)	
ATAATTTTTTGACACCTCTAGCTAGGTCATTTGACCTGATACAACATCGGATTTTCATGGTCTAGTTGGGGCTCCGTGGGCATATTTGATGCAAACTTGA	10 (0.000189%)	
ATACACATGACATCAAGTCATATTCGACTCCAAAACACTAACCAACCTTCTTCTTGCTTCTCAAAGCTTTCATGGTGTAGCCAAAGTCCATATGAGTCTT	291 (0.005505%)	
ATACCCGGCCGTCGGGGCAAGAGCCAGGCCTCGATGAGTAGGAGGGCGCGGCGGTCGCTGCAAAACCTAGGGCGCGAGCCCGGGCGGAGCGGCCGTCGGT	11 (0.000208%)	
ATACTCAATCATACACATGACAACAAGTCATATTCGACTCCAAAACACTAACCAACCTTCTTCTTGCTTCTCAAAGCTTTCATGGTGTAGCCAAAGTCCA	14 (0.000265%)	

ATACTCAATCATACACATGACATCAAGTCATATTCGACTCCAAAACACTAACCAACCTTCTTCTTGCTTCTCAAAGCTTTCATGGTGTAGCCAAAGTCCA	30 (0.000568%)
ATAGGCCACGCTTTCACGGTTCGTATTCGTACTGAAAATCAGAATCAAACGAGCTTTTACCCTTTTGTTCCACACGAGATTTCTGTTCTCGTTGAGCTCA	88 (0.001665%)
ATAGTCAGAGGTGAAATTCTTGGATTTATGAAAGACGAACAACTGCGAAAGCATTTGCCAAGGATGTTTTCATTAATCAAGAACGAAAGTTGGGGGCTCG	7 (0.000132%)
ATAGTGGCCTACCATGGTGGTAACGGGTGACGGAGAATTAGGGTTCGATTCCGGAGAGGGAGCCTGAGAAACGGCTACCACATCCAAGGAAGGCAGCAGG	18 (0.000341%)
ATAGTTTGTTTGATGGTAACTACTACTCGGATAACCGTAGTAATTCTAGAGCTAATACGTGCAACAAACCCCGACTTATGGAAGGGACGCATTTATTAGA	6 (0.000114%)
ATATGACTTGATGTCATGTGTATGATTGAGTATAAGAACT	154 (0.001165%)
ATATGCTTGTCTCAAAGATTAAGCCATGCATGTGTAAGTATGAACGAATTCAGACTGTGAAACTGCGAATGGCTCATTAAATCAGTTATAGTTTGTTTGA	57 (0.001078%)
ATATGGACTTTGGCTACACCATGAAAGCTTTGAGAAGCAAGAAGAAGGTTGGTTAGTGTTTTGGAGTCGAATATGACTTGATGTCATGTGTATGATTGAG	26 (0.000492%)
ATATTCGACTCCAAAACACTAACCAACCTTCTTCTTGCTTCTCAAAGCTTTCATGGTGTAGCCAAAGTCCATATGAGTCTTTGGCTTTGTGTCTTCTAAC	164 (0.003103%)
ATCAAACGAGCTTTTACCCTTTTGTTCCACACGAGATTTCTGTTCTCGTTGAGCTCATCTTAGGACACCTGCGTTATCTTTTAACAGATGTGCCGCCCCA	7 (0.000132%)
ATCAAGTCATATTCGACTCCAAAACACTAACCAACCTTCTTCTTGCTTCTCAAAGCTTTCATGGTGTAGCCAAAGTCCATATGAGTCTTTGGCTTTGTGT	1462 (0.027658%)
ATCAAGTTCACCACCGCATGTCGGTACGCTCCAGGCGTCCTTGGCTCGGATTTAGGCCAACCGCGTGCGGTAACACACGGGAGACCAGCTTCCGTCCCGC	106 (0.002005%)
ATCACGGCAATTCCCCGCCACATCCTCTCAAACGCAATGGAAAGAGAGAAAGGACGAGGTCTTGACCGTCATCTTTTGCCCGAAGGACGGATGAGCTTTG	8 (0.000151%)
ATCAGAATCAAACGAGCTTTTACCCTTTTGTTCCACACGAGATTTCTGTTCTCGTTGAGCTCATCTTAGGACACCTGCGTTATCTTTTAACAGATGTGCC	12 (0.000227%)
ATCAGAGCAACGGGCAGAGCCCGCGTCGACCTTTTATCTAATAAATGCGTCCCTTCCATAAGTCGGGGTTTGTTGCACGTATTAGCTCTAGAATTACTAC	51 (0.000965%)
ATCAGCGTGCTCCGGGCGTCGGCCTGTGGGCTCCCCATTCGACCCGTCTTGAAACACGGACCAAGGAGTCTGACATGTGTGCGAGTCAACGGGTGAGTAA	7 (0.000132%)
ATCAGTTATAGTTTGTTTGATGGTAACTACTACTCGGATAACCGTAGTAATTCTAGAGCTAATACGTGCAACAAACCCCGACTTATGGAAGGGACGCATT	6 (0.000114%)
ATCATAAGGATACTAAATCCTATTTTCTGGTAAATTTTCATAATTTTTTGACACCTCTAGCTAGGTCATTTGACCTGATACAACATCGGATTTTCATGGT	3 (0.000057%)
ATCATACACATGACAACAAGTCATATTCGACTCCAAAACACTAACCAACCTTCTTCTTGCTTCTCAAAGCTTTCATGGTGTAGCCAAAGTCCATATGAGT	4 (0.000076%)
ATCATACACATGACATCAAGTCATATTCGACTCCAAAACACTAACCAACCTTCTTCTTGCTTCTCAAAGCTTTCATGGTGTAGCCAAAGTCCATATGAGT	30 (0.000568%)
ATCATCAGAGCAACGGGCAGAGCCCGCGTCGACCTTTTATCTAATAAATGCGTCCCTTCCATAAGTCGGGGTTTGTTGCACGTATTAGCTCTAGAATTAC	8 (0.000151%)
ATCATGAATCATCAGAGCAACGGGCAGAGCCCGCGTCGACCTTTTATCTAATAAATGCGTCCCTTCCATAAGTCGGGGTTTGTTGCACGTATTAGCTCTA	75 (0.001419%)
ATCATTCAATCGGTAGGAGCGACGGGCGGTGTGTACAAAGGGCAGGGACGTAGTCAACGCGAGCTGATGACTCGCGCTTACTAGGAATTCCTCGTTGAAG	20 (0.000378%)
ATCCAACCTAGGCGAGACAAGGGTTCACATTTCGTTCATCACCCTTGGCCGGCTATCGAACAGCCGGACTCCCATCAAAAGATGGTTGCCAAGAACATCT	23 (0.000435%)
ATCCAACCTAGGCGAGACAAGGGTTCACATTTCGTTCATCACCCTTGGCCGGCTTTCGAACAGCCGGACTCCCATCAAAAGATGGTTGCCAAGAACATCT	24 (0.000454%)
ATCCAGAGCGTAGGCTTGCTTTGAGCACTCTAATTTCTTCAAAGTAACAGCGCCGGAGGCACGACCCGGCCAATTAAGACCAGGAGCGTATCGCCGACCG	13 (0.000246%)
ATCCATGCTTTCCAACGAAGCACGCCCATCCAACCTAGGCGAGACAAGGGTTCACATTTCGTTCATCACCCTTGGCCGGCTATCGAACAGCCGGACTCCC	7 (0.000132%)
ATCCATTGGAGGGCAAGTCTGGTGCCAGCAGCCGCGGTAATTCCAGCTCCAATAGCGTATATTTAAGTTGTTGCAGTTAAAAAGCTCGTAGTTGAACCTT	29 (0.000549%)
ATCCCATGCTAATGTATCCAGAGCGTAGGCTTGCTTTGAGCACTCTAATTTCTTCAAAGTAACAGCGCCGGAGGCACGACCCGGCCAATTAAGACCAGGA	13 (0.000246%)
ATCCGCCTAGGCTGTCCCGAGTGTGAGCGAGGTGTGAGTGTCGCCCATGGGCATCGACACCTTGCGGCTAGGAACTGGAACGAGACGGGTAGCAAAGATT	60 (0.001135%)
ATCCGCCTAGGCTGTCCCGAGTGTGAGCGAGGTGTGAGTGTCGCCCATGGGCATCGACACCTTGCGGCTAGGAACTGGAACGAGACGGGTGGCAAAGATT	114 (0.002157%)
ATCCGCTAAGGAGTGTGTAACAACTCACCTGCCGAATCAACTAGCCCCGAAAATGGATGGCGCTTAAGCGCGCGACCTATACCCGGCCGTCGGGGCAAGA	8 (0.000151%)
ATCCGGTTAAAATTCCGGAACCGGGACGTGGCGGTTGACGGCAACGTTAGGGAGTCCGGAGACGTCGGCGGGGGCCTCGGGAAGAGTTATCTTTTCTGTT	19 (0.000359%)
ATCCGTAACTTCGGGAAAAGGATTGGCTCTGAGGGCTGGGCTCGGGGGTCCCAGTTCCGAACCCGTCGGCTGTCAGCGGACTGCTCGAGCTGCTTCCGCG	4 (0.000076%)
ATCCGTCGAGTTATCATGAATCATCAGAGCAACGGGCAGAGCCCGCGTCGACCTTTTATCTAATAAATGCGTCCCTTCCATAAGTCGGGGTTTGTTGCAC	7 (0.000132%)
ATCCTATGATGTTATCCCATGCTAATGTATCCAGAGCGTAGGCTTGCTTTGAGCACTCTAATTTCTTCAAAGTAACAGCGCCGGAGGCACGACCCGGCCA	62 (0.001173%)
ATCCTCGTTAAGGGATTTAGATTGTACTCATTCCAATTACCAGACTCGAAAGAGCCCGGTATTGTTATTTATTGTCACTACCTCCCCGTGTCAGGATTGG	12 (0.000227%)
ATCCTCTCAAACGCAATGGAAAGAGAGAAAGGACGAGGTCTTGACCGTCATCTTTTGCCCGAAGGACGGATGAGCTTTGGCGGGACTGAATCACTTCGAG	3 (0.000057%)
ATCCTTGTTAGAAGACACAAAGCCAAAGACTCATATGGACTTTGGCTACACCATGAAAGCTTTGAGAAGCAAGAAGAAGGTTGGTTAGTGTTTTGGAGTC	6 (0.000114%)
ATCCTTGTTAGTTTCTTTTCCTCCGCTTATTGATATGCTTAAACTCAGCGGGTAATCCCGCCTGACCTGGGGTCGCTATATGGACTTTGGGTCATCTACA	7 (0.000132%)
ATCCTTTGCTGATGCGGGACGGAAGCTGGTCTCCCGTGTGTTACCGCACGCGGTTGGCCTAAATCCGAGCCAAGGACGCCTGGAGCGTACCGACATGCGG	6 (0.000114%)
ATCGAAATCCTATGATGTTATCCCATGCTAATGTATCCAGAGCGTAGGCTTGCTTTGAGCACTCTAATTTCTTCAAAGTAACAGCGCCGGAGGCACGACC	8 (0.000151%)
ATCGACACCTTGCGGCTAGGAACTGGAACGAGACGGGTGGCAAAGATTTCGAGTAGCACTTCATACTACCGTGGGTTTTTTAAACCTTCCGAGTTTTGTT	13 (0.000246%)

ATCGACCAGAGGCTGTTCACCTTGGAGACCTGATGCGGTTATGAGTACGACCGGGCGTGAGCGGCACTCGG<br>TCCTCCGGATTTTCAAGGGCCGCCGGGGG	34 (0.000643%)	
ATCGATCAAGTTCACCACCGCATGTCGGTACGCTCCAGGCGTCCTTGGCTCGGATTTAGGCCAACCGCGTG<br>CGGTAACACACGGGAGACCAGCTTCCGTC	5 (0.000095%)	
ATCGATCACGGCAATTCCCCGCCACATCCTCTCAAACGCAATGGAAAGAGAGAAAGGACGAGGTCTTGACC<br>GTCATCTTTTGCCCGAAGGACGGATGAGC	131 (0.002478%)	
ATCGATCCATGCTTTCCAACGAAGCACGCCCATCCAACCTAGGCGAGACAAGGGTTCACATTTCGTTCATC<br>ACCCTTGGCCGGCTATCGAACAGCCGGAC	38 (0.000719%)	
ATCGATCCGCCTAGGCTGTCCCGAGTGTGAGCGAGGTGTGAGTGTCGCCCATGGGCATCGACACCTTGCGG<br>CTAGGAACTGGAACGAGACGGGTAGCAAA	12 (0.000227%)	
ATCGATCCGCCTAGGCTGTCCCGAGTGTGAGCGAGGTGTGAGTGTCGCCCATGGGCATCGACACCTTGCGG<br>CTAGGAACTGGAACGAGACGGGTGGCAAA	24 (0.000454%)	
ATCGATCCGGTTAAAATTCCGGAACCGGGACGTGGCGGTTGACGGCAACGTTAGGGAGTCCGGAGACGTCG<br>GCGGGGGCCTCGGGAAGAGTTATCTTTTC	17 (0.000322%)	
ATCGATCCGTAACTTCGGGAAAAGGATTGGCTCTGAGGGCTGGGCTCGGGGGTCCCAGTTCCGAACCCGTC<br>GGCTGTCAGCGGACTGCTCGAGCTGCTTC	36 (0.000681%)	
ATCGATCCGTCGAGTTATCATGAATCATCAGAGCAACGGGCAGAGCCCGCGTCGACCTTTTATCTAATAAA<br>TGCGTCCCTTCCATAAGTCGGGGTTTGTT	17 (0.000322%)	
ATCGATCCTCGTTAAGGGATTTAGATTGTACTCATTCCAATTACCAGACTCGAAAGAGCCCGGTATTGTTA<br>TTTATTGTCACTACCTCCCCGTGTCAGGA	45 (0.000851%)	
ATCGATCGAAATCCTATGATGTTATCCCATGCTAATGTATCCAGAGCGTAGGCTTGCTTTGAGCACTCTAA<br>TTTCTTCAAAGTAACAGCGCCGGAGGCAC	11 (0.000208%)	
ATCGATCGGCGGACCGGATTGCTCCGTTCCGCATCCGACCAGGACGCATCGCCGGCCCCCATCCGCTTCCC<br>TCCCGACAATTTCAAGCACTCTTTGACTC	18 (0.000341%)	
ATCGATCGGGTTGCGGTTTAAGTTGTTATACTCAATCATA	23 (0.000174%)	
ATCGGAAGAGCACACGTCTGAACTCCAGTCACGAGATTCC	43 (0.000325%)	
ATCGGCGGACCGGATTGCTCCGTTCCGCATCCGACCAGGACGCATCGCCGGCCCCCATCCGCTTCCCTCCC<br>GACAATTTCAAGCACTCTTTGACTCTCTT	5 (0.000095%)	
ATCGGGTTGCGGTTTAAGTTGTTATACTCAATCATACACA	132 (0.000999%)	
ATCGGTAGGAGCGACGGGCGGTGTGTACAAAGGGCAGGGACGTAGTCAACGCGAGCTGATGACTCGCGCTT<br>ACTAGGAATTCCTCGTTGAAGACCAACAA	74 (0.001400%)	
ATCTAATAAATGCGTCCCTTCCATAAGTCGGGGTTTGTTGCACGTATTAGCTCTAGAATTACTACGGTTAT<br>CCGAGTAGTAGTTACCATCAAACAAACTA	5 (0.000095%)	
ATCTGTTAAAAGATAACGCAGGTGTCCTAAGATGAGCTCAACGAGAACAGAAATCTCGTGTGGAACAAAAG<br>GGTAAAAGCTCGTTTGATTCTGATTTTCA	6 (0.000114%)	
ATCTTAAAAGCCTAAGTAGTGTTTCCTTGTTAGAAGACACAAAGCCAAAGACTCATATGGACTTTGGCTAC<br>ACCATGAAAGCTTTGAGAAGCAAGAAGAA	593 (0.011219%)	
ATCTTAAAGGCGTAAGAATTGTATCCTTGTTAAAAGACACAAAGCCAAAGACTCATATGGACTTTGGCTAC<br>ACCATGAAAGCTTTGAGAAGCAAGAAGAA	43 (0.000813%)	
ATCTTAAAGGCGTAAGAATTGTATCCTTGTTAGAAGACACAAAGCCAAAGACTCATATGGACTTTGGCTAC<br>ACCATGAAAGCTTTGAGAAGCAAGAAGAA	28 (0.000530%)	
ATGAATCATCAGAGCAACGGGCAGAGCCCGCGTCGACCTTTTATCTAATAAATGCGTCCCTTCCATAAGTC<br>GGGGTTTGTTGCACGTATTAGCTCTAGAA	82 (0.001551%)	
ATGACATCAAGTCATATTCGACTCCAAAACACTAACCAACCTTCTTCTTGCTTCTCAAAGCTTTCATGGTG<br>TAGCCAAAGTCCATATGAGTCTTTGGCTT	117 (0.002213%)	
ATGACTTGATGTCATGTGTATGATTGAGTATAAGAACTTA	285 (0.002157%)	
ATGATGTTATCCCATGCTAATGTATCCAGAGCGTAGGCTTGCTTTGAGCACTCTAATTTCTTCAAAGTAAC<br>AGCGCCGGAGGCACGACCCGGCCAATTAA	66 (0.001249%)	
ATGATTAACAGGGACAGTCGGGGGCATTCGTATTTCATAGTCAGAGGTGAAATTCTTGGATTTATGAAAGA<br>CGAACAACTGCGAAAGCATTTGCCAAGGA	100 (0.001892%)	
ATGCATCATAAGGATACTAAATCCTATTTTCTGGTAAATTTTCATAATTTTTTGACACCTCTAGCTAGGTC<br>ATTTGACCTGATACAACATCGGATTTTCA	7 (0.000132%)	
ATGCCAGCCGTTCGTTTGCATGTTCCTTGACACTTTTCGTGCCGGGGTTTTGTGATATCCGGAAGCAACGC<br>GCACGACAAGACCGAGATAAAAGCTCCCG	11 (0.000208%)	
ATGCCCTTAGATGTTCTGGGCCGCACGCGCGCTACACTGATGTATTCAACGAGTTCACACCTTGGCCGACA<br>GGCCCGGGTAATCTTTGAAATTTCATCGT	6 (0.000114%)	
ATGCTAATGTATCCAGAGCGTAGGCTTGCTTTGAGCACTCTAATTTCTTCAAAGTAACAGCGCCGGAGGCA<br>CGACCCGGCCAATTAAGACCAGGAGCGTA	14 (0.000265%)	
ATGCTTGTCTCAAAGATTAAGCCATGCATGTGTAAGTATGAACGAATTCAGACTGTGAAACTGCGAATGGC<br>TCATTAAATCAGTTATAGTTTGTTTGATG	16 (0.000303%)	
ATGCTTTCCAACGAAGCACGCCCATCCAACCTAGGCGAGACAAGGGTTCACATTTCGTTCATCACCCTTGG<br>CCGGCTATCGAACAGCCGGACTCCCATCA	297 (0.005619%)	
ATGGACTTTGGCTACACCATGAAAGCTTTGAGAAGCAAGAAGAAGGTTGGTTAGTGTTTTGGAGTCGAATA<br>TGACTTGATGTCATGTGTATGATTGAGTA	81 (0.001532%)	
ATGGCTCATTAAATCAGTTATAGTTTGTTTGATGGTAACTACTACTCGGATAACCGTAGTAATTCTAGAGC<br>TAATACGTGCAACAAACCCCGACTTATGG	8 (0.000151%)	
ATGGGCATCGACACCTTGCGGCTAGGAACTGGAACGAGACGGGTGGCAAAGATTTCGAGTAGCACTTCATA<br>CTACCGTGGGTTTTTTAAACCTTCCGAGT	10 (0.000189%)	
ATGTATCCAGAGCGTAGGCTTGCTTTGAGCACTCTAATTTCTTCAAAGTAACAGCGCCGGAGGCACGACCC<br>GGCCAATTAAGACCAGGAGCGTATCGCCG	3 (0.000057%)	
ATGTCATGTGTATGATTGAGTATAAGAACTTAAACCGCAA	125 (0.000946%)	
ATGTCGGTACGCTCCAGGCGTCCTTGGCTCGGATTTAGGCCAACCGCGTGCGGTAACACACGGGAGACCAG<br>CTTCCGTCCCGCATCAGCAAAGGATGGTG	4 (0.000076%)	
ATGTGTAAGTATGAACGAATTCAGACTGTGAAACTGCGAATGGCTCATTAAATCAGTTATAGTTTGTTTGA<br>TGGTAACTACTACTCGGATAACCGTAGTA	10 (0.000189%)	
ATGTTATCCCATGCTAATGTATCCAGAGCGTAGGCTTGCTTTGAGCACTCTAATTTCTTCAAAGTAACAGC<br>GCCGGAGGCACGACCCGGCCAATTAAGAC	17 (0.000322%)	
ATGTTCTGGGCCGCACGCGCGCTACACTGATGTATTCAACGAGTTCACACCTTGGCCGACAGGCCCGGGTA<br>ATCTTTGAAATTTCATCGTGATGGGGATA	5 (0.000095%)	
ATTAAATCAGTTATAGTTTGTTTGATGGTAACTACTACTCGGATAACCGTAGTAATTCTAGAGCTAATACG<br>TGCAACAAACCCCGACTTATGGAAGGGAC	38 (0.000719%)	

ATTAACAGGGACAGTCGGGGGCATTCGTATTTCATAGTCAGAGGTGAAATTCTTGGATTTATGAAAGACGAACAACTGCGAAAGCATTTGCCAAGGATGT	102 (0.001930%)
ATTAAGCCATGCATGTGTAAGTATGAACGAATTCAGACTGTGAAACTGCGAATGGCTCATTAAATCAGTTATAGTTTGTTTGATGGTAACTACTACTCGG	19 (0.000359%)
ATTACCAGACTCGAAAGAGCCCGGTATTGTTATTTATTGTCACTACCTCCCCGTGTCAGGATTGGGTAATTTGCGCGCCTGCTGCCTTCCTTGGATGTGG	5 (0.000095%)
ATTAGGGTTCGATTCCGGAGAGGGAGCCTGAGAAACGGCTACCACATCCAAGGAAGGCAGCAGGCGCGCAAATTACCCAATCCTGACACGGGGAGGTAGT	6 (0.000114%)
ATTCAATCGGTAGGAGCGACGGGCGGTGTGTACAAAGGGCAGGGACGTAGTCAACGCGAGCTGATGACTCGCGCTTACTAGGAATTCCTCGTTGAAGACC	339 (0.006413%)
ATTCCAATTACCAGACTCGAAAGAGCCCGGTATTGTTATTTATTGTCACTACCTCCCCGTGTCAGGATTGGGTAATTTGCGCGCCTGCTGCCTTCCTTGG	23 (0.000435%)
ATTCCAGCTCCAATAGCGTATATTTAAGTTGTTGCAGTTAAAAAGCTCGTAGTTGAACCTTGGGATGGGTCGGCCGGTCCGCCTTTGGTGTGCATTGGTC	5 (0.000095%)
ATTCCCCGCCACATCCTCTCAAACGCAATGGAAAGAGAGAAAGGACGAGGTCTTGACCGTCATCTTTTGCCCGAAGGACGGATGAGCTTTGGCGGGACTG	10 (0.000189%)
ATTCGACTCCAAAACACTAACCAACCTTCTTCTTGCTTCTCAAAGCTTTCATGGTGTAGCCAAAGTCCATATGAGTCTTTGGCTTTGTGTCTTCTAACAA	112 (0.002119%)
ATTCGTACTGAAAATCAGAATCAAACGAGCTTTTACCCTTTTGTTCCACACGAGATTTCTGTTCTCGTTGAGCTCATCTTAGGACACCTGCGTTATCTTT	10 (0.000189%)
ATTCGTATTTCATAGTCAGAGGTGAAATTCTTGGATTTATGAAAGACGAACAACTGCGAAAGCATTTGCCAAGGATGTTTTCATTAATCAAGAACGAAAG	8 (0.000151%)
ATTGGAGGGCAAGTCTGGTGCCAGCAGCCGCGGTAATTCCAGCTCCAATAGCGTATATTTAAGTTGTTGCAGTTAAAAAGCTCGTAGTTGAACCTTGGGA	205 (0.003878%)
ATTGTACTCATTCCAATTACCAGACTCGAAAGAGCCCGGTATTGTTATTTATTGTCACTACCTCCCCGTGTCAGGATTGGGTAATTTGCGCGCCTGCTGC	8 (0.000151%)
ATTGTATCCTTGTTAGAAGACACAAAGCCAAAGACTCATATGGACTTTGGCTACACCATGAAAGCTTTGAGAAGCAAGAAGAAGGTTGGTTAGTGTTTTG	38 (0.000719%)
ATTGTCAGGTGGGGAGTTTGGCTGGGGCGGCACATCTGTTAAAAGATAACGCAGGTGTCCTAAGATGAGCTCAACGAGAACAGAAATCTCGTGTGGAACA	38 (0.000719%)
ATTGTTCCATCGACCAGAGGCTGTTCACCTTGGAGACCTGATGCGGTTATGAGTACGACCGGGCGTGAGCGGCACTCGGTCCTCCGGATTTTCAAGGGCC	13 (0.000246%)
ATTGTTGGTCTTCAACGAGGAATTCCTAGTAAGCGCGAGTCATCAGCTCGCGTTGACTACGTCCCTGCCCTTTGTACACACCGCCCGTCGCTCCTACCGA	40 (0.000757%)
ATTTAGATTGTACTCATTCCAATTACCAGACTCGAAAGAGCCCGGTATTGTTATTTATTGTCACTACCTCCCCGTGTCAGGATTGGGTAATTTGCGCGCC	18 (0.000341%)
ATTTCATAGTCAGAGGTGAAATTCTTGGATTTATGAAAGACGAACAACTGCGAAAGCATTTGCCAAGGATGTTTTCATTAATCAAGAACGAAAGTTGGGG	10 (0.000189%)
ATTTCGTTCATCACCCTTGGCCGGCTATCGAACAGCCGGACTCCCATCAAAAGATGGTTGCCAAGAACATCTTCGTTACGGTTTGCTAATTCTCGGAATA	24 (0.000454%)
ATTTTCATAATTTTTTGACACCTCTAGCTAGGTCATTTGACCTGATACAACATCGGATTTTCATGGTCTAGTTGGGGCTCCGTGGGCATATTTGATGCAA	4 (0.000076%)
ATTTTCTGGTAAATTTTCATAATTTTTTGACACCTCTAGCTAGGTCATTTGACCTGATACAACATCGGATTTTCATGGTCTAGTTGGGGCTCCGTGGGCA	8 (0.000151%)
CAAAAAGCAACGTCGCTATGAACGCTTGGCTGCCACAAGCCAGTTATCCCTGTGGTAACTTTTCTGACACCTCTAGCTTCAAATTCCGAAGGTCTAAAGG	1357 (0.025672%)
CAAACGCAATGGAAAGAGAGAAAGGACGAGGTCTTGACCGTCATCTTTTGCCCGAAGGACGGATGAGCTTTGGCGGGACTGAATCACTTCGAGTCACCGT	6 (0.000114%)
CAAAGACTCATATGGACTTTGGCTACACCATGAAAGCTTTGAGAAGCAAGAAGAAGGTTGGTTAGTGTTTTGGAGTCGAATATGACTTGATGTCATGTGT	119 (0.002251%)
CAAAGATTAAGCCATGCATGTGTAAGTATGAACGAATTCAGACTGTGAAACTGCGAATGGCTCATTAAATCAGTTATAGTTTGTTTGATGGTAACTACTA	64 (0.001211%)
CAAAGCCAAAGACTCATATGGACTTTGGCTACACCATGAAAGCTTTGAGAAGCAAGAAGAAGGTTGGTTAGTGTTTTGGAGTCGAATATGACTTGATGTC	108 (0.002043%)
CAACCTAGGCGAGACAAGGGTTCACATTTCGTTCATCACCCTTGGCCGGCTATCGAACAGCCGGACTCCCATCAAAAGATGGTTGCCAAGAACATCTTCG	9 (0.000170%)
CAACCTAGGCGAGACAAGGGTTCACATTTCGTTCATCACCCTTGGCCGGCTTTCGAACAGCCGGACTCCCATCAAAAGATGGTTGCCAAGAACATCTTCG	5 (0.000095%)
CAACGAAGCACGCCCATCCAACCTAGGCGAGACAAGGGTTCACATTTCGTTCATCACCCTTGGCCGGCTATCGAACAGCCGGACTCCCATCAAAAGATGG	67 (0.001268%)
CAACGAAGCACGCCCATCCAACCTAGGCGAGACAAGGGTTCACATTTCGTTCATCACCCTTGGCCGGCTTTCGAACAGCCGGACTCCCATCAAAAGATGG	86 (0.001627%)
CAACGAGGAATTCCTAGTAAGCGCGAGTCATCAGCTCGCGTTGACTACGTCCCTGCCCTTTGTACACACCGCCCGTCGCTCCTACCGATTGAATGATCGA	6 (0.000114%)
CAACGGGCAGAGCCCGCGTCGACCTTTTATCTAATAAATGCGTCCCTTCCATAAGTCGGGGTTTGTTGCACGTATTAGCTCTAGAATTACTACGGTTATC	19 (0.000359%)
CAACGTCGCTATGAACGCTTGGCTGCCACAAGCCAGTTATCCCTGTGGTAACTTTTCTGACACCTCTAGCTTCAAATTCCGAAGGTCTAAAGGATCGATC	22 (0.000416%)
CAAGGGTTCACATTTCGTTCATCACCCTTGGCCGGCTATCGAACAGCCGGACTCCCATCAAAAGATGGTTGCCAAGAACATCTTCGTTACGGTTTGCTAA	22 (0.000416%)
CAAGGGTTCACATTTCGTTCATCACCCTTGGCCGGCTTTCGAACAGCCGGACTCCCATCAAAAGATGGTTGCCAAGAACATCTTCGTTACGGTTTGCTAA	35 (0.000662%)
CAAGTCATATTCGACTCCAAAACACTAACCAACCTTCTTCTTGCTTCTCAAAGCTTTCATGGTGTAGCCAAAGTCCATATGAGTCTTTGGCTTTGTGTCT	142 (0.002686%)
CAAGTCTGGTGCCAGCAGCCGCGGTAATTCCAGCTCCAATAGCGTATATTTAAGTTGTTGCAGTTAAAAAGCTCGTAGTTGAACCTTGGGATGGGTCGGC	24 (0.000454%)
CAAGTTCACCACCGCATGTCGGTACGCTCCAGGCGTCCTTGGCTCGGATTTAGGCCAACCGCGTGCGGTAACACACGGGAGACCAGCTTCCGTCCCGCAT	18 (0.000341%)
CAATAACAGGTCTGTGATGCCCTTAGATGTTCTGGGCCGCACGCGCGCTACACTGATGTATTCAACGAGTTCACACCTTGGCCGACAGGCCCGGGTAATC	36 (0.000681%)
CAATCATACACATGACAACAAGTCATATTCGACTCCAAAACACTAACCAACCTTCTTCTTGCTTCTCAAAGCTTTCATGGTGTAGCCAAAGTCCATATGA	24 (0.000454%)
CAATCATACACATGACATCAAGTCATATTCGACTCCAAAACACTAACCAACCTTCTTCTTGCTTCTCAAAGCTTTCATGGTGTAGCCAAAGTCCATATGA	178 (0.003367%)
CAATCGGTAGGAGCGACGGGCGGTGTGTACAAAGGGCAGGGACGTAGTCAACGCGAGCTGATGACTCGCGCTTACTAGGAATTCCTCGTTGAAGACCAAC	107 (0.002024%)

CAATTACCAGACTCGAAAGAGCCCGGTATTGTTATTTATTGTCACTACCTCCCCGTGTCAGGATTGGGTAA TTTGCGCGCCTGCTGCCTTCCTTGGATGT	26 (0.000492%)	
CAATTCCCCGCCACATCCTCTCAAACGCAATGGAAAGAGAGAAAGGACGAGGTCTTGACCGTCATCTTTTG CCCGAAGGACGGATGAGCTTTGGCGGGAC	35 (0.000662%)	
CACAAAGCCAAAGACTCATATGGACTTTGGCTACACCATGAAAGCTTTGAGAAGCAAGAAGAAGGTTGGTT AGTGTTTTGGAGTCGAATATGACTTGATC	15 (0.000284%)	
CACAAAGCCAAAGACTCATATGGACTTTGGCTACACCATGAAAGCTTTGAGAAGCAAGAAGAAGGTTGGTT AGTGTTTTGGAGTCGAATATGACTTGATG	40 (0.000757%)	
CACATCCTCTCAAACGCAATGGAAAGAGAGAAAGGACGAGGTCTTGACCGTCATCTTTTGCCCGAAGGACG GATGAGCTTTGGCGGGACTGAATCACTTC	5 (0.000095%)	
CACATCTGTTAAAAGATAACGCAGGTGTCCTAAGATGAGCTCAACGAGAACAGAAATCTCGTGTGGAACAA AAGGGTAAAAGCTCGTTTGATTCTGATTT	7 (0.000132%)	
CACATGACATCAAGTCATATTCGACTCCAAAACACTAACCAACCTTCTTCTTGCTTCTCAAAGCTTTCATG GTGTAGCCAAAGTCCATATGAGTCTTTGG	49 (0.000927%)	
CACATTTCGTTCATCACCCTTGGCCGGCTATCGAACAGCCGGACTCCCATCAAAAGATGGTTGCCAAGAAC ATCTTCGTTACGGTTTGCTAATTCTCGGA	5 (0.000095%)	
CACATTTCGTTCATCACCCTTGGCCGGCTTTCGAACAGCCGGACTCCCATCAAAAGATGGTTGCCAAGAAC ATCTTCGTTACGGTTTGCTAATTCTCGGA	4 (0.000076%)	
CACCACCGCATGTCGGTACGCTCCAGGCGTCCTTGGCTCGGATTTAGGCCAACCGCGTGCGGTAACACACG GGAGACCAGCTTCCGTCCCGCATCAGCAA	29 (0.000549%)	
CACCATCCTTTGCTGATGCGGGACGGAAGCTGGTCTCCCGTGTGTTACCGCACGCGGTTGGCCTAAATCCG AGCCAAGGACGCCTGGAGCGTACCGACAT	7 (0.000132%)	
CACCCGTTGACTCGCACACATGTCAGACTCCTTGGTCCGTGTTTCAAGACGGGTCGAATGGGGAGCCCACA GGCCGACGCCCGGAGCACGCTGATGCCGA	12 (0.000227%)	
CACCCTAACGCCTCGAAGAACTAATGGCAGCCCACGCAAGGCAAGCCCATTCTCCTCGACGATTCAGCAGT TTTTGTCCGAGAACTGCTGAGAAAACTCG	8 (0.000151%)	
CACGCCCATCCAACCTAGGCGAGACAAGGGTTCACATTTCGTTCATCACCCTTGGCCGGCTATCGAACAGC CGGACTCCCATCAAAAGATGGTTGCCAAG	26 (0.000492%)	
CACGCCCATCCAACCTAGGCGAGACAAGGGTTCACATTTCGTTCATCACCCTTGGCCGGCTTTCGAACAGC CGGACTCCCATCAAAAGATGGTTGCCAAG	26 (0.000492%)	
CACGCGCCTAACGGCGTGCCTCGGCATCAGCGTGCTCCGGGCGTCGGCCTGTGGGCTCCCCATTCGACCCG TCTTGAAACACGGACCAAGGAGTCTGACA	103 (0.001949%)	
CACGCTTTCACGGTTCGTATTCGTACTGAAAATCAGAATCAAACGAGCTTTTACCCTTTTGTTCCACACGA GATTTCTGTTCTCGTTGAGCTCATCTTAG	103 (0.001949%)	
CACGGCAATTCCCCGCCACATCCTCTCAAACGCAATGGAAAGAGAGAAAGGACGAGGTCTTGACCGTCATC TTTTGCCCGAAGGACGGATGAGCTTTGGC	11 (0.000208%)	
CACGGTTCGTATTCGTACTGAAAATCAGAATCAAACGAGCTTTTACCCTTTTGTTCCACACGAGATTTCTG TTCTCGTTGAGCTCATCTTAGGACACCTG	4 (0.000076%)	
CAGAATCAAACGAGCTTTTACCCTTTTGTTCCACACGAGATTTCTGTTCTCGTTGAGCTCATCTTAGGACA CCTGCGTTATCTTTTAACAGATGTGCCGC	17 (0.000322%)	
CAGACTCGAAAGAGCCCGGTATTGTTATTTATTGTCACTACCTCCCCGTGTCAGGATTGGGTAATTTGCGC GCCTGCTGCCTTCCTTGGATGTGGTAGCC	12 (0.000227%)	
CAGAGCAACGGGCAGAGCCCGCGTCGACCTTTTATCTAATAAATGCGTCCCTTCCATAAGTCGGGGTTTGT TGCACGTATTAGCTCTAGAATTACTACGG	11 (0.000208%)	
CAGAGCCCGCGTCGACCTTTTATCTAATAAATGCGTCCCTTCCATAAGTCGGGGTTTGTTGCACGTATTAG CTCTAGAATTACTACGGTTATCCGAGTAG	13 (0.000246%)	
CAGAGCGTAGGCTTGCTTTGAGCACTCTAATTTCTTCAAAGTAACAGCGCCGGAGGCACGACCCGGCCAAT TAAGACCAGGAGCGTATCGCCGACCGAAG	29 (0.000549%)	
CAGAGGCTGTTCACCTTGGAGACCTGATGCGGTTATGAGTACGACCGGGCGTGAGCGGCACTCGGTCCTCC GGATTTTCAAGGGCCGCCGGGGGCGCACC	10 (0.000189%)	
CAGAGGTGAAATTCTTGGATTTATGAAAGACGAACAACTGCGAAAGCATTTGCCAAGGATGTTTTCATTAA TCAAGAACGAAAGTTGGGGGCTCGAAGAC	21 (0.000397%)	
CAGCACGCGCCTAACGGCGTGCCTCGGCATCAGCGTGCTCCGGGCGTCGGCCTGTGGGCTCCCCATTCGAC CCGTCTTGAAACACGGACCAAGGAGTCTG	65 (0.001230%)	
CAGCAGCCGCGGTAATTCCAGCTCCAATAGCGTATATTTAAGTTGTTGCAGTTAAAAAGCTCGTAGTTGAA CCTTGGGATGGGTCGGCCGGTCCGCCTTT	21 (0.000397%)	
CAGCCGCGGTAATTCCAGCTCCAATAGCGTATATTTAAGTTGTTGCAGTTAAAAAGCTCGTAGTTGAACCT TGGGATGGGTCGGCCGGTCCGCCTTTGGT	7 (0.000132%)	
CAGCGTGCTCCGGGCGTCGGCCTGTGGGCTCCCCATTCGACCCGTCTTGAAACACGGACCAAGGAGTCTGA CATGTGTGCGAGTCAACGGGTGAGTAAAC	7 (0.000132%)	
CAGGGACAGTCGGGGGCATTCGTATTTCATAGTCAGAGGTGAAATTCTTGGATTTATGAAAGACGAACAAC TGCGAAAGCATTTGCCAAGGATGTTTTCA	37 (0.000700%)	
CAGGTCTGTGATGCCCTTAGATGTTCTGGGCCGCACGCGCGCTACACTGATGTATTCAACGAGTTCACACC TTGGCCGACAGGCCCGGGTAATCTTTGAA	15 (0.000284%)	
CAGGTGGGGAGTTTGGCTGGGGCGGCACATCTGTTAAAAGATAACGCAGGTGTCCTAAGATGAGCTCAACG AGAACAGAAATCTCGTGTGGAACAAAAGG	31 (0.000586%)	
CAGTAGTCATATGCTTGTCTCAAAGATTAAGCCATGCATGTGTAAGTATGAACGAATTCAGACTGTGAAAC TGCGAATGGCTCATTAAATCAGTTATAGT	141 (0.002667%)	
CAGTCGGGGGCATTCGTATTTCATAGTCAGAGGTGAAATTCTTGGATTTATGAAAGACGAACAACTGCGAA AGCATTTGCCAAGGATGTTTTCATTAATC	5 (0.000095%)	
CAGTTATAGTTTGTTTGATGGTAACTACTACTCGGATAACCGTAGTAATTCTAGAGCTAATACGTGCAACA AACCCCGACTTATGGAAGGGACGCATTTA	4 (0.000076%)	
CATAAGGATACTAAATCCTATTTTCTGGTAAATTTTCATAATTTTTTGACACCTCTAGCTAGGTCATTTGA CCTGATACAACATCGGATTTTCATGGTCT	19 (0.000359%)	
CATACACATGACATCAAGTCATATTCGACTCCAAAACACTAACCAACCTTCTTCTTGCTTCTCAAAGCTTT CATGGTGTAGCCAAAGTCCATATGAGTCT	52 (0.000984%)	
CATAGTCAGAGGTGAAATTCTTGGATTTATGAAAGACGAACAACTGCGAAAGCATTTGCCAAGGATGTTTT CATTAATCAAGAACGAAAGTTGGGGGCTC	19 (0.000359%)	
CATATGCTTGTCTCAAAGATTAAGCCATGCATGTGTAAGTATGAACGAATTCAGACTGTGAAACTGCGAAT GGCTCATTAAATCAGTTATAGTTTGTTTG	47 (0.000889%)	
CATATGGACTTTGGCTACACCATGAAAGCTTTGAGAAGCAAGAAGAAGGTTGGTTAGTGTTTTGGAGTCGA ATATGACTTGATGTCATGTGTATGATTGA	14 (0.000265%)	
CATATTCGACTCCAAAACACTAACCAACCTTCTTCTTGCTTCTCAAAGCTTTCATGGTGTAGCCAAAGTCC ATATGAGTCTTTGGCTTTGTGTCTTCTAA	92 (0.001740%)	
CATCAAGTCATATTCGACTCCAAAACACTAACCAACCTTCTTCTTGCTTCTCAAAGCTTTCATGGTGTAGC CAAAGTCCATATGAGTCTTTGGCTTTGTG	51 (0.000965%)	

CATCAGAGCAACGGGCAGAGCCCGCGTCGACCTTTTATCTAATAAATGCGTCCCTTCCATAAGTCGGGGTT<br>TGTTGCACGTATTAGCTCTAGAATTACTA	33 (0.000624%)	
CATCAGCGTGCTCCGGGCGTCGGCCTGTGGGCTCCCCATTCGACCCGTCTTGAAACACGGACCAAGGAGTC<br>TGACATGTGTGCGAGTCAACGGGTGAGTA	11 (0.000208%)	
CATCCAACCTAGGCGAGACAAGGGTTCACATTTCGTTCATCACCCTTGGCCGGCTATCGAACAGCCGGACT<br>CCCATCAAAAGATGGTTGCCAAGAACATC	5 (0.000095%)	
CATCCAACCTAGGCGAGACAAGGGTTCACATTTCGTTCATCACCCTTGGCCGGCTTTCGAACAGCCGGACT<br>CCCATCAAAAGATGGTTGCCAAGAACATC	5 (0.000095%)	
CATCCTCTCAAACGCAATGGAAAGAGAGAAAGGACGAGGTCTTGACCGTCATCTTTTGCCCGAAGGACGGA<br>TGAGCTTTGGCGGGACTGAATCACTTCGA	3 (0.000057%)	
CATCCTTTGCTGATGCGGGACGGAAGCTGGTCTCCCGTGTGTTACCGCACGCGGTTGGCCTAAATCCGAGC<br>CAAGGACGCCTGGAGCGTACCGACATGCG	134 (0.002535%)	
CATCGACACCTTGCGGCTAGGAACTGGAACGAGACGGGTGGCAAAGATTTCGAGTAGCACTTCATACTACC<br>GTGGGTTTTTTAAACCTTCCGAGTTTTGT	4 (0.000076%)	
CATCGACCAGAGGCTGTTCACCTTGGAGACCTGATGCGGTTATGAGTACGACCGGGCGTGAGCGGCACTCG<br>GTCCTCCGGATTTTCAAGGGCCGCCGGGG	9 (0.000170%)	
CATGAATCATCAGAGCAACGGGCAGAGCCCGCGTCGACCTTTTATCTAATAAATGCGTCCCTTCCATAAGT<br>CGGGGTTTGTTGCACGTATTAGCTCTAGA	165 (0.003122%)	
CATGACATCAAGTCATATTCGACTCCAAAACACTAACCAACCTTCTTCTTGCTTCTCAAAGCTTTCATGGT<br>GTAGCCAAAGTCCATATGAGTCTTTGGCT	38 (0.000719%)	
CATGCATGTGTAAGTATGAACGAATTCAGACTGTGAAACTGCGAATGGCTCATTAAATCAGTTATAGTTTG<br>TTTGATGGTAACTACTACTCGGATAACCG	7 (0.000132%)	
CATGCTAATGTATCCAGAGCGTAGGCTTGCTTTGAGCACTCTAATTTCTTCAAAGTAACAGCGCCGGAGGC<br>ACGACCCGGCCAATTAAGACCAGGAGCGT	5 (0.000095%)	
CATGCTTTCCAACGAAGCACGCCCATCCAACCTAGGCGAGACAAGGGTTCACATTTCGTTCATCACCCTTG<br>GCCGGCTATCGAACAGCCGGACTCCCATC	11 (0.000208%)	
CATGGGCATCGACACCTTGCGGCTAGGAACTGGAACGAGACGGGTGGCAAAGATTTCGAGTAGCACTTCAT<br>ACTACCGTGGGTTTTTTAAACCTTCCGAG	3 (0.000057%)	
CATGTCGGTACGCTCCAGGCGTCCTTGGCTCGGATTTAGGCCAACCGCGTGCGGTAACACACGGGAGACCA<br>GCTTCCGTCCCGCATCAGCAAAGGATGGT	4 (0.000076%)	
CATTAAATCAGTTATAGTTTGTTTGATGGTAACTACTACTCGGATAACCGTAGTAATTCTAGAGCTAATAC<br>GTGCAACAAACCCCGACTTATGGAAGGGA	14 (0.000265%)	
CATTCAATCGGTAGGAGCGACGGGCGGTGTGTACAAAGGGCAGGGACGTAGTCAACGCGAGCTGATGACTC<br>GCGCTTACTAGGAATTCCTCGTTGAAGAC	15 (0.000284%)	
CATTCCAATTACCAGACTCGAAAGAGCCCGGTATTGTTATTTATTGTCACTACCTCCCCGTGTCAGGATTG<br>GGTAATTTGCGCGCCTGCTGCCTTCCTTG	7 (0.000132%)	
CATTCGTATTTCATAGTCAGAGGTGAAATTCTTGGATTTATGAAAGACGAACAACTGCGAAAGCATTTGCC<br>AAGGATGTTTTCATTAATCAAGAACGAAA	8 (0.000151%)	
CATTGGAGGGCAAGTCTGGTGCCAGCAGCCGCGGTAATTCCAGCTCCAATAGCGTATATTTAAGTTGTTGC<br>AGTTAAAAAGCTCGTAGTTGAACCTTGGG	30 (0.000568%)	
CATTGTCAGGTGGGGAGTTTGGCTGGGGCGGCACATCTGTTAAAAGATAACGCAGGTGTCCTAAGATGAGC<br>TCAACGAGAACAGAAATCTCGTGTGGAAC	8 (0.000151%)	
CATTGTTCCATCGACCAGAGGCTGTTCACCTTGGAGACCTGATGCGGTTATGAGTACGACCGGGCGTGAGC<br>GGCACTCGGTCCTCCGGATTTTCAAGGGC	28 (0.000530%)	
CCAAAGACTCATATGGACTTTGGCTACACCATGAAAGCTTTGAGAAGCAAGAAGAAGGTTGGTTAGTGTTT<br>TGGAGTCGAATATGACTTGATGTCATGTG	205 (0.003878%)	
CCAACCTAGGCGAGACAAGGGTTCACATTTCGTTCATCACCCTTGGCCGGCTATCGAACAGCCGGACTCCC<br>ATCAAAAGATGGTTGCCAAGAACATCTTC	18 (0.000341%)	
CCAACCTAGGCGAGACAAGGGTTCACATTTCGTTCATCACCCTTGGCCGGCTTTCGAACAGCCGGACTCCC<br>ATCAAAAGATGGTTGCCAAGAACATCTTC	27 (0.000511%)	
CCAACGAAGCACGCCCATCCAACCTAGGCGAGACAAGGGTTCACATTTCGTTCATCACCCTTGGCCGGCTA<br>TCGAACAGCCGGACTCCCATCAAAAGATG	15 (0.000284%)	
CCAACGAAGCACGCCCATCCAACCTAGGCGAGACAAGGGTTCACATTTCGTTCATCACCCTTGGCCGGCTT<br>TCGAACAGCCGGACTCCCATCAAAAGATG	9 (0.000170%)	
CCAATTACCAGACTCGAAAGAGCCCGGTATTGTTATTTATTGTCACTACCTCCCCGTGTCAGGATTGGGTA<br>ATTTGCGCGCCTGCTGCCTTCCTTGGATG	5 (0.000095%)	
CCACCCTAACGCCTCGAAGAACTAATGGCAGCCCACGCAAGGCAAGCCCATTCTCCTCGACGATTCAGCAG<br>TTTTTGTCCGAGAACTGCTGAGAAAACTC	7 (0.000132%)	
CCACCGCATGTCGGTACGCTCCAGGCGTCCTTGGCTCGGATTTAGGCCAACCGCGTGCGGTAACACACGGG<br>AGACCAGCTTCCGTCCCGCATCAGCAAAG	5 (0.000095%)	
CCACGCTTTCACGGTTCGTATTCGTACTGAAAATCAGAATCAAACGAGCTTTTACCCTTTTGTTCCACACG<br>AGATTTCTGTTCTCGTTGAGCTCATCTTA	67 (0.001268%)	
CCAGACTCGAAAGAGCCCGGTATTGTTATTTATTGTCACTACCTCCCCGTGTCAGGATTGGGTAATTTGCG<br>CGCCTGCTGCCTTCCTTGGATGTGGTAGC	12 (0.000227%)	
CCAGAGCGTAGGCTTGCTTTGAGCACTCTAATTTCTTCAAAGTAACAGCGCCGGAGGCACGACCCGGCCAA<br>TTAAGACCAGGAGCGTATCGCCGACCGAA	21 (0.000397%)	
CCAGAGGCTGTTCACCTTGGAGACCTGATGCGGTTATGAGTACGACCGGGCGTGAGCGGCACTCGGTCCTC<br>CGGATTTTCAAGGGCCGCCGGGGGCGCAC	15 (0.000284%)	
CCAGCAGCCGCGGTAATTCCAGCTCCAATAGCGTATATTTAAGTTGTTGCAGTTAAAAAGCTCGTAGTTGA<br>ACCTTGGGATGGGTCGGCCGGTCCGCCTT	14 (0.000265%)	
CCAGCCGTTCGTTTGCATGTTCCTTGACACTTTTCGTGCCGGGGTTTTGTGATATCCGGAAGCAACGCGCA<br>CGACAAGACCGAGATAAAAGCTCCCGATC	4 (0.000076%)	
CCAGTAGTCATATGCTTGTCTCAAAGATTAAGCCATGCATGTGTAAGTATGAACGAATTCAGACTGTGAAA<br>CTGCGAATGGCTCATTAAATCAGTTATAG	70 (0.001324%)	
CCATCCTTTGCTGATGCGGGACGGAAGCTGGTCTCCCGTGTGTTACCGCACGCGGTTGGCCTAAATCCGAG<br>CCAAGGACGCCTGGAGCGTACCGACATGC	8 (0.000151%)	
CCATCGACCAGAGGCTGTTCACCTTGGAGACCTGATGCGGTTATGAGTACGACCGGGCGTGAGCGGCACTC<br>GGTCCTCCGGATTTTCAAGGGCCGCCGGG	9 (0.000170%)	
CCATGCTAATGTATCCAGAGCGTAGGCTTGCTTTGAGCACTCTAATTTCTTCAAAGTAACAGCGCCGGAGG<br>CACGACCCGGCCAATTAAGACCAGGAGCG	8 (0.000151%)	
CCATGCTTTCCAACGAAGCACGCCCATCCAACCTAGGCGAGACAAGGGTTCACATTTCGTTCATCACCCTT<br>GGCCGGCTATCGAACAGCCGGACTCCCAT	34 (0.000643%)	
CCATGGGCATCGACACCTTGCGGCTAGGAACTGGAACGAGACGGGTGGCAAAGATTTCGAGTAGCACTTCA<br>TACTACCGTGGGTTTTTTAAACCTTCCGA	6 (0.000114%)	
CCATTGGAGGGCAAGTCTGGTGCCAGCAGCCGCGGTAATTCCAGCTCCAATAGCGTATATTTAAGTTGTTG<br>CAGTTAAAAAGCTCGTAGTTGAACCTTGG	19 (0.000359%)	

CCCATCCAACCTAGGCGAGACAAGGGTTCACATTTCGTTCATCACCCTTGGCCGGCTATCGAACAGCCGGA<br>CTCCCATCAAAAGATGGTTGCCAAGAACA	16 (0.000303%)
CCCATCCAACCTAGGCGAGACAAGGGTTCACATTTCGTTCATCACCCTTGGCCGGCTTTCGAACAGCCGGA<br>CTCCCATCAAAAGATGGTTGCCAAGAACA	9 (0.000170%)
CCCATGCTAATGTATCCAGAGCGTAGGCTTGCTTTGAGCACTCTAATTTCTTCAAAGTAACAGCGCCGGAG<br>GCACGACCCGGCCAATTAAGACCAGGAGC	6 (0.000114%)
CCCATGGGCATCGACACCTTGCGGCTAGGAACTGGAACGAGACGGGTGGCAAAGATTTCGAGTAGCACTTC<br>ATACTACCGTGGGTTTTTTAAACCTTCCG	14 (0.000265%)
CCCCGCCACATCCTCTCAAACGCAATGGAAAGAGAGAAAGGACGAGGTCTTGACCGTCATCTTTTGCCCGA<br>AGGACGGATGAGCTTTGGCGGGACTGAAT	6 (0.000114%)
CCCGAGTGTGAGCGAGGTGTGAGTGTCGCCCATGGGCATCGACACCTTGCGGCTAGGAACTGGAACGAGAC<br>GGGTAGCAAAGATTTCGAGTAGCACTTCA	15 (0.000284%)
CCCGAGTGTGAGCGAGGTGTGAGTGTCGCCCATGGGCATCGACACCTTGCGGCTAGGAACTGGAACGAGAC<br>GGGTGGCAAAGATTTCGAGTAGCACTTCA	18 (0.000341%)
CCCGCCACATCCTCTCAAACGCAATGGAAAGAGAGAAAGGACGAGGTCTTGACCGTCATCTTTTGCCCGAA<br>GGACGGATGAGCTTTGGCGGGACTGAATC	9 (0.000170%)
CCCGCGTCGACCTTTTATCTAATAAATGCGTCCCTTCCATAAGTCGGGGTTTGTTGCACGTATTAGCTCTA<br>GAATTACTACGGTTATCCGAGTAGTAGTT	44 (0.000832%)
CCCGGCCGTCGGGGCAAGAGCCAGGCCTCGATGAGTAGGAGGGCGCGGCGGTCGCTGCAAAACCTAGGGCG<br>CGAGCCCGGGCGGAGCGGCCGTCGGTGCA	21 (0.000397%)
CCCTAACGCCTCGAAGAACTAATGGCAGCCCACGCAAGGCAAGCCCATTCTCCTCGACGATTCAGCAGTTT<br>TTGTCCGAGAACTGCTGAGAAAACTCGGA	11 (0.000208%)
CCCTCACCATCCTTTGCTGATGCGGGACGGAAGCTGGTCTCCCGTGTGTTACCGCACGCGGTTGGCCTAAA<br>TCCGAGCCAAGGACGCCTGGAGCGTACCG	16 (0.000303%)
CCCTGTTGAGCTTGACTCTAGTCCGACTTTGTGAAATGACTTGAGAGGTGTAGGATAAGTGGGAGCTTCGG<br>CGCAAGTGAAATACCACTACTTTTAACGT	13 (0.000246%)
CCCTTAGATGTTCTGGGCCGCACGCGCGCTACACTGATGTATTCAACGAGTTCACACCTTGGCCGACAGGC<br>CCGGGTAATCTTTGAAATTTCATCGTGAT	4 (0.000076%)
CCCTTGCCTACATTGTTCCATCGACCAGAGGCTGTTCACCTTGGAGACCTGATGCGGTTATGAGTACGACC<br>GGGCGTGAGCGGCACTCGGTCCTCCGGAT	30 (0.000568%)
CCGACTTCCCTTGCCTACATTGTTCCATCGACCAGAGGCTGTTCACCTTGGAGACCTGATGCGGTTATGAG<br>TACGACCGGGCGTGAGCGGCACTCGGTCC	52 (0.000984%)
CCGACTTTGTGAAATGACTTGAGAGGTGTAGGATAAGTGGGAGCTTCGGCGCAAGTGAAATACCACTACTT<br>TTAACGTTATTTTACTTACTCCGTGAATC	21 (0.000397%)
CCGAGTGTGAGCGAGGTGTGAGTGTCGCCCATGGGCATCGACACCTTGCGGCTAGGAACTGGAACGAGACG<br>GGTAGCAAAGATTTCGAGTAGCACTTCAT	10 (0.000189%)
CCGAGTGTGAGCGAGGTGTGAGTGTCGCCCATGGGCATCGACACCTTGCGGCTAGGAACTGGAACGAGACG<br>GGTGGCAAAGATTTCGAGTAGCACTTCAT	18 (0.000341%)
CCGCCACATCCTCTCAAACGCAATGGAAAGAGAGAAAGGACGAGGTCTTGACCGTCATCTTTTGCCCGAAG<br>GACGGATGAGCTTTGGCGGGACTGAATCA	7 (0.000132%)
CCGCCTAGGCTGTCCCGAGTGTGAGCGAGGTGTGAGTGTCGCCCATGGGCATCGACACCTTGCGGCTAGGA<br>ACTGGAACGAGACGGGTAGCAAAGATTTC	27 (0.000511%)
CCGCCTAGGCTGTCCCGAGTGTGAGCGAGGTGTGAGTGTCGCCCATGGGCATCGACACCTTGCGGCTAGGA<br>ACTGGAACGAGACGGGTGGCAAAGATTTC	62 (0.001173%)
CCGCGTCGACCTTTTATCTAATAAATGCGTCCCTTCCATAAGTCGGGGTTTGTTGCACGTATTAGCTCTAG<br>AATTACTACGGTTATCCGAGTAGTAGTTA	13 (0.000246%)
CCGCTAAGGAGTGTGTAACAACTCACCTGCCGAATCAACTAGCCCCGAAAATGGATGGCGCTTAAGCGCGC<br>GACCTATACCCGGCCGTCGGGGCAAGAGC	19 (0.000359%)
CCGGATTGCTCCGTTCCGCATCCGACCAGGACGCATCGCCGGCCCCCATCCGCTTCCCTCCCGACAATTTC<br>AAGCACTCTTTGACTCTCTTTTCAAAGTC	49 (0.000927%)
CCGGCCGTCGGGGCAAGAGCCAGGCCTCGATGAGTAGGAGGGCGCGGCGGTCGCTGCAAAACCTAGGGCGC<br>GAGCCCGGGCGGAGCGGCCGTCGGTGCAG	10 (0.000189%)
CCGGGCGTCGGCCTGTGGGCTCCCCATTCGACCCGTCTTGAAACACGGACCAAGGAGTCTGACATGTGTGC<br>GAGTCAACGGGTGAGTAAACCCGTAAGGC	13 (0.000246%)
CCGGGGAGGCGAATGCCAGCCGTTCGTTTGCATGTTCCTTGACACTTTTCGTGCCGGGGTTTTGTGATATC<br>CGGAAGCAACGCGCACGACAAGACCGAGA	7 (0.000132%)
CCGTAACTTCGGGAAAAGGATTGGCTCTGAGGGCTGGGCTCGGGGGTCCCAGTTCCGAACCCGTCGGCTGT<br>CAGCGGACTGCTCGAGCTGCTTCCGCGGC	3 (0.000057%)
CCGTCGAGTTATCATGAATCATCAGAGCAACGGGCAGAGCCCGCGTCGACCTTTTATCTAATAAATGCGTC<br>CCTTCCATAAGTCGGGGTTTGTTGCACGT	21 (0.000397%)
CCGTCTCCGGGGAGGCGAATGCCAGCCGTTCGTTTGCATGTTCCTTGACACTTTTCGTGCCGGGGTTTTGT<br>GATATCCGGAAGCAACGCGCACGACAAGA	7 (0.000132%)
CCTAACGCCTCGAAGAACTAATGGCAGCCCACGCAAGGCAAGCCCATTCTCCTCGACGATTCAGCAGTTTT<br>TGTCCGAGAACTGCTGAGAAAACTCGGAA	18 (0.000341%)
CCTAACGGCGTGCCTCGGCATCAGCGTGCTCCGGGCGTCGGCCTGTGGGCTCCCCATTCGACCCGTCTTGA<br>AACACGGACCAAGGAGTCTGACATGTGTG	4 (0.000076%)
CCTAAGTAGTGTTTCCTTGTTAGAAGACACAAAGCCAAAGACTCATATGGACTTTGGCTACACCATGAAAG<br>CTTTGAGAAGCAAGAAGAAGGTTGGTTAG	66 (0.001249%)
CCTACATTGTTCCATCGACCAGAGGCTGTTCACCTTGGAGACCTGATGCGGTTATGAGTACGACCGGGCGT<br>GAGCGGCACTCGGTCCTCCGGATTTTCAA	13 (0.000246%)
CCTAGGCGAGACAAGGGTTCACATTTCGTTCATCACCCTTGGCCGGCTATCGAACAGCCGGACTCCCATCA<br>AAAGATGGTTGCCAAGAACATCTTCGTTA	6 (0.000114%)
CCTAGGCGAGACAAGGGTTCACATTTCGTTCATCACCCTTGGCCGGCTTTCGAACAGCCGGACTCCCATCA<br>AAAGATGGTTGCCAAGAACATCTTCGTTA	12 (0.000227%)
CCTAGGCTGTCCCGAGTGTGAGCGAGGTGTGAGTGTCGCCCATGGGCATCGACACCTTGCGGCTAGGAACT<br>GGAACGAGACGGGTAGCAAAGATTTCGAG	39 (0.000738%)
CCTAGGCTGTCCCGAGTGTGAGCGAGGTGTGAGTGTCGCCCATGGGCATCGACACCTTGCGGCTAGGAACT<br>GGAACGAGACGGGTGGCAAAGATTTCGAG	73 (0.001381%)
CCTATACCCGGCCGTCGGGGCAAGAGCCAGGCCTCGATGAGTAGGAGGGCGCGGCGGTCGCTGCAAAACCT<br>AGGGCGCGAGCCCGGGCGGAGCGGCCGTC	31 (0.000586%)
CCTATGATGTTATCCCATGCTAATGTATCCAGAGCGTAGGCTTGCTTTGAGCACTCTAATTTCTTCAAAGT<br>AACAGCGCCGGAGGCACGACCCGGCCAAT	27 (0.000511%)
CCTATTTTCTGGTAAATTTTCATAATTTTTTGACACCTCTAGCTAGGTCATTTGACCTGATACAACATCGG<br>ATTTTCATGGTCTAGTTGGGGCTCCGTGG	6 (0.000114%)
CCTCACCATCCTTTGCTGATGCGGGACGGAAGCTGGTCTCCCGTGTGTTACCGCACGCGGTTGGCCTAAAT<br>CCGAGCCAAGGACGCCTGGAGCGTACCGA	57 (0.001078%)

CCTCCGCTTATTGATATGCTTAAACTCAGCGGGTAATCCCGCCTGACCTGGGGTCGCTATATGGACTTTGGGTCATCTACAGCTTCCGGACAAGAGCGAC	4 (0.000076%)	
CCTCGAAGAACTAATGGCAGCCCACGCAAGGCAAGCCCATTCTCCTCGACGATTCAGCAGTTTTTGTCCGAGAACTGCTGAGAAAACTCGGAAAAAGGCA	12 (0.000227%)	
CCTCGGCATCAGCGTGCTCCGGGCGTCGGCCTGTGGGCTCCCCATTCGACCCGTCTTGAAACACGGACCAAGGAGTCTGACATGTGTGCGAGTCAACGGG	8 (0.000151%)	
CCTCGTTAAGGGATTTAGATTGTACTCATTCCAATTACCAGACTCGAAAGAGCCCGGTATTGTTATTTATTGTCACTACCTCCCCGTGTCAGGATTGGGT	218 (0.004124%)	
CCTGTTGAGCTTGACTCTAGTCCGACTTTGTGAAATGACTTGAGAGGTGTAGGATAAGTGGGAGCTTCGGCGCAAGTGAAATACCACTACTTTTAACGTT	6 (0.000114%)	
CCTTAGATGTTCTGGGCCGCACGCGCGCTACACTGATGTATTCAACGAGTTCACACCTTGGCCGACAGGCCCGGGTAATCTTTGAAATTTCATCGTGATG	5 (0.000095%)	
CCTTGCCTACATTGTTCCATCGACCAGAGGCTGTTCACCTTGGAGACCTGATGCGGTTATGAGTACGACCGGGCGTGAGCGGCACTCGGTCCTCCGGATT	8 (0.000151%)	
CCTTGTTAGAAGACACAAAGCCAAAGACTCATATGGACTTTGGCTACACCATGAAAGCTTTGAGAAGCAAGAAGAAGGTTGGTTAGTGTTTTGGAGTCGA	43 (0.000813%)	
CCTTGTTAGTTTCTTTTCCTCCGCTTATTGATATGCTTAAACTCAGCGGGTAATCCCGCCTGACCTGGGGTCGCTATATGGACTTTGGGTCATCTACAGC	8 (0.000151%)	
CCTTTGCTGATGCGGGACGGAAGCTGGTCTCCCGTGTGTTACCGCACGCGGTTGGCCTAAATCCGAGCCAAGGACGCCTGGAGCGTACCGACATGCGGTG	5 (0.000095%)	
CGAAAGAGCCCGGTATTGTTATTTATTGTCACTACCTCCCCGTGTCAGGATTGGGTAATTTGCGCGCCTGCTGCCTTCCTTGGATGTGGTAGCCGTTTCT	20 (0.000378%)	
CGAAATCCGCTAAGGAGTGTGTAACAACTCACCTGCCGAATCAACTAGCCCCGAAAATGGATGGCGCTTAAGCGCGCGACCTATACCCGGCCGTCGGGGC	17 (0.000322%)	
CGAAATCCTATGATGTTATCCCATGCTAATGTATCCAGAGCGTAGGCTTGCTTTGAGCACTCTAATTTCTTCAAAGTAACAGCGCCGGAGGCACGACCCG	426 (0.008059%)	
CGAAGCACGCCCATCCAACCTAGGCGAGACAAGGGTTCACATTTCGTTCATCACCCTTGGCCGGCTATCGAACAGCCGGACTCCCATCAAAAGATGGTTG	25 (0.000473%)	
CGAAGCACGCCCATCCAACCTAGGCGAGACAAGGGTTCACATTTCGTTCATCACCCTTGGCCGGCTTTCGAACAGCCGGACTCCCATCAAAAGATGGTTG	18 (0.000341%)	
CGAATATGACTTGATGTCATGTGTATGATTGAGTATAAGA	542 (0.004101%)	
CGAATGCCAGCCGTTCGTTTGCATGTTCCTTGACACTTTTCGTGCCGGGGTTTTGTGATATCCGGAAGCAACGCGCACGACAAGACCGAGATAAAAGCTC	22 (0.000416%)	
CGAATGGCTCATTAAATCAGTTATAGTTTGTTTGATGGTAACTACTACTCGGATAACCGTAGTAATTCTAGAGCTAATACGTGCAACAAACCCCGACTTA	11 (0.000208%)	
CGACCAGAGGCTGTTCACCTTGGAGACCTGATGCGGTTATGAGTACGACCGGGCGTGAGCGGCACTCGGTCCTCCGGATTTTCAAGGGCCGCCGGGGGCG	19 (0.000359%)	
CGACCTATACCCGGCCGTCGGGGCAAGAGCCAGGCCTCGATGAGTAGGAGGGCGCGGCGGTCGCTGCAAAACCTAGGGCGCGAGCCCGGGCGGAGCGGCC	5 (0.000095%)	
CGACCTTTTATCTAATAAATGCGTCCCTTCCATAAGTCGGGGTTTGTTGCACGTATTAGCTCTAGAATTACTACGGTTATCCGAGTAGTAGTTACCATCA	23 (0.000435%)	
CGACGGGCGGTGTGTACAAAGGGCAGGGACGTAGTCAACGCGAGCTGATGACTCGCGCTTACTAGGAATTCCTCGTTGAAGACCAACAATTGCAATGATC	37 (0.000700%)	
CGACGTGGGTGGTTCGCCGCCCGCGACGTCGCGAGAAGTCCACTAAACCTTATCATTTAGAGGAAGGAGAAGTCGTAACAAGGTTTCCGTAGGTGAACCT	98 (0.001854%)	
CGACTCCAAAACACTAACCAACCTTCTTCTTGCTTCTCAAAGCTTTCATGGTGTAGCCAAAGTCCATATGAGTCTTTGGCTTTGTGTCTTCTAACAAGGA	55 (0.001041%)	
CGACTTCCCTTGCCTACATTGTTCCATCGACCAGAGGCTGTTCACCTTGGAGACCTGATGCGGTTATGAGTACGACCGGGCGTGAGCGGCACTCGGTCCT	34 (0.000643%)	
CGACTTTGTGAAATGACTTGAGAGGTGTAGGATAAGTGGGAGCTTCGGCGCAAGTGAAATACCACTACTTTTAACGTTATTTTACTTACTCCGTGAATCG	11 (0.000208%)	
CGAGACAAGGGTTCACATTTCGTTCATCACCCTTGGCCGGCTATCGAACAGCCGGACTCCCATCAAAAGATGGTTGCCAAGAACATCTTCGTTACGGTTT	12 (0.000227%)	
CGAGACAAGGGTTCACATTTCGTTCATCACCCTTGGCCGGCTTTCGAACAGCCGGACTCCCATCAAAAGATGGTTGCCAAGAACATCTTCGTTACGGTTT	4 (0.000076%)	
CGAGGTGTGAGTGTCGCCCATGGGCATCGACACCTTGCGGCTAGGAACTGGAACGAGACGGGTAGCAAAGATTTCGAGTAGCACTTCATACTACCGTGGG	15 (0.000284%)	
CGAGGTGTGAGTGTCGCCCATGGGCATCGACACCTTGCGGCTAGGAACTGGAACGAGACGGGTGGCAAAGATTTCGAGTAGCACTTCATACTACCGTGGG	21 (0.000397%)	
CGAGTGTGAGCGAGGTGTGAGTGTCGCCCATGGGCATCGACACCTTGCGGCTAGGAACTGGAACGAGACGGGTAGCAAAGATTTCGAGTAGCACTTCATA	33 (0.000624%)	
CGAGTGTGAGCGAGGTGTGAGTGTCGCCCATGGGCATCGACACCTTGCGGCTAGGAACTGGAACGAGACGGGTGGCAAAGATTTCGAGTAGCACTTCATA	35 (0.000662%)	
CGAGTTATCATGAATCATCAGAGCAACGGGCAGAGCCCGCGTCGACCTTTTATCTAATAAATGCGTCCCTTCCATAAGTCGGGGTTTGTTGCACGTATTA	138 (0.002611%)	
CGATCACGGCAATTCCCCGCCACATCCTCTCAAACGCAATGGAAAGAGAGAAAGGACGAGGTCTTGACCGTCATCTTTTGCCCGAAGGACGGATGAGCTT	3 (0.000057%)	
CGATCCGCCTAGGCTGTCCCGAGTGTGAGCGAGGTGTGAGTGTCGCCCATGGGCATCGACACCTTGCGGCTAGGAACTGGAACGAGACGGGTGGCAAAGA	6 (0.000114%)	
CGATCCGGTTAAAATTCCGGAACCGGGACGTGGCGGTTGACGGCAACGTTAGGGAGTCCGGAGACGTCGGCGGGGGCCTCGGGAAGAGTTATCTTTTCTG	3 (0.000057%)	
CGATCCGTCGAGTTATCATGAATCATCAGAGCAACGGGCAGAGCCCGCGTCGACCTTTTATCTAATAAATGCGTCCCTTCCATAAGTCGGGGTTTGTTGC	151 (0.002857%)	
CGATCCTCGTTAAGGGATTTAGATTGTACTCATTCCAATTACCAGACTCGAAAGAGCCCGGTATTGTTATTTATTGTCACTACCTCCCCGTGTCAGGATT	6 (0.000114%)	
CGATCGAAATCCTATGATGTTATCCCATGCTAATGTATCCAGAGCGTAGGCTTGCTTTGAGCACTCTAATTTCTTCAAAGTAACAGCGCCGGAGGCACGA	8 (0.000151%)	
CGATCGGGTTGCGGTTTAAGTTGTTATACTCAATCATACA	76 (0.000575%)	
CGCAACAAGATCTTAAAGGCGTAAGAATTGTATCCTTGTTAAAAGACACAAAGCCAAAGACTCATATGGACTTTGGCTACACCATGAAAGCTTTGAGAAG	32 (0.000605%)	
CGCAATGGAAAGAGAGAAAGGACGAGGTCTTGACCGTCATCTTTTGCCCGAAGGACGGATGAGCTTTGGCGGGACTGAATCACTTCGAGTCACCGTCGAC	3 (0.000057%)	
CGCATGTCGGTACGCTCCAGGCGTCCTTGGCTCGGATTTAGGCCAACCGCGTGCGGTAACACACGGGAGACCAGCTTCCGTCCCGCATCAGCAAAGGATG	8 (0.000151%)	

CGCCACATCCTCTCAAACGCAATGGAAAGAGAGAAAGGACGAGGTCTTGACCGTCATCTTTTGCCCGAAGG ACGGATGAGCTTTGGCGGGACTGAATCAC	7 (0.000132%)	
CGCCCATCCAACCTAGGCGAGACAAGGGTTCACATTTCGTTCATCACCCTTGGCCGGCTATCGAACAGCCG GACTCCCATCAAAAGATGGTTGCCAAGAA	73 (0.001381%)	
CGCCCATCCAACCTAGGCGAGACAAGGGTTCACATTTCGTTCATCACCCTTGGCCGGCTTTCGAACAGCCG GACTCCCATCAAAAGATGGTTGCCAAGAA	94 (0.001778%)	
CGCCCATGGGCATCGACACCTTGCGGCTAGGAACTGGAACGAGACGGGTGGCAAAGATTTCGAGTAGCACT TCATACTACCGTGGGTTTTTTAAACCTTC	32 (0.000605%)	
CGCCGCCCGCGACGTCGCGAGAAGTCCACTAAACCTTATCATTTAGAGGAAGGAGAAGTCGTAACAAGGTT TCCGTAGGTGAACCTGCGGAAGGATCGAT	7 (0.000132%)	
CGCCGTTACTAAGGGAATCCTTGTTAGTTTCTTTTCCTCCGCTTATTGATATGCTTAAACTCAGCGGGTAA TCCCGCCTGACCTGGGGTCGCTATATGGA	5 (0.000095%)	
CGCCTAACGGCGTGCCTCGGCATCAGCGTGCTCCGGGCGTCGGCCTGTGGGCTCCCCATTCGACCCGTCTT GAAACACGGACCAAGGAGTCTGACATGTG	42 (0.000795%)	
CGCCTAGGCTGTCCCGAGTGTGAGCGAGGTGTGAGTGTCGCCCATGGGCATCGACACCTTGCGGCTAGGAA CTGGAACGAGACGGGTAGCAAAGATTTCG	115 (0.002176%)	
CGCCTAGGCTGTCCCGAGTGTGAGCGAGGTGTGAGTGTCGCCCATGGGCATCGACACCTTGCGGCTAGGAA CTGGAACGAGACGGGTGGCAAAGATTTCG	207 (0.003916%)	
CGCCTCGAAGAACTAATGGCAGCCCACGCAAGGCAAGCCCATTCTCCTCGACGATTCAGCAGTTTTTGTCC GAGAACTGCTGAGAAAACTCGGAAAAAGG	3 (0.000057%)	
CGCGACCTATACCCGGCCGTCGGGGCAAGAGCCAGGCCTCGATGAGTAGGAGGGCGCGGCGGTCGCTGCAA AACCTAGGGCGCGAGCCCGGGCGGAGCGG	14 (0.000265%)	
CGCGCCTAACGGCGTGCCTCGGCATCAGCGTGCTCCGGGCGTCGGCCTGTGGGCTCCCCATTCGACCCGTC TTGAAACACGGACCAAGGAGTCTGACATG	43 (0.000813%)	
CGCGGTAATTCCAGCTCCAATAGCGTATATTTAAGTTGTTGCAGTTAAAAAGCTCGTAGTTGAACCTTGGG ATGGGTCGGCCGGTCCGCCTTTGGTGTGC	12 (0.000227%)	
CGCGTCGACCTTTTATCTAATAAATGCGTCCCTTCCATAAGTCGGGGTTTGTTGCACGTATTAGCTCTAGA ATTACTACGGTTATCCGAGTAGTAGTTAC	5 (0.000095%)	
CGCTAAGGAGTGTGTAACAACTCACCTGCCGAATCAACTAGCCCCGAAAATGGATGGCGCTTAAGCGCGCG ACCTATACCCGGCCGTCGGGGCAAGAGCC	8 (0.000151%)	
CGCTTATTGATATGCTTAAACTCAGCGGGTAATCCCGCCTGACCTGGGGTCGCTATATGGACTTTGGGTCA TCTACAGCTTCCGGACAAGAGCGACCGAT	8 (0.000151%)	
CGCTTTCACGGTTCGTATTCGTACTGAAAATCAGAATCAAACGAGCTTTTACCCTTTTGTTCCACACGAGA TTTCTGTTCTCGTTGAGCTCATCTTAGGA	9 (0.000170%)	
CGGAAGAGCACACGTCTGAACTCCAGTCACGAGATTCCAT	17 (0.000129%)	
CGGACCGGATTGCTCCGTTCCGCATCCGACCAGGACGCATCGCCGGCCCCCATCCGCTTCCCTCCCGACAA TTTCAAGCACTCTTTGACTCTCTTTTCAA	3 (0.000057%)	
CGGAGAATTAGGGTTCGATTCCGGAGAGGGAGCCTGAGAAACGGCTACCACATCCAAGGAAGGCAGCAGGC GCGCAAATTACCCAATCCTGACACGGGGA	18 (0.000341%)	
CGGATCTTAAAGGCGTAAGAATTGTATCCTTGTTAGAAGACACAAAGCCAAAGACTCATATGGACTTTGGC TACACCATGAAAGCTTTGAGAAGCAAGAA	11 (0.000208%)	
CGGATTGCTCCGTTCCGCATCCGACCAGGACGCATCGCCGGCCCCCATCCGCTTCCCTCCCGACAATTTCA AGCACTCTTTGACTCTCTTTTCAAAGTCC	25 (0.000473%)	
CGGCAATTCCCCGCCACATCCTCTCAAACGCAATGGAAAGAGAGAAAGGACGAGGTCTTGACCGTCATCTT TTGCCCGAAGGACGGATGAGCTTTGGCGG	172 (0.003254%)	
CGGCACATCTGTTAAAAGATAACGCAGGTGTCCTAAGATGAGCTCAACGAGAACAGAAATCTCGTGTGGAA CAAAAGGGTAAAAGCTCGTTTGATTCTGA	5 (0.000095%)	
CGGCATCAGCGTGCTCCGGGCGTCGGCCTGTGGGCTCCCCATTCGACCCGTCTTGAAACACGGACCAAGGA GTCTGACATGTGTGCGAGTCAACGGGTGA	15 (0.000284%)	
CGGCCGTCGGGGCAAGAGCCAGGCCTCGATGAGTAGGAGGGCGCGGCGGTCGCTGCAAAACCTAGGGCGCG AGCCCGGGCGGAGCGGCCGTCGGTGCAGA	46 (0.000870%)	
CGGCGGACCGGATTGCTCCGTTCCGCATCCGACCAGGACGCATCGCCGGCCCCCATCCGCTTCCCTCCCGA CAATTTCAAGCACTCTTTGACTCTCTTTT	10 (0.000189%)	
CGGCGTGCCTCGGCATCAGCGTGCTCCGGGCGTCGGCCTGTGGGCTCCCCATTCGACCCGTCTTGAAACAC GGACCAAGGAGTCTGACATGTGTGCGAGT	9 (0.000170%)	
CGGGCAGAGCCCGCGTCGACCTTTTATCTAATAAATGCGTCCCTTCCATAAGTCGGGGTTTGTTGCACGTA TTAGCTCTAGAATTACTACGGTTATCCGA	66 (0.001249%)	
CGGGCGGTGTGTACAAAGGGCAGGGACGTAGTCAACGCGAGCTGATGACTCGCGCTTACTAGGAATTCCTC GTTGAAGACCAACAATTGCAATGATCGAT	32 (0.000605%)	
CGGGCGTCGGCCTGTGGGCTCCCCATTCGACCCGTCTTGAAACACGGACCAAGGAGTCTGACATGTGTGCG AGTCAACGGGTGAGTAAACCCGTAAGGCG	54 (0.001022%)	
CGGGGAGGCGAATGCCAGCCGTTCGTTTGCATGTTCCTTGACACTTTTCGTGCCGGGGTTTTGTGATATCC GGAAGCAACGCGCACGACAAGACCGAGAT	12 (0.000227%)	
CGGGGGCATTCGTATTTCATAGTCAGAGGTGAAATTCTTGGATTTATGAAAGACGAACAACTGCGAAAGCA TTTGCCAAGGATGTTTTCATTAATCAAGA	32 (0.000605%)	
CGGGTGACGGAGAATTAGGGTTCGATTCCGGAGAGGGAGCCTGAGAAACGGCTACCACATCCAAGGAAGGC AGCAGGCGCGCAAATTACCCAATCCTGAC	7 (0.000132%)	
CGGGTTGCGGTTTAAGTTGTTATACTCAATCATACACATG	488 (0.003693%)	
CGGGTTTACTCACCCGTTGACTCGCACACATGTCAGACTCCTTGGTCCGTGTTTCAAGACGGGTCGAATGG GGAGCCCACAGGCCGACGCCCGGAGCACG	13 (0.000246%)	
CGGTAATTCCAGCTCCAATAGCGTATATTTAAGTTGTTGCAGTTAAAAAGCTCGTAGTTGAACCTTGGGAT GGGTCGGCCGGTCCGCCTTTGGTGTGCAT	14 (0.000265%)	
CGGTACGCTCCAGGCGTCCTTGGCTCGGATTTAGGCCAACCGCGTGCGGTAACACACGGGAGACCAGCTTC CGTCCCGCATCAGCAAAGGATGGTGAGGG	8 (0.000151%)	
CGGTAGGAGCGACGGGCGGTGTGTACAAAGGGCAGGGACGTAGTCAACGCGAGCTGATGACTCGCGCTTAC TAGGAATTCCTCGTTGAAGACCAACAATT	51 (0.000965%)	
CGGTTTAAGTTCTTATACTCAATCATACACATGACATCAA	519 (0.003927%)	
CGGTTTAAGTTGTTATACTCAATCATACACATGACAACAAGTCATATTCGACTCCAAAACACTAACCAACC TTCTTCTTGCTTCTCAAAGCTTTCATGGT	1007 (0.019051%)	
CGTAACTTCGGGAAAAGGATTGGCTCTGAGGGCTGGGCTCGGGGGTCCCAGTTCCGAACCCGTCGGCTGTC AGCGGACTGCTCGAGCTGCTTCCGCGGCG	6 (0.000114%)	
CGTAAGAATTGTATCCTTGTTAGAAGACACAAAGCCAAAGACTCATATGGACTTTGGCTACACCATGAAAG CTTTGAGAAGCAAGAAGGTTGGTTAG	62 (0.001173%)	

CGTACTGAAAATCAGAATCAAACGAGCTTTTACCCTTTTGTTCCACACGAGATTTCTGTTCTCGTTGAGCTCATCTTAGGACACCTGCGTTATCTTTTAA	6 (0.000114%)	
CGTAGGCTTGCTTTGAGCACTCTAATTTCTTCAAAGTAACAGCGCCGGAGGCACGACCCGGCCAATTAAGACCAGGAGCGTATCGCCGACCGAAGGGACA	19 (0.000359%)	
CGTATTCGTACTGAAAATCAGAATCAAACGAGCTTTTACCCTTTTGTTCCACACGAGATTTCTGTTCTCGTTGAGCTCATCTTAGGACACCTGCGTTATC	24 (0.000454%)	
CGTATTTCATAGTCAGAGGTGAAATTCTTGGATTTATGAAAGACGAACAACTGCGAAAGCATTTGCCAAGGATGTTTTCATTAATCAAGAACGAAAGTTG	15 (0.000284%)	
CGTCCCTCACCATCCTTTGCTGATGCGGGACGGAAGCTGGTCTCCCGTGTGTTACCGCACGCGGTTGGCCTAAATCCGAGCCAAGGACGCCTGGAGCGTA	12 (0.000227%)	
CGTCGACCTTTTATCTAATAAATGCGTCCCTTCCATAAGTCGGGGTTTGTTGCACGTATTAGCTCTAGAATTACTACGGTTATCCGAGTAGTAGTTACCA	5 (0.000095%)	
CGTCGAGTTATCATGAATCATCAGAGCAACGGGCAGAGCCCGCGTCGACCTTTTATCTAATAAATGCGTCCCTTCCATAAGTCGGGGTTTGTTGCACGTA	110 (0.002081%)	
CGTCGGCCTGTGGGCTCCCCATTCGACCCGTCTTGAAACACGGACCAAGGAGTCTGACATGTGTGCGAGTCAACGGGTGAGTAAACCCGTAAGGCGCAAG	6 (0.000114%)	
CGTCTCCGGGGAGGCGAATGCCAGCCGTTCGTTTGCATGTTCCTTGACACTTTTCGTGCCGGGGTTTTGTGATATCCGGAAGCAACGCGCACGACAAGAC	3 (0.000057%)	
CGTGCCTCGGCATCAGCGTGCTCCGGGCGTCGGCCTGTGGGCTCCCCATTCGACCCGTCTTGAAACACGGACCAAGGAGTCTGACATGTGTGCGAGTCAA	32 (0.000605%)	
CGTGCTCCGGGCGTCGGCCTGTGGGCTCCCCATTCGACCCGTCTTGAAACACGGACCAAGGAGTCTGACATGTGTGCGAGTCAACGGGTGAGTAAACCCG	7 (0.000132%)	
CGTGGGTGGTTCGCCGCCCGCGACGTCGCGAGAAGTCCACTAAACCTTATCATTTAGAGGAAGGAGAAGTCGTAACAAGGTTTCCGTAGGTGAACCTGCG	32 (0.000605%)	
CGTTAAGGGATTTAGATTGTACTCATTCCAATTACCAGACTCGAAAGAGCCCGGTATTGTTATTTATTGTCACTACCTCCCCGTGTCAGGATTGGGTAAT	64 (0.001211%)	
CTAAATCCTATTTTCTGGTAAATTTTCATAATTTTTTGACACCTCTAGCTAGGTCATTTGACCTGATACAACATCGGATTTTCATGGTCTAGTTGGGGCT	22 (0.000416%)	
CTAACGCCTCGAAGAACTAATGGCAGCCCACGCAAGGCAAGCCCATTCTCCTCGACGATTCAGCAGTTTTTGTCCGAGAACTGCTGAGAAAACTCGGAAA	32 (0.000605%)	
CTAACGGCGTGCCTCGGCATCAGCGTGCTCCGGGCGTCGGCCTGTGGGCTCCCCATTCGACCCGTCTTGAAACACGGACCAAGGAGTCTGACATGTGTGC	100 (0.001892%)	
CTAAGTAGTGTTTCCTTGTTAGAAGACACAAAGCCAAAGACTCATATGGACTTTGGCTACACCATGAAAGCTTTGAGAAGCAAGAAGAAGGTTGGTTAGT	83 (0.001570%)	
CTAATGTATCCAGAGCGTAGGCTTGCTTTGAGCACTCTAATTTCTTCAAAGTAACAGCGCCGGAGGCACGACCCGGCCAATTAAGACCAGGAGCGTATCG	49 (0.000927%)	
CTACATTGTTCCATCGACCAGAGGCTGTTCACCTTGGAGACCTGATGCGGTTATGAGTACGACCGGGCGTGAGCGGCACTCGGTCCTCCGGATTTTCAAG	50 (0.000946%)	
CTAGGCGAGACAAGGGTTCACATTTCGTTCATCACCCTTGGCCGGCTATCGAACAGCCGGACTCCCATCAAAAGATGGTTGCCAAGAACATCTTCGTTAC	27 (0.000511%)	
CTAGGCGAGACAAGGGTTCACATTTCGTTCATCACCCTTGGCCGGCTTTCGAACAGCCGGACTCCCATCAAAAGATGGTTGCCAAGAACATCTTCGTTAC	34 (0.000643%)	
CTAGGCTGTCCCGAGTGTGAGCGAGGTGTGAGTGTCGCCCATGGGCATCGACACCTTGCGGCTAGGAACTGGAACGAGACGGGTAGCAAAGATTTCGAGT	287 (0.005430%)	
CTAGGCTGTCCCGAGTGTGAGCGAGGTGTGAGTGTCGCCCATGGGCATCGACACCTTGCGGCTAGGAACTGGAACGAGACGGGTGGCAAAGATTTCGAGT	447 (0.008456%)	
CTAGTCCGACTTTGTGAAATGACTTGAGAGGTGTAGGATAAGTGGGAGCTTCGGCGCAAGTGAAATACCACTACTTTTAACGTTATTTTACTTACTCCGT	12 (0.000227%)	
CTATACCCGGCCGTCGGGGCAAGAGCCAGGCCTCGATGAGTAGGAGGGCGCGGCGGTCGCTGCAAAACCTAGGGCGCGAGCCCGGGCGGAGCGGCCGTCG	71 (0.001343%)	
CTATGATGTTATCCCATGCTAATGTATCCAGAGCGTAGGCTTGCTTTGAGCACTCTAATTTCTTCAAAGTAACAGCGCCGGAGGCACGACCCGGCCAATT	227 (0.004294%)	
CTATTTTCTGGTAAATTTTCATAATTTTTTGACACCTCTAGCTAGGTCATTTGACCTGATACAACATCGGATTTTCATGGTCTAGTTGGGGCTCCGTGGG	23 (0.000435%)	
CTCAAACGCAATGGAAAGAGAGAAAGGACGAGGTCTTGACCGTCATCTTTTGCCCGAAGGACGGATGAGCTTTGGCGGGACTGAATCACTTCGAGTCACC	17 (0.000322%)	
CTCAAAGATTAAGCCATGCATGTGTAAGTATGAACGAATTCAGACTGTGAAACTGCGAATGGCTCATTAAATCAGTTATAGTTTGTTTGATGGTAACTAC	18 (0.000341%)	
CTCAAAGCTTTCATGGTGTAGCCAAAGTCCATATGAGTCTTTGGCTTTGTGTCTTCTAACAAGGAAACACTACTTAGGCTTATAAGATGCGGTTGCGGTT	7 (0.000132%)	
CTCAATCATACACATGACAACAAGTCATATTCGACTCCAAAACACTAACCAACCTTCTTCTTGCTTCTCAAAGCTTTCATGGTGTAGCCAAAGTCCATAT	19 (0.000359%)	
CTCAATCATACACATGACATCAAGTCATATTCGACTCCAAAACACTAACCAACCTTCTTCTTGCTTCTCAAAGCTTTCATGGTGTAGCCAAAGTCCATAT	100 (0.001892%)	
CTCACCATCCTTTGCTGATGCGGGACGGAAGCTGGTCTCCCGTGTGTTACCGCACGCGGTTGGCCTAAATCCGAGCCAAGGACGCCTGGAGCGTACCGAC	21 (0.000397%)	
CTCACCCGTTGACTCGCACACATGTCAGACTCCTTGGTCCGTGTTTCAAGACGGGTCGAATGGGGAGCCCACAGGCCGACGCCCGGAGCACGCTGATGCC	17 (0.000322%)	
CTCATATGGACTTTGGCTACACCATGAAAGCTTTGAGAAGCAAGAAGAAGGTTGGTTAGTGTTTTGGAGTCGAATATGACTTGATGTCATGTGTATGATT	88 (0.001665%)	
CTCATTAAATCAGTTATAGTTTGTTTGATGGTAACTACTACTCGGATAACCGTAGTAATTCTAGAGCTAATACGTGCAACAAACCCCGACTTATGGAAGG	10 (0.000189%)	
CTCATTCCAATTACCAGACTCGAAAGAGCCCGGTATTGTTATTTATTGTCACTACCTCCCCGTGTCAGGATTGGGTAATTTGCGCGCCTGCTGCCTTCCT	15 (0.000284%)	
CTCCGCTTATTGATATGCTTAAACTCAGCGGGTAATCCCGCCTGACCTGGGGTCGCTATATGGACTTTGGGTCATCTACAGCTTCCGGACAAGAGCGACC	5 (0.000095%)	
CTCCGGGCGTCGGCCTGTGGGCTCCCCATTCGACCCGTCTTGAAACACGGACCAAGGAGTCTGACATGTGTGCGAGTCAACGGGTGAGTAAACCCGTAAG	21 (0.000397%)	
CTCCGGGGAGGCGAATGCCAGCCGTTCGTTTGCATGTTCCTTGACACTTTTCGTGCCGGGGTTTTGTGATATCCGGAAGCAACGCGCACGACAAGACCGA	41 (0.000776%)	
CTCCGTTCCGCATCCGACCAGGACGCATCGCCGGCCCCCATCCGCTTCCCTCCCGACAATTTCAAGCACTCTTTGACTCTCTTTTCAAAGTCCTTTTCAT	5 (0.000095%)	
CTCGAAAGAGCCCGGTATTGTTATTTATTGTCACTACCTCCCCGTGTCAGGATTGGGTAATTTGCGCGCCTGCTGCCTTCCTTGGATGTGGTAGCCGTTT	32 (0.000605%)	
CTCGAAGAACTAATGGCAGCCCACGCAAGGCAAGCCCATTCTCCTCGACGATTCAGCAGTTTTTGTCCGAGAACTGCTGAGAAAACTCGGAAAAAGGCAA	11 (0.000208%)	

CTCGCCGTTACTAAGGGAATCCTTGTTAGTTTCTTTTCCTCCGCTTATTGATATGCTTAAACTCAGCGGGT<br>AATCCCGCCTGACCTGGGGTCGCTATATG	44 (0.000832%)	
CTCGGCATCAGCGTGCTCCGGGCGTCGGCCTGTGGGCTCCCCATTCGACCCGTCTTGAAACACGGACCAAG<br>GAGTCTGACATGTGTGCGAGTCAACGGGT	21 (0.000397%)	
CTCGTTAAGGGATTTAGATTGTACTCATTCCAATTACCAGACTCGAAAGAGCCCGGTATTGTTATTTATTG<br>TCACTACCTCCCCGTGTCAGGATTGGGTA	127 (0.002403%)	
CTCTAGTCCGACTTTGTGAAATGACTTGAGAGGTGTAGGATAAGTGGGAGCTTCGGCGCAAGTGAAATACC<br>ACTACTTTTAACGTTATTTTACTTACTCC	14 (0.000265%)	
CTCTCAAACGCAATGGAAAGAGAGAAAGGACGAGGTCTTGACCGTCATCTTTTGCCCGAAGGACGGATGAG<br>CTTTGGCGGGACTGAATCACTTCGAGTCA	4 (0.000076%)	
CTCTCTCTCTCTCTCTCTCTCTCTCTCTCTCTCTCTCT	49 (0.000371%)	
CTGAAAATCAGAATCAAACGAGCTTTTACCCTTTTGTTCCACACGAGATTTCTGTTCTCGTTGAGCTCATC<br>TTAGGACACCTGCGTTATCTTTTAACAGA	23 (0.000435%)	
CTGATGCGGGACGGAAGCTGGTCTCCCGTGTGTTACCGCACGCGGTTGGCCTAAATCCGAGCCAAGGACGC<br>CTGGAGCGTACCGACATGCGGTGGTGAAC	25 (0.000473%)	
CTGCAGCACGCGCCTAACGGCGTGCCTCGGCATCAGCGTGCTCCGGGCGTCGGCCTGTGGGCTCCCCATTC<br>GACCCGTCTTGAAACACGGACCAAGGAGT	2141 (0.040504%)	
CTGCCAGTAGTCATATGCTTGTCTCAAAGATTAAGCCATGCATGTGTAAGTATGAACGAATTCAGACTGTG<br>AAACTGCGAATGGCTCATTAAATCAGTTA	1952 (0.036928%)	
CTGCGAATGGCTCATTAAATCAGTTATAGTTTGTTTGATGGTAACTACTACTCGGATAACCGTAGTAATTC<br>TAGAGCTAATACGTGCAACAAACCCCGAC	29 (0.000549%)	
CTGGGCCGCACGCGCGCTACACTGATGTATTCAACGAGTTCACACCTTGGCCGACAGGCCCGGGTAATCTT<br>TGAAATTTCATCGTGATGGGGATAGATCG	66 (0.001249%)	
CTGGGGCGGCACATCTGTTAAAAGATAACGCAGGTGTCCTAAGATGAGCTCAACGAGAACAGAAATCTCGT<br>GTGGAACAAAAGGGTAAAAGCTCGTTTGA	11 (0.000208%)	
CTGGTGCCAGCAGCCGCGGTAATTCCAGCTCCAATAGCGTATATTTAAGTTGTTGCAGTTAAAAAGCTCGT<br>AGTTGAACCTTGGGATGGGTCGGCCGGTC	69 (0.001305%)	
CTGTCCCGAGTGTGAGCGAGGTGTGAGTGTCGCCCATGGGCATCGACACCTTGCGGCTAGGAACTGGAACG<br>AGACGGGTAGCAAAGATTTCGAGTAGCAC	14 (0.000265%)	
CTGTCCCGAGTGTGAGCGAGGTGTGAGTGTCGCCCATGGGCATCGACACCTTGCGGCTAGGAACTGGAACG<br>AGACGGGTGGCAAAGATTTCGAGTAGCAC	22 (0.000416%)	
CTGTGATGCCCTTAGATGTTCTGGGCCGCACGCGCGCTACACTGATGTATTCAACGAGTTCACACCTTGGC<br>CGACAGGCCCGGGTAATCTTTGAAATTTC	92 (0.001740%)	
CTGTTAAAAGATAACGCAGGTGTCCTAAGATGAGCTCAACGAGAACAGAAATCTCGTGTGGAACAAAAGGG<br>TAAAAGCTCGTTTGATTCTGATTTTCAGT	5 (0.000095%)	
CTGTTGAGCTTGACTCTAGTCCGACTTTGTGAAATGACTTGAGAGGTGTAGGATAAGTGGGAGCTTCGGCG<br>CAAGTGAAATACCACTACTTTTAACGTTA	13 (0.000246%)	
CTTAAAAGCCTAAGTAGTGTTTCCTTGTTAGAAGACACAAAGCCAAAGACTCATATGGACTTTGGCTACAC<br>CATGAAAGCTTTGAGAAGCAAGAAGAAGG	210 (0.003973%)	
CTTAAAGGCGTAAGAATTGTATCCTTGTTAAAAGACACAAAGCCAAAGACTCATATGGACTTTGGCTACAC<br>CATGAAAGCTTTGAGAAGCAAGAAGAAGG	238 (0.004503%)	
CTTAAAGGCGTAAGAATTGTATCCTTGTTAGAAGACACAAAGCCAAAGACTCATATGGACTTTGGCTACAC<br>CATGAAAGCTTTGAGAAGCAAGAAGAAGG	209 (0.003954%)	
CTTAAGCGCGCGACCTATACCCGGCCGTCGGGGCAAGAGCCAGGCCTCGATGAGTAGGAGGGCGCGGCGGT<br>CGCTGCAAAACCTAGGGCGCGAGCCCGGG	52 (0.000984%)	
CTTAGATGTTCTGGGCCGCACGCGCGCTACACTGATGTATTCAACGAGTTCACACCTTGGCCGACAGGCCC<br>GGGTAATCTTTGAAATTTCATCGTGATGG	110 (0.002081%)	
CTTATACTCAATCATACACATGACATCAAGTCATATTCGACTCCAAAACACTAACCAACCTTCTTCTTGCT<br>TCTCAAAGCTTTCATGGTGTAGCCAAAGT	146 (0.002762%)	
CTTATTGATATGCTTAAACTCAGCGGGTAATCCCGCCTGACCTGGGGTCGCTATATGGACTTTGGGTCATC<br>TACAGCTTCCGGACAAGAGCGACCGATAA	23 (0.000435%)	
CTTCAACGAGGAATTCCTAGTAAGCGCGAGTCATCAGCTCGCGTTGACTACGTCCCTGCCCTTTGTACACA<br>CCGCCCGTCGCTCCTACCGATTGAATGAT	58 (0.001097%)	
CTTCCCTTGCCTACATTGTTCCATCGACCAGAGGCTGTTCACCTTGGAGACCTGATGCGGTTATGAGTACG<br>ACCGGGCGTGAGCGGCACTCGGTCCTCCG	61 (0.001154%)	
CTTCTCAAAGCTTTCATGGTGTAGCCAAAGTCCATATGAGTCTTTGGCTTTGTGTCTTCTAACAAGGAAAC<br>ACTACTTAGGCTTATAAGATGCGGTTGCG	10 (0.000189%)	
CTTGACTCTAGTCCGACTTTGTGAAATGACTTGAGAGGTGTAGGATAAGTGGGAGCTTCGGCGCAAGTGAA<br>ATACCACTACTTTTAACGTTATTTTACTT	67 (0.001268%)	
CTTGATGTCATGTGTATGATTGAGTATAAGAACTTAAACC	310 (0.002346%)	
CTTGCCTACATTGTTCCATCGACCAGAGGCTGTTCACCTTGGAGACCTGATGCGGTTATGAGTACGACCGG<br>GCGTGAGCGGCACTCGGTCCTCCGGATTT	49 (0.000927%)	
CTTGCTTCTCAAAGCTTTCATGGTGTAGCCAAAGTCCATATGAGTCTTTGGCTTTGTGTCTTCTAACAAGG<br>AAACACTACTTAGGCTTATAAGATGCGGT	66 (0.001249%)	
CTTGCTTTGAGCACTCTAATTTCTTCAAAGTAACAGCGCCGGAGGCACGACCCGGCCAATTAAGACCAGGA<br>GCGTATCGCCGACCGAAGGGACAAGCCGA	69 (0.001305%)	
CTTGTCTCAAAGATTAAGCCATGCATGTGTAAGTATGAACGAATTCAGACTGTGAAACTGCGAATGGCTCA<br>TTAAATCAGTTATAGTTTGTTTGATGGTA	53 (0.001003%)	
CTTGTTAGAAGACACAAAGCCAAAGACTCATATGGACTTTGGCTACACCATGAAAGCTTTGAGAAGCAAGA<br>AGAAGGTTGGTTAGTGTTTTGGAGTCGAA	120 (0.002270%)	
CTTGTTAGTTTCTTTTCCTCCGCTTATTGATATGCTTAAACTCAGCGGGTAATCCCGCCTGACCTGGGGTC<br>GCTATATGGACTTTGGGTCATCTACAGCT	28 (0.000530%)	
CTTTCACGGTTCGTATTCGTACTGAAAATCAGAATCAAACGAGCTTTTACCCTTTTGTTCCACACGAGATT<br>TCTGTTCTCGTTGAGCTCATCTTAGGACA	55 (0.001041%)	
CTTTCCAACGAAGCACGCCCATCCAACCTAGGCGAGACAAGGGTTCACATTTCGTTCATCACCCTTGGCCG<br>GCTATCGAACAGCCGGACTCCCATCAAAA	15 (0.000284%)	
CTTTGCTGATGCGGGACGGAAGCTGGTCTCCCGTGTGTTACCGCACGCGGTTGGCCTAAATCCGAGCCAAG<br>GACGCCTGGAGCGTACCGACATGCGGTGG	52 (0.000984%)	
CTTTTATCTAATAAATGCGTCCCTTCCATAAGTCGGGGTTTGTTGCACGTATTAGCTCTAGAATTACTACG<br>GTTATCCGAGTAGTAGTTACCATCAAACA	83 (0.001570%)	
CTTTTCCTCCGCTTATTGATATGCTTAAACTCAGCGGGTAATCCCGCCTGACCTGGGGTCGCTATATGGAC<br>TTTGGGTCATCTACAGCTTCCGGACAAGA	33 (0.000624%)	
GAAAATCAGAATCAAACGAGCTTTTACCCTTTTGTTCCACACGAGATTTCTGTTCTCGTTGAGCTCATCTT<br>AGGACACCTGCGTTATCTTTTAACAGATG	8 (0.000151%)	

GAAACTGCGAATGGCTCATTAAATCAGTTATAGTTTGTTTGATGGTAACTACTACTCGGATAACCGTAGTAATTCTAGAGCTAATACGTGCAACAAACCC	217 (0.004105%)
GAAAGAAGACCCTGTTGAGCTTGACTCTAGTCCGACTTTGTGAAATGACTTGAGAGGTGTAGGATAAGTGGGAGCTTCGGCGCAAGTGAAATACCACTAC	41 (0.000776%)
GAAAGAGAGAAAGGACGAGGTCTTGACCGTCATCTTTTGCCCGAAGGACGGATGAGCTTTGGCGGGACTGAATCACTTCGAGTCACCGTCGACAACTTTT	3 (0.000057%)
GAAAGAGCCCGGTATTGTTATTTATTGTCACTACCTCCCCGTGTCAGGATTGGGTAATTTGCGCGCCTGCTGCCTTCCTTGGATGTGGTAGCCGTTTCTC	27 (0.000511%)
GAAATCCGCTAAGGAGTGTGTAACAACTCACCTGCCGAATCAACTAGCCCCGAAAATGGATGGCGCTTAAGCGCGCGACCTATACCCGGCCGTCGGGGCA	27 (0.000511%)
GAAATCCTATGATGTTATCCCATGCTAATGTATCCAGAGCGTAGGCTTGCTTTGAGCACTCTAATTTCTTCAAAGTAACAGCGCCGGAGGCACGACCCGG	179 (0.003386%)
GAAGACACAAAGCCAAAGACTCATATGGACTTTGGCTACACCATGAAAGCTTTGAGAAGCAAGAAGAAGGTTGGTTAGTGTTTTGGAGTCGAATATGACT	42 (0.000795%)
GAAGACCCTGTTGAGCTTGACTCTAGTCCGACTTTGTGAAATGACTTGAGAGGTGTAGGATAAGTGGGAGCTTCGGCGCAAGTGAAATACCACTACTTTT	13 (0.000246%)
GAAGAGCACACGTCTGAACTCCAGTCACGAGATTCCATCT	23 (0.000174%)
GAAGCACGCCCATCCAACCTAGGCGAGACAAGGGTTCACATTTCGTTCATCACCCTTGGCCGGCTATCGAACAGCCGGACTCCCATCAAAAGATGGTTGC	18 (0.000341%)
GAAGCACGCCCATCCAACCTAGGCGAGACAAGGGTTCACATTTCGTTCATCACCCTTGGCCGGCTTTCGAACAGCCGGACTCCCATCAAAAGATGGTTGC	10 (0.000189%)
GAAGTCGAAATCCGCTAAGGAGTGTGTAACAACTCACCTGCCGAATCAACTAGCCCCGAAAATGGATGGCGCTTAAGCGCGCGACCTATACCCGGCCGTC	4 (0.000076%)
GAATATGACTTGATGTCATGTGTATGATTGAGTATAAGAA	480 (0.003632%)
GAATCAAACGAGCTTTTACCCTTTTGTTCCACACGAGATTTCTGTTCTCGTTGAGCTCATCTTAGGACACCTGCGTTATCTTTTAACAGATGTGCCGCCC	3 (0.000057%)
GAATCATCAGAGCAACGGGCAGAGCCCGCGTCGACCTTTTATCTAATAAATGCGTCCCTTCCATAAGTCGGGGTTTGTTGCACGTATTAGCTCTAGAATT	123 (0.002327%)
GAATCCTTGTTAGTTTCTTTTCCTCCGCTTATTGATATGCTTAAACTCAGCGGGTAATCCCGCCTGACCTGGGGTCGCTATATGGACTTTGGGTCATCTA	6 (0.000114%)
GAATGCCAGCCGTTCGTTTGCATGTTCCTTGACACTTTTCGTGCCGGGGTTTTGTGATATCCGGAAGCAACGCGCACGACAAGACCGAGATAAAAGCTCC	18 (0.000341%)
GAATGGCTCATTAAATCAGTTATAGTTTGTTTGATGGTAACTACTACTCGGATAACCGTAGTAATTCTAGAGCTAATACGTGCAACAAACCCCGACTTAT	10 (0.000189%)
GAATTAGGGTTCGATTCCGGAGAGGGAGCCTGAGAAACGGCTACCACATCCAAGGAAGGCAGCAGGCGCGCAAATTACCCAATCCTGACACGGGGAGGTA	10 (0.000189%)
GAATTGTATCCTTGTTAGAAGACACAAAGCCAAAGACTCATATGGACTTTGGCTACACCATGAAAGCTTTGAGAAGCAAGAAGAAGGTTGGTTAGTGTTT	22 (0.000416%)
GACAAGGGTTCACATTTCGTTCATCACCCTTGGCCGGCTATCGAACAGCCGGACTCCCATCAAAAGATGGTTGCCAAGAACATCTTCGTTACGGTTTGCT	11 (0.000208%)
GACAAGGGTTCACATTTCGTTCATCACCCTTGGCCGGCTTTCGAACAGCCGGACTCCCATCAAAAGATGGTTGCCAAGAACATCTTCGTTACGGTTTGCT	12 (0.000227%)
GACACAAAGCCAAAGACTCATATGGACTTTGGCTACACCATGAAAGCTTTGAGAAGCAAGAAGAAGGTTGGTTAGTGTTTTGGAGTCGAATATGACTTGA	53 (0.001003%)
GACATCAAGTCATATTCGACTCCAAAACACTAACCAACCTTCTTCTTGCTTCTCAAAGCTTTCATGGTGTAGCCAAAGTCCATATGAGTCTTTGGCTTTG	42 (0.000795%)
GACATTGTCAGGTGGGGAGTTTGGCTGGGGCGGCACATCTGTTAAAAGATAACGCAGGTGTCCTAAGATGAGCTCAACGAGAACAGAAATCTCGTGTGGA	33 (0.000624%)
GACCAGAGGCTGTTCACCTTGGAGACCTGATGCGGTTATGAGTACGACCGGGCGTGAGCGGCACTCGGTCCTCCGGATTTTCAAGGGCCGCCGGGGGCGC	3 (0.000057%)
GACCCTGTTGAGCTTGACTCTAGTCCGACTTTGTGAAATGACTTGAGAGGTGTAGGATAAGTGGGAGCTTCGGCGCAAGTGAAATACCACTACTTTTAAC	3 (0.000057%)
GACCGGATTGCTCCGTTCCGCATCCGACCAGGACGCATCGCCGGCCCCCATCCGCTTCCCTCCCGACAATTTCAAGCACTCTTTGACTCTCTTTTCAAAG	9 (0.000170%)
GACCTATACCCGGCCGTCGGGGCAAGAGCCAGGCCTCGATGAGTAGGAGGGCGCGGCGGTCGCTGCAAAACCTAGGGCGCGAGCCCGGGCGGAGCGGCCG	10 (0.000189%)
GACCTTTTATCTAATAAATGCGTCCCTTCCATAAGTCGGGGTTTGTTGCACGTATTAGCTCTAGAATTACTACGGTTATCCGAGTAGTAGTTACCATCAA	10 (0.000189%)
GACGGAGAATTAGGGTTCGATTCCGGAGAGGGAGCCTGAGAAACGGCTACCACATCCAAGGAAGGCAGCAGGCGCGCAAATTACCCAATCCTGACACGGG	7 (0.000132%)
GACGGGCGGTGTGTACAAAGGGCAGGGACGTAGTCAACGCGAGCTGATGACTCGCGCTTACTAGGAATTCCTCGTTGAAGACCAACAATTGCAATGATCG	11 (0.000208%)
GACGTGGGTGGTTCGCCGCCCGCGACGTCGCGAGAAGTCCACTAAACCTTATCATTTAGAGGAAGGAGAAGTCGTAACAAGGTTTCCGTAGGTGAACCTG	48 (0.000908%)
GACTCATATGGACTTTGGCTACACCATGAAAGCTTTGAGAAGCAAGAAGAAGGTTGGTTAGTGTTTTGGAGTCGAATATGACTTGATGTCATGTGTATGA	4 (0.000076%)
GACTCCAAAACACTAACCAACCTTCTTCTTGCTTCTCAAAGCTTTCATGGTGTAGCCAAAGTCCATATGAGTCTTTGGCTTTGTGTCTTCTAACAAGGAA	265 (0.005013%)
GACTCTAGTCCGACTTTGTGAAATGACTTGAGAGGTGTAGGATAAGTGGGAGCTTCGGCGCAAGTGAAATACCACTACTTTTAACGTTATTTTACTTACT	4 (0.000076%)
GACTTCCCTTGCCTACATTGTTCCATCGACCAGAGGCTGTTCACCTTGGAGACCTGATGCGGTTATGAGTACGACCGGGCGTGAGCGGCACTCGGTCCTC	19 (0.000359%)
GACTTGATGTCATGTGTATGATTGAGTATAAGAACTTAAA	138 (0.001044%)
GACTTTGGCTACACCATGAAAGCTTTGAGAAGCAAGAAGAAGGTTGGTTAGTGTTTTGGAGTCGAATATGACTTGATGTCATGTGTATGATTGAGTATAA	50 (0.000946%)
GACTTTGTGAAATGACTTGAGAGGTGTAGGATAAGTGGGAGCTTCGGCGCAAGTGAAATACCACTACTTTTAACGTTATTTTACTTACTCCGTGAATCGG	6 (0.000114%)
GAGAATTAGGGTTCGATTCCGGAGAGGGAGCCTGAGAAACGGCTACCACATCCAAGGAAGGCAGCAGGCGCGCAAATTACCCAATCCTGACACGGGGAGG	11 (0.000208%)
GAGACAAGGGTTCACATTTCGTTCATCACCCTTGGCCGGCTATCGAACAGCCGGACTCCCATCAAAAGATGGTTGCCAAGAACATCTTCGTTACGGTTTG	6 (0.000114%)
GAGACAAGGGTTCACATTTCGTTCATCACCCTTGGCCGGCTTTCGAACAGCCGGACTCCCATCAAAAGATGGTTGCCAAGAACATCTTCGTTACGGTTTG	8 (0.000151%)

GAGCAACGGGCAGAGCCCGCGTCGACCTTTTATCTAATAAATGCGTCCCTTCCATAAGTCGGGGTTTGTTGCACGTATTAGCTCTAGAATTACTACGGTT	19 (0.000359%)	
GAGCCCGCGTCGACCTTTTATCTAATAAATGCGTCCCTTCCATAAGTCGGGGTTTGTTGCACGTATTAGCTCTAGAATTACTACGGTTATCCGAGTAGTA	7 (0.000132%)	
GAGCGACGGGCGGTGTGTACAAAGGGCAGGGACGTAGTCAACGCGAGCTGATGACTCGCGCTTACTAGGAATTCCTCGTTGAAGACCAACAATTGCAATG	15 (0.000284%)	
GAGCGAGGTGTGAGTGTCGCCCATGGGCATCGACACCTTGCGGCTAGGAACTGGAACGAGACGGGTAGCAAAGATTTCGAGTAGCACTTCATACTACCGT	7 (0.000132%)	
GAGCGAGGTGTGAGTGTCGCCCATGGGCATCGACACCTTGCGGCTAGGAACTGGAACGAGACGGGTGGCAAAGATTTCGAGTAGCACTTCATACTACCGT	4 (0.000076%)	
GAGCGTAGGCTTGCTTTGAGCACTCTAATTTCTTCAAAGTAACAGCGCCGGAGGCACGACCCGGCCAATTAAGACCAGGAGCGTATCGCCGACCGAAGGG	3 (0.000057%)	
GAGCTTGACTCTAGTCCGACTTTGTGAAATGACTTGAGAGGTGTAGGATAAGTGGGAGCTTCGGCGCAAGTGAAATACCACTACTTTTAACGTTATTTTA	4 (0.000076%)	
GAGGACATTGTCAGGTGGGGAGTTTGGCTGGGGCGGCACATCTGTTAAAAGATAACGCAGGTGTCCTAAGATGAGCTCAACGAGAACAGAAATCTCGTGT	50 (0.000946%)	
GAGGCGAATGCCAGCCGTTCGTTTGCATGTTCCTTGACACTTTTCGTGCCGGGGTTTTGTGATATCCGGAAGCAACGCGCACGACAAGACCGAGATAAAA	8 (0.000151%)	
GAGGGCAAGTCTGGTGCCAGCAGCCGCGGTAATTCCAGCTCCAATAGCGTATATTTAAGTTGTTGCAGTTAAAAAGCTCGTAGTTGAACCTTGGGATGGG	19 (0.000359%)	
GAGGTGTGAGTGTCGCCCATGGGCATCGACACCTTGCGGCTAGGAACTGGAACGAGACGGGTGGCAAAGATTTCGAGTAGCACTTCATACTACCGTGGGT	14 (0.000265%)	
GAGTTATCATGAATCATCAGAGCAACGGGCAGAGCCCGCGTCGACCTTTTATCTAATAAATGCGTCCCTTCCATAAGTCGGGGTTTGTTGCACGTATTAG	69 (0.001305%)	
GAGTTTGGCTGGGGCGGCACATCTGTTAAAAGATAACGCAGGTGTCCTAAGATGAGCTCAACGAGAACAGAAATCTCGTGTGGAACAAAAGGGTAAAAGC	3 (0.000057%)	
GATACTAAATCCTATTTTCTGGTAAATTTTCATAATTTTTTGACACCTCTAGCTAGGTCATTTGACCTGATACAACATCGGATTTTCATGGTCTAGTTGG	3 (0.000057%)	
GATAGGCCACGCTTTCACGGTTCGTATTCGTACTGAAAATCAGAATCAAACGAGCTTTTACCCTTTTGTTCCACACGAGATTTCTGTTCTCGTTGAGCTC	1738 (0.032880%)	
GATAGTGGCCTACCATGGTGGTAACGGGTGACGGAGAATTAGGGTTCGATTCCGGAGAGGGAGCCTGAGAAACGGCTACCACATCCAAGGAAGGCAGCAG	4 (0.000076%)	
GATCAAGTTCACCACCGCATGTCGGTACGCTCCAGGCGTCCTTGGCTCGGATTTAGGCCAACCGCGTGCGGTAACACACGGGAGACCAGCTTCCGTCCCG	57 (0.001078%)	
GATCACGGCAATTCCCCGCCACATCCTCTCAAACGCAATGGAAAGAGAGAAAGGACGAGGTCTTGACCGTCATCTTTTGCCCGAAGGACGGATGAGCTTT	31 (0.000586%)	
GATCATTCAATCGGTAGGAGCGACGGGCGGTGTGTACAAAGGGCAGGGACGTAGTCAACGCGAGCTGATGACTCGCGCTTACTAGGAATTCCTCGTTGAA	837 (0.015835%)	
GATCCATGCTTTCCAACGAAGCACGCCCATCCAACCTAGGCGAGACAAGGGTTCACATTTCGTTCATCACCCTTGGCCGGCTATCGAACAGCCGGACTCC	106 (0.002005%)	
GATCCATTGGAGGGCAAGTCTGGTGCCAGCAGCCGCGGTAATTCCAGCTCCAATAGCGTATATTTAAGTTGTTGCAGTTAAAAAGCTCGTAGTTGAACCT	1540 (0.029134%)	
GATCCGCCTAGGCTGTCCCGAGTGTGAGCGAGGTGTGAGTGTCGCCCATGGGCATCGACACCTTGCGGCTAGGAACTGGAACGAGACGGGTAGCAAAGAT	124 (0.002346%)	
GATCCGCCTAGGCTGTCCCGAGTGTGAGCGAGGTGTGAGTGTCGCCCATGGGCATCGACACCTTGCGGCTAGGAACTGGAACGAGACGGGTGGCAAAGAT	168 (0.003178%)	
GATCCGGTTAAAATTCCGGAACCGGGACGTGGCGGTTGACGGCAACGTTAGGGAGTCCGGAGACGTCGGCGGGGGCCTCGGGAAGAGTTATCTTTTCTGT	135 (0.002554%)	
GATCCGTAACTTCGGGAAAAGGATTGGCTCTGAGGGCTGGGCTCGGGGGTCCCAGTTCCGAACCCGTCGGCTGTCAGCGGACTGCTCGAGCTGCTTCCGC	29 (0.000549%)	
GATCCGTCGAGTTATCATGAATCATCAGAGCAACGGGCAGAGCCCGCGTCGACCTTTTATCTAATAAATGCGTCCCTTCCATAAGTCGGGGTTTGTTGCA	116 (0.002195%)	
GATCCTCGTTAAGGGATTTAGATTGTACTCATTCCAATTACCAGACTCGAAAGAGCCCGGTATTGTTATTTATTGTCACTACCTCCCCGTGTCAGGATTG	96 (0.001816%)	
GATCGAAATCCTATGATGTTATCCCATGCTAATGTATCCAGAGCGTAGGCTTGCTTTGAGCACTCTAATTTCTTCAAAGTAACAGCGCCGGAGGCACGAC	410 (0.007756%)	
GATCGATCAA	145598 (0.275446%)	
GATCGATCAAGTTCACCACCGCATGTCGGTACGCTCCAGGCGTCCTTGGCTCGGATTTAGGCCAACCGCGTGCGGTAACACACGGGAGACCAGCTTCCGT	1241 (0.023478%)	
GATCGATCACGGCAATTCCCCGCCACATCCTCTCAAACGCAATGGAAAGAGAGAAAGGACGAGGTCTTGACCGTCATCTTTTGCCCGAAGGACGGATGAG	1028 (0.019448%)	
GATCGATCCATGCTTTCCAACGAAGCACGCCCATCCAACCTAGGCGAGACAAGGGTTCACATTTCGTTCATCACCCTTGGCCGGCTATCGAACAGCCGGA	1450 (0.027431%)	
GATCGATCCGCCTAGGCTGTCCCGAGTGTGAGCGAGGTGTGAGTGTCGCCCATGGGCATCGACACCTTGCGGCTAGGAACTGGAACGAGACGGGTAGCAA	1037 (0.019618%)	
GATCGATCCGCCTAGGCTGTCCCGAGTGTGAGCGAGGTGTGAGTGTCGCCCATGGGCATCGACACCTTGCGGCTAGGAACTGGAACGAGACGGGTGGCAA	1584 (0.029966%)	
GATCGATCCGGTTAAAATTCCGGAACCGGGACGTGGCGGTTGACGGCAACGTTAGGGAGTCCGGAGACGTCGGCGGGGGCCTCGGGAAGAGTTATCTTTT	1600 (0.030269%)	
GATCGATCCGTAACTTCGGGAAAAGGATTGGCTCTGAGGGCTGGGCTCGGGGGTCCCAGTTCCGAACCCGTCGGCTGTCAGCGGACTGCTCGAGCTGCTT	1276 (0.024140%)	
GATCGATCCGTCGAGTTATCATGAATCATCAGAGCAACGGGCAGAGCCCGCGTCGACCTTTTATCTAATAAATGCGTCCCTTCCATAAGTCGGGGTTTGT	1106 (0.020924%)	
GATCGATCCTCGTTAAGGGATTTAGATTGTACTCATTCCAATTACCAGACTCGAAAGAGCCCGGTATTGTTATTTATTGTCACTACCTCCCCGTGTCAGG	1484 (0.028075%)	
GATCGATCGAAATCCTATGATGTTATCCCATGCTAATGTATCCAGAGCGTAGGCTTGCTTTGAGCACTCTAATTTCTTCAAAGTAACAGCGCCGGAGGCA	1275 (0.024121%)	
GATCGATCGGCGGACCGGATTGCTCCGTTCCGCATCCGACCAGGACGCATCGCCGGCCCCCATCCGCTTCCCTCCCGACAATTTCAAGCACTCTTTGACT	868 (0.016421%)	
GATCGATCGGGTTGCGGTTTAAGTTGTTATACTCAATCAT	4573 (0.034605%)	
GATCGATCTT	168028 (0.317879%)	
GATCGGAAGAGCACACGTCTGAACTCCAGTCACGAGATTC	35 (0.000265%)	

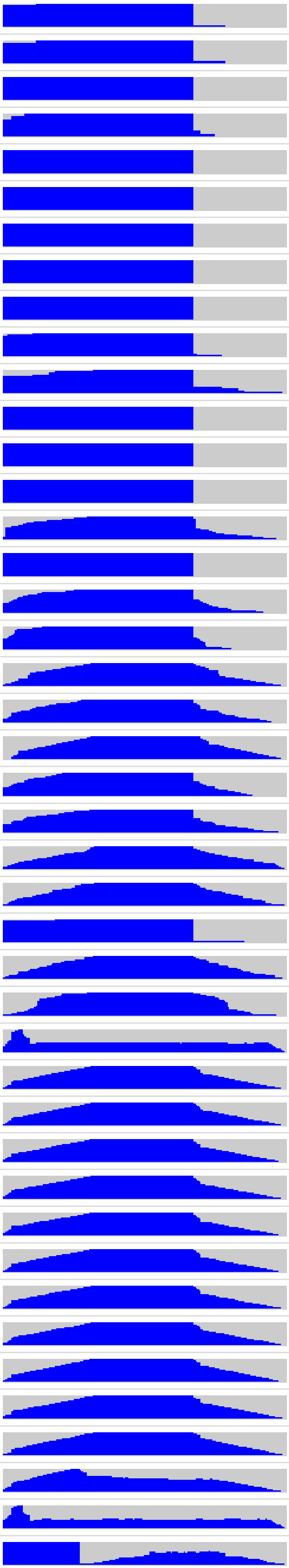

GATCGGCGGACCGGATTGCTCCGTTCCGCATCCGACCAGGACGCATCGCCGGCCCCCATCCGCTTCCCTCC CGACAATTTCAAGCACTCTTTGACTCTCT	15 (0.000284%)	
GATCGGGTTGCGGTTTAAGTTGTTATACTCAATCATACAC	1413 (0.010693%)	
GATCTTAAAGGCGTAAGAATTGTATCCTTGTTAAAAGACACAAAGCCAAAGACTCATATGGACTTTGGCTA CACCATGAAAGCTTTGAGAAGCAAGAAGA	707 (0.013375%)	
GATCTTAAAGGCGTAAGAATTGTATCCTTGTTAGAAGACACAAAGCCAAAGACTCATATGGACTTTGGCTA CACCATGAAAGCTTTGAGAAGCAAGAAGA	516 (0.009762%)	
GATGCCCTTAGATGTTCTGGGCCGCACGCGCGCTACACTGATGTATTCAACGAGTTCACACCTTGGCCGAC AGGCCCGGGTAATCTTTGAAATTTCATCG	8 (0.000151%)	
GATGTCATGTGTATGATTGAGTATAAGAACTTAAACCGCA	172 (0.001302%)	
GATGTTATCCCATGCTAATGTATCCAGAGCGTAGGCTTGCTTTGAGCACTCTAATTTCTTCAAAGTAACAG CGCCGGAGGCACGACCCGGCCAATTAAGA	10 (0.000189%)	
GATGTTCTGGGCCGCACGCGCGCTACACTGATGTATTCAACGAGTTCACACCTTGGCCGACAGGCCCGGGT AATCTTTGAAATTTCATCGTGATGGGGAT	5 (0.000095%)	
GATTAACAGGGACAGTCGGGGGCATTCGTATTTCATAGTCAGAGGTGAAATTCTTGGATTTATGAAAGACG AACAACTGCGAAAGCATTTGCCAAGGATG	196 (0.003708%)	
GATTAAGCCATGCATGTGTAAGTATGAACGAATTCAGACTGTGAAACTGCGAATGGCTCATTAAATCAGTT ATAGTTTGTTTGATGGTAACTACTACTCG	19 (0.000359%)	
GATTGTACTCATTCCAATTACCAGACTCGAAAGAGCCCGGTATTGTTATTTATTGTCACTACCTCCCCGTG TCAGGATTGGGTAATTTGCGCGCCTGCTG	7 (0.000132%)	
GATTTAGATTGTACTCATTCCAATTACCAGACTCGAAAGAGCCCGGTATTGTTATTTATTGTCACTACCTC CCCGTGTCAGGATTGGGTAATTTGCGCGC	18 (0.000341%)	
GCAACCGGATCTTAAAGGCGTAAGAATTGTATCCTTGTTAGAAGACACAAAGCCAAAGACTCATATGGACT TTGGCTACACCATGAAAGCTTTGAGAAGC	3 (0.000057%)	
GCAACGGGCAGAGCCCGCGTCGACCTTTTATCTAATAAATGCGTCCCTTCCATAAGTCGGGGTTTGTTGCA CGTATTAGCTCTAGAATTACTACGGTTAT	40 (0.000757%)	
GCAACGTCGCTATGAACGCTTGGCTGCCACAAGCCAGTTATCCCTGTGGTAACTTTTCTGACACCTCTAGC TTCAAATTCCGAAGGTCTAAAGGATCGAT	61 (0.001154%)	
GCAAGTCTGGTGCCAGCAGCCGCGGTAATTCCAGCTCCAATAGCGTATATTTAAGTTGTTGCAGTTAAAAA GCTCGTAGTTGAACCTTGGGATGGGTCGG	26 (0.000492%)	
GCAATAACAGGTCTGTGATGCCCTTAGATGTTCTGGGCCGCACGCGCGCTACACTGATGTATTCAACGAGT TCACACCTTGGCCGACAGGCCCGGGTAAT	1004 (0.018994%)	
GCAATTCCCCGCCACATCCTCTCAAACGCAATGGAAAGAGAGAAAGGACGAGGTCTTGACCGTCATCTTTT GCCCGAAGGACGGATGAGCTTTGGCGGGA	32 (0.000605%)	
GCACACGTCTGAACTCCAGTCACGAGATTCCATCTCGTAT	6 (0.000045%)	
GCACGCCCATCCAACCTAGGCGAGACAAGGGTTCACATTTCGTTCATCACCCTTGGCCGGCTATCGAACAG CCGGACTCCCATCAAAAGATGGTTGCCAA	29 (0.000549%)	
GCACGCCCATCCAACCTAGGCGAGACAAGGGTTCACATTTCGTTCATCACCCTTGGCCGGCTTTCGAACAG CCGGACTCCCATCAAAAGATGGTTGCCAA	25 (0.000473%)	
GCACGCGCCTAACGGCGTGCCTCGGCATCAGCGTGCTCCGGGCGTCGGCCTGTGGGCTCCCCATTCGACCC GTCTTGAAACACGGACCAAGGAGTCTGAC	23 (0.000435%)	
GCAGAGCCCGCGTCGACCTTTTATCTAATAAATGCGTCCCTTCCATAAGTCGGGGTTTGTTGCACGTATTA GCTCTAGAATTACTACGGTTATCCGAGTA	18 (0.000341%)	
GCAGCACGCGCCTAACGGCGTGCCTCGGCATCAGCGTGCTCCGGGCGTCGGCCTGTGGGCTCCCCATTCGA CCCGTCTTGAAACACGGACCAAGGAGTCT	461 (0.008721%)	
GCAGCCGCGGTAATTCCAGCTCCAATAGCGTATATTTAAGTTGTTGCAGTTAAAAAGCTCGTAGTTGAACC TTGGGATGGGTCGGCCGGTCCGCCTTTGG	10 (0.000189%)	
GCATCAGCGTGCTCCGGGCGTCGGCCTGTGGGCTCCCCATTCGACCCGTCTTGAAACACGGACCAAGGAGT CTGACATGTGTGCGAGTCAACGGGTGAGT	11 (0.000208%)	
GCATCATAAGGATACTAAATCCTATTTTCTGGTAAATTTTCATAATTTTTTGACACCTCTAGCTAGGTCAT TTGACCTGATACAACATCGGATTTTCATG	11 (0.000208%)	
GCATGTCGGTACGCTCCAGGCGTCCTTGGCTCGGATTTAGGCCAACCGCGTGCGGTAACACACGGGAGACC AGCTTCCGTCCCGCATCAGCAAAGGATGG	4 (0.000076%)	
GCATGTGTAAGTATGAACGAATTCAGACTGTGAAACTGCGAATGGCTCATTAAATCAGTTATAGTTTGTTT GATGGTAACTACTACTCGGATAACCGTAG	8 (0.000151%)	
GCCAAAGACTCATATGGACTTTGGCTACACCATGAAAGCTTTGAGAAGCAAGAAGAAGGTTGGTTAGTGTT TTGGAGTCGAATATGACTTGATGTCATGT	46 (0.000870%)	
GCCACATCCTCTCAAACGCAATGGAAAGAGAGAAAGGACGAGGTCTTGACCGTCATCTTTTGCCCGAAGGA CGGATGAGCTTTGGCGGGACTGAATCACT	17 (0.000322%)	
GCCACCCTAACGCCTCGAAGAACTAATGGCAGCCCACGCAAGGCAAGCCCATTCTCCTCGACGATTCAGCA GTTTTTGTCCGAGAACTGCTGAGAAAACT	868 (0.016421%)	
GCCACGCTTTCACGGTTCGTATTCGTACTGAAAATCAGAATCAAACGAGCTTTTACCCTTTTGTTCCACAC GAGATTTCTGTTCTCGTTGAGCTCATCTT	167 (0.003159%)	
GCCAGCAGCCGCGGTAATTCCAGCTCCAATAGCGTATATTTAAGTTGTTGCAGTTAAAAAGCTCGTAGTTG AACCTTGGGATGGGTCGGCCGGTCCGCCT	30 (0.000568%)	
GCCAGCCGTTCGTTTGCATGTTCCTTGACACTTTTCGTGCCGGGGTTTTGTGATATCCGGAAGCAACGCGC ACGACAAGACCGAGATAAAAGCTCCCGAT	36 (0.000681%)	
GCCAGTAGTCATATGCTTGTCTCAAAGATTAAGCCATGCATGTGTAAGTATGAACGAATTCAGACTGTGAA ACTGCGAATGGCTCATTAAATCAGTTATA	233 (0.004408%)	
GCCATGCATGTGTAAGTATGAACGAATTCAGACTGTGAAACTGCGAATGGCTCATTAAATCAGTTATAGTT TGTTTGATGGTAACTACTACTCGGATAAC	10 (0.000189%)	
GCCCATCCAACCTAGGCGAGACAAGGGTTCACATTTCGTTCATCACCCTTGGCCGGCTATCGAACAGCCGG ACTCCCATCAAAAGATGGTTGCCAAGAAC	47 (0.000889%)	
GCCCATCCAACCTAGGCGAGACAAGGGTTCACATTTCGTTCATCACCCTTGGCCGGCTTTCGAACAGCCGG ACTCCCATCAAAAGATGGTTGCCAAGAAC	45 (0.000851%)	
GCCCATGGGCATCGACACCTTGCGGCTAGGAACTGGAACGAGACGGGTGGCAAAGATTTCGAGTAGCACTT CATACTACCGTGGGTTTTTTAAACCTTCC	10 (0.000189%)	
GCCCGCGTCGACCTTTTATCTAATAAATGCGTCCCTTCCATAAGTCGGGGTTTGTTGCACGTATTAGCTCT AGAATTACTACGGTTATCCGAGTAGTAGT	3 (0.000057%)	
GCCCTTAGATGTTCTGGGCCGCACGCGCGCTACACTGATGTATTCAACGAGTTCACACCTTGGCCGACAGG CCCGGGTAATCTTTGAAATTTCATCGTGA	14 (0.000265%)	
GCCGACTTCCCTTGCCTACATTGTTCCATCGACCAGAGGCTGTTCACCTTGGAGACCTGATGCGGTTATGA GTACGACCGGGCGTGAGCGGCACTCGGTC	228 (0.004313%)	

GCCGCCCGCGACGTCGCGAGAAGTCCACTAAACCTTATCATTTAGAGGAAGGAGAAGTCGTAACAAGGTTTCCGTAGGTGAACCTGCGGAAGGATCGATC	18 (0.000341%)	
GCCGCGGTAATTCCAGCTCCAATAGCGTATATTTAAGTTGTTGCAGTTAAAAAGCTCGTAGTTGAACCTTGGGATGGGTCGGCCGGTCCGCCTTTGGTGT	14 (0.000265%)	
GCCGTCGGGGCAAGAGCCAGGCCTCGATGAGTAGGAGGGCGCGGCGGTCGCTGCAAAACCTAGGGCGCGAGCCCGGGCGGAGCGGCCGTCGGTGCAGATC	9 (0.000170%)	
GCCGTTACTAAGGGAATCCTTGTTAGTTTCTTTTCCTCCGCTTATTGATATGCTTAAACTCAGCGGGTAATCCCGCCTGACCTGGGGTCGCTATATGGAC	22 (0.000416%)	
GCCTAACGGCGTGCCTCGGCATCAGCGTGCTCCGGGCGTCGGCCTGTGGGCTCCCCATTCGACCCGTCTTGAAACACGGACCAAGGAGTCTGACATGTGT	43 (0.000813%)	
GCCTAAGTAGTGTTTCCTTGTTAGAAGACACAAAGCCAAAGACTCATATGGACTTTGGCTACACCATGAAAGCTTTGAGAAGCAAGAAGAAGGTTGGTTA	49 (0.000927%)	
GCCTACATTGTTCCATCGACCAGAGGCTGTTCACCTTGGAGACCTGATGCGGTTATGAGTACGACCGGGCGTGAGCGGCACTCGGTCCTCCGGATTTTCA	6 (0.000114%)	
GCCTAGGCTGTCCCGAGTGTGAGCGAGGTGTGAGTGTCGCCCATGGGCATCGACACCTTGCGGCTAGGAACTGGAACGAGACGGGTAGCAAAGATTTCGA	267 (0.005051%)	
GCCTAGGCTGTCCCGAGTGTGAGCGAGGTGTGAGTGTCGCCCATGGGCATCGACACCTTGCGGCTAGGAACTGGAACGAGACGGGTGGCAAAGATTTCGA	379 (0.007170%)	
GCCTCGAAGAACTAATGGCAGCCCACGCAAGGCAAGCCCATTCTCCTCGACGATTCAGCAGTTTTTGTCCGAGAACTGCTGAGAAAACTCGGAAAAAGGC	13 (0.000246%)	
GCCTCGGCATCAGCGTGCTCCGGGCGTCGGCCTGTGGGCTCCCCATTCGACCCGTCTTGAAACACGGACCAAGGAGTCTGACATGTGTGCGAGTCAACGG	17 (0.000322%)	
GCGAATGCCAGCCGTTCGTTTGCATGTTCCTTGACACTTTTCGTGCCGGGGTTTTGTGATATCCGGAAGCAACGCGCACGACAAGACCGAGATAAAAGCT	27 (0.000511%)	
GCGAATGGCTCATTAAATCAGTTATAGTTTGTTTGATGGTAACTACTACTCGGATAACCGTAGTAATTCTAGAGCTAATACGTGCAACAAACCCCGACTT	22 (0.000416%)	
GCGACCTATACCCGGCCGTCGGGGCAAGAGCCAGGCCTCGATGAGTAGGAGGGCGCGGCGGTCGCTGCAAAACCTAGGGCGCGAGCCCGGGCGGAGCGGC	112 (0.002119%)	
GCGACGGGCGGTGTGTACAAAGGGCAGGGACGTAGTCAACGCGAGCTGATGACTCGCGCTTACTAGGAATTCCTCGTTGAAGACCAACAATTGCAATGAT	47 (0.000889%)	
GCGACGTGGGTGGTTCGCCGCCCGCGACGTCGCGAGAAGTCCACTAAACCTTATCATTTAGAGGAAGGAGAAGTCGTAACAAGGTTTCCGTAGGTGAACC	2219 (0.041980%)	
GCGAGACAAGGGTTCACATTTCGTTCATCACCCTTGGCCGGCTATCGAACAGCCGGACTCCCATCAAAAGATGGTTGCCAAGAACATCTTCGTTACGGTT	31 (0.000586%)	
GCGAGACAAGGGTTCACATTTCGTTCATCACCCTTGGCCGGCTTTCGAACAGCCGGACTCCCATCAAAAGATGGTTGCCAAGAACATCTTCGTTACGGTT	28 (0.000530%)	
GCGAGGTGTGAGTGTCGCCCATGGGCATCGACACCTTGCGGCTAGGAACTGGAACGAGACGGGTAGCAAAGATTTCGAGTAGCACTTCATACTACCGTGG	26 (0.000492%)	
GCGAGGTGTGAGTGTCGCCCATGGGCATCGACACCTTGCGGCTAGGAACTGGAACGAGACGGGTGGCAAAGATTTCGAGTAGCACTTCATACTACCGTGG	44 (0.000832%)	
GCGCCTAACGGCGTGCCTCGGCATCAGCGTGCTCCGGGCGTCGGCCTGTGGGCTCCCCATTCGACCCGTCTTGAAACACGGACCAAGGAGTCTGACATGT	24 (0.000454%)	
GCGCGACCTATACCCGGCCGTCGGGGCAAGAGCCAGGCCTCGATGAGTAGGAGGGCGCGGCGGTCGCTGCAAAACCTAGGGCGCGAGCCCGGGCGGAGCG	30 (0.000568%)	
GCGGACCGGATTGCTCCGTTCCGCATCCGACCAGGACGCATCGCCGGCCCCCATCCGCTTCCCTCCCGACAATTTCAAGCACTCTTTGACTCTCTTTTCA	17 (0.000322%)	
GCGGAGGACATTGTCAGGTGGGGAGTTTGGCTGGGGCGGCACATCTGTTAAAAGATAACGCAGGTGTCCTAAGATGAGCTCAACGAGAACAGAAATCTCG	1465 (0.027715%)	
GCGGCACATCTGTTAAAAGATAACGCAGGTGTCCTAAGATGAGCTCAACGAGAACAGAAATCTCGTGTGGAACAAAAGGGTAAAAGCTCGTTTGATTCTG	15 (0.000284%)	
GCGGTAATTCCAGCTCCAATAGCGTATATTTAAGTTGTTGCAGTTAAAAAGCTCGTAGTTGAACCTTGGGATGGGTCGGCCGGTCCGCCTTTGGTGTGCA	22 (0.000416%)	
GCGGTTTAAGTTCTTATACTCAATCATACACATGACATCA	640 (0.004843%)	
GCGTAAGAATTGTATCCTTGTTAGAAGACACAAAGCCAAAGACTCATATGGACTTTGGCTACACCATGAAAGCTTTGAGAAGCAAGAAGAAGGTTGGTTA	12 (0.000227%)	
GCGTAGGCTTGCTTTGAGCACTCTAATTTCTTCAAAGTAACAGCGCCGGAGGCACGACCCGGCCAATTAAGACCAGGAGCGTATCGCCGACCGAAGGGAC	49 (0.000927%)	
GCGTCGACCTTTTATCTAATAAATGCGTCCCTTCCATAAGTCGGGGTTTGTTGCACGTATTAGCTCTAGAATTACTACGGTTATCCGAGTAGTAGTTACC	11 (0.000208%)	
GCGTCGGCCTGTGGGCTCCCCATTCGACCCGTCTTGAAACACGGACCAAGGAGTCTGACATGTGTGCGAGTCAACGGGTGAGTAAACCCGTAAGGCGCAA	5 (0.000095%)	
GCGTGCCTCGGCATCAGCGTGCTCCGGGCGTCGGCCTGTGGGCTCCCCATTCGACCCGTCTTGAAACACGGACCAAGGAGTCTGACATGTGTGCGAGTCA	5 (0.000095%)	
GCGTGCTCCGGGCGTCGGCCTGTGGGCTCCCCATTCGACCCGTCTTGAAACACGGACCAAGGAGTCTGACATGTGTGCGAGTCAACGGGTGAGTAAACCC	21 (0.000397%)	
GCTAAGGAGTGTGTAACAACTCACCTGCCGAATCAACTAGCCCCGAAAATGGATGGCGCTTAAGCGCGCGACCTATACCCGGCCGTCGGGGCAAGAGCCA	58 (0.001097%)	
GCTAATGTATCCAGAGCGTAGGCTTGCTTTGAGCACTCTAATTTCTTCAAAGTAACAGCGCCGGAGGCACGACCCGGCCAATTAAGACCAGGAGCGTATC	20 (0.000378%)	
GCTCATTAAATCAGTTATAGTTTGTTTGATGGTAACTACTACTCGGATAACCGTAGTAATTCTAGAGCTAATACGTGCAACAAACCCCGACTTATGGAAG	12 (0.000227%)	
GCTCCGGGCGTCGGCCTGTGGGCTCCCCATTCGACCCGTCTTGAAACACGGACCAAGGAGTCTGACATGTGTGCGAGTCAACGGGTGAGTAAACCCGTAA	7 (0.000132%)	
GCTCCGTTCCGCATCCGACCAGGACGCATCGCCGGCCCCCATCCGCTTCCCTCCCGACAATTTCAAGCACTCTTTGACTCTCTTTTCAAAGTCCTTTTCA	7 (0.000132%)	
GCTCGCCGTTACTAAGGGAATCCTTGTTAGTTTCTTTTCCTCCGCTTATTGATATGCTTAAACTCAGCGGGTAATCCCGCCTGACCTGGGGTCGCTATAT	59 (0.001116%)	
GCTGATGCGGGACGGAAGCTGGTCTCCCGTGTGTTACCGCACGCGGTTGGCCTAAATCCGAGCCAAGGACGCCTGGAGCGTACCGACATGCGGTGGTGAA	7 (0.000132%)	
GCTGGGGCGGCACATCTGTTAAAAGATAACGCAGGTGTCCTAAGATGAGCTCAACGAGAACAGAAATCTCGTGTGGAACAAAAGGGTAAAAGCTCGTTTG	10 (0.000189%)	
GCTGTCCCGAGTGTGAGCGAGGTGTGAGTGTCGCCCATGGGCATCGACACCTTGCGGCTAGGAACTGGAACGAGACGGGTAGCAAAGATTTCGAGTAGCA	62 (0.001173%)	
GCTGTCCCGAGTGTGAGCGAGGTGTGAGTGTCGCCCATGGGCATCGACACCTTGCGGCTAGGAACTGGAACGAGACGGGTGGCAAAGATTTCGAGTAGCA	99 (0.001873%)	

GCTTAAGCGCGCGACCTATACCCGGCCGTCGGGGCAAGAGCCAGGCCTCGATGAGTAGGAGGGCGCGGCGGTCGCTGCAAAACCTAGGGCGCGAGCCCGG	1065 (0.020148%)	
GCTTATTGATATGCTTAAACTCAGCGGGTAATCCCGCCTGACCTGGGGTCGCTATATGGACTTTGGGTCATCTACAGCTTCCGGACAAGAGCGACCGATA	21 (0.000397%)	
GCTTCTCAAAGCTTTCATGGTGTAGCCAAAGTCCATATGAGTCTTTGGCTTTGTGTCTTCTAACAAGGAAACACTACTTAGGCTTATAAGATGCGGTTGC	14 (0.000265%)	
GCTTGACTCTAGTCCGACTTTGTGAAATGACTTGAGAGGTGTAGGATAAGTGGGAGCTTCGGCGCAAGTGAAATACCACTACTTTTAACGTTATTTTACT	32 (0.000605%)	
GCTTGCTTTGAGCACTCTAATTTCTTCAAAGTAACAGCGCCGGAGGCACGACCCGGCCAATTAAGACCAGGAGCGTATCGCCGACCGAAGGGACAAGCCG	16 (0.000303%)	
GCTTGTCTCAAAGATTAAGCCATGCATGTGTAAGTATGAACGAATTCAGACTGTGAAACTGCGAATGGCTCATTAAATCAGTTATAGTTTGTTTGATGGT	19 (0.000359%)	
GCTTTCACGGTTCGTATTCGTACTGAAAATCAGAATCAAACGAGCTTTTACCCTTTTGTTCCACACGAGATTTCTGTTCTCGTTGAGCTCATCTTAGGAC	13 (0.000246%)	
GCTTTCCAACGAAGCACGCCCATCCAACCTAGGCGAGACAAGGGTTCACATTTCGTTCATCACCCTTGGCCGGCTATCGAACAGCCGGACTCCCATCAAA	76 (0.001438%)	
GGAAAGAAGACCCTGTTGAGCTTGACTCTAGTCCGACTTTGTGAAATGACTTGAGAGGTGTAGGATAAGTGGGAGCTTCGGCGCAAGTGAAATACCACTA	760 (0.014378%)	
GGAAAGAGAGAAAGGACGAGGTCTTGACCGTCATCTTTTGCCCGAAGGACGGATGAGCTTTGGCGGGACTGAATCACTTCGAGTCACCGTCGACAACTTT	5 (0.000095%)	
GGAAGAGCACACGTCTGAACTCCAGTCACGAGATTCCATC	50 (0.000378%)	
GGAAGTCGAAATCCGCTAAGGAGTGTGTAACAACTCACCTGCCGAATCAACTAGCCCCGAAAATGGATGGCGCTTAAGCGCGCGACCTATACCCGGCCGT	23 (0.000435%)	
GGAATCCTTGTTAGTTTCTTTTCCTCCGCTTATTGATATGCTTAAACTCAGCGGGTAATCCCGCCTGACCTGGGGTCGCTATATGGACTTTGGGTCATCT	18 (0.000341%)	
GGACAGTCGGGGGCATTCGTATTTCATAGTCAGAGGTGAAATTCTTGGATTTATGAAAGACGAACAACTGCGAAAGCATTTGCCAAGGATGTTTTCATTA	66 (0.001249%)	
GGACATTGTCAGGTGGGGAGTTTGGCTGGGGCGGCACATCTGTTAAAAGATAACGCAGGTGTCCTAAGATGAGCTCAACGAGAACAGAAATCTCGTGTGG	184 (0.003481%)	
GGACCGGATTGCTCCGTTCCGCATCCGACCAGGACGCATCGCCGGCCCCCATCCGCTTCCCTCCCGACAATTTCAAGCACTCTTTGACTCTCTTTTCAAA	24 (0.000454%)	
GGACTTTGGCTACACCATGAAAGCTTTGAGAAGCAAGAAGAAGGTTGGTTAGTGTTTTGGAGTCGAATATGACTTGATGTCATGTGTATGATTGAGTATA	69 (0.001305%)	
GGAGAATTAGGGTTCGATTCCGGAGAGGGAGCCTGAGAAACGGCTACCACATCCAAGGAAGGCAGCAGGCGCGCAAATTACCCAATCCTGACACGGGGAG	102 (0.001930%)	
GGAGCGACGGGCGGTGTGTACAAAGGGCAGGGACGTAGTCAACGCGAGCTGATGACTCGCGCTTACTAGGAATTCCTCGTTGAAGACCAACAATTGCAAT	56 (0.001059%)	
GGAGGACATTGTCAGGTGGGGAGTTTGGCTGGGGCGGCACATCTGTTAAAAGATAACGCAGGTGTCCTAAGATGAGCTCAACGAGAACAGAAATCTCGTG	411 (0.007775%)	
GGAGGCGAATGCCAGCCGTTCGTTTGCATGTTCCTTGACACTTTTCGTGCCGGGGTTTTGTGATATCCGGAAGCAACGCGCACGACAAGACCGAGATAAA	13 (0.000246%)	
GGAGGGCAAGTCTGGTGCCAGCAGCCGCGGTAATTCCAGCTCCAATAGCGTATATTTAAGTTGTTGCAGTTAAAAAGCTCGTAGTTGAACCTTGGGATGG	45 (0.000851%)	
GGAGTTTGGCTGGGGCGGCACATCTGTTAAAAGATAACGCAGGTGTCCTAAGATGAGCTCAACGAGAACAGAAATCTCGTGTGGAACAAAAGGGTAAAAG	6 (0.000114%)	
GGATACTAAATCCTATTTTCTGGTAAATTTTCATAATTTTTTGACACCTCTAGCTAGGTCATTTGACCTGATACAACATCGGATTTTCATGGTCTAGTTG	12 (0.000227%)	
GGATAGTGGCCTACCATGGTGGTAACGGGTGACGGAGAATTAGGGTTCGATTCCGGAGAGGGAGCCTGAGAAACGGCTACCACATCCAAGGAAGGCAGCA	715 (0.013527%)	
GGATCTTAAAGGCGTAAGAATTGTATCCTTGTTAGAAGACACAAAGCCAAAGACTCATATGGACTTTGGCTACACCATGAAAGCTTTGAGAAGCAAGAAG	22 (0.000416%)	
GGATTGCTCCGTTCCGCATCCGACCAGGACGCATCGCCGGCCCCCATCCGCTTCCCTCCCGACAATTTCAAGCACTCTTTGACTCTCTTTTCAAAGTCCT	12 (0.000227%)	
GGCAAGTCTGGTGCCAGCAGCCGCGGTAATTCCAGCTCCAATAGCGTATATTTAAGTTGTTGCAGTTAAAAAGCTCGTAGTTGAACCTTGGGATGGGTCG	62 (0.001173%)	
GGCAATTCCCCGCCACATCCTCTCAAACGCAATGGAAAGAGAGAAAGGACGAGGTCTTGACCGTCATCTTTTGCCCGAAGGACGGATGAGCTTTGGCGGG	82 (0.001551%)	
GGCACATCTGTTAAAAGATAACGCAGGTGTCCTAAGATGAGCTCAACGAGAACAGAAATCTCGTGTGGAACAAAAGGGTAAAAGCTCGTTTGATTCTGAT	20 (0.000378%)	
GGCAGAGCCCGCGTCGACCTTTTATCTAATAAATGCGTCCCTTCCATAAGTCGGGGTTTGTTGCACGTATTAGCTCTAGAATTACTACGGTTATCCGAGT	20 (0.000378%)	
GGCATCAGCGTGCTCCGGGCGTCGGCCTGTGGGCTCCCCATTCGACCCGTCTTGAAACACGGACCAAGGAGTCTGACATGTGTGCGAGTCAACGGGTGAG	10 (0.000189%)	
GGCATCGACACCTTGCGGCTAGGAACTGGAACGAGACGGGTGGCAAAGATTTCGAGTAGCACTTCATACTACCGTGGGTTTTTTAAACCTTCCGAGTTTT	13 (0.000246%)	
GGCATGCATCATAAGGATACTAAATCCTATTTTCTGGTAAATTTTCATAATTTTTTGACACCTCTAGCTAGGTCATTTGACCTGATACAACATCGGATTT	3 (0.000057%)	
GGCATTCGTATTTCATAGTCAGAGGTGAAATTCTTGGATTTATGAAAGACGAACAACTGCGAAAGCATTTGCCAAGGATGTTTTCATTAATCAAGAACGA	4 (0.000076%)	
GGCCACGCTTTCACGGTTCGTATTCGTACTGAAAATCAGAATCAAACGAGCTTTTACCCTTTTGTTCCACACGAGATTTCTGTTCTCGTTGAGCTCATCT	38 (0.000719%)	
GGCCGCACGCGCGCTACACTGATGTATTCAACGAGTTCACACCTTGGCCGACAGGCCCGGGTAATCTTTGAAATTTCATCGTGATGGGGATAGATCGATC	5 (0.000095%)	
GGCCGTCGGGGCAAGAGCCAGGCCTCGATGAGTAGGAGGGCGCGGCGGTCGCTGCAAAACCTAGGGCGCGAGCCCGGGCGGAGCGGCCGTCGGTGCAGAT	16 (0.000303%)	
GGCCTACCATGGTGGTAACGGGTGACGGAGAATTAGGGTTCGATTCCGGAGAGGGAGCCTGAGAAACGGCTACCACATCCAAGGAAGGCAGCAGGCGCGC	20 (0.000378%)	
GGCGAATGCCAGCCGTTCGTTTGCATGTTCCTTGACACTTTTCGTGCCGGGGTTTTGTGATATCCGGAAGCAACGCGCACGACAAGACCGAGATAAAAGC	48 (0.000908%)	
GGCGAGACAAGGGTTCACATTTCGTTCATCACCCTTGGCCGGCTATCGAACAGCCGGACTCCCATCAAAAGATGGTTGCCAAGAACATCTTCGTTACGGT	18 (0.000341%)	
GGCGAGACAAGGGTTCACATTTCGTTCATCACCCTTGGCCGGCTTTCGAACAGCCGGACTCCCATCAAAAGATGGTTGCCAAGAACATCTTCGTTACGGT	17 (0.000322%)	
GGCGGACCGGATTGCTCCGTTCCGCATCCGACCAGGACGCATCGCCGGCCCCCATCCGCTTCCCTCCCGACAATTTCAAGCACTCTTTGACTCTCTTTTC	23 (0.000435%)	

GGCGGCACATCTGTTAAAAGATAACGCAGGTGTCCTAAGATGAGCTCAACGAGAACAGAAATCTCGTGTGGAACAAAAGGGTAAAAGCTCGTTTGATTCT	5 (0.000095%)
GGCGTAAGAATTGTATCCTTGTTAGAAGACACAAAGCCAAAGACTCATATGGACTTTGGCTACACCATGAAAGCTTTGAGAAGCAAGAAGAAGGTTGGTT	11 (0.000208%)
GGCGTGCCTCGGCATCAGCGTGCTCCGGGCGTCGGCCTGTGGGCTCCCCATTCGACCCGTCTTGAAACACGGACCAAGGAGTCTGACATGTGTGCGAGTC	17 (0.000322%)
GGCTCATTAAATCAGTTATAGTTTGTTTGATGGTAACTACTACTCGGATAACCGTAGTAATTCTAGAGCTAATACGTGCAACAAACCCCGACTTATGGAA	10 (0.000189%)
GGCTGGGGCGGCACATCTGTTAAAAGATAACGCAGGTGTCCTAAGATGAGCTCAACGAGAACAGAAATCTCGTGTGGAACAAAAGGGTAAAAGCTCGTTT	6 (0.000114%)
GGCTGTCCCGAGTGTGAGCGAGGTGTGAGTGTCGCCCATGGGCATCGACACCTTGCGGCTAGGAACTGGAACGAGACGGGTAGCAAAGATTTCGAGTAGC	18 (0.000341%)
GGCTGTCCCGAGTGTGAGCGAGGTGTGAGTGTCGCCCATGGGCATCGACACCTTGCGGCTAGGAACTGGAACGAGACGGGTGGCAAAGATTTCGAGTAGC	20 (0.000378%)
GGCTTGCTTTGAGCACTCTAATTTCTTCAAAGTAACAGCGCCGGAGGCACGACCCGGCCAATTAAGACCAGGAGCGTATCGCCGACCGAAGGGACAAGCC	7 (0.000132%)
GGGAATCCTTGTTAGTTTCTTTTCCTCCGCTTATTGATATGCTTAAACTCAGCGGGTAATCCCGCCTGACCTGGGGTCGCTATATGGACTTTGGGTCATC	61 (0.001154%)
GGGACAGTCGGGGGCATTCGTATTTCATAGTCAGAGGTGAAATTCTTGGATTTATGAAAGACGAACAACTGCGAAAGCATTTGCCAAGGATGTTTTCATT	33 (0.000624%)
GGGAGGCGAATGCCAGCCGTTCGTTTGCATGTTCCTTGACACTTTTCGTGCCGGGGTTTTGTGATATCCGGAAGCAACGCGCACGACAAGACCGAGATAA	14 (0.000265%)
GGGAGTTTGGCTGGGGCGGCACATCTGTTAAAAGATAACGCAGGTGTCCTAAGATGAGCTCAACGAGAACAGAAATCTCGTGTGGAACAAAAGGGTAAAA	42 (0.000795%)
GGGATTTAGATTGTACTCATTCCAATTACCAGACTCGAAAGAGCCCGGTATTGTTATTTATTGTCACTACCTCCCCGTGTCAGGATTGGGTAATTTGCGC	42 (0.000795%)
GGGCAAGTCTGGTGCCAGCAGCCGCGGTAATTCCAGCTCCAATAGCGTATATTTAAGTTGTTGCAGTTAAAAAGCTCGTAGTTGAACCTTGGGATGGGTC	161 (0.003046%)
GGGCAGAGCCCGCGTCGACCTTTTATCTAATAAATGCGTCCCTTCCATAAGTCGGGGTTTGTTGCACGTATTAGCTCTAGAATTACTACGGTTATCCGAG	49 (0.000927%)
GGGCATCGACACCTTGCGGCTAGGAACTGGAACGAGACGGGTGGCAAAGATTTCGAGTAGCACTTCATACTACCGTGGGTTTTTTAAACCTTCCGAGTTT	12 (0.000227%)
GGGCATTCGTATTTCATAGTCAGAGGTGAAATTCTTGGATTTATGAAAGACGAACAACTGCGAAAGCATTTGCCAAGGATGTTTTCATTAATCAAGAACG	35 (0.000662%)
GGGCCGCACGCGCGCTACACTGATGTATTCAACGAGTTCACACCTTGGCCGACAGGCCCGGGTAATCTTTGAAATTTCATCGTGATGGGGATAGATCGAT	7 (0.000132%)
GGGCGGCACATCTGTTAAAAGATAACGCAGGTGTCCTAAGATGAGCTCAACGAGAACAGAAATCTCGTGTGGAACAAAAGGGTAAAAGCTCGTTTGATTC	5 (0.000095%)
GGGCGGTGTGTACAAAGGGCAGGGACGTAGTCAACGCGAGCTGATGACTCGCGCTTACTAGGAATTCCTCGTTGAAGACCAACAATTGCAATGATCGATC	32 (0.000605%)
GGGCGTCGGCCTGTGGGCTCCCCATTCGACCCGTCTTGAAACACGGACCAAGGAGTCTGACATGTGTGCGAGTCAACGGGTGAGTAAACCCGTAAGGCGC	7 (0.000132%)
GGGGAGGCGAATGCCAGCCGTTCGTTTGCATGTTCCTTGACACTTTTCGTGCCGGGGTTTTGTGATATCCGGAAGCAACGCGCACGACAAGACCGAGATA	33 (0.000624%)
GGGGAGTTTGGCTGGGGCGGCACATCTGTTAAAAGATAACGCAGGTGTCCTAAGATGAGCTCAACGAGAACAGAAATCTCGTGTGGAACAAAAGGGTAAA	33 (0.000624%)
GGGGCATTCGTATTTCATAGTCAGAGGTGAAATTCTTGGATTTATGAAAGACGAACAACTGCGAAAGCATTTGCCAAGGATGTTTTCATTAATCAAGAAC	37 (0.000700%)
GGGGCGGCACATCTGTTAAAAGATAACGCAGGTGTCCTAAGATGAGCTCAACGAGAACAGAAATCTCGTGTGGAACAAAAGGGTAAAAGCTCGTTTGATT	5 (0.000095%)
GGGGGCATTCGTATTTCATAGTCAGAGGTGAAATTCTTGGATTTATGAAAGACGAACAACTGCGAAAGCATTTGCCAAGGATGTTTTCATTAATCAAGAA	18 (0.000341%)
GGGTGACGGAGAATTAGGGTTCGATTCCGGAGAGGGAGCCTGAGAAACGGCTACCACATCCAAGGAAGGCAGCAGGCGCGCAAATTACCCAATCCTGACA	17 (0.000322%)
GGGTGGTTCGCCGCCCGCGACGTCGCGAGAAGTCCACTAAACCTTATCATTTAGAGGAAGGAGAAGTCGTAACAAGGTTTCCGTAGGTGAACCTGCGGAA	189 (0.003576%)
GGGTTGCGGTTTAAGTTGTTATACTCAATCATACACATGA	465 (0.003519%)
GGGTTTACTCACCCGTTGACTCGCACACATGTCAGACTCCTTGGTCCGTGTTTCAAGACGGGTCGAATGGGGAGCCCACAGGCCGACGCCCGGAGCACGC	6 (0.000114%)
GGTAAATTTTCATAATTTTTTGACACCTCTAGCTAGGTCATTTGACCTGATACAACATCGGATTTTCATGGTCTAGTTGGGGCTCCGTGGGCATATTTGA	5 (0.000095%)
GGTAACGGGTGACGGAGAATTAGGGTTCGATTCCGGAGAGGGAGCCTGAGAAACGGCTACCACATCCAAGGAAGGCAGCAGGCGCGCAAATTACCCAATC	83 (0.001570%)
GGTAATTCCAGCTCCAATAGCGTATATTTAAGTTGTTGCAGTTAAAAAGCTCGTAGTTGAACCTTGGGATGGGTCGGCCGGTCCGCCTTTGGTGTGCATT	99 (0.001873%)
GGTAGGAGCGACGGGCGGTGTGTACAAAGGGCAGGGACGTAGTCAACGCGAGCTGATGACTCGCGCTTACTAGGAATTCCTCGTTGAAGACCAACAATTG	41 (0.000776%)
GGTCTGTGATGCCCTTAGATGTTCTGGGCCGCACGCGCGCTACACTGATGTATTCAACGAGTTCACACCTTGGCCGACAGGCCCGGGTAATCTTTGAAAT	13 (0.000246%)
GGTCTTCAACGAGGAATTCCTAGTAAGCGCGAGTCATCAGCTCGCGTTGACTACGTCCCTGCCCTTTGTACACACCGCCCGTCGCTCCTACCGATTGAAT	7 (0.000132%)
GGTGCCAGCAGCCGCGGTAATTCCAGCTCCAATAGCGTATATTTAAGTTGTTGCAGTTAAAAAGCTCGTAGTTGAACCTTGGGATGGGTCGGCCGGTCCG	12 (0.000227%)
GGTGGGGAGTTTGGCTGGGGCGGCACATCTGTTAAAAGATAACGCAGGTGTCCTAAGATGAGCTCAACGAGAACAGAAATCTCGTGTGGAACAAAAGGGT	5 (0.000095%)
GGTGGTTCGCCGCCCGCGACGTCGCGAGAAGTCCACTAAACCTTATCATTTAGAGGAAGGAGAAGTCGTAACAAGGTTTCCGTAGGTGAACCTGCGGAAG	28 (0.000530%)
GGTGTGAGTGTCGCCCATGGGCATCGACACCTTGCGGCTAGGAACTGGAACGAGACGGGTAGCAAAGATTTCGAGTAGCACTTCATACTACCGTGGGTTT	3 (0.000057%)
GGTGTGAGTGTCGCCCATGGGCATCGACACCTTGCGGCTAGGAACTGGAACGAGACGGGTGGCAAAGATTTCGAGTAGCACTTCATACTACCGTGGGTTT	9 (0.000170%)
GGTTCACATTTCGTTCATCACCCTTGGCCGGCTATCGAACAGCCGGACTCCCATCAAAAGATGGTTGCCAAGAACATCTTCGTTACGGTTTGCTAATTCT	4 (0.000076%)
GGTTCACATTTCGTTCATCACCCTTGGCCGGCTTTCGAACAGCCGGACTCCCATCAAAAGATGGTTGCCAAGAACATCTTCGTTACGGTTTGCTAATTCT	3 (0.000057%)

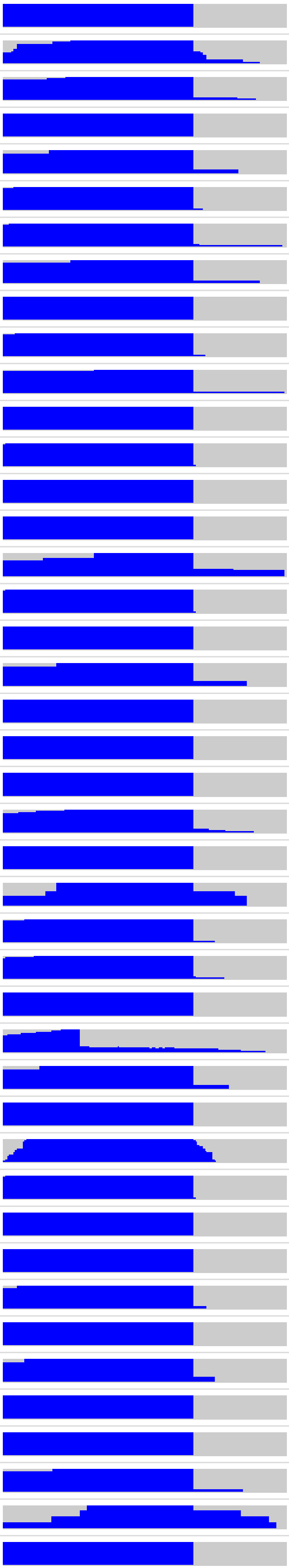

GGTTCGCCGCCCGCGACGTCGCGAGAAGTCCACTAAACCTTATCATTTAGAGGAAGGAGAAGTCGTAACAAGGTTTCCGTAGGTGAACCTGCGGAAGGAT	14 (0.000265%)
GGTTCGTATTCGTACTGAAAATCAGAATCAAACGAGCTTTTACCCTTTTGTTCCACACGAGATTTCTGTTCTCGTTGAGCTCATCTTAGGACACCTGCGT	9 (0.000170%)
GGTTGCGGTTTAAGTTCTTATACTCAATCATACACATGAC	9684 (0.073282%)
GGTTGCGGTTTAAGTTGTTATACTCAATCATACACATGAC	963 (0.007287%)
GGTTTAAGTTCTTATACTCAATCATACACATGACATCAAG	1531 (0.011586%)
GGTTTAAGTTGTTATACTCAATCATACACATGACAACAAGTCATATTCGACTCCAAAACACTAACCAACCTTCTTCTTGCTTCTCAAAGCTTTCATGGTG	112 (0.002119%)
GGTTTAAGTTGTTATACTCAATCATACACATGACATCAAG	2183 (0.016519%)
GGTTTACTCACCCGTTGACTCGCACACATGTCAGACTCCTTGGTCCGTGTTTCAAGACGGGTCGAATGGGGAGCCCACAGGCCGACGCCCGGAGCACGCT	17 (0.000322%)
GTAAATTTTCATAATTTTTTGACACCTCTAGCTAGGTCATTTGACCTGATACAACATCGGATTTTCATGGTCTAGTTGGGGCTCCGTGGGCATATTTGAT	16 (0.000303%)
GTAACGGGTGACGGAGAATTAGGGTTCGATTCCGGAGAGGGAGCCTGAGAAACGGCTACCACATCCAAGGAAGGCAGCAGGCGCGCAAATTACCCAATCC	18 (0.000341%)
GTAACTTCGGGAAAAGGATTGGCTCTGAGGGCTGGGCTCGGGGGTCCCAGTTCCGAACCCGTCGGCTGTCAGCGGACTGCTCGAGCTGCTTCCGCGGCGA	12 (0.000227%)
GTAAGAATTGTATCCTTGTTAGAAGACACAAAGCCAAAGACTCATATGGACTTTGGCTACACCATGAAAGCTTTGAGAAGCAAGAAGAAGGTTGGTTAGT	60 (0.001135%)
GTAAGTATGAACGAATTCAGACTGTGAAACTGCGAATGGCTCATTAAATCAGTTATAGTTTGTTTGATGGTAACTACTACTCGGATAACCGTAGTAATTC	23 (0.000435%)
GTAATTCCAGCTCCAATAGCGTATATTTAAGTTGTTGCAGTTAAAAAGCTCGTAGTTGAACCTTGGGATGGGTCGGCCGGTCCGCCTTTGGTGTGCATTG	13 (0.000246%)
GTACGCTCCAGGCGTCCTTGGCTCGGATTTAGGCCAACCGCGTGCGGTAACACACGGGAGACCAGCTTCCGTCCCGCATCAGCAAAGGATGGTGAGGGAC	16 (0.000303%)
GTACTCATTCCAATTACCAGACTCGAAAGAGCCCGGTATTGTTATTTATTGTCACTACCTCCCCGTGTCAGGATTGGGTAATTTGCGCGCCTGCTGCCTT	5 (0.000095%)
GTACTGAAAATCAGAATCAAACGAGCTTTTACCCTTTTGTTCCACACGAGATTTCTGTTCTCGTTGAGCTCATCTTAGGACACCTGCGTTATCTTTTAAC	7 (0.000132%)
GTAGGAGCGACGGGCGGTGTGTACAAAGGGCAGGGACGTAGTCAACGCGAGCTGATGACTCGCGCTTACTAGGAATTCCTCGTTGAAGACCAACAATTGC	118 (0.002232%)
GTAGGCTTGCTTTGAGCACTCTAATTTCTTCAAAGTAACAGCGCCGGAGGCACGACCCGGCCAATTAAGACCAGGAGCGTATCGCCGACCGAAGGGACAA	26 (0.000492%)
GTAGTCATATGCTTGTCTCAAAGATTAAGCCATGCATGTGTAAGTATGAACGAATTCAGACTGTGAAACTGCGAATGGCTCATTAAATCAGTTATAGTTT	102 (0.001930%)
GTAGTGTTTCCTTGTTAGAAGACACAAAGCCAAAGACTCATATGGACTTTGGCTACACCATGAAAGCTTTGAGAAGCAAGAAGAAGGTTGGTTAGTGTTT	15 (0.000284%)
GTATCCAGAGCGTAGGCTTGCTTTGAGCACTCTAATTTCTTCAAAGTAACAGCGCCGGAGGCACGACCCGGCCAATTAAGACCAGGAGCGTATCGCCGAC	17 (0.000322%)
GTATCCTTGTTAGAAGACACAAAGCCAAAGACTCATATGGACTTTGGCTACACCATGAAAGCTTTGAGAAGCAAGAAGAAGGTTGGTTAGTGTTTTGGAG	23 (0.000435%)
GTATGAACGAATTCAGACTGTGAAACTGCGAATGGCTCATTAAATCAGTTATAGTTTGTTTGATGGTAACTACTACTCGGATAACCGTAGTAATTCTAGA	12 (0.000227%)
GTATTTCATAGTCAGAGGTGAAATTCTTGGATTTATGAAAGACGAACAACTGCGAAAGCATTTGCCAAGGATGTTTTCATTAATCAAGAACGAAAGTTGG	6 (0.000114%)
GTCAGAGGTGAAATTCTTGGATTTATGAAAGACGAACAACTGCGAAAGCATTTGCCAAGGATGTTTTCATTAATCAAGAACGAAAGTTGGGGGCTCGAAG	20 (0.000378%)
GTCAGGTGGGGAGTTTGGCTGGGGCGGCACATCTGTTAAAAGATAACGCAGGTGTCCTAAGATGAGCTCAACGAGAACAGAAATCTCGTGTGGAACAAAA	8 (0.000151%)
GTCATATGCTTGTCTCAAAGATTAAGCCATGCATGTGTAAGTATGAACGAATTCAGACTGTGAAACTGCGAATGGCTCATTAAATCAGTTATAGTTTGTT	72 (0.001362%)
GTCATATTCGACTCCAAAACACTAACCAACCTTCTTCTTGCTTCTCAAAGCTTTCATGGTGTAGCCAAAGTCCATATGAGTCTTTGGCTTTGTGTCTTCT	245 (0.004635%)
GTCCATATGAGTCTTTGGCTTTGTGTCTTCTAACAAGGAT	7701 (0.058276%)
GTCCCGAGTGTGAGCGAGGTGTGAGTGTCGCCCATGGGCATCGACACCTTGCGGCTAGGAACTGGAACGAGACGGGTAGCAAAGATTTCGAGTAGCACTT	15 (0.000284%)
GTCCCGAGTGTGAGCGAGGTGTGAGTGTCGCCCATGGGCATCGACACCTTGCGGCTAGGAACTGGAACGAGACGGGTGGCAAAGATTTCGAGTAGCACTT	20 (0.000378%)
GTCCCTCACCATCCTTTGCTGATGCGGGACGGAAGCTGGTCTCCCGTGTGTTACCGCACGCGGTTGGCCTAAATCCGAGCCAAGGACGCCTGGAGCGTAC	12 (0.000227%)
GTCCGACTTTGTGAAATGACTTGAGAGGTGTAGGATAAGTGGGAGCTTCGGCGCAAGTGAAATACCACTACTTTTAACGTTATTTTACTTACTCCGTGAA	19 (0.000359%)
GTCGAAATCCGCTAAGGAGTGTGTAACAACTCACCTGCCGAATCAACTAGCCCCGAAAATGGATGGCGCTTAAGCGCGCGACCTATACCCGGCCGTCGGG	75 (0.001419%)
GTCGACCTTTTATCTAATAAATGCGTCCCTTCCATAAGTCGGGGTTTGTTGCACGTATTAGCTCTAGAATTACTACGGTTATCCGAGTAGTAGTTACCAT	25 (0.000473%)
GTCGAGTTATCATGAATCATCAGAGCAACGGGCAGAGCCCGCGTCGACCTTTTATCTAATAAATGCGTCCCTTCCATAAGTCGGGGTTTGTTGCACGTAT	463 (0.008759%)
GTCGCCCATGGGCATCGACACCTTGCGGCTAGGAACTGGAACGAGACGGGTGGCAAAGATTTCGAGTAGCACTTCATACTACCGTGGGTTTTTAAACCT	30 (0.000568%)
GTCGGCCTGTGGGCTCCCCATTCGACCCGTCTTGAAACACGGACCAAGGAGTCTGACATGTGTGCGAGTCAACGGGTGAGTAAACCCGTAAGGCGCAAGG	25 (0.000473%)
GTCGGGGGCATTCGTATTTCATAGTCAGAGGTGAAATTCTTGGATTTATGAAAGACGAACAACTGCGAAAGCATTTGCCAAGGATGTTTTCATTAATCAA	8 (0.000151%)
GTCGGTACGCTCCAGGCGTCCTTGGCTCGGATTTAGGCCAACCGCGTGCGGTAACACACGGGAGACCAGCTTCCGTCCCGCATCAGCAAAGGATGGTGAG	10 (0.000189%)
GTCGTCCCTCACCATCCTTTGCTGATGCGGGACGGAAGCTGGTCTCCCGTGTGTTACCGCACGCGGTTGGCCTAAATCCGAGCCAAGGACGCCTGGAGCG	721 (0.013640%)
GTCTCAAAGATTAAGCCATGCATGTGTAAGTATGAACGAATTCAGACTGTGAAACTGCGAATGGCTCATTAAATCAGTTATAGTTTGTTTGATGGTAACT	3 (0.000057%)

GTCTCCGGGGAGGCGAATGCCAGCCGTTCGTTTGCATGTTCCTTGACACTTTTCGTGCCGGGGTTTTGTGA TATCCGGAAGCAACGCGCACGACAAGACC	9 (0.000170%)
GTCTGAACTCCAGTCACGAGATTCCATCTCGTATGCCGTC	10 (0.000076%)
GTCTGGTGCCAGCAGCCGCGGTAATTCCAGCTCCAATAGCGTATATTTAAGTTGTTGCAGTTAAAAAGCTC GTAGTTGAACCTTGGGATGGGTCGGCCGG	28 (0.000530%)
GTCTGTGATGCCCTTAGATGTTCTGGGCCGCACGCGCGCTACACTGATGTATTCAACGAGTTCACACCTTG GCCGACAGGCCCGGGTAATCTTTGAAATT	19 (0.000359%)
GTCTTCAACGAGGAATTCCTAGTAAGCGCGAGTCATCAGCTCGCGTTGACTACGTCCCTGCCCTTTGTACA CACCGCCCGTCGCTCCTACCGATTGAATG	15 (0.000284%)
GTGACGGAGAATTAGGGTTCGATTCCGGAGAGGGAGCCTGAGAAACGGCTACCACATCCAAGGAAGGCAGC AGGCGCGCAAATTACCCAATCCTGACACG	10 (0.000189%)
GTGAGCGAGGTGTGAGTGTCGCCCATGGGCATCGACACCTTGCGGCTAGGAACTGGAACGAGACGGGTAGC AAAGATTTCGAGTAGCACTTCATACTACC	13 (0.000246%)
GTGAGCGAGGTGTGAGTGTCGCCCATGGGCATCGACACCTTGCGGCTAGGAACTGGAACGAGACGGGTGGC AAAGATTTCGAGTAGCACTTCATACTACC	20 (0.000378%)
GTGAGTGTCGCCCATGGGCATCGACACCTTGCGGCTAGGAACTGGAACGAGACGGGTAGCAAAGATTTCGA GTAGCACTTCATACTACCGTGGGTTTTTT	25 (0.000473%)
GTGAGTGTCGCCCATGGGCATCGACACCTTGCGGCTAGGAACTGGAACGAGACGGGTGGCAAAGATTTCGA GTAGCACTTCATACTACCGTGGGTTTTTT	38 (0.000719%)
GTGATGCCCTTAGATGTTCTGGGCCGCACGCGCGCTACACTGATGTATTCAACGAGTTCACACCTTGGCCG ACAGGCCCGGGTAATCTTTGAAATTTCAT	19 (0.000359%)
GTGCCAGCAGCCGCGGTAATTCCAGCTCCAATAGCGTATATTTAAGTTGTTGCAGTTAAAAAGCTCGTAGT TGAACCTTGGGATGGGTCGGCCGGTCCGC	24 (0.000454%)
GTGCCTCGGCATCAGCGTGCTCCGGGCGTCGGCCTGTGGGCTCCCCATTCGACCCGTCTTGAAACACGGAC CAAGGAGTCTGACATGTGTGCGAGTCAAC	10 (0.000189%)
GTGCTCCGGGCGTCGGCCTGTGGGCTCCCCATTCGACCCGTCTTGAAACACGGACCAAGGAGTCTGACATG TGTGCGAGTCAACGGGTGAGTAAACCCGT	12 (0.000227%)
GTGGCCTACCATGGTGGTAACGGGTGACGGAGAATTAGGGTTCGATTCCGGAGAGGGAGCCTGAGAAACGG CTACCACATCCAAGGAAGGCAGCAGGCGC	18 (0.000341%)
GTGGGGAGTTTGGCTGGGGCGGCACATCTGTTAAAAGATAACGCAGGTGTCCTAAGATGAGCTCAACGAGA ACAGAAATCTCGTGTGGAACAAAAGGGTA	4 (0.000076%)
GTGGGTGGTTCGCCGCCCGCGACGTCGCGAGAAGTCCACTAAACCTTATCATTTAGAGGAAGGAGAAGTCG TAACAAGGTTTCCGTAGGTGAACCTGCGG	67 (0.001268%)
GTGGTTCGCCGCCCGCGACGTCGCGAGAAGTCCACTAAACCTTATCATTTAGAGGAAGGAGAAGTCGTAAC AAGGTTTCCGTAGGTGAACCTGCGGAAGG	23 (0.000435%)
GTGTAAGTATGAACGAATTCAGACTGTGAAACTGCGAATGGCTCATTAAATCAGTTATAGTTTGTTTGATG GTAACTACTACTCGGATAACCGTAGTAAT	12 (0.000227%)
GTGTATCCTTGTTAGAAGACACAAAGCCAAAGACTCATATGGACTTTGGCTACACCATGAAAGCTTTGAGA AGCAAGAAGAAGGTTGGTTAGTGTTTTGG	7 (0.000132%)
GTGTCGCCCATGGGCATCGACACCTTGCGGCTAGGAACTGGAACGAGACGGGTGGCAAAGATTTCGAGTAG CACTTCATACTACCGTGGGTTTTTTAAAC	10 (0.000189%)
GTGTGAGCGAGGTGTGAGTGTCGCCCATGGGCATCGACACCTTGCGGCTAGGAACTGGAACGAGACGGGTA GCAAAGATTTCGAGTAGCACTTCATACTA	18 (0.000341%)
GTGTGAGCGAGGTGTGAGTGTCGCCCATGGGCATCGACACCTTGCGGCTAGGAACTGGAACGAGACGGGTG GCAAAGATTTCGAGTAGCACTTCATACTA	34 (0.000643%)
GTGTGAGTGTCGCCCATGGGCATCGACACCTTGCGGCTAGGAACTGGAACGAGACGGGTAGCAAAGATTTC GAGTAGCACTTCATACTACCGTGGGTTTT	15 (0.000284%)
GTGTGAGTGTCGCCCATGGGCATCGACACCTTGCGGCTAGGAACTGGAACGAGACGGGTGGCAAAGATTTC GAGTAGCACTTCATACTACCGTGGGTTTT	20 (0.000378%)
GTGTTTCCTTGTTAGAAGACACAAAGCCAAAGACTCATATGGACTTTGGCTACACCATGAAAGCTTTGAGA AGCAAGAAGAAGGTTGGTTAGTGTTTTGG	44 (0.000832%)
GTTAAAAGATAACGCAGGTGTCCTAAGATGAGCTCAACGAGAACAGAAATCTCGTGTGGAACAAAAGGGTA AAAGCTCGTTTGATTCTGATTTTCAGTAC	23 (0.000435%)
GTTAAGGGATTTAGATTGTACTCATTCCAATTACCAGACTCGAAAGAGCCCGGTATTGTTATTTATTGTCA CTACCTCCCCGTGTCAGGATTGGGTAATT	85 (0.001608%)
GTTAGAAGACACAAAGCCAAAGACTCATATGGACTTTGGCTACACCATGAAAGCTTTGAGAAGCAAGAAGA AGGTTGGTTAGTGTTTTGGAGTCGAATAT	84 (0.001589%)
GTTAGTTTCTTTTCCTCCGCTTATTGATATGCTTAAACTCAGCGGGTAATCCCGCCTGACCTGGGGTCGCT ATATGGACTTTGGGTCATCTACAGCTTCC	25 (0.000473%)
GTTATACTCAATCATACACATGACAACAAGTCATATTCGACTCCAAAACACTAACCAACCTTCTTCTTGCT TCTCAAAGCTTTCATGGTGTAGCCAAAGT	126 (0.002384%)
GTTATAGTTTGTTTGATGGTAACTACTACTCGGATAACCGTAGTAATTCTAGAGCTAATACGTGCAACAAA CCCCGACTTATGGAAGGGACGCATTTATT	18 (0.000341%)
GTTATCATGAATCATCAGAGCAACGGGCAGAGCCCGCGTCGACCTTTTATCTAATAAATGCGTCCCTTCCA TAAGTCGGGGTTTGTTGCACGTATTAGCT	277 (0.005240%)
GTTATCCCATGCTAATGTATCCAGAGCGTAGGCTTGCTTTGAGCACTCTAATTTCTTCAAAGTAACAGCGC CGGAGGCACGACCCGGCCAATTAAGACCA	58 (0.001097%)
GTTCACATTTCGTTCATCACCCTTGGCCGGCTATCGAACAGCCGGACTCCCATCAAAAGATGGTTGCCAAG AACATCTTCGTTACGGTTTGCTAATTCTC	9 (0.000170%)
GTTCACATTTCGTTCATCACCCTTGGCCGGCTTTCGAACAGCCGGACTCCCATCAAAAGATGGTTGCCAAG AACATCTTCGTTACGGTTTGCTAATTCTC	14 (0.000265%)
GTTCACCACCGCATGTCGGTACGCTCCAGGCGTCCTTGGCTCGGATTTAGGCCAACCGCGTGCGGTAACAC ACGGGAGACCAGCTTCCGTCCCGCATCAG	110 (0.002081%)
GTTCCATCGACCAGAGGCTGTTCACCTTGGAGACCTGATGCGGTTATGAGTACGACCGGGCGTGAGCGGCA CTCGGTCCTCCGGATTTTCAAGGGCCGCC	41 (0.000776%)
GTTCGCCGCCCGCGACGTCGCGAGAAGTCCACTAAACCTTATCATTTAGAGGAAGGAGAAGTCGTAACAAG GTTTCCGTAGGTGAACCTGCGGAAGGATC	35 (0.000662%)
GTTCGCTCGCCGTTACTAAGGGAATCCTTGTTAGTTTCTTTTCCTCCGCTTATTGATATGCTTAAACTCAG CGGGTAATCCCGCCTGACCTGGGGTCGCT	1065 (0.020148%)
GTTCGTATTCGTACTGAAAATCAGAATCAAACGAGCTTTTACCCTTTTGTTCCACACGAGATTTCTGTTCT CGTTGAGCTCATCTTAGGACACCTGCGTT	14 (0.000265%)
GTTCTGGGCCGCACGCGCGCTACACTGATGTATTCAACGAGTTCACACCTTGGCCGACAGGCCCGGGTAAT CTTTGAAATTTCATCGTGATGGGGATAGA	49 (0.000927%)
GTTCTTATACTCAATCATACACATGACATCAAGTCATATTCGACTCCAAAACACTAACCAACCTTCTTCTT GCTTCTCAAAGCTTTCATGGTGTAGCCAA	91 (0.001722%)

GTTGAGCTTGACTCTAGTCCGACTTTGTGAAATGACTTGAGAGGTGTAGGATAAGTGGGAGCTTCGGCGCA<br>AGTGAAATACCACTACTTTTAACGTTATT	36 (0.000681%)	
GTTGCGGTTTAAGTTCTTATACTCAATCATACACATGACA	2325 (0.017594%)	
GTTGCGGTTTAAGTTGTTATACTCAATCATACACATGACA	2552 (0.019312%)	
GTTGGTCTTCAACGAGGAATTCCTAGTAAGCGCGAGTCATCAGCTCGCGTTGACTACGTCCCTGCCCTTTG<br>TACACACCGCCCGTCGCTCCTACCGATTG	198 (0.003746%)	
GTTGTTATACTCAATCATACACATGACATCAAGTCATATT	255 (0.001930%)	
GTTTAAGTTCTTATACTCAATCATACACATGACATCAAGT	2182 (0.016512%)	
GTTTAAGTTGTTATACTCAATCATACACATGACAACAAGTCATATTCGACTCCAAAACACTAACCAACCTT<br>CTTCTTGCTTCTCAAAGCTTTCATGGTGT	133 (0.002516%)	
GTTTAAGTTGTTATACTCAATCATACACATGACATCAAGT	1021 (0.007726%)	
GTTTACTCACCCGTTGACTCGCACACATGTCAGACTCCTTGGTCCGTGTTTCAAGACGGGTCGAATGGGGA<br>GCCCACAGGCCGACGCCCGGAGCACGCTG	28 (0.000530%)	
GTTTCCTTGTTAGAAGACACAAAGCCAAAGACTCATATGGACTTTGGCTACACCATGAAAGCTTTGAGAAG<br>CAAGAAGAAGGTTGGTTAGTGTTTTGGAG	41 (0.000776%)	
GTTTCTTTTCCTCCGCTTATTGATATGCTTAAACTCAGCGGGTAATCCCGCCTGACCTGGGGTCGCTATAT<br>GGACTTTGGGTCATCTACAGCTTCCGGAC	21 (0.000397%)	
GTTTGATGGTAACTACTACTCGGATAACCGTAGTAATTCTAGAGCTAATACGTGCAACAAACCCCGACTTA<br>TGGAAGGGACGCATTTATTAGATAAAAGG	27 (0.000511%)	
GTTTGGCTGGGGCGGCACATCTGTTAAAAGATAACGCAGGTGTCCTAAGATGAGCTCAACGAGAACAGAAA<br>TCTCGTGTGGAACAAAAGGGTAAAAGCTC	45 (0.000851%)	
GTTTGTTTGATGGTAACTACTACTCGGATAACCGTAGTAATTCTAGAGCTAATACGTGCAACAAACCCCGA<br>CTTATGGAAGGGACGCATTTATTAGATAA	46 (0.000870%)	
TAAAAGATAACGCAGGTGTCCTAAGATGAGCTCAACGAGAACAGAAATCTCGTGTGGAACAAAAGGGTAAA<br>AGCTCGTTTGATTCTGATTTTCAGTACGA	17 (0.000322%)	
TAAAAGCCTAAGTAGTGTTTCCTTGTTAGAAGACACAAAGCCAAAGACTCATATGGACTTTGGCTACACCA<br>TGAAAGCTTTGAGAAGCAAGAAGAAGGTT	33 (0.000624%)	
TAAAGGCGTAAGAATTGTATCCTTGTTAAAAGACACAAAGCCAAAGACTCATATGGACTTTGGCTACACCA<br>TGAAAGCTTTGAGAAGCAAGAAGAAGGTT	75 (0.001419%)	
TAAAGGCGTAAGAATTGTATCCTTGTTAGAAGACACAAAGCCAAAGACTCATATGGACTTTGGCTACACCA<br>TGAAAGCTTTGAGAAGCAAGAAGAAGGTT	159 (0.003008%)	
TAAATCAGTTATAGTTTGTTTGATGGTAACTACTACTCGGATAACCGTAGTAATTCTAGAGCTAATACGTG<br>CAACAAACCCCGACTTATGGAAGGGACGC	9 (0.000170%)	
TAAATCCTATTTTCTGGTAAATTTTCATAATTTTTTGACACCTCTAGCTAGGTCATTTGACCTGATACAAC<br>ATCGGATTTTCATGGTCTAGTTGGGGCTC	5 (0.000095%)	
TAAATTTTCATAATTTTTTGACACCTCTAGCTAGGTCATTTGACCTGATACAACATCGGATTTTCATGGTC<br>TAGTTGGGGCTCCGTGGGCATATTTGATG	6 (0.000114%)	
TAACAGGGACAGTCGGGGGCATTCGTATTTCATAGTCAGAGGTGAAATTCTTGGATTTATGAAAGACGAAC<br>AACTGCGAAAGCATTTGCCAAGGATGTTT	47 (0.000889%)	
TAACAGGTCTGTGATGCCCTTAGATGTTCTGGGCCGCACGCGCGCTACACTGATGTATTCAACGAGTTCAC<br>ACCTTGGCCGACAGGCCCGGGTAATCTTT	50 (0.000946%)	
TAACGCCTCGAAGAACTAATGGCAGCCCACGCAAGGCAAGCCCATTCTCCTCGACGATTCAGCAGTTTTTG<br>TCCGAGAACTGCTGAGAAAACTCGGAAAA	6 (0.000114%)	
TAACGGCGTGCCTCGGCATCAGCGTGCTCCGGGCGTCGGCCTGTGGGCTCCCCATTCGACCCGTCTTGAAA<br>CACGGACCAAGGAGTCTGACATGTGTGCG	39 (0.000738%)	
TAACGGGTGACGGAGAATTAGGGTTCGATTCCGGAGAGGGAGCCTGAGAAACGGCTACCACATCCAAGGAA<br>GGCAGCAGGCGCGCAAATTACCCAATCCT	5 (0.000095%)	
TAAGAATTGTATCCTTGTTAGAAGACACAAAGCCAAAGACTCATATGGACTTTGGCTACACCATGAAAGCT<br>TTGAGAAGCAAGAAGAAGGTTGGTTAGTG	84 (0.001589%)	
TAAGCCATGCATGTGTAAGTATGAACGAATTCAGACTGTGAAACTGCGAATGGCTCATTAAATCAGTTATA<br>GTTTGTTTGATGGTAACTACTACTCGGAT	8 (0.000151%)	
TAAGCGCGCGACCTATACCCGGCCGTCGGGGCAAGAGCCAGGCCTCGATGAGTAGGAGGGCGCGGCGGTCG<br>CTGCAAAACCTAGGGCGCGAGCCCGGGCG	7 (0.000132%)	
TAAGGATACTAAATCCTATTTTCTGGTAAATTTTCATAATTTTTTGACACCTCTAGCTAGGTCATTTGACC<br>TGATACAACATCGGATTTTCATGGTCTAG	5 (0.000095%)	
TAAGGGATTTAGATTGTACTCATTCCAATTACCAGACTCGAAAGAGCCCGGTATTGTTATTTATTGTCACT<br>ACCTCCCCGTGTCAGGATTGGGTAATTTG	43 (0.000813%)	
TAAGTAGTGTTTCCTTGTTAGAAGACACAAAGCCAAAGACTCATATGGACTTTGGCTACACCATGAAAGCT<br>TTGAGAAGCAAGAAGAAGGTTGGTTAGTG	43 (0.000813%)	
TAAGTATGAACGAATTCAGACTGTGAAACTGCGAATGGCTCATTAAATCAGTTATAGTTTGTTTGATGGTA<br>ACTACTACTCGGATAACCGTAGTAATTCT	9 (0.000170%)	
TAAGTTCTTATACTCAATCATACACATGACATCAAGTCATATTCGACTCCAAAACACTAACCAACCTTCTT<br>CTTGCTTCTCAAAGCTTTCATGGTGTAGC	22 (0.000416%)	
TAAGTTGTTATACTCAATCATACACATGACATCAAGTCAT	81 (0.000613%)	
TAATGATTAACAGGGACAGTCGGGGGCATTCGTATTTCATAGTCAGAGGTGAAATTCTTGGATTTATGAAA<br>GACGAACAACTGCGAAAGCATTTGCCAAG	1489 (0.028169%)	
TAATGTATCCAGAGCGTAGGCTTGCTTTGAGCACTCTAATTTCTTCAAAGTAACAGCGCCGGAGGCACGAC<br>CCGGCCAATTAAGACCAGGAGCGTATCGC	5 (0.000095%)	
TAATTCCAGCTCCAATAGCGTATATTTAAGTTGTTGCAGTTAAAAAGCTCGTAGTTGAACCTTGGGATGGG<br>TCGGCCGGTCCGCCTTTGGTGTGCATTGG	7 (0.000132%)	
TAATTTTTTGACACCTCTAGCTAGGTCATTTGACCTGATACAACATCGGATTTTCATGGTCTAGTTGGGGC<br>TCCGTGGGCATATTTGATGCAAACTTGAC	9 (0.000170%)	
TACACATGACATCAAGTCATATTCGACTCCAAAACACTAACCAACCTTCTTCTTGCTTCTCAAAGCTTTCA<br>TGGTGTAGCCAAAGTCCATATGAGTCTTT	18 (0.000341%)	
TACACCATGAAAGCTTTGAGAAGCAAGAAGAAGGTTGGTTAGTGTTTTGGAGTCGAATATGACTTGATGTC<br>ATGTGTATGATTGAGTATAACAACTTAAA	135 (0.002554%)	
TACCAGACTCGAAAGAGCCCGGTATTGTTATTTATTGTCACTACCTCCCCGTGTCAGGATTGGGTAATTTG<br>CGCGCCTGCTGCCTTCCTTGGATGTGGTA	6 (0.000114%)	
TACCCGGCCGTCGGGGCAAGAGCCAGGCCTCGATGAGTAGGAGGGCGCGGCGGTCGCTGCAAAACCTAGGG<br>CGCGAGCCCGGGCGGAGCGGCCGTCGGTG	5 (0.000095%)	

TACGGGTTTACTCACCCGTTGACTCGCACACATGTCAGACTCCTTGGTCCGTGTTTCAAGACGGGTCGAATGGGGAGCCCACAGGCCGACGCCCGGAGCA	4 (0.000076%)
TACTCAATCATACACATGACAACAAGTCATATTCGACTCCAAAACACTAACCAACCTTCTTCTTGCTTCTCAAAGCTTTCATGGTGTAGCCAAAGTCCAT	20 (0.000378%)
TACTCAATCATACACATGACATCAAGTCATATTCGACTCCAAAACACTAACCAACCTTCTTCTTGCTTCTCAAAGCTTTCATGGTGTAGCCAAAGTCCAT	13 (0.000246%)
TAGAAGACACAAAGCCAAAGACTCATATGGACTTTGGCTACACCATGAAAGCTTTGAGAAGCAAGAAGAAGGTTGGTTAGTGTTTTGGAGTCGAATATGA	44 (0.000832%)
TAGATGTTCTGGGCCGCACGCGCGCTACACTGATGTATTCAACGAGTTCACACCTTGGCCGACAGGCCCGGGTAATCTTTGAAATTTCATCGTGATGGGG	18 (0.000341%)
TAGATTGTACTCATTCCAATTACCAGACTCGAAAGAGCCCGGTATTGTTATTTATTGTCACTACCTCCCCGTGTCAGGATTGGGTAATTTGCGCGCCTGC	5 (0.000095%)
TAGGAGCGACGGGCGGTGTGTACAAAGGGCAGGGACGTAGTCAACGCGAGCTGATGACTCGCGCTTACTAGGAATTCCTCGTTGAAGACCAACAATTGCA	12 (0.000227%)
TAGGCCACGCTTTCACGGTTCGTATTCGTACTGAAAATCAGAATCAAACGAGCTTTTACCCTTTTGTTCCACACGAGATTTCTGTTCTCGTTGAGCTCAT	78 (0.001476%)
TAGGCGAGACAAGGGTTCACATTTCGTTCATCACCCTTGGCCGGCTATCGAACAGCCGGACTCCCATCAAAAGATGGTTGCCAAGAACATCTTCGTTACG	5 (0.000095%)
TAGGCGAGACAAGGGTTCACATTTCGTTCATCACCCTTGGCCGGCTTTCGAACAGCCGGACTCCCATCAAAAGATGGTTGCCAAGAACATCTTCGTTACG	7 (0.000132%)
TAGGCTGTCCCGAGTGTGAGCGAGGTGTGAGTGTCGCCCATGGGCATCGACACCTTGCGGCTAGGAACTGGAACGAGACGGGTAGCAAAGATTTCGAGTA	18 (0.000341%)
TAGGCTGTCCCGAGTGTGAGCGAGGTGTGAGTGTCGCCCATGGGCATCGACACCTTGCGGCTAGGAACTGGAACGAGACGGGTGGCAAAGATTTCGAGTA	24 (0.000454%)
TAGGCTTGCTTTGAGCACTCTAATTTCTTCAAAGTAACAGCGCCGGAGGCACGACCCGGCCAATTAAGACCAGGAGCGTATCGCCGACCGAAGGGACAAG	3 (0.000057%)
TAGTCATATGCTTGTCTCAAAGATTAAGCCATGCATGTGTAAGTATGAACGAATTCAGACTGTGAAACTGCGAATGGCTCATTAAATCAGTTATAGTTTG	56 (0.001059%)
TAGTGGCCTACCATGGTGGTAACGGGTGACGGAGAATTAGGGTTCGATTCCGGAGAGGGAGCCTGAGAAACGGCTACCACATCCAAGGAAGGCAGCAGGC	9 (0.000170%)
TAGTGTTTCCTTGTTAGAAGACACAAAGCCAAAGACTCATATGGACTTTGGCTACACCATGAAAGCTTTGAGAAGCAAGAAGAAGGTTGGTTAGTGTTTT	8 (0.000151%)
TAGTTTCTTTTCCTCCGCTTATTGATATGCTTAAACTCAGCGGGTAATCCCGCCTGACCTGGGGTCGCTATATGGACTTTGGGTCATCTACAGCTTCCGG	9 (0.000170%)
TATACCCGGCCGTCGGGGCAAGAGCCAGGCCTCGATGAGTAGGAGGGCGCGGCGGTCGCTGCAAAACCTAGGGCGCGAGCCCGGGCGGAGCGGCCGTCGG	7 (0.000132%)
TATACTCAATCATACACATGACAACAAGTCATATTCGACTCCAAAACACTAACCAACCTTCTTCTTGCTTCTCAAAGCTTTCATGGTGTAGCCAAAGTCC	13 (0.000246%)
TATACTCAATCATACACATGACATCAAGTCATATTCGACTCCAAAACACTAACCAACCTTCTTCTTGCTTCTCAAAGCTTTCATGGTGTAGCCAAAGTCC	45 (0.000851%)
TATAGTTTGTTTGATGGTAACTACTACTCGGATAACCGTAGTAATTCTAGAGCTAATACGTGCAACAAACCCCGACTTATGGAAGGGACGCATTTATTAG	9 (0.000170%)
TATCATGAATCATCAGAGCAACGGGCAGAGCCCGCGTCGACCTTTTATCTAATAAATGCGTCCCTTCCATAAGTCGGGGTTTGTTGCACGTATTAGCTCT	80 (0.001513%)
TATCCAGAGCGTAGGCTTGCTTTGAGCACTCTAATTTCTTCAAAGTAACAGCGCCGGAGGCACGACCCGGCCAATTAAGACCAGGAGCGTATCGCCGACC	4 (0.000076%)
TATCCCATGCTAATGTATCCAGAGCGTAGGCTTGCTTTGAGCACTCTAATTTCTTCAAAGTAACAGCGCCGGAGGCACGACCCGGCCAATTAAGACCAGG	8 (0.000151%)
TATCCTTGTTAGAAGACACAAAGCCAAAGACTCATATGGACTTTGGCTACACCATGAAAGCTTTGAGAAGCAAGAAGAAGGTTGGTTAGTGTTTTGGAGT	42 (0.000795%)
TATCTAATAAATGCGTCCCTTCCATAAGTCGGGGTTTGTTGCACGTATTAGCTCTAGAATTACTACGGTTATCCGAGTAGTAGTTACCATCAAACAAACT	4 (0.000076%)
TATGACTTGATGTCATGTGTATGATTGAGTATAAGAACTT	322 (0.002437%)
TATGATGTTATCCCATGCTAATGTATCCAGAGCGTAGGCTTGCTTTGAGCACTCTAATTTCTTCAAAGTAACAGCGCCGGAGGCACGACCCGGCCAATTA	35 (0.000662%)
TATGCTTGTCTCAAAGATTAAGCCATGCATGTGTAAGTATGAACGAATTCAGACTGTGAAACTGCGAATGGCTCATTAAATCAGTTATAGTTTGTTTGAT	17 (0.000322%)
TATGGACTTTGGCTACACCATGAAAGCTTTGAGAAGCAAGAAGAAGGTTGGTTAGTGTTTTGGAGTCGAATATGACTTGATGTCATGTGTATGATTGAGT	16 (0.000303%)
TATTCGACTCCAAAACACTAACCAACCTTCTTCTTGCTTCTCAAAGCTTTCATGGTGTAGCCAAAGTCCATATGAGTCTTTGGCTTTGTGTCTTCTAACA	45 (0.000851%)
TATTCGTACTGAAAATCAGAATCAAACGAGCTTTTACCCTTTTGTTCCACACGAGATTTCTGTTCTCGTTGAGCTCATCTTAGGACACCTGCGTTATCTT	3 (0.000057%)
TCAAAGATTAAGCCATGCATGTGTAAGTATGAACGAATTCAGACTGTGAAACTGCGAATGGCTCATTAAATCAGTTATAGTTTGTTTGATGGTAACTACT	5 (0.000095%)
TCAAAGCTTTCATGGTGTAGCCAAAGTCCATATGAGTCTTTGGCTTTGTGTCTTCTAACAAGGAAACACTACTTAGGCTTATAAGATGCGGTTGCGGTTT	16 (0.000303%)
TCAACGAGGAATTCCTAGTAAGCGCGAGTCATCAGCTCGCGTTGACTACGTCCCTGCCCTTTGTACACACCGCCCGTCGCTCCTACCGATTGAATGATCG	3 (0.000057%)
TCAAGTCATATTCGACTCCAAAACACTAACCAACCTTCTTCTTGCTTCTCAAAGCTTTCATGGTGTAGCCAAAGTCCATATGAGTCTTTGGCTTTGTGTC	55 (0.001041%)
TCAATCATACACATGACAACAAGTCATATTCGACTCCAAAACACTAACCAACCTTCTTCTTGCTTCTCAAAGCTTTCATGGTGTAGCCAAAGTCCATATG	14 (0.000265%)
TCAATCATACACATGACATCAAGTCATATTCGACTCCAAAACACTAACCAACCTTCTTCTTGCTTCTCAAAGCTTTCATGGTGTAGCCAAAGTCCATATG	34 (0.000643%)
TCAATCGGTAGGAGCGACGGGCGGTGTGTACAAAGGGCAGGGACGTAGTCAACGCGAGCTGATGACTCGCGCTTACTAGGAATTCCTCGTTGAAGACCAA	49 (0.000927%)
TCACATTTCGTTCATCACCCTTGGCCGGCTATCGAACAGCCGGACTCCCATCAAAAGATGGTTGCCAAGAACATCTTCGTTACGGTTTGCTAATTCTCGG	11 (0.000208%)
TCACATTTCGTTCATCACCCTTGGCCGGCTTTCGAACAGCCGGACTCCCATCAAAAGATGGTTGCCAAGAACATCTTCGTTACGGTTTGCTAATTCTCGG	3 (0.000057%)
TCACCACCGCATGTCGGTACGCTCCAGGCGTCCTTGGCTCGGATTTAGGCCAACCGCGTGCGGTAACACACGGGAGACCAGCTTCCGTCCCGCATCAGCA	15 (0.000284%)
TCACCATCCTTTGCTGATGCGGGACGGAAGCTGGTCTCCCGTGTGTTACCGCACGCGGTTGGCCTAAATCCGAGCCAAGGACGCCTGGAGCGTACCGACA	8 (0.000151%)

TCAGAATCAAACGAGCTTTTACCCTTTTGTTCCACACGAGATTTCTGTTCTCGTTGAGCTCATCTTAGGACACCTGCGTTATCTTTTAACAGATGTGCCG	12 (0.000227%)
TCAGAGGTGAAATTCTTGGATTTATGAAAGACGAACAACTGCGAAAGCATTTGCCAAGGATGTTTTCATTAATCAAGAACGAAAGTTGGGGGCTCGAAGA	4 (0.000076%)
TCATAATTTTTTGACACCTCTAGCTAGGTCATTTGACCTGATACAACATCGGATTTTCATGGTCTAGTTGGGGCTCCGTGGGCATATTTGATGCAAACTT	3 (0.000057%)
TCATACACATGACATCAAGTCATATTCGACTCCAAAACACTAACCAACCTTCTTCTTGCTTCTCAAAGCTTTCATGGTGTAGCCAAAGTCCATATGAGTC	6 (0.000114%)
TCATAGTCAGAGGTGAAATTCTTGGATTTATGAAAGACGAACAACTGCGAAAGCATTTGCCAAGGATGTTTTCATTAATCAAGAACGAAAGTTGGGGGCT	12 (0.000227%)
TCATATGGACTTTGGCTACACCATGAAAGCTTTGAGAAGCAAGAAGAAGGTTGGTTAGTGTTTTGGAGTCGAATATGACTTGATGTCATGTGTATGATTG	33 (0.000624%)
TCATATTCGACTCCAAAACACTAACCAACCTTCTTCTTGCTTCTCAAAGCTTTCATGGTGTAGCCAAAGTCCATATGAGTCTTTGGCTTTGTGTCTTCTA	72 (0.001362%)
TCATCAGAGCAACGGGCAGAGCCCGCGTCGACCTTTTATCTAATAAATGCGTCCCTTCCATAAGTCGGGGTTTGTTGCACGTATTAGCTCTAGAATTACT	4 (0.000076%)
TCATGAATCATCAGAGCAACGGGCAGAGCCCGCGTCGACCTTTTATCTAATAAATGCGTCCCTTCCATAAGTCGGGGTTTGTTGCACGTATTAGCTCTAG	18 (0.000341%)
TCATGTGTATGATTGAGTATAAGAACTTAAACCGCAACCG	4662 (0.035279%)
TCCAACCTAGGCGAGACAAGGGTTCACATTTCGTTCATCACCCTTGGCCGGCTATCGAACAGCCGGACTCCCATCAAAAGATGGTTGCCAAGAACATCTT	3 (0.000057%)
TCCAACCTAGGCGAGACAAGGGTTCACATTTCGTTCATCACCCTTGGCCGGCTTTCGAACAGCCGGACTCCCATCAAAAGATGGTTGCCAAGAACATCTT	5 (0.000095%)
TCCAACGAAGCACGCCCATCCAACCTAGGCGAGACAAGGGTTCACATTTCGTTCATCACCCTTGGCCGGCTATCGAACAGCCGGACTCCCATCAAAAGAT	19 (0.000359%)
TCCAACGAAGCACGCCCATCCAACCTAGGCGAGACAAGGGTTCACATTTCGTTCATCACCCTTGGCCGGCTTTCGAACAGCCGGACTCCCATCAAAAGAT	19 (0.000359%)
TCCAATTACCAGACTCGAAAGAGCCCGGTATTGTTATTTATTGTCACTACCTCCCCGTGTCAGGATTGGGTAATTTGCGCGCCTGCTGCCTTCCTTGGAT	3 (0.000057%)
TCCAGAGCGTAGGCTTGCTTTGAGCACTCTAATTTCTTCAAAGTAACAGCGCCGGAGGCACGACCCGGCCAATTAAGACCAGGAGCGTATCGCCGACCGA	5 (0.000095%)
TCCATATGAGTCTTTGGCTTTGTGTCTTCTAACAAGGATA	265 (0.002005%)
TCCATCGACCAGAGGCTGTTCACCTTGGAGACCTGATGCGGTTATGAGTACGACCGGGCGTGAGCGGCACTCGGTCCTCCGGATTTTCAAGGGCCGCCGG	4 (0.000076%)
TCCATGCTTTCCAACGAAGCACGCCCATCCAACCTAGGCGAGACAAGGGTTCACATTTCGTTCATCACCCTTGGCCGGCTATCGAACAGCCGGACTCCCA	13 (0.000246%)
TCCATTGGAGGGCAAGTCTGGTGCCAGCAGCCGCGGTAATTCCAGCTCCAATAGCGTATATTTAAGTTGTTGCAGTTAAAAAGCTCGTAGTTGAACCTTG	4 (0.000076%)
TCCCATGCTAATGTATCCAGAGCGTAGGCTTGCTTTGAGCACTCTAATTTCTTCAAAGTAACAGCGCCGGAGGCACGACCCGGCCAATTAAGACCAGGAG	11 (0.000208%)
TCCCTCACCATCCTTTGCTGATGCGGGACGGAAGCTGGTCTCCCGTGTGTTACCGCACGCGGTTGGCCTAAATCCGAGCCAAGGACGCCTGGAGCGTACC	3 (0.000057%)
TCCCTTGCCTACATTGTTCCATCGACCAGAGGCTGTTCACCTTGGAGACCTGATGCGGTTATGAGTACGACCGGGCGTGAGCGGCACTCGGTCCTCCGGA	4 (0.000076%)
TCCGACTTTGTGAAATGACTTGAGAGGTGTAGGATAAGTGGGAGCTTCGGCGCAAGTGAAATACCACTACTTTTAACGTTATTTTACTTACTCCGTGAAT	5 (0.000095%)
TCCGCCTAGGCTGTCCCGAGTGTGAGCGAGGTGTGAGTGTCGCCCATGGGCATCGACACCTTGCGGCTAGGAACTGGAACGAGACGGGTAGCAAAGATTT	164 (0.003103%)
TCCGCCTAGGCTGTCCCGAGTGTGAGCGAGGTGTGAGTGTCGCCCATGGGCATCGACACCTTGCGGCTAGGAACTGGAACGAGACGGGTGGCAAAGATTT	289 (0.005467%)
TCCGCTTATTGATATGCTTAAACTCAGCGGGTAATCCCGCCTGACCTGGGGTCGCTATATGGACTTTGGGTCATCTACAGCTTCCGGACAAGAGCGACCG	3 (0.000057%)
TCCGGGCGTCGGCCTGTGGGCTCCCCATTCGACCCGTCTTGAAACACGGACCAAGGAGTCTGACATGTGTGCGAGTCAACGGGTGAGTAAACCCGTAAGG	3 (0.000057%)
TCCGGGGAGGCGAATGCCAGCCGTTCGTTTGCATGTTCCTTGACACTTTTCGTGCCGGGGTTTTGTGATATCCGGAAGCAACGCGCACGACAAGACCGAG	8 (0.000151%)
TCCGGTTAAAATTCCGGAACCGGGACGTGGCGGTTGACGGCAACGTTAGGGAGTCCGGAGACGTCGGCGGGGGCCTCGGGAAGAGTTATCTTTTCTGTTT	20 (0.000378%)
TCCGTCGAGTTATCATGAATCATCAGAGCAACGGGCAGAGCCCGCGTCGACCTTTTATCTAATAAATGCGTCCCTTCCATAAGTCGGGGTTTGTTGCACG	15 (0.000284%)
TCCGTCTCCGGGGAGGCGAATGCCAGCCGTTCGTTTGCATGTTCCTTGACACTTTTCGTGCCGGGGTTTTGTGATATCCGGAAGCAACGCGCACGACAAG	762 (0.014416%)
TCCTATGATGTTATCCCATGCTAATGTATCCAGAGCGTAGGCTTGCTTTGAGCACTCTAATTTCTTCAAAGTAACAGCGCCGGAGGCACGACCCGGCCAA	9 (0.000170%)
TCCTCGTTAAGGGATTTAGATTGTACTCATTCCAATTACCAGACTCGAAAGAGCCCGGTATTGTTATTTATTGTCACTACCTCCCCGTGTCAGGATTGGG	23 (0.000435%)
TCCTTGTTAGAAGACACAAAGCCAAAGACTCATATGGACTTTGGCTACACCATGAAAGCTTTGAGAAGCAAGAAGAAGGTTGGTTAGTGTTTTGGAGTCG	8 (0.000151%)
TCGAAAGAGCCCGGTATTGTTATTTATTGTCACTACCTCCCCGTGTCAGGATTGGGTAATTTGCGCGCCTGCTGCCTTCCTTGGATGTGGTAGCCGTTTC	8 (0.000151%)
TCGAAATCCGCTAAGGAGTGTGTAACAACTCACCTGCCGAATCAACTAGCCCCGAAAATGGATGGCGCTTAAGCGCGCGACCTATACCCGGCCGTCGGGG	58 (0.001097%)
TCGAAATCCTATGATGTTATCCCATGCTAATGTATCCAGAGCGTAGGCTTGCTTTGAGCACTCTAATTTCTTCAAAGTAACAGCGCCGGAGGCACGACCC	227 (0.004294%)
TCGAATATGACTTGATGTCATGTGTATGATTGAGTATAAG	9266 (0.070119%)
TCGACCTTTTATCTAATAAATGCGTCCCTTCCATAAGTCGGGGTTTGTTGCACGTATTAGCTCTAGAATTACTACGGTTATCCGAGTAGTAGTTACCATC	10 (0.000189%)
TCGACTCCAAAACACTAACCAACCTTCTTCTTGCTTCTCAAAGCTTTCATGGTGTAGCCAAAGTCCATATGAGTCTTTGGCTTTGTGTCTTCTAACAAGG	34 (0.000643%)
TCGAGTTATCATGAATCATCAGAGCAACGGGCAGAGCCCGCGTCGACCTTTTATCTAATAAATGCGTCCCTTCCATAAGTCGGGGTTTGTTGCACGTATT	63 (0.001192%)
TCGATCACGGCAATTCCCCGCCACATCCTCTCAAACGCAATGGAAAGAGAGAAAGGACGAGGTCTTGACCGTCATCTTTTGCCCGAAGGACGGATGAGCT	3 (0.000057%)

TCGATCCATGCTTTCCAACGAAGCACGCCCATCCAACCTAGGCGAGACAAGGGTTCACATTTCGTTCATCACCCTTGGCCGGCTATCGAACAGCCGGACT	6 (0.000114%)	
TCGATCCGGTTAAAATTCCGGAACCGGGACGTGGCGGTTGACGGCAACGTTAGGGAGTCCGGAGACGTCGGCGGGGGCCTCGGGAAGAGTTATCTTTTCT	5 (0.000095%)	
TCGATCGAAATCCTATGATGTTATCCCATGCTAATGTATCCAGAGCGTAGGCTTGCTTTGAGCACTCTAATTTCTTCAAAGTAACAGCGCCGGAGGCACG	3 (0.000057%)	
TCGATCGGGTTGCGGTTTAAGTTGTTATACTCAATCATAC	9 (0.000068%)	
TCGCCGCCCGCGACGTCGCGAGAAGTCCACTAAACCTTATCATTTAGAGGAAGGAGAAGTCGTAACAAGGTTTCCGTAGGTGAACCTGCGGAAGGATCGA	10 (0.000189%)	
TCGGAAGAGCACACGTCTGAACTCCAGTCACGAGATTCCA	6 (0.000045%)	
TCGGCATCAGCGTGCTCCGGGCGTCGGCCTGTGGGCTCCCCATTCGACCCGTCTTGAAACACGGACCAAGGAGTCTGACATGTGTGCGAGTCAACGGGTG	17 (0.000322%)	
TCGGCCTGTGGGCTCCCCATTCGACCCGTCTTGAAACACGGACCAAGGAGTCTGACATGTGTGCGAGTCAACGGGTGAGTAAACCCGTAAGGCGCAAGGA	15 (0.000284%)	
TCGGGTTGCGGTTTAAGTTGTTATACTCAATCATACACAT	144 (0.001090%)	
TCGGTACGCTCCAGGCGTCCTTGGCTCGGATTTAGGCCAACCGCGTGCGGTAACACACGGGAGACCAGCTTCCGTCCCGCATCAGCAAAGGATGGTGAGG	3 (0.000057%)	
TCGGTAGGAGCGACGGGCGGTGTGTACAAAGGGCAGGGACGTAGTCAACGCGAGCTGATGACTCGCGCTTACTAGGAATTCCTCGTTGAAGACCAACAAT	21 (0.000397%)	
TCGTACTGAAAATCAGAATCAAACGAGCTTTTACCCTTTTGTTCCACACGAGATTTCTGTTCTCGTTGAGCTCATCTTAGGACACCTGCGTTATCTTTTA	3 (0.000057%)	
TCGTATTCGTACTGAAAATCAGAATCAAACGAGCTTTTACCCTTTTGTTCCACACGAGATTTCTGTTCTCGTTGAGCTCATCTTAGGACACCTGCGTTAT	8 (0.000151%)	
TCGTCCCTCACCATCCTTTGCTGATGCGGGACGGAAGCTGGTCTCCCGTGTGTTACCGCACGCGGTTGGCCTAAATCCGAGCCAAGGACGCCTGGAGCGT	5 (0.000095%)	
TCGTTAAGGGATTTAGATTGTACTCATTCCAATTACCAGACTCGAAAGAGCCCGGTATTGTTATTTATTGTCACTACCTCCCCGTGTCAGGATTGGGTAA	8 (0.000151%)	
TCTAATAAATGCGTCCCTTCCATAAGTCGGGGTTTGTTGCACGTATTAGCTCTAGAATTACTACGGTTATCCGAGTAGTAGTTACCATCAAACAAACTAT	3 (0.000057%)	
TCTAGTCCGACTTTGTGAAATGACTTGAGAGGTGTAGGATAAGTGGGAGCTTCGGCGCAAGTGAAATACCACTACTTTTAACGTTATTTTACTTACTCCG	15 (0.000284%)	
TCTCAAACGCAATGGAAAGAGAGAAAGGACGAGGTCTTGACCGTCATCTTTTGCCCGAAGGACGGATGAGCTTTGGCGGGACTGAATCACTTCGAGTCAC	7 (0.000132%)	
TCTCAAAGCTTTCATGGTGTAGCCAAAGTCCATATGAGTCTTTGGCTTTGTGTCTTCTAACAAGGAAACACTACTTAGGCTTATAAGATGCGGTTGCGGT	4 (0.000076%)	
TCTCTCTCTCTCTCTCTCTCTCTCTCTCTCTCTCTCTCTC	80 (0.000605%)	
TCTGGGCCGCACGCGCGCTACACTGATGTATTCAACGAGTTCACACCTTGGCCGACAGGCCCGGGTAATCTTTGAAATTTCATCGTGATGGGGATAGATC	4 (0.000076%)	
TCTGGTAAATTTTCATAATTTTTTGACACCTCTAGCTAGGTCATTTGACCTGATACAACATCGGATTTTCATGGTCTAGTTGGGGCTCCGTGGGCATATT	3 (0.000057%)	
TCTGGTGCCAGCAGCCGCGGTAATTCCAGCTCCAATAGCGTATATTTAAGTTGTTGCAGTTAAAAAGCTCGTAGTTGAACCTTGGGATGGGTCGGCCGGT	16 (0.000303%)	
TCTGTGATGCCCTTAGATGTTCTGGGCCGCACGCGCGCTACACTGATGTATTCAACGAGTTCACACCTTGGCCGACAGGCCCGGGTAATCTTTGAAATTT	9 (0.000170%)	
TCTTAAAAGCCTAAGTAGTGTTTCCTTGTTAGAAGACACAAAGCCAAAGACTCATATGGACTTTGGCTACACCATGAAAGCTTTGAGAAGCAAGAAGAAG	37 (0.000700%)	
TCTTAAAGGCGTAAGAATTGTATCCTTGTTAAAAGACACAAAGCCAAAGACTCATATGGACTTTGGCTACACCATGAAAGCTTTGAGAAGCAAGAAGAAG	102 (0.001930%)	
TCTTAAAGGCGTAAGAATTGTATCCTTGTTAGAAGACACAAAGCCAAAGACTCATATGGACTTTGGCTACACCATGAAAGCTTTGAGAAGCAAGAAGAAG	74 (0.001400%)	
TCTTATACTCAATCATACACATGACATCAAGTCATATTCGACTCCAAAACACTAACCAACCTTCTTCTTGCTTCTCAAAGCTTTCATGGTGTAGCCAAAG	226 (0.004276%)	
TCTTCAACGAGGAATTCCTAGTAAGCGCGAGTCATCAGCTCGCGTTGACTACGTCCCTGCCCTTTGTACACACCGCCCGTCGCTCCTACCGATTGAATGA	14 (0.000265%)	
TCTTGCTTCTCAAAGCTTTCATGGTGTAGCCAAAGTCCATATGAGTCTTTGGCTTTGTGTCTTCTAACAAGGAAACACTACTTAGGCTTATAAGATGCGG	5 (0.000095%)	
TCTTTTCCTCCGCTTATTGATATGCTTAAACTCAGCGGGTAATCCCGCCTGACCTGGGGTCGCTATATGGACTTTGGGTCATCTACAGCTTCCGGACAAG	3 (0.000057%)	
TGAAAATCAGAATCAAACGAGCTTTTACCCTTTTGTTCCACACGAGATTTCTGTTCTCGTTGAGCTCATCTTAGGACACCTGCGTTATCTTTTAACAGAT	43 (0.000813%)	
TGAATCATCAGAGCAACGGGCAGAGCCCGCGTCGACCTTTTATCTAATAAATGCGTCCCTTCCATAAGTCGGGGTTTGTTGCACGTATTAGCTCTAGAAT	10 (0.000189%)	
TGACATCAAGTCATATTCGACTCCAAAACACTAACCAACCTTCTTCTTGCTTCTCAAAGCTTTCATGGTGTAGCCAAAGTCCATATGAGTCTTTGGCTTT	31 (0.000586%)	
TGACTCTAGTCCGACTTTGTGAAATGACTTGAGAGGTGTAGGATAAGTGGGAGCTTCGGCGCAAGTGAAATACCACTACTTTTAACGTTATTTTACTTAC	12 (0.000227%)	
TGACTTGATGTCATGTGTATGATTGAGTATAAGAACTTAA	81 (0.000613%)	
TGAGCGAGGTGTGAGTGTCGCCCATGGGCATCGACACCTTGCGGCTAGGAACTGGAACGAGACGGGTAGCAAAGATTTCGAGTAGCACTTCATACTACCG	4 (0.000076%)	
TGAGCGAGGTGTGAGTGTCGCCCATGGGCATCGACACCTTGCGGCTAGGAACTGGAACGAGACGGGTGGCAAAGATTTCGAGTAGCACTTCATACTACCG	5 (0.000095%)	
TGAGCTTGACTCTAGTCCGACTTTGTGAAATGACTTGAGAGGTGTAGGATAAGTGGGAGCTTCGGCGCAAGTGAAATACCACTACTTTTAACGTTATTTT	9 (0.000170%)	
TGAGTGTCGCCCATGGGCATCGACACCTTGCGGCTAGGAACTGGAACGAGACGGGTAGCAAAGATTTCGAGTAGCACTTCATACTACCGTGGGTTTTTTA	6 (0.000114%)	
TGAGTGTCGCCCATGGGCATCGACACCTTGCGGCTAGGAACTGGAACGAGACGGGTGGCAAAGATTTCGAGTAGCACTTCATACTACCGTGGGTTTTTTA	6 (0.000114%)	
TGATGCCCTTAGATGTTCTGGGCCGCACGCGCGCTACACTGATGTATTCAACGAGTTCACACCTTGGCCGACAGGCCCGGGTAATCTTTGAAATTTCATC	3 (0.000057%)	
TGATGCGGGACGGAAGCTGGTCTCCCGTGTGTTACCGCACGCGGTTGGCCTAAATCCGAGCCAAGGACGCCTGGAGCGTACCGACATGCGGTGGTGAACT	7 (0.000132%)	

TGATGGTAACTACTACTCGGATAACCGTAGTAATTCTAGAGCTAATACGTGCAACAAACCCCGACTTATGG AAGGGACGCATTTATTAGATAAAAGGTCG	4 (0.000076%)	
TGATGTCATGTGTATGATTGAGTATAAGAACTTAAACCGC	98 (0.000742%)	
TGATGTTATCCCATGCTAATGTATCCAGAGCGTAGGCTTGCTTTGAGCACTCTAATTTCTTCAAAGTAACA GCGCCGGAGGCACGACCCGGCCAATTAAG	28 (0.000530%)	
TGATTAACAGGGACAGTCGGGGGCATTCGTATTTCATAGTCAGAGGTGAAATTCTTGGATTTATGAAAGAC GAACAACTGCGAAAGCATTTGCCAAGGAT	113 (0.002138%)	
TGCAGCACGCGCCTAACGGCGTGCCTCGGCATCAGCGTGCTCCGGGCGTCGGCCTGTGGGCTCCCCATTCG ACCCGTCTTGAAACACGGACCAAGGAGTC	80 (0.001513%)	
TGCATGTGTAAGTATGAACGAATTCAGACTGTGAAACTGCGAATGGCTCATTAAATCAGTTATAGTTTGTT TGATGGTAACTACTACTCGGATAACCGTA	8 (0.000151%)	
TGCCAGCAGCCGCGGTAATTCCAGCTCCAATAGCGTATATTTAAGTTGTTGCAGTTAAAAAGCTCGTAGTT GAACCTTGGGATGGGTCGGCCGGTCCGCC	6 (0.000114%)	
TGCCAGTAGTCATATGCTTGTCTCAAAGATTAAGCCATGCATGTGTAAGTATGAACGAATTCAGACTGTGA AACTGCGAATGGCTCATTAAATCAGTTAT	125 (0.002365%)	
TGCCCTTAGATGTTCTGGGCCGCACGCGCGCTACACTGATGTATTCAACGAGTTCACACCTTGGCCGACAG GCCCGGGTAATCTTTGAAATTTCATCGTG	8 (0.000151%)	
TGCCGACTTCCCTTGCCTACATTGTTCCATCGACCAGAGGCTGTTCACCTTGGAGACCTGATGCGGTTATG AGTACGACCGGGCGTGAGCGGCACTCGGT	95 (0.001797%)	
TGCCTACATTGTTCCATCGACCAGAGGCTGTTCACCTTGGAGACCTGATGCGGTTATGAGTACGACCGGGC GTGAGCGGCACTCGGTCCTCCGGATTTTC	12 (0.000227%)	
TGCGAATGGCTCATTAAATCAGTTATAGTTTGTTTGATGGTAACTACTACTCGGATAACCGTAGTAATTCT AGAGCTAATACGTGCAACAAACCCCGACT	10 (0.000189%)	
TGCGGTTTAAGTTCTTATACTCAATCATACACATGACATC	3686 (0.027893%)	
TGCTAATGTATCCAGAGCGTAGGCTTGCTTTGAGCACTCTAATTTCTTCAAAGTAACAGCGCCGGAGGCAC GACCCGGCCAATTAAGACCAGGAGCGTAT	11 (0.000208%)	
TGCTCCGGGCGTCGGCCTGTGGGCTCCCCATTCGACCCGTCTTGAAACACGGACCAAGGAGTCTGACATGT GTGCGAGTCAACGGGTGAGTAAACCCGTA	5 (0.000095%)	
TGCTGATGCGGGACGGAAGCTGGTCTCCCGTGTGTTACCGCACGCGGTTGGCCTAAATCCGAGCCAAGGAC GCCTGGAGCGTACCGACATGCGGTGGTGA	3 (0.000057%)	
TGCTTCTCAAAGCTTTCATGGTGTAGCCAAAGTCCATATGAGTCTTTGGCTTTGTGTCTTCTAACAAGGAA ACACTACTTAGGCTTATAAGATGCGGTTG	3 (0.000057%)	
TGCTTGTCTCAAAGATTAAGCCATGCATGTGTAAGTATGAACGAATTCAGACTGTGAAACTGCGAATGGCT CATTAAATCAGTTATAGTTTGTTTGATGG	5 (0.000095%)	
TGCTTTCCAACGAAGCACGCCCATCCAACCTAGGCGAGACAAGGGTTCACATTTCGTTCATCACCCTTGGC CGGCTATCGAACAGCCGGACTCCCATCAA	17 (0.000322%)	
TGGAAAGAGAGAAAGGACGAGGTCTTGACCGTCATCTTTTGCCCGAAGGACGGATGAGCTTTGGCGGGACT GAATCACTTCGAGTCACCGTCGACAACTT	10 (0.000189%)	
TGGAAGTCGAAATCCGCTAAGGAGTGTGTAACAACTCACCTGCCGAATCAACTAGCCCCGAAAATGGATGG CGCTTAAGCGCGCGACCTATACCCGGCCG	936 (0.017707%)	
TGGACTTTGGCTACACCATGAAAGCTTTGAGAAGCAAGAAGAAGGTTGGTTAGTGTTTTGGAGTCGAATAT GACTTGATGTCATGTGTATGATTGAGTAT	53 (0.001003%)	
TGGAGGGCAAGTCTGGTGCCAGCAGCCGCGGTAATTCCAGCTCCAATAGCGTATATTTAAGTTGTTGCAGT TAAAAAGCTCGTAGTTGAACCTTGGGATG	298 (0.005638%)	
TGGCTGGGGCGGCACATCTGTTAAAAGATAACGCAGGTGTCCTAAGATGAGCTCAACGAGAACAGAAATCT CGTGTGGAACAAAAGGGTAAAAGCTCGTT	4 (0.000076%)	
TGGGCATCGACACCTTGCGGCTAGGAACTGGAACGAGACGGGTGGCAAAGATTTCGAGTAGCACTTCATAC TACCGTGGGTTTTTTAAACCTTCCGAGTT	27 (0.000511%)	
TGGGCCGCACGCGCGCTACACTGATGTATTCAACGAGTTCACACCTTGGCCGACAGGCCCGGGTAATCTTT GAAATTTCATCGTGATGGGGATAGATCGA	16 (0.000303%)	
TGGGGAGTTTGGCTGGGGCGGCACATCTGTTAAAAGATAACGCAGGTGTCCTAAGATGAGCTCAACGAGAA CAGAAATCTCGTGTGGAACAAAAGGGTAA	4 (0.000076%)	
TGGGGCGGCACATCTGTTAAAAGATAACGCAGGTGTCCTAAGATGAGCTCAACGAGAACAGAAATCTCGTG TGGAACAAAAGGGTAAAAGCTCGTTTGAT	14 (0.000265%)	
TGGGTGGTTCGCCGCCCGCGACGTCGCGAGAAGTCCACTAAACCTTATCATTTAGAGGAAGGAGAAGTCGT AACAAGGTTTCCGTAGGTGAACCTGCGGA	7 (0.000132%)	
TGGTCTTCAACGAGGAATTCCTAGTAAGCGCGAGTCATCAGCTCGCGTTGACTACGTCCCTGCCCTTTGTA CACACCGCCCGTCGCTCCTACCGATTGAA	52 (0.000984%)	
TGGTGCCAGCAGCCGCGGTAATTCCAGCTCCAATAGCGTATATTTAAGTTGTTGCAGTTAAAAAGCTCGTA GTTGAACCTTGGGATGGGTCGGCCGGTCC	5 (0.000095%)	
TGGTTCGCCGCCCGCGACGTCGCGAGAAGTCCACTAAACCTTATCATTTAGAGGAAGGAGAAGTCGTAACA AGGTTTCCGTAGGTGAACCTGCGGAAGGA	3 (0.000057%)	
TGTACTCATTCCAATTACCAGACTCGAAAGAGCCCGGTATTGTTATTTATTGTCACTACCTCCCCGTGTCA GGATTGGGTAATTTGCGCGCCTGCTGCCT	9 (0.000170%)	
TGTAGCCAAAGTCCATATGAGTCTTTGGCTTTGTGTCTTT	6910 (0.052290%)	
TGTATCCAGAGCGTAGGCTTGCTTTGAGCACTCTAATTTCTTCAAAGTAACAGCGCCGGAGGCACGACCCG GCCAATTAAGACCAGGAGCGTATCGCCGA	26 (0.000492%)	
TGTATCCTTGTTAGAAGACACAAAGCCAAAGACTCATATGGACTTTGGCTACACCATGAAAGCTTTGAGAA GCAAGAAGAAGGTTGGTTAGTGTTTTGGA	37 (0.000700%)	
TGTCAGGTGGGGAGTTTGGCTGGGGCGGCACATCTGTTAAAAGATAACGCAGGTGTCCTAAGATGAGCTCA ACGAGAACAGAAATCTCGTGTGGAACAAA	36 (0.000681%)	
TGTCATGTGTATGATTGAGTATAAGAACTTAAACCGCAAC	725 (0.005486%)	
TGTCCCGAGTGTGAGCGAGGTGTGAGTGTCGCCCATGGGCATCGACACCTTGCGGCTAGGAACTGGAACGA GACGGGTGGCAAAGATTTCGAGTAGCACT	7 (0.000132%)	
TGTCGCCCATGGGCATCGACACCTTGCGGCTAGGAACTGGAACGAGACGGGTGGCAAAGATTTCGAGTAGC ACTTCATACTACCGTGGGTTTTTTAAACC	3 (0.000057%)	
TGTCGGTACGCTCCAGGCGTCCTTGGCTCGGATTTAGGCCAACCGCGTGCGGTAACACACGGGAGACCAGC TTCCGTCCCGCATCAGCAAAGGATGGTGA	3 (0.000057%)	
TGTCTCAAAGATTAAGCCATGCATGTGTAAGTATGAACGAATTCAGACTGTGAAACTGCGAATGGCTCATT AAATCAGTTATAGTTTGTTTGATGGTAAC	4 (0.000076%)	
TGTGAGCGAGGTGTGAGTGTCGCCCATGGGCATCGACACCTTGCGGCTAGGAACTGGAACGAGACGGGTAG CAAAGATTTCGAGTAGCACTTCATACTAC	7 (0.000132%)	

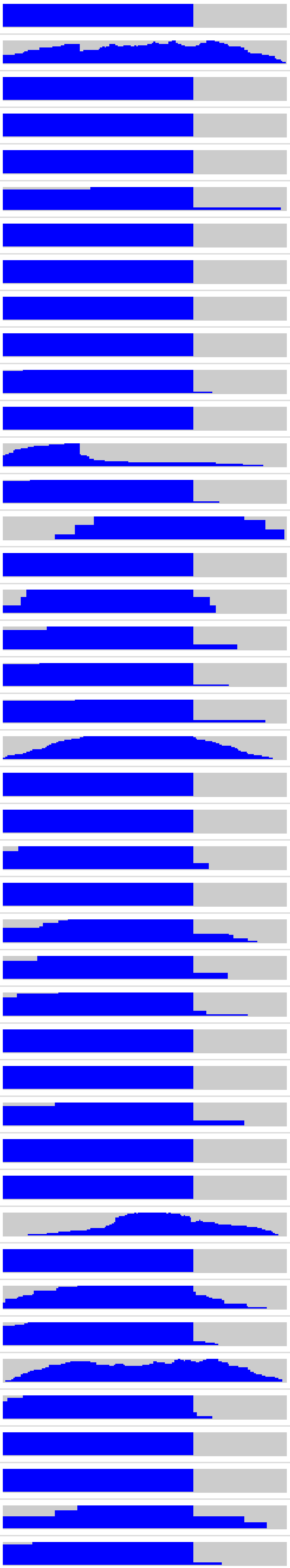

TGTGAGCGAGGTGTGAGTGTCGCCCATGGGCATCGACACCTTGCGGCTAGGAACTGGAACGAGACGGGTGGCAAAGATTTCGAGTAGCACTTCATACTAC	3 (0.000057%)	
TGTGAGTGTCGCCCATGGGCATCGACACCTTGCGGCTAGGAACTGGAACGAGACGGGTAGCAAAGATTTCGAGTAGCACTTCATACTACCGTGGGTTTTT	10 (0.000189%)	
TGTGAGTGTCGCCCATGGGCATCGACACCTTGCGGCTAGGAACTGGAACGAGACGGGTGGCAAAGATTTCGAGTAGCACTTCATACTACCGTGGGTTTTT	9 (0.000170%)	
TGTGATGCCCTTAGATGTTCTGGGCCGCACGCGCGCTACACTGATGTATTCAACGAGTTCACACCTTGGCCGACAGGCCCGGGTAATCTTTGAAATTTCA	6 (0.000114%)	
TGTTAAAAGATAACGCAGGTGTCCTAAGATGAGCTCAACGAGAACAGAAATCTCGTGTGGAACAAAAGGGTAAAAGCTCGTTTGATTCTGATTTTCAGTA	3 (0.000057%)	
TGTTAGAAGACACAAAGCCAAAGACTCATATGGACTTTGGCTACACCATGAAAGCTTTGAGAAGCAAGAAGAAGGTTGGTTAGTGTTTTGGAGTCGAATA	39 (0.000738%)	
TGTTAGTTTCTTTTCCTCCGCTTATTGATATGCTTAAACTCAGCGGGTAATCCCGCCTGACCTGGGGTCGCTATATGGACTTTGGGTCATCTACAGCTTC	8 (0.000151%)	
TGTTATCCCATGCTAATGTATCCAGAGCGTAGGCTTGCTTTGAGCACTCTAATTTCTTCAAAGTAACAGCGCCGGAGGCACGACCCGGCCAATTAAGACC	14 (0.000265%)	
TGTTCCATCGACCAGAGGCTGTTCACCTTGGAGACCTGATGCGGTTATGAGTACGACCGGGCGTGAGCGGCACTCGGTCCTCCGGATTTTCAAGGGCCGC	3 (0.000057%)	
TGTTGAGCTTGACTCTAGTCCGACTTTGTGAAATGACTTGAGAGGTGTAGGATAAGTGGGAGCTTCGGCGCAAGTGAAATACCACTACTTTTAACGTTAT	15 (0.000284%)	
TGTTGGTCTTCAACGAGGAATTCCTAGTAAGCGCGAGTCATCAGCTCGCGTTGACTACGTCCCTGCCCTTTGTACACACCGCCCGTCGCTCCTACCGATT	199 (0.003765%)	
TGTTTCCTTGTTAGAAGACACAAAGCCAAAGACTCATATGGACTTTGGCTACACCATGAAAGCTTTGAGAAGCAAGAAGAAGGTTGGTTAGTGTTTTGGA	27 (0.000511%)	
TTAAAAGATAACGCAGGTGTCCTAAGATGAGCTCAACGAGAACAGAAATCTCGTGTGGAACAAAAGGGTAAAAGCTCGTTTGATTCTGATTTTCAGTACG	25 (0.000473%)	
TTAAAAGCCTAAGTAGTGTTTCCTTGTTAGAAGACACAAAGCCAAAGACTCATATGGACTTTGGCTACACCATGAAAGCTTTGAGAAGCAAGAAGAAGGT	146 (0.002762%)	
TTAAAGGCGTAAGAATTGTATCCTTGTTAAAAGACACAAAGCCAAAGACTCATATGGACTTTGGCTACACCATGAAAGCTTTGAGAAGCAAGAAGAAGGT	151 (0.002857%)	
TTAAAGGCGTAAGAATTGTATCCTTGTTAGAAGACACAAAGCCAAAGACTCATATGGACTTTGGCTACACCATGAAAGCTTTGAGAAGCAAGAAGAAGGT	116 (0.002195%)	
TTAAATCAGTTATAGTTTGTTTGATGGTAACTACTACTCGGATAACCGTAGTAATTCTAGAGCTAATACGTGCAACAAACCCCGACTTATGGAAGGGACG	5 (0.000095%)	
TTAACAGGGACAGTCGGGGGCATTCGTATTTCATAGTCAGAGGTGAAATTCTTGGATTTATGAAAGACGAACAACTGCGAAAGCATTTGCCAAGGATGTT	203 (0.003840%)	
TTAAGCCATGCATGTGTAAGTATGAACGAATTCAGACTGTGAAACTGCGAATGGCTCATTAAATCAGTTATAGTTTGTTTGATGGTAACTACTACTCGGA	15 (0.000284%)	
TTAAGCGCGCGACCTATACCCGGCCGTCGGGGCAAGAGCCAGGCCTCGATGAGTAGGAGGGCGCGGCGGTCGCTGCAAAACCTAGGGCGCGAGCCCGGGC	12 (0.000227%)	
TTAAGGGATTTAGATTGTACTCATTCCAATTACCAGACTCGAAAGAGCCCGGTATTGTTATTTATTGTCACTACCTCCCCGTGTCAGGATTGGGTAATTT	17 (0.000322%)	
TTAAGTTCTTATACTCAATCATACACATGACATCAAGTCATATTCGACTCCAAAACACTAACCAACCTTCTTCTTGCTTCTCAAAGCTTTCATGGTGTAG	2940 (0.055620%)	
TTAAGTTGTTATACTCAATCATACACATGACAACAAGTCATATTCGACTCCAAAACACTAACCAACCTTCTTCTTGCTTCTCAAAGCTTTCATGGTGTAG	64 (0.001211%)	
TTAAGTTGTTATACTCAATCATACACATGACATCAAGTCA	457 (0.003458%)	
TTACCAGACTCGAAAGAGCCCGGTATTGTTATTTATTGTCACTACCTCCCCGTGTCAGGATTGGGTAATTTGCGCGCCTGCTGCCTTCCTTGGATGTGGT	34 (0.000643%)	
TTACGGGTTTACTCACCCGTTGACTCGCACACATGTCAGACTCCTTGGTCCGTGTTTCAAGACGGGTCGAATGGGGAGCCCACAGGCCGACGCCCGGAGC	3214 (0.060803%)	
TTACTCACCCGTTGACTCGCACACATGTCAGACTCCTTGGTCCGTGTTTCAAGACGGGTCGAATGGGGAGCCCACAGGCCGACGCCCGGAGCACGCTGAT	9 (0.000170%)	
TTAGAAGACACAAAGCCAAAGACTCATATGGACTTTGGCTACACCATGAAAGCTTTGAGAAGCAAGAAGAAGGTTGGTTAGTGTTTTGGAGTCGAATATG	97 (0.001835%)	
TTAGATGTTCTGGGCCGCACGCGCGCTACACTGATGTATTCAACGAGTTCACACCTTGGCCGACAGGCCCGGGTAATCTTTGAAATTTCATCGTGATGGG	15 (0.000284%)	
TTAGATTGTACTCATTCCAATTACCAGACTCGAAAGAGCCCGGTATTGTTATTTATTGTCACTACCTCCCCGTGTCAGGATTGGGTAATTTGCGCGCCTG	23 (0.000435%)	
TTAGTTTCTTTTCCTCCGCTTATTGATATGCTTAAACTCAGCGGGTAATCCCGCCTGACCTGGGGTCGCTATATGGACTTTGGGTCATCTACAGCTTCCG	4 (0.000076%)	
TTATACTCAATCATACACATGACAACAAGTCATATTCGACTCCAAAACACTAACCAACCTTCTTCTTGCTTCTCAAAGCTTTCATGGTGTAGCCAAAGTC	295 (0.005581%)	
TTATACTCAATCATACACATGACATCAAGTCATATTCGACTCCAAAACACTAACCAACCTTCTTCTTGCTTCTCAAAGCTTTCATGGTGTAGCCAAAGTC	827 (0.015645%)	
TTATAGTTTGTTTGATGGTAACTACTACTCGGATAACCGTAGTAATTCTAGAGCTAATACGTGCAACAAACCCCGACTTATGGAAGGGACGCATTTATTA	5 (0.000095%)	
TTATCATGAATCATCAGAGCAACGGGCAGAGCCCGCGTCGACCTTTTATCTAATAAATGCGTCCCTTCCATAAGTCGGGGTTTGTTGCACGTATTAGCTC	74 (0.001400%)	
TTATCCCATGCTAATGTATCCAGAGCGTAGGCTTGCTTTGAGCACTCTAATTTCTTCAAAGTAACAGCGCCGGAGGCACGACCCGGCCAATTAAGACCAG	3 (0.000057%)	
TTATCTAATAAATGCGTCCCTTCCATAAGTCGGGGTTTGTTGCACGTATTAGCTCTAGAATTACTACGGTTATCCGAGTAGTAGTTACCATCAAACAAAC	5 (0.000095%)	
TTATTGATATGCTTAAACTCAGCGGGTAATCCCGCCTGACCTGGGGTCGCTATATGGACTTTGGGTCATCTACAGCTTCCGGACAAGAGCGACCGATAAA	13 (0.000246%)	
TTCAACGAGGAATTCCTAGTAAGCGCGAGTCATCAGCTCGCGTTGACTACGTCCCTGCCCTTTGTACACACCGCCCGTCGCTCCTACCGATTGAATGATC	12 (0.000227%)	
TTCAATCGGTAGGAGCGACGGGCGGTGTGTACAAAGGGCAGGGACGTAGTCAACGCGAGCTGATGACTCGCGCTTACTAGGAATTCCTCGTTGAAGACCA	80 (0.001513%)	
TTCACATTTCGTTCATCACCCTTGGCCGGCTATCGAACAGCCGGACTCCCATCAAAAGATGGTTGCCAAGAACATCTTCGTTACGGTTTGCTAATTCTCG	22 (0.000416%)	
TTCACATTTCGTTCATCACCCTTGGCCGGCTTTCGAACAGCCGGACTCCCATCAAAAGATGGTTGCCAAGAACATCTTCGTTACGGTTTGCTAATTCTCG	22 (0.000416%)	
TTCACCACCGCATGTCGGTACGCTCCAGGCGTCCTTGGCTCGGATTTAGGCCAACCGCGTGCGGTAACACACGGGAGACCAGCTTCCGTCCCGCATCAGC	11 (0.000208%)	

TTCACGGTTCGTATTCGTACTGAAAATCAGAATCAAACGAGCTTTTACCCTTTTGTTCCACACGAGATTTCTGTTCTCGTTGAGCTCATCTTAGGACACC	14 (0.000265%)	
TTCATAGTCAGAGGTGAAATTCTTGGATTTATGAAAGACGAACAACTGCGAAAGCATTTGCCAAGGATGTTTTCATTAATCAAGAACGAAAGTTGGGGGC	38 (0.000719%)	
TTCCAACGAAGCACGCCCATCCAACCTAGGCGAGACAAGGGTTCACATTTCGTTCATCACCCTTGGCCGGCTATCGAACAGCCGGACTCCCATCAAAAGA	115 (0.002176%)	
TTCCAACGAAGCACGCCCATCCAACCTAGGCGAGACAAGGGTTCACATTTCGTTCATCACCCTTGGCCGGCTTTCGAACAGCCGGACTCCCATCAAAAGA	2301 (0.043531%)	
TTCCAATTACCAGACTCGAAAGAGCCCGGTATTGTTATTTATTGTCACTACCTCCCCGTGTCAGGATTGGGTAATTTGCGCGCCTGCTGCCTTCCTTGGA	11 (0.000208%)	
TTCCATCGACCAGAGGCTGTTCACCTTGGAGACCTGATGCGGTTATGAGTACGACCGGGCGTGAGCGGCACTCGGTCCTCCGGATTTTCAAGGGCCGCCG	4 (0.000076%)	
TTCCCCGCCACATCCTCTCAAACGCAATGGAAAGAGAGAAAGGACGAGGTCTTGACCGTCATCTTTTGCCCGAAGGACGGATGAGCTTTGGCGGGACTGA	8 (0.000151%)	
TTCCCTTGCCTACATTGTTCCATCGACCAGAGGCTGTTCACCTTGGAGACCTGATGCGGTTATGAGTACGACCGGGCGTGAGCGGCACTCGGTCCTCCGG	10 (0.000189%)	
TTCCTCCGCTTATTGATATGCTTAAACTCAGCGGGTAATCCCGCCTGACCTGGGGTCGCTATATGGACTTTGGGTCATCTACAGCTTCCGGACAAGAGCG	4 (0.000076%)	
TTCCTTGTTAGAAGACACAAAGCCAAAGACTCATATGGACTTTGGCTACACCATGAAAGCTTTGAGAAGCAAGAAGAAGGTTGGTTAGTGTTTTGGAGTC	58 (0.001097%)	
TTCGACTCCAAAACACTAACCAACCTTCTTCTTGCTTCTCAAAGCTTTCATGGTGTAGCCAAAGTCCATATGAGTCTTTGGCTTTGTGTCTTCTAACAAG	130 (0.002459%)	
TTCGCCGCCCGCGACGTCGCGAGAAGTCCACTAAACCTTATCATTTAGAGGAAGGAGAAGTCGTAACAAGGTTTCCGTAGGTGAACCTGCGGAAGGATCG	7 (0.000132%)	
TTCGTACTGAAAATCAGAATCAAACGAGCTTTTACCCTTTTGTTCCACACGAGATTTCTGTTCTCGTTGAGCTCATCTTAGGACACCTGCGTTATCTTTT	25 (0.000473%)	
TTCGTATTCGTACTGAAAATCAGAATCAAACGAGCTTTTACCCTTTTGTTCCACACGAGATTTCTGTTCTCGTTGAGCTCATCTTAGGACACCTGCGTTA	15 (0.000284%)	
TTCGTATTTCATAGTCAGAGGTGAAATTCTTGGATTTATGAAAGACGAACAACTGCGAAAGCATTTGCCAAGGATGTTTTCATTAATCAAGAACGAAAGT	11 (0.000208%)	
TTCTCAAAGCTTTCATGGTGTAGCCAAAGTCCATATGAGTCTTTGGCTTTGTGTCTTCTAACAAGGAAACACTACTTAGGCTTATAAGATGCGGTTGCGG	13 (0.000246%)	
TTCTTATACTCAATCATACACATGACATCAAGTCATATTCGACTCCAAAACACTAACCAACCTTCTTCTTGCTTCTCAAAGCTTTCATGGTGTAGCCAAA	96 (0.001816%)	
TTCTTGCTTCTCAAAGCTTTCATGGTGTAGCCAAAGTCCATATGAGTCTTTGGCTTTGTGTCTTCTAACAAGGAAACACTACTTAGGCTTATAAGATGCG	347 (0.006565%)	
TTCTTTTCCTCCGCTTATTGATATGCTTAAACTCAGCGGGTAATCCCGCCTGACCTGGGGTCGCTATATGGACTTTGGGTCATCTACAGCTTCCGGACAA	37 (0.000700%)	
TTGACTCTAGTCCGACTTTGTGAAATGACTTGAGAGGTGTAGGATAAGTGGGAGCTTCGGCGCAAGTGAAATACCACTACTTTTAACGTTATTTTACTTA	16 (0.000303%)	
TTGAGCTTGACTCTAGTCCGACTTTGTGAAATGACTTGAGAGGTGTAGGATAAGTGGGAGCTTCGGCGCAAGTGAAATACCACTACTTTTAACGTTATTT	32 (0.000605%)	
TTGATCGATC	182040 (0.344387%)	
TTGATGGTAACTACTACTCGGATAACCGTAGTAATTCTAGAGCTAATACGTGCAACAAACCCCGACTTATGGAAGGGACGCATTTATTAGATAAAAGGTC	27 (0.000511%)	
TTGATGTCATGTGTATGATTGAGTATAAGAACTTAAACCG	399 (0.003019%)	
TTGCCGACTTCCCTTGCCTACATTGTTCCATCGACCAGAGGCTGTTCACCTTGGAGACCTGATGCGGTTATGAGTACGACCGGGCGTGAGCGGCACTCGG	145 (0.002743%)	
TTGCCTACATTGTTCCATCGACCAGAGGCTGTTCACCTTGGAGACCTGATGCGGTTATGAGTACGACCGGGCGTGAGCGGCACTCGGTCCTCCGGATTTT	4 (0.000076%)	
TTGCGGTTTAAGTTCTTATACTCAATCATACACATGACAT	2081 (0.015748%)	
TTGCGGTTTAAGTTGTTATACTCAATCATACACATGACAT	664 (0.005025%)	
TTGCTCCGTTCCGCATCCGACCAGGACGCATCGCCGGCCCCCATCCGCTTCCCTCCCGACAATTTCAAGCACTCTTTGACTCTCTTTTCAAAGTCCTTTT	8 (0.000151%)	
TTGCTGATGCGGGACGGAAGCTGGTCTCCCGTGTGTTACCGCACGCGGTTGGCCTAAATCCGAGCCAAGGACGCCTGGAGCGTACCGACATGCGGTGGTG	5 (0.000095%)	
TTGCTTTGAGCACTCTAATTTCTTCAAAGTAACAGCGCCGGAGGCACGACCCGGCCAATTAAGACCAGGAGCGTATCGCCGACCGAAGGGACAAGCCGAC	5 (0.000095%)	
TTGGAGGGCAAGTCTGGTGCCAGCAGCCGCGGTAATTCCAGCTCCAATAGCGTATATTTAAGTTGTTGCAGTTAAAAAGCTCGTAGTTGAACCTTGGGAT	347 (0.006565%)	
TTGGCATGCATCATAAGGATACTAAATCCTATTTTCTGGTAAATTTTCATAATTTTTTGACACCTCTAGCTAGGTCATTTGACCTGATACAACATCGGAT	387 (0.007321%)	
TTGGCTGGGGCGGCACATCTGTTAAAAGATAACGCAGGTGTCCTAAGATGAGCTCAACGAGAACAGAAATCTCGTGTGGAACAAAAGGGTAAAAGCTCGT	9 (0.000170%)	
TTGGTCTTCAACGAGGAATTCCTAGTAAGCGCGAGTCATCAGCTCGCGTTGACTACGTCCCTGCCCTTTGTACACACCGCCCGTCGCTCCTACCGATTGA	111 (0.002100%)	
TTGTACTCATTCCAATTACCAGACTCGAAAGAGCCCGGTATTGTTATTTATTGTCACTACCTCCCCGTGTCAGGATTGGGTAATTTGCGCGCCTGCTGCC	6 (0.000114%)	
TTGTATCCTTGTTAGAAGACACAAAGCCAAAGACTCATATGGACTTTGGCTACACCATGAAAGCTTTGAGAAGCAAGAAGAAGGTTGGTTAGTGTTTTGG	16 (0.000303%)	
TTGTCAGGTGGGGAGTTTGGCTGGGGCGGCACATCTGTTAAAAGATAACGCAGGTGTCCTAAGATGAGCTCAACGAGAACAGAAATCTCGTGTGGAACAA	8 (0.000151%)	
TTGTCTCAAAGATTAAGCCATGCATGTGTAAGTATGAACGAATTCAGACTGTGAAACTGCGAATGGCTCATTAAATCAGTTATAGTTTGTTTGATGGTAA	6 (0.000114%)	
TTGTTAGAAGACACAAAGCCAAAGACTCATATGGACTTTGGCTACACCATGAAAGCTTTGAGAAGCAAGAAGAAGGTTGGTTAGTGTTTTGGAGTCGAAT	71 (0.001343%)	
TTGTTAGTTTCTTTTCCTCCGCTTATTGATATGCTTAAACTCAGCGGGTAATCCCGCCTGACCTGGGGTCGCTATATGGACTTTGGGTCATCTACAGCTT	10 (0.000189%)	
TTGTTCCATCGACCAGAGGCTGTTCACCTTGGAGACCTGATGCGGTTATGAGTACGACCGGGCGTGAGCGGCACTCGGTCCTCCGGATTTTCAAGGGCCG	4 (0.000076%)	
TTGTTGGTCTTCAACGAGGAATTCCTAGTAAGCGCGAGTCATCAGCTCGCGTTGACTACGTCCCTGCCCTTTGTACACACCGCCCGTCGCTCCTACCGAT	211 (0.003992%)	

TTGTTTGATGGTAACTACTACTCGGATAACCGTAGTAATTCTAGAGCTAATACGTGCAACAAACCCCGACTTATGGAAGGGACGCATTTATTAGATAAAA	12 (0.000227%)	
TTTAAGTTCTTATACTCAATCATACACATGACATCAAGTC	812 (0.006145%)	
TTTAAGTTGTTATACTCAATCATACACATGACAACAAGTCATATTCGACTCCAAAACACTAACCAACCTTCTTCTTGCTTCTCAAAGCTTTCATGGTGTA	30 (0.000568%)	
TTTAAGTTGTTATACTCAATCATACACATGACATCAAGTC	379 (0.002868%)	
TTTACTCACCCGTTGACTCGCACACATGTCAGACTCCTTGGTCCGTGTTTCAAGACGGGTCGAATGGGGAGCCCACAGGCCGACGCCCGGAGCACGCTGA	9 (0.000170%)	
TTTAGATTGTACTCATTCCAATTACCAGACTCGAAAGAGCCCGGTATTGTTATTTATTGTCACTACCTCCCCGTGTCAGGATTGGGTAATTTGCGCGCCT	145 (0.002743%)	
TTTATCTAATAAATGCGTCCCTTCCATAAGTCGGGGTTTGTTGCACGTATTAGCTCTAGAATTACTACGGTTATCCGAGTAGTAGTTACCATCAAACAAA	7 (0.000132%)	
TTTCACGGTTCGTATTCGTACTGAAAATCAGAATCAAACGAGCTTTTACCCTTTTGTTCCACACGAGATTTCTGTTCTCGTTGAGCTCATCTTAGGACAC	57 (0.001078%)	
TTTCATAATTTTTTGACACCTCTAGCTAGGTCATTTGACCTGATACAACATCGGATTTTCATGGTCTAGTTGGGGCTCCGTGGGCATATTTGATGCAAAC	9 (0.000170%)	
TTTCATAGTCAGAGGTGAAATTCTTGGATTTATGAAAGACGAACAACTGCGAAAGCATTTGCCAAGGATGTTTTCATTAATCAAGAACGAAAGTTGGGGG	9 (0.000170%)	
TTTCCAACGAAGCACGCCCATCCAACCTAGGCGAGACAAGGGTTCACATTTCGTTCATCACCCTTGGCCGGCTATCGAACAGCCGGACTCCCATCAAAAG	270 (0.005108%)	
TTTCCTCCGCTTATTGATATGCTTAAACTCAGCGGGTAATCCCGCCTGACCTGGGGTCGCTATATGGACTTTGGGTCATCTACAGCTTCCGGACAAGAGC	11 (0.000208%)	
TTTCCTTGTTAGAAGACACAAAGCCAAAGACTCATATGGACTTTGGCTACACCATGAAAGCTTTGAGAAGCAAGAAGAAGGTTGGTTAGTGTTTTGGAGT	631 (0.011937%)	
TTTCGTTCATCACCCTTGGCCGGCTATCGAACAGCCGGACTCCCATCAAAAGATGGTTGCCAAGAACATCTTCGTTACGGTTTGCTAATTCTCGGAATAA	10 (0.000189%)	
TTTCTGGTAAATTTTCATAATTTTTTGACACCTCTAGCTAGGTCATTTGACCTGATACAACATCGGATTTTCATGGTCTAGTTGGGGCTCCGTGGGCATA	7 (0.000132%)	
TTTCTTTTCCTCCGCTTATTGATATGCTTAAACTCAGCGGGTAATCCCGCCTGACCTGGGGTCGCTATATGGACTTTGGGTCATCTACAGCTTCCGGACA	12 (0.000227%)	
TTTGATGGTAACTACTACTCGGATAACCGTAGTAATTCTAGAGCTAATACGTGCAACAAACCCCGACTTATGGAAGGGACGCATTTATTAGATAAAAGGT	3 (0.000057%)	
TTTGCCGACTTCCCTTGCCTACATTGTTCCATCGACCAGAGGCTGTTCACCTTGGAGACCTGATGCGGTTATGAGTACGACCGGGCGTGAGCGGCACTCG	2275 (0.043039%)	
TTTGCTGATGCGGGACGGAAGCTGGTCTCCCGTGTGTTACCGCACGCGGTTGGCCTAAATCCGAGCCAAGGACGCCTGGAGCGTACCGACATGCGGTGGT	19 (0.000359%)	
TTTGGCTGGGGCGGCACATCTGTTAAAAGATAACGCAGGTGTCCTAAGATGAGCTCAACGAGAACAGAAATCTCGTGTGGAACAAAAGGGTAAAAGCTCG	21 (0.000397%)	
TTTGTTTGATGGTAACTACTACTCGGATAACCGTAGTAATTCTAGAGCTAATACGTGCAACAAACCCCGACTTATGGAAGGGACGCATTTATTAGATAAA	7 (0.000132%)	
TTTTATCTAATAAATGCGTCCCTTCCATAAGTCGGGGTTTGTTGCACGTATTAGCTCTAGAATTACTACGGTTATCCGAGTAGTAGTTACCATCAAACAA	10 (0.000189%)	
TTTTCATAATTTTTTGACACCTCTAGCTAGGTCATTTGACCTGATACAACATCGGATTTTCATGGTCTAGTTGGGGCTCCGTGGGCATATTTGATGCAAA	6 (0.000114%)	
TTTTCCTCCGCTTATTGATATGCTTAAACTCAGCGGGTAATCCCGCCTGACCTGGGGTCGCTATATGGACTTTGGGTCATCTACAGCTTCCGGACAAGAG	22 (0.000416%)	
TTTTCTGGTAAATTTTCATAATTTTTTGACACCTCTAGCTAGGTCATTTGACCTGATACAACATCGGATTTTCATGGTCTAGTTGGGGCTCCGTGGGCAT	9 (0.000170%)	
TTTTTTTTTT	55119 (0.104275%)	

Before filtering: read2: quality

Value of each position will be shown on mouse over.

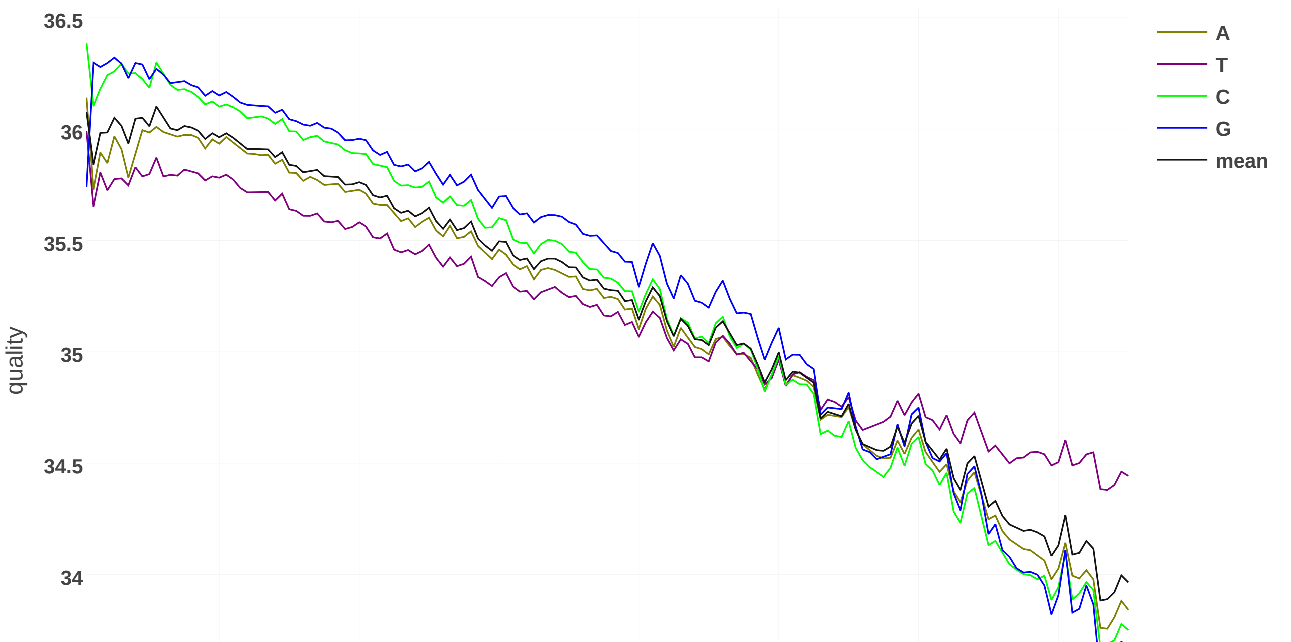

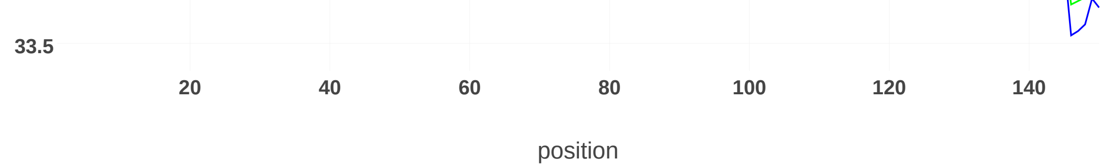

Before filtering: read2: base contents

Value of each position will be shown on mouse over.

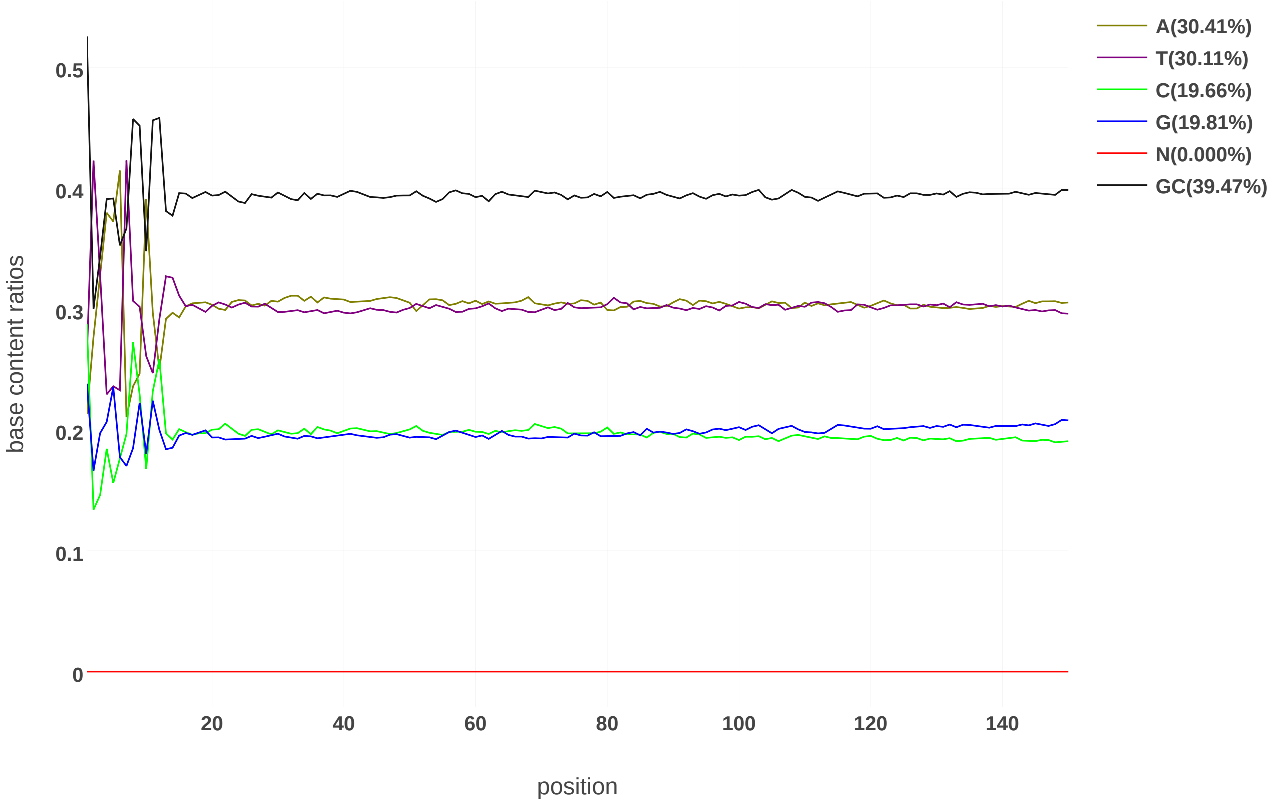

Before filtering: read2: KMER counting

Darker background means larger counts. The count will be shown on mouse over.

	AA	AT	AC	AG	TA	TT	TC	TG	CA	CT	CC	CG	GA	GT	GC	GG
AAA	AAAAA	AAAAT	AAAAC	AAAAG	AAATA	AAATT	AAATC	AAATG	AAACA	AAACT	AAACC	AAACG	AAAGA	AAAGT	AAAGC	AAAGG
AAT	AATAA	AATAT	AATAC	AATAG	AATTA	AATTT	AATTC	AATTG	AATCA	AATCT	AATCC	AATCG	AATGA	AATGT	AATGC	AATGG
AAC	AACAA	AACAT	AACAC	AACAG	AACTA	AACTT	AACTC	AACTG	AACCA	AACCT	AACCC	AACCG	AACGA	AACGT	AACGC	AACGG
AAG	AAGAA	AAGAT	AAGAC	AAGAG	AAGTA	AAGTT	AAGTC	AAGTG	AAGCA	AAGCT	AAGCC	AAGCG	AAGGA	AAGGT	AAGGC	AAGGG
ATA	ATAAA	ATAAT	ATAAC	ATAAG	ATATA	ATATT	ATATC	ATATG	ATACA	ATACT	ATACC	ATACG	ATAGA	ATAGT	ATAGC	ATAGG
ATT	ATTAA	ATTAT	ATTAC	ATTAG	ATTTA	ATTTT	ATTTC	ATTTG	ATTCA	ATTCT	ATTCC	ATTCG	ATTGA	ATTGT	ATTGC	ATTGG
ATC	ATCAA	ATCAT	ATCAC	ATCAG	ATCTA	ATCTT	ATCTC	ATCTG	ATCCA	ATCCT	ATCCC	ATCCG	ATCGA	ATCGT	ATCGC	ATCGG
ATG	ATGAA	ATGAT	ATGAC	ATGAG	ATGTA	ATGTT	ATGTC	ATGTG	ATGCA	ATGCT	ATGCC	ATGCG	ATGGA	ATGGT	ATGGC	ATGGG
ACA	ACAAA	ACAAT	ACAAC	ACAAG	ACATA	ACATT	ACATC	ACATG	ACACA	ACACT	ACACC	ACACG	ACAGA	ACAGT	ACAGC	ACAGG
ACT	ACTAA	ACTAT	ACTAC	ACTAG	ACTTA	ACTTT	ACTTC	ACTTG	ACTCA	ACTCT	ACTCC	ACTCG	ACTGA	ACTGT	ACTGC	ACTGG
ACC	ACCAA	ACCAT	ACCAC	ACCAG	ACCTA	ACCTT	ACCTC	ACCTG	ACCCA	ACCCT	ACCCC	ACCCG	ACCGA	ACCGT	ACCGC	ACCGG
ACG	ACGAA	ACGAT	ACGAC	ACGAG	ACGTA	ACGTT	ACGTC	ACGTG	ACGCA	ACGCT	ACGCC	ACGCG	ACGGA	ACGGT	ACGGC	ACGGG
AGA	AGAAA	AGAAT	AGAAC	AGAAG	AGATA	AGATT	AGATC	AGATG	AGACA	AGACT	AGACC	AGACG	AGAGA	AGAGT	AGAGC	AGAGG
AGT	AGTAA	AGTAT	AGTAC	AGTAG	AGTTA	AGTTT	AGTTC	AGTTG	AGTCA	AGTCT	AGTCC	AGTCG	AGTGA	AGTGT	AGTGC	AGTGG
AGC	AGCAA	AGCAT	AGCAC	AGCAG	AGCTA	AGCTT	AGCTC	AGCTG	AGCCA	AGCCT	AGCCC	AGCCG	AGCGA	AGCGT	AGCGC	AGCGG
AGG	AGGAA	AGGAT	AGGAC	AGGAG	AGGTA	AGGTT	AGGTC	AGGTG	AGGCA	AGGCT	AGGCC	AGGCG	AGGGA	AGGGT	AGGGC	AGGGG
TAA	TAAAA	TAAAT	TAAAC	TAAAG	TAATA	TAATT	TAATC	TAATG	TAACA	TAACT	TAACC	TAACG	TAAGA	TAAGT	TAAGC	TAAGG
TAT	TATAA	TATAT	TATAC	TATAG	TATTA	TATTT	TATTC	TATTG	TATCA	TATCT	TATCC	TATCG	TATGA	TATGT	TATGC	TATGG
TAC	TACAA	TACAT	TACAC	TACAG	TACTA	TACTT	TACTC	TACTG	TACCA	TACCT	TACCC	TACCG	TACGA	TACGT	TACGC	TACGG
TAG	TAGAA	TAGAT	TAGAC	TAGAG	TAGTA	TAGTT	TAGTC	TAGTG	TAGCA	TAGCT	TAGCC	TAGCG	TAGGA	TAGGT	TAGGC	TAGGG
TTA	TTAAA	TTAAT	TTAAC	TTAAG	TTATA	TTATT	TTATC	TTATG	TTACA	TTACT	TTACC	TTACG	TTAGA	TTAGT	TTAGC	TTAGG
TTT	TTTAA	TTTAT	TTTAC	TTTAG	TTTTA	TTTTT	TTTTC	TTTTG	TTTCA	TTTCT	TTTCC	TTTCG	TTTGA	TTTGT	TTTGC	TTTGG
TTC	TTCAA	TTCAT	TTCAC	TTCAG	TTCTA	TTCTT	TTCTC	TTCTG	TTCCA	TTCCT	TTCCC	TTCCG	TTCGA	TTCGT	TTCGC	TTCGG
TTG	TTGAA	TTGAT	TTGAC	TTGAG	TTGTA	TTGTT	TTGTC	TTGTG	TTGCA	TTGCT	TTGCC	TTGCG	TTGGA	TTGGT	TTGGC	TTGGG
TCA	TCAAA	TCAAT	TCAAC	TCAAG	TCATA	TCATT	TCATC	TCATG	TCACA	TCACT	TCACC	TCACG	TCAGA	TCAGT	TCAGC	TCAGG
TCT	TCTAA	TCTAT	TCTAC	TCTAG	TCTTA	TCTTT	TCTTC	TCTTG	TCTCA	TCTCT	TCTCC	TCTCG	TCTGA	TCTGT	TCTGC	TCTGG
TCC	TCCAA	TCCAT	TCCAC	TCCAG	TCCTA	TCCTT	TCCTC	TCCTG	TCCCA	TCCCT	TCCCC	TCCCG	TCCGA	TCCGT	TCCGC	TCCGG
TCG	TCGAA	TCGAT	TCGAC	TCGAG	TCGTA	TCGTT	TCGTC	TCGTG	TCGCA	TCGCT	TCGCC	TCGCG	TCGGA	TCGGT	TCGGC	TCGGG
TGA	TGAAA	TGAAT	TGAAC	TGAAG	TGATA	TGATT	TGATC	TGATG	TGACA	TGACT	TGACC	TGACG	TGAGA	TGAGT	TGAGC	TGAGG
TGT	TGTAA	TGTAT	TGTAC	TGTAG	TGTTA	TGTTT	TGTTC	TGTTG	TGTCA	TGTCT	TGTCC	TGTCG	TGTGA	TGTGT	TGTGC	TGTGG
TGC	TGCAA	TGCAT	TGCAC	TGCAG	TGCTA	TGCTT	TGCTC	TGCTG	TGCCA	TGCCT	TGCCC	TGCCG	TGCGA	TGCGT	TGCGC	TGCGG
TGG	TGGAA	TGGAT	TGGAC	TGGAG	TGGTA	TGGTT	TGGTC	TGGTG	TGGCA	TGGCT	TGGCC	TGGCG	TGGGA	TGGGT	TGGGC	TGGGG
CAA	CAAAA	CAAAT	CAAAC	CAAAG	CAATA	CAATT	CAATC	CAATG	CAACA	CAACT	CAACC	CAACG	CAAGA	CAAGT	CAAGC	CAAGG
CAT	CATAA	CATAT	CATAC	CATAG	CATTA	CATTT	CATTC	CATTG	CATCA	CATCT	CATCC	CATCG	CATGA	CATGT	CATGC	CATGG
CAC	CACAA	CACAT	CACAC	CACAG	CACTA	CACTT	CACTC	CACTG	CACCA	CACCT	CACCC	CACCG	CACGA	CACGT	CACGC	CACGG
CAG	CAGAA	CAGAT	CAGAC	CAGAG	CAGTA	CAGTT	CAGTC	CAGTG	CAGCA	CAGCT	CAGCC	CAGCG	CAGGA	CAGGT	CAGGC	CAGGG
CTA	CTAAA	CTAAT	CTAAC	CTAAG	CTATA	CTATT	CTATC	CTATG	CTACA	CTACT	CTACC	CTACG	CTAGA	CTAGT	CTAGC	CTAGG
CTT	CTTAA	CTTAT	CTTAC	CTTAG	CTTTA	CTTTT	CTTTC	CTTTG	CTTCA	CTTCT	CTTCC	CTTCG	CTTGA	CTTGT	CTTGC	CTTGG
CTC	CTCAA	CTCAT	CTCAC	CTCAG	CTCTA	CTCTT	CTCTC	CTCTG	CTCCA	CTCCT	CTCCC	CTCCG	CTCGA	CTCGT	CTCGC	CTCGG
CTG	CTGAA	CTGAT	CTGAC	CTGAG	CTGTA	CTGTT	CTGTC	CTGTG	CTGCA	CTGCT	CTGCC	CTGCG	CTGGA	CTGGT	CTGGC	CTGGG
CCA	CCAAA	CCAAT	CCAAC	CCAAG	CCATA	CCATT	CCATC	CCATG	CCACA	CCACT	CCACC	CCACG	CCAGA	CCAGT	CCAGC	CCAGG
CCT	CCTAA	CCTAT	CCTAC	CCTAG	CCTTA	CCTTT	CCTTC	CCTTG	CCTCA	CCTCT	CCTCC	CCTCG	CCTGA	CCTGT	CCTGC	CCTGG
CCC	CCCAA	CCCAT	CCCAC	CCCAG	CCCTA	CCCTT	CCCTC	CCCTG	CCCCA	CCCCT	CCCCC	CCCCG	CCCGA	CCCGT	CCCGC	CCCGG
CCG	CCGAA	CCGAT	CCGAC	CCGAG	CCGTA	CCGTT	CCGTC	CCGTG	CCGCA	CCGCT	CCGCC	CCGCG	CCGGA	CCGGT	CCGGC	CCGGG
CGA	CGAAA	CGAAT	CGAAC	CGAAG	CGATA	CGATT	CGATC	CGATG	CGACA	CGACT	CGACC	CGACG	CGAGA	CGAGT	CGAGC	CGAGG
CGT	CGTAA	CGTAT	CGTAC	CGTAG	CGTTA	CGTTT	CGTTC	CGTTG	CGTCA	CGTCT	CGTCC	CGTCG	CGTGA	CGTGT	CGTGC	CGTGG
CGC	CGCAA	CGCAT	CGCAC	CGCAG	CGCTA	CGCTT	CGCTC	CGCTG	CGCCA	CGCCT	CGCCC	CGCCG	CGCGA	CGCGT	CGCGC	CGCGG
CGG	CGGAA	CGGAT	CGGAC	CGGAG	CGGTA	CGGTT	CGGTC	CGGTG	CGGCA	CGGCT	CGGCC	CGGCG	CGGGA	CGGGT	CGGGC	CGGGG
GAA	GAAAA	GAAAT	GAAAC	GAAAG	GAATA	GAATT	GAATC	GAATG	GAACA	GAACT	GAACC	GAACG	GAAGA	GAAGT	GAAGC	GAAGG
GAT	GATAA	GATAT	GATAC	GATAG	GATTA	GATTT	GATTC	GATTG	GATCA	GATCT	GATCC	GATCG	GATGA	GATGT	GATGC	GATGG

GAC	GACAA	GACAT	GACAC	GACAG	GACTA	GACTT	GACTC	GACTG	GACCA	GACCT	GACCC	GACCG	GACGA	GACGT	GACGC	GACGG
GAG	GAGAA	GAGAT	GAGAC	GAGAG	GAGTA	GAGTT	GAGTC	GAGTG	GAGCA	GAGCT	GAGCC	GAGCG	GAGGA	GAGGT	GAGGC	GAGGG
GTA	GTAAA	GTAAT	GTAAC	GTAAG	GTATA	GTATT	GTATC	GTATG	GTACA	GTACT	GTACC	GTACG	GTAGA	GTAGT	GTAGC	GTAGG
GTT	GTTAA	GTTAT	GTTAC	GTTAG	GTTTA	GTTTT	GTTTC	GTTTG	GTTCA	GTTCT	GTTCC	GTTCG	GTTGA	GTTGT	GTTGC	GTTGG
GTC	GTCAA	GTCAT	GTCAC	GTCAG	GTCTA	GTCTT	GTCTC	GTCTG	GTCCA	GTCCT	GTCCC	GTCCG	GTCGA	GTCGT	GTCGC	GTCGG
GTG	GTGAA	GTGAT	GTGAC	GTGAG	GTGTA	GTGTT	GTGTC	GTGTG	GTGCA	GTGCT	GTGCC	GTGCG	GTGGA	GTGGT	GTGGC	GTGGG
GCA	GCAAA	GCAAT	GCAAC	GCAAG	GCATA	GCATT	GCATC	GCATG	GCACA	GCACT	GCACC	GCACG	GCAGA	GCAGT	GCAGC	GCAGG
GCT	GCTAA	GCTAT	GCTAC	GCTAG	GCTTA	GCTTT	GCTTC	GCTTG	GCTCA	GCTCT	GCTCC	GCTCG	GCTGA	GCTGT	GCTGC	GCTGG
GCC	GCCAA	GCCAT	GCCAC	GCCAG	GCCTA	GCCTT	GCCTC	GCCTG	GCCCA	GCCCT	GCCCC	GCCCG	GCCGA	GCCGT	GCCGC	GCCGG
GCG	GCGAA	GCGAT	GCGAC	GCGAG	GCGTA	GCGTT	GCGTC	GCGTG	GCGCA	GCGCT	GCGCC	GCGCG	GCGGA	GCGGT	GCGGC	GCGGG
GGA	GGAAA	GGAAT	GGAAC	GGAAG	GGATA	GGATT	GGATC	GGATG	GGACA	GGACT	GGACC	GGACG	GGAGA	GGAGT	GGAGC	GGAGG
GGT	GGTAA	GGTAT	GGTAC	GGTAG	GGTTA	GGTTT	GGTTC	GGTTG	GGTCA	GGTCT	GGTCC	GGTCG	GGTGA	GGTGT	GGTGC	GGTGG
GGC	GGCAA	GGCAT	GGCAC	GGCAG	GGCTA	GGCTT	GGCTC	GGCTG	GGCCA	GGCCT	GGCCC	GGCCG	GGCGA	GGCGT	GGCGC	GGCGG
GGG	GGGAA	GGGAT	GGGAC	GGGAG	GGGTA	GGGTT	GGGTC	GGGTG	GGGCA	GGGCT	GGGCC	GGGCG	GGGGA	GGGGT	GGGGC	GGGGG

Before filtering: read2: overrepresented sequences

Sampling rate: 1 / 20

overrepresented sequence	count (% of bases)	distribution: cycle 1 ~ cycle 150
AAAAAAAAAAAAAAAAAAAA	6570 (0.024859%)	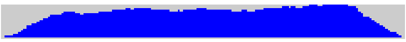
AAAAGATAACGCAGGTGTCCTAAGATGAGCTCAACGAGAACAGAAATCTCGTGTGGAACAAAAGGGTAAAAGCTCGTTTGATTCTGATTTTCAGTACGAA	7 (0.000132%)	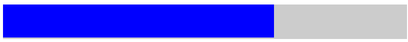
AAAAGTGTCGGAGTTTTTTCAGCAGTTCTCGGACAAAAATTGCTGAGTGGCCGAGAAGAATGGGCGTGTCATGCGTGGGCTGACATGGATTCTTCGAGGC	5 (0.000095%)	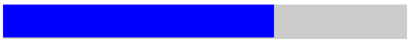
AAAATCATCAATCGTTCCAACTAATCTACCGAAGTACTCGGCTAAGAAGAAAGAAGACGGACGAATCCGAGCCAAAGCCGTACAAGCGCGAGATACCTTC	22 (0.000416%)	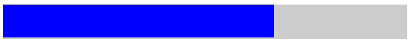
AAACACAGGCCCCGGAACTCATCATCGAGCGTAACATCGCCCGTGAATTAACTGAGAAGGATAGGTGGTAGGTAGTTCGATGCGCGAGCATGGAGCCTAC	11 (0.000208%)	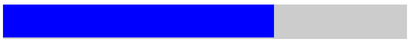
AAACACTTGGTGATATGAACACAAACGTTCAATATGACAAACCCATGCCAAGTAAAGAGAAAATGAAAACTGGTGATTGTTGCGGAAATCGTCCAGGATT	293 (0.005543%)	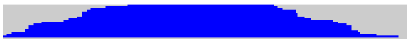
AAACGTTCAATATGACAAACCCATGCCAAGTAAAGAGAAAATGAAAACTGGTGATTGTTGCGGAAATCGTCCAGGATTCCTCGACCAGGACTTGAAATCG	4 (0.000076%)	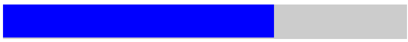
AAAGCCAAAGACTCATATGGACTTTGGCTACACCATGAAAGCTTTGAGAAGCAAGAAGAAGGTTGGTTAGTGTTTTGGAGTCGAATATGACTTGATGTCA	279 (0.005278%)	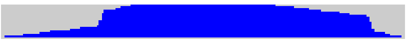
AAAGGCGTAAGAATTGTATCCTTGTTAGAAGACACAAAGC	199 (0.001506%)	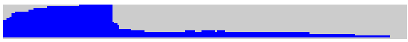
AAAGGGTGTTGGTCGATTAAGACAGCAGGACGGTGGTCATGGAAGTCGAAATCCGCTAAGGAGTGTGTAACAACTCACCTGCCGAATCAACTAGCCCCGA	16 (0.000303%)	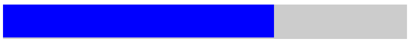
AAAGTTGGGAATTCGTTAAGGAGCTGTTGCTTTGTTAGTGTAGAAACACTTGTGTAGAATTGGGGATTGTTTTTTTTGGAGTGATTTAGGGGAGGGTCGA	11 (0.000208%)	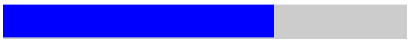
AAATACGGGCGAGAGACCGATAGCGAACAAGTACCGCGAGGTAAAGATGAAAAGGACTTTGAAAAGAGAGTCAAAGAGTGCTTGAAATTGTCGGGAGGGA	6 (0.000114%)	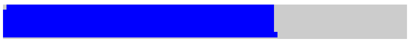
AAATCATCAATCGTTCCAACTAATCTACCGAAGTACTCGGCTAAGAAGAAAGAAGACGGACGAATCCGAGCCAAAGCCGTACAAGCGCGAGATACCTTCG	9 (0.000170%)	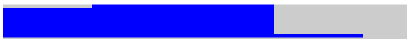
AAATCCTATGATGTTATCCCATGCTAATGTATCCAGAGCGTAGGCTTGCTTTGAGCACTCTAATTTCTTCAAAGTAACAGCGCCGGAGGCACGACCCGGC	35 (0.000662%)	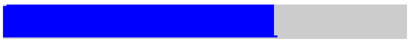
AAATCGTCGTCCCTCACCATCCTTTGCTGATGCGGGACGGAAGCTGGTCTCCCGTGTGTTACCGCACGCGGTTGGCCTAAATCCGAGCCAAGGACGCCTG	28 (0.000530%)	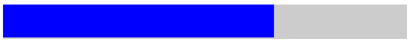
AAATTAGGTACGAAACACAGGCCCCGGAACTCATCATCGAGCGTAACATCGCCCGTGAATTAACTGAGAAGGATAGGTGGTAGGTAGTTCGATGCGCGAG	10 (0.000189%)	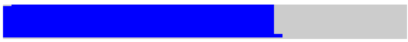
AAATTTCTGCCCTATCAACTTTCGATGGTAGGATAGTGGCCTACCATGGTGGTAACGGGTGACGGAGAATTAGGGTTCGATTCCGGAGAGGGAGCCTGAG	37 (0.000700%)	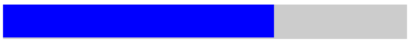
AACACAGGCCCCGGAACTCATCATCGAGCGTAACATCGCCCGTGAATTAACTGAGAAGGATAGGTGGTAGGTAGTTCGATGCGCGAGCATGGAGCCTACG	23 (0.000435%)	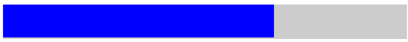
AACAGGGACAGTCGGGGGCATTCGTATTTCATAGTCAGAGGTGAAATTCTTGGATTTATGAAAGACGAACAACTGCGAAAGCATTTGCCAAGGATGTTTT	11 (0.000208%)	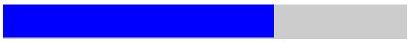
AACCCACAAAGGGTGTTGGTCGATTAAGACAGCAGGACGGTGGTCATGGAAGTCGAAATCCGCTAAGGAGTGTGTAACAACTCACCTGCCGAATCAACTA	4 (0.000076%)	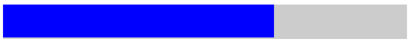
AACCGGGACGTGGCGGTTGACGGCAACGTTAGGGAGTCCGGAGACGTCGGCGGGGGCCTCGGGAAGAGTTATCTTTTCTGTTTAACAGCCTGCCCACCCT	3 (0.000057%)	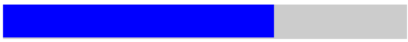
AACCTAGGCGAGACAAGGGTTCACATTTCGTTCATCACCCTTGGCCGGCTATCGAACAGCCGGACTCCCATCAAAAGATGGTTGCCAAGAACATCTTCGT	3 (0.000057%)	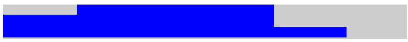
AACGAAGCACGCCCATCCAACCTAGGCGAGACAAGGGTTCACATTTCGTTCATCACCCTTGGCCGGCTATCGAACAGCCGGACTCCCATCAAAAGATGGT	21 (0.000397%)	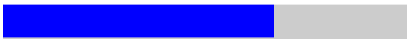
AACGGCGTGCCTCGGCATCAGCGTGCTCCGGGCGTCGGCCTGTGGGCTCCCCATTCGACCCGTCTTGAAACACGGACCAAGGAGTCTGACATGTGTGCGA	7 (0.000132%)	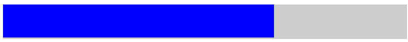
AACGGGCAGAGCCCGCGTCGACCTTTTATCTAATAAATGCGTCCCTTCCATAAGTCGGGGTTTGTTGCACGTATTAGCTCTAGAATTACTACGGTTATCC	8 (0.000151%)	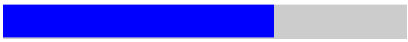
AACGTTAGGGAGTCCGGAGACGTCGGCGGGGGCCTCGGGAAGAGTTATCTTTTCTGTTTAACAGCCTGCCCACCCTGGAAACGGCTCAGCCGGAGGTAGG	3 (0.000057%)	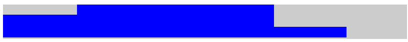
AACTAGCTACGTGGAGGCATCCCTTCACGGCCGGCTTCTTAGAGGGACTATGGCCGTTTAGGCCAAGGAAGTTTGAGGCAATAACAGGTCTGTGATGCCC	4 (0.000076%)	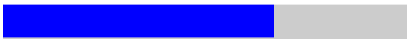
AACTTTCGATGGTAGGATAGTGGCCTACCATGGTGGTAACGGGTGACGGAGAATTAGGGTTCGATTCCGGAGAGGGAGCCTGAGAAACGGCTACCACATC	4 (0.000076%)	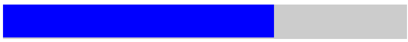
AAGAATTGTATCCTTGTTAGAAGACACAAAGCCAAAGACTCATATGGACTTTGGCTACACCATGAAAGCTTTGAGAAGCAAGAAGAAGGTTGGTTAGTGT	17 (0.000322%)	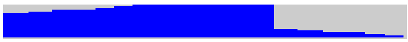
AAGACACAAAGCCAAAGACTCATATGGACTTTGGCTACACCATGAAAGCTTTGAGAAGCAAGAAGAAGGTTGGTTAGTGTTTTGGAGTCGAATATGACTT	528 (0.009989%)	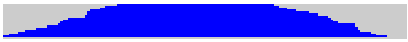
AAGACAGCAGGACGGTGGTCATGGAAGTCGAAATCCGCTAAGGAGTGTGTAACAACTCACCTGCCGAATCAACTAGCCCCGAAAATGGATGGCGCTTAAG	14 (0.000265%)	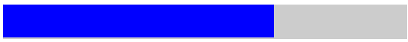
AAGAGCGTCGTGTAGGGAAAGAGTGTTCAGAGCCGTGTAG	11 (0.000083%)	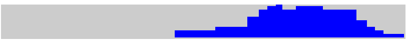
AAGATCGATC	204027 (0.385983%)	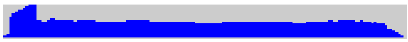
AAGCACGCCCATCCAACCTAGGCGAGACAAGGGTTCACATTTCGTTCATCACCCTTGGCCGGCTATCGAACAGCCGGACTCCCATCAAAAGATGGTTGCC	5 (0.000095%)	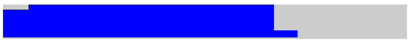
AAGCCAAAGACTCATATGGACTTTGGCTACACCATGAAAGCTTTGAGAAGCAAGAAGAAGGTTGGTTAGTGTTTTGGAGTCGAATATGACTTGATGTCAT	9 (0.000170%)	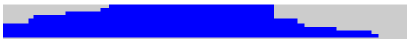
AAGCCTAAGTAGTGTTTCCTTGTTAGAACATACAAAGCCAAAGACTCATACGGACTTTGGCTACACCATGAAAGCTTTGAGAAGCTAGAAGAAGGTTGGT	1229 (0.023251%)	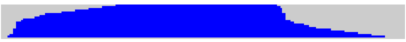

AAGCCTAAGTAGTGTTTCCTTGTTAGAAGACACAAAGCCAAAGACTCATATGGACTTTGGCTACACCATGA<br>AAGCTTTGAGAAGCAAGAAGAAGGTTGGT	1683 (0.031839%)	
AAGGAGCTGTTGCTTTGTTAGTGTAGAAACACTTGTGTAGAATTGGGGATTGTTTTTTTTGGAGTGATTTA<br>GGGGAGGGTCGAATCTTAGCGACAAAGGG	5 (0.000095%)	
AAGGCGTAAGAATTGTATCCTTGTTAGAAGACACAAAGCC	61 (0.000462%)	
AAGGGTGTTGGTCGATTAAGACAGCAGGACGGTGGTCATGGAAGTCGAAATCCGCTAAGGAGTGTGTAACA<br>ACTCACCTGCCGAATCAACTAGCCCCGAA	3 (0.000057%)	
AAGGGTTCACATTTCGTTCATCACCCTTGGCCGGCTTTCGAACAGCCGGACTCCCATCAAAAGATGGTTGC<br>CAAGAACATCTTCGTTACGGTTTGCTAAT	3 (0.000057%)	
AAGGTATCTCGCGCTTGTACGGCTTTGGCTCGGATTCGTCCGTCTTCTTTCTTCTTAGCCGAGTACTTCGG<br>TAGATTAGTTGGAACGATTGATGATTTTG	13 (0.000246%)	
AAGTAGTGTTTCCTTGTTAGAAGACACAAAGCCAAAGACTCATATGGACTTTGGCTACACCATGAAAGCTT<br>TGAGAAGCAAGAAGAAGGTTGGTTAGTGT	27 (0.000511%)	
AAGTCATATTCGACTCCAAAACACTAACCAACCTTCTTCTTGCTTCTCAAAGCTTTCATGGTGTAGCCAAA<br>GTCCATATGAGTCTTTGGCTTTGTGTCTT	128 (0.002422%)	
AAGTCTGGTGCCAGCAGCCGCGGTAATTCCAGCTCCAATAGCGTATATTTAAGTTGTTGCAGTTAAAAAGC<br>TCGTAGTTGAACCTTGGGATGGGTCGGCC	6 (0.000114%)	
AAGTTCTTATACTCAATCATACACATGACATCAAGTCATATTCGACTCCAAAACACTAACCAACCTTCTTC<br>TTGCTTCTCAAAGCTTTCATGGTGTAGCC	29 (0.000549%)	
AAGTTGTTATACTCAATCATACACATGACAACAAGTCATATTCGACTCCAAAACACTAACCAACCTTCTTC<br>TTGCTTCTCAAAGCTTTCATGGTGTAGCC	31 (0.000586%)	
AAGTTGTTATACTCAATCATACACATGACATCAAGTCATA	126 (0.000953%)	
AATACGGGCGAGAGACCGATAGCGAACAAGTACCGCGAGGTAAAGATGAAAAGGACTTTGAAAAGAGAGTC<br>AAAGAGTGCTTGAAATTGTCGGGAGGGAA	5 (0.000095%)	
AATCAGCTTCCTTGCGCCTTACGGGTTTACTCACCCGTTGACTCGCACACATGTCAGACTCCTTGGTCCGT<br>GTTTCAAGACGGGTCGAATGGGGAGCCCA	173 (0.003273%)	
AATCATACACATGACATCAAGTCATATTCGACTCCAAAACACTAACCAACCTTCTTCTTGCTTCTCAAAGC<br>TTTCATGGTGTAGCCAAAGTCCATATGAG	53 (0.001003%)	
AATCATCAATCGTTCCAACTAATCTACCGAAGTACTCGGCTAAGAAGAAAGAAGACGGACGAATCCGAGCC<br>AAAGCCGTACAAGCGCGAGATACCTTCGG	3 (0.000057%)	
AATCATCAGAGCAACGGGCAGAGCCCGCGTCGACCTTTTATCTAATAAATGCGTCCCTTCCATAAGTCGGG<br>GTTTGTTGCACGTATTAGCTCTAGAATTA	44 (0.000832%)	
AATCCTATGATGTTATCCCATGCTAATGTATCCAGAGCGTAGGCTTGCTTTGAGCACTCTAATTTCTTCAA<br>AGTAACAGCGCCGGAGGCACGACCCGGCC	39 (0.000738%)	
AATCGGTAGGAGCGACGGGCGGTGTGTACAAAGGGCAGGGACGTAGTCAACGCGAGCTGATGACTCGCGCT<br>TACTAGGAATTCCTCGTTGAAGACCAACA	157 (0.002970%)	
AATCGTCGTCCCTCACCATCCTTTGCTGATGCGGGACGGAAGCTGGTCTCCCGTGTGTTACCGCACGCGGT<br>TGGCCTAAATCCGAGCCAAGGACGCCTGG	7 (0.000132%)	
AATCGTTCCAACTAATCTACCGAAGTACTCGGCTAAGAAGAAAGAAGACGGACGAATCCGAGCCAAAGCCG<br>TACAAGCGCGAGATACCTTCGGGACAGCC	14 (0.000265%)	
AATGATTAACAGGGACAGTCGGGGGCATTCGTATTTCATAGTCAGAGGTGAAATTCTTGGATTTATGAAAG<br>ACGAACAACTGCGAAAGCATTTGCCAAGG	571 (0.010802%)	
AATGCTTTGTTTTAATTAAACAGTCGGATTCCCCTTGTCCGTACCAGTTCTGAGCTGACTGTTCGACGCCC<br>GGGGAAAGCTCCCGAGAGAGCCGTTCCCA	686 (0.012978%)	
AATGTATCCAGAGCGTAGGCTTGCTTTGAGCACTCTAATTTCTTCAAAGTAACAGCGCCGGAGGCACGACC<br>CGGCCAATTAAGACCAGGAGCGTATCGCC	5 (0.000095%)	
AATTAGGTACGAAACACAGGCCCCGGAACTCATCATCGAGCGTAACATCGCCCGTGAATTAACTGAGAAGG<br>ATAGGTGGTAGGTAGTTCGATGCGCGAGC	4 (0.000076%)	
AATTCCAGCTCCAATAGCGTATATTTAAGTTGTTGCAGTTAAAAAGCTCGTAGTTGAACCTTGGGATGGGT<br>CGGCCGGTCCGCCTTTGGTGTGCATTGGT	15 (0.000284%)	
AATTGTTGGTCTTCAACGAGGAATTCCTAGTAAGCGCGAGTCATCAGCTCGCGTTGACTACGTCCCTGCCC<br>TTTGTACACACCGCCCGTCGCTCCTACCG	1386 (0.026221%)	
AATTTCTGCCCTATCAACTTTCGATGGTAGGATAGTGGCCTACCATGGTGGTAACGGGTGACGGAGAATTA<br>GGGTTCGATTCCGGAGAGGGAGCCTGAGA	14 (0.000265%)	
ACAAACGTTCAATATGACAAACCCATGCCAAGTAAAGAGAAAATGAAAACTGGTGATTGTTGCGGAAATCG<br>TCCAGGATTCCTCGACCAGGACTTGAAAT	3 (0.000057%)	
ACAAAGCCAAAGACTCATATGGACTTTGGCTACACCATGAAAGCTTTGAGAAGCAAGAAGAAGGTTGGTTA<br>GTGTTTTGGAGTCGAATATGACTTGATGT	150 (0.002838%)	
ACAAAGGGTGTTGGTCGATTAAGACAGCAGGACGGTGGTCATGGAAGTCGAAATCCGCTAAGGAGTGTGTA<br>ACAACTCACCTGCCGAATCAACTAGCCCC	10 (0.000189%)	
ACAAATCGTCGTCCCTCACCATCCTTTGCTGATGCGGGACGGAAGCTGGTCTCCCGTGTGTTACCGCACGC<br>GGTTGGCCTAAATCCGAGCCAAGGACGCC	8 (0.000151%)	
ACAAGGGTTCACATTTCGTTCATCACCCTTGGCCGGCTATCGAACAGCCGGACTCCCATCAAAAGATGGTT<br>GCCAAGAACATCTTCGTTACGGTTTGCTA	4 (0.000076%)	
ACACAAAGCCAAAGACTCATATGGACTTTGGCTACACCATGAAAGCTTTGAGAAGCAAGAAGAAGGTTGGT<br>TAGTGTTTTGGAGTCGAATATGACTTGAT	148 (0.002800%)	
ACACAGGCCCCGGAACTCATCATCGAGCGTAACATCGCCCGTGAATTAACTGAGAAGGATAGGTGGTAGGT<br>AGTTCGATGCGCGAGCATGGAGCCTACGA	3 (0.000057%)	
ACACATGACATCAAGTCATATTCGACTCCAAAACACTAACCAACCTTCTTCTTGCTTCTCAAAGCTTTCAT<br>GGTGTAGCCAAAGTCCATATGAGTCTTTG	21 (0.000397%)	
ACATCAAGTCATATTCGACTCCAAAACACTAACCAACCTTCTTCTTGCTTCTCAAAGCTTTCATGGTGTAG<br>CCAAAGTCCATATGAGTCTTTGGCTTTGT	43 (0.000813%)	
ACATGACATCAAGTCATATTCGACTCCAAAACACTAACCAACCTTCTTCTTGCTTCTCAAAGCTTTCATGG<br>TGTAGCCAAAGTCCATATGAGTCTTTGGC	67 (0.001268%)	
ACATTGTCAGGTGGGGAGTTTGGCTGGGGCGGCACATCTGTTAAAAGATAACGCAGGTGTCCTAAGATGAG<br>CTCAACGAGAACAGAAATCTCGTGTGGAA	14 (0.000265%)	
ACCAGGGGTTGAAATCGTCGACCAGGTCCGAGACTTCATCGACCGGGTCCGAGGATTCGTCGACCAGGACG<br>GCCGGATGTCCGAGAAAAAAAAAATGTTGC	7 (0.000132%)	
ACCATCCTTTGCTGATGCGGGACGGAAGCTGGTCTCCCGTGTGTTACCGCACGCGGTTGGCCTAAATCCGA<br>GCCAAGGACGCCTGGAGCGTACCGACATG	10 (0.000189%)	
ACCCGCCGAAGCGAGCCTTGGGACCAAAAACAGGGGTTGTACCCCGCCTCCGATTCACGGAGTAAGTAAAA<br>TAACGTTAAAAGTAGTGGTATTTCACTTG	5 (0.000095%)	
ACCGGACACCACGCGACGTGCGGTGCTCTTCCAGCCGCTGGACCCTACCTCCGGCTGAGCCGTTTCCAGGG<br>TGGGCAGGCTGTTAAACAGAAAAGATAAC	5 (0.000095%)	

ACCGGGACGTGGCGGTTGACGGCAACGTTAGGGAGTCCGGAGACGTCGGCGGGGGCCTCGGGAAGAGTTATCTTTTCTGTTTAACAGCCTGCCCACCCTG	4 (0.000076%)	
ACCTAGGCGAGACAAGGGTTCACATTTCGTTCATCACCCTTGGCCGGCTATCGAACAGCCGGACTCCCATCAAAAGATGGTTGCCAAGAACATCTTCGTT	3 (0.000057%)	
ACCTCAGCCTGCTAACTAGCTACGTGGAGGCATCCCTTCACGGCCGGCTTCTTAGAGGGACTATGGCCGTTTAGGCCAAGGAAGTTTGAGGCAATAACAG	15 (0.000284%)	
ACGAAACACAGGCCCCGGAACTCATCATCGAGCGTAACATCGCCCGTGAATTAACTGAGAAGGATAGGTGGTAGGTAGTTCGATGCGCGAGCATGGAGCC	31 (0.000586%)	
ACGAAGCACGCCCATCCAACCTAGGCGAGACAAGGGTTCACATTTCGTTCATCACCCTTGGCCGGCTATCGAACAGCCGGACTCCCATCAAAAGATGGTT	9 (0.000170%)	
ACGCCCATCCAACCTAGGCGAGACAAGGGTTCACATTTCGTTCATCACCCTTGGCCGGCTTTCGAACAGCCGGACTCCCATCAAAAGATGGTTGCCAAGA	2021 (0.038234%)	
ACGCGCCTAACGGCGTGCCTCGGCATCAGCGTGCTCCGGGCGTCGGCCTGTGGGCTCCCCATTCGACCCGTCTTGAAACACGGACCAAGGAGTCTGACAT	23 (0.000435%)	
ACGCTTTCACGGTTCGTATTCGTACTGAAAATCAGAATCAAACGAGCTTTTACCCTTTTGTTCCACACGAGATTTCTGTTCTCGTTGAGCTCATCTTAGG	7 (0.000132%)	
ACGGCAACGTTAGGGAGTCCGGAGACGTCGGCGGGGGCCTCGGGAAGAGTTATCTTTTCTGTTTAACAGCCTGCCCACCCTGGAAACGGCTCAGCCGGAG	4 (0.000076%)	
ACGGCGTGCCTCGGCATCAGCGTGCTCCGGGCGTCGGCCTGTGGGCTCCCCATTCGACCCGTCTTGAAACACGGACCAAGGAGTCTGACATGTGTGCGAG	5 (0.000095%)	
ACGGCTTTGGCTCGGATTCGTCCGTCTTCTTTCTTCTTAGCCGAGTACTTCGGTAGATTAGTTGGAACGATTGATGATTTTGAGTTAATTGAACGTTCGG	11 (0.000208%)	
ACGGGCGAGAGACCGATAGCGAACAAGTACCGCGAGGTAAAGATGAAAAGGACTTTGAAAAGAGAGTCAAAGAGTGCTTGAAATTGTCGGGAGGGAAGCG	3 (0.000057%)	
ACGGTGGTCATGGAAGTCGAAATCCGCTAAGGAGTGTGTAACAACTCACCTGCCGAATCAACTAGCCCCGAAAATGGATGGCGCTTAAGCGCGCGACCTA	4 (0.000076%)	
ACGTGGGTGGTTCGCCGCCCGCGACGTCGCGAGAAGTCCACTAAACCTTATCATTTAGAGGAAGGAGAAGTCGTAACAAGGTTTCCGTAGGTGAACCTGC	5 (0.000095%)	
ACTACCTACCACCTATCCTTCTCAGTTAATTCACGGGCGATGTTACGCTCGATGATGAGTTCCGGGGCCTGTGTTTCGTACCTAATTTGAAGGAATTGTT	3 (0.000057%)	
ACTAGCTACGTGGAGGCATCCCTTCACGGCCGGCTTCTTAGAGGGACTATGGCCGTTTAGGCCAAGGAAGTTTGAGGCAATAACAGGTCTGTGATGCCCT	3 (0.000057%)	
ACTCAATCATACACATGACATCAAGTCATATTCGACTCCAAAACACTAACCAACCTTCTTCTTGCTTCTCAAAGCTTTCATGGTGTAGCCAAAGTCCATA	25 (0.000473%)	
ACTCCAAAACACTAACCAACCTTCTTCTTGCTTCTCAAAGCTTTCATGGTGTAGCCAAAGTCCATATGAGTCTTTGGCTTTGTGTCTTCTAACAAGGAAA	415 (0.007851%)	
ACTCCAAAACACTAACCAACCTTCTTCTTGCTTCTCAAAGCTTTCATGGTGTAGCCAAAGTCCATATGAGTCTTTGGCTTTGTGTCTTCTAACAAGGATA	10 (0.000189%)	
ACTCGGTCCTCCGGATTTTCAAGGGCCGCCGGGGGCGCACCGGACACCACGCGACGTGCGGTGCTCTTCCAGCCGCTGGACCCTACCTCCGGCTGAGCCG	15 (0.000284%)	
ACTTCCCTTGCCTACATTGTTCCATCGACCAGAGGCTGTTCACCTTGGAGACCTGATGCGGTTATGAGTACGACCGGGCGTGAGCGGCACTCGGTCCTCC	19 (0.000359%)	
ACTTGGTGATATGAACACAAACGTTCAATATGACAAACCCATGCCAAGTAAAGAGAAAATGAAAACTGGTGATTGTTGCGGAAATCGTCCAGGATTCCTC	5 (0.000095%)	
AGAAGACACAAAGCCAAAGACTCATATGGACTTTGGCTACACCATGAAAGCTTTGAGAAGCAAGAAGAAGGTTGGTTAGTGTTTTGGAGTCGAATATGAC	70 (0.001324%)	
AGAATTGTATCCTTGTTAGAAGACACAAAGCCAAAGACTCATATGGACTTTGGCTACACCATGAAAGCTTTGAGAAGCAAGAAGAAGGTTGGTTAGTGTT	4 (0.000076%)	
AGACAAGGGTTCACATTTCGTTCATCACCCTTGGCCGGCTTTCGAACAGCCGGACTCCCATCAAAAGATGGTTGCCAAGAACATCTTCGTTACGGTTTGC	3 (0.000057%)	
AGACACAAAGCCAAAGACTCATATGGACTTTGGCTACACCATGAAAGCTTTGAGAAGCAAGAAGAAGGTTGGTTAGTGTTTTGGAGTCGAATATGACTTG	41 (0.000776%)	
AGACAGCAGGACGGTGGTCATGGAAGTCGAAATCCGCTAAGGAGTGTGTAACAACTCACCTGCCGAATCAACTAGCCCCGAAAATGGATGGCGCTTAAGC	10 (0.000189%)	
AGACGTCGGCGGGGGCCTCGGGAAGAGTTATCTTTTCTGTTTAACAGCCTGCCCACCCTGGAAACGGCTCAGCCGGAGGTAGGGTCCAGCGGCTGGAAGA	3 (0.000057%)	
AGAGCAACGGGCAGAGCCCGCGTCGACCTTTTATCTAATAAATGCGTCCCTTCCATAAGTCGGGGTTTGTTGCACGTATTAGCTCTAGAATTACTACGGT	38 (0.000719%)	
AGAGCCCGCGTCGACCTTTTATCTAATAAATGCGTCCCTTCCATAAGTCGGGGTTTGTTGCACGTATTAGCTCTAGAATTACTACGGTTATCCGAGTAGT	9 (0.000170%)	
AGAGCGTAGGCTTGCTTTGAGCACTCTAATTTCTTCAAAGTAACAGCGCCGGAGGCACGACCCGGCCAATTAAGACCAGGAGCGTATCGCCGACCGAAGG	14 (0.000265%)	
AGAGCGTCGTGTAGGGAAAGAGTGTTCAGAGCCGTGTAGA	13 (0.000098%)	
AGAGGCTGTTCACCTTGGAGACCTGATGCGGTTATGAGTACGACCGGGCGTGAGCGGCACTCGGTCCTCCGGATTTTCAAGGGCCGCCGGGGGCGCACCG	148 (0.002800%)	
AGATCGGAAGAGCGTCGTGTAGGGAAAGAGTGTTCAGAGC	19671 (0.148856%)	
AGCAACGGGCAGAGCCCGCGTCGACCTTTTATCTAATAAATGCGTCCCTTCCATAAGTCGGGGTTTGTTGCACGTATTAGCTCTAGAATTACTACGGTTA	3 (0.000057%)	
AGCACGCCCATCCAACCTAGGCGAGACAAGGGTTCACATTTCGTTCATCACCCTTGGCCGGCTATCGAACAGCCGGACTCCCATCAAAAGATGGTTGCCA	12 (0.000227%)	
AGCACGCGCCTAACGGCGTGCCTCGGCATCAGCGTGCTCCGGGCGTCGGCCTGTGGGCTCCCCATTCGACCCGTCTTGAAACACGGACCAAGGAGTCTGA	30 (0.000568%)	
AGCAGCCGCGGTAATTCCAGCTCCAATAGCGTATATTTAAGTTGTTGCAGTTAAAAAGCTCGTAGTTGAACCTTGGGATGGGTCGGCCGGTCCGCCTTTG	8 (0.000151%)	
AGCAGGACGGTGGTCATGGAAGTCGAAATCCGCTAAGGAGTGTGTAACAACTCACCTGCCGAATCAACTAGCCCCGAAAATGGATGGCGCTTAAGCGCGC	6 (0.000114%)	
AGCCAAAGACTCATATGGACTTTGGCTACACCATGAAAGCTTTGAGAAGCAAGAAGAAGGTTGGTTAGTGTTTTGGAGTCGAATATGACTTGATGTCATG	10 (0.000189%)	
AGCCCGCGTCGACCTTTTATCTAATAAATGCGTCCCTTCCATAAGTCGGGGTTTGTTGCACGTATTAGCTCTAGAATTACTACGGTTATCCGAGTAGTAG	3 (0.000057%)	
AGCCGCGGTAATTCCAGCTCCAATAGCGTATATTTAAGTTGTTGCAGTTAAAAAGCTCGTAGTTGAACCTTGGGATGGGTCGGCCGGTCCGCCTTTGGTG	7 (0.000132%)	
AGCCTAAGTAGTGTTTCCTTGTTAGAAGACACAAAGCCAAAGACTCATATGGACTTTGGCTACACCATGAAAGCTTTGAGAAGCAAGAAGAAGGTTGGTT	36 (0.000681%)	

AGCCTGCTAACTAGCTACGTGGAGGCATCCCTTCACGGCCGGCTTCTTAGAGGGACTATGGCCGTTTAGGC CAAGGAAGTTTGAGGCAATAACAGGTCTG	8 (0.000151%)	
AGCGACGGGCGGTGTGTACAAAGGGCAGGGACGTAGTCAACGCGAGCTGATGACTCGCGCTTACTAGGAAT TCCTCGTTGAAGACCAACAATTGCAATGA	4 (0.000076%)	
AGCGAGGTGTGAGTGTCGCCCATGGGCATCGACACCTTGCGGCTAGGAACTGGAACGAGACGGGTGGCAAA GATTTCGAGTAGCACTTCATACTACCGTG	4 (0.000076%)	
AGCGGCACTCGGTCCTCCGGATTTTCAAGGGCCGCCGGGGGCGCACCGGACACCACGCGACGTGCGGTGCT CTTCCAGCCGCTGGACCCTACCTCCGGCT	6 (0.000114%)	
AGCGTAGGCTTGCTTTGAGCACTCTAATTTCTTCAAAGTAACAGCGCCGGAGGCACGACCCGGCCAATTAA GACCAGGAGCGTATCGCCGACCGAAGGGA	7 (0.000132%)	
AGCGTCGTGTAGGGAAAGAGTGTTCAGAGCCGTGTAGATC	8 (0.000061%)	
AGCTAGTGTTCGTAGGCTCCATGCTCGCGCATCGAACTACCTACCACCTATCCTTCTCAGTTAATTCACGG GCGATGTTACGCTCGATGATGAGTTCCGG	10 (0.000189%)	
AGCTCCAATAGCGTATATTTAAGTTGTTGCAGTTAAAAAGCTCGTAGTTGAACCTTGGGATGGGTCGGCCG GTCCGCCTTTGGTGTGCATTGGTCGGCTT	5 (0.000095%)	
AGCTCGTGTAAAGTTGGGAATTCGTTAAGGAGCTGTTGCTTTGTTAGTGTAGAAACACTTGTGTAGAATTG GGGATTGTTTTTTTTGGAGTGATTTAGGG	10 (0.000189%)	
AGCTTCCTTGCGCCTTACGGGTTTACTCACCCGTTGACTCGCACACATGTCAGACTCCTTGGTCCGTGTTT CAAGACGGGTCGAATGGGGAGCCCACAGG	30 (0.000568%)	
AGGACATTGTCAGGTGGGGAGTTTGGCTGGGGCGGCACATCTGTTAAAAGATAACGCAGGTGTCCTAAGAT GAGCTCAACGAGAACAGAAATCTCGTGTG	179 (0.003386%)	
AGGACGGTGGTCATGGAAGTCGAAATCCGCTAAGGAGTGTGTAACAACTCACCTGCCGAATCAACTAGCCC CGAAAATGGATGGCGCTTAAGCGCGCGAC	27 (0.000511%)	
AGGAGCGACGGGCGGTGTGTACAAAGGGCAGGGACGTAGTCAACGCGAGCTGATGACTCGCGCTTACTAGG AATTCCTCGTTGAAGACCAACAATTGCAA	17 (0.000322%)	
AGGAGCTGTTGCTTTGTTAGTGTAGAAACACTTGTGTAGAATTGGGGATTGTTTTTTTTGGAGTGATTTAG GGGAGGGTCGAATCTTAGCGACAAAGGGC	11 (0.000208%)	
AGGCGAGACAAGGGTTCACATTTCGTTCATCACCCTTGGCCGGCTATCGAACAGCCGGACTCCCATCAAAA GATGGTTGCCAAGAACATCTTCGTTACGG	5 (0.000095%)	
AGGCGTAAGAATTGTATCCTTGTTAGAAGACACAAAGCCA	116 (0.000878%)	
AGGCTCCATGCTCGCGCATCGAACTACCTACCACCTATCCTTCTCAGTTAATTCACGGGCGATGTTACGCT CGATGATGAGTTCCGGGGCCTGTGTTTCG	5 (0.000095%)	
AGGCTGTCCCGAGTGTGAGCGAGGTGTGAGTGTCGCCCATGGGCATCGACACCTTGCGGCTAGGAACTGGA ACGAGACGGGTAGCAAAGATTTCGAGTAG	1796 (0.033977%)	
AGGCTGTCCCGAGTGTGAGCGAGGTGTGAGTGTCGCCCATGGGCATCGACACCTTGCGGCTAGGAACTGGA ACGAGACGGGTGGCAAAGATTTCGAGTAG	15 (0.000284%)	
AGGCTGTTCACCTTGGAGACCTGATGCGGTTATGAGTACGACCGGGCGTGAGCGGCACTCGGTCCTCCGGA TTTTCAAGGGCCGCCGGGGGCGCACCGGA	33 (0.000624%)	
AGGGAAAGAGTGTTCAGAGCCGTGTAGATCTCGGTGGTCG	52 (0.000393%)	
AGGGACAGTCGGGGGCATTCGTATTTCATAGTCAGAGGTGAAATTCTTGGATTTATGAAAGACGAACAACT GCGAAAGCATTTGCCAAGGATGTTTTCAT	10 (0.000189%)	
AGGGAGTCCGGAGACGTCGGCGGGGGCCTCGGGAAGAGTTATCTTTTCTGTTTAACAGCCTGCCCACCCTG GAAACGGCTCAGCCGGAGGTAGGGTCCAG	6 (0.000114%)	
AGGGCAAGTCTGGTGCCAGCAGCCGCGGTAATTCCAGCTCCAATAGCGTATATTTAAGTTGTTGCAGTTAA AAAGCTCGTAGTTGAACCTTGGGATGGGT	22 (0.000416%)	
AGGGTGTTGGTCGATTAAGACAGCAGGACGGTGGTCATGGAAGTCGAAATCCGCTAAGGAGTGTGTAACAA CTCACCTGCCGAATCAACTAGCCCCGAAA	13 (0.000246%)	
AGGTACGAAACACAGGCCCCGGAACTCATCATCGAGCGTAACATCGCCCGTGAATTAACTGAGAAGGATAG GTGGTAGGTAGTTCGATGCGCGAGCATGG	3 (0.000057%)	
AGGTATCTCGCGCTTGTACGGCTTTGGCTCGGATTCGTCCGTCTTCTTTCTTCTTAGCCGAGTACTTCGGT AGATTAGTTGGAACGATTGATGATTTTGA	6 (0.000114%)	
AGGTGTGAGTGTCGCCCATGGGCATCGACACCTTGCGGCTAGGAACTGGAACGAGACGGGTGGCAAAGATT TCGAGTAGCACTTCATACTACCGTGGGTT	3 (0.000057%)	
AGTAGTCATATGCTTGTCTCAAAGATTAAGCCATGCATGTGTAAGTATGAACGAATTCAGACTGTGAAACT GCGAATGGCTCATTAAATCAGTTATAGTT	2227 (0.042131%)	
AGTAGTGTTTCCTTGTTAGAAGACACAAAGCCAAAGACTCATATGGACTTTGGCTACACCATGAAAGCTTT GAGAAGCAAGAAGAAGGTTGGTTAGTGTT	23 (0.000435%)	
AGTATAAGAACTTAAACCGCAACCGCATCTTATAAGCCTAAGTAGTGTTTCCTTGTTAGAAGACACAAAGC CAAAGACTCATATGGACTTTGGCTACACC	6 (0.000114%)	
AGTCATATGCTTGTCTCAAAGATTAAGCCATGCATGTGTAAGTATGAACGAATTCAGACTGTGAAACTGCG AATGGCTCATTAAATCAGTTATAGTTTGT	14 (0.000265%)	
AGTCATATTCGACTCCAAAACACTAACCAACCTTCTTCTTGCTTCTCAAAGCTTTCATGGTGTAGCCAAAG TCCATATGAGTCTTTGGCTTTGTGTCTTC	5 (0.000095%)	
AGTCGAATATGACTTGATGTCATGTGTATGATTGAGTATA	1222 (0.009247%)	
AGTCGGGGGCATTCGTATTTCATAGTCAGAGGTGAAATTCTTGGATTTATGAAAGACGAACAACTGCGAAA GCATTTGCCAAGGATGTTTTCATTAATCA	3 (0.000057%)	
AGTGTATCCTTGTTAGAAGACACAAAGCCAAAGACTCATA	8087 (0.061197%)	
AGTGTTCGTAGGCTCCATGCTCGCGCATCGAACTACCTACCACCTATCCTTCTCAGTTAATTCACGGGCGA TGTTACGCTCGATGATGAGTTCCGGGGCC	262 (0.004957%)	
AGTGTTTCCTTGTTAGAAGACACAAAGCCAAAGACTCATATGGACTTTGGCTACACCATGAAAGCTTTGAG AAGCAAGAAGAAGGTTGGTTAGTGTTTTG	12 (0.000227%)	
AGTGTTTTGGAGTCGAATATGACTTGATGTCATGTGTATG	204 (0.001544%)	
AGTTATCATGAATCATCAGAGCAACGGGCAGAGCCCGCGTCGACCTTTTATCTAATAAATGCGTCCCTTCC ATAAGTCGGGGTTTGTTGCACGTATTAGC	19 (0.000359%)	
AGTTCTCGGACAAAAATTGCTGAGTGGCCGAGAAGAATGGGCGTGTCATGCGTGGGCTGACATGGATTCTT CGAGGCCTAGGGGTGGCGGTATATAACTT	5 (0.000095%)	
AGTTCTTATACTCAATCATACACATGACATCAAGTCATATTCGACTCCAAAACACTAACCAACCTTCTTCT TGCTTCTCAAAGCTTTCATGGTGTAGCCA	19 (0.000359%)	
AGTTGTTATACTCAATCATACACATGACAACAAGTCATATTCGACTCCAAAACACTAACCAACCTTCTTCT TGCTTCTCAAAGCTTTCATGGTGTAGCCA	15 (0.000284%)	

AGTTGTTATACTCAATCATACACATGACATCAAGTCATAT	98 (0.000742%)
AGTTTTTTCAGCAGTTCTCGGACAAAAATTGCTGAGTGGCCGAGAAGAATGGGCGTGTCATGCGTGGGCTG<br>ACATGGATTCTTCGAGGCCTAGGGGTGGC	3 (0.000057%)
ATAAAAGTGTCGGAGTTTTTTCAGCAGTTCTCGGACAAAAATTGCTGAGTGGCCGAGAAGAATGGGCGTGT<br>CATGCGTGGGCTGACATGGATTCTTCGAG	153 (0.002894%)
ATAAGAACTTAAACCGCAACCGCATCTTATAAGCCTAAGTAGTGTTTCCTTGTTAGAAGACACAAAGCCAA<br>AGACTCATATGGACTTTGGCTACACCATG	26 (0.000492%)
ATACACATGACATCAAGTCATATTCGACTCCAAAACACTAACCAACCTTCTTCTTGCTTCTCAAAGCTTTC<br>ATGGTGTAGCCAAAGTCCATATGAGTCTT	272 (0.005146%)
ATACGGGCGAGAGACCGATAGCGAACAAGTACCGCGAGGTAAAGATGAAAAGGACTTTGAAAAGAGAGTCA<br>AAGAGTGCTTGAAATTGTCGGGAGGGAAG	17 (0.000322%)
ATACTCAATCATACACATGACATCAAGTCATATTCGACTCCAAAACACTAACCAACCTTCTTCTTGCTTCT<br>CAAAGCTTTCATGGTGTAGCCAAAGTCCA	30 (0.000568%)
ATAGCTAGTGTTCGTAGGCTCCATGCTCGCGCATCGAACTACCTACCACCTATCCTTCTCAGTTAATTCAC<br>GGGCGATGTTACGCTCGATGATGAGTTCC	5 (0.000095%)
ATATGCTTGTCTCAAAGATTAAGCCATGCATGTGTAAGTATGAACGAATTCAGACTGTGAAACTGCGAATG<br>GCTCATTAAATCAGTTATAGTTTGTTTGA	55 (0.001041%)
ATATTCGACTCCAAAACACTAACCAACCTTCTTCTTGCTTCTCAAAGCTTTCATGGTGTAGCCAAAGTCCA<br>TATGAGTCTTTGGCTTTGTGTCTTCTAAC	151 (0.002857%)
ATATTCGACTCCAAAACACTAACCAACCTTCTTCTTGCTTCTCAAAGCTTTCATGGTGTAGCCAAAGTCCA<br>TATGAGTCTTTGGCTTTGTGTCTTTTAAC	59 (0.001116%)
ATATTTAAGTTGTTGCAGTTAAAAAGCTCGTAGTTGAACCTTGGGATGGGTCGGCCGGTCCGCCTTTGGTG<br>TGCATTGGTCGGCTTGTCCCTTCGGTCGG	7 (0.000132%)
ATCAACTTTCGATGGTAGGATAGTGGCCTACCATGGTGGTAACGGGTGACGGAGAATTAGGGTTCGATTCC<br>GGAGAGGGAGCCTGAGAAACGGCTACCAC	9 (0.000170%)
ATCAAGTCATATTCGACTCCAAAACACTAACCAACCTTCTTCTTGCTTCTCAAAGCTTTCATGGTGTAGCC<br>AAAGTCCATATGAGTCTTTGGCTTTGTGT	1473 (0.027867%)
ATCAATCGTTCCAACTAATCTACCGAAGTACTCGGCTAAGAAGAAAGAAGACGGACGAATCCGAGCCAAAG<br>CCGTACAAGCGCGAGATACCTTCGGGACA	4 (0.000076%)
ATCAGAGCAACGGGCAGAGCCCGCGTCGACCTTTTATCTAATAAATGCGTCCCTTCCATAAGTCGGGGTTT<br>GTTGCACGTATTAGCTCTAGAATTACTAC	32 (0.000605%)
ATCAGCGTGCTCCGGGCGTCGGCCTGTGGGCTCCCCATTCGACCCGTCTTGAAACACGGACCAAGGAGTCT<br>GACATGTGTGCGAGTCAACGGGTGAGTAA	5 (0.000095%)
ATCAGCTTCCTTGCGCCTTACGGGTTTACTCACCCGTTGACTCGCACACATGTCAGACTCCTTGGTCCGTG<br>TTTCAAGACGGGTCGAATGGGGAGCCCAC	47 (0.000889%)
ATCATACACATGACATCAAGTCATATTCGACTCCAAAACACTAACCAACCTTCTTCTTGCTTCTCAAAGCT<br>TTCATGGTGTAGCCAAAGTCCATATGAGT	29 (0.000549%)
ATCATCAGAGCAACGGGCAGAGCCCGCGTCGACCTTTTATCTAATAAATGCGTCCCTTCCATAAGTCGGGG<br>TTTGTTGCACGTATTAGCTCTAGAATTAC	15 (0.000284%)
ATCATGAATCATCAGAGCAACGGGCAGAGCCCGCGTCGACCTTTTATCTAATAAATGCGTCCCTTCCATAA<br>GTCGGGGTTTGTTGCACGTATTAGCTCTA	62 (0.001173%)
ATCATTCAAATTTCTGCCCTATCAACTTTCGATGGTAGGATAGTGGCCTACCATGGTGGTAACGGGTGACG<br>GAGAATTAGGGTTCGATTCCGGAGAGGGA	4 (0.000076%)
ATCCAACCTAGGCGAGACAAGGGTTCACATTTCGTTCATCACCCTTGGCCGGCTATCGAACAGCCGGACTC<br>CCATCAAAAGATGGTTGCCAAGAACATCT	25 (0.000473%)
ATCCAACCTAGGCGAGACAAGGGTTCACATTTCGTTCATCACCCTTGGCCGGCTTTCGAACAGCCGGACTC<br>CCATCAAAAGATGGTTGCCAAGAACATCT	27 (0.000511%)
ATCCAGAGCGTAGGCTTGCTTTGAGCACTCTAATTTCTTCAAAGTAACAGCGCCGGAGGCACGACCCGGCC<br>AATTAAGACCAGGAGCGTATCGCCGACCG	15 (0.000284%)
ATCCATTACATTTTATCGGTCGCTCTTGTCCGGAAGCTGTAGATGACCCAAAGTCCATATAGCGACCCCAG<br>GTCAGGCGGGATTACCCGCTGAGTTTAAG	3 (0.000057%)
ATCCCATGCTAATGTATCCAGAGCGTAGGCTTGCTTTGAGCACTCTAATTTCTTCAAAGTAACAGCGCCGG<br>AGGCACGACCCGGCCAATTAAGACCAGGA	11 (0.000208%)
ATCCCGCCAATCAGCTTCCTTGCGCCTTACGGGTTTACTCACCCGTTGACTCGCACACATGTCAGACTCCT<br>TGGTCCGTGTTTCAAGACGGGTCGAATGG	10 (0.000189%)
ATCCGTCGAGTTATCATGAATCATCAGAGCAACGGGCAGAGCCCGCGTCGACCTTTTATCTAATAAATGCG<br>TCCCTTCCATAAGTCGGGGTTTGTTGCAC	9 (0.000170%)
ATCCTATGATGTTATCCCATGCTAATGTATCCAGAGCGTAGGCTTGCTTTGAGCACTCTAATTTCTTCAAA<br>GTAACAGCGCCGGAGGCACGACCCGGCCA	37 (0.000700%)
ATCCTTGTTAGAAGACACAAAGCCAAAGACTCATATGGACTTTGGCTACACCATGAAAGCTTTGAGAAGCA<br>AGAAGAAGGTTGGTTAGTGTTTTGGAGTC	10 (0.000189%)
ATCCTTTGCTGATGCGGGACGGAAGCTGGTCTCCCGTGTGTTACCGCACGCGGTTGGCCTAAATCCGAGCC<br>AAGGACGCCTGGAGCGTACCGACATGCGG	4 (0.000076%)
ATCGAAATCCTATGATGTTATCCCATGCTAATGTATCCAGAGCGTAGGCTTGCTTTGAGCACTCTAATTTC<br>TTCAAAGTAACAGCGCCGGAGGCACGACC	16 (0.000303%)
ATCGAACTACCTACCACCTATCCTTCTCAGTTAATTCACGGGCGATGTTACGCTCGATGATGAGTTCCGGG<br>GCCTGTGTTTCGTACCTAATTTGAAGGAA	5 (0.000095%)
ATCGACACCTTGCGGCTAGGAACTGGAACGAGACGGGTGGCAAAGATTTCGAGTAGCACTTCATACTACCG<br>TGGGTTTTTTAAACCTTCCGAGTTTTGTT	7 (0.000132%)
ATCGACCCGCCGAAGCGAGCCTTGGGACCAAAAACAGGGGTTGTACCCCGCCTCCGATTCACGGAGTAAGT<br>AAAATAACGTTAAAAGTAGTGGTATTTCA	4 (0.000076%)
ATCGATCCATTACATTTTATCGGTCGCTCTTGTCCGGAAGCTGTAGATGACCCAAAGTCCATATAGCGACC<br>CCAGGTCAGGCGGGATTACCCGCTGAGTT	8 (0.000151%)
ATCGATCCCGCCAATCAGCTTCCTTGCGCCTTACGGGTTTACTCACCCGTTGACTCGCACACATGTCAGAC<br>TCCTTGGTCCGTGTTTCAAGACGGGTCGA	1263 (0.023894%)
ATCGATCCGTAACTTCGGGAAAAGGATTGGCTCTGAGGGCTGGGCTCGGGGGTCCCAGTTCCGAACCCGTC<br>GGCTGTCAGCGGACTGCTCGAGCTGCTTC	32 (0.000605%)
ATCGATCCGTCGAGTTATCATGAATCATCAGAGCAACGGGCAGAGCCCGCGTCGACCTTTTATCTAATAAA<br>TGCGTCCCTTCCATAAGTCGGGGTTTGTT	23 (0.000435%)
ATCGATCGACCCGCCGAAGCGAGCCTTGGGACCAAAAACAGGGGTTGTACCCCGCCTCCGATTCACGGAGT<br>AAGTAAAATAACGTTAAAAGTAGTGGTAT	12 (0.000227%)
ATCGATCTCATGTGTATGATTGAGTATAAGAACTTAAACCGCAACCGCATCTTATAAGCCTAAGTAGTGTT<br>TCCTTGTTAGAAGACACAAAGCCAAAGAC	12 (0.000227%)
ATCGATCTTAAAGGCGTAAGAATTGTATCCTTGTTAAAAGACACAAAGCCAAAGACTCATATGGACTTTGG<br>CTACACCATGAAAGCTTTGAGAAGCAAGA	17 (0.000322%)

ATCGATCTTCTGAGAAGGGTTCGAGTGTGAGCATGCCTGTCGGGACCCGAAAGATGGTGAACTATGCCTGAGCGGGGTAAAGCCAGAGGAAACTCTGGTG	18 (0.000341%)
ATCGGAAGAGCGTCGTGTAGGGAAAGAGTGTTCAGAGCCG	19 (0.000144%)
ATCGGTAGGAGCGACGGGCGGTGTGTACAAAGGGCAGGGACGTAGTCAACGCGAGCTGATGACTCGCGCTTACTAGGAATTCCTCGTTGAAGACCAACAA	83 (0.001570%)
ATCGTCGTCCCTCACCATCCTTTGCTGATGCGGGACGGAAGCTGGTCTCCCGTGTGTTACCGCACGCGGTTGGCCTAAATCCGAGCCAAGGACGCCTGGA	4 (0.000076%)
ATCTCATGTGTATGATTGAGTATAAGAACTTAAACCGCAACCGCATCTTATAAGCCTAAGTAGTGTTTCCTTGTTAGAAGACACAAAGCCAAAGACTCAT	7 (0.000132%)
ATCTCGCGCTTGTACGGCTTTGGCTCGGATTCGTCCGTCTTCTTTCTTCTTAGCCGAGTACTTCGGTAGATTAGTTGGAACGATTGATGATTTTGAGTTA	4 (0.000076%)
ATCTTAAAGGCGTAAGAATTGTATCCTTGTTAAAAGACACAAAGCCAAAGACTCATATGGACTTTGGCTACACCATGAAAGCTTTGAGAAGCAAGAAGAA	47 (0.000889%)
ATCTTAAAGGCGTAAGAATTGTATCCTTGTTAGAAGACAC	1325 (0.010027%)
ATCTTCTGAGAAGGGTTCGAGTGTGAGCATGCCTGTCGGGACCCGAAAGATGGTGAACTATGCCTGAGCGGGGTAAAGCCAGAGGAAACTCTGGTGGAAG	3 (0.000057%)
ATGAACACAAACGTTCAATATGACAAACCCATGCCAAGTAAAGAGAAAATGAAAACTGGTGATTGTTGCGGAAATCGTCCAGGATTCCTCGACCAGGACT	11 (0.000208%)
ATGAATCATCAGAGCAACGGGCAGAGCCCGCGTCGACCTTTTATCTAATAAATGCGTCCCTTCCATAAGTCGGGGTTTGTTGCACGTATTAGCTCTAGAA	71 (0.001343%)
ATGACATCAAGTCATATTCGACTCCAAAACACTAACCAACCTTCTTCTTGCTTCTCAAAGCTTTCATGGTGTAGCCAAAGTCCATATGAGTCTTTGGCTT	117 (0.002213%)
ATGATGTTATCCCATGCTAATGTATCCAGAGCGTAGGCTTGCTTTGAGCACTCTAATTTCTTCAAAGTAACAGCGCCGGAGGCACGACCCGGCCAATTAA	63 (0.001192%)
ATGATTAACAGGGACAGTCGGGGGCATTCGTATTTCATAGTCAGAGGTGAAATTCTTGGATTTATGAAAGACGAACAACTGCGAAAGCATTTGCCAAGGA	93 (0.001759%)
ATGATTGAGTATAAGAACTTAAACCGCAACCGCATCTTATAAGCCTAAGTAGTGTTTCCTTGTTAGAAGACACAAAGCCAAAGACTCATATGGACTTTGG	29 (0.000549%)
ATGCTAATGTATCCAGAGCGTAGGCTTGCTTTGAGCACTCTAATTTCTTCAAAGTAACAGCGCCGGAGGCACGACCCGGCCAATTAAGACCAGGAGCGTA	14 (0.000265%)
ATGCTTGTCTCAAAGATTAAGCCATGCATGTGTAAGTATGAACGAATTCAGACTGTGAAACTGCGAATGGCTCATTAAATCAGTTATAGTTTGTTTGATG	14 (0.000265%)
ATGCTTTGTTTTAATTAAACAGTCGGATTCCCCTTGTCCGTACCAGTTCTGAGCTGACTGTTCGACGCCCGGGGAAAGCTCCCGAGAGAGCCGTTCCCAG	4 (0.000076%)
ATGGAAGTCGAAATCCGCTAAGGAGTGTGTAACAACTCACCTGCCGAATCAACTAGCCCCGAAAATGGATGGCGCTTAAGCGCGCGACCTATACCCGGCC	33 (0.000624%)
ATGGCCTCTGTGCTGGCGACGCATCATTCAAATTTCTGCCCTATCAACTTTCGATGGTAGGATAGTGGCCTACCATGGTGGTAACGGGTGACGGAGAATT	381 (0.007208%)
ATGGGCATCGACACCTTGCGGCTAGGAACTGGAACGAGACGGGTGGCAAAGATTTCGAGTAGCACTTCATACTACCGTGGGTTTTTTAAACCTTCCGAGT	8 (0.000151%)
ATGTATCCAGAGCGTAGGCTTGCTTTGAGCACTCTAATTTCTTCAAAGTAACAGCGCCGGAGGCACGACCCGGCCAATTAAGACCAGGAGCGTATCGCCG	5 (0.000095%)
ATGTGTAAGTATGAACGAATTCAGACTGTGAAACTGCGAATGGCTCATTAAATCAGTTATAGTTTGTTTGATGGTAACTACTACTCGGATAACCGTAGTA	188 (0.003557%)
ATGTGTATGATTGAGTATAAGAACTTAAACCGCAACCGCATCTTATAAGCCTAAGTAGTGTTTCCTTGTTAGAAGACACAAAGCCAAAGACTCATATGGA	13 (0.000246%)
ATGTTATCCCATGCTAATGTATCCAGAGCGTAGGCTTGCTTTGAGCACTCTAATTTCTTCAAAGTAACAGCGCCGGAGGCACGACCCGGCCAATTAAGAC	13 (0.000246%)
ATTAACAGGGACAGTCGGGGGCATTCGTATTTCATAGTCAGAGGTGAAATTCTTGGATTTATGAAAGACGAACAACTGCGAAAGCATTTGCCAAGGATGT	99 (0.001873%)
ATTAAGACAGCAGGACGGTGGTCATGGAAGTCGAAATCCGCTAAGGAGTGTGTAACAACTCACCTGCCGAATCAACTAGCCCCGAAAATGGATGGCGCTT	21 (0.000397%)
ATTAGGTACGAAACACAGGCCCCGGAACTCATCATCGAGCGTAACATCGCCCGTGAATTAACTGAGAAGGATAGGTGGTAGGTAGTTCGATGCGCGAGCA	13 (0.000246%)
ATTAGTCTTTCGCCCCTATACCCAAGTCAGACGAACGATTTGCACGTCAGTATCGCTGCGGGCTTCCACCAGAGTTTCCTCTGGCTTTACCCCGCTCAGG	24 (0.000454%)
ATTCAAATTTCTGCCCTATCAACTTTCGATGGTAGGATAGTGGCCTACCATGGTGGTAACGGGTGACGGAGAATTAGGGTTCGATTCCGGAGAGGGAGCC	12 (0.000227%)
ATTCAATCGGTAGGAGCGACGGGCGGTGTGTACAAAGGGCAGGGACGTAGTCAACGCGAGCTGATGACTCGCGCTTACTAGGAATTCCTCGTTGAAGACC	1057 (0.019997%)
ATTCCAGCTCCAATAGCGTATATTTAAGTTGTTGCAGTTAAAAAGCTCGTAGTTGAACCTTGGGATGGGTCGGCCGGTCCGCCTTTGGTGTGCATTGGTC	4 (0.000076%)
ATTCGACTCCAAAACACTAACCAACCTTCTTCTTGCTTCTCAAAGCTTTCATGGTGTAGCCAAAGTCCATATGAGTCTTTGGCTTTGTGTCTTCTAACAA	93 (0.001759%)
ATTCGACTCCAAAACACTAACCAACCTTCTTCTTGCTTCTCAAAGCTTTCATGGTGTAGCCAAAGTCCATATGAGTCTTTGGCTTTGTGTCTTTTAACAA	201 (0.003803%)
ATTCGTACTGAAAATCAGAATCAAACGAGCTTTTACCCTTTTGTTCCACACGAGATTTCTGTTCTCGTTGAGCTCATCTTAGGACACCTGCGTTATCTTT	12 (0.000227%)
ATTCGTATTTCATAGTCAGAGGTGAAATTCTTGGATTTATGAAAGACGAACAACTGCGAAAGCATTTGCCAAGGATGTTTTCATTAATCAAGAACGAAAG	8 (0.000151%)
ATTGAGTATAAGAACTTAAACCGCAACCGCATCTTATAAGCCTAAGTAGTGTTTCCTTGTTAGAAGACACAAAGCCAAAGACTCATATGGACTTTGGCTA	29 (0.000549%)
ATTGGAGGGCAAGTCTGGTGCCAGCAGCCGCGGTAATTCCAGCTCCAATAGCGTATATTTAAGTTGTTGCAGTTAAAAAGCTCGTAGTTGAACCTTGGGA	1509 (0.028548%)
ATTGTATCCTTGTTAGAAGACACAAAGCCAAAGACTCATATGGACTTTGGCTACACCATGAAAGCTTTGAGAAGCAAGAAGAAGGTTGGTTAGTGTTTTG	26 (0.000492%)
ATTGTCAGGTGGGGAGTTTGGCTGGGGCGGCACATCTGTTAAAAGATAACGCAGGTGTCCTAAGATGAGCTCAACGAGAACAGAAATCTCGTGTGGAACA	24 (0.000454%)
ATTGTTCCATCGACCAGAGGCTGTTCACCTTGGAGACCTGATGCGGTTATGAGTACGACCGGGCGTGAGCGGCACTCGGTCCTCCGGATTTTCAAGGGCC	11 (0.000208%)
ATTGTTGGTCTTCAACGAGGAATTCCTAGTAAGCGCGAGTCATCAGCTCGCGTTGACTACGTCCCTGCCCTTTGTACACACCGCCCGTCGCTCCTACCGA	41 (0.000776%)
ATTTCATAGTCAGAGGTGAAATTCTTGGATTTATGAAAGACGAACAACTGCGAAAGCATTTGCCAAGGATGTTTTCATTAATCAAGAACGAAAGTTGGGG	6 (0.000114%)

ATTTCGTTCATCACCCTTGGCCGGCTATCGAACAGCCGGACTCCCATCAAAAGATGGTTGCCAAGAACATC TTCGTTACGGTTTGCTAATTCTCGGAATA	19 (0.000359%)	
ATTTCGTTCATCACCCTTGGCCGGCTTTCGAACAGCCGGACTCCCATCAAAAGATGGTTGCCAAGAACATC TTCGTTACGGTTTGCTAATTCTCGGAATA	12 (0.000227%)	
ATTTCTGCCCTATCAACTTTCGATGGTAGGATAGTGGCCTACCATGGTGGTAACGGGTGACGGAGAATTAG GGTTCGATTCCGGAGAGGGAGCCTGAGAA	7 (0.000132%)	
ATTTGCACGTCAGTATCGCTGCGGGCTTCCACCAGAGTTTCCTCTGGCTTTACCCCGCTCAGGCATAGTTC ACCATCTTTCGGGTCCCGACAGGCATGCT	33 (0.000624%)	
ATTTTATCGGTCGCTCTTGTCCGGAAGCTGTAGATGACCCAAAGTCCATATAGCGACCCCAGGTCAGGCGG GATTACCCGCTGAGTTTAAGCATATCAAT	16 (0.000303%)	
ATTTTCAAGGGCCGCCGGGGGCGCACCGGACACCACGCGACGTGCGGTGCTCTTCCAGCCGCTGGACCCTA CCTCCGGCTGAGCCGTTTCCAGGGTGGGC	7 (0.000132%)	
CAAAATCATCAATCGTTCCAACTAATCTACCGAAGTACTCGGCTAAGAAGAAAGAAGACGGACGAATCCGA GCCAAAGCCGTACAAGCGCGAGATACCTT	29 (0.000549%)	
CAAACGTTCAATATGACAAACCCATGCCAAGTAAAGAGAAAATGAAAACTGGTGATTGTTGCGGAAATCGT CCAGGATTCCTCGACCAGGACTTGAAATC	28 (0.000530%)	
CAAAGCCAAAGACTCATATGGACTTTGGCTACACCATGAAAGCTTTGAGAAGCAAGAAGAAGGTTGGTTAG TGTTTTGGAGTCGAATATGACTTGATGTC	113 (0.002138%)	
CAAAGGGTGTTGGTCGATTAAGACAGCAGGACGGTGGTCATGGAAGTCGAAATCCGCTAAGGAGTGTGTAA CAACTCACCTGCCGAATCAACTAGCCCCG	31 (0.000586%)	
CAAATCGTCGTCCCTCACCATCCTTTGCTGATGCGGGACGGAAGCTGGTCTCCCGTGTGTTACCGCACGCG GTTGGCCTAAATCCGAGCCAAGGACGCCT	5 (0.000095%)	
CAAATTAGGTACGAAACACAGGCCCCGGAACTCATCATCGAGCGTAACATCGCCCGTGAATTAACTGAGAA GGATAGGTGGTAGGTAGTTCGATGCGCGA	8 (0.000151%)	
CAAATTTCTGCCCTATCAACTTTCGATGGTAGGATAGTGGCCTACCATGGTGGTAACGGGTGACGGAGAAT TAGGGTTCGATTCCGGAGAGGGAGCCTGA	113 (0.002138%)	
CAACCTAGGCGAGACAAGGGTTCACATTTCGTTCATCACCCTTGGCCGGCTATCGAACAGCCGGACTCCCA TCAAAAGATGGTTGCCAAGAACATCTTCG	4 (0.000076%)	
CAACGAAGCACGCCCATCCAACCTAGGCGAGACAAGGGTTCACATTTCGTTCATCACCCTTGGCCGGCTAT CGAACAGCCGGACTCCCATCAAAAGATGG	71 (0.001343%)	
CAACGGGCAGAGCCCGCGTCGACCTTTTATCTAATAAATGCGTCCCTTCCATAAGTCGGGGTTTGTTGCAC GTATTAGCTCTAGAATTACTACGGTTATC	22 (0.000416%)	
CAACGTTAGGGAGTCCGGAGACGTCGGCGGGGGCCTCGGGAAGAGTTATCTTTTCTGTTTAACAGCCTGCC CACCCTGGAAACGGCTCAGCCGGAGGTAG	4 (0.000076%)	
CAACTTTCGATGGTAGGATAGTGGCCTACCATGGTGGTAACGGGTGACGGAGAATTAGGGTTCGATTCCGG AGAGGGAGCCTGAGAAACGGCTACCACAT	22 (0.000416%)	
CAAGGGCCGCCGGGGGCGCACCGGACACCACGCGACGTGCGGTGCTCTTCCAGCCGCTGGACCCTACCTCC GGCTGAGCCGTTTCCAGGGTGGGCAGGCT	8 (0.000151%)	
CAAGGGTTCACATTTCGTTCATCACCCTTGGCCGGCTATCGAACAGCCGGACTCCCATCAAAAGATGGTTG CCAAGAACATCTTCGTTACGGTTTGCTAA	26 (0.000492%)	
CAAGGGTTCACATTTCGTTCATCACCCTTGGCCGGCTTTCGAACAGCCGGACTCCCATCAAAAGATGGTTG CCAAGAACATCTTCGTTACGGTTTGCTAA	22 (0.000416%)	
CAAGTCATATTCGACTCCAAAACACTAACCAACCTTCTTCTTGCTTCTCAAAGCTTTCATGGTGTAGCCAA AGTCCATATGAGTCTTTGGCTTTGTGTCT	150 (0.002838%)	
CAAGTCTGGTGCCAGCAGCCGCGGTAATTCCAGCTCCAATAGCGTATATTTAAGTTGTTGCAGTTAAAAAG CTCGTAGTTGAACCTTGGGATGGGTCGGC	21 (0.000397%)	
CAATAGCGTATATTTAAGTTGTTGCAGTTAAAAAGCTCGTAGTTGAACCTTGGGATGGGTCGGCCGGTCCG CCTTTGGTGTGCATTGGTCGGCTTGTCCC	9 (0.000170%)	
CAATCAGCTTCCTTGCGCCTTACGGGTTTACTCACCCGTTGACTCGCACACATGTCAGACTCCTTGGTCCG TGTTTCAAGACGGGTCGAATGGGGAGCCC	156 (0.002951%)	
CAATCATACACATGACATCAAGTCATATTCGACTCCAAAACACTAACCAACCTTCTTCTTGCTTCTCAAAG CTTTCATGGTGTAGCCAAAGTCCATATGA	142 (0.002686%)	
CAATCGTTCCAACTAATCTACCGAAGTACTCGGCTAAGAAGAAAGAAGACGGACGAATCCGAGCCAAAGCC GTACAAGCGCGAGATACCTTCGGGACAGC	3 (0.000057%)	
CACAAACGTTCAATATGACAAACCCATGCCAAGTAAAGAGAAAATGAAAACTGGTGATTGTTGCGGAAATC GTCCAGGATTCCTCGACCAGGACTTGAAA	16 (0.000303%)	
CACAAAGCCAAAGACTCATATGGACTTTGGCTACACCATGAAAGCTTTGAGAAGCAAGAAGAAGGTTGGTT AGTGTTTTGGAGTCGAATATGACTTGATG	46 (0.000870%)	
CACAAAGGGTGTTGGTCGATTAAGACAGCAGGACGGTGGTCATGGAAGTCGAAATCCGCTAAGGAGTGTGT AACAACTCACCTGCCGAATCAACTAGCCC	49 (0.000927%)	
CACAAATCGTCGTCCCTCACCATCCTTTGCTGATGCGGGACGGAAGCTGGTCTCCCGTGTGTTACCGCACG CGGTTGGCCTAAATCCGAGCCAAGGACGC	52 (0.000984%)	
CACACATGTCAGACTCCTTGGTCCGTGTTTCAAGACGGGTCGAATGGGGAGCCCACAGGCCGACGCCCGGA GCACGCTGATGCCGAGGCACGCCGTTAGG	7 (0.000132%)	
CACAGGCCCCGGAACTCATCATCGAGCGTAACATCGCCCGTGAATTAACTGAGAAGGATAGGTGGTAGGTA GTTCGATGCGCGAGCATGGAGCCTACGAA	26 (0.000492%)	
CACATCTGTTAAAAGATAACGCAGGTGTCCTAAGATGAGCTCAACGAGAACAGAAATCTCGTGTGGAACAA AAGGGTAAAAGCTCGTTTGATTCTGATTT	10 (0.000189%)	
CACATGACATCAAGTCATATTCGACTCCAAAACACTAACCAACCTTCTTCTTGCTTCTCAAAGCTTTCATG GTGTAGCCAAAGTCCATATGAGTCTTTGG	41 (0.000776%)	
CACATTTCGTTCATCACCCTTGGCCGGCTATCGAACAGCCGGACTCCCATCAAAAGATGGTTGCCAAGAAC ATCTTCGTTACGGTTTGCTAATTCTCGGA	9 (0.000170%)	
CACATTTCGTTCATCACCCTTGGCCGGCTTTCGAACAGCCGGACTCCCATCAAAAGATGGTTGCCAAGAAC ATCTTCGTTACGGTTTGCTAATTCTCGGA	7 (0.000132%)	
CACCATCCTTTGCTGATGCGGGACGGAAGCTGGTCTCCCGTGTGTTACCGCACGCGGTTGGCCTAAATCCG AGCCAAGGACGCCTGGAGCGTACCGACAT	5 (0.000095%)	
CACCCGTTGACTCGCACACATGTCAGACTCCTTGGTCCGTGTTTCAAGACGGGTCGAATGGGGAGCCCACA GGCCGACGCCCGGAGCACGCTGATGCCGA	12 (0.000227%)	
CACCGGACACCACGCGACGTGCGGTGCTCTTCCAGCCGCTGGACCCTACCTCCGGCTGAGCCGTTTCCAGG GTGGGCAGGCTGTTAAACAGAAAAGATAA	3 (0.000057%)	
CACGCCCATCCAACCTAGGCGAGACAAGGGTTCACATTTCGTTCATCACCCTTGGCCGGCTATCGAACAGC CGGACTCCCATCAAAAGATGGTTGCCAAG	21 (0.000397%)	
CACGCGCCTAACGGCGTGCCTCGGCATCAGCGTGCTCCGGGCGTCGGCCTGTGGGCTCCCCATTCGACCCG TCTTGAAACACGGACCAAGGAGTCTGACA	90 (0.001703%)	
CACGCTTTCACGGTTCGTATTCGTACTGAAAATCAGAATCAAACGAGCTTTTACCCTTTTGTTCCACACGA GATTTCTGTTCTCGTTGAGCTCATCTTAG	2001 (0.037855%)	

CACGGTTCGTATTCGTACTGAAAATCAGAATCAAACGAGCTTTTACCCTTTTGTTCCACACGAGATTTCTG TTCTCGTTGAGCTCATCTTAGGACACCTG	6 (0.000114%)
CACTTGGTGATATGAACACAAACGTTCAATATGACAAACCCATGCCAAGTAAAGAGAAAATGAAAACTGGT GATTGTTGCGGAAATCGTCCAGGATTCCT	3 (0.000057%)
CAGAGCAACGGGCAGAGCCCGCGTCGACCTTTTATCTAATAAATGCGTCCCTTCCATAAGTCGGGGTTTGT TGCACGTATTAGCTCTAGAATTACTACGG	9 (0.000170%)
CAGAGCCCGCGTCGACCTTTTATCTAATAAATGCGTCCCTTCCATAAGTCGGGGTTTGTTGCACGTATTAG CTCTAGAATTACTACGGTTATCCGAGTAG	11 (0.000208%)
CAGAGCGTAGGCTTGCTTTGAGCACTCTAATTTCTTCAAAGTAACAGCGCCGGAGGCACGACCCGGCCAAT TAAGACCAGGAGCGTATCGCCGACCGAAG	34 (0.000643%)
CAGCACGCGCCTAACGGCGTGCCTCGGCATCAGCGTGCTCCGGGCGTCGGCCTGTGGGCTCCCCATTCGAC CCGTCTTGAAACACGGACCAAGGAGTCTG	74 (0.001400%)
CAGCAGCCGCGGTAATTCCAGCTCCAATAGCGTATATTTAAGTTGTTGCAGTTAAAAAGCTCGTAGTTGAA CCTTGGGATGGGTCGGCCGGTCCGCCTTT	26 (0.000492%)
CAGCAGGACGGTGGTCATGGAAGTCGAAATCCGCTAAGGAGTGTGTAACAACTCACCTGCCGAATCAACTA GCCCCGAAAATGGATGGCGCTTAAGCGCG	5 (0.000095%)
CAGCAGTTCTCGGACAAAAATTGCTGAGTGGCCGAGAAGAATGGGCGTGTCATGCGTGGGCTGACATGGAT TCTTCGAGGCCTAGGGGTGGCGGTATATA	4 (0.000076%)
CAGCCGCGGTAATTCCAGCTCCAATAGCGTATATTTAAGTTGTTGCAGTTAAAAAGCTCGTAGTTGAACCT TGGGATGGGTCGGCCGGTCCGCCTTTGGT	9 (0.000170%)
CAGCCTGCTAACTAGCTACGTGGAGGCATCCCTTCACGGCCGGCTTCTTAGAGGGACTATGGCCGTTTAGG CCAAGGAAGTTTGAGGCAATAACAGGTCT	26 (0.000492%)
CAGCGTGCTCCGGGCGTCGGCCTGTGGGCTCCCCATTCGACCCGTCTTGAAACACGGACCAAGGAGTCTGA CATGTGTGCGAGTCAACGGGTGAGTAAAC	6 (0.000114%)
CAGCTCCAATAGCGTATATTTAAGTTGTTGCAGTTAAAAAGCTCGTAGTTGAACCTTGGGATGGGTCGGCC GGTCCGCCTTTGGTGTGCATTGGTCGGCT	5 (0.000095%)
CAGCTTCCTTGCGCCTTACGGGTTTACTCACCCGTTGACTCGCACACATGTCAGACTCCTTGGTCCGTGTT TCAAGACGGGTCGAATGGGGAGCCCACAG	108 (0.002043%)
CAGGACGGTGGTCATGGAAGTCGAAATCCGCTAAGGAGTGTGTAACAACTCACCTGCCGAATCAACTAGCC CCGAAAATGGATGGCGCTTAAGCGCGCGA	21 (0.000397%)
CAGGGACAGTCGGGGGCATTCGTATTTCATAGTCAGAGGTGAAATTCTTGGATTTATGAAAGACGAACAAC TGCGAAAGCATTTGCCAAGGATGTTTTCA	34 (0.000643%)
CAGGGGTTGAAATCGTCGACCAGGTCCGAGACTTCATCGACCGGGTCCGAGGATTCGTCGACCAGGACGGC CGGATGTCCGAGAAAAAAAAATGTTGCCG	18 (0.000341%)
CAGGTGGGGAGTTTGGCTGGGGCGGCACATCTGTTAAAAGATAACGCAGGTGTCCTAAGATGAGCTCAACG AGAACAGAAATCTCGTGTGGAACAAAAGG	27 (0.000511%)
CAGTCGGGGGCATTCGTATTTCATAGTCAGAGGTGAAATTCTTGGATTTATGAAAGACGAACAACTGCGAA AGCATTTGCCAAGGATGTTTTCATTAATC	9 (0.000170%)
CAGTTCTCGGACAAAAATTGCTGAGTGGCCGAGAAGAATGGGCGTGTCATGCGTGGGCTGACATGGATTCT TCGAGGCCTAGGGGTGGCGGTATATAACT	5 (0.000095%)
CATACACATGACATCAAGTCATATTCGACTCCAAAACACTAACCAACCTTCTTCTTGCTTCTCAAAGCTTT CATGGTGTAGCCAAAGTCCATATGAGTCT	26 (0.000492%)
CATATGAGTCTTTGGCTTTGTGTCTTCTAACAAGGATACA	6766 (0.051200%)
CATATGCTTGTCTCAAAGATTAAGCCATGCATGTGTAAGTATGAACGAATTCAGACTGTGAAACTGCGAAT GGCTCATTAAATCAGTTATAGTTTGTTTG	47 (0.000889%)
CATATTCGACTCCAAAACACTAACCAACCTTCTTCTTGCTTCTCAAAGCTTTCATGGTGTAGCCAAAGTCC ATATGAGTCTTTGGCTTTGTGTCTTCTAA	75 (0.001419%)
CATATTCGACTCCAAAACACTAACCAACCTTCTTCTTGCTTCTCAAAGCTTTCATGGTGTAGCCAAAGTCC ATATGAGTCTTTGGCTTTGTGTCTTTTAA	320 (0.006054%)
CATCAAGTCATATTCGACTCCAAAACACTAACCAACCTTCTTCTTGCTTCTCAAAGCTTTCATGGTGTAGC CAAAGTCCATATGAGTCTTTGGCTTTGTG	45 (0.000851%)
CATCAATCGTTCCAACTAATCTACCGAAGTACTCGGCTAAGAAGAAAGAAGACGGACGAATCCGAGCCAAA GCCGTACAAGCGCGAGATACCTTCGGGAC	10 (0.000189%)
CATCAGAGCAACGGGCAGAGCCCGCGTCGACCTTTTATCTAATAAATGCGTCCCTTCCATAAGTCGGGGTT TGTTGCACGTATTAGCTCTAGAATTACTA	47 (0.000889%)
CATCAGCGTGCTCCGGGCGTCGGCCTGTGGGCTCCCCATTCGACCCGTCTTGAAACACGGACCAAGGAGTC TGACATGTGTGCGAGTCAACGGGTGAGTA	12 (0.000227%)
CATCATCGAGCGTAACATCGCCCGTGAATTAACTGAGAAGGATAGGTGGTAGGTAGTTCGATGCGCGAGCA TGGAGCCTACGAACACTAGCTATCCGATC	16 (0.000303%)
CATCATTCAAATTTCTGCCCTATCAACTTTCGATGGTAGGATAGTGGCCTACCATGGTGGTAACGGGTGAC GGAGAATTAGGGTTCGATTCCGGAGAGGG	25 (0.000473%)
CATCCAACCTAGGCGAGACAAGGGTTCACATTTCGTTCATCACCCTTGGCCGGCTTTCGAACAGCCGGACT CCCATCAAAAGATGGTTGCCAAGAACATC	4 (0.000076%)
CATCCTTTGCTGATGCGGGACGGAAGCTGGTCTCCCGTGTGTTACCGCACGCGGTTGGCCTAAATCCGAGC CAAGGACGCCTGGAGCGTACCGACATGCG	113 (0.002138%)
CATCGAACTACCTACCACCTATCCTTCTCAGTTAATTCACGGGCGATGTTACGCTCGATGATGAGTTCCGG GGCCTGTGTTTCGTACCTAATTTGAAGGA	10 (0.000189%)
CATCGACACCTTGCGGCTAGGAACTGGAACGAGACGGGTGGCAAAGATTTCGAGTAGCACTTCATACTACC GTGGGTTTTTTAAACCTTCCGAGTTTTGT	6 (0.000114%)
CATGAATCATCAGAGCAACGGGCAGAGCCCGCGTCGACCTTTTATCTAATAAATGCGTCCCTTCCATAAGT CGGGGTTTGTTGCACGTATTAGCTCTAGA	169 (0.003197%)
CATGACATCAAGTCATATTCGACTCCAAAACACTAACCAACCTTCTTCTTGCTTCTCAAAGCTTTCATGGT GTAGCCAAAGTCCATATGAGTCTTTGGCT	39 (0.000738%)
CATGCTAATGTATCCAGAGCGTAGGCTTGCTTTGAGCACTCTAATTTCTTCAAAGTAACAGCGCCGGAGGC ACGACCCGGCCAATTAAGACCAGGAGCGT	4 (0.000076%)
CATGGAAGTCGAAATCCGCTAAGGAGTGTGTAACAACTCACCTGCCGAATCAACTAGCCCCGAAAATGGAT GGCGCTTAAGCGCGCGACCTATACCCGGC	49 (0.000927%)
CATGGCCTCTGTGCTGGCGACGCATCATTCAAATTTCTGCCCTATCAACTTTCGATGGTAGGATAGTGGCC TACCATGGTGGTAACGGGTGACGGAGAAT	257 (0.004862%)
CATGTGTATGATTGAGTATAAGAACTTAAACCGCAACCGCATCTTATAAGCCTAAGTAGTGTTTCCTTGTT AGAAGACACAAAGCCAAAGACTCATATGG	17 (0.000322%)
CATTCAAATTTCTGCCCTATCAACTTTCGATGGTAGGATAGTGGCCTACCATGGTGGTAACGGGTGACGGA GAATTAGGGTTCGATTCCGGAGAGGGAGC	8 (0.000151%)
CATTGTCAGGTGGGGAGTTTGGCTGGGGCGGCACATCTGTTAAAAGATAACGCAGGTGTCCTAAGATGAGC TCAACGAGAACAGAAATCTCGTGTGGAAC	11 (0.000208%)

CATTGTTCCATCGACCAGAGGCTGTTCACCTTGGAGACCTGATGCGGTTATGAGTACGACCGGGCGTGAGC<br>GGCACTCGGTCCTCCGGATTTTCAAGGGC	26 (0.000492%)	
CCAAAGACTCATATGGACTTTGGCTACACCATGAAAGCTTTGAGAAGCAAGAAGAAGGTTGGTTAGTGTTT<br>TGGAGTCGAATATGACTTGATGTCATGTG	131 (0.002478%)	
CCAACCTAGGCGAGACAAGGGTTCACATTTCGTTCATCACCCTTGGCCGGCTATCGAACAGCCGGACTCCC<br>ATCAAAAGATGGTTGCCAAGAACATCTTC	23 (0.000435%)	
CCAACGAAGCACGCCCATCCAACCTAGGCGAGACAAGGGTTCACATTTCGTTCATCACCCTTGGCCGGCTA<br>TCGAACAGCCGGACTCCCATCAAAAGATG	6 (0.000114%)	
CCAATCAGCTTCCTTGCGCCTTACGGGTTTACTCACCCGTTGACTCGCACACATGTCAGACTCCTTGGTCC<br>GTGTTTCAAGACGGGTCGAATGGGGAGCC	14 (0.000265%)	
CCACAAAGGGTGTTGGTCGATTAAGACAGCAGGACGGTGGTCATGGAAGTCGAAATCCGCTAAGGAGTGTG<br>TAACAACTCACCTGCCGAATCAACTAGCC	6 (0.000114%)	
CCAGAGCGTAGGCTTGCTTTGAGCACTCTAATTTCTTCAAAGTAACAGCGCCGGAGGCACGACCCGGCCAA<br>TTAAGACCAGGAGCGTATCGCCGACCGAA	17 (0.000322%)	
CCAGCAGCCGCGGTAATTCCAGCTCCAATAGCGTATATTTAAGTTGTTGCAGTTAAAAAGCTCGTAGTTGA<br>ACCTTGGGATGGGTCGGCCGGTCCGCCTT	9 (0.000170%)	
CCAGCTCCAATAGCGTATATTTAAGTTGTTGCAGTTAAAAAGCTCGTAGTTGAACCTTGGGATGGGTCGGC<br>CGGTCCGCCTTTGGTGTGCATTGGTCGGC	14 (0.000265%)	
CCAGGGGTTGAAATCGTCGACCAGGTCCGAGACTTCATCGACCGGGTCCGAGGATTCGTCGACCAGGACGG<br>CCGGATGTCCGAGAAAAAAAAATGTTGCC	3 (0.000057%)	
CCATCCAACCTAGGCGAGACAAGGGTTCACATTTCGTTCATCACCCTTGGCCGGCTTTCGAACAGCCGGAC<br>TCCCATCAAAAGATGGTTGCCAAGAACAT	3 (0.000057%)	
CCATCCTTTGCTGATGCGGGACGGAAGCTGGTCTCCCGTGTGTTACCGCACGCGGTTGGCCTAAATCCGAG<br>CCAAGGACGCCTGGAGCGTACCGACATGC	14 (0.000265%)	
CCATGCTAATGTATCCAGAGCGTAGGCTTGCTTTGAGCACTCTAATTTCTTCAAAGTAACAGCGCCGGAGG<br>CACGACCCGGCCAATTAAGACCAGGAGCG	14 (0.000265%)	
CCATGCTCGCGCATCGAACTACCTACCACCTATCCTTCTCAGTTAATTCACGGGCGATGTTACGCTCGATG<br>ATGAGTTCCGGGGCCTGTGTTTCGTACCT	4 (0.000076%)	
CCATGGGCATCGACACCTTGCGGCTAGGAACTGGAACGAGACGGGTGGCAAAGATTTCGAGTAGCACTTCA<br>TACTACCGTGGGTTTTTTAAACCTTCCGA	10 (0.000189%)	
CCCACAAAGGGTGTTGGTCGATTAAGACAGCAGGACGGTGGTCATGGAAGTCGAAATCCGCTAAGGAGTGT<br>GTAACAACTCACCTGCCGAATCAACTAGC	9 (0.000170%)	
CCCATCCAACCTAGGCGAGACAAGGGTTCACATTTCGTTCATCACCCTTGGCCGGCTATCGAACAGCCGGA<br>CTCCCATCAAAAGATGGTTGCCAAGAACA	17 (0.000322%)	
CCCATCCAACCTAGGCGAGACAAGGGTTCACATTTCGTTCATCACCCTTGGCCGGCTTTCGAACAGCCGGA<br>CTCCCATCAAAAGATGGTTGCCAAGAACA	15 (0.000284%)	
CCCATGCTAATGTATCCAGAGCGTAGGCTTGCTTTGAGCACTCTAATTTCTTCAAAGTAACAGCGCCGGAG<br>GCACGACCCGGCCAATTAAGACCAGGAGC	17 (0.000322%)	
CCCATGGGCATCGACACCTTGCGGCTAGGAACTGGAACGAGACGGGTGGCAAAGATTTCGAGTAGCACTTC<br>ATACTACCGTGGGTTTTTTAAACCTTCCG	7 (0.000132%)	
CCCGAAGGTATCTCGCGCTTGTACGGCTTTGGCTCGGATTCGTCCGTCTTCTTTCTTCTTAGCCGAGTACT<br>TCGGTAGATTAGTTGGAACGATTGATGAT	48 (0.000908%)	
CCCGAGTGTGAGCGAGGTGTGAGTGTCGCCCATGGGCATCGACACCTTGCGGCTAGGAACTGGAACGAGAC<br>GGGTAGCAAAGATTTCGAGTAGCACTTCA	14 (0.000265%)	
CCCGAGTGTGAGCGAGGTGTGAGTGTCGCCCATGGGCATCGACACCTTGCGGCTAGGAACTGGAACGAGAC<br>GGGTGGCAAAGATTTCGAGTAGCACTTCA	19 (0.000359%)	
CCCGCCAATCAGCTTCCTTGCGCCTTACGGGTTTACTCACCCGTTGACTCGCACACATGTCAGACTCCTTG<br>GTCCGTGTTTCAAGACGGGTCGAATGGGG	14 (0.000265%)	
CCCGCCGAAGCGAGCCTTGGGACCAAAAACAGGGGTTGTACCCCGCCTCCGATTCACGGAGTAAGTAAAAT<br>AACGTTAAAAGTAGTGGTATTTCACTTGC	6 (0.000114%)	
CCCGCGTCGACCTTTTATCTAATAAATGCGTCCCTTCCATAAGTCGGGGTTTGTTGCACGTATTAGCTCTA<br>GAATTACTACGGTTATCCGAGTAGTAGTT	36 (0.000681%)	
CCCTCACCATCCTTTGCTGATGCGGGACGGAAGCTGGTCTCCCGTGTGTTACCGCACGCGGTTGGCCTAAA<br>TCCGAGCCAAGGACGCCTGGAGCGTACCG	19 (0.000359%)	
CCCTTGCCTACATTGTTCCATCGACCAGAGGCTGTTCACCTTGGAGACCTGATGCGGTTATGAGTACGACC<br>GGGCGTGAGCGGCACTCGGTCCTCCGGAT	25 (0.000473%)	
CCGAAGCGAGCCTTGGGACCAAAAACAGGGGTTGTACCCCGCCTCCGATTCACGGAGTAAGTAAAATAACG<br>TTAAAAGTAGTGGTATTTCACTTGCGCCG	12 (0.000227%)	
CCGAAGGTATCTCGCGCTTGTACGGCTTTGGCTCGGATTCGTCCGTCTTCTTTCTTCTTAGCCGAGTACTT<br>CGGTAGATTAGTTGGAACGATTGATGATT	6 (0.000114%)	
CCGACTTCCCTTGCCTACATTGTTCCATCGACCAGAGGCTGTTCACCTTGGAGACCTGATGCGGTTATGAG<br>TACGACCGGGCGTGAGCGGCACTCGGTCC	49 (0.000927%)	
CCGAGTGTGAGCGAGGTGTGAGTGTCGCCCATGGGCATCGACACCTTGCGGCTAGGAACTGGAACGAGACG<br>GGTAGCAAAGATTTCGAGTAGCACTTCAT	12 (0.000227%)	
CCGAGTGTGAGCGAGGTGTGAGTGTCGCCCATGGGCATCGACACCTTGCGGCTAGGAACTGGAACGAGACG<br>GGTGGCAAAGATTTCGAGTAGCACTTCAT	18 (0.000341%)	
CCGCCAATCAGCTTCCTTGCGCCTTACGGGTTTACTCACCCGTTGACTCGCACACATGTCAGACTCCTTGG<br>TCCGTGTTTCAAGACGGGTCGAATGGGGA	57 (0.001078%)	
CCGCCGGGGGCGCACCGGACACCACGCGACGTGCGGTGCTCTTCCAGCCGCTGGACCCTACCTCCGGCTGA<br>GCCGTTTCCAGGGTGGGCAGGCTGTTAAA	5 (0.000095%)	
CCGCCTAGGCTGTCCCGAGTGTGAGCGAGGTGTGAGTGTCGCCCATGGGCATCGACACCTTGCGGCTAGGA<br>ACTGGAACGAGACGGGTGGCAAAGATTTC	69 (0.001305%)	
CCGCGGTAATTCCAGCTCCAATAGCGTATATTTAAGTTGTTGCAGTTAAAAAGCTCGTAGTTGAACCTTGG<br>GATGGGTCGGCCGGTCCGCCTTTGGTGTG	9 (0.000170%)	
CCGGAACCGGGACGTGGCGGTTGACGGCAACGTTAGGGAGTCCGGAGACGTCGGCGGGGGCCTCGGGAAGA<br>GTTATCTTTTCTGTTTAACAGCCTGCCCA	110 (0.002081%)	
CCGGACACCACGCGACGTGCGGTGCTCTTCCAGCCGCTGGACCCTACCTCCGGCTGAGCCGTTTCCAGGGT<br>GGGCAGGCTGTTAAACAGAAAAGATAACT	27 (0.000511%)	
CCGGAGACGTCGGCGGGGGCCTCGGGAAGAGTTATCTTTTCTGTTTAACAGCCTGCCCACCCTGGAAACGG<br>CTCAGCCGGAGGTAGGGTCCAGCGGCTGG	7 (0.000132%)	
CCGGATTTTCAAGGGCCGCCGGGGGCGCACCGGACACCACGCGACGTGCGGTGCTCTTCCAGCCGCTGGAC<br>CCTACCTCCGGCTGAGCCGTTTCCAGGGT	5 (0.000095%)	
CCGGGACGTGGCGGTTGACGGCAACGTTAGGGAGTCCGGAGACGTCGGCGGGGGCCTCGGGAAGAGTTATC<br>TTTTCTGTTTAACAGCCTGCCCACCCTGG	32 (0.000605%)	
CCGGGCGTCGGCCTGTGGGCTCCCCATTCGACCCGTCTTGAAACACGGACCAAGGAGTCTGACATGTGTGC<br>GAGTCAACGGGTGAGTAAACCCGTAAGGC	4 (0.000076%)	

CCGGGGGCGCACCGGACACCACGCGACGTGCGGTGCTCTTCCAGCCGCTGGACCCTACCTCCGGCTGAGCCGTTTCCAGGGTGGGCAGGCTGTTAAACAG	6 (0.000114%)
CCGTCGAGTTATCATGAATCATCAGAGCAACGGGCAGAGCCCGCGTCGACCTTTTATCTAATAAATGCGTCCCTTCCATAAGTCGGGGTTTGTTGCACGT	20 (0.000378%)
CCGTTGACTCGCACACATGTCAGACTCCTTGGTCCGTGTTTCAAGACGGGTCGAATGGGGAGCCCACAGGCCGACGCCCGGAGCACGCTGATGCCGAGGC	3 (0.000057%)
CCTAACGGCGTGCCTCGGCATCAGCGTGCTCCGGGCGTCGGCCTGTGGGCTCCCCATTCGACCCGTCTTGAAACACGGACCAAGGAGTCTGACATGTGTG	8 (0.000151%)
CCTAAGTAGTGTTTCCTTGTTAGAAGACACAAAGCCAAAGACTCATATGGACTTTGGCTACACCATGAAAGCTTTGAGAAGCAAGAAGAAGGTTGGTTAG	59 (0.001116%)
CCTACATTGTTCCATCGACCAGAGGCTGTTCACCTTGGAGACCTGATGCGGTTATGAGTACGACCGGGCGTGAGCGGCACTCGGTCCTCCGGATTTTCAA	9 (0.000170%)
CCTAGGCGAGACAAGGGTTCACATTTCGTTCATCACCCTTGGCCGGCTATCGAACAGCCGGACTCCCATCAAAAGATGGTTGCCAAGAACATCTTCGTTA	8 (0.000151%)
CCTAGGCTGTCCCGAGTGTGAGCGAGGTGTGAGTGTCGCCCATGGGCATCGACACCTTGCGGCTAGGAACTGGAACGAGACGGGTGGCAAAGATTTCGAG	48 (0.000908%)
CCTATGATGTTATCCCATGCTAATGTATCCAGAGCGTAGGCTTGCTTTGAGCACTCTAATTTCTTCAAAGTAACAGCGCCGGAGGCACGACCCGGCCAAT	43 (0.000813%)
CCTCACCATCCTTTGCTGATGCGGGACGGAAGCTGGTCTCCCGTGTGTTACCGCACGCGGTTGGCCTAAATCCGAGCCAAGGACGCCTGGAGCGTACCGA	78 (0.001476%)
CCTCAGCCTGCTAACTAGCTACGTGGAGGCATCCCTTCACGGCCGGCTTCTTAGAGGGACTATGGCCGTTTAGGCCAAGGAAGTTTGAGGCAATAACAGG	107 (0.002024%)
CCTCCGGATTTTCAAGGGCCGCCGGGGGCGCACCGGACACCACGCGACGTGCGGTGCTCTTCCAGCCGCTGGACCCTACCTCCGGCTGAGCCGTTTCCAG	3 (0.000057%)
CCTCGGCATCAGCGTGCTCCGGGCGTCGGCCTGTGGGCTCCCCATTCGACCCGTCTTGAAACACGGACCAAGGAGTCTGACATGTGTGCGAGTCAACGGG	4 (0.000076%)
CCTCTGTGCTGGCGACGCATCATTCAAATTTCTGCCCTATCAACTTTCGATGGTAGGATAGTGGCCTACCATGGTGGTAACGGGTGACGGAGAATTAGGG	106 (0.002005%)
CCTTGCCTACATTGTTCCATCGACCAGAGGCTGTTCACCTTGGAGACCTGATGCGGTTATGAGTACGACCGGGCGTGAGCGGCACTCGGTCCTCCGGATT	10 (0.000189%)
CCTTGCGCCTTACGGGTTTACTCACCCGTTGACTCGCACACATGTCAGACTCCTTGGTCCGTGTTTCAAGACGGGTCGAATGGGGAGCCCACAGGCCGAC	7 (0.000132%)
CCTTGTTAGAAGACACAAAGCCAAAGACTCATATGGACTTTGGCTACACCATGAAAGCTTTGAGAAGCAAGAAGAAGGTTGGTTAGTGTTTTGGAGTCGA	46 (0.000870%)
CCTTTGCTGATGCGGGACGGAAGCTGGTCTCCCGTGTGTTACCGCACGCGGTTGGCCTAAATCCGAGCCAAGGACGCCTGGAGCGTACCGACATGCGGTG	4 (0.000076%)
CGAAACACAGGCCCCGGAACTCATCATCGAGCGTAACATCGCCCGTGAATTAACTGAGAAGGATAGGTGGTAGGTAGTTCGATGCGCGAGCATGGAGCCT	37 (0.000700%)
CGAAATCCTATGATGTTATCCCATGCTAATGTATCCAGAGCGTAGGCTTGCTTTGAGCACTCTAATTTCTTCAAAGTAACAGCGCCGGAGGCACGACCCG	444 (0.008400%)
CGAACTACCTACCACCTATCCTTCTCAGTTAATTCACGGGCGATGTTACGCTCGATGATGAGTTCCGGGGCCTGTGTTTCGTACCTAATTTGAAGGAATT	5 (0.000095%)
CGAAGCACGCCCATCCAACCTAGGCGAGACAAGGGTTCACATTTCGTTCATCACCCTTGGCCGGCTATCGAACAGCCGGACTCCCATCAAAAGATGGTTG	27 (0.000511%)
CGAAGGTATCTCGCGCTTGTACGGCTTTGGCTCGGATTCGTCCGTCTTCTTTCTTCTTAGCCGAGTACTTCGGTAGATTAGTTGGAACGATTGATGATTT	19 (0.000359%)
CGACCAGGGGTTGAAATCGTCGACCAGGTCCGAGACTTCATCGACCGGGTCCGAGGATTCGTCGACCAGGACGGCCGGATGTCCGAGAAAAAAAAATGTT	27 (0.000511%)
CGACGCATCATTCAAATTTCTGCCCTATCAACTTTCGATGGTAGGATAGTGGCCTACCATGGTGGTAACGGGTGACGGAGAATTAGGGTTCGATTCCGGA	23 (0.000435%)
CGACGGGCGGTGTGTACAAAGGGCAGGGACGTAGTCAACGCGAGCTGATGACTCGCGCTTACTAGGAATTCCTCGTTGAAGACCAACAATTGCAATGATC	16 (0.000303%)
CGACGTGGGTGGTTCGCCGCCCGCGACGTCGCGAGAAGTCCACTAAACCTTATCATTTAGAGGAAGGAGAAGTCGTAACAAGGTTTCCGTAGGTGAACCT	124 (0.002346%)
CGACTCCAAAACACTAACCAACCTTCTTCTTGCTTCTCAAAGCTTTCATGGTGTAGCCAAAGTCCATATGAGTCTTTGGCTTTGTGTCTTCTAACAAGGA	58 (0.001097%)
CGACTCCAAAACACTAACCAACCTTCTTCTTGCTTCTCAAAGCTTTCATGGTGTAGCCAAAGTCCATATGAGTCTTTGGCTTTGTGTCTTTTAACAAGGA	30 (0.000568%)
CGACTTCCCTTGCCTACATTGTTCCATCGACCAGAGGCTGTTCACCTTGGAGACCTGATGCGGTTATGAGTACGACCGGGCGTGAGCGGCACTCGGTCCT	24 (0.000454%)
CGAGACAAGGGTTCACATTTCGTTCATCACCCTTGGCCGGCTATCGAACAGCCGGACTCCCATCAAAAGATGGTTGCCAAGAACATCTTCGTTACGGTTT	6 (0.000114%)
CGAGACAAGGGTTCACATTTCGTTCATCACCCTTGGCCGGCTTTCGAACAGCCGGACTCCCATCAAAAGATGGTTGCCAAGAACATCTTCGTTACGGTTT	7 (0.000132%)
CGAGCTCGTGTAAAGTTGGGAATTCGTTAAGGAGCTGTTGCTTTGTTAGTGTAGAAACACTTGTGTAGAATTGGGGATTGTTTTTTTTGGAGTGATTTAG	741 (0.014018%)
CGAGGTGTGAGTGTCGCCCATGGGCATCGACACCTTGCGGCTAGGAACTGGAACGAGACGGGTGGCAAAGATTTCGAGTAGCACTTCATACTACCGTGGG	22 (0.000416%)
CGAGTGTGAGCATGCCTGTCGGGACCCGAAAGATGGTGAACTATGCCTGAGCGGGGTAAAGCCAGAGGAAACTCTGGTGGAAGCCCGCAGCGATACTGAC	4 (0.000076%)
CGAGTGTGAGCGAGGTGTGAGTGTCGCCCATGGGCATCGACACCTTGCGGCTAGGAACTGGAACGAGACGGGTAGCAAAGATTTCGAGTAGCACTTCATA	23 (0.000435%)
CGAGTGTGAGCGAGGTGTGAGTGTCGCCCATGGGCATCGACACCTTGCGGCTAGGAACTGGAACGAGACGGGTGGCAAAGATTTCGAGTAGCACTTCATA	34 (0.000643%)
CGAGTTATCATGAATCATCAGAGCAACGGGCAGAGCCCGCGTCGACCTTTTATCTAATAAATGCGTCCCTTCCATAAGTCGGGGTTTGTTGCACGTATTA	131 (0.002478%)
CGATCCATTACATTTTATCGGTCGCTCTTGTCCGGAAGCTGTAGATGACCCAAAGTCCATATAGCGACCCCAGGTCAGGCGGGATTACCCGCTGAGTTTA	6 (0.000114%)
CGATCCCGCCAATCAGCTTCCTTGCGCCTTACGGGTTTACTCACCCGTTGACTCGCACACATGTCAGACTCCTTGGTCCGTGTTTCAAGACGGGTCGAAT	4 (0.000076%)
CGATCCGTCGAGTTATCATGAATCATCAGAGCAACGGGCAGAGCCCGCGTCGACCTTTTATCTAATAAATGCGTCCCTTCCATAAGTCGGGGTTTGTTGC	163 (0.003084%)
CGATCGACCCGCCGAAGCGAGCCTTGGGACCAAAAACAGGGGTTGTACCCCGCCTCCGATTCACGGAGTAAGTAAAATAACGTTAAAAGTAGTGGTATTT	3 (0.000057%)
CGATTAAGACAGCAGGACGGTGGTCATGGAAGTCGAAATCCGCTAAGGAGTGTGTAACAACTCACCTGCCGAATCAACTAGCCCCGAAAATGGATGGCGC	14 (0.000265%)

CGATTAGTCTTTCGCCCCTATACCCAAGTCAGACGAACGATTTGCACGTCAGTATCGCTGCGGGCTTCCACCAGAGTTTCCTCTGGCTTTACCCCGCTCA	11 (0.000208%)	
CGATTTGCACGTCAGTATCGCTGCGGGCTTCCACCAGAGTTTCCTCTGGCTTTACCCCGCTCAGGCATAGTTCACCATCTTTCGGGTCCCGACAGGCATG	503 (0.009516%)	
CGCACACATGTCAGACTCCTTGGTCCGTGTTTCAAGACGGGTCGAATGGGGAGCCCACAGGCCGACGCCCGGAGCACGCTGATGCCGAGGCACGCCGTTA	20 (0.000378%)	
CGCACCGGACACCACGCGACGTGCGGTGCTCTTCCAGCCGCTGGACCCTACCTCCGGCTGAGCCGTTTCCAGGGTGGGCAGGCTGTTAAACAGAAAAGAT	4 (0.000076%)	
CGCATCATTCAAATTTCTGCCCTATCAACTTTCGATGGTAGGATAGTGGCCTACCATGGTGGTAACGGGTGACGGAGAATTAGGGTTCGATTCCGGAGAG	4 (0.000076%)	
CGCCAATCAGCTTCCTTGCGCCTTACGGGTTTACTCACCCGTTGACTCGCACACATGTCAGACTCCTTGGTCCGTGTTTCAAGACGGGTCGAATGGGGAG	1196 (0.022626%)	
CGCCCATCCAACCTAGGCGAGACAAGGGTTCACATTTCGTTCATCACCCTTGGCCGGCTATCGAACAGCCGGACTCCCATCAAAAGATGGTTGCCAAGAA	76 (0.001438%)	
CGCCCATCCAACCTAGGCGAGACAAGGGTTCACATTTCGTTCATCACCCTTGGCCGGCTTTCGAACAGCCGGACTCCCATCAAAAGATGGTTGCCAAGAA	89 (0.001684%)	
CGCCCATGGGCATCGACACCTTGCGGCTAGGAACTGGAACGAGACGGGTGGCAAAGATTTCGAGTAGCACTTCATACTACCGTGGGTTTTTTAAACCTTC	30 (0.000568%)	
CGCCGAAGCGAGCCTTGGGACCAAAAACAGGGGTTGTACCCCGCCTCCGATTCACGGAGTAAGTAAAATAACGTTAAAAGTAGTGGTATTTCACTTGCGC	4 (0.000076%)	
CGCCGCCCGCGACGTCGCGAGAAGTCCACTAAACCTTATCATTTAGAGGAAGGAGAAGTCGTAACAAGGTTTCCGTAGGTGAACCTGCGGAAGGATCGAT	16 (0.000303%)	
CGCCGGGGGCGCACCGGACACCACGCGACGTGCGGTGCTCTTCCAGCCGCTGGACCCTACCTCCGGCTGAGCCGTTTCCAGGGTGGGCAGGCTGTTAAAC	17 (0.000322%)	
CGCCTAACGGCGTGCCTCGGCATCAGCGTGCTCCGGGCGTCGGCCTGTGGGCTCCCCATTCGACCCGTCTTGAAACACGGACCAAGGAGTCTGACATGTG	30 (0.000568%)	
CGCCTAGGCTGTCCCGAGTGTGAGCGAGGTGTGAGTGTCGCCCATGGGCATCGACACCTTGCGGCTAGGAACTGGAACGAGACGGGTGGCAAAGATTTCG	183 (0.003462%)	
CGCCTTACGGGTTTACTCACCCGTTGACTCGCACACATGTCAGACTCCTTGGTCCGTGTTTCAAGACGGGTCGAATGGGGAGCCCACAGGCCGACGCCCG	9 (0.000170%)	
CGCGCATCGAACTACCTACCACCTATCCTTCTCAGTTAATTCACGGGCGATGTTACGCTCGATGATGAGTTCCGGGGCCTGTGTTTCGTACCTAATTTGA	12 (0.000227%)	
CGCGCCTAACGGCGTGCCTCGGCATCAGCGTGCTCCGGGCGTCGGCCTGTGGGCTCCCCATTCGACCCGTCTTGAAACACGGACCAAGGAGTCTGACATG	23 (0.000435%)	
CGCGCTTGTACGGCTTTGGCTCGGATTCGTCCGTCTTCTTTCTTCTTAGCCGAGTACTTCGGTAGATTAGTTGGAACGATTGATGATTTTGAGTTAATTG	26 (0.000492%)	
CGCGGTAATTCCAGCTCCAATAGCGTATATTTAAGTTGTTGCAGTTAAAAAGCTCGTAGTTGAACCTTGGGATGGGTCGGCCGGTCCGCCTTTGGTGTGC	11 (0.000208%)	
CGCTTGTACGGCTTTGGCTCGGATTCGTCCGTCTTCTTTCTTCTTAGCCGAGTACTTCGGTAGATTAGTTGGAACGATTGATGATTTTGAGTTAATTGAA	5 (0.000095%)	
CGCTTTCACGGTTCGTATTCGTACTGAAAATCAGAATCAAACGAGCTTTTACCCTTTTGTTCCACACGAGATTTCTGTTCTCGTTGAGCTCATCTTAGGA	9 (0.000170%)	
CGGAACCGGGACGTGGCGGTTGACGGCAACGTTAGGGAGTCCGGAGACGTCGGCGGGGGCCTCGGGAAGAGTTATCTTTTCTGTTTAACAGCCTGCCCAC	90 (0.001703%)	
CGGAAGAGCGTCGTGTAGGGAAAGAGTGTTCAGAGCCGTG	9 (0.000068%)	
CGGACACCACGCGACGTGCGGTGCTCTTCCAGCCGCTGGACCCTACCTCCGGCTGAGCCGTTTCCAGGGTGGGCAGGCTGTTAAACAGAAAAGATAACTC	36 (0.000681%)	
CGGAGACGTCGGCGGGGGCCTCGGGAAGAGTTATCTTTTCTGTTTAACAGCCTGCCCACCCTGGAAACGGCTCAGCCGGAGGTAGGGTCCAGCGGCTGGA	41 (0.000776%)	
CGGAGGACATTGTCAGGTGGGGAGTTTGGCTGGGGCGGCACATCTGTTAAAAGATAACGCAGGTGTCCTAAGATGAGCTCAACGAGAACAGAAATCTCGT	53 (0.001003%)	
CGGATAGCTAGTGTTCGTAGGCTCCATGCTCGCGCATCGAACTACCTACCACCTATCCTTCTCAGTTAATTCACGGGCGATGTTACGCTCGATGATGAGT	695 (0.013148%)	
CGGATTTTCAAGGGCCGCCGGGGGCGCACCGGACACCACGCGACGTGCGGTGCTCTTCCAGCCGCTGGACCCTACCTCCGGCTGAGCCGTTTCCAGGGTG	38 (0.000719%)	
CGGCAACGTTAGGGAGTCCGGAGACGTCGGCGGGGGCCTCGGGAAGAGTTATCTTTTCTGTTTAACAGCCTGCCCACCCTGGAAACGGCTCAGCCGGAGG	30 (0.000568%)	
CGGCAATTCCCCGCCACATCCTCTCAAACGCAATGGAAAGAGAGAAAGGACGAGGTCTTGACCGTCATCTTTTGCCCGAAGGACGGATGAGCTTTGGCGG	1085 (0.020526%)	
CGGCACATCTGTTAAAAGATAACGCAGGTGTCCTAAGATGAGCTCAACGAGAACAGAAATCTCGTGTGGAACAAAAGGGTAAAAGCTCGTTTGATTCTGA	11 (0.000208%)	
CGGCACTCGGTCCTCCGGATTTTCAAGGGCCGCCGGGGGCGCACCGGACACCACGCGACGTGCGGTGCTCTTCCAGCCGCTGGACCCTACCTCCGGCTGA	60 (0.001135%)	
CGGCATCAGCGTGCTCCGGGCGTCGGCCTGTGGGCTCCCCATTCGACCCGTCTTGAAACACGGACCAAGGAGTCTGACATGTGTGCGAGTCAACGGGTGA	14 (0.000265%)	
CGGCCTGTGGGCTCCCCATTCGACCCGTCTTGAAACACGGACCAAGGAGTCTGACATGTGTGCGAGTCAACGGGTGAGTAAACCCGTAAGGCGCAAGGAA	9 (0.000170%)	
CGGCGACGTGGGTGGTTCGCCGCCCGCGACGTCGCGAGAAGTCCACTAAACCTTATCATTTAGAGGAAGGAGAAGTCGTAACAAGGTTTCCGTAGGTGAA	464 (0.008778%)	
CGGCGTGCCTCGGCATCAGCGTGCTCCGGGCGTCGGCCTGTGGGCTCCCCATTCGACCCGTCTTGAAACACGGACCAAGGAGTCTGACATGTGTGCGAGT	14 (0.000265%)	
CGGGACGTGGCGGTTGACGGCAACGTTAGGGAGTCCGGAGACGTCGGCGGGGGCCTCGGGAAGAGTTATCTTTTCTGTTTAACAGCCTGCCCACCCTGGA	41 (0.000776%)	
CGGGCAGAGCCCGCGTCGACCTTTTATCTAATAAATGCGTCCCTTCCATAAGTCGGGGTTTGTTGCACGTATTAGCTCTAGAATTACTACGGTTATCCGA	51 (0.000965%)	
CGGGCGAGAGACCGATAGCGAACAAGTACCGCGAGGTAAAGATGAAAAGGACTTTGAAAAGAGAGTCAAAGAGTGCTTGAAATTGTCGGGAGGGAAGCGG	7 (0.000132%)	
CGGGCGGTGTGTACAAAGGGCAGGGACGTAGTCAACGCGAGCTGATGACTCGCGCTTACTAGGAATTCCTCGTTGAAGACCAACAATTGCAATGATCGAT	33 (0.000624%)	
CGGGCGTCGGCCTGTGGGCTCCCCATTCGACCCGTCTTGAAACACGGACCAAGGAGTCTGACATGTGTGCGAGTCAACGGGTGAGTAAACCCGTAAGGCG	32 (0.000605%)	
CGGGGGCATTCGTATTTCATAGTCAGAGGTGAAATTCTTGGATTTATGAAAGACGAACAACTGCGAAAGCATTTGCCAAGGATGTTTTCATTAATCAAGA	37 (0.000700%)	
CGGGGGCGCACCGGACACCACGCGACGTGCGGTGCTCTTCCAGCCGCTGGACCCTACCTCCGGCTGAGCCGTTTCCAGGGTGGGCAGGCTGTTAAACAGA	39 (0.000738%)	

CGGGTTTACTCACCCGTTGACTCGCACACATGTCAGACTCCTTGGTCCGTGTTTCAAGACGGGTCGAATGGGGAGCCCACAGGCCGACGCCCGGAGCACG	22 (0.000416%)	
CGGTAATTCCAGCTCCAATAGCGTATATTTAAGTTGTTGCAGTTAAAAAGCTCGTAGTTGAACCTTGGGATGGGTCGGCCGGTCCGCCTTTGGTGTGCAT	16 (0.000303%)	
CGGTAGGAGCGACGGGCGGTGTGTACAAAGGGCAGGGACGTAGTCAACGCGAGCTGATGACTCGCGCTTACTAGGAATTCCTCGTTGAAGACCAACAATT	65 (0.001230%)	
CGGTCCTCCGGATTTTCAAGGGCCGCCGGGGGCGCACCGGACACCACGCGACGTGCGGTGCTCTTCCAGCCGCTGGACCCTACCTCCGGCTGAGCCGTTT	12 (0.000227%)	
CGGTGGTCATGGAAGTCGAAATCCGCTAAGGAGTGTGTAACAACTCACCTGCCGAATCAACTAGCCCCGAAAATGGATGGCGCTTAAGCGCGCGACCTAT	21 (0.000397%)	
CGGTTGACGGCAACGTTAGGGAGTCCGGAGACGTCGGCGGGGGCCTCGGGAAGAGTTATCTTTTCTGTTTAACAGCCTGCCCACCCTGGAAACGGCTCAG	6 (0.000114%)	
CGGTTTAAGTTCTTATACTCAATCATACACATGACATCAAGTCATATTCGACTCCAAAACACTAACCAACCTTCTTCTTGCTTCTCAAAGCTTTCATGGT	70 (0.001324%)	
CGGTTTAAGTTGTTATACTCAATCATACACATGACAACAAGTCATATTCGACTCCAAAACACTAACCAACCTTCTTCTTGCTTCTCAAAGCTTTCATGGT	105 (0.001986%)	
CGTAAGAATTGTATCCTTGTTAAAAGACACAAAGCCAAAGACTCATATGGACTTTGGCTACACCATGAAAGCTTTGAGAAGCAAGAAGAAGGTTGGTTAG	539 (0.010197%)	
CGTAAGAATTGTATCCTTGTTAGAAGACACAAAGCCAAAG	236 (0.001786%)	
CGTATATTTAAGTTGTTGCAGTTAAAAAGCTCGTAGTTGAACCTTGGGATGGGTCGGCCGGTCCGCCTTTGGTGTGCATTGGTCGGCTTGTCCCTTCGGT	8 (0.000151%)	
CGTATTCGTACTGAAAATCAGAATCAAACGAGCTTTTACCCTTTTGTTCCACACGAGATTTCTGTTCTCGTTGAGCTCATCTTAGGACACCTGCGTTATC	23 (0.000435%)	
CGTATTTCATAGTCAGAGGTGAAATTCTTGGATTTATGAAAGACGAACAACTGCGAAAGCATTTGCCAAGGATGTTTTCATTAATCAAGAACGAAAGTTG	10 (0.000189%)	
CGTCCCTCACCATCCTTTGCTGATGCGGGACGGAAGCTGGTCTCCCGTGTGTTACCGCACGCGGTTGGCCTAAATCCGAGCCAAGGACGCCTGGAGCGTA	19 (0.000359%)	
CGTCGAGTTATCATGAATCATCAGAGCAACGGGCAGAGCCCGCGTCGACCTTTTATCTAATAAATGCGTCCCTTCCATAAGTCGGGGTTTGTTGCACGTA	103 (0.001949%)	
CGTCGTCCCTCACCATCCTTTGCTGATGCGGGACGGAAGCTGGTCTCCCGTGTGTTACCGCACGCGGTTGGCCTAAATCCGAGCCAAGGACGCCTGGAGC	4 (0.000076%)	
CGTCGTGTAGGGAAAGAGTGTTCAGAGCCGTGTAGATCTC	7 (0.000053%)	
CGTGCCTCGGCATCAGCGTGCTCCGGGCGTCGGCCTGTGGGCTCCCCATTCGACCCGTCTTGAAACACGGACCAAGGAGTCTGACATGTGTGCGAGTCAA	21 (0.000397%)	
CGTGCTCCGGGCGTCGGCCTGTGGGCTCCCCATTCGACCCGTCTTGAAACACGGACCAAGGAGTCTGACATGTGTGCGAGTCAACGGGTGAGTAAACCCG	4 (0.000076%)	
CGTGGCGGTTGACGGCAACGTTAGGGAGTCCGGAGACGTCGGCGGGGGCCTCGGGAAGAGTTATCTTTTCTGTTTAACAGCCTGCCCACCCTGGAAACGG	7 (0.000132%)	
CGTGGGTGGTTCGCCGCCCGCGACGTCGCGAGAAGTCCACTAAACCTTATCATTTAGAGGAAGGAGAAGTCGTAACAAGGTTTCCGTAGGTGAACCTGCG	33 (0.000624%)	
CGTGTAGGGAAAGAGTGTTCAGAGCCGTGTAGATCTCGGT	11 (0.000083%)	
CGTTAAGGAGCTGTTGCTTTGTTAGTGTAGAAACACTTGTGTAGAATTGGGGATTGTTTTTTTTGGAGTGATTTAGGGGAGGGTCGAATCTTAGCGACAA	197 (0.003727%)	
CGTTAGGGAGTCCGGAGACGTCGGCGGGGGCCTCGGGAAGAGTTATCTTTTCTGTTTAACAGCCTGCCCACCCTGGAAACGGCTCAGCCGGAGGTAGGGT	7 (0.000132%)	
CGTTCATCACCCTTGGCCGGCTTTCGAACAGCCGGACTCCCATCAAAAGATGGTTGCCAAGAACATCTTCGTTACGGTTTGCTAATTCTCGGAATAACAT	22 (0.000416%)	
CGTTGACTCGCACACATGTCAGACTCCTTGGTCCGTGTTTCAAGACGGGTCGAATGGGGAGCCCACAGGCCGACGCCCGGAGCACGCTGATGCCGAGGCA	13 (0.000246%)	
CTAAATACGGGCGAGAGACCGATAGCGAACAAGTACCGCGAGGTAAAGATGAAAAGGACTTTGAAAAGAGAGTCAAAGAGTGCTTGAAATTGTCGGGAGG	705 (0.013337%)	
CTAACGGCGTGCCTCGGCATCAGCGTGCTCCGGGCGTCGGCCTGTGGGCTCCCCATTCGACCCGTCTTGAAACACGGACCAAGGAGTCTGACATGTGTGC	100 (0.001892%)	
CTAACTAGCTACGTGGAGGCATCCCTTCACGGCCGGCTTCTTAGAGGGACTATGGCCGTTTAGGCCAAGGAAGTTTGAGGCAATAACAGGTCTGTGATGC	51 (0.000965%)	
CTAAGTAGTGTTTCCTTGTTAGAAGACACAAAGCCAAAGACTCATATGGACTTTGGCTACACCATGAAAGCTTTGAGAAGCAAGAAGAAGGTTGGTTAGT	56 (0.001059%)	
CTAATGTATCCAGAGCGTAGGCTTGCTTTGAGCACTCTAATTTCTTCAAAGTAACAGCGCCGGAGGCACGACCCGGCCAATTAAGACCAGGAGCGTATCG	45 (0.000851%)	
CTACATTGTTCCATCGACCAGAGGCTGTTCACCTTGGAGACCTGATGCGGTTATGAGTACGACCGGGCGTGAGCGGCACTCGGTCCTCCGGATTTTCAAG	35 (0.000662%)	
CTACCTACCACCTATCCTTCTCAGTTAATTCACGGGCGATGTTACGCTCGATGATGAGTTCCGGGGCCTGTGTTTCGTACCTAATTTGAAGGAATTGTTG	11 (0.000208%)	
CTAGCTACGTGGAGGCATCCCTTCACGGCCGGCTTCTTAGAGGGACTATGGCCGTTTAGGCCAAGGAAGTTTGAGGCAATAACAGGTCTGTGATGCCCTT	25 (0.000473%)	
CTAGGCGAGACAAGGGTTCACATTTCGTTCATCACCCTTGGCCGGCTATCGAACAGCCGGACTCCCATCAAAAGATGGTTGCCAAGAACATCTTCGTTAC	32 (0.000605%)	
CTAGGCGAGACAAGGGTTCACATTTCGTTCATCACCCTTGGCCGGCTTTCGAACAGCCGGACTCCCATCAAAAGATGGTTGCCAAGAACATCTTCGTTAC	61 (0.001154%)	
CTAGGCTGTCCCGAGTGTGAGCGAGGTGTGAGTGTCGCCCATGGGCATCGACACCTTGCGGCTAGGAACTGGAACGAGACGGGTGGCAAAGATTTCGAGT	411 (0.007775%)	
CTAGTGTTCGTAGGCTCCATGCTCGCGCATCGAACTACCTACCACCTATCCTTCTCAGTTAATTCACGGGCGATGTTACGCTCGATGATGAGTTCCGGGG	12 (0.000227%)	
CTATGATGTTATCCCATGCTAATGTATCCAGAGCGTAGGCTTGCTTTGAGCACTCTAATTTCTTCAAAGTAACAGCGCCGGAGGCACGACCCGGCCAATT	211 (0.003992%)	
CTCAAAATCATCAATCGTTCCAACTAATCTACCGAAGTACTCGGCTAAGAAGAAAGAAGACGGACGAATCCGAGCCAAAGCCGTACAAGCGCGAGATACC	1987 (0.037591%)	
CTCAAAGATTAAGCCATGCATGTGTAAGTATGAACGAATTCAGACTGTGAAACTGCGAATGGCTCATTAAATCAGTTATAGTTTGTTTGATGGTAACTAC	13 (0.000246%)	
CTCAATCATACACATGACATCAAGTCATATTCGACTCCAAAACACTAACCAACCTTCTTCTTGCTTCTCAAAGCTTTCATGGTGTAGCCAAAGTCCATAT	73 (0.001381%)	
CTCACCATCCTTTGCTGATGCGGGACGGAAGCTGGTCTCCCGTGTGTTACCGCACGCGGTTGGCCTAAATCCGAGCCAAGGACGCCTGGAGCGTACCGAC	16 (0.000303%)	

CTCACCCGTTGACTCGCACACATGTCAGACTCCTTGGTCCGTGTTTCAAGACGGGTCGAATGGGGAGCCCACAGGCCGACGCCCGGAGCACGCTGATGCC	13 (0.000246%)
CTCAGCCTGCTAACTAGCTACGTGGAGGCATCCCTTCACGGCCGGCTTCTTAGAGGGACTATGGCCGTTTAGGCCAAGGAAGTTTGAGGCAATAACAGGT	37 (0.000700%)
CTCATGTGTATGATTGAGTATAAGAACTTAAACCGCAACCGCATCTTATAAGCCTAAGTAGTGTTTCCTTGTTAGAAGACACAAAGCCAAAGACTCATAT	105 (0.001986%)
CTCCAAAACACTAACCAACCTTCTTCTTGCTTCTCAAAGCTTTCATGGTGTAGCCAAAGTCCATATGAGTCTTTGGCTTTGTGTCTTCTAACAAGGATAC	19 (0.000359%)
CTCCAATAGCGTATATTTAAGTTGTTGCAGTTAAAAAGCTCGTAGTTGAACCTTGGGATGGGTCGGCCGGTCCGCCTTTGGTGTGCATTGGTCGGCTTGT	32 (0.000605%)
CTCCATGCTCGCGCATCGAACTACCTACCACCTATCCTTCTCAGTTAATTCACGGGCGATGTTACGCTCGATGATGAGTTCCGGGGCCTGTGTTTCGTAC	11 (0.000208%)
CTCCGGATTTTCAAGGGCCGCCGGGGGCGCACCGGACACCACGCGACGTGCGGTGCTCTTCCAGCCGCTGGACCCTACCTCCGGCTGAGCCGTTTCCAGG	3 (0.000057%)
CTCCGGGCGTCGGCCTGTGGGCTCCCCATTCGACCCGTCTTGAAACACGGACCAAGGAGTCTGACATGTGTGCGAGTCAACGGGTGAGTAAACCCGTAAG	10 (0.000189%)
CTCGCACACATGTCAGACTCCTTGGTCCGTGTTTCAAGACGGGTCGAATGGGGAGCCCACAGGCCGACGCCCGGAGCACGCTGATGCCGAGGCACGCCGT	22 (0.000416%)
CTCGCGCTTGTACGGCTTTGGCTCGGATTCGTCCGTCTTCTTTCTTCTTAGCCGAGTACTTCGGTAGATTAGTTGGAACGATTGATGATTTTGAGTTAAT	11 (0.000208%)
CTCGGCATCAGCGTGCTCCGGGCGTCGGCCTGTGGGCTCCCCATTCGACCCGTCTTGAAACACGGACCAAGGAGTCTGACATGTGTGCGAGTCAACGGGT	29 (0.000549%)
CTCGGTCCTCCGGATTTTCAAGGGCCGCCGGGGGCGCACCGGACACCACGCGACGTGCGGTGCTCTTCCAGCCGCTGGACCCTACCTCCGGCTGAGCCGT	22 (0.000416%)
CTCGTGTAAAGTTGGGAATTCGTTAAGGAGCTGTTGCTTTGTTAGTGTAGAAACACTTGTGTAGAATTGGGGATTGTTTTTTTTGGAGTGATTTAGGGGA	39 (0.000738%)
CTCTGTGCTGGCGACGCATCATTCAAATTTCTGCCCTATCAACTTTCGATGGTAGGATAGTGGCCTACCATGGTGGTAACGGGTGACGGAGAATTAGGGT	57 (0.001078%)
CTGAGAAGGGTTCGAGTGTGAGCATGCCTGTCGGGACCCGAAAGATGGTGAACTATGCCTGAGCGGGGTAAAGCCAGAGGAAACTCTGGTGGAAGCCCGC	27 (0.000511%)
CTGATGCGGGACGGAAGCTGGTCTCCCGTGTGTTACCGCACGCGGTTGGCCTAAATCCGAGCCAAGGACGCCTGGAGCGTACCGACATGCGGTGGTGAAC	29 (0.000549%)
CTGCAGCACGCGCCTAACGGCGTGCCTCGGCATCAGCGTGCTCCGGGCGTCGGCCTGTGGGCTCCCCATTCGACCCGTCTTGAAACACGGACCAAGGAGT	2138 (0.040447%)
CTGCCCTATCAACTTTCGATGGTAGGATAGTGGCCTACCATGGTGGTAACGGGTGACGGAGAATTAGGGTTCGATTCCGGAGAGGGAGCCTGAGAAACGG	14 (0.000265%)
CTGCTAACTAGCTACGTGGAGGCATCCCTTCACGGCCGGCTTCTTAGAGGGACTATGGCCGTTTAGGCCAAGGAAGTTTGAGGCAATAACAGGTCTGTGA	56 (0.001059%)
CTGGCGACGCATCATTCAAATTTCTGCCCTATCAACTTTCGATGGTAGGATAGTGGCCTACCATGGTGGTAACGGGTGACGGAGAATTAGGGTTCGATTC	24 (0.000454%)
CTGGGGCGGCACATCTGTTAAAAGATAACGCAGGTGTCCTAAGATGAGCTCAACGAGAACAGAAATCTCGTGTGGAACAAAAGGGTAAAAGCTCGTTTGA	27 (0.000511%)
CTGGGTGTCACAAATCGTCGTCCCTCACCATCCTTTGCTGATGCGGGACGGAAGCTGGTCTCCCGTGTGTTACCGCACGCGGTTGGCCTAAATCCGAGCC	826 (0.015626%)
CTGGTGCCAGCAGCCGCGGTAATTCCAGCTCCAATAGCGTATATTTAAGTTGTTGCAGTTAAAAAGCTCGTAGTTGAACCTTGGGATGGGTCGGCCGGTC	31 (0.000586%)
CTGTCCCGAAGGTATCTCGCGCTTGTACGGCTTTGGCTCGGATTCGTCCGTCTTCTTTCTTCTTAGCCGAGTACTTCGGTAGATTAGTTGGAACGATTGA	61 (0.001154%)
CTGTCCCGAGTGTGAGCGAGGTGTGAGTGTCGCCCATGGGCATCGACACCTTGCGGCTAGGAACTGGAACGAGACGGGTAGCAAAGATTTCGAGTAGCAC	23 (0.000435%)
CTGTCCCGAGTGTGAGCGAGGTGTGAGTGTCGCCCATGGGCATCGACACCTTGCGGCTAGGAACTGGAACGAGACGGGTGGCAAAGATTTCGAGTAGCAC	28 (0.000530%)
CTGTGCTGGCGACGCATCATTCAAATTTCTGCCCTATCAACTTTCGATGGTAGGATAGTGGCCTACCATGGTGGTAACGGGTGACGGAGAATTAGGGTTC	33 (0.000624%)
CTGTTAAAAGATAACGCAGGTGTCCTAAGATGAGCTCAACGAGAACAGAAATCTCGTGTGGAACAAAAGGGTAAAAGCTCGTTTGATTCTGATTTTCAGT	5 (0.000095%)
CTGTTGCTTTGTTAGTGTAGAAACACTTGTGTAGAATTGGGGATTGTTTTTTTTGGAGTGATTTAGGGGAGGGTCGAATCTTAGCGACAAAGGGCTGAAT	19 (0.000359%)
CTTAAAGGCGTAAGAATTGTATCCTTGTTAGAAGACACAA	1035 (0.007832%)
CTTACGGGTTTACTCACCCGTTGACTCGCACACATGTCAGACTCCTTGGTCCGTGTTTCAAGACGGGTCGAATGGGGAGCCCACAGGCCGACGCCCGGAG	13 (0.000246%)
CTTATACTCAATCATACACATGACATCAAGTCATATTCGACTCCAAAACACTAACCAACCTTCTTCTTGCTTCTCAAAGCTTTCATGGTGTAGCCAAAGT	148 (0.002800%)
CTTCAAATTAGGTACGAAACACAGGCCCCGGAACTCATCATCGAGCGTAACATCGCCCGTGAATTAACTGAGAAGGATAGGTGGTAGGTAGTTCGATGCG	771 (0.014586%)
CTTCAACGAGGAATTCCTAGTAAGCGCGAGTCATCAGCTCGCGTTGACTACGTCCCTGCCCTTTGTACACACCGCCCGTCGCTCCTACCGATTGAATGAT	59 (0.001116%)
CTTCCCTTGCCTACATTGTTCCATCGACCAGAGGCTGTTCACCTTGGAGACCTGATGCGGTTATGAGTACGACCGGGCGTGAGCGGCACTCGGTCCTCCG	53 (0.001003%)
CTTCCTTGCGCCTTACGGGTTTACTCACCCGTTGACTCGCACACATGTCAGACTCCTTGGTCCGTGTTTCAAGACGGGTCGAATGGGGAGCCCACAGGCC	100 (0.001892%)
CTTCTGAGAAGGGTTCGAGTGTGAGCATGCCTGTCGGGACCCGAAAGATGGTGAACTATGCCTGAGCGGGGTAAAGCCAGAGGAAACTCTGGTGGAAGCC	14 (0.000265%)
CTTGCCTACATTGTTCCATCGACCAGAGGCTGTTCACCTTGGAGACCTGATGCGGTTATGAGTACGACCGGGCGTGAGCGGCACTCGGTCCTCCGGATTT	33 (0.000624%)
CTTGCGCCTTACGGGTTTACTCACCCGTTGACTCGCACACATGTCAGACTCCTTGGTCCGTGTTTCAAGACGGGTCGAATGGGGAGCCCACAGGCCGACG	16 (0.000303%)
CTTGGAGACCTGATGCGGTTATGAGTACGACCGGGCGTGAGCGGCACTCGGTCCTCCGGATTTTCAAGGGCCGCCGGGGGCGCACCGGACACCACGCGAC	131 (0.002478%)
CTTGGTGATATGAACACAAACGTTCAATATGACAAACCCATGCCAAGTAAAGAGAAAATGAAAACTGGTGATTGTTGCGGAAATCGTCCAGGATTCCTCG	16 (0.000303%)
CTTGTACGGCTTTGGCTCGGATTCGTCCGTCTTCTTTCTTCTTAGCCGAGTACTTCGGTAGATTAGTTGGAACGATTGATGATTTTGAGTTAATTGAACG	14 (0.000265%)
CTTGTCTCAAAGATTAAGCCATGCATGTGTAAGTATGAACGAATTCAGACTGTGAAACTGCGAATGGCTCATTAAATCAGTTATAGTTTGTTTGATGGTA	69 (0.001305%)

CTTGTTAGAAGACACAAAGCCAAAGACTCATATGGACTTTGGCTACACCATGAAAGCTTTGAGAAGCAAGAAGAAGGTTGGTTAGTGTTTTGGAGTCGAA	109 (0.002062%)	
CTTTCACGGTTCGTATTCGTACTGAAAATCAGAATCAAACGAGCTTTTACCCTTTTGTTCCACACGAGATTTCTGTTCTCGTTGAGCTCATCTTAGGACA	35 (0.000662%)	
CTTTCGATGGTAGGATAGTGGCCTACCATGGTGGTAACGGGTGACGGAGAATTAGGGTTCGATTCCGGAGAGGGAGCCTGAGAAACGGCTACCACATCCA	8 (0.000151%)	
CTTTGCTGATGCGGGACGGAAGCTGGTCTCCCGTGTGTTACCGCACGCGGTTGGCCTAAATCCGAGCCAAGGACGCCTGGAGCGTACCGACATGCGGTGG	60 (0.001135%)	
CTTTGGCTCGGATTCGTCCGTCTTCTTTCTTCTTAGCCGAGTACTTCGGTAGATTAGTTGGAACGATTGATGATTTTGAGTTAATTGAACGTTCGGCGTA	6 (0.000114%)	
CTTTGTTTTAATTAAACAGTCGGATTCCCCTTGTCCGTACCAGTTCTGAGCTGACTGTTCGACGCCCGGGGAAAGCTCCCGAGAGAGCCGTTCCCAGTCC	77 (0.001457%)	
GAAACACAGGCCCCGGAACTCATCATCGAGCGTAACATCGCCCGTGAATTAACTGAGAAGGATAGGTGGTAGGTAGTTCGATGCGCGAGCATGGAGCCTA	72 (0.001362%)	
GAAATCCTATGATGTTATCCCATGCTAATGTATCCAGAGCGTAGGCTTGCTTTGAGCACTCTAATTTCTTCAAAGTAACAGCGCCGGAGGCACGACCCGG	175 (0.003311%)	
GAACCCACAAAGGGTGTTGGTCGATTAAGACAGCAGGACGGTGGTCATGGAAGTCGAAATCCGCTAAGGAGTGTGTAACAACTCACCTGCCGAATCAACT	884 (0.016724%)	
GAACCGGGACGTGGCGGTTGACGGCAACGTTAGGGAGTCCGGAGACGTCGGCGGGGGCCTCGGGAAGAGTTATCTTTTCTGTTTAACAGCCTGCCCACCC	7 (0.000132%)	
GAAGACACAAAGCCAAAGACTCATATGGACTTTGGCTACACCATGAAAGCTTTGAGAAGCAAGAAGAAGGTTGGTTAGTGTTTTGGAGTCGAATATGACT	35 (0.000662%)	
GAAGAGCGTCGTGTAGGGAAAGAGTGTTCAGAGCCGTGTA	21 (0.000159%)	
GAAGCACGCCCATCCAACCTAGGCGAGACAAGGGTTCACATTTCGTTCATCACCCTTGGCCGGCTATCGAACAGCCGGACTCCCATCAAAAGATGGTTGC	13 (0.000246%)	
GAAGGTATCTCGCGCTTGTACGGCTTTGGCTCGGATTCGTCCGTCTTCTTTCTTCTTAGCCGAGTACTTCGGTAGATTAGTTGGAACGATTGATGATTTT	10 (0.000189%)	
GAAGTCGAAATCCGCTAAGGAGTGTGTAACAACTCACCTGCCGAATCAACTAGCCCCGAAAATGGATGGCGCTTAAGCGCGCGACCTATACCCGGCCGTC	4 (0.000076%)	
GAATCATCAGAGCAACGGGCAGAGCCCGCGTCGACCTTTTATCTAATAAATGCGTCCCTTCCATAAGTCGGGGTTTGTTGCACGTATTAGCTCTAGAATT	104 (0.001967%)	
GAATTGTATCCTTGTTAGAAGACACAAAGCCAAAGACTCATATGGACTTTGGCTACACCATGAAAGCTTTGAGAAGCAAGAAGAAGGTTGGTTAGTGTTT	18 (0.000341%)	
GACAAGGGTTCACATTTCGTTCATCACCCTTGGCCGGCTATCGAACAGCCGGACTCCCATCAAAAGATGGTTGCCAAGAACATCTTCGTTACGGTTTGCT	14 (0.000265%)	
GACAAGGGTTCACATTTCGTTCATCACCCTTGGCCGGCTTTCGAACAGCCGGACTCCCATCAAAAGATGGTTGCCAAGAACATCTTCGTTACGGTTTGCT	9 (0.000170%)	
GACACAAAGCCAAAGACTCATATGGACTTTGGCTACACCATGAAAGCTTTGAGAAGCAAGAAGAAGGTTGGTTAGTGTTTTGGAGTCGAATATGACTTGA	49 (0.000927%)	
GACAGCAGGACGGTGGTCATGGAAGTCGAAATCCGCTAAGGAGTGTGTAACAACTCACCTGCCGAATCAACTAGCCCCGAAAATGGATGGCGCTTAAGCG	7 (0.000132%)	
GACAGTCGGGGGCATTCGTATTTCATAGTCAGAGGTGAAATTCTTGGATTTATGAAAGACGAACAACTGCGAAAGCATTTGCCAAGGATGTTTTCATTAA	7 (0.000132%)	
GACATCAAGTCATATTCGACTCCAAAACACTAACCAACCTTCTTCTTGCTTCTCAAAGCTTTCATGGTGTAGCCAAAGTCCATATGAGTCTTTGGCTTTG	45 (0.000851%)	
GACATTGTCAGGTGGGGAGTTTGGCTGGGGCGGCACATCTGTTAAAAGATAACGCAGGTGTCCTAAGATGAGCTCAACGAGAACAGAAATCTCGTGTGGA	36 (0.000681%)	
GACCAGGGGTTGAAATCGTCGACCAGGTCCGAGACTTCATCGACCGGGTCCGAGGATTCGTCGACCAGGACGGCCGGATGTCCGAGAAAAAAAAATGTTG	6 (0.000114%)	
GACCCGCCGAAGCGAGCCTTGGGACCAAAAACAGGGGTTGTACCCCGCCTCCGATTCACGGAGTAAGTAAAATAACGTTAAAAGTAGTGGTATTTCACTT	4 (0.000076%)	
GACCTCAGCCTGCTAACTAGCTACGTGGAGGCATCCCTTCACGGCCGGCTTCTTAGAGGGACTATGGCCGTTTAGGCCAAGGAAGTTTGAGGCAATAACA	6 (0.000114%)	
GACGCATCATTCAAATTTCTGCCCTATCAACTTTCGATGGTAGGATAGTGGCCTACCATGGTGGTAACGGGTGACGGAGAATTAGGGTTCGATTCCGGAG	21 (0.000397%)	
GACGGGCGGTGTGTACAAAGGGCAGGGACGTAGTCAACGCGAGCTGATGACTCGCGCTTACTAGGAATTCCTCGTTGAAGACCAACAATTGCAATGATCG	17 (0.000322%)	
GACGGTGGTCATGGAAGTCGAAATCCGCTAAGGAGTGTGTAACAACTCACCTGCCGAATCAACTAGCCCCGAAAATGGATGGCGCTTAAGCGCGCGACCT	9 (0.000170%)	
GACGTGGGTGGTTCGCCGCCCGCGACGTCGCGAGAAGTCCACTAAACCTTATCATTTAGAGGAAGGAGAAGTCGTAACAAGGTTTCCGTAGGTGAACCTG	47 (0.000889%)	
GACTCCAAAACACTAACCAACCTTCTTCTTGCTTCTCAAAGCTTTCATGGTGTAGCCAAAGTCCATATGAGTCTTTGGCTTTGTGTCTTCTAACAAGGAT	30 (0.000568%)	
GACTTCCCTTGCCTACATTGTTCCATCGACCAGAGGCTGTTCACCTTGGAGACCTGATGCGGTTATGAGTACGACCGGGCGTGAGCGGCACTCGGTCCTC	22 (0.000416%)	
GAGAAGGGTTCGAGTGTGAGCATGCCTGTCGGGACCCGAAAGATGGTGAACTATGCCTGAGCGGGGTAAAGCCAGAGGAAACTCTGGTGGAAGCCCGCAG	8 (0.000151%)	
GAGACAAGGGTTCACATTTCGTTCATCACCCTTGGCCGGCTATCGAACAGCCGGACTCCCATCAAAAGATGGTTGCCAAGAACATCTTCGTTACGGTTTG	8 (0.000151%)	
GAGACCTCAGCCTGCTAACTAGCTACGTGGAGGCATCCCTTCACGGCCGGCTTCTTAGAGGGACTATGGCCGTTTAGGCCAAGGAAGTTTGAGGCAATAA	972 (0.018389%)	
GAGACGTCGGCGGGGGCCTCGGGAAGAGTTATCTTTTCTGTTTAACAGCCTGCCCACCCTGGAAACGGCTCAGCCGGAGGTAGGGTCCAGCGGCTGGAAG	17 (0.000322%)	
GAGCAACGGGCAGAGCCCGCGTCGACCTTTTATCTAATAAATGCGTCCCTTCCATAAGTCGGGGTTTGTTGCACGTATTAGCTCTAGAATTACTACGGTT	14 (0.000265%)	
GAGCCCGCGTCGACCTTTTATCTAATAAATGCGTCCCTTCCATAAGTCGGGGTTTGTTGCACGTATTAGCTCTAGAATTACTACGGTTATCCGAGTAGTA	6 (0.000114%)	
GAGCGACGGGCGGTGTGTACAAAGGGCAGGGACGTAGTCAACGCGAGCTGATGACTCGCGCTTACTAGGAATTCCTCGTTGAAGACCAACAATTGCAATG	9 (0.000170%)	
GAGCGAGGTGTGAGTGTCGCCCATGGGCATCGACACCTTGCGGCTAGGAACTGGAACGAGACGGGTGGCAAAGATTTCGAGTAGCACTTCATACTACCGT	10 (0.000189%)	
GAGCGGCACTCGGTCCTCCGGATTTTCAAGGGCCGCCGGGGGCGCACCGGACACCACGCGACGTGCGGTGCTCTTCCAGCCGCTGGACCCTACCTCCGGC	13 (0.000246%)	
GAGCGTAGGCTTGCTTTGAGCACTCTAATTTCTTCAAAGTAACAGCGCCGGAGGCACGACCCGGCCAATTAAGACCAGGAGCGTATCGCCGACCGAAGGG	4 (0.000076%)	

GAGCGTCGTGTAGGGAAAGAGTGTTCAGAGCCGTGTAGAT	17 (0.000129%)	
GAGCTCGTGTAAAGTTGGGAATTCGTTAAGGAGCTGTTGCTTTGTTAGTGTAGAAACACTTGTGTAGAATTGGGGATTGTTTTTTTTGGAGTGATTTAGG	4 (0.000076%)	
GAGCTGTTGCTTTGTTAGTGTAGAAACACTTGTGTAGAATTGGGGATTGTTTTTTTTGGAGTGATTTAGGGGAGGGTCGAATCTTAGCGACAAAGGGCTG	7 (0.000132%)	
GAGGACATTGTCAGGTGGGGAGTTTGGCTGGGGCGGCACATCTGTTAAAAGATAACGCAGGTGTCCTAAGATGAGCTCAACGAGAACAGAAATCTCGTGT	53 (0.001003%)	
GAGGCTGTTCACCTTGGAGACCTGATGCGGTTATGAGTACGACCGGGCGTGAGCGGCACTCGGTCCTCCGGATTTTCAAGGGCCGCCGGGGGCGCACCGG	10 (0.000189%)	
GAGGGCAAGTCTGGTGCCAGCAGCCGCGGTAATTCCAGCTCCAATAGCGTATATTTAAGTTGTTGCAGTTAAAAAGCTCGTAGTTGAACCTTGGGATGGG	28 (0.000530%)	
GAGGTGTGAGTGTCGCCCATGGGCATCGACACCTTGCGGCTAGGAACTGGAACGAGACGGGTAGCAAAGATTTCGAGTAGCACTTCATACTACCGTGGGT	68 (0.001286%)	
GAGGTGTGAGTGTCGCCCATGGGCATCGACACCTTGCGGCTAGGAACTGGAACGAGACGGGTGGCAAAGATTTCGAGTAGCACTTCATACTACCGTGGGT	7 (0.000132%)	
GAGTATAAGAACTTAAACCGCAACCGCATCTTATAAGCCTAAGTAGTGTTTCCTTGTTAGAAGACACAAAGCCAAAGACTCATATGGACTTTGGCTACAC	7 (0.000132%)	
GAGTCGAATATGACTTGATGTCATGTGTATGATTGAGTAT	1492 (0.011290%)	
GAGTGTCGCCCATGGGCATCGACACCTTGCGGCTAGGAACTGGAACGAGACGGGTGGCAAAGATTTCGAGTAGCACTTCATACTACCGTGGGTTTTTTAA	4 (0.000076%)	
GAGTGTGAGCGAGGTGTGAGTGTCGCCCATGGGCATCGACACCTTGCGGCTAGGAACTGGAACGAGACGGGTGGCAAAGATTTCGAGTAGCACTTCATAC	5 (0.000095%)	
GAGTTATCATGAATCATCAGAGCAACGGGCAGAGCCCGCGTCGACCTTTTATCTAATAAATGCGTCCCTTCCATAAGTCGGGGTTTGTTGCACGTATTAG	62 (0.001173%)	
GATAGCTAGTGTTCGTAGGCTCCATGCTCGCGCATCGAACTACCTACCACCTATCCTTCTCAGTTAATTCACGGGCGATGTTACGCTCGATGATGAGTTC	23 (0.000435%)	
GATCCATTACATTTTATCGGTCGCTCTTGTCCGGAAGCTGTAGATGACCCAAAGTCCATATAGCGACCCCAGGTCAGGCGGGATTACCCGCTGAGTTTAA	28 (0.000530%)	
GATCCCGCCAATCAGCTTCCTTGCGCCTTACGGGTTTACTCACCCGTTGACTCGCACACATGTCAGACTCCTTGGTCCGTGTTTCAAGACGGGTCGAATG	562 (0.010632%)	
GATCCGTCGAGTTATCATGAATCATCAGAGCAACGGGCAGAGCCCGCGTCGACCTTTTATCTAATAAATGCGTCCCTTCCATAAGTCGGGGTTTGTTGCA	117 (0.002213%)	
GATCGAAATCCTATGATGTTATCCCATGCTAATGTATCCAGAGCGTAGGCTTGCTTTGAGCACTCTAATTTCTTCAAAGTAACAGCGCCGGAGGCACGAC	1570 (0.029702%)	
GATCGACCCGCCGAAGCGAGCCTTGGGACCAAAAACAGGGGTTGTACCCCGCCTCCGATTCACGGAGTAAGTAAAATAACGTTAAAAGTAGTGGTATTTC	33 (0.000624%)	
GATCGATCAA	169469 (0.320605%)	
GATCGATCCATTACATTTTATCGGTCGCTCTTGTCCGGAAGCTGTAGATGACCCAAAGTCCATATAGCGACCCCAGGTCAGGCGGGATTACCCGCTGAGT	1200 (0.022702%)	
GATCGATCCGTAACTTCGGGAAAAGGATTGGCTCTGAGGGCTGGGCTCGGGGGTCCCAGTTCCGAACCCGTCGGCTGTCAGCGGACTGCTCGAGCTGCTT	1226 (0.023194%)	
GATCGATCCGTCGAGTTATCATGAATCATCAGAGCAACGGGCAGAGCCCGCGTCGACCTTTTATCTAATAAATGCGTCCCTTCCATAAGTCGGGGTTTGT	1171 (0.022153%)	
GATCGATCGACCCGCCGAAGCGAGCCTTGGGACCAAAAACAGGGGTTGTACCCCGCCTCCGATTCACGGAGTAAGTAAAATAACGTTAAAAGTAGTGGTA	948 (0.017934%)	
GATCGATCTCATGTGTATGATTGAGTATAAGAACTTAAACCGCAACCGCATCTTATAAGCCTAAGTAGTGTTTCCTTGTTAGAAGACACAAAGCCAAAGA	207 (0.003916%)	
GATCGATCTT	189995 (0.359437%)	
GATCGATCTTAAAGGCGTAAGAATTGTATCCTTGTTAAAAGACACAAAGCCAAAGACTCATATGGACTTTGGCTACACCATGAAAGCTTTGAGAAGCAAG	488 (0.009232%)	
GATCGATCTTAAAGGCGTAAGAATTGTATCCTTGTTAGAA	4489 (0.033970%)	
GATCGATCTTCTGAGAAGGGTTCGAGTGTGAGCATGCCTGTCGGGACCCGAAAGATGGTGAACTATGCCTGAGCGGGGTAAAGCCAGAGGAAACTCTGGT	1117 (0.021132%)	
GATCGGAAGAGCGTCGTGTAGGGAAAGAGTGTTCAGAGCC	7 (0.000053%)	
GATCTCATGTGTATGATTGAGTATAAGAACTTAAACCGCAACCGCATCTTATAAGCCTAAGTAGTGTTTCCTTGTTAGAAGACACAAAGCCAAAGACTCA	56 (0.001059%)	
GATCTTAAAGGCGTAAGAATTGTATCCTTGTTAAAAGACACAAAGCCAAAGACTCATATGGACTTTGGCTACACCATGAAAGCTTTGAGAAGCAAGAAGA	233 (0.004408%)	
GATCTTCTGAGAAGGGTTCGAGTGTGAGCATGCCTGTCGGGACCCGAAAGATGGTGAACTATGCCTGAGCGGGGTAAAGCCAGAGGAAACTCTGGTGGAA	137 (0.002592%)	
GATGTTATCCCATGCTAATGTATCCAGAGCGTAGGCTTGCTTTGAGCACTCTAATTTCTTCAAAGTAACAGCGCCGGAGGCACGACCCGGCCAATTAAGA	10 (0.000189%)	
GATTAACAGGGACAGTCGGGGGCATTCGTATTTCATAGTCAGAGGTGAAATTCTTGGATTTATGAAAGACGAACAACTGCGAAAGCATTTGCCAAGGATG	171 (0.003235%)	
GATTAAGACAGCAGGACGGTGGTCATGGAAGTCGAAATCCGCTAAGGAGTGTGTAACAACTCACCTGCCGAATCAACTAGCCCCGAAAATGGATGGCGCT	41 (0.000776%)	
GATTAGTCTTTCGCCCCTATACCCAAGTCAGACGAACGATTTGCACGTCAGTATCGCTGCGGGCTTCCACCAGAGTTTCCTCTGGCTTTACCCCGCTCAG	11 (0.000208%)	
GATTGAGTATAAGAACTTAAACCGCAACCGCATCTTATAAGCCTAAGTAGTGTTTCCTTGTTAGAAGACACAAAGCCAAAGACTCATATGGACTTTGGCT	38 (0.000719%)	
GATTTGCACGTCAGTATCGCTGCGGGCTTCCACCAGAGTTTCCTCTGGCTTTACCCCGCTCAGGCATAGTTCACCATCTTTCGGGTCCCGACAGGCATGC	26 (0.000492%)	
GATTTTCAAGGGCCGCCGGGGGCGCACCGGACACCACGCGACGTGCGGTGCTCTTCCAGCCGCTGGACCCTACCTCCGGCTGAGCCGTTTCCAGGGTGGG	14 (0.000265%)	
GCAACGGGCAGAGCCCGCGTCGACCTTTTATCTAATAAATGCGTCCCTTCCATAAGTCGGGGTTTGTTGCACGTATTAGCTCTAGAATTACTACGGTTAT	38 (0.000719%)	
GCAACGTTAGGGAGTCCGGAGACGTCGGCGGGGGCCTCGGGAAGAGTTATCTTTTCTGTTTAACAGCCTGCCCACCCTGGAAACGGCTCAGCCGGAGGTA	19 (0.000359%)	
GCAAGTCTGGTGCCAGCAGCCGCGGTAATTCCAGCTCCAATAGCGTATATTTAAGTTGTTGCAGTTAAAAAGCTCGTAGTTGAACCTTGGGATGGGTCGG	12 (0.000227%)	

GCACACATGTCAGACTCCTTGGTCCGTGTTTCAAGACGGGTCGAATGGGGAGCCCACAGGCCGACGCCCGGAGCACGCTGATGCCGAGGCACGCCGTTAG	4 (0.000076%)	
GCACCGGACACCACGCGACGTGCGGTGCTCTTCCAGCCGCTGGACCCTACCTCCGGCTGAGCCGTTTCCAGGGTGGGCAGGCTGTTAAACAGAAAAGATA	31 (0.000586%)	
GCACGCCCATCCAACCTAGGCGAGACAAGGGTTCACATTTCGTTCATCACCCTTGGCCGGCTATCGAACAGCCGGACTCCCATCAAAAGATGGTTGCCAA	41 (0.000776%)	
GCACGCGCCTAACGGCGTGCCTCGGCATCAGCGTGCTCCGGGCGTCGGCCTGTGGGCTCCCCATTCGACCCGTCTTGAAACACGGACCAAGGAGTCTGAC	25 (0.000473%)	
GCACTCGGTCCTCCGGATTTTCAAGGGCCGCCGGGGGCGCACCGGACACCACGCGACGTGCGGTGCTCTTCCAGCCGCTGGACCCTACCTCCGGCTGAGC	4 (0.000076%)	
GCAGAGCCCGCGTCGACCTTTTATCTAATAAATGCGTCCCTTCCATAAGTCGGGGTTTGTTGCACGTATTAGCTCTAGAATTACTACGGTTATCCGAGTA	12 (0.000227%)	
GCAGCACGCGCCTAACGGCGTGCCTCGGCATCAGCGTGCTCCGGGCGTCGGCCTGTGGGCTCCCCATTCGACCCGTCTTGAAACACGGACCAAGGAGTCT	427 (0.008078%)	
GCAGCCGCGGTAATTCCAGCTCCAATAGCGTATATTTAAGTTGTTGCAGTTAAAAAGCTCGTAGTTGAACCTTGGGATGGGTCGGCCGGTCCGCCTTTGG	13 (0.000246%)	
GCAGGACGGTGGTCATGGAAGTCGAAATCCGCTAAGGAGTGTGTAACAACTCACCTGCCGAATCAACTAGCCCCGAAAATGGATGGCGCTTAAGCGCGCG	42 (0.000795%)	
GCATCAGCGTGCTCCGGGCGTCGGCCTGTGGGCTCCCCATTCGACCCGTCTTGAAACACGGACCAAGGAGTCTGACATGTGTGCGAGTCAACGGGTGAGT	10 (0.000189%)	
GCATCATTCAAATTTCTGCCCTATCAACTTTCGATGGTAGGATAGTGGCCTACCATGGTGGTAACGGGTGACGGAGAATTAGGGTTCGATTCCGGAGAGG	42 (0.000795%)	
GCATCGAACTACCTACCACCTATCCTTCTCAGTTAATTCACGGGCGATGTTACGCTCGATGATGAGTTCCGGGGCCTGTGTTTCGTACCTAATTTGAAGG	5 (0.000095%)	
GCATCGACACCTTGCGGCTAGGAACTGGAACGAGACGGGTGGCAAAGATTTCGAGTAGCACTTCATACTACCGTGGGTTTTTTAAACCTTCCGAGTTTTG	6 (0.000114%)	
GCATGGCCTCTGTGCTGGCGACGCATCATTCAAATTTCTGCCCTATCAACTTTCGATGGTAGGATAGTGGCCTACCATGGTGGTAACGGGTGACGGAGAA	1694 (0.032047%)	
GCCAAAGACTCATATGGACTTTGGCTACACCATGAAAGCTTTGAGAAGCAAGAAGAAGGTTGGTTAGTGTTTTGGAGTCGAATATGACTTGATGTCATGT	48 (0.000908%)	
GCCAATCAGCTTCCTTGCGCCTTACGGGTTTACTCACCCGTTGACTCGCACACATGTCAGACTCCTTGGTCCGTGTTTCAAGACGGGTCGAATGGGGAGC	120 (0.002270%)	
GCCAGCAGCCGCGGTAATTCCAGCTCCAATAGCGTATATTTAAGTTGTTGCAGTTAAAAAGCTCGTAGTTGAACCTTGGGATGGGTCGGCCGGTCCGCCT	13 (0.000246%)	
GCCCATCCAACCTAGGCGAGACAAGGGTTCACATTTCGTTCATCACCCTTGGCCGGCTATCGAACAGCCGGACTCCCATCAAAAGATGGTTGCCAAGAAC	58 (0.001097%)	
GCCCATCCAACCTAGGCGAGACAAGGGTTCACATTTCGTTCATCACCCTTGGCCGGCTTTCGAACAGCCGGACTCCCATCAAAAGATGGTTGCCAAGAAC	48 (0.000908%)	
GCCCATGGGCATCGACACCTTGCGGCTAGGAACTGGAACGAGACGGGTGGCAAAGATTTCGAGTAGCACTTCATACTACCGTGGGTTTTTTAAACCTTCC	5 (0.000095%)	
GCCCGCGTCGACCTTTTATCTAATAAATGCGTCCCTTCCATAAGTCGGGGTTTGTTGCACGTATTAGCTCTAGAATTACTACGGTTATCCGAGTAGTAGT	4 (0.000076%)	
GCCCTATCAACTTTCGATGGTAGGATAGTGGCCTACCATGGTGGTAACGGGTGACGGAGAATTAGGGTTCGATTCCGGAGAGGGAGCCTGAGAAACGGCT	16 (0.000303%)	
GCCGAAGCGAGCCTTGGGACCAAAAACAGGGGTTGTACCCCGCCTCCGATTCACGGAGTAAGTAAAATAACGTTAAAAGTAGTGGTATTTCACTTGCGCC	39 (0.000738%)	
GCCGACTTCCCTTGCCTACATTGTTCCATCGACCAGAGGCTGTTCACCTTGGAGACCTGATGCGGTTATGAGTACGACCGGGCGTGAGCGGCACTCGGTC	257 (0.004862%)	
GCCGCCCGCGACGTCGCGAGAAGTCCACTAAACCTTATCATTTAGAGGAAGGAGAAGTCGTAACAAGGTTTCCGTAGGTGAACCTGCGGAAGGATCGATC	18 (0.000341%)	
GCCGCCGGGGGCGCACCGGACACCACGCGACGTGCGGTGCTCTTCCAGCCGCTGGACCCTACCTCCGGCTGAGCCGTTTCCAGGGTGGGCAGGCTGTTAA	10 (0.000189%)	
GCCGCGGTAATTCCAGCTCCAATAGCGTATATTTAAGTTGTTGCAGTTAAAAAGCTCGTAGTTGAACCTTGGGATGGGTCGGCCGGTCCGCCTTTGGTGT	8 (0.000151%)	
GCCGGGGGCGCACCGGACACCACGCGACGTGCGGTGCTCTTCCAGCCGCTGGACCCTACCTCCGGCTGAGCCGTTTCCAGGGTGGGCAGGCTGTTAAACA	25 (0.000473%)	
GCCTAACGGCGTGCCTCGGCATCAGCGTGCTCCGGGCGTCGGCCTGTGGGCTCCCCATTCGACCCGTCTTGAAACACGGACCAAGGAGTCTGACATGTGT	43 (0.000813%)	
GCCTAAGTAGTGTTTCCTTGTTAGAACATACAAAGCCAAAGACTCATACGGACTTTGGCTACACCATGAAAGCTTTGAGAAGCTAGAAGAAGGTTGGTTA	48 (0.000908%)	
GCCTAAGTAGTGTTTCCTTGTTAGAAGACACAAAGCCAAAGACTCATATGGACTTTGGCTACACCATGAAAGCTTTGAGAAGCAAGAAGAAGGTTGGTTA	40 (0.000757%)	
GCCTACATTGTTCCATCGACCAGAGGCTGTTCACCTTGGAGACCTGATGCGGTTATGAGTACGACCGGGCGTGAGCGGCACTCGGTCCTCCGGATTTTCA	8 (0.000151%)	
GCCTAGGCTGTCCCGAGTGTGAGCGAGGTGTGAGTGTCGCCCATGGGCATCGACACCTTGCGGCTAGGAACTGGAACGAGACGGGTGGCAAAGATTTCGA	359 (0.006792%)	
GCCTCGGCATCAGCGTGCTCCGGGCGTCGGCCTGTGGGCTCCCCATTCGACCCGTCTTGAAACACGGACCAAGGAGTCTGACATGTGTGCGAGTCAACGG	13 (0.000246%)	
GCCTCTGTGCTGGCGACGCATCATTCAAATTTCTGCCCTATCAACTTTCGATGGTAGGATAGTGGCCTACCATGGTGGTAACGGGTGACGGAGAATTAGG	12 (0.000227%)	
GCCTGCTAACTAGCTACGTGGAGGCATCCCTTCACGGCCGGCTTCTTAGAGGGACTATGGCCGTTTAGGCCAAGGAAGTTTGAGGCAATAACAGGTCTGT	4 (0.000076%)	
GCCTTACGGGTTTACTCACCCGTTGACTCGCACACATGTCAGACTCCTTGGTCCGTGTTTCAAGACGGGTCGAATGGGGAGCCCACAGGCCGACGCCCGG	22 (0.000416%)	
GCGACGCATCATTCAAATTTCTGCCCTATCAACTTTCGATGGTAGGATAGTGGCCTACCATGGTGGTAACGGGTGACGGAGAATTAGGGTTCGATTCCGG	37 (0.000700%)	
GCGACGGGCGGTGTGTACAAAGGGCAGGGACGTAGTCAACGCGAGCTGATGACTCGCGCTTACTAGGAATTCCTCGTTGAAGACCAACAATTGCAATGAT	41 (0.000776%)	
GCGACGTGGGTGGTTCGCCGCCCGCGACGTCGCGAGAAGTCCACTAAACCTTATCATTTAGAGGAAGGAGAAGTCGTAACAAGGTTTCCGTAGGTGAACC	388 (0.007340%)	
GCGAGACAAGGGTTCACATTTCGTTCATCACCCTTGGCCGGCTATCGAACAGCCGGACTCCCATCAAAAGATGGTTGCCAAGAACATCTTCGTTACGGTT	31 (0.000586%)	
GCGAGACAAGGGTTCACATTTCGTTCATCACCCTTGGCCGGCTTTCGAACAGCCGGACTCCCATCAAAAGATGGTTGCCAAGAACATCTTCGTTACGGTT	39 (0.000738%)	
GCGAGGTGTGAGTGTCGCCCATGGGCATCGACACCTTGCGGCTAGGAACTGGAACGAGACGGGTGGCAAAGATTTCGAGTAGCACTTCATACTACCGTGG	42 (0.000795%)	

GCGCACCGGACACCACGCGACGTGCGGTGCTCTTCCAGCCGCTGGACCCTACCTCCGGCTGAGCCGTTTCCAGGGTGGGCAGGCTGTTAAACAGAAAAGA	23 (0.000435%)	
GCGCATCGAACTACCTACCACCTATCCTTCTCAGTTAATTCACGGGCGATGTTACGCTCGATGATGAGTTCCGGGGCCTGTGTTTCGTACCTAATTTGAA	8 (0.000151%)	
GCGCCTAACGGCGTGCCTCGGCATCAGCGTGCTCCGGGCGTCGGCCTGTGGGCTCCCCATTCGACCCGTCTTGAAACACGGACCAAGGAGTCTGACATGT	19 (0.000359%)	
GCGCCTTACGGGTTTACTCACCCGTTGACTCGCACACATGTCAGACTCCTTGGTCCGTGTTTCAAGACGGGTCGAATGGGGAGCCCACAGGCCGACGCCC	14 (0.000265%)	
GCGCTTGTACGGCTTTGGCTCGGATTCGTCCGTCTTCTTTCTTCTTAGCCGAGTACTTCGGTAGATTAGTTGGAACGATTGATGATTTTGAGTTAATTGA	6 (0.000114%)	
GCGGAGGACATTGTCAGGTGGGGAGTTTGGCTGGGGCGGCACATCTGTTAAAAGATAACGCAGGTGTCCTAAGATGAGCTCAACGAGAACAGAAATCTCG	1497 (0.028321%)	
GCGGCACATCTGTTAAAAGATAACGCAGGTGTCCTAAGATGAGCTCAACGAGAACAGAAATCTCGTGTGGAACAAAAGGGTAAAAGCTCGTTTGATTCTG	18 (0.000341%)	
GCGGCACTCGGTCCTCCGGATTTTCAAGGGCCGCCGGGGGCGCACCGGACACCACGCGACGTGCGGTGCTCTTCCAGCCGCTGGACCCTACCTCCGGCTG	56 (0.001059%)	
GCGGCGACGTGGGTGGTTCGCCGCCCGCGACGTCGCGAGAAGTCCACTAAACCTTATCATTTAGAGGAAGGAGAAGTCGTAACAAGGTTTCCGTAGGTGA	1266 (0.023950%)	
GCGGTAATTCCAGCTCCAATAGCGTATATTTAAGTTGTTGCAGTTAAAAAGCTCGTAGTTGAACCTTGGGATGGGTCGGCCGGTCCGCCTTTGGTGTGCA	15 (0.000284%)	
GCGGTTGACGGCAACGTTAGGGAGTCCGGAGACGTCGGCGGGGGCCTCGGGAAGAGTTATCTTTTCTGTTTAACAGCCTGCCCACCCTGGAAACGGCTCA	7 (0.000132%)	
GCGGTTTAAGTTCTTATACTCAATCATACACATGACATCAAGTCATATTCGACTCCAAAACACTAACCAACCTTCTTCTTGCTTCTCAAAGCTTTCATGG	140 (0.002649%)	
GCGGTTTAAGTTGTTATACTCAATCATACACATGACAACAAGTCATATTCGACTCCAAAACACTAACCAACCTTCTTCTTGCTTCTCAAAGCTTTCATGG	52 (0.000984%)	
GCGTAAGAATTGTATCCTTGTTAGAAGACACAAAGCCAAA	190 (0.001438%)	
GCGTCGTGTAGGGAAAGAGTGTTCAGAGCCGTGTAGATCT	8 (0.000061%)	
GCGTGCCTCGGCATCAGCGTGCTCCGGGCGTCGGCCTGTGGGCTCCCCATTCGACCCGTCTTGAAACACGGACCAAGGAGTCTGACATGTGTGCGAGTCA	8 (0.000151%)	
GCGTGCTCCGGGCGTCGGCCTGTGGGCTCCCCATTCGACCCGTCTTGAAACACGGACCAAGGAGTCTGACATGTGTGCGAGTCAACGGGTGAGTAAACCC	3 (0.000057%)	
GCTAACTAGCTACGTGGAGGCATCCCTTCACGGCCGGCTTCTTAGAGGGACTATGGCCGTTTAGGCCAAGGAAGTTTGAGGCAATAACAGGTCTGTGATG	28 (0.000530%)	
GCTAATGTATCCAGAGCGTAGGCTTGCTTTGAGCACTCTAATTTCTTCAAAGTAACAGCGCCGGAGGCACGACCCGGCCAATTAAGACCAGGAGCGTATC	23 (0.000435%)	
GCTAGTGTTCGTAGGCTCCATGCTCGCGCATCGAACTACCTACCACCTATCCTTCTCAGTTAATTCACGGGCGATGTTACGCTCGATGATGAGTTCCGGG	23 (0.000435%)	
GCTCCAATAGCGTATATTTAAGTTGTTGCAGTTAAAAAGCTCGTAGTTGAACCTTGGGATGGGTCGGCCGGTCCGCCTTTGGTGTGCATTGGTCGGCTTG	12 (0.000227%)	
GCTCCATGCTCGCGCATCGAACTACCTACCACCTATCCTTCTCAGTTAATTCACGGGCGATGTTACGCTCGATGATGAGTTCCGGGGCCTGTGTTTCGTA	11 (0.000208%)	
GCTCCGGGCGTCGGCCTGTGGGCTCCCCATTCGACCCGTCTTGAAACACGGACCAAGGAGTCTGACATGTGTGCGAGTCAACGGGTGAGTAAACCCGTAA	9 (0.000170%)	
GCTCGCGCATCGAACTACCTACCACCTATCCTTCTCAGTTAATTCACGGGCGATGTTACGCTCGATGATGAGTTCCGGGGCCTGTGTTTCGTACCTAATT	6 (0.000114%)	
GCTCGTGTAAAGTTGGGAATTCGTTAAGGAGCTGTTGCTTTGTTAGTGTAGAAACACTTGTGTAGAATTGGGGATTGTTTTTTTTGGAGTGATTTAGGGG	5 (0.000095%)	
GCTGATGCGGGACGGAAGCTGGTCTCCCGTGTGTTACCGCACGCGGTTGGCCTAAATCCGAGCCAAGGACGCCTGGAGCGTACCGACATGCGGTGGTGAA	6 (0.000114%)	
GCTGGCGACGCATCATTCAAATTTCTGCCCTATCAACTTTCGATGGTAGGATAGTGGCCTACCATGGTGGTAACGGGTGACGGAGAATTAGGGTTCGATT	32 (0.000605%)	
GCTGGGGCGGCACATCTGTTAAAAGATAACGCAGGTGTCCTAAGATGAGCTCAACGAGAACAGAAATCTCGTGTGGAACAAAAGGGTAAAAGCTCGTTTG	8 (0.000151%)	
GCTGTCCCGAAGGTATCTCGCGCTTGTACGGCTTTGGCTCGGATTCGTCCGTCTTCTTTCTTCTTAGCCGAGTACTTCGGTAGATTAGTTGGAACGATTG	57 (0.001078%)	
GCTGTCCCGAGTGTGAGCGAGGTGTGAGTGTCGCCCATGGGCATCGACACCTTGCGGCTAGGAACTGGAACGAGACGGGTAGCAAAGATTTCGAGTAGCA	68 (0.001286%)	
GCTGTCCCGAGTGTGAGCGAGGTGTGAGTGTCGCCCATGGGCATCGACACCTTGCGGCTAGGAACTGGAACGAGACGGGTGGCAAAGATTTCGAGTAGCA	105 (0.001986%)	
GCTGTTGCTTTGTTAGTGTAGAAACACTTGTGTAGAATTGGGGATTGTTTTTTTTGGAGTGATTTAGGGGAGGGTCGAATCTTAGCGACAAAGGGCTGAA	3 (0.000057%)	
GCTTCCTTGCGCCTTACGGGTTTACTCACCCGTTGACTCGCACACATGTCAGACTCCTTGGTCCGTGTTTCAAGACGGGTCGAATGGGGAGCCCACAGGC	42 (0.000795%)	
GCTTGTACGGCTTTGGCTCGGATTCGTCCGTCTTCTTTCTTCTTAGCCGAGTACTTCGGTAGATTAGTTGGAACGATTGATGATTTTGAGTTAATTGAAC	21 (0.000397%)	
GCTTGTCTCAAAGATTAAGCCATGCATGTGTAAGTATGAACGAATTCAGACTGTGAAACTGCGAATGGCTCATTAAATCAGTTATAGTTTGTTTGATGGT	13 (0.000246%)	
GCTTTCACGGTTCGTATTCGTACTGAAAATCAGAATCAAACGAGCTTTTACCCTTTTGTTCCACACGAGATTTCTGTTCTCGTTGAGCTCATCTTAGGAC	16 (0.000303%)	
GCTTTGGCTCGGATTCGTCCGTCTTCTTTCTTCTTAGCCGAGTACTTCGGTAGATTAGTTGGAACGATTGATGATTTTGAGTTAATTGAACGTTCGGCGT	5 (0.000095%)	
GCTTTGTTTTAATTAAACAGTCGGATTCCCCTTGTCCGTACCAGTTCTGAGCTGACTGTTCGACGCCCGGGGAAAGCTCCCGAGAGAGCCGTTCCCAGTC	13 (0.000246%)	
GGAAAGAGTGTTCAGAGCCGTGTAGATCTCGGTGGTCGCC	15 (0.000114%)	
GGAACCGGGACGTGGCGGTTGACGGCAACGTTAGGGAGTCCGGAGACGTCGGCGGGGGCCTCGGGAAGAGTTATCTTTTCTGTTTAACAGCCTGCCCACC	51 (0.000965%)	
GGAAGAGCGTCGTGTAGGGAAAGAGTGTTCAGAGCCGTGT	48 (0.000363%)	
GGAAGTCGAAATCCGCTAAGGAGTGTGTAACAACTCACCTGCCGAATCAACTAGCCCCGAAAATGGATGGCGCTTAAGCGCGCGACCTATACCCGGCCGT	30 (0.000568%)	
GGACAGTCGGGGGCATTCGTATTTCATAGTCAGAGGTGAAATTCTTGGATTTATGAAAGACGAACAACTGCGAAAGCATTTGCCAAGGATGTTTTCATTA	64 (0.001211%)	

GGACATTGTCAGGTGGGGAGTTTGGCTGGGGCGGCACATCTGTTAAAAGATAACGCAGGTGTCCTAAGATG AGCTCAACGAGAACAGAAATCTCGTGTGG	179 (0.003386%)
GGACGGTGGTCATGGAAGTCGAAATCCGCTAAGGAGTGTGTAACAACTCACCTGCCGAATCAACTAGCCCC GAAAATGGATGGCGCTTAAGCGCGCGACC	47 (0.000889%)
GGACGTGGCGGTTGACGGCAACGTTAGGGAGTCCGGAGACGTCGGCGGGGGCCTCGGGAAGAGTTATCTTT TCTGTTTAACAGCCTGCCCACCCTGGAAA	3 (0.000057%)
GGAGACGTCGGCGGGGGCCTCGGGAAGAGTTATCTTTTCTGTTTAACAGCCTGCCCACCCTGGAAACGGCT CAGCCGGAGGTAGGGTCCAGCGGCTGGAA	21 (0.000397%)
GGAGCGACGGGCGGTGTGTACAAAGGGCAGGGACGTAGTCAACGCGAGCTGATGACTCGCGCTTACTAGGA ATTCCTCGTTGAAGACCAACAATTGCAAT	33 (0.000624%)
GGAGCTGTTGCTTTGTTAGTGTAGAAACACTTGTGTAGAATTGGGGATTGTTTTTTTTGGAGTGATTTAGG GGAGGGTCGAATCTTAGCGACAAAGGGCT	16 (0.000303%)
GGAGGACATTGTCAGGTGGGGAGTTTGGCTGGGGCGGCACATCTGTTAAAAGATAACGCAGGTGTCCTAAG ATGAGCTCAACGAGAACAGAAATCTCGTG	373 (0.007057%)
GGAGGGCAAGTCTGGTGCCAGCAGCCGCGGTAATTCCAGCTCCAATAGCGTATATTTAAGTTGTTGCAGTT AAAAAGCTCGTAGTTGAACCTTGGGATGG	74 (0.001400%)
GGAGTAATGATTAACAGGGACAGTCGGGGGCATTCGTATTTCATAGTCAGAGGTGAAATTCTTGGATTTAT GAAAGACGAACAACTGCGAAAGCATTTGC	1017 (0.019240%)
GGAGTCCGGAGACGTCGGCGGGGGCCTCGGGAAGAGTTATCTTTTCTGTTTAACAGCCTGCCCACCCTGGA AACGGCTCAGCCGGAGGTAGGGTCCAGCG	4 (0.000076%)
GGAGTTTGGCTGGGGCGGCACATCTGTTAAAAGATAACGCAGGTGTCCTAAGATGAGCTCAACGAGAACAG AAATCTCGTGTGGAACAAAAGGGTAAAAG	6 (0.000114%)
GGAGTTTTTTCAGCAGTTCTCGGACAAAAATTGCTGAGTGGCCGAGAAGAATGGGCGTGTCATGCGTGGGC TGACATGGATTCTTCGAGGCCTAGGGGTG	4 (0.000076%)
GGATAGCTAGTGTTCGTAGGCTCCATGCTCGCGCATCGAACTACCTACCACCTATCCTTCTCAGTTAATTC ACGGGCGATGTTACGCTCGATGATGAGTT	33 (0.000624%)
GGATTTTCAAGGGCCGCCGGGGGCGCACCGGACACCACGCGACGTGCGGTGCTCTTCCAGCCGCTGGACCC TACCTCCGGCTGAGCCGTTTCCAGGGTGG	48 (0.000908%)
GGCAACGTTAGGGAGTCCGGAGACGTCGGCGGGGGCCTCGGGAAGAGTTATCTTTTCTGTTTAACAGCCTG CCCACCCTGGAAACGGCTCAGCCGGAGGT	9 (0.000170%)
GGCAAGTCTGGTGCCAGCAGCCGCGGTAATTCCAGCTCCAATAGCGTATATTTAAGTTGTTGCAGTTAAAA AGCTCGTAGTTGAACCTTGGGATGGGTCG	69 (0.001305%)
GGCAATTCCCCGCCACATCCTCTCAAACGCAATGGAAAGAGAGAAAGGACGAGGTCTTGACCGTCATCTTT TGCCCGAAGGACGGATGAGCTTTGGCGGG	71 (0.001343%)
GGCACATCTGTTAAAAGATAACGCAGGTGTCCTAAGATGAGCTCAACGAGAACAGAAATCTCGTGTGGAAC AAAAGGGTAAAAGCTCGTTTGATTCTGAT	14 (0.000265%)
GGCACTCGGTCCTCCGGATTTTCAAGGGCCGCCGGGGGCGCACCGGACACCACGCGACGTGCGGTGCTCTT CCAGCCGCTGGACCCTACCTCCGGCTGAG	14 (0.000265%)
GGCAGAGCCCGCGTCGACCTTTTATCTAATAAATGCGTCCCTTCCATAAGTCGGGGTTTGTTGCACGTATT AGCTCTAGAATTACTACGGTTATCCGAGT	28 (0.000530%)
GGCATCAGCGTGCTCCGGGCGTCGGCCTGTGGGCTCCCCATTCGACCCGTCTTGAAACACGGACCAAGGAG TCTGACATGTGTGCGAGTCAACGGGTGAG	5 (0.000095%)
GGCATCGACACCTTGCGGCTAGGAACTGGAACGAGACGGGTGGCAAAGATTTCGAGTAGCACTTCATACTA CCGTGGGTTTTTTAAACCTTCCGAGTTTT	15 (0.000284%)
GGCATTCGTATTTCATAGTCAGAGGTGAAATTCTTGGATTTATGAAAGACGAACAACTGCGAAAGCATTTG CCAAGGATGTTTTCATTAATCAAGAACGA	9 (0.000170%)
GGCCGCCGGGGGCGCACCGGACACCACGCGACGTGCGGTGCTCTTCCAGCCGCTGGACCCTACCTCCGGCT GAGCCGTTTCCAGGGTGGGCAGGCTGTTA	8 (0.000151%)
GGCCTCTGTGCTGGCGACGCATCATTCAAATTTCTGCCCTATCAACTTTCGATGGTAGGATAGTGGCCTAC CATGGTGGTAACGGGTGACGGAGAATTAG	157 (0.002970%)
GGCCTGTGGGCTCCCCATTCGACCCGTCTTGAAACACGGACCAAGGAGTCTGACATGTGTGCGAGTCAACG GGTGAGTAAACCCGTAAGGCGCAAGGAAG	5 (0.000095%)
GGCGACGCATCATTCAAATTTCTGCCCTATCAACTTTCGATGGTAGGATAGTGGCCTACCATGGTGGTAAC GGGTGACGGAGAATTAGGGTTCGATTCCG	21 (0.000397%)
GGCGACGTGGGTGGTTCGCCGCCCGCGACGTCGCGAGAAGTCCACTAAACCTTATCATTTAGAGGAAGGAG AAGTCGTAACAAGGTTTCCGTAGGTGAAC	378 (0.007151%)
GGCGAGACAAGGGTTCACATTTCGTTCATCACCCTTGGCCGGCTATCGAACAGCCGGACTCCCATCAAAAG ATGGTTGCCAAGAACATCTTCGTTACGGT	14 (0.000265%)
GGCGAGACAAGGGTTCACATTTCGTTCATCACCCTTGGCCGGCTTTCGAACAGCCGGACTCCCATCAAAAG ATGGTTGCCAAGAACATCTTCGTTACGGT	17 (0.000322%)
GGCGCACCGGACACCACGCGACGTGCGGTGCTCTTCCAGCCGCTGGACCCTACCTCCGGCTGAGCCGTTTC CAGGGTGGGCAGGCTGTTAAACAGAAAAG	30 (0.000568%)
GGCGGCACATCTGTTAAAAGATAACGCAGGTGTCCTAAGATGAGCTCAACGAGAACAGAAATCTCGTGTGG AACAAAAGGGTAAAAGCTCGTTTGATTCT	3 (0.000057%)
GGCGGTTGACGGCAACGTTAGGGAGTCCGGAGACGTCGGCGGGGGCCTCGGGAAGAGTTATCTTTTCTGTT TAACAGCCTGCCCACCCTGGAAACGGCTC	18 (0.000341%)
GGCGTAAGAATTGTATCCTTGTTAGAAGACACAAAGCCAA	62 (0.000469%)
GGCGTGCCTCGGCATCAGCGTGCTCCGGGCGTCGGCCTGTGGGCTCCCCATTCGACCCGTCTTGAAACACG GACCAAGGAGTCTGACATGTGTGCGAGTC	19 (0.000359%)
GGCTGGGGCGGCACATCTGTTAAAAGATAACGCAGGTGTCCTAAGATGAGCTCAACGAGAACAGAAATCTC GTGTGGAACAAAAGGGTAAAAGCTCGTTT	6 (0.000114%)
GGCTGTCCCGAGTGTGAGCGAGGTGTGAGTGTCGCCCATGGGCATCGACACCTTGCGGCTAGGAACTGGAA CGAGACGGGTAGCAAAGATTTCGAGTAGC	9 (0.000170%)
GGCTGTCCCGAGTGTGAGCGAGGTGTGAGTGTCGCCCATGGGCATCGACACCTTGCGGCTAGGAACTGGAA CGAGACGGGTGGCAAAGATTTCGAGTAGC	22 (0.000416%)
GGCTTTGGCTCGGATTCGTCCGTCTTCTTTCTTCTTAGCCGAGTACTTCGGTAGATTAGTTGGAACGATTG ATGATTTTGAGTTAATTGAACGTTCGGCG	8 (0.000151%)
GGGAAAGAGTGTTCAGAGCCGTGTAGATCTCGGTGGTCGC	16 (0.000121%)
GGGACAGTCGGGGGCATTCGTATTTCATAGTCAGAGGTGAAATTCTTGGATTTATGAAAGACGAACAACTG CGAAAGCATTTGCCAAGGATGTTTTCATT	26 (0.000492%)
GGGACGTGGCGGTTGACGGCAACGTTAGGGAGTCCGGAGACGTCGGCGGGGGCCTCGGGAAGAGTTATCTT TTCTGTTTAACAGCCTGCCCACCCTGGAA	21 (0.000397%)
GGGAGTCCGGAGACGTCGGCGGGGGCCTCGGGAAGAGTTATCTTTTCTGTTTAACAGCCTGCCCACCCTGG AAACGGCTCAGCCGGAGGTAGGGTCCAGC	3 (0.000057%)

GGGAGTTTGGCTGGGGCGGCACATCTGTTAAAAGATAACGCAGGTGTCCTAAGATGAGCTCAACGAGAACA<br>GAAATCTCGTGTGGAACAAAAGGGTAAAA	38 (0.000719%)
GGGCAAGTCTGGTGCCAGCAGCCGCGGTAATTCCAGCTCCAATAGCGTATATTTAAGTTGTTGCAGTTAAA<br>AAGCTCGTAGTTGAACCTTGGGATGGGTC	144 (0.002724%)
GGGCAGAGCCCGCGTCGACCTTTTATCTAATAAATGCGTCCCTTCCATAAGTCGGGGTTTGTTGCACGTAT<br>TAGCTCTAGAATTACTACGGTTATCCGAG	55 (0.001041%)
GGGCATCGACACCTTGCGGCTAGGAACTGGAACGAGACGGGTGGCAAAGATTTCGAGTAGCACTTCATACT<br>ACCGTGGGTTTTTTAAACCTTCCGAGTTT	13 (0.000246%)
GGGCATTCGTATTTCATAGTCAGAGGTGAAATTCTTGGATTTATGAAAGACGAACAACTGCGAAAGCATTT<br>GCCAAGGATGTTTTCATTAATCAAGAACG	32 (0.000605%)
GGGCCGCCGGGGGCGCACCGGACACCACGCGACGTGCGGTGCTCTTCCAGCCGCTGGACCCTACCTCCGGC<br>TGAGCCGTTTCCAGGGTGGGCAGGCTGTT	3 (0.000057%)
GGGCGCACCGGACACCACGCGACGTGCGGTGCTCTTCCAGCCGCTGGACCCTACCTCCGGCTGAGCCGTTT<br>CCAGGGTGGGCAGGCTGTTAAACAGAAAA	6 (0.000114%)
GGGCGGCACATCTGTTAAAAGATAACGCAGGTGTCCTAAGATGAGCTCAACGAGAACAGAAATCTCGTGTG<br>GAACAAAAGGGTAAAAGCTCGTTTGATTC	4 (0.000076%)
GGGCGGTGTGTACAAAGGGCAGGGACGTAGTCAACGCGAGCTGATGACTCGCGCTTACTAGGAATTCCTCG<br>TTGAAGACCAACAATTGCAATGATCGATC	20 (0.000378%)
GGGCGTCGGCCTGTGGGCTCCCCATTCGACCCGTCTTGAAACACGGACCAAGGAGTCTGACATGTGTGCGA<br>GTCAACGGGTGAGTAAACCCGTAAGGCGC	8 (0.000151%)
GGGGAGTTTGGCTGGGGCGGCACATCTGTTAAAAGATAACGCAGGTGTCCTAAGATGAGCTCAACGAGAAC<br>AGAAATCTCGTGTGGAACAAAAGGGTAAA	32 (0.000605%)
GGGGCATTCGTATTTCATAGTCAGAGGTGAAATTCTTGGATTTATGAAAGACGAACAACTGCGAAAGCATT<br>TGCCAAGGATGTTTTCATTAATCAAGAAC	27 (0.000511%)
GGGGCGCACCGGACACCACGCGACGTGCGGTGCTCTTCCAGCCGCTGGACCCTACCTCCGGCTGAGCCGTT<br>TCCAGGGTGGGCAGGCTGTTAAACAGAAA	10 (0.000189%)
GGGGCGGCACATCTGTTAAAAGATAACGCAGGTGTCCTAAGATGAGCTCAACGAGAACAGAAATCTCGTGT<br>GGAACAAAAGGGTAAAAGCTCGTTTGATT	7 (0.000132%)
GGGGGCATTCGTATTTCATAGTCAGAGGTGAAATTCTTGGATTTATGAAAGACGAACAACTGCGAAAGCAT<br>TTGCCAAGGATGTTTTCATTAATCAAGAA	17 (0.000322%)
GGGGGCGCACCGGACACCACGCGACGTGCGGTGCTCTTCCAGCCGCTGGACCCTACCTCCGGCTGAGCCGT<br>TTCCAGGGTGGGCAGGCTGTTAAACAGAA	23 (0.000435%)
GGGGTTGAAATCGTCGACCAGGTCCGAGACTTCATCGACCGGGTCCGAGGATTCGTCGACCAGGACGGCCG<br>GATGTCCGAGAAAAAAAAATGTTGCCGAA	5 (0.000095%)
GGGTGGTTCGCCGCCCGCGACGTCGCGAGAAGTCCACTAAACCTTATCATTTAGAGGAAGGAGAAGTCGTA<br>ACAAGGTTTCCGTAGGTGAACCTGCGGAA	138 (0.002611%)
GGGTGTCACAAATCGTCGTCCCTCACCATCCTTTGCTGATGCGGGACGGAAGCTGGTCTCCCGTGTGTTAC<br>CGCACGCGGTTGGCCTAAATCCGAGCCAA	7 (0.000132%)
GGGTGTTGGTCGATTAAGACAGCAGGACGGTGGTCATGGAAGTCGAAATCCGCTAAGGAGTGTGTAACAAC<br>TCACCTGCCGAATCAACTAGCCCCGAAAA	24 (0.000454%)
GGGTTCACATTTCGTTCATCACCCTTGGCCGGCTATCGAACAGCCGGACTCCCATCAAAAGATGGTTGCCA<br>AGAACATCTTCGTTACGGTTTGCTAATTC	3 (0.000057%)
GGGTTCACATTTCGTTCATCACCCTTGGCCGGCTTTCGAACAGCCGGACTCCCATCAAAAGATGGTTGCCA<br>AGAACATCTTCGTTACGGTTTGCTAATTC	3 (0.000057%)
GGGTTGAAATCGTCGACCAGGTCCGAGACTTCATCGACCGGGTCCGAGGATTCGTCGACCAGGACGGCCGG<br>ATGTCCGAGAAAAAAAAATGTTGCCGAAT	3 (0.000057%)
GGGTTGCGGTTTAAGTTGTTATACTCAATCATACACATGACAACAAGTCATATTCGACTCCAAAACACTAA<br>CCAACCTTCTTCTTGCTTCTCAAAGCTTT	474 (0.008967%)
GGGTTTACTCACCCGTTGACTCGCACACATGTCAGACTCCTTGGTCCGTGTTTCAAGACGGGTCGAATGGG<br>GAGCCCACAGGCCGACGCCCGGAGCACGC	7 (0.000132%)
GGTAATTCCAGCTCCAATAGCGTATATTTAAGTTGTTGCAGTTAAAAAGCTCGTAGTTGAACCTTGGGATG<br>GGTCGGCCGGTCCGCCTTTGGTGTGCATT	108 (0.002043%)
GGTACGAAACACAGGCCCCGGAACTCATCATCGAGCGTAACATCGCCCGTGAATTAACTGAGAAGGATAGG<br>TGGTAGGTAGTTCGATGCGCGAGCATGGA	3 (0.000057%)
GGTAGGAGCGACGGGCGGTGTGTACAAAGGGCAGGGACGTAGTCAACGCGAGCTGATGACTCGCGCTTACT<br>AGGAATTCCTCGTTGAAGACCAACAATTG	41 (0.000776%)
GGTATCTCGCGCTTGTACGGCTTTGGCTCGGATTCGTCCGTCTTCTTTCTTCTTAGCCGAGTACTTCGGTA<br>GATTAGTTGGAACGATTGATGATTTTGAG	20 (0.000378%)
GGTCCTCCGGATTTTCAAGGGCCGCCGGGGGCGCACCGGACACCACGCGACGTGCGGTGCTCTTCCAGCCG<br>CTGGACCCTACCTCCGGCTGAGCCGTTTC	6 (0.000114%)
GGTCGACCAGGGGTTGAAATCGTCGACCAGGTCCGAGACTTCATCGACCGGGTCCGAGGATTCGTCGACCA<br>GGACGGCCGGATGTCCGAGAAAAAAAAAT	774 (0.014643%)
GGTCGATTAAGACAGCAGGACGGTGGTCATGGAAGTCGAAATCCGCTAAGGAGTGTGTAACAACTCACCTG<br>CCGAATCAACTAGCCCCGAAAATGGATGG	43 (0.000813%)
GGTCTTCAACGAGGAATTCCTAGTAAGCGCGAGTCATCAGCTCGCGTTGACTACGTCCCTGCCCTTTGTAC<br>ACACCGCCCGTCGCTCCTACCGATTGAAT	9 (0.000170%)
GGTGATATGAACACAAACGTTCAATATGACAAACCCATGCCAAGTAAAGAGAAAATGAAAACTGGTGATTG<br>TTGCGGAAATCGTCCAGGATTCCTCGACC	3 (0.000057%)
GGTGCCAGCAGCCGCGGTAATTCCAGCTCCAATAGCGTATATTTAAGTTGTTGCAGTTAAAAAGCTCGTAG<br>TTGAACCTTGGGATGGGTCGGCCGGTCCG	9 (0.000170%)
GGTGGGGAGTTTGGCTGGGGCGGCACATCTGTTAAAAGATAACGCAGGTGTCCTAAGATGAGCTCAACGAG<br>AACAGAAATCTCGTGTGGAACAAAAGGGT	3 (0.000057%)
GGTGGTCATGGAAGTCGAAATCCGCTAAGGAGTGTGTAACAACTCACCTGCCGAATCAACTAGCCCCGAAA<br>ATGGATGGCGCTTAAGCGCGACCTATA	9 (0.000170%)
GGTGGTTCGCCGCCCGCGACGTCGCGAGAAGTCCACTAAACCTTATCATTTAGAGGAAGGAGAAGTCGTAA<br>CAAGGTTTCCGTAGGTGAACCTGCGGAAG	14 (0.000265%)
GGTGTCACAAATCGTCGTCCCTCACCATCCTTTGCTGATGCGGGACGGAAGCTGGTCTCCCGTGTGTTACC<br>GCACGCGGTTGGCCTAAATCCGAGCCAAG	6 (0.000114%)
GGTGTGAGTGTCGCCCATGGGCATCGACACCTTGCGGCTAGGAACTGGAACGAGACGGGTAGCAAAGATTT<br>CGAGTAGCACTTCATACTACCGTGGGTTT	3 (0.000057%)
GGTGTGAGTGTCGCCCATGGGCATCGACACCTTGCGGCTAGGAACTGGAACGAGACGGGTGGCAAAGATTT<br>CGAGTAGCACTTCATACTACCGTGGGTTT	3 (0.000057%)
GGTGTTGGTCGATTAAGACAGCAGGACGGTGGTCATGGAAGTCGAAATCCGCTAAGGAGTGTGTAACAACT<br>CACCTGCCGAATCAACTAGCCCCGAAAAT	4 (0.000076%)
GGTTCACATTTCGTTCATCACCCTTGGCCGGCTATCGAACAGCCGGACTCCCATCAAAAGATGGTTGCCAA<br>GAACATCTTCGTTACGGTTTGCTAATTCT	4 (0.000076%)

GGTTCACATTTCGTTCATCACCCTTGGCCGGCTTTCGAACAGCCGGACTCCCATCAAAAGATGGTTGCCAAGAACATCTTCGTTACGGTTTGCTAATTCT	3 (0.000057%)	
GGTTCGCCGCCCGCGACGTCGCGAGAAGTCCACTAAACCTTATCATTTAGAGGAAGGAGAAGTCGTAACAAGGTTTCCGTAGGTGAACCTGCGGAAGGAT	15 (0.000284%)	
GGTTCGTATTCGTACTGAAAATCAGAATCAAACGAGCTTTTACCCTTTTGTTCCACACGAGATTTCTGTTCTCGTTGAGCTCATCTTAGGACACCTGCGT	12 (0.000227%)	
GGTTGAAATCGTCGACCAGGTCCGAGACTTCATCGACCGGGTCCGAGGATTCGTCGACCAGGACGGCCGGATGTCCGAGAAAAAAAAATGTTGCCGAATA	14 (0.000265%)	
GGTTGACGGCAACGTTAGGGAGTCCGGAGACGTCGGCGGGGGCCTCGGGAAGAGTTATCTTTTCTGTTTAACAGCCTGCCCACCCTGGAAACGGCTCAGC	14 (0.000265%)	
GGTTGCGGTTTAAGTTCTTATACTCAATCATACACATGACATCAAGTCATATTCGACTCCAAAACACTAACCAACCTTCTTCTTGCTTCTCAAAGCTTTC	1252 (0.023686%)	
GGTTGCGGTTTAAGTTGTTATACTCAATCATACACATGACAACAAGTCATATTCGACTCCAAAACACTAACCAACCTTCTTCTTGCTTCTCAAAGCTTTC	32 (0.000605%)	
GGTTTAAGTTCTTATACTCAATCATACACATGACATCAAGTCATATTCGACTCCAAAACACTAACCAACCTTCTTCTTGCTTCTCAAAGCTTTCATGGTG	241 (0.004559%)	
GGTTTAAGTTGTTATACTCAATCATACACATGACAACAAGTCATATTCGACTCCAAAACACTAACCAACCTTCTTCTTGCTTCTCAAAGCTTTCATGGTG	99 (0.001873%)	
GGTTTACTCACCCGTTGACTCGCACACATGTCAGACTCCTTGGTCCGTGTTTCAAGACGGGTCGAATGGGGAGCCCACAGGCCGACGCCCGGAGCACGCT	26 (0.000492%)	
GTAAAGTTGGGAATTCGTTAAGGAGCTGTTGCTTTGTTAGTGTAGAAACACTTGTGTAGAATTGGGGATTGTTTTTTTTGGAGTGATTTAGGGGAGGGTC	57 (0.001078%)	
GTAAGAATTGTATCCTTGTTAGAAGACACAAAGCCAAAGA	342 (0.002588%)	
GTAATTCCAGCTCCAATAGCGTATATTTAAGTTGTTGCAGTTAAAAAGCTCGTAGTTGAACCTTGGGATGGGTCGGCCGGTCCGCCTTTGGTGTGCATTG	12 (0.000227%)	
GTACGAAACACAGGCCCCGGAACTCATCATCGAGCGTAACATCGCCCGTGAATTAACTGAGAAGGATAGGTGGTAGGTAGTTCGATGCGCGAGCATGGAG	20 (0.000378%)	
GTACGGCTTTGGCTCGGATTCGTCCGTCTTCTTTCTTCTTAGCCGAGTACTTCGGTAGATTAGTTGGAACGATTGATGATTTTGAGTTAATTGAACGTTC	7 (0.000132%)	
GTAGGAGCGACGGGCGGTGTGTACAAAGGGCAGGGACGTAGTCAACGCGAGCTGATGACTCGCGCTTACTAGGAATTCCTCGTTGAAGACCAACAATTGC	90 (0.001703%)	
GTAGGCTCCATGCTCGCGCATCGAACTACCTACCACCTATCCTTCTCAGTTAATTCACGGGCGATGTTACGCTCGATGATGAGTTCCGGGGCCTGTGTTT	19 (0.000359%)	
GTAGGGAAAGAGTGTTCAGAGCCGTGTAGATCTCGGTGGT	39 (0.000295%)	
GTAGTCATATGCTTGTCTCAAAGATTAAGCCATGCATGTGTAAGTATGAACGAATTCAGACTGTGAAACTGCGAATGGCTCATTAAATCAGTTATAGTTT	83 (0.001570%)	
GTATAAGAACTTAAACCGCAACCGCATCTTATAAGCCTAAGTAGTGTTTCCTTGTTAGAAGACACAAAGCCAAAGACTCATATGGACTTTGGCTACACCA	10 (0.000189%)	
GTATATTTAAGTTGTTGCAGTTAAAAAGCTCGTAGTTGAACCTTGGGATGGGTCGGCCGGTCCGCCTTTGGTGTGCATTGGTCGGCTTGTCCCTTCGGTC	15 (0.000284%)	
GTATCCAGAGCGTAGGCTTGCTTTGAGCACTCTAATTTCTTCAAAGTAACAGCGCCGGAGGCACGACCCGGCCAATTAAGACCAGGAGCGTATCGCCGAC	17 (0.000322%)	
GTATCCTTGTTAGAAGACACAAAGCCAAAGACTCATATGGACTTTGGCTACACCATGAAAGCTTTGAGAAGCAAGAAGAAGGTTGGTTAGTGTTTTGGAG	16 (0.000303%)	
GTATGATTGAGTATAAGAACTTAAACCGCAACCGCATCTTATAAGCCTAAGTAGTGTTTCCTTGTTAGAAGACACAAAGCCAAAGACTCATATGGACTTT	51 (0.000965%)	
GTATTTCATAGTCAGAGGTGAAATTCTTGGATTTATGAAAGACGAACAACTGCGAAAGCATTTGCCAAGGATGTTTTCATTAATCAAGAACGAAAGTTGG	4 (0.000076%)	
GTCACAAATCGTCGTCCCTCACCATCCTTTGCTGATGCGGGACGGAAGCTGGTCTCCCGTGTGTTACCGCACGCGGTTGGCCTAAATCCGAGCCAAGGAC	5 (0.000095%)	
GTCAGGTGGGGAGTTTGGCTGGGGCGGCACATCTGTTAAAAGATAACGCAGGTGTCCTAAGATGAGCTCAACGAGAACAGAAATCTCGTGTGGAACAAAA	15 (0.000284%)	
GTCATATGCTTGTCTCAAAGATTAAGCCATGCATGTGTAAGTATGAACGAATTCAGACTGTGAAACTGCGAATGGCTCATTAAATCAGTTATAGTTTGTT	43 (0.000813%)	
GTCATATTCGACTCCAAAACACTAACCAACCTTCTTCTTGCTTCTCAAAGCTTTCATGGTGTAGCCAAAGTCCATATGAGTCTTTGGCTTTGTGTCTTCT	202 (0.003821%)	
GTCCCGAAGGTATCTCGCGCTTGTACGGCTTTGGCTCGGATTCGTCCGTCTTCTTTCTTCTTAGCCGAGTACTTCGGTAGATTAGTTGGAACGATTGATG	3 (0.000057%)	
GTCCCGAGTGTGAGCGAGGTGTGAGTGTCGCCCATGGGCATCGACACCTTGCGGCTAGGAACTGGAACGAGACGGGTAGCAAAGATTTCGAGTAGCACTT	20 (0.000378%)	
GTCCCGAGTGTGAGCGAGGTGTGAGTGTCGCCCATGGGCATCGACACCTTGCGGCTAGGAACTGGAACGAGACGGGTGGCAAAGATTTCGAGTAGCACTT	19 (0.000359%)	
GTCCCTCACCATCCTTTGCTGATGCGGGACGGAAGCTGGTCTCCCGTGTGTTACCGCACGCGGTTGGCCTAAATCCGAGCCAAGGACGCCTGGAGCGTAC	18 (0.000341%)	
GTCCGGAGACGTCGGCGGGGGCCTCGGGAAGAGTTATCTTTTCTGTTTAACAGCCTGCCCACCCTGGAAACGGCTCAGCCGGAGGTAGGGTCCAGCGGCT	9 (0.000170%)	
GTCCTCCGGATTTTCAAGGGCCGCCGGGGGCGCACCGGACACCACGCGACGTGCGGTGCTCTTCCAGCCGCTGGACCCTACCTCCGGCTGAGCCGTTTCC	5 (0.000095%)	
GTCGAATATGACTTGATGTCATGTGTATGATTGAGTATAA	486 (0.003678%)	
GTCGACCAGGGGTTGAAATCGTCGACCAGGTCCGAGACTTCATCGACCGGGTCCGAGGATTCGTCGACCAGGACGGCCGGATGTCCGAGAAAAAAAAATG	53 (0.001003%)	
GTCGAGTTATCATGAATCATCAGAGCAACGGGCAGAGCCCGCGTCGACCTTTTATCTAATAAATGCGTCCCTTCCATAAGTCGGGGTTTGTTGCACGTAT	423 (0.008002%)	
GTCGATTAAGACAGCAGGACGGTGGTCATGGAAGTCGAAATCCGCTAAGGAGTGTGTAACAACTCACCTGCCGAATCAACTAGCCCCGAAAATGGATGGC	29 (0.000549%)	
GTCGCCCATGGGCATCGACACCTTGCGGCTAGGAACTGGAACGAGACGGGTGGCAAAGATTTCGAGTAGCACTTCATACTACCGTGGGTTTTTTAAACCT	23 (0.000435%)	
GTCGGAGTTTTTTCAGCAGTTCTCGGACAAAAATTGCTGAGTGGCCGAGAAGAATGGGCGTGTCATGCGTGGGCTGACATGGATTCTTCGAGGCCTAGGG	3 (0.000057%)	
GTCGGGGGCATTCGTATTTCATAGTCAGAGGTGAAATTCTTGGATTTATGAAAGACGAACAACTGCGAAAGCATTTGCCAAGGATGTTTTCATTAATCAA	6 (0.000114%)	
GTCGTCCCTCACCATCCTTTGCTGATGCGGGACGGAAGCTGGTCTCCCGTGTGTTACCGCACGCGGTTGGCCTAAATCCGAGCCAAGGACGCCTGGAGCG	32 (0.000605%)	

GTCGTGTAGGGAAAGAGTGTTCAGAGCCGTGTAGATCTCG	16 (0.000121%)	
GTCTGGTGCCAGCAGCCGCGGTAATTCCAGCTCCAATAGCGTATATTTAAGTTGTTGCAGTTAAAAAGCTCGTAGTTGAACCTTGGGATGGGTCGGCCGG	23 (0.000435%)	
GTCTTCAACGAGGAATTCCTAGTAAGCGCGAGTCATCAGCTCGCGTTGACTACGTCCCTGCCCTTTGTACACACCGCCCGTCGCTCCTACCGATTGAATG	16 (0.000303%)	
GTGAGCGAGGTGTGAGTGTCGCCCATGGGCATCGACACCTTGCGGCTAGGAACTGGAACGAGACGGGTGGCAAAGATTTCGAGTAGCACTTCATACTACC	30 (0.000568%)	
GTGAGCGGCACTCGGTCCTCCGGATTTTCAAGGGCCGCCGGGGGCGCACCGGACACCACGCGACGTGCGGTGCTCTTCCAGCCGCTGGACCCTACCTCCG	363 (0.006867%)	
GTGAGTGTCGCCCATGGGCATCGACACCTTGCGGCTAGGAACTGGAACGAGACGGGTGGCAAAGATTTCGAGTAGCACTTCATACTACCGTGGGTTTTTT	46 (0.000870%)	
GTGATATGAACACAAACGTTCAATATGACAAACCCATGCCAAGTAAAGAGAAAATGAAAACTGGTGATTGTTGCGGAAATCGTCCAGGATTCCTCGACCA	3 (0.000057%)	
GTGCCAGCAGCCGCGGTAATTCCAGCTCCAATAGCGTATATTTAAGTTGTTGCAGTTAAAAAGCTCGTAGTTGAACCTTGGGATGGGTCGGCCGGTCCGC	26 (0.000492%)	
GTGCCTCGGCATCAGCGTGCTCCGGGCGTCGGCCTGTGGGCTCCCCATTCGACCCGTCTTGAAACACGGACCAAGGAGTCTGACATGTGTGCGAGTCAAC	8 (0.000151%)	
GTGCTCCGGGCGTCGGCCTGTGGGCTCCCCATTCGACCCGTCTTGAAACACGGACCAAGGAGTCTGACATGTGTGCGAGTCAACGGGTGAGTAAACCCGT	13 (0.000246%)	
GTGCTGGCGACGCATCATTCAAATTTCTGCCCTATCAACTTTCGATGGTAGGATAGTGGCCTACCATGGTGGTAACGGGTGACGGAGAATTAGGGTTCGA	40 (0.000757%)	
GTGGCGGTTGACGGCAACGTTAGGGAGTCCGGAGACGTCGGCGGGGGCCTCGGGAAGAGTTATCTTTTCTGTTTAACAGCCTGCCCACCCTGGAAACGGC	10 (0.000189%)	
GTGGGGAGTTTGGCTGGGGCGGCACATCTGTTAAAAGATAACGCAGGTGTCCTAAGATGAGCTCAACGAGAACAGAAATCTCGTGTGGAACAAAAGGGTA	9 (0.000170%)	
GTGGGTGGTTCGCCGCCCGCGACGTCGCGAGAAGTCCACTAAACCTTATCATTTAGAGGAAGGAGAAGTCGTAACAAGGTTTCCGTAGGTGAACCTGCGG	86 (0.001627%)	
GTGGTCATGGAAGTCGAAATCCGCTAAGGAGTGTGTAACAACTCACCTGCCGAATCAACTAGCCCCGAAAATGGATGGCGCTTAAGCGCGCGACCTATAC	12 (0.000227%)	
GTGGTTCGCCGCCCGCGACGTCGCGAGAAGTCCACTAAACCTTATCATTTAGAGGAAGGAGAAGTCGTAACAAGGTTTCCGTAGGTGAACCTGCGGAAGG	13 (0.000246%)	
GTGTAAAGTTGGGAATTCGTTAAGGAGCTGTTGCTTTGTTAGTGTAGAAACACTTGTGTAGAATTGGGGATTGTTTTTTTTGGAGTGATTTAGGGGAGGG	18 (0.000341%)	
GTGTAGGGAAAGAGTGTTCAGAGCCGTGTAGATCTCGGTG	25 (0.000189%)	
GTGTATCCTTGTTAGAAGACACAAAGCCAAAGACTCATAT	69 (0.000522%)	
GTGTATGATTGAGTATAAGAACTTAAACCGCAACCGCATCTTATAAGCCTAAGTAGTGTTTCCTTGTTAGAAGACACAAAGCCAAAGACTCATATGGACT	11 (0.000208%)	
GTGTCACAAATCGTCGTCCCTCACCATCCTTTGCTGATGCGGGACGGAAGCTGGTCTCCCGTGTGTTACCGCACGCGGTTGGCCTAAATCCGAGCCAAGG	7 (0.000132%)	
GTGTCGCCCATGGGCATCGACACCTTGCGGCTAGGAACTGGAACGAGACGGGTGGCAAAGATTTCGAGTAGCACTTCATACTACCGTGGGTTTTTTAAAC	6 (0.000114%)	
GTGTGAGCATGCCTGTCGGGACCCGAAAGATGGTGAACTATGCCTGAGCGGGGTAAAGCCAGAGGAAACTCTGGTGGAAGCCCGCAGCGATACTGACGTG	3 (0.000057%)	
GTGTGAGCGAGGTGTGAGTGTCGCCCATGGGCATCGACACCTTGCGGCTAGGAACTGGAACGAGACGGGTAGCAAAGATTTCGAGTAGCACTTCATACTA	22 (0.000416%)	
GTGTGAGCGAGGTGTGAGTGTCGCCCATGGGCATCGACACCTTGCGGCTAGGAACTGGAACGAGACGGGTGGCAAAGATTTCGAGTAGCACTTCATACTA	30 (0.000568%)	
GTGTGAGTGTCGCCCATGGGCATCGACACCTTGCGGCTAGGAACTGGAACGAGACGGGTAGCAAAGATTTCGAGTAGCACTTCATACTACCGTGGGTTTT	16 (0.000303%)	
GTGTGAGTGTCGCCCATGGGCATCGACACCTTGCGGCTAGGAACTGGAACGAGACGGGTGGCAAAGATTTCGAGTAGCACTTCATACTACCGTGGGTTTT	8 (0.000151%)	
GTGTTCGTAGGCTCCATGCTCGCGCATCGAACTACCTACCACCTATCCTTCTCAGTTAATTCACGGGCGATGTTACGCTCGATGATGAGTTCCGGGGCCT	7 (0.000132%)	
GTGTTGGTCGATTAAGACAGCAGGACGGTGGTCATGGAAGTCGAAATCCGCTAAGGAGTGTGTAACAACTCACCTGCCGAATCAACTAGCCCCGAAAATG	4 (0.000076%)	
GTGTTTCCTTGTTAGAAGACACAAAGCCAAAGACTCATATGGACTTTGGCTACACCATGAAAGCTTTGAGAAGCAAGAAGAAGGTTGGTTAGTGTTTTGG	28 (0.000530%)	
GTGTTTTGGAGTCGAATATGACTTGATGTCATGTGTATGA	215 (0.001627%)	
GTTAAAAGATAACGCAGGTGTCCTAAGATGAGCTCAACGAGAACAGAAATCTCGTGTGGAACAAAAGGGTAAAAGCTCGTTTGATTCTGATTTTCAGTAC	32 (0.000605%)	
GTTAAGGAGCTGTTGCTTTGTTAGTGTAGAAACACTTGTGTAGAATTGGGGATTGTTTTTTTTGGAGTGATTTAGGGGAGGGTCGAATCTTAGCGACAAA	40 (0.000757%)	
GTTAGAAGACACAAAGCCAAAGACTCATATGGACTTTGGCTACACCATGAAAGCTTTGAGAAGCAAGAAGAAGGTTGGTTAGTGTTTTGGAGTCGAATAT	80 (0.001513%)	
GTTAGGGAGTCCGGAGACGTCGGCGGGGGCCTCGGGAAGAGTTATCTTTTCTGTTTAACAGCCTGCCCACCCTGGAAACGGCTCAGCCGGAGGTAGGGTC	16 (0.000303%)	
GTTAGTGTTTTGGAGTCGAATATGACTTGATGTCATGTGT	16246 (0.122938%)	
GTTATCATGAATCATCAGAGCAACGGGCAGAGCCCGCGTCGACCTTTTATCTAATAAATGCGTCCCTTCCATAAGTCGGGGTTTGTTGCACGTATTAGCT	282 (0.005335%)	
GTTATCCCATGCTAATGTATCCAGAGCGTAGGCTTGCTTTGAGCACTCTAATTTCTTCAAAGTAACAGCGCCGGAGGCACGACCCGGCCAATTAAGACCA	47 (0.000889%)	
GTTCAATATGACAAACCCATGCCAAGTAAAGAGAAAATGAAAACTGGTGATTGTTGCGGAAATCGTCCAGGATTCCTCGACCAGGACTTGAAATCGTCGA	25 (0.000473%)	
GTTCACATTTCGTTCATCACCCTTGGCCGGCTATCGAACAGCCGGACTCCCATCAAAAGATGGTTGCCAAGAACATCTTCGTTACGGTTTGCTAATTCTC	15 (0.000284%)	
GTTCACATTTCGTTCATCACCCTTGGCCGGCTTTCGAACAGCCGGACTCCCATCAAAAGATGGTTGCCAAGAACATCTTCGTTACGGTTTGCTAATTCTC	18 (0.000341%)	
GTTCATCACCCTTGGCCGGCTTTCGAACAGCCGGACTCCCATCAAAAGATGGTTGCCAAGAACATCTTCGTTACGGTTTGCTAATTCTCGGAATAACATC	9 (0.000170%)	
GTTCGAGTGTGAGCATGCCTGTCGGGACCCGAAAGATGGTGAACTATGCCTGAGCGGGGTAAAGCCAGAGGAAACTCTGGTGGAAGCCCGCAGCGATACT	4 (0.000076%)	

GTTCGATTAGTCTTTCGCCCCTATACCCAAGTCAGACGAACGATTTGCACGTCAGTATCGCTGCGGGCTTCCACCAGAGTTTCCTCTGGCTTTACCCCGC	784 (0.014832%)	
GTTCGCCGCCCGCGACGTCGCGAGAAGTCCACTAAACCTTATCATTTAGAGGAAGGAGAAGTCGTAACAAGGTTTCCGTAGGTGAACCTGCGGAAGGATC	33 (0.000624%)	
GTTCGTAGGCTCCATGCTCGCGCATCGAACTACCTACCACCTATCCTTCTCAGTTAATTCACGGGCGATGTTACGCTCGATGATGAGTTCCGGGGCCTGT	17 (0.000322%)	
GTTCGTATTCGTACTGAAAATCAGAATCAAACGAGCTTTTACCCTTTTGTTCCACACGAGATTTCTGTTCTCGTTGAGCTCATCTTAGGACACCTGCGTT	12 (0.000227%)	
GTTCTTATACTCAATCATACACATGACATCAAGTCATATTCGACTCCAAAACACTAACCAACCTTCTTCTTGCTTCTCAAAGCTTTCATGGTGTAGCCAA	86 (0.001627%)	
GTTGAAATCGTCGACCAGGTCCGAGACTTCATCGACCGGGTCCGAGGATTCGTCGACCAGGACGGCCGGATGTCCGAGAAAAAAAAATGTTGCCGAATAA	46 (0.000870%)	
GTTGACGGCAACGTTAGGGAGTCCGGAGACGTCGGCGGGGGCCTCGGGAAGAGTTATCTTTTCTGTTTAACAGCCTGCCCACCCTGGAAACGGCTCAGCC	38 (0.000719%)	
GTTGACTCGCACACATGTCAGACTCCTTGGTCCGTGTTTCAAGACGGGTCGAATGGGGAGCCCACAGGCCGACGCCCGGAGCACGCTGATGCCGAGGCAC	47 (0.000889%)	
GTTGCGGTTTAAGTTCTTATACTCAATCATACACATGACATCAAGTCATATTCGACTCCAAAACACTAACCAACCTTCTTCTTGCTTCTCAAAGCTTTCA	277 (0.005240%)	
GTTGCGGTTTAAGTTGTTATACTCAATCATACACATGACAACAAGTCATATTCGACTCCAAAACACTAACCAACCTTCTTCTTGCTTCTCAAAGCTTTCA	148 (0.002800%)	
GTTGGTCGATTAAGACAGCAGGACGGTGGTCATGGAAGTCGAAATCCGCTAAGGAGTGTGTAACAACTCACCTGCCGAATCAACTAGCCCCGAAAATGGA	17 (0.000322%)	
GTTGGTCTTCAACGAGGAATTCCTAGTAAGCGCGAGTCATCAGCTCGCGTTGACTACGTCCCTGCCCTTTGTACACACCGCCCGTCGCTCCTACCGATTG	174 (0.003292%)	
GTTGTTATACTCAATCATACACATGACAACAAGTCATATTCGACTCCAAAACACTAACCAACCTTCTTCTTGCTTCTCAAAGCTTTCATGGTGTAGCCAA	22 (0.000416%)	
GTTGTTATACTCAATCATACACATGACATCAAGTCATATT	256 (0.001937%)	
GTTTAAGTTCTTATACTCAATCATACACATGACATCAAGTCATATTCGACTCCAAAACACTAACCAACCTTCTTCTTGCTTCTCAAAGCTTTCATGGTGT	409 (0.007738%)	
GTTTAAGTTGTTATACTCAATCATACACATGACAACAAGTCATATTCGACTCCAAAACACTAACCAACCTTCTTCTTGCTTCTCAAAGCTTTCATGGTGT	118 (0.002232%)	
GTTTACTCACCCGTTGACTCGCACACATGTCAGACTCCTTGGTCCGTGTTTCAAGACGGGTCGAATGGGGAGCCCACAGGCCGACGCCCGGAGCACGCTG	26 (0.000492%)	
GTTTGGCTGGGGCGGCACATCTGTTAAAAGATAACGCAGGTGTCCTAAGATGAGCTCAACGAGAACAGAAATCTCGTGTGGAACAAAAGGGTAAAAGCTC	32 (0.000605%)	
GTTTTAATTAAACAGTCGGATTCCCCTTGTCCGTACCAGTTCTGAGCTGACTGTTCGACGCCCGGGGAAAGCTCCCGAGAGAGCCGTTCCCAGTCCGTCC	25 (0.000473%)	
GTTTTGGAGTCGAATATGACTTGATGTCATGTGTATGATT	475 (0.003594%)	
GTTTTTTCAGCAGTTCTCGGACAAAAATTGCTGAGTGGCCGAGAAGAATGGGCGTGTCATGCGTGGGCTGACATGGATTCTTCGAGGCCTAGGGGTGGCG	19 (0.000359%)	
TAAAAGATAACGCAGGTGTCCTAAGATGAGCTCAACGAGAACAGAAATCTCGTGTGGAACAAAAGGGTAAAAGCTCGTTTGATTCTGATTTTCAGTACGA	22 (0.000416%)	
TAAAAGTGTCGGAGTTTTTTCAGCAGTTCTCGGACAAAAATTGCTGAGTGGCCGAGAAGAATGGGCGTGTCATGCGTGGGCTGACATGGATTCTTCGAGG	6 (0.000114%)	
TAAAGGCGTAAGAATTGTATCCTTGTTAGAAGACACAAAG	576 (0.004359%)	
TAAAGTTGGGAATTCGTTAAGGAGCTGTTGCTTTGTTAGTGTAGAAACACTTGTGTAGAATTGGGGATTGTTTTTTTTGGAGTGATTTAGGGGAGGGTCG	7 (0.000132%)	
TAAATACGGGCGAGAGACCGATAGCGAACAAGTACCGCGAGGTAAAGATGAAAAGGACTTTGAAAAGAGAGTCAAAGAGTGCTTGAAATTGTCGGGAGGG	22 (0.000416%)	
TAACAGGGACAGTCGGGGGCATTCGTATTTCATAGTCAGAGGTGAAATTCTTGGATTTATGAAAGACGAACAACTGCGAAAGCATTTGCCAAGGATGTTT	55 (0.001041%)	
TAACGGCGTGCCTCGGCATCAGCGTGCTCCGGGCGTCGGCCTGTGGGCTCCCCATTCGACCCGTCTTGAAACACGGACCAAGGAGTCTGACATGTGTGCG	55 (0.001041%)	
TAACTAGCTACGTGGAGGCATCCCTTCACGGCCGGCTTCTTAGAGGGACTATGGCCGTTTAGGCCAAGGAAGTTTGAGGCAATAACAGGTCTGTGATGCC	16 (0.000303%)	
TAAGAACTTAAACCGCAACCGCATCTTATAAGCCTAAGTAGTGTTTCCTTGTTAGAAGACACAAAGCCAAAGACTCATATGGACTTTGGCTACACCATGA	26 (0.000492%)	
TAAGAATTGTATCCTTGTTAGAAGACACAAAGCCAAAGACTCATATGGACTTTGGCTACACCATGAAAGCTTTGAGAAGCAAGAAGAAGGTTGGTTAGTG	1088 (0.020583%)	
TAAGACAGCAGGACGGTGGTCATGGAAGTCGAAATCCGCTAAGGAGTGTGTAACAACTCACCTGCCGAATCAACTAGCCCCGAAAATGGATGGCGCTTAA	55 (0.001041%)	
TAAGGAGCTGTTGCTTTGTTAGTGTAGAAACACTTGTGTAGAATTGGGGATTGTTTTTTTTGGAGTGATTTAGGGGAGGGTCGAATCTTAGCGACAAAGG	24 (0.000454%)	
TAAGTAGTGTTTCCTTGTTAGAAGACACAAAGCCAAAGACTCATATGGACTTTGGCTACACCATGAAAGCTTTGAGAAGCAAGAAGAAGGTTGGTTAGTG	44 (0.000832%)	
TAAGTTCTTATACTCAATCATACACATGACATCAAGTCATATTCGACTCCAAAACACTAACCAACCTTCTTCTTGCTTCTCAAAGCTTTCATGGTGTAGC	41 (0.000776%)	
TAAGTTGTTATACTCAATCATACACATGACAACAAGTCATATTCGACTCCAAAACACTAACCAACCTTCTTCTTGCTTCTCAAAGCTTTCATGGTGTAGC	10 (0.000189%)	
TAAGTTGTTATACTCAATCATACACATGACATCAAGTCAT	9616 (0.072767%)	
TAATTCCAGCTCCAATAGCGTATATTTAAGTTGTTGCAGTTAAAAAGCTCGTAGTTGAACCTTGGGATGGGTCGGCCGGTCCGCCTTTGGTGTGCATTGG	10 (0.000189%)	
TACACATGACATCAAGTCATATTCGACTCCAAAACACTAACCAACCTTCTTCTTGCTTCTCAAAGCTTTCATGGTGTAGCCAAAGTCCATATGAGTCTTT	23 (0.000435%)	
TACATTGTTCCATCGACCAGAGGCTGTTCACCTTGGAGACCTGATGCGGTTATGAGTACGACCGGGCGTGAGCGGCACTCGGTCCTCCGGATTTTCAAGG	3 (0.000057%)	
TACATTTTATCGGTCGCTCTTGTCCGGAAGCTGTAGATGACCCAAAGTCCATATAGCGACCCCAGGTCAGGCGGGATTACCCGCTGAGTTTAAGCATATC	14 (0.000265%)	
TACCTACCACCTATCCTTCTCAGTTAATTCACGGGCGATGTTACGCTCGATGATGAGTTCCGGGGCCTGTGTTTCGTACCTAATTTGAAGGAATTGTTGA	3 (0.000057%)	
TACGAAACACAGGCCCCGGAACTCATCATCGAGCGTAACATCGCCCGTGAATTAACTGAGAAGGATAGGTGGTAGGTAGTTCGATGCGCGAGCATGGAGC	6 (0.000114%)	

TACGGCTTTGGCTCGGATTCGTCCGTCTTCTTTCTTCTTAGCCGAGTACTTCGGTAGATTAGTTGGAACGATTGATGATTTTGAGTTAATTGAACGTTCG	3 (0.000057%)	
TACGGGTTTACTCACCCGTTGACTCGCACACATGTCAGACTCCTTGGTCCGTGTTTCAAGACGGGTCGAATGGGGAGCCCACAGGCCGACGCCCGGAGCA	6 (0.000114%)	
TACTCAATCATACACATGACATCAAGTCATATTCGACTCCAAAACACTAACCAACCTTCTTCTTGCTTCTCAAAGCTTTCATGGTGTAGCCAAAGTCCAT	23 (0.000435%)	
TACTCACCCGTTGACTCGCACACATGTCAGACTCCTTGGTCCGTGTTTCAAGACGGGTCGAATGGGGAGCCCACAGGCCGACGCCCGGAGCACGCTGATG	6 (0.000114%)	
TAGAAGACACAAAGCCAAAGACTCATATGGACTTTGGCTACACCATGAAAGCTTTGAGAAGCAAGAAGAAGGTTGGTTAGTGTTTTGGAGTCGAATATGA	38 (0.000719%)	
TAGCTAGTGTTCGTAGGCTCCATGCTCGCGCATCGAACTACCTACCACCTATCCTTCTCAGTTAATTCACGGGCGATGTTACGCTCGATGATGAGTTCCG	4 (0.000076%)	
TAGGAGCGACGGGCGGTGTGTACAAAGGGCAGGGACGTAGTCAACGCGAGCTGATGACTCGCGCTTACTAGGAATTCCTCGTTGAAGACCAACAATTGCA	12 (0.000227%)	
TAGGCGAGACAAGGGTTCACATTTCGTTCATCACCCTTGGCCGGCTATCGAACAGCCGGACTCCCATCAAAAGATGGTTGCCAAGAACATCTTCGTTACG	4 (0.000076%)	
TAGGCGAGACAAGGGTTCACATTTCGTTCATCACCCTTGGCCGGCTTTCGAACAGCCGGACTCCCATCAAAAGATGGTTGCCAAGAACATCTTCGTTACG	3 (0.000057%)	
TAGGCTGTCCCGAAGGTATCTCGCGCTTGTACGGCTTTGGCTCGGATTCGTCCGTCTTCTTTCTTCTTAGCCGAGTACTTCGGTAGATTAGTTGGAACGA	1809 (0.034223%)	
TAGGCTGTCCCGAGTGTGAGCGAGGTGTGAGTGTCGCCCATGGGCATCGACACCTTGCGGCTAGGAACTGGAACGAGACGGGTGGCAAAGATTTCGAGTA	41 (0.000776%)	
TAGGGAAAGAGTGTTCAGAGCCGTGTAGATCTCGGTGGTC	13 (0.000098%)	
TAGGGAGTCCGGAGACGTCGGCGGGGGCCTCGGGAAGAGTTATCTTTTCTGTTTAACAGCCTGCCCACCCTGGAAACGGCTCAGCCGGAGGTAGGGTCCA	7 (0.000132%)	
TAGGTACGAAACACAGGCCCCGGAACTCATCATCGAGCGTAACATCGCCCGTGAATTAACTGAGAAGGATAGGTGGTAGGTAGTTCGATGCGCGAGCATG	16 (0.000303%)	
TAGTCATATGCTTGTCTCAAAGATTAAGCCATGCATGTGTAAGTATGAACGAATTCAGACTGTGAAACTGCGAATGGCTCATTAAATCAGTTATAGTTTG	32 (0.000605%)	
TAGTGTTCGTAGGCTCCATGCTCGCGCATCGAACTACCTACCACCTATCCTTCTCAGTTAATTCACGGGCGATGTTACGCTCGATGATGAGTTCCGGGGC	5 (0.000095%)	
TAGTGTTTCCTTGTTAGAAGACACAAAGCCAAAGACTCATATGGACTTTGGCTACACCATGAAAGCTTTGAGAAGCAAGAAGAAGGTTGGTTAGTGTTTT	23 (0.000435%)	
TAGTGTTTTGGAGTCGAATATGACTTGATGTCATGTGTAT	264 (0.001998%)	
TATAAGAACTTAAACCGCAACCGCATCTTATAAGCCTAAGTAGTGTTTCCTTGTTAGAAGACACAAAGCCAAAGACTCATATGGACTTTGGCTACACCAT	16 (0.000303%)	
TATACTCAATCATACACATGACATCAAGTCATATTCGACTCCAAAACACTAACCAACCTTCTTCTTGCTTCTCAAAGCTTTCATGGTGTAGCCAAAGTCC	36 (0.000681%)	
TATCAACTTTCGATGGTAGGATAGTGGCCTACCATGGTGGTAACGGGTGACGGAGAATTAGGGTTCGATTCCGGAGAGGGAGCCTGAGAAACGGCTACCA	3 (0.000057%)	
TATCATGAATCATCAGAGCAACGGGCAGAGCCCGCGTCGACCTTTTATCTAATAAATGCGTCCCTTCCATAAGTCGGGGTTTGTTGCACGTATTAGCTCT	64 (0.001211%)	
TATCCAGAGCGTAGGCTTGCTTTGAGCACTCTAATTTCTTCAAAGTAACAGCGCCGGAGGCACGACCCGGCCAATTAAGACCAGGAGCGTATCGCCGACC	7 (0.000132%)	
TATCCTTGTTAGAAGACACAAAGCCAAAGACTCATATGGACTTTGGCTACACCATGAAAGCTTTGAGAAGCAAGAAGAAGGTTGGTTAGTGTTTTGGAGT	35 (0.000662%)	
TATGAACACAAACGTTCAATATGACAAACCCATGCCAAGTAAAGAGAAAATGAAAACTGGTGATTGTTGCGGAAATCGTCCAGGATTCCTCGACCAGGAC	4 (0.000076%)	
TATGATGTTATCCCATGCTAATGTATCCAGAGCGTAGGCTTGCTTTGAGCACTCTAATTTCTTCAAAGTAACAGCGCCGGAGGCACGACCCGGCCAATTA	25 (0.000473%)	
TATGATTGAGTATAAGAACTTAAACCGCAACCGCATCTTATAAGCCTAAGTAGTGTTTCCTTGTTAGAAGACACAAAGCCAAAGACTCATATGGACTTTG	16 (0.000303%)	
TATGCTTGTCTCAAAGATTAAGCCATGCATGTGTAAGTATGAACGAATTCAGACTGTGAAACTGCGAATGGCTCATTAAATCAGTTATAGTTTGTTTGAT	7 (0.000132%)	
TATTCGACTCCAAAACACTAACCAACCTTCTTCTTGCTTCTCAAAGCTTTCATGGTGTAGCCAAAGTCCATATGAGTCTTTGGCTTTGTGTCTTCTAACA	48 (0.000908%)	
TATTCGACTCCAAAACACTAACCAACCTTCTTCTTGCTTCTCAAAGCTTTCATGGTGTAGCCAAAGTCCATATGAGTCTTTGGCTTTGTGTCTTTTAACA	8 (0.000151%)	
TATTCGTACTGAAAATCAGAATCAAACGAGCTTTTACCCTTTTGTTCCACACGAGATTTCTGTTCTCGTTGAGCTCATCTTAGGACACCTGCGTTATCTT	4 (0.000076%)	
TATTTCATAGTCAGAGGTGAAATTCTTGGATTTATGAAAGACGAACAACTGCGAAAGCATTTGCCAAGGATGTTTTCATTAATCAAGAACGAAAGTTGGG	3 (0.000057%)	
TCAAAATCATCAATCGTTCCAACTAATCTACCGAAGTACTCGGCTAAGAAGAAAGAAGACGGACGAATCCGAGCCAAAGCCGTACAAGCGCGAGATACCT	17 (0.000322%)	
TCAAAGATTAAGCCATGCATGTGTAAGTATGAACGAATTCAGACTGTGAAACTGCGAATGGCTCATTAAATCAGTTATAGTTTGTTTGATGGTAACTACT	8 (0.000151%)	
TCAAATTAGGTACGAAACACAGGCCCCGGAACTCATCATCGAGCGTAACATCGCCCGTGAATTAACTGAGAAGGATAGGTGGTAGGTAGTTCGATGCGCG	3 (0.000057%)	
TCAAATTTCTGCCCTATCAACTTTCGATGGTAGGATAGTGGCCTACCATGGTGGTAACGGGTGACGGAGAATTAGGGTTCGATTCCGGAGAGGGAGCCTG	16 (0.000303%)	
TCAACTTTCGATGGTAGGATAGTGGCCTACCATGGTGGTAACGGGTGACGGAGAATTAGGGTTCGATTCCGGAGAGGGAGCCTGAGAAACGGCTACCACA	3 (0.000057%)	
TCAAGGGCCGCCGGGGGCGCACCGGACACCACGCGACGTGCGGTGCTCTTCCAGCCGCTGGACCCTACCTCCGGCTGAGCCGTTTCCAGGGTGGGCAGGC	8 (0.000151%)	
TCAAGTCATATTCGACTCCAAAACACTAACCAACCTTCTTCTTGCTTCTCAAAGCTTTCATGGTGTAGCCAAAGTCCATATGAGTCTTTGGCTTTGTGTC	39 (0.000738%)	
TCAATCATACACATGACATCAAGTCATATTCGACTCCAAAACACTAACCAACCTTCTTCTTGCTTCTCAAAGCTTTCATGGTGTAGCCAAAGTCCATATG	34 (0.000643%)	
TCAATCGGTAGGAGCGACGGGCGGTGTGTACAAAGGGCAGGGACGTAGTCAACGCGAGCTGATGACTCGCGCTTACTAGGAATTCCTCGTTGAAGACCAA	45 (0.000851%)	
TCACATTTCGTTCATCACCCTTGGCCGGCTATCGAACAGCCGGACTCCCATCAAAAGATGGTTGCCAAGAACATCTTCGTTACGGTTTGCTAATTCTCGG	10 (0.000189%)	
TCACATTTCGTTCATCACCCTTGGCCGGCTTTCGAACAGCCGGACTCCCATCAAAAGATGGTTGCCAAGAACATCTTCGTTACGGTTTGCTAATTCTCGG	3 (0.000057%)	

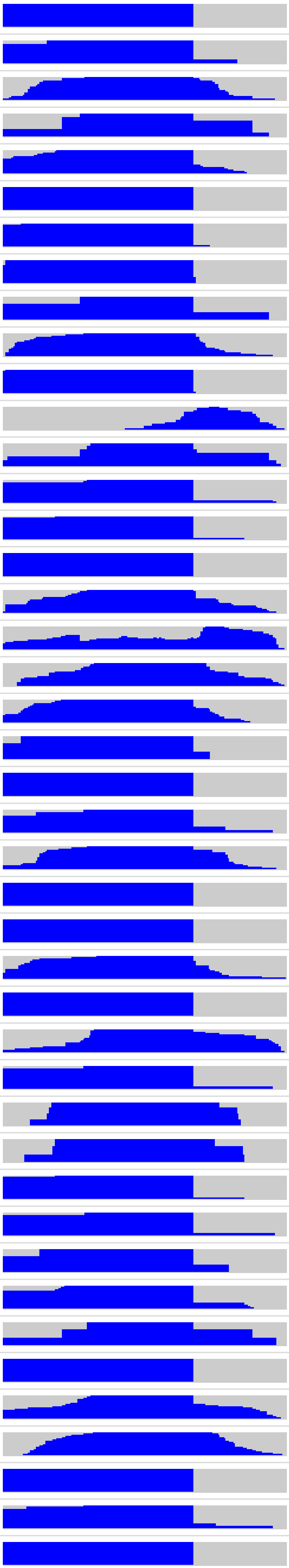

TCACCATCCTTTGCTGATGCGGGACGGAAGCTGGTCTCCCGTGTGTTACCGCACGCGGTTGGCCTAAATCCGAGCCAAGGACGCCTGGAGCGTACCGACA	8 (0.000151%)	
TCAGCCTGCTAACTAGCTACGTGGAGGCATCCCTTCACGGCCGGCTTCTTAGAGGGACTATGGCCGTTTAGGCCAAGGAAGTTTGAGGCAATAACAGGTC	5 (0.000095%)	
TCAGCGTGCTCCGGGCGTCGGCCTGTGGGCTCCCCATTCGACCCGTCTTGAAACACGGACCAAGGAGTCTGACATGTGTGCGAGTCAACGGGTGAGTAAA	4 (0.000076%)	
TCAGCTTCCTTGCGCCTTACGGGTTTACTCACCCGTTGACTCGCACACATGTCAGACTCCTTGGTCCGTGTTTCAAGACGGGTCGAATGGGGAGCCCACA	26 (0.000492%)	
TCATACACATGACATCAAGTCATATTCGACTCCAAAACACTAACCAACCTTCTTCTTGCTTCTCAAAGCTTTCATGGTGTAGCCAAAGTCCATATGAGTC	5 (0.000095%)	
TCATATGCTTGTCTCAAAGATTAAGCCATGCATGTGTAAGTATGAACGAATTCAGACTGTGAAACTGCGAATGGCTCATTAAATCAGTTATAGTTTGTTT	6 (0.000114%)	
TCATATTCGACTCCAAAACACTAACCAACCTTCTTCTTGCTTCTCAAAGCTTTCATGGTGTAGCCAAAGTCCATATGAGTCTTTGGCTTTGTGTCTTCTA	68 (0.001286%)	
TCATCAGAGCAACGGGCAGAGCCCGCGTCGACCTTTTATCTAATAAATGCGTCCCTTCCATAAGTCGGGGTTTGTTGCACGTATTAGCTCTAGAATTACT	5 (0.000095%)	
TCATCATCGAGCGTAACATCGCCCGTGAATTAACTGAGAAGGATAGGTGGTAGGTAGTTCGATGCGCGAGCATGGAGCCTACGAACACTAGCTATCCGAT	227 (0.004294%)	
TCATGAATCATCAGAGCAACGGGCAGAGCCCGCGTCGACCTTTTATCTAATAAATGCGTCCCTTCCATAAGTCGGGGTTTGTTGCACGTATTAGCTCTAG	21 (0.000397%)	
TCATGTGTATGATTGAGTATAAGAACTTAAACCGCAACCGCATCTTATAAGCCTAAGTAGTGTTTCCTTGTTAGAAGACACAAAGCCAAAGACTCATATG	8 (0.000151%)	
TCATTCAAATTTCTGCCCTATCAACTTTCGATGGTAGGATAGTGGCCTACCATGGTGGTAACGGGTGACGGAGAATTAGGGTTCGATTCCGGAGAGGGAG	3 (0.000057%)	
TCCAACCTAGGCGAGACAAGGGTTCACATTTCGTTCATCACCCTTGGCCGGCTATCGAACAGCCGGACTCCCATCAAAAGATGGTTGCCAAGAACATCTT	3 (0.000057%)	
TCCAACCTAGGCGAGACAAGGGTTCACATTTCGTTCATCACCCTTGGCCGGCTTTCGAACAGCCGGACTCCCATCAAAAGATGGTTGCCAAGAACATCTT	4 (0.000076%)	
TCCAACGAAGCACGCCCATCCAACCTAGGCGAGACAAGGGTTCACATTTCGTTCATCACCCTTGGCCGGCTATCGAACAGCCGGACTCCCATCAAAAGAT	17 (0.000322%)	
TCCAGAGCGTAGGCTTGCTTTGAGCACTCTAATTTCTTCAAAGTAACAGCGCCGGAGGCACGACCCGGCCAATTAAGACCAGGAGCGTATCGCCGACCGA	4 (0.000076%)	
TCCAGCTCCAATAGCGTATATTTAAGTTGTTGCAGTTAAAAAGCTCGTAGTTGAACCTTGGGATGGGTCGGCCGGTCCGCCTTTGGTGTGCATTGGTCGG	50 (0.000946%)	
TCCATGCTCGCGCATCGAACTACCTACCACCTATCCTTCTCAGTTAATTCACGGGCGATGTTACGCTCGATGATGAGTTCCGGGGCCTGTGTTTCGTACC	3 (0.000057%)	
TCCCATGCTAATGTATCCAGAGCGTAGGCTTGCTTTGAGCACTCTAATTTCTTCAAAGTAACAGCGCCGGAGGCACGACCCGGCCAATTAAGACCAGGAG	3 (0.000057%)	
TCCCGAAGGTATCTCGCGCTTGTACGGCTTTGGCTCGGATTCGTCCGTCTTCTTTCTTCTTAGCCGAGTACTTCGGTAGATTAGTTGGAACGATTGATGA	3 (0.000057%)	
TCCCGCCAATCAGCTTCCTTGCGCCTTACGGGTTTACTCACCCGTTGACTCGCACACATGTCAGACTCCTTGGTCCGTGTTTCAAGACGGGTCGAATGGG	5 (0.000095%)	
TCCCTTGCCTACATTGTTCCATCGACCAGAGGCTGTTCACCTTGGAGACCTGATGCGGTTATGAGTACGACCGGGCGTGAGCGGCACTCGGTCCTCCGGA	5 (0.000095%)	
TCCGCCTAGGCTGTCCCGAGTGTGAGCGAGGTGTGAGTGTCGCCCATGGGCATCGACACCTTGCGGCTAGGAACTGGAACGAGACGGGTGGCAAAGATTT	2010 (0.038026%)	
TCCGGAACCGGGACGTGGCGGTTGACGGCAACGTTAGGGAGTCCGGAGACGTCGGCGGGGGCCTCGGGAAGAGTTATCTTTTCTGTTTAACAGCCTGCCC	21 (0.000397%)	
TCCGGATTTTCAAGGGCCGCCGGGGGCGCACCGGACACCACGCGACGTGCGGTGCTCTTCCAGCCGCTGGACCCTACCTCCGGCTGAGCCGTTTCCAGGG	5 (0.000095%)	
TCCGGGCGTCGGCCTGTGGGCTCCCCATTCGACCCGTCTTGAAACACGGACCAAGGAGTCTGACATGTGTGCGAGTCAACGGGTGAGTAAACCCGTAAGG	3 (0.000057%)	
TCCGTCGAGTTATCATGAATCATCAGAGCAACGGGCAGAGCCCGCGTCGACCTTTTATCTAATAAATGCGTCCCTTCCATAAGTCGGGGTTTGTTGCACG	22 (0.000416%)	
TCCTATGATGTTATCCCATGCTAATGTATCCAGAGCGTAGGCTTGCTTTGAGCACTCTAATTTCTTCAAAGTAACAGCGCCGGAGGCACGACCCGGCCAA	5 (0.000095%)	
TCCTTGCGCCTTACGGGTTTACTCACCCGTTGACTCGCACACATGTCAGACTCCTTGGTCCGTGTTTCAAGACGGGTCGAATGGGGAGCCCACAGGCCGA	6 (0.000114%)	
TCCTTGTTAGAAGACACAAAGCCAAAGACTCATATGGACTTTGGCTACACCATGAAAGCTTTGAGAAGCAAGAAGAAGGTTGGTTAGTGTTTTGGAGTCG	240 (0.004540%)	
TCGAAATCCTATGATGTTATCCCATGCTAATGTATCCAGAGCGTAGGCTTGCTTTGAGCACTCTAATTTCTTCAAAGTAACAGCGCCGGAGGCACGACCC	238 (0.004503%)	
TCGACCAGGGGTTGAAATCGTCGACCAGGTCCGAGACTTCATCGACCGGGTCCGAGGATTCGTCGACCAGGACGGCCGGATGTCCGAGAAAAAAAAATGT	9 (0.000170%)	
TCGACCCGCCGAAGCGAGCCTTGGGACCAAAAACAGGGGTTGTACCCCGCCTCCGATTCACGGAGTAAGTAAAATAACGTTAAAAGTAGTGGTATTTCAC	5 (0.000095%)	
TCGACTCCAAAACACTAACCAACCTTCTTCTTGCTTCTCAAAGCTTTCATGGTGTAGCCAAAGTCCATATGAGTCTTTGGCTTTGTGTCTTCTAACAAGG	43 (0.000813%)	
TCGACTCCAAAACACTAACCAACCTTCTTCTTGCTTCTCAAAGCTTTCATGGTGTAGCCAAAGTCCATATGAGTCTTTGGCTTTGTGTCTTTTAACAAGG	14 (0.000265%)	
TCGAGTTATCATGAATCATCAGAGCAACGGGCAGAGCCCGCGTCGACCTTTTATCTAATAAATGCGTCCCTTCCATAAGTCGGGGTTTGTTGCACGTATT	66 (0.001249%)	
TCGATCCATTACATTTTATCGGTCGCTCTTGTCCGGAAGCTGTAGATGACCCAAAGTCCATATAGCGACCCCAGGTCAGGCGGGATTACCCGCTGAGTTT	5 (0.000095%)	
TCGATCCGTCGAGTTATCATGAATCATCAGAGCAACGGGCAGAGCCCGCGTCGACCTTTTATCTAATAAATGCGTCCCTTCCATAAGTCGGGGTTTGTTG	4 (0.000076%)	
TCGATCGACCCGCCGAAGCGAGCCTTGGGACCAAAAACAGGGGTTGTACCCCGCCTCCGATTCACGGAGTAAGTAAAATAACGTTAAAAGTAGTGGTATT	5 (0.000095%)	
TCGATCTCATGTGTATGATTGAGTATAAGAACTTAAACCGCAACCGCATCTTATAAGCCTAAGTAGTGTTTCCTTGTTAGAAGACACAAAGCCAAAGACT	3 (0.000057%)	
TCGATTAGTCTTTCGCCCCTATACCCAAGTCAGACGAACGATTTGCACGTCAGTATCGCTGCGGGCTTCCACCAGAGTTTCCTCTGGCTTTACCCCGCTC	5 (0.000095%)	
TCGCACACATGTCAGACTCCTTGGTCCGTGTTTCAAGACGGGTCGAATGGGGAGCCCACAGGCCGACGCCCGGAGCACGCTGATGCCGAGGCACGCCGTT	6 (0.000114%)	
TCGCCCATGGGCATCGACACCTTGCGGCTAGGAACTGGAACGAGACGGGTGGCAAAGATTTCGAGTAGCACTTCATACTACCGTGGGTTTTTTAAACCTT	4 (0.000076%)	

TCGCCGCCCGCGACGTCGCGAGAAGTCCACTAAACCTTATCATTTAGAGGAAGGAGAAGTCGTAACAAGGTTTCCGTAGGTGAACCTGCGGAAGGATCGA	6 (0.000114%)	
TCGCGCTTGTACGGCTTTGGCTCGGATTCGTCCGTCTTCTTTCTTCTTAGCCGAGTACTTCGGTAGATTAGTTGGAACGATTGATGATTTTGAGTTAATT	3 (0.000057%)	
TCGGCATCAGCGTGCTCCGGGCGTCGGCCTGTGGGCTCCCCATTCGACCCGTCTTGAAACACGGACCAAGGAGTCTGACATGTGTGCGAGTCAACGGGTG	25 (0.000473%)	
TCGGCCTGTGGGCTCCCCATTCGACCCGTCTTGAAACACGGACCAAGGAGTCTGACATGTGTGCGAGTCAACGGGTGAGTAAACCCGTAAGGCGCAAGGA	44 (0.000832%)	
TCGGTAGGAGCGACGGGCGGTGTGTACAAAGGGCAGGGACGTAGTCAACGCGAGCTGATGACTCGCGCTTACTAGGAATTCCTCGTTGAAGACCAACAAT	31 (0.000586%)	
TCGGTCCTCCGGATTTTCAAGGGCCGCCGGGGGCGCACCGGACACCACGCGACGTGCGGTGCTCTTCCAGCCGCTGGACCCTACCTCCGGCTGAGCCGTT	10 (0.000189%)	
TCGTACTGAAAATCAGAATCAAACGAGCTTTTACCCTTTTGTTCCACACGAGATTTCTGTTCTCGTTGAGCTCATCTTAGGACACCTGCGTTATCTTTTA	3 (0.000057%)	
TCGTAGGCTCCATGCTCGCGCATCGAACTACCTACCACCTATCCTTCTCAGTTAATTCACGGGCGATGTTACGCTCGATGATGAGTTCCGGGGCCTGTGT	6 (0.000114%)	
TCGTATTCGTACTGAAAATCAGAATCAAACGAGCTTTTACCCTTTTGTTCCACACGAGATTTCTGTTCTCGTTGAGCTCATCTTAGGACACCTGCGTTAT	9 (0.000170%)	
TCGTCCCTCACCATCCTTTGCTGATGCGGGACGGAAGCTGGTCTCCCGTGTGTTACCGCACGCGGTTGGCCTAAATCCGAGCCAAGGACGCCTGGAGCGT	3 (0.000057%)	
TCGTTCATCACCCTTGGCCGGCTTTCGAACAGCCGGACTCCCATCAAAAGATGGTTGCCAAGAACATCTTCGTTACGGTTTGCTAATTCTCGGAATAACA	4 (0.000076%)	
TCTCGCGCTTGTACGGCTTTGGCTCGGATTCGTCCGTCTTCTTTCTTCTTAGCCGAGTACTTCGGTAGATTAGTTGGAACGATTGATGATTTTGAGTTAA	10 (0.000189%)	
TCTGAGAAGGGTTCGAGTGTGAGCATGCCTGTCGGGACCCGAAAGATGGTGAACTATGCCTGAGCGGGGTAAAGCCAGAGGAAACTCTGGTGGAAGCCCG	4 (0.000076%)	
TCTGCCCTATCAACTTTCGATGGTAGGATAGTGGCCTACCATGGTGGTAACGGGTGACGGAGAATTAGGGTTCGATTCCGGAGAGGGAGCCTGAGAAACG	8 (0.000151%)	
TCTGGTGCCAGCAGCCGCGGTAATTCCAGCTCCAATAGCGTATATTTAAGTTGTTGCAGTTAAAAAGCTCGTAGTTGAACCTTGGGATGGGTCGGCCGGT	17 (0.000322%)	
TCTGTGCTGGCGACGCATCATTCAAATTTCTGCCCTATCAACTTTCGATGGTAGGATAGTGGCCTACCATGGTGGTAACGGGTGACGGAGAATTAGGGTT	21 (0.000397%)	
TCTTAAAGGCGTAAGAATTGTATCCTTGTTAGAAGACACA	415 (0.003140%)	
TCTTATACTCAATCATACACATGACATCAAGTCATATTCGACTCCAAAACACTAACCAACCTTCTTCTTGCTTCTCAAAGCTTTCATGGTGTAGCCAAAG	208 (0.003935%)	
TCTTCAACGAGGAATTCCTAGTAAGCGCGAGTCATCAGCTCGCGTTGACTACGTCCCTGCCCTTTGTACACACCGCCCGTCGCTCCTACCGATTGAATGA	19 (0.000359%)	
TGAACACAAACGTTCAATATGACAAACCCATGCCAAGTAAAGAGAAAATGAAAACTGGTGATTGTTGCGGAAATCGTCCAGGATTCCTCGACCAGGACTT	20 (0.000378%)	
TGAATCATCAGAGCAACGGGCAGAGCCCGCGTCGACCTTTTATCTAATAAATGCGTCCCTTCCATAAGTCGGGGTTTGTTGCACGTATTAGCTCTAGAAT	8 (0.000151%)	
TGACATCAAGTCATATTCGACTCCAAAACACTAACCAACCTTCTTCTTGCTTCTCAAAGCTTTCATGGTGTAGCCAAAGTCCATATGAGTCTTTGGCTTT	38 (0.000719%)	
TGACGGCAACGTTAGGGAGTCCGGAGACGTCGGCGGGGGCCTCGGGAAGAGTTATCTTTTCTGTTTAACAGCCTGCCCACCCTGGAAACGGCTCAGCCGG	15 (0.000284%)	
TGACTCGCACACATGTCAGACTCCTTGGTCCGTGTTTCAAGACGGGTCGAATGGGGAGCCCACAGGCCGACGCCCGGAGCACGCTGATGCCGAGGCACGC	6 (0.000114%)	
TGAGAAGGGTTCGAGTGTGAGCATGCCTGTCGGGACCCGAAAGATGGTGAACTATGCCTGAGCGGGGTAAAGCCAGAGGAAACTCTGGTGGAAGCCCGCA	8 (0.000151%)	
TGAGCGAGGTGTGAGTGTCGCCCATGGGCATCGACACCTTGCGGCTAGGAACTGGAACGAGACGGGTGGCAAAGATTTCGAGTAGCACTTCATACTACCG	4 (0.000076%)	
TGAGCGGCACTCGGTCCTCCGGATTTTCAAGGGCCGCCGGGGGCGCACCGGACACCACGCGACGTGCGGTGCTCTTCCAGCCGCTGGACCCTACCTCCGG	3 (0.000057%)	
TGAGTATAAGAACTTAAACCGCAACCGCATCTTATAAGCCTAAGTAGTGTTTCCTTGTTAGAAGACACAAAGCCAAAGACTCATATGGACTTTGGCTACA	21 (0.000397%)	
TGAGTGTCGCCCATGGGCATCGACACCTTGCGGCTAGGAACTGGAACGAGACGGGTGGCAAAGATTTCGAGTAGCACTTCATACTACCGTGGGTTTTTTA	5 (0.000095%)	
TGATGTTATCCCATGCTAATGTATCCAGAGCGTAGGCTTGCTTTGAGCACTCTAATTTCTTCAAAGTAACAGCGCCGGAGGCACGACCCGGCCAATTAAG	22 (0.000416%)	
TGATTAACAGGGACAGTCGGGGGCATTCGTATTTCATAGTCAGAGGTGAAATTCTTGGATTTATGAAAGACGAACAACTGCGAAAGCATTTGCCAAGGAT	109 (0.002062%)	
TGATTGAGTATAAGAACTTAAACCGCAACCGCATCTTATAAGCCTAAGTAGTGTTTCCTTGTTAGAAGACACAAAGCCAAAGACTCATATGGACTTTGGC	7 (0.000132%)	
TGCAGCACGCGCCTAACGGCGTGCCTCGGCATCAGCGTGCTCCGGGCGTCGGCCTGTGGGCTCCCCATTCGACCCGTCTTGAAACACGGACCAAGGAGTC	61 (0.001154%)	
TGCCGACTTCCCTTGCCTACATTGTTCCATCGACCAGAGGCTGTTCACCTTGGAGACCTGATGCGGTTATGAGTACGACCGGGCGTGAGCGGCACTCGGT	2363 (0.044704%)	
TGCCTACATTGTTCCATCGACCAGAGGCTGTTCACCTTGGAGACCTGATGCGGTTATGAGTACGACCGGGCGTGAGCGGCACTCGGTCCTCCGGATTTTC	8 (0.000151%)	
TGCGCCTTACGGGTTTACTCACCCGTTGACTCGCACACATGTCAGACTCCTTGGTCCGTGTTTCAAGACGGGTCGAATGGGGAGCCCACAGGCCGACGCC	15 (0.000284%)	
TGCGGTTTAAGTTCTTATACTCAATCATACACATGACATCAAGTCATATTCGACTCCAAAACACTAACCAACCTTCTTCTTGCTTCTCAAAGCTTTCATG	405 (0.007662%)	
TGCGGTTTAAGTTGTTATACTCAATCATACACATGACAACAAGTCATATTCGACTCCAAAACACTAACCAACCTTCTTCTTGCTTCTCAAAGCTTTCATG	110 (0.002081%)	
TGCTAACTAGCTACGTGGAGGCATCCCTTCACGGCCGGCTTCTTAGAGGGACTATGGCCGTTTAGGCCAAGGAAGTTTGAGGCAATAACAGGTCTGTGAT	19 (0.000359%)	
TGCTAATGTATCCAGAGCGTAGGCTTGCTTTGAGCACTCTAATTTCTTCAAAGTAACAGCGCCGGAGGCACGACCCGGCCAATTAAGACCAGGAGCGTAT	10 (0.000189%)	
TGCTCGCGCATCGAACTACCTACCACCTATCCTTCTCAGTTAATTCACGGGCGATGTTACGCTCGATGATGAGTTCCGGGGCCTGTGTTTCGTACCTAAT	4 (0.000076%)	
TGCTGATGCGGGACGGAAGCTGGTCTCCCGTGTGTTACCGCACGCGGTTGGCCTAAATCCGAGCCAAGGACGCCTGGAGCGTACCGACATGCGGTGGTGA	5 (0.000095%)	
TGCTGGCGACGCATCATTCAAATTTCTGCCCTATCAACTTTCGATGGTAGGATAGTGGCCTACCATGGTGGTAACGGGTGACGGAGAATTAGGGTTCGAT	5 (0.000095%)	

TGCTTGTCTCAAAGATTAAGCCATGCATGTGTAAGTATGAACGAATTCAGACTGTGAAACTGCGAATGGCTCATTAAATCAGTTATAGTTTGTTTGATGG	8 (0.000151%)	
TGCTTTGTTTTAATTAAACAGTCGGATTCCCCTTGTCCGTACCAGTTCTGAGCTGACTGTTCGACGCCCGGGGAAAGCTCCCGAGAGAGCCGTTCCCAGT	3 (0.000057%)	
TGGAAGTCGAAATCCGCTAAGGAGTGTGTAACAACTCACCTGCCGAATCAACTAGCCCCGAAAATGGATGGCGCTTAAGCGCGCGACCTATACCCGGCCG	88 (0.001665%)	
TGGAGGGCAAGTCTGGTGCCAGCAGCCGCGGTAATTCCAGCTCCAATAGCGTATATTTAAGTTGTTGCAGTTAAAAAGCTCGTAGTTGAACCTTGGGATG	296 (0.005600%)	
TGGCCTCTGTGCTGGCGACGCATCATTCAAATTTCTGCCCTATCAACTTTCGATGGTAGGATAGTGGCCTACCATGGTGGTAACGGGTGACGGAGAATTA	23 (0.000435%)	
TGGCGACGCATCATTCAAATTTCTGCCCTATCAACTTTCGATGGTAGGATAGTGGCCTACCATGGTGGTAACGGGTGACGGAGAATTAGGGTTCGATTCC	22 (0.000416%)	
TGGCGGTTGACGGCAACGTTAGGGAGTCCGGAGACGTCGGCGGGGGCCTCGGGAAGAGTTATCTTTTCTGTTTAACAGCCTGCCCACCCTGGAAACGGCT	4 (0.000076%)	
TGGCTGGGGCGGCACATCTGTTAAAAGATAACGCAGGTGTCCTAAGATGAGCTCAACGAGAACAGAAATCTCGTGTGGAACAAAAGGGTAAAAGCTCGTT	3 (0.000057%)	
TGGGCATCGACACCTTGCGGCTAGGAACTGGAACGAGACGGGTGGCAAAGATTTCGAGTAGCACTTCATACTACCGTGGGTTTTTTAAACCTTCCGAGTT	23 (0.000435%)	
TGGGGAGTTTGGCTGGGGCGGCACATCTGTTAAAAGATAACGCAGGTGTCCTAAGATGAGCTCAACGAGAACAGAAATCTCGTGTGGAACAAAAGGGTAA	5 (0.000095%)	
TGGGGCGGCACATCTGTTAAAAGATAACGCAGGTGTCCTAAGATGAGCTCAACGAGAACAGAAATCTCGTGTGGAACAAAAGGGTAAAAGCTCGTTTGAT	17 (0.000322%)	
TGGGTGGTTCGCCGCCCGCGACGTCGCGAGAAGTCCACTAAACCTTATCATTTAGAGGAAGGAGAAGTCGTAACAAGGTTTCCGTAGGTGAACCTGCGGA	10 (0.000189%)	
TGGGTGTCACAAATCGTCGTCCCTCACCATCCTTTGCTGATGCGGGACGGAAGCTGGTCTCCCGTGTGTTACCGCACGCGGTTGGCCTAAATCCGAGCCA	3 (0.000057%)	
TGGTCATGGAAGTCGAAATCCGCTAAGGAGTGTGTAACAACTCACCTGCCGAATCAACTAGCCCCGAAAATGGATGGCGCTTAAGCGCGCGACCTATACC	23 (0.000435%)	
TGGTCGATTAAGACAGCAGGACGGTGGTCATGGAAGTCGAAATCCGCTAAGGAGTGTGTAACAACTCACCTGCCGAATCAACTAGCCCCGAAAATGGATG	9 (0.000170%)	
TGGTCTTCAACGAGGAATTCCTAGTAAGCGCGAGTCATCAGCTCGCGTTGACTACGTCCCTGCCCTTTGTACACACCGCCCGTCGCTCCTACCGATTGAA	37 (0.000700%)	
TGGTGATATGAACACAAACGTTCAATATGACAAACCCATGCCAAGTAAAGAGAAAATGAAAACTGGTGATTGTTGCGGAAATCGTCCAGGATTCCTCGAC	3 (0.000057%)	
TGGTGCCAGCAGCCGCGGTAATTCCAGCTCCAATAGCGTATATTTAAGTTGTTGCAGTTAAAAAGCTCGTAGTTGAACCTTGGGATGGGTCGGCCGGTCC	5 (0.000095%)	
TGGTTCGCCGCCCGCGACGTCGCGAGAAGTCCACTAAACCTTATCATTTAGAGGAAGGAGAAGTCGTAACAAGGTTTCCGTAGGTGAACCTGCGGAAGGA	6 (0.000114%)	
TGTAAAGTTGGGAATTCGTTAAGGAGCTGTTGCTTTGTTAGTGTAGAAACACTTGTGTAGAATTGGGGATTGTTTTTTTTGGAGTGATTTAGGGGAGGGT	15 (0.000284%)	
TGTACGGCTTTGGCTCGGATTCGTCCGTCTTCTTTCTTCTTAGCCGAGTACTTCGGTAGATTAGTTGGAACGATTGATGATTTTGAGTTAATTGAACGTT	3 (0.000057%)	
TGTAGGGAAAGAGTGTTCAGAGCCGTGTAGATCTCGGTGG	7 (0.000053%)	
TGTATCCAGAGCGTAGGCTTGCTTTGAGCACTCTAATTTCTTCAAAGTAACAGCGCCGGAGGCACGACCCGGCCAATTAAGACCAGGAGCGTATCGCCGA	27 (0.000511%)	
TGTATCCTTGTTAGAAGACACAAAGCCAAAGACTCATATGGACTTTGGCTACACCATGAAAGCTTTGAGAAGCAAGAAGAAGGTTGGTTAGTGTTTTGGA	680 (0.012864%)	
TGTCAGGTGGGGAGTTTGGCTGGGGCGGCACATCTGTTAAAAGATAACGCAGGTGTCCTAAGATGAGCTCAACGAGAACAGAAATCTCGTGTGGAACAAA	49 (0.000927%)	
TGTCCCGAAGGTATCTCGCGCTTGTACGGCTTTGGCTCGGATTCGTCCGTCTTCTTTCTTCTTAGCCGAGTACTTCGGTAGATTAGTTGGAACGATTGAT	4 (0.000076%)	
TGTCCCGAGTGTGAGCGAGGTGTGAGTGTCGCCCATGGGCATCGACACCTTGCGGCTAGGAACTGGAACGAGACGGGTGGCAAAGATTTCGAGTAGCACT	3 (0.000057%)	
TGTCGCCCATGGGCATCGACACCTTGCGGCTAGGAACTGGAACGAGACGGGTGGCAAAGATTTCGAGTAGCACTTCATACTACCGTGGGTTTTTTAAACC	7 (0.000132%)	
TGTCTCAAAGATTAAGCCATGCATGTGTAAGTATGAACGAATTCAGACTGTGAAACTGCGAATGGCTCATTAAATCAGTTATAGTTTGTTTGATGGTAAC	3 (0.000057%)	
TGTGAGCGAGGTGTGAGTGTCGCCCATGGGCATCGACACCTTGCGGCTAGGAACTGGAACGAGACGGGTGGCAAAGATTTCGAGTAGCACTTCATACTAC	5 (0.000095%)	
TGTGAGTGTCGCCCATGGGCATCGACACCTTGCGGCTAGGAACTGGAACGAGACGGGTAGCAAAGATTTCGAGTAGCACTTCATACTACCGTGGGTTTTT	7 (0.000132%)	
TGTGAGTGTCGCCCATGGGCATCGACACCTTGCGGCTAGGAACTGGAACGAGACGGGTGGCAAAGATTTCGAGTAGCACTTCATACTACCGTGGGTTTTT	6 (0.000114%)	
TGTGCTGGCGACGCATCATTCAAATTTCTGCCCTATCAACTTTCGATGGTAGGATAGTGGCCTACCATGGTGGTAACGGGTGACGGAGAATTAGGGTTCG	10 (0.000189%)	
TGTTAGAAGACACAAAGCCAAAGACTCATATGGACTTTGGCTACACCATGAAAGCTTTGAGAAGCAAGAAGAAGGTTGGTTAGTGTTTTGGAGTCGAATA	29 (0.000549%)	
TGTTATACTCAATCATACACATGACAACAAGTCATATTCGACTCCAAAACACTAACCAACCTTCTTCTTGCTTCTCAAAGCTTTCATGGTGTAGCCAAAG	6 (0.000114%)	
TGTTATCCCATGCTAATGTATCCAGAGCGTAGGCTTGCTTTGAGCACTCTAATTTCTTCAAAGTAACAGCGCCGGAGGCACGACCCGGCCAATTAAGACC	10 (0.000189%)	
TGTTCCATCGACCAGAGGCTGTTCACCTTGGAGACCTGATGCGGTTATGAGTACGACCGGGCGTGAGCGGCACTCGGTCCTCCGGATTTTCAAGGGCCGC	5 (0.000095%)	
TGTTGCTTTGTTAGTGTAGAAACACTTGTGTAGAATTGGGGATTGTTTTTTTTGGAGTGATTTAGGGGAGGGTCGAATCTTAGCGACAAAGGGCTGAATC	16 (0.000303%)	
TGTTGGTCGATTAAGACAGCAGGACGGTGGTCATGGAAGTCGAAATCCGCTAAGGAGTGTGTAACAACTCACCTGCCGAATCAACTAGCCCCGAAAATGG	13 (0.000246%)	
TGTTGGTCTTCAACGAGGAATTCCTAGTAAGCGCGAGTCATCAGCTCGCGTTGACTACGTCCCTGCCCTTTGTACACACCGCCCGTCGCTCCTACCGATT	193 (0.003651%)	
TGTTTTGGAGTCGAATATGACTTGATGTCATGTGTATGAT	272 (0.002058%)	
TTAAAAGATAACGCAGGTGTCCTAAGATGAGCTCAACGAGAACAGAAATCTCGTGTGGAACAAAAGGGTAAAAGCTCGTTTGATTCTGATTTTCAGTACG	20 (0.000378%)	
TTAAAGGCGTAAGAATTGTATCCTTGTTAGAAGACACAAA	465 (0.003519%)	

TTAACAGGGACAGTCGGGGGCATTCGTATTTCATAGTCAGAGGTGAAATTCTTGGATTTATGAAAGACGAA<br>CAACTGCGAAAGCATTTGCCAAGGATGTT	189 (0.003576%)	
TTAAGACAGCAGGACGGTGGTCATGGAAGTCGAAATCCGCTAAGGAGTGTGTAACAACTCACCTGCCGAAT<br>CAACTAGCCCCGAAAATGGATGGCGCTTA	78 (0.001476%)	
TTAAGGAGCTGTTGCTTTGTTAGTGTAGAAACACTTGTGTAGAATTGGGGATTGTTTTTTTTGGAGTGATT<br>TAGGGGAGGGTCGAATCTTAGCGACAAAG	18 (0.000341%)	
TTAAGTTCTTATACTCAATCATACACATGACATCAAGTCATATTCGACTCCAAAACACTAACCAACCTTCT<br>TCTTGCTTCTCAAAGCTTTCATGGTGTAG	176 (0.003330%)	
TTAAGTTGTTATACTCAATCATACACATGACAACAAGTCATATTCGACTCCAAAACACTAACCAACCTTCT<br>TCTTGCTTCTCAAAGCTTTCATGGTGTAG	61 (0.001154%)	
TTACATTTTATCGGTCGCTCTTGTCCGGAAGCTGTAGATGACCCAAAGTCCATATAGCGACCCCAGGTCAG<br>GCGGGATTACCCGCTGAGTTTAAGCATAT	12 (0.000227%)	
TTACGGGTTTACTCACCCGTTGACTCGCACACATGTCAGACTCCTTGGTCCGTGTTTCAAGACGGGTCGAA<br>TGGGGAGCCCACAGGCCGACGCCCGGAGC	30 (0.000568%)	
TTACTCACCCGTTGACTCGCACACATGTCAGACTCCTTGGTCCGTGTTTCAAGACGGGTCGAATGGGGAGC<br>CCACAGGCCGACGCCCGGAGCACGCTGAT	11 (0.000208%)	
TTAGAAGACACAAAGCCAAAGACTCATATGGACTTTGGCTACACCATGAAAGCTTTGAGAAGCAAGAAGAA<br>GGTTGGTTAGTGTTTTGGAGTCGAATATG	92 (0.001740%)	
TTAGGTACGAAACACAGGCCCCGGAACTCATCATCGAGCGTAACATCGCCCGTGAATTAACTGAGAAGGAT<br>AGGTGGTAGGTAGTTCGATGCGCGAGCAT	9 (0.000170%)	
TTAGTGTTTTGGAGTCGAATATGACTTGATGTCATGTGTA	357 (0.002702%)	
TTATACTCAATCATACACATGACAACAAGTCATATTCGACTCCAAAACACTAACCAACCTTCTTCTTGCTT<br>CTCAAAGCTTTCATGGTGTAGCCAAAGTC	318 (0.006016%)	
TTATACTCAATCATACACATGACATCAAGTCATATTCGACTCCAAAACACTAACCAACCTTCTTCTTGCTT<br>CTCAAAGCTTTCATGGTGTAGCCAAAGTC	684 (0.012940%)	
TTATCATGAATCATCAGAGCAACGGGCAGAGCCCGCGTCGACCTTTTATCTAATAAATGCGTCCCTTCCAT<br>AAGTCGGGGTTTGTTGCACGTATTAGCTC	67 (0.001268%)	
TTATCCCATGCTAATGTATCCAGAGCGTAGGCTTGCTTTGAGCACTCTAATTTCTTCAAAGTAACAGCGCC<br>GGAGGCACGACCCGGCCAATTAAGACCAG	5 (0.000095%)	
TTATCGGTCGCTCTTGTCCGGAAGCTGTAGATGACCCAAAGTCCATATAGCGACCCCAGGTCAGGCGGGAT<br>TACCCGCTGAGTTTAAGCATATCAATAAG	8 (0.000151%)	
TTCAAATTAGGTACGAAACACAGGCCCCGGAACTCATCATCGAGCGTAACATCGCCCGTGAATTAACTGAG<br>AAGGATAGGTGGTAGGTAGTTCGATGCGC	38 (0.000719%)	
TTCAAATTTCTGCCCTATCAACTTTCGATGGTAGGATAGTGGCCTACCATGGTGGTAACGGGTGACGGAGA<br>ATTAGGGTTCGATTCCGGAGAGGGAGCCT	26 (0.000492%)	
TTCAACGAGGAATTCCTAGTAAGCGCGAGTCATCAGCTCGCGTTGACTACGTCCCTGCCCTTTGTACACAC<br>CGCCCGTCGCTCCTACCGATTGAATGATC	5 (0.000095%)	
TTCAAGGGCCGCCGGGGGCGCACCGGACACCACGCGACGTGCGGTGCTCTTCCAGCCGCTGGACCCTACCT<br>CCGGCTGAGCCGTTTCCAGGGTGGGCAGG	14 (0.000265%)	
TTCAATATGACAAACCCATGCCAAGTAAAGAGAAAATGAAAACTGGTGATTGTTGCGGAAATCGTCCAGGA<br>TTCCTCGACCAGGACTTGAAATCGTCGAG	3 (0.000057%)	
TTCAATCGGTAGGAGCGACGGGCGGTGTGTACAAAGGGCAGGGACGTAGTCAACGCGAGCTGATGACTCGC<br>GCTTACTAGGAATTCCTCGTTGAAGACCA	51 (0.000965%)	
TTCAATGTCTGTTGGTGCCAAGAGGGAAAAGGGCTATTAAGCTATATAGGGGGGTGGGTGTTGAGGGAGTC<br>TGGGCAGTCCGTGGGGAACCCCCTTTTTC	459 (0.008683%)	
TTCACATTTCGTTCATCACCCTTGGCCGGCTATCGAACAGCCGGACTCCCATCAAAAGATGGTTGCCAAGA<br>ACATCTTCGTTACGGTTTGCTAATTCTCG	18 (0.000341%)	
TTCACATTTCGTTCATCACCCTTGGCCGGCTTTCGAACAGCCGGACTCCCATCAAAAGATGGTTGCCAAGA<br>ACATCTTCGTTACGGTTTGCTAATTCTCG	14 (0.000265%)	
TTCACGGTTCGTATTCGTACTGAAAATCAGAATCAAACGAGCTTTTACCCTTTTGTTCCACACGAGATTTC<br>TGTTCTCGTTGAGCTCATCTTAGGACACC	17 (0.000322%)	
TTCCAACGAAGCACGCCCATCCAACCTAGGCGAGACAAGGGTTCACATTTCGTTCATCACCCTTGGCCGGC<br>TATCGAACAGCCGGACTCCCATCAAAAGA	106 (0.002005%)	
TTCCAGCTCCAATAGCGTATATTTAAGTTGTTGCAGTTAAAAAGCTCGTAGTTGAACCTTGGGATGGGTCG<br>GCCGGTCCGCCTTTGGTGTGCATTGGTCG	5 (0.000095%)	
TTCCCTTGCCTACATTGTTCCATCGACCAGAGGCTGTTCACCTTGGAGACCTGATGCGGTTATGAGTACGA<br>CCGGGCGTGAGCGGCACTCGGTCCTCCGG	6 (0.000114%)	
TTCCGGAACCGGGACGTGGCGGTTGACGGCAACGTTAGGGAGTCCGGAGACGTCGGCGGGGGCCTCGGGAA<br>GAGTTATCTTTTCTGTTTAACAGCCTGCC	2316 (0.043815%)	
TTCCTTGCGCCTTACGGGTTTACTCACCCGTTGACTCGCACACATGTCAGACTCCTTGGTCCGTGTTTCAA<br>GACGGGTCGAATGGGGAGCCCACAGGCCG	11 (0.000208%)	
TTCGACTCCAAAACACTAACCAACCTTCTTCTTGCTTCTCAAAGCTTTCATGGTGTAGCCAAAGTCCATAT<br>GAGTCTTTGGCTTTGTGTCTTCTAACAAG	120 (0.002270%)	
TTCGACTCCAAAACACTAACCAACCTTCTTCTTGCTTCTCAAAGCTTTCATGGTGTAGCCAAAGTCCATAT<br>GAGTCTTTGGCTTTGTGTCTTTTAACAAG	74 (0.001400%)	
TTCGAGTGTGAGCATGCCTGTCGGGACCCGAAAGATGGTGAACTATGCCTGAGCGGGGTAAAGCCAGAGGA<br>AACTCTGGTGGAAGCCCGCAGCGATACTG	5 (0.000095%)	
TTCGATTAGTCTTTCGCCCCTATACCCAAGTCAGACGAACGATTTGCACGTCAGTATCGCTGCGGGCTTCC<br>ACCAGAGTTTCCTCTGGCTTTACCCCGCT	10 (0.000189%)	
TTCGCCGCCCGCGACGTCGCGAGAAGTCCACTAAACCTTATCATTTAGAGGAAGGAGAAGTCGTAACAAGG<br>TTTCCGTAGGTGAACCTGCGGAAGGATCG	5 (0.000095%)	
TTCGTACTGAAAATCAGAATCAAACGAGCTTTTACCCTTTTGTTCCACACGAGATTTCTGTTCTCGTTGAG<br>CTCATCTTAGGACACCTGCGTTATCTTTT	20 (0.000378%)	
TTCGTAGGCTCCATGCTCGCGCATCGAACTACCTACCACCTATCCTTCTCAGTTAATTCACGGGCGATGTT<br>ACGCTCGATGATGAGTTCCGGGGCCTGTG	11 (0.000208%)	
TTCGTATTCGTACTGAAAATCAGAATCAAACGAGCTTTTACCCTTTTGTTCCACACGAGATTTCTGTTCTC<br>GTTGAGCTCATCTTAGGACACCTGCGTTA	12 (0.000227%)	
TTCGTATTTCATAGTCAGAGGTGAAATTCTTGGATTTATGAAAGACGAACAACTGCGAAAGCATTTGCCAA<br>GGATGTTTTCATTAATCAAGAACGAAAGT	24 (0.000454%)	
TTCGTTCATCACCCTTGGCCGGCTATCGAACAGCCGGACTCCCATCAAAAGATGGTTGCCAAGAACATCTT<br>CGTTACGGTTTGCTAATTCTCGGAATAAC	18 (0.000341%)	
TTCGTTCATCACCCTTGGCCGGCTTTCGAACAGCCGGACTCCCATCAAAAGATGGTTGCCAAGAACATCTT<br>CGTTACGGTTTGCTAATTCTCGGAATAAC	12 (0.000227%)	
TTCTGAGAAGGGTTCGAGTGTGAGCATGCCTGTCGGGACCCGAAAGATGGTGAACTATGCCTGAGCGGGGT<br>AAAGCCAGAGGAAACTCTGGTGGAAGCCC	9 (0.000170%)	

TTCTGCCCTATCAACTTTCGATGGTAGGATAGTGGCCTACCATGGTGGTAACGGGTGACGGAGAATTAGGGTTCGATTCCGGAGAGGGAGCCTGAGAAAC	11 (0.000208%)	
TTCTTATACTCAATCATACACATGACATCAAGTCATATTCGACTCCAAAACACTAACCAACCTTCTTCTTGCTTCTCAAAGCTTTCATGGTGTAGCCAAA	99 (0.001873%)	
TTGAAATCGTCGACCAGGTCCGAGACTTCATCGACCGGGTCCGAGGATTCGTCGACCAGGACGGCCGGATGTCCGAGAAAAAAAAATGTTGCCGAATAAC	47 (0.000889%)	
TTGACGGCAACGTTAGGGAGTCCGGAGACGTCGGCGGGGGCCTCGGGAAGAGTTATCTTTTCTGTTTAACAGCCTGCCCACCCTGGAAACGGCTCAGCCG	6 (0.000114%)	
TTGACTCGCACACATGTCAGACTCCTTGGTCCGTGTTTCAAGACGGGTCGAATGGGGAGCCCACAGGCCGACGCCCGGAGCACGCTGATGCCGAGGCACG	6 (0.000114%)	
TTGAGTATAAGAACTTAAACCGCAACCGCATCTTATAAGCCTAAGTAGTGTTTCCTTGTTAGAAGACACAAAGCCAAAGACTCATATGGACTTTGGCTAC	7 (0.000132%)	
TTGCCTACATTGTTCCATCGACCAGAGGCTGTTCACCTTGGAGACCTGATGCGGTTATGAGTACGACCGGGCGTGAGCGGCACTCGGTCCTCCGGATTTT	6 (0.000114%)	
TTGCGCCTTACGGGTTTACTCACCCGTTGACTCGCACACATGTCAGACTCCTTGGTCCGTGTTTCAAGACGGGTCGAATGGGGAGCCCACAGGCCGACGC	22 (0.000416%)	
TTGCGGTTTAAGTTCTTATACTCAATCATACACATGACATCAAGTCATATTCGACTCCAAAACACTAACCAACCTTCTTCTTGCTTCTCAAAGCTTTCAT	449 (0.008494%)	
TTGCGGTTTAAGTTGTTATACTCAATCATACACATGACAACAAGTCATATTCGACTCCAAAACACTAACCAACCTTCTTCTTGCTTCTCAAAGCTTTCAT	79 (0.001495%)	
TTGCTGATGCGGGACGGAAGCTGGTCTCCCGTGTGTTACCGCACGCGGTTGGCCTAAATCCGAGCCAAGGACGCCTGGAGCGTACCGACATGCGGTGGTG	3 (0.000057%)	
TTGGAGACCTGATGCGGTTATGAGTACGACCGGGCGTGAGCGGCACTCGGTCCTCCGGATTTTCAAGGGCCGCCGGGGGCGCACCGGACACCACGCGACG	25 (0.000473%)	
TTGGAGGGCAAGTCTGGTGCCAGCAGCCGCGGTAATTCCAGCTCCAATAGCGTATATTTAAGTTGTTGCAGTTAAAAAGCTCGTAGTTGAACCTTGGGAT	338 (0.006394%)	
TTGGAGTCGAATATGACTTGATGTCATGTGTATGATTGAG	284 (0.002149%)	
TTGGCTCGGATTCGTCCGTCTTCTTTCTTCTTAGCCGAGTACTTCGGTAGATTAGTTGGAACGATTGATGATTTTGAGTTAATTGAACGTTCGGCGTATG	5 (0.000095%)	
TTGGCTGGGGCGGCACATCTGTTAAAAGATAACGCAGGTGTCCTAAGATGAGCTCAACGAGAACAGAAATCTCGTGTGGAACAAAAGGGTAAAAGCTCGT	25 (0.000473%)	
TTGGTCGATTAAGACAGCAGGACGGTGGTCATGGAAGTCGAAATCCGCTAAGGAGTGTGTAACAACTCACCTGCCGAATCAACTAGCCCCGAAAATGGAT	6 (0.000114%)	
TTGGTCTTCAACGAGGAATTCCTAGTAAGCGCGAGTCATCAGCTCGCGTTGACTACGTCCCTGCCCTTTGTACACACCGCCCGTCGCTCCTACCGATTGA	104 (0.001967%)	
TTGGTGATATGAACACAAACGTTCAATATGACAAACCCATGCCAAGTAAAGAGAAAATGAAAACTGGTGATTGTTGCGGAAATCGTCCAGGATTCCTCGA	3 (0.000057%)	
TTGTACGGCTTTGGCTCGGATTCGTCCGTCTTCTTTCTTCTTAGCCGAGTACTTCGGTAGATTAGTTGGAACGATTGATGATTTTGAGTTAATTGAACGT	10 (0.000189%)	
TTGTATCCTTGTTAGAAGACACAAAGCCAAAGACTCATATGGACTTTGGCTACACCATGAAAGCTTTGAGAAGCAAGAAGAAGGTTGGTTAGTGTTTTGG	14 (0.000265%)	
TTGTCAGGTGGGGAGTTTGGCTGGGGCGGCACATCTGTTAAAAGATAACGCAGGTGTCCTAAGATGAGCTCAACGAGAACAGAAATCTCGTGTGGAACAA	11 (0.000208%)	
TTGTCTCAAAGATTAAGCCATGCATGTGTAAGTATGAACGAATTCAGACTGTGAAACTGCGAATGGCTCATTAAATCAGTTATAGTTTGTTTGATGGTAA	10 (0.000189%)	
TTGTTAGAAGACACAAAGCCAAAGACTCATATGGACTTTGGCTACACCATGAAAGCTTTGAGAAGCAAGAAGAAGGTTGGTTAGTGTTTTGGAGTCGAAT	56 (0.001059%)	
TTGTTATACTCAATCATACACATGACAACAAGTCATATTCGACTCCAAAACACTAACCAACCTTCTTCTTGCTTCTCAAAGCTTTCATGGTGTAGCCAAA	45 (0.000851%)	
TTGTTGGTCTTCAACGAGGAATTCCTAGTAAGCGCGAGTCATCAGCTCGCGTTGACTACGTCCCTGCCCTTTGTACACACCGCCCGTCGCTCCTACCGAT	180 (0.003405%)	
TTTAAGTTCTTATACTCAATCATACACATGACATCAAGTCATATTCGACTCCAAAACACTAACCAACCTTCTTCTTGCTTCTCAAAGCTTTCATGGTGTA	113 (0.002138%)	
TTTAAGTTGTTATACTCAATCATACACATGACAACAAGTCATATTCGACTCCAAAACACTAACCAACCTTCTTCTTGCTTCTCAAAGCTTTCATGGTGTA	53 (0.001003%)	
TTTAATTAAACAGTCGGATTCCCCTTGTCCGTACCAGTTCTGAGCTGACTGTTCGACGCCCGGGGAAAGCTCCCGAGAGAGCCGTTCCCAGTCCGTCCCC	17 (0.000322%)	
TTTACTCACCCGTTGACTCGCACACATGTCAGACTCCTTGGTCCGTGTTTCAAGACGGGTCGAATGGGGAGCCCACAGGCCGACGCCCGGAGCACGCTGA	13 (0.000246%)	
TTTATCGGTCGCTCTTGTCCGGAAGCTGTAGATGACCCAAAGTCCATATAGCGACCCCAGGTCAGGCGGGATTACCCGCTGAGTTTAAGCATATCAATAA	15 (0.000284%)	
TTTCAAGGGCCGCCGGGGGCGCACCGGACACCACGCGACGTGCGGTGCTCTTCCAGCCGCTGGACCCTACCTCCGGCTGAGCCGTTTCCAGGGTGGGCAG	29 (0.000549%)	
TTTCACGGTTCGTATTCGTACTGAAAATCAGAATCAAACGAGCTTTTACCCTTTTGTTCCACACGAGATTTCTGTTCTCGTTGAGCTCATCTTAGGACAC	54 (0.001022%)	
TTTCAGCAGTTCTCGGACAAAAATTGCTGAGTGGCCGAGAAGAATGGGCGTGTCATGCGTGGGCTGACATGGATTCTTCGAGGCCTAGGGGTGGCGGTAT	10 (0.000189%)	
TTTCCAACGAAGCACGCCCATCCAACCTAGGCGAGACAAGGGTTCACATTTCGTTCATCACCCTTGGCCGGCTATCGAACAGCCGGACTCCCATCAAAAG	1826 (0.034545%)	
TTTCGTTCATCACCCTTGGCCGGCTATCGAACAGCCGGACTCCCATCAAAAGATGGTTGCCAAGAACATCTTCGTTACGGTTTGCTAATTCTCGGAATAA	9 (0.000170%)	
TTTCGTTCATCACCCTTGGCCGGCTTTCGAACAGCCGGACTCCCATCAAAAGATGGTTGCCAAGAACATCTTCGTTACGGTTTGCTAATTCTCGGAATAA	9 (0.000170%)	
TTTCTGCCCTATCAACTTTCGATGGTAGGATAGTGGCCTACCATGGTGGTAACGGGTGACGGAGAATTAGGGTTCGATTCCGGAGAGGGAGCCTGAGAAA	14 (0.000265%)	
TTTGCTGATGCGGGACGGAAGCTGGTCTCCCGTGTGTTACCGCACGCGGTTGGCCTAAATCCGAGCCAAGGACGCCTGGAGCGTACCGACATGCGGTGGT	27 (0.000511%)	
TTTGGAGTCGAATATGACTTGATGTCATGTGTATGATTGA	368 (0.002785%)	
TTTGGCTCGGATTCGTCCGTCTTCTTTCTTCTTAGCCGAGTACTTCGGTAGATTAGTTGGAACGATTGATGATTTTGAGTTAATTGAACGTTCGGCGTAT	27 (0.000511%)	
TTTGGCTGGGGCGGCACATCTGTTAAAAGATAACGCAGGTGTCCTAAGATGAGCTCAACGAGAACAGAAATCTCGTGTGGAACAAAAGGGTAAAAGCTCG	20 (0.000378%)	
TTTGGCTTTGTGTCTTCTAACAAGGAAACACTACTTAGGC	6916 (0.052335%)	

TTTGTTTTAATTAAACAGTCGGATTCCCCTTGTCCGTACCAGTTCTGAGCTGACTGTTCGACGCCCGGGGAAAGCTCCCGAGAGAGCCGTTCCCAGTCCG	21 (0.000397%)	
TTTTAATTAAACAGTCGGATTCCCCTTGTCCGTACCAGTTCTGAGCTGACTGTTCGACGCCCGGGGAAAGCTCCCGAGAGAGCCGTTCCCAGTCCGTCCC	17 (0.000322%)	
TTTTATCGGTCGCTCTTGTCCGGAAGCTGTAGATGACCCAAAGTCCATATAGCGACCCCAGGTCAGGCGGGATTACCCGCTGAGTTTAAGCATATCAATA	3 (0.000057%)	
TTTTCAAGGGCCGCCGGGGGCGCACCGGACACCACGCGACGTGCGGTGCTCTTCCAGCCGCTGGACCCTACCTCCGGCTGAGCCGTTTCCAGGGTGGGCA	24 (0.000454%)	
TTTTCAGCAGTTCTCGGACAAAAATTGCTGAGTGGCCGAGAAGAATGGGCGTGTCATGCGTGGGCTGACATGGATTCTTCGAGGCCTAGGGGTGGCGGTA	11 (0.000208%)	
TTTTGGAGTCGAATATGACTTGATGTCATGTGTATGATTG	535 (0.004049%)	
TTTTTCAGCAGTTCTCGGACAAAAATTGCTGAGTGGCCGAGAAGAATGGGCGTGTCATGCGTGGGCTGACATGGATTCTTCGAGGCCTAGGGGTGGCGGT	31 (0.000586%)	
TTTTTTTTTT	54385 (0.102887%)	

After filtering

After filtering: read1: quality

Value of each position will be shown on mouse over.

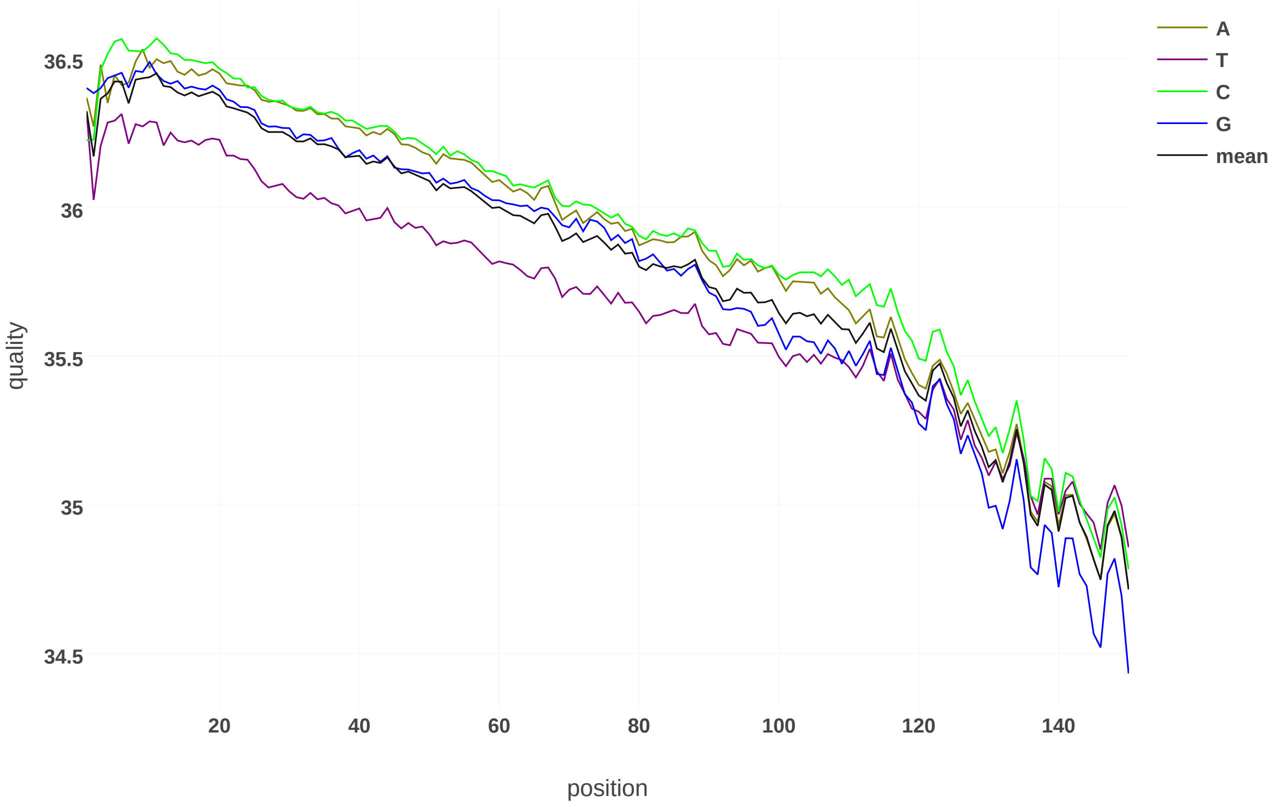

After filtering: read1: base contents

Value of each position will be shown on mouse over.

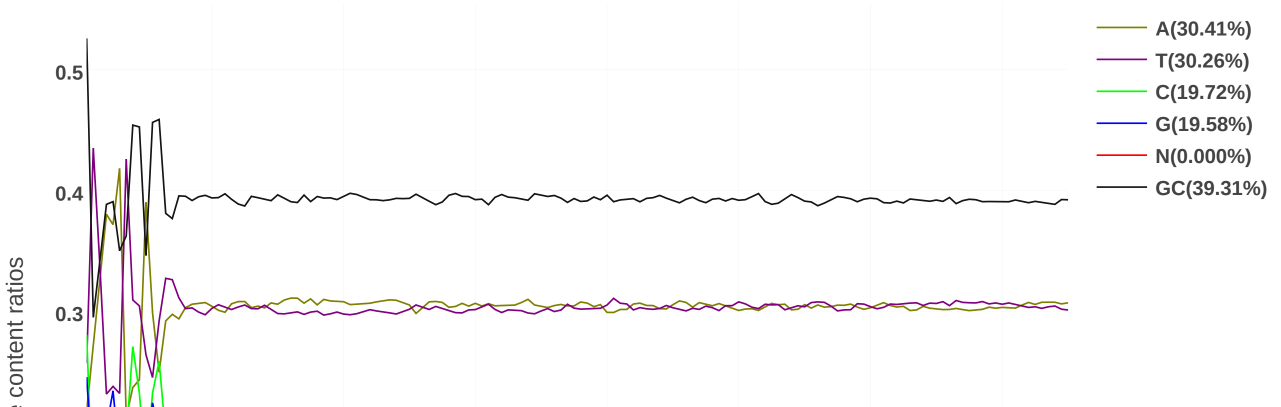

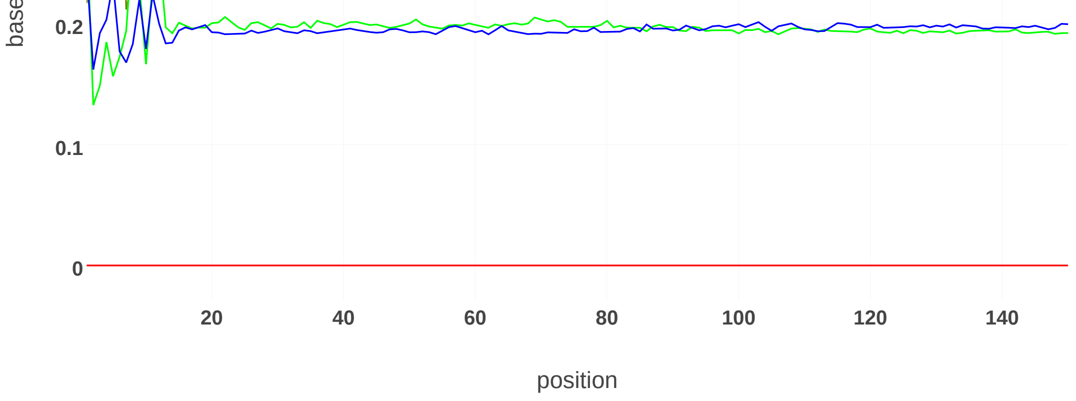

After filtering: read1: KMER counting

Darker background means larger counts. The count will be shown on mouse over.

	AA	AT	AC	AG	TA	TT	TC	TG	CA	CT	CC	CG	GA	GT	GC	GG
AAA	AAAAA	AAAAT	AAAAC	AAAAG	AAATA	AAATT	AAATC	AAATG	AAACA	AAACT	AAACC	AAACG	AAAGA	AAAGT	AAAGC	AAAGG
AAT	AATAA	AATAT	AATAC	AATAG	AATTA	AATTT	AATTC	AATTG	AATCA	AATCT	AATCC	AATCG	AATGA	AATGT	AATGC	AATGG
AAC	AACAA	AACAT	AACAC	AACAG	AACTA	AACTT	AACTC	AACTG	AACCA	AACCT	AACCC	AACCG	AACGA	AACGT	AACGC	AACGG
AAG	AAGAA	AAGAT	AAGAC	AAGAG	AAGTA	AAGTT	AAGTC	AAGTG	AAGCA	AAGCT	AAGCC	AAGCG	AAGGA	AAGGT	AAGGC	AAGGG
ATA	ATAAA	ATAAT	ATAAC	ATAAG	ATATA	ATATT	ATATC	ATATG	ATACA	ATACT	ATACC	ATACG	ATAGA	ATAGT	ATAGC	ATAGG
ATT	ATTAA	ATTAT	ATTAC	ATTAG	ATTTA	ATTTT	ATTTC	ATTTG	ATTCA	ATTCT	ATTCC	ATTCG	ATTGA	ATTGT	ATTGC	ATTGG
ATC	ATCAA	ATCAT	ATCAC	ATCAG	ATCTA	ATCTT	ATCTC	ATCTG	ATCCA	ATCCT	ATCCC	ATCCG	ATCGA	ATCGT	ATCGC	ATCGG
ATG	ATGAA	ATGAT	ATGAC	ATGAG	ATGTA	ATGTT	ATGTC	ATGTG	ATGCA	ATGCT	ATGCC	ATGCG	ATGGA	ATGGT	ATGGC	ATGGG
ACA	ACAAA	ACAAT	ACAAC	ACAAG	ACATA	ACATT	ACATC	ACATG	ACACA	ACACT	ACACC	ACACG	ACAGA	ACAGT	ACAGC	ACAGG
ACT	ACTAA	ACTAT	ACTAC	ACTAG	ACTTA	ACTTT	ACTTC	ACTTG	ACTCA	ACTCT	ACTCC	ACTCG	ACTGA	ACTGT	ACTGC	ACTGG
ACC	ACCAA	ACCAT	ACCAC	ACCAG	ACCTA	ACCTT	ACCTC	ACCTG	ACCCA	ACCCT	ACCCC	ACCCG	ACCGA	ACCGT	ACCGC	ACCGG
ACG	ACGAA	ACGAT	ACGAC	ACGAG	ACGTA	ACGTT	ACGTC	ACGTG	ACGCA	ACGCT	ACGCC	ACGCG	ACGGA	ACGGT	ACGGC	ACGGG
AGA	AGAAA	AGAAT	AGAAC	AGAAG	AGATA	AGATT	AGATC	AGATG	AGACA	AGACT	AGACC	AGACG	AGAGA	AGAGT	AGAGC	AGAGG
AGT	AGTAA	AGTAT	AGTAC	AGTAG	AGTTA	AGTTT	AGTTC	AGTTG	AGTCA	AGTCT	AGTCC	AGTCG	AGTGA	AGTGT	AGTGC	AGTGG
AGC	AGCAA	AGCAT	AGCAC	AGCAG	AGCTA	AGCTT	AGCTC	AGCTG	AGCCA	AGCCT	AGCCC	AGCCG	AGCGA	AGCGT	AGCGC	AGCGG
AGG	AGGAA	AGGAT	AGGAC	AGGAG	AGGTA	AGGTT	AGGTC	AGGTG	AGGCA	AGGCT	AGGCC	AGGCG	AGGGA	AGGGT	AGGGC	AGGGG
TAA	TAAAA	TAAAT	TAAAC	TAAAG	TAATA	TAATT	TAATC	TAATG	TAACA	TAACT	TAACC	TAACG	TAAGA	TAAGT	TAAGC	TAAGG
TAT	TATAA	TATAT	TATAC	TATAG	TATTA	TATTT	TATTC	TATTG	TATCA	TATCT	TATCC	TATCG	TATGA	TATGT	TATGC	TATGG
TAC	TACAA	TACAT	TACAC	TACAG	TACTA	TACTT	TACTC	TACTG	TACCA	TACCT	TACCC	TACCG	TACGA	TACGT	TACGC	TACGG
TAG	TAGAA	TAGAT	TAGAC	TAGAG	TAGTA	TAGTT	TAGTC	TAGTG	TAGCA	TAGCT	TAGCC	TAGCG	TAGGA	TAGGT	TAGGC	TAGGG
TTA	TTAAA	TTAAT	TTAAC	TTAAG	TTATA	TTATT	TTATC	TTATG	TTACA	TTACT	TTACC	TTACG	TTAGA	TTAGT	TTAGC	TTAGG
TTT	TTTAA	TTTAT	TTTAC	TTTAG	TTTTA	TTTTT	TTTTC	TTTTG	TTTCA	TTTCT	TTTCC	TTTCG	TTTGA	TTTGT	TTTGC	TTTGG
TTC	TTCAA	TTCAT	TTCAC	TTCAG	TTCTA	TTCTT	TTCTC	TTCTG	TTCCA	TTCCT	TTCCC	TTCCG	TTCGA	TTCGT	TTCGC	TTCGG
TTG	TTGAA	TTGAT	TTGAC	TTGAG	TTGTA	TTGTT	TTGTC	TTGTG	TTGCA	TTGCT	TTGCC	TTGCG	TTGGA	TTGGT	TTGGC	TTGGG
TCA	TCAAA	TCAAT	TCAAC	TCAAG	TCATA	TCATT	TCATC	TCATG	TCACA	TCACT	TCACC	TCACG	TCAGA	TCAGT	TCAGC	TCAGG
TCT	TCTAA	TCTAT	TCTAC	TCTAG	TCTTA	TCTTT	TCTTC	TCTTG	TCTCA	TCTCT	TCTCC	TCTCG	TCTGA	TCTGT	TCTGC	TCTGG
TCC	TCCAA	TCCAT	TCCAC	TCCAG	TCCTA	TCCTT	TCCTC	TCCTG	TCCCA	TCCCT	TCCCC	TCCCG	TCCGA	TCCGT	TCCGC	TCCGG
TCG	TCGAA	TCGAT	TCGAC	TCGAG	TCGTA	TCGTT	TCGTC	TCGTG	TCGCA	TCGCT	TCGCC	TCGCG	TCGGA	TCGGT	TCGGC	TCGGG
TGA	TGAAA	TGAAT	TGAAC	TGAAG	TGATA	TGATT	TGATC	TGATG	TGACA	TGACT	TGACC	TGACG	TGAGA	TGAGT	TGAGC	TGAGG
TGT	TGTAA	TGTAT	TGTAC	TGTAG	TGTTA	TGTTT	TGTTC	TGTTG	TGTCA	TGTCT	TGTCC	TGTCG	TGTGA	TGTGT	TGTGC	TGTGG
TGC	TGCAA	TGCAT	TGCAC	TGCAG	TGCTA	TGCTT	TGCTC	TGCTG	TGCCA	TGCCT	TGCCC	TGCCG	TGCGA	TGCGT	TGCGC	TGCGG
TGG	TGGAA	TGGAT	TGGAC	TGGAG	TGGTA	TGGTT	TGGTC	TGGTG	TGGCA	TGGCT	TGGCC	TGGCG	TGGGA	TGGGT	TGGGC	TGGGG
CAA	CAAAA	CAAAT	CAAAC	CAAAG	CAATA	CAATT	CAATC	CAATG	CAACA	CAACT	CAACC	CAACG	CAAGA	CAAGT	CAAGC	CAAGG
CAT	CATAA	CATAT	CATAC	CATAG	CATTA	CATTT	CATTC	CATTG	CATCA	CATCT	CATCC	CATCG	CATGA	CATGT	CATGC	CATGG
CAC	CACAA	CACAT	CACAC	CACAG	CACTA	CACTT	CACTC	CACTG	CACCA	CACCT	CACCC	CACCG	CACGA	CACGT	CACGC	CACGG
CAG	CAGAA	CAGAT	CAGAC	CAGAG	CAGTA	CAGTT	CAGTC	CAGTG	CAGCA	CAGCT	CAGCC	CAGCG	CAGGA	CAGGT	CAGGC	CAGGG
CTA	CTAAA	CTAAT	CTAAC	CTAAG	CTATA	CTATT	CTATC	CTATG	CTACA	CTACT	CTACC	CTACG	CTAGA	CTAGT	CTAGC	CTAGG
CTT	CTTAA	CTTAT	CTTAC	CTTAG	CTTTA	CTTTT	CTTTC	CTTTG	CTTCA	CTTCT	CTTCC	CTTCG	CTTGA	CTTGT	CTTGC	CTTGG
CTC	CTCAA	CTCAT	CTCAC	CTCAG	CTCTA	CTCTT	CTCTC	CTCTG	CTCCA	CTCCT	CTCCC	CTCCG	CTCGA	CTCGT	CTCGC	CTCGG
CTG	CTGAA	CTGAT	CTGAC	CTGAG	CTGTA	CTGTT	CTGTC	CTGTG	CTGCA	CTGCT	CTGCC	CTGCG	CTGGA	CTGGT	CTGGC	CTGGG
CCA	CCAAA	CCAAT	CCAAC	CCAAG	CCATA	CCATT	CCATC	CCATG	CCACA	CCACT	CCACC	CCACG	CCAGA	CCAGT	CCAGC	CCAGG
CCT	CCTAA	CCTAT	CCTAC	CCTAG	CCTTA	CCTTT	CCTTC	CCTTG	CCTCA	CCTCT	CCTCC	CCTCG	CCTGA	CCTGT	CCTGC	CCTGG
CCC	CCCAA	CCCAT	CCCAC	CCCAG	CCCTA	CCCTT	CCCTC	CCCTG	CCCCA	CCCCT	CCCCC	CCCCG	CCCGA	CCCGT	CCCGC	CCCGG
CCG	CCGAA	CCGAT	CCGAC	CCGAG	CCGTA	CCGTT	CCGTC	CCGTG	CCGCA	CCGCT	CCGCC	CCGCG	CCGGA	CCGGT	CCGGC	CCGGG
CGA	CGAAA	CGAAT	CGAAC	CGAAG	CGATA	CGATT	CGATC	CGATG	CGACA	CGACT	CGACC	CGACG	CGAGA	CGAGT	CGAGC	CGAGG
CGT	CGTAA	CGTAT	CGTAC	CGTAG	CGTTA	CGTTT	CGTTC	CGTTG	CGTCA	CGTCT	CGTCC	CGTCG	CGTGA	CGTGT	CGTGC	CGTGG
CGC	CGCAA	CGCAT	CGCAC	CGCAG	CGCTA	CGCTT	CGCTC	CGCTG	CGCCA	CGCCT	CGCCC	CGCCG	CGCGA	CGCGT	CGCGC	CGCGG
CGG	CGGAA	CGGAT	CGGAC	CGGAG	CGGTA	CGGTT	CGGTC	CGGTG	CGGCA	CGGCT	CGGCC	CGGCG	CGGGA	CGGGT	CGGGC	CGGGG
GAA	GAAAA	GAAAT	GAAAC	GAAAG	GAATA	GAATT	GAATC	GAATG	GAACA	GAACT	GAACC	GAACG	GAAGA	GAAGT	GAAGC	GAAGG
GAT	GATAA	GATAT	GATAC	GATAG	GATTA	GATTT	GATTC	GATTG	GATCA	GATCT	GATCC	GATCG	GATGA	GATGT	GATGC	GATGG
GAC	GACAA	GACAT	GACAC	GACAG	GACTA	GACTT	GACTC	GACTG	GACCA	GACCT	GACCC	GACCG	GACGA	GACGT	GACGC	GACGG
GAG	GAGAA	GAGAT	GAGAC	GAGAG	GAGTA	GAGTT	GAGTC	GAGTG	GAGCA	GAGCT	GAGCC	GAGCG	GAGGA	GAGGT	GAGGC	GAGGG
GTA	GTAAA	GTAAT	GTAAC	GTAAG	GTATA	GTATT	GTATC	GTATG	GTACA	GTACT	GTACC	GTACG	GTAGA	GTAGT	GTAGC	GTAGG
GTT	GTTAA	GTTAT	GTTAC	GTTAG	GTTTA	GTTTT	GTTTC	GTTTG	GTTCA	GTTCT	GTTCC	GTTCG	GTTGA	GTTGT	GTTGC	GTTGG
GTC	GTCAA	GTCAT	GTCAC	GTCAG	GTCTA	GTCTT	GTCTC	GTCTG	GTCCA	GTCCT	GTCCC	GTCCG	GTCGA	GTCGT	GTCGC	GTCGG
GTG	GTGAA	GTGAT	GTGAC	GTGAG	GTGTA	GTGTT	GTGTC	GTGTG	GTGCA	GTGCT	GTGCC	GTGCG	GTGGA	GTGGT	GTGGC	GTGGG
GCA	GCAAA	GCAAT	GCAAC	GCAAG	GCATA	GCATT	GCATC	GCATG	GCACA	GCACT	GCACC	GCACG	GCAGA	GCAGT	GCAGC	GCAGG
GCT	GCTAA	GCTAT	GCTAC	GCTAG	GCTTA	GCTTT	GCTTC	GCTTG	GCTCA	GCTCT	GCTCC	GCTCG	GCTGA	GCTGT	GCTGC	GCTGG
GCC	GCCAA	GCCAT	GCCAC	GCCAG	GCCTA	GCCTT	GCCTC	GCCTG	GCCCA	GCCCT	GCCCC	GCCCG	GCCGA	GCCGT	GCCGC	GCCGG
GCG	GCGAA	GCGAT	GCGAC	GCGAG	GCGTA	GCGTT	GCGTC	GCGTG	GCGCA	GCGCT	GCGCC	GCGCG	GCGGA	GCGGT	GCGGC	GCGGG
GGA	GGAAA	GGAAT	GGAAC	GGAAG	GGATA	GGATT	GGATC	GGATG	GGACA	GGACT	GGACC	GGACG	GGAGA	GGAGT	GGAGC	GGAGG
GGT	GGTAA	GGTAT	GGTAC	GGTAG	GGTTA	GGTTT	GGTTC	GGTTG	GGTCA	GGTCT	GGTCC	GGTCG	GGTGA	GGTGT	GGTGC	GGTGG
GGC	GGCAA	GGCAT	GGCAC	GGCAG	GGCTA	GGCTT	GGCTC	GGCTG	GGCCA	GGCCT	GGCCC	GGCCG	GGCGA	GGCGT	GGCGC	GGCGG
GGG	GGGAA	GGGAT	GGGAC	GGGAG	GGGTA	GGGTT	GGGTC	GGGTG	GGGCA	GGGCT	GGGCC	GGGCG	GGGGA	GGGGT	GGGGC	GGGGG

After filtering: read1: overrepresented sequences

Sampling rate: 1 / 20

overrepresented sequence	count (% of bases)	distribution: cycle 1 ~ cycle 150
AAAAAAAAAA	57742 (0.110660%)	
AAAAAGCAACGTCGCTATGAACGCTTGGCTGCCACAAGCCAGTTATCCCTGTGGTAACTTTTCTGACACCTCTAGCTTCAAATTCCGAAGGTCTAAAGGA	331 (0.006343%)	
AAAAGATAACGCAGGTGTCCTAAGATGAGCTCAACGAGAACAGAAATCTCGTGTGGAACAAAAGGGTAAAAGCTCGTTTGATTCTGATTTTCAGTACGAA	10 (0.000192%)	
AAAAGCAACGTCGCTATGAACGCTTGGCTGCCACAAGCCAGTTATCCCTGTGGTAACTTTTCTGACACCTCTAGCTTCAAATTCCGAAGGTCTAAAGGAT	26 (0.000498%)	
AAAAGCCTAAGTAGTGTTTCCTTGTTAGAAGACACAAAGCCAAAGACTCATATGGACTTTGGCTACACCATGAAAGCTTTGAGAAGCAAGAAGAAGGTTG	22 (0.000422%)	
AAACCGCAACCGGATCTTAAAGGCGTAAGAATTGTATCCTTGTTAGAAGACACAAAGCCAAAGACTCATATGGACTTTGGCTACACCATGAAAGCTTTGA	64 (0.001227%)	
AAACGAGCTTTTACCCTTTTGTTCCACACGAGATTTCTGTTCTCGTTGAGCTCATCTTAGGACACCTGCGTTATCTTTTAACAGATGTGCCGCCCCAGCC	5 (0.000096%)	
AAACTGCGAATGGCTCATTAAATCAGTTATAGTTTGTTTGATGGTAACTACTACTCGGATAACCGTAGTAATTCTAGAGCTAATACGTGCAACAAACCCC	4 (0.000077%)	
AAAGAAGACCCTGTTGAGCTTGACTCTAGTCCGACTTTGTGAAATGACTTGAGAGGTGTAGGATAAGTGGGAGCTTCGGCGCAAGTGAAATACCACTACT	11 (0.000211%)	

AAAGACTCATATGGACTTTGGCTACACCATGAAAGCTTTGAGAAGCAAGAAGAAGGTTGGTTAGTGTTTTGGAGTCGAATATGACTTGATGTCATGTGTA	76 (0.001457%)
AAAGAGAGAAAGGACGAGGTCTTGACCGTCATCTTTTGCCCGAAGGACGGATGAGCTTTGGCGGGACTGAATCACTTCGAGTCACCGTCGACAACTTTTC	5 (0.000096%)
AAAGATTAAGCCATGCATGTGTAAGTATGAACGAATTCAGACTGTGAAACTGCGAATGGCTCATTAAATCAGTTATAGTTTGTTTGATGGTAACTACTAC	13 (0.000249%)
AAAGCAACGTCGCTATGAACGCTTGGCTGCCACAAGCCAGTTATCCCTGTGGTAACTTTTCTGACACCTCTAGCTTCAAATTCCGAAGGTCTAAAGGATC	275 (0.005270%)
AAAGCCAAAGACTCATATGGACTTTGGCTACACCATGAAAGCTTTGAGAAGCAAGAAGAAGGTTGGTTAGTGTTTTGGAGTCGAATATGACTTGATGTCA	307 (0.005883%)
AAAGCCTAAGTAGTGTTTCCTTGTTAGAAGACACAAAGCCAAAGACTCATATGGACTTTGGCTACACCATGAAAGCTTTGAGAAGCAAGAAGAAGGTTGG	7 (0.000134%)
AAAGCTTTCATGGTGTAGCCAAAGTCCATATGAGTCTTTGGCTTTGTGTCTTCTAACAAGGAAACACTACTTAGGCTTATAAGATGCGGTTGCGGTTTAA	7 (0.000134%)
AAAGGCGTAAGAATTGTATCCTTGTTAAAAGACACAAAGCCAAAGACTCATATGGACTTTGGCTACACCATGAAAGCTTTGAGAAGCAAGAAGAAGGTTG	10 (0.000192%)
AAAGGCGTAAGAATTGTATCCTTGTTAGAAGACACAAAGCCAAAGACTCATATGGACTTTGGCTACACCATGAAAGCTTTGAGAAGCAAGAAGAAGGTTG	13 (0.000249%)
AAATCAGAATCAAACGAGCTTTTACCCTTTTGTTCCACACGAGATTTCTGTTCTCGTTGAGCTCATCTTAGGACACCTGCGTTATCTTTTAACAGATGTG	4 (0.000077%)
AAATCAGTTATAGTTTGTTTGATGGTAACTACTACTCGGATAACCGTAGTAATTCTAGAGCTAATACGTGCAACAAACCCCGACTTATGGAAGGGACGCA	5 (0.000096%)
AAATCCGCTAAGGAGTGTGTAACAACTCACCTGCCGAATCAACTAGCCCCGAAAATGGATGGCGCTTAAGCGCGCGACCTATACCCGGCCGTCGGGGCAA	8 (0.000153%)
AAATCCTATGATGTTATCCCATGCTAATGTATCCAGAGCGTAGGCTTGCTTTGAGCACTCTAATTTCTTCAAAGTAACAGCGCCGGAGGCACGACCCGGC	27 (0.000517%)
AAATTTTCATAATTTTTTGACACCTCTAGCTAGGTCATTTGACCTGATACAACATCGGATTTTCATGGTCTAGTTGGGGCTCCGTGGGCATATTTGATGC	17 (0.000326%)
AACAGGGACAGTCGGGGGCATTCGTATTTCATAGTCAGAGGTGAAATTCTTGGATTTATGAAAGACGAACAACTGCGAAAGCATTTGCCAAGGATGTTTT	19 (0.000364%)
AACAGGTCTGTGATGCCCTTAGATGTTCTGGGCCGCACGCGCGCTACACTGATGTATTCAACGAGTTCACACCTTGGCCGACAGGCCCGGGTAATCTTTG	14 (0.000268%)
AACCTAGGCGAGACAAGGGTTCACATTTCGTTCATCACCCTTGGCCGGCTATCGAACAGCCGGACTCCCATCAAAAGATGGTTGCCAAGAACATCTTCGT	3 (0.000057%)
AACCTAGGCGAGACAAGGGTTCACATTTCGTTCATCACCCTTGGCCGGCTTTCGAACAGCCGGACTCCCATCAAAAGATGGTTGCCAAGAACATCTTCGT	5 (0.000096%)
AACGAAGCACGCCCATCCAACCTAGGCGAGACAAGGGTTCACATTTCGTTCATCACCCTTGGCCGGCTATCGAACAGCCGGACTCCCATCAAAAGATGGT	21 (0.000402%)
AACGAAGCACGCCCATCCAACCTAGGCGAGACAAGGGTTCACATTTCGTTCATCACCCTTGGCCGGCTTTCGAACAGCCGGACTCCCATCAAAAGATGGT	19 (0.000364%)
AACGCCTCGAAGAACTAATGGCAGCCCACGCAAGGCAAGCCCATTCTCCTCGACGATTCAGCAGTTTTTGTCCGAGAACTGCTGAGAAAACTCGGAAAAA	11 (0.000211%)
AACGGCGTGCCTCGGCATCAGCGTGCTCCGGGCGTCGGCCTGTGGGCTCCCCATTCGACCCGTCTTGAAACACGGACCAAGGAGTCTGACATGTGTGCGA	4 (0.000077%)
AACGGGCAGAGCCCGCGTCGACCTTTTATCTAATAAATGCGTCCCTTCCATAAGTCGGGGTTTGTTGCACGTATTAGCTCTAGAATTACTACGGTTATCC	14 (0.000268%)
AACGGGTGACGGAGAATTAGGGTTCGATTCCGGAGAGGGAGCCTGAGAAACGGCTACCACATCCAAGGAAGGCAGCAGGCGCGCAAATTACCCAATCCTG	4 (0.000077%)
AAGAAGACCCTGTTGAGCTTGACTCTAGTCCGACTTTGTGAAATGACTTGAGAGGTGTAGGATAAGTGGGAGCTTCGGCGCAAGTGAAATACCACTACTT	11 (0.000211%)
AAGAATTGTATCCTTGTTAGAAGACACAAAGCCAAAGACTCATATGGACTTTGGCTACACCATGAAAGCTTTGAGAAGCAAGAAGAAGGTTGGTTAGTGT	22 (0.000422%)
AAGACACAAAGCCAAAGACTCATATGGACTTTGGCTACACCATGAAAGCTTTGAGAAGCAAGAAGAAGGTTGGTTAGTGTTTTGGAGTCGAATATGACTT	617 (0.011824%)
AAGACCCTGTTGAGCTTGACTCTAGTCCGACTTTGTGAAATGACTTGAGAGGTGTAGGATAAGTGGGAGCTTCGGCGCAAGTGAAATACCACTACTTTTA	4 (0.000077%)
AAGACTCATATGGACTTTGGCTACACCATGAAAGCTTTGAGAAGCAAGAAGAAGGTTGGTTAGTGTTTTGGAGTCGAATATGACTTGATGTCATGTGTAT	25 (0.000479%)
AAGAGCCCGGTATTGTTATTTATTGTCACTACCTCCCCGTGTCAGGATTGGGTAATTTGCGCGCCTGCTGCCTTCCTTGGATGTGGTAGCCGTTTCTCAG	9 (0.000172%)
AAGATCGATC	199249 (0.381850%)
AAGCAACGTCGCTATGAACGCTTGGCTGCCACAAGCCAGTTATCCCTGTGGTAACTTTTCTGACACCTCTAGCTTCAAATTCCGAAGGTCTAAAGGATCG	130 (0.002491%)
AAGCCAAAGACTCATATGGACTTTGGCTACACCATGAAAGCTTTGAGAAGCAAGAAGAAGGTTGGTTAGTGTTTTGGAGTCGAATATGACTTGATGTCAT	12 (0.000230%)
AAGCCATGCATGTGTAAGTATGAACGAATTCAGACTGTGAAACTGCGAATGGCTCATTAAATCAGTTATAGTTTGTTTGATGGTAACTACTACTCGGATA	5 (0.000096%)
AAGCCTAAGTAGTGTTTCCTTGTTAGAAGACACAAAGCCAAAGACTCATATGGACTTTGGCTACACCATGAAAGCTTTGAGAAGCAAGAAGAAGGTTGGT	1195 (0.022902%)
AAGCGCGCGACCTATACCCGGCCGTCGGGGCAAGAGCCAGGCCTCGATGAGTAGGAGGGCGCGGCGGTCGCTGCAAAACCTAGGGCGCGAGCCCGGGCGG	18 (0.000345%)
AAGCTTTCATGGTGTAGCCAAAGTCCATATGAGTCTTTGGCTTTGTGTCTTCTAACAAGGAAACACTACTTAGGCTTATAAGATGCGGTTGCGGTTTAAG	3 (0.000057%)
AAGGATACTAAATCCTATTTTCTGGTAAATTTTCATAATTTTTTGACACCTCTAGCTAGGTCATTTGACCTGATACAACATCGGATTTTCATGGTCTAGT	7 (0.000134%)
AAGGCGTAAGAATTGTATCCTTGTTAAAAGACACAAAGCCAAAGACTCATATGGACTTTGGCTACACCATGAAAGCTTTGAGAAGCAAGAAGAAGGTTGG	5 (0.000096%)
AAGGCGTAAGAATTGTATCCTTGTTAGAAGACACAAAGCCAAAGACTCATATGGACTTTGGCTACACCATGAAAGCTTTGAGAAGCAAGAAGAAGGTTGG	7 (0.000134%)
AAGGGATTTAGATTGTACTCATTCCAATTACCAGACTCGAAAGAGCCCGGTATTGTTATTTATTGTCACTACCTCCCCGTGTCAGGATTGGGTAATTTGC	51 (0.000977%)
AAGGGTTCACATTTCGTTCATCACCCTTGGCCGGCTATCGAACAGCCGGACTCCCATCAAAAGATGGTTGCCAAGAACATCTTCGTTACGGTTTGCTAAT	4 (0.000077%)
AAGTAGTGTTTCCTTGTTAGAAGACACAAAGCCAAAGACTCATATGGACTTTGGCTACACCATGAAAGCTTTGAGAAGCAAGAAGAAGGTTGGTTAGTGT	28 (0.000537%)

AAGTATGAACGAATTCAGACTGTGAAACTGCGAATGGCTCATTAAATCAGTTATAGTTTGTTTGATGGTAACTACTACTCGGATAACCGTAGTAATTCTA	3 (0.000057%)	
AAGTCATATTCGACTCCAAAACACTAACCAACCTTCTTCTTGCTTCTCAAAGCTTTCATGGTGTAGCCAAAGTCCATATGAGTCTTTGGCTTTGTGTCTT	138 (0.002645%)	
AAGTCTGGTGCCAGCAGCCGCGGTAATTCCAGCTCCAATAGCGTATATTTAAGTTGTTGCAGTTAAAAAGCTCGTAGTTGAACCTTGGGATGGGTCGGCC	5 (0.000096%)	
AAGTTCACCACCGCATGTCGGTACGCTCCAGGCGTCCTTGGCTCGGATTTAGGCCAACCGCGTGCGGTAACACACGGGAGACCAGCTTCCGTCCCGCATC	16 (0.000307%)	
AAGTTCTTATACTCAATCATACACATGACATCAAGTCATATTCGACTCCAAAACACTAACCAACCTTCTTCTTGCTTCTCAAAGCTTTCATGGTGTAGCC	45 (0.000862%)	
AAGTTGTTATACTCAATCATACACATGACATCAAGTCATA	115 (0.000882%)	
AATAACAGGTCTGTGATGCCCTTAGATGTTCTGGGCCGCACGCGCGCTACACTGATGTATTCAACGAGTTCACACCTTGGCCGACAGGCCCGGGTAATCT	14 (0.000268%)	
AATATGACTTGATGTCATGTGTATGATTGAGTATAAGAAC	1373 (0.010525%)	
AATCAAACGAGCTTTTACCCTTTTGTTCCACACGAGATTTCTGTTCTCGTTGAGCTCATCTTAGGACACCTGCGTTATCTTTTAACAGATGTGCCGCCCC	28 (0.000537%)	
AATCAGTTATAGTTTGTTTGATGGTAACTACTACTCGGATAACCGTAGTAATTCTAGAGCTAATACGTGCAACAAACCCCGACTTATGGAAGGGACGCAT	5 (0.000096%)	
AATCATACACATGACAACAAGTCATATTCGACTCCAAAACACTAACCAACCTTCTTCTTGCTTCTCAAAGCTTTCATGGTGTAGCCAAAGTCCATATGAG	16 (0.000307%)	
AATCATACACATGACATCAAGTCATATTCGACTCCAAAACACTAACCAACCTTCTTCTTGCTTCTCAAAGCTTTCATGGTGTAGCCAAAGTCCATATGAG	70 (0.001342%)	
AATCATCAGAGCAACGGGCAGAGCCCGCGTCGACCTTTTATCTAATAAATGCGTCCCTTCCATAAGTCGGGGTTTGTTGCACGTATTAGCTCTAGAATTA	45 (0.000862%)	
AATCCGCTAAGGAGTGTGTAACAACTCACCTGCCGAATCAACTAGCCCCGAAAATGGATGGCGCTTAAGCGCGCGACCTATACCCGGCCGTCGGGGCAAG	14 (0.000268%)	
AATCCTATGATGTTATCCCATGCTAATGTATCCAGAGCGTAGGCTTGCTTTGAGCACTCTAATTTCTTCAAAGTAACAGCGCCGGAGGCACGACCCGGCC	34 (0.000652%)	
AATCCTTGTTAGTTTCTTTTCCTCCGCTTATTGATATGCTTAAACTCAGCGGGTAATCCCGCCTGACCTGGGGTCGCTATATGGACTTTGGGTCATCTAC	7 (0.000134%)	
AATCGGTAGGAGCGACGGGCGGTGTGTACAAAGGGCAGGGACGTAGTCAACGCGAGCTGATGACTCGCGCTTACTAGGAATTCCTCGTTGAAGACCAACA	68 (0.001303%)	
AATGATTAACAGGGACAGTCGGGGGCATTCGTATTTCATAGTCAGAGGTGAAATTCTTGGATTTATGAAAGACGAACAACTGCGAAAGCATTTGCCAAGG	89 (0.001706%)	
AATGTATCCAGAGCGTAGGCTTGCTTTGAGCACTCTAATTTCTTCAAAGTAACAGCGCCGGAGGCACGACCCGGCCAATTAAGACCAGGAGCGTATCGCC	8 (0.000153%)	
AATTACCAGACTCGAAAGAGCCCGGTATTGTTATTTATTGTCACTACCTCCCCGTGTCAGGATTGGGTAATTTGCGCGCCTGCTGCCTTCCTTGGATGTG	21 (0.000402%)	
AATTAGGGTTCGATTCCGGAGAGGGAGCCTGAGAAACGGCTACCACATCCAAGGAAGGCAGCAGGCGCGCAAATTACCCAATCCTGACACGGGGAGGTAG	17 (0.000326%)	
AATTCCAGCTCCAATAGCGTATATTTAAGTTGTTGCAGTTAAAAAGCTCGTAGTTGAACCTTGGGATGGGTCGGCCGGTCCGCCTTTGGTGTGCATTGGT	10 (0.000192%)	
AATTCCCCGCCACATCCTCTCAAACGCAATGGAAAGAGAGAAAGGACGAGGTCTTGACCGTCATCTTTTGCCCGAAGGACGGATGAGCTTTGGCGGGACT	13 (0.000249%)	
AATTGTATCCTTGTTAGAAGACACAAAGCCAAAGACTCATATGGACTTTGGCTACACCATGAAAGCTTTGAGAAGCAAGAAGAAGGTTGGTTAGTGTTTT	4 (0.000077%)	
AATTGTTGGTCTTCAACGAGGAATTCCTAGTAAGCGCGAGTCATCAGCTCGCGTTGACTACGTCCCTGCCCTTTGTACACACCGCCCGTCGCTCCTACCG	1354 (0.025949%)	
AATTTTCATAATTTTTTGACACCTCTAGCTAGGTCATTTGACCTGATACAACATCGGATTTTCATGGTCTAGTTGGGGCTCCGTGGGCATATTTGATGCA	3 (0.000057%)	
ACAAAGCCAAAGACTCATATGGACTTTGGCTACACCATGAAAGCTTTGAGAAGCAAGAAGAAGGTTGGTTAGTGTTTTGGAGTCGAATATGACTTGATGT	142 (0.002721%)	
ACAAGGGTTCACATTTCGTTCATCACCCTTGGCCGGCTTTCGAACAGCCGGACTCCCATCAAAAGATGGTTGCCAAGAACATCTTCGTTACGGTTTGCTA	3 (0.000057%)	
ACACAAAGCCAAAGACTCATATGGACTTTGGCTACACCATGAAAGCTTTGAGAAGCAAGAAGAAGGTTGGTTAGTGTTTTGGAGTCGAATATGACTTGAT	171 (0.003277%)	
ACACATGACATCAAGTCATATTCGACTCCAAAACACTAACCAACCTTCTTCTTGCTTCTCAAAGCTTTCATGGTGTAGCCAAAGTCCATATGAGTCTTTG	32 (0.000613%)	
ACACCATGAAAGCTTTGAGAAGCAAGAAGAAGGTTGGTTAGTGTTTTGGAGTCGAATATGACTTGATGTCATGTGTATGATTGAGTATAACAACTTAAAC	4 (0.000077%)	
ACAGGTCTGTGATGCCCTTAGATGTTCTGGGCCGCACGCGCGCTACACTGATGTATTCAACGAGTTCACACCTTGGCCGACAGGCCCGGGTAATCTTTGA	4 (0.000077%)	
ACATCAAGTCATATTCGACTCCAAAACACTAACCAACCTTCTTCTTGCTTCTCAAAGCTTTCATGGTGTAGCCAAAGTCCATATGAGTCTTTGGCTTTGT	48 (0.000920%)	
ACATCTGTTAAAAGATAACGCAGGTGTCCTAAGATGAGCTCAACGAGAACAGAAATCTCGTGTGGAACAAAAGGGTAAAAGCTCGTTTGATTCTGATTTT	3 (0.000057%)	
ACATGACATCAAGTCATATTCGACTCCAAAACACTAACCAACCTTCTTCTTGCTTCTCAAAGCTTTCATGGTGTAGCCAAAGTCCATATGAGTCTTTGGC	67 (0.001284%)	
ACATTGTCAGGTGGGGAGTTTGGCTGGGGCGGCACATCTGTTAAAAGATAACGCAGGTGTCCTAAGATGAGCTCAACGAGAACAGAAATCTCGTGTGGAA	7 (0.000134%)	
ACCACCGCATGTCGGTACGCTCCAGGCGTCCTTGGCTCGGATTTAGGCCAACCGCGTGCGGTAACACACGGGAGACCAGCTTCCGTCCCGCATCAGCAAA	5 (0.000096%)	
ACCAGAGGCTGTTCACCTTGGAGACCTGATGCGGTTATGAGTACGACCGGGCGTGAGCGGCACTCGGTCCTCCGGATTTTCAAGGGCCGCCGGGGGCGCA	14 (0.000268%)	
ACCATCCTTTGCTGATGCGGGACGGAAGCTGGTCTCCCGTGTGTTACCGCACGCGGTTGGCCTAAATCCGAGCCAAGGACGCCTGGAGCGTACCGACATG	9 (0.000172%)	
ACCCTAACGCCTCGAAGAACTAATGGCAGCCCACGCAAGGCAAGCCCATTCTCCTCGACGATTCAGCAGTTTTTGTCCGAGAACTGCTGAGAAAACTCGG	5 (0.000096%)	
ACCCTGTTGAGCTTGACTCTAGTCCGACTTTGTGAAATGACTTGAGAGGTGTAGGATAAGTGGGAGCTTCGGCGCAAGTGAAATACCACTACTTTTAACG	11 (0.000211%)	
ACCGCATGTCGGTACGCTCCAGGCGTCCTTGGCTCGGATTTAGGCCAACCGCGTGCGGTAACACACGGGAGACCAGCTTCCGTCCCGCATCAGCAAAGGA	5 (0.000096%)	
ACCGGATTGCTCCGTTCCGCATCCGACCAGGACGCATCGCCGGCCCCCATCCGCTTCCCTCCCGACAATTTCAAGCACTCTTTGACTCTCTTTTCAAAGT	6 (0.000115%)	

ACCTAGGCGAGACAAGGGTTCACATTTCGTTCATCACCCTTGGCCGGCTATCGAACAGCCGGACTCCCATC<br>AAAAGATGGTTGCCAAGAACATCTTCGTT	6 (0.000115%)	
ACCTAGGCGAGACAAGGGTTCACATTTCGTTCATCACCCTTGGCCGGCTTTCGAACAGCCGGACTCCCATC<br>AAAAGATGGTTGCCAAGAACATCTTCGTT	7 (0.000134%)	
ACGAAGCACGCCCATCCAACCTAGGCGAGACAAGGGTTCACATTTCGTTCATCACCCTTGGCCGGCTATCG<br>AACAGCCGGACTCCCATCAAAAGATGGTT	9 (0.000172%)	
ACGAAGCACGCCCATCCAACCTAGGCGAGACAAGGGTTCACATTTCGTTCATCACCCTTGGCCGGCTTTCG<br>AACAGCCGGACTCCCATCAAAAGATGGTT	7 (0.000134%)	
ACGCGCCTAACGGCGTGCCTCGGCATCAGCGTGCTCCGGGCGTCGGCCTGTGGGCTCCCCATTCGACCCGT<br>CTTGAAACACGGACCAAGGAGTCTGACAT	21 (0.000402%)	
ACGCTTTCACGGTTCGTATTCGTACTGAAAATCAGAATCAAACGAGCTTTTACCCTTTTGTTCCACACGAG<br>ATTTCTGTTCTCGTTGAGCTCATCTTAGG	11 (0.000211%)	
ACGGGCAGAGCCCGCGTCGACCTTTTATCTAATAAATGCGTCCCTTCCATAAGTCGGGGTTTGTTGCACGT<br>ATTAGCTCTAGAATTACTACGGTTATCCG	4 (0.000077%)	
ACGGGCGGTGTGTACAAAGGGCAGGGACGTAGTCAACGCGAGCTGATGACTCGCGCTTACTAGGAATTCCT<br>CGTTGAAGACCAACAATTGCAATGATCGA	3 (0.000057%)	
ACGTGGGTGGTTCGCCGCCCGCGACGTCGCGAGAAGTCCACTAAACCTTATCATTTAGAGGAAGGAGAAGT<br>CGTAACAAGGTTTCCGTAGGTGAACCTGC	4 (0.000077%)	
ACTAAATCCTATTTTCTGGTAAATTTTCATAATTTTTTGACACCTCTAGCTAGGTCATTTGACCTGATACA<br>ACATCGGATTTTCATGGTCTAGTTGGGGC	20 (0.000383%)	
ACTCAATCATACACATGACAACAAGTCATATTCGACTCCAAAACACTAACCAACCTTCTTCTTGCTTCTCA<br>AAGCTTTCATGGTGTAGCCAAAGTCCATA	6 (0.000115%)	
ACTCAATCATACACATGACATCAAGTCATATTCGACTCCAAAACACTAACCAACCTTCTTCTTGCTTCTCA<br>AAGCTTTCATGGTGTAGCCAAAGTCCATA	25 (0.000479%)	
ACTCATATGGACTTTGGCTACACCATGAAAGCTTTGAGAAGCAAGAAGAAGGTTGGTTAGTGTTTTGGAGT<br>CGAATATGACTTGATGTCATGTGTATGAT	74 (0.001418%)	
ACTCATTCCAATTACCAGACTCGAAAGAGCCCGGTATTGTTATTTATTGTCACTACCTCCCCGTGTCAGGA<br>TTGGGTAATTTGCGCGCCTGCTGCCTTCC	43 (0.000824%)	
ACTCCAAAACACTAACCAACCTTCTTCTTGCTTCTCAAAGCTTTCATGGTGTAGCCAAAGTCCATATGAGT<br>CTTTGGCTTTGTGTCTTCTAACAAGGAAA	177 (0.003392%)	
ACTCGAAAGAGCCCGGTATTGTTATTTATTGTCACTACCTCCCCGTGTCAGGATTGGGTAATTTGCGCGCC<br>TGCTGCCTTCCTTGGATGTGGTAGCCGTT	4 (0.000077%)	
ACTGAAAATCAGAATCAAACGAGCTTTTACCCTTTTGTTCCACACGAGATTTCTGTTCTCGTTGAGCTCAT<br>CTTAGGACACCTGCGTTATCTTTTAACAG	6 (0.000115%)	
ACTTCCCTTGCCTACATTGTTCCATCGACCAGAGGCTGTTCACCTTGGAGACCTGATGCGGTTATGAGTAC<br>GACCGGGCGTGAGCGGCACTCGGTCCTCC	23 (0.000441%)	
ACTTGATGTCATGTGTATGATTGAGTATAAGAACTTAAAC	319 (0.002445%)	
ACTTTGTGAAATGACTTGAGAGGTGTAGGATAAGTGGGAGCTTCGGCGCAAGTGAAATACCACTACTTTTA<br>ACGTTATTTTACTTACTCCGTGAATCGGA	5 (0.000096%)	
AGAAGACACAAAGCCAAAGACTCATATGGACTTTGGCTACACCATGAAAGCTTTGAGAAGCAAGAAGAAGG<br>TTGGTTAGTGTTTTGGAGTCGAATATGAC	71 (0.001361%)	
AGAAGACCCTGTTGAGCTTGACTCTAGTCCGACTTTGTGAAATGACTTGAGAGGTGTAGGATAAGTGGGAG<br>CTTCGGCGCAAGTGAAATACCACTACTTT	31 (0.000594%)	
AGAATTAGGGTTCGATTCCGGAGAGGGAGCCTGAGAAACGGCTACCACATCCAAGGAAGGCAGCAGGCGCG<br>CAAATTACCCAATCCTGACACGGGGAGGT	4 (0.000077%)	
AGAATTGTATCCTTGTTAGAAGACACAAAGCCAAAGACTCATATGGACTTTGGCTACACCATGAAAGCTTT<br>GAGAAGCAAGAAGAAGGTTGGTTAGTGTT	7 (0.000134%)	
AGACACAAAGCCAAAGACTCATATGGACTTTGGCTACACCATGAAAGCTTTGAGAAGCAAGAAGAAGGTTG<br>GTTAGTGTTTTGGAGTCGAATATGACTTG	57 (0.001092%)	
AGACCCTGTTGAGCTTGACTCTAGTCCGACTTTGTGAAATGACTTGAGAGGTGTAGGATAAGTGGGAGCTT<br>CGGCGCAAGTGAAATACCACTACTTTTAA	5 (0.000096%)	
AGACTCATATGGACTTTGGCTACACCATGAAAGCTTTGAGAAGCAAGAAGAAGGTTGGTTAGTGTTTTGGA<br>GTCGAATATGACTTGATGTCATGTGTATG	8 (0.000153%)	
AGACTCGAAAGAGCCCGGTATTGTTATTTATTGTCACTACCTCCCCGTGTCAGGATTGGGTAATTTGCGCG<br>CCTGCTGCCTTCCTTGGATGTGGTAGCCG	6 (0.000115%)	
AGAGCAACGGGCAGAGCCCGCGTCGACCTTTTATCTAATAAATGCGTCCCTTCCATAAGTCGGGGTTTGTT<br>GCACGTATTAGCTCTAGAATTACTACGGT	30 (0.000575%)	
AGAGCCCGCGTCGACCTTTTATCTAATAAATGCGTCCCTTCCATAAGTCGGGGTTTGTTGCACGTATTAGC<br>TCTAGAATTACTACGGTTATCCGAGTAGT	10 (0.000192%)	
AGAGCGTAGGCTTGCTTTGAGCACTCTAATTTCTTCAAAGTAACAGCGCCGGAGGCACGACCCGGCCAATT<br>AAGACCAGGAGCGTATCGCCGACCGAAGG	10 (0.000192%)	
AGAGGTGAAATTCTTGGATTTATGAAAGACGAACAACTGCGAAAGCATTTGCCAAGGATGTTTTCATTAAT<br>CAAGAACGAAAGTTGGGGGCTCGAAGACG	4 (0.000077%)	
AGATCGGAAGAGCACACGTCTGAACTCCAGTCACGAGATT	52 (0.000399%)	
AGATGTTCTGGGCCGCACGCGCGCTACACTGATGTATTCAACGAGTTCACACCTTGGCCGACAGGCCCGGG<br>TAATCTTTGAAATTTCATCGTGATGGGGA	4 (0.000077%)	
AGATTAAGCCATGCATGTGTAAGTATGAACGAATTCAGACTGTGAAACTGCGAATGGCTCATTAAATCAGT<br>TATAGTTTGTTTGATGGTAACTACTACTC	5 (0.000096%)	
AGATTGTACTCATTCCAATTACCAGACTCGAAAGAGCCCGGTATTGTTATTTATTGTCACTACCTCCCCGT<br>GTCAGGATTGGGTAATTTGCGCGCCTGCT	5 (0.000096%)	
AGCAACGGGCAGAGCCCGCGTCGACCTTTTATCTAATAAATGCGTCCCTTCCATAAGTCGGGGTTTGTTGC<br>ACGTATTAGCTCTAGAATTACTACGGTTA	3 (0.000057%)	
AGCAACGTCGCTATGAACGCTTGGCTGCCACAAGCCAGTTATCCCTGTGGTAACTTTTCTGACACCTCTAG<br>CTTCAAATTCCGAAGGTCTAAAGGATCGA	16 (0.000307%)	
AGCACGCCCATCCAACCTAGGCGAGACAAGGGTTCACATTTCGTTCATCACCCTTGGCCGGCTATCGAACA<br>GCCGGACTCCCATCAAAAGATGGTTGCCA	17 (0.000326%)	
AGCACGCCCATCCAACCTAGGCGAGACAAGGGTTCACATTTCGTTCATCACCCTTGGCCGGCTTTCGAACA<br>GCCGGACTCCCATCAAAAGATGGTTGCCA	20 (0.000383%)	
AGCACGCGCCTAACGGCGTGCCTCGGCATCAGCGTGCTCCGGGCGTCGGCCTGTGGGCTCCCCATTCGACC<br>CGTCTTGAAACACGGACCAAGGAGTCTGA	36 (0.000690%)	
AGCAGCCGCGGTAATTCCAGCTCCAATAGCGTATATTTAAGTTGTTGCAGTTAAAAAGCTCGTAGTTGAAC<br>CTTGGGATGGGTCGGCCGGTCCGCCTTTG	8 (0.000153%)	
AGCCAAAGACTCATATGGACTTTGGCTACACCATGAAAGCTTTGAGAAGCAAGAAGAAGGTTGGTTAGTGT<br>TTTGGAGTCGAATATGACTTGATGTCATG	11 (0.000211%)	

AGCCGCGGTAATTCCAGCTCCAATAGCGTATATTTAAGTTGTTGCAGTTAAAAAGCTCGTAGTTGAACCTTGGGATGGGTCGGCCGGTCCGCCTTTGGTG	6 (0.000115%)
AGCCTAAGTAGTGTTTCCTTGTTAGAAGACACAAAGCCAAAGACTCATATGGACTTTGGCTACACCATGAAAGCTTTGAGAAGCAAGAAGAAGGTTGGTT	35 (0.000671%)
AGCGACGGGCGGTGTGTACAAAGGGCAGGGACGTAGTCAACGCGAGCTGATGACTCGCGCTTACTAGGAATTCCTCGTTGAAGACCAACAATTGCAATGA	6 (0.000115%)
AGGACATTGTCAGGTGGGGAGTTTGGCTGGGGCGGCACATCTGTTAAAAGATAACGCAGGTGTCCTAAGATGAGCTCAACGAGAACAGAAATCTCGTGTG	154 (0.002951%)
AGGAGCGACGGGCGGTGTGTACAAAGGGCAGGGACGTAGTCAACGCGAGCTGATGACTCGCGCTTACTAGGAATTCCTCGTTGAAGACCAACAATTGCAA	13 (0.000249%)
AGGCCACGCTTTCACGGTTCGTATTCGTACTGAAAATCAGAATCAAACGAGCTTTTACCCTTTTGTTCCACACGAGATTTCTGTTCTCGTTGAGCTCATC	20 (0.000383%)
AGGCGAGACAAGGGTTCACATTTCGTTCATCACCCTTGGCCGGCTATCGAACAGCCGGACTCCCATCAAAAGATGGTTGCCAAGAACATCTTCGTTACGG	3 (0.000057%)
AGGCGAGACAAGGGTTCACATTTCGTTCATCACCCTTGGCCGGCTTTCGAACAGCCGGACTCCCATCAAAAGATGGTTGCCAAGAACATCTTCGTTACGG	4 (0.000077%)
AGGCGTAAGAATTGTATCCTTGTTAAAAGACACAAAGCCAAAGACTCATATGGACTTTGGCTACACCATGAAAGCTTTGAGAAGCAAGAAGAAGGTTGGT	13 (0.000249%)
AGGCGTAAGAATTGTATCCTTGTTAGAAGACACAAAGCCAAAGACTCATATGGACTTTGGCTACACCATGAAAGCTTTGAGAAGCAAGAAGAAGGTTGGT	11 (0.000211%)
AGGCTGTCCCGAGTGTGAGCGAGGTGTGAGTGTCGCCCATGGGCATCGACACCTTGCGGCTAGGAACTGGAACGAGACGGGTAGCAAAGATTTCGAGTAG	11 (0.000211%)
AGGCTGTCCCGAGTGTGAGCGAGGTGTGAGTGTCGCCCATGGGCATCGACACCTTGCGGCTAGGAACTGGAACGAGACGGGTGGCAAAGATTTCGAGTAG	11 (0.000211%)
AGGCTTGCTTTGAGCACTCTAATTTCTTCAAAGTAACAGCGCCGGAGGCACGACCCGGCCAATTAAGACCAGGAGCGTATCGCCGACCGAAGGGACAAGC	4 (0.000077%)
AGGGAATCCTTGTTAGTTTCTTTTCCTCCGCTTATTGATATGCTTAAACTCAGCGGGTAATCCCGCCTGACCTGGGGTCGCTATATGGACTTTGGGTCAT	216 (0.004140%)
AGGGACAGTCGGGGGCATTCGTATTTCATAGTCAGAGGTGAAATTCTTGGATTTATGAAAGACGAACAACTGCGAAAGCATTTGCCAAGGATGTTTTCAT	6 (0.000115%)
AGGGATTTAGATTGTACTCATTCCAATTACCAGACTCGAAAGAGCCCGGTATTGTTATTTATTGTCACTACCTCCCCGTGTCAGGATTGGGTAATTTGCG	10 (0.000192%)
AGGGCAAGTCTGGTGCCAGCAGCCGCGGTAATTCCAGCTCCAATAGCGTATATTTAAGTTGTTGCAGTTAAAAAGCTCGTAGTTGAACCTTGGGATGGGT	27 (0.000517%)
AGGTCTGTGATGCCCTTAGATGTTCTGGGCCGCACGCGCGCTACACTGATGTATTCAACGAGTTCACACCTTGGCCGACAGGCCCGGGTAATCTTTGAAA	3 (0.000057%)
AGGTGGGGAGTTTGGCTGGGGCGGCACATCTGTTAAAAGATAACGCAGGTGTCCTAAGATGAGCTCAACGAGAACAGAAATCTCGTGTGGAACAAAAGGG	6 (0.000115%)
AGGTGTGAGTGTCGCCCATGGGCATCGACACCTTGCGGCTAGGAACTGGAACGAGACGGGTGGCAAAGATTTCGAGTAGCACTTCATACTACCGTGGGTT	3 (0.000057%)
AGTAGTCATATGCTTGTCTCAAAGATTAAGCCATGCATGTGTAAGTATGAACGAATTCAGACTGTGAAACTGCGAATGGCTCATTAAATCAGTTATAGTT	89 (0.001706%)
AGTAGTGTTTCCTTGTTAGAAGACACAAAGCCAAAGACTCATATGGACTTTGGCTACACCATGAAAGCTTTGAGAAGCAAGAAGAAGGTTGGTTAGTGTT	12 (0.000230%)
AGTCAGAGGTGAAATTCTTGGATTTATGAAAGACGAACAACTGCGAAAGCATTTGCCAAGGATGTTTTCATTAATCAAGAACGAAAGTTGGGGGCTCGAA	9 (0.000172%)
AGTCATATGCTTGTCTCAAAGATTAAGCCATGCATGTGTAAGTATGAACGAATTCAGACTGTGAAACTGCGAATGGCTCATTAAATCAGTTATAGTTTGT	18 (0.000345%)
AGTCGAAATCCGCTAAGGAGTGTGTAACAACTCACCTGCCGAATCAACTAGCCCCGAAAATGGATGGCGCTTAAGCGCGCGACCTATACCCGGCCGTCGG	7 (0.000134%)
AGTCGGGGGCATTCGTATTTCATAGTCAGAGGTGAAATTCTTGGATTTATGAAAGACGAACAACTGCGAAAGCATTTGCCAAGGATGTTTTCATTAATCA	4 (0.000077%)
AGTGTATCCTTGTTAGAAGACACAAAGCCAAAGACTCATATGGACTTTGGCTACACCATGAAAGCTTTGAGAAGCAAGAAGAAGGTTGGTTAGTGTTTTG	756 (0.014488%)
AGTGTTTCCTTGTTAGAAGACACAAAGCCAAAGACTCATATGGACTTTGGCTACACCATGAAAGCTTTGAGAAGCAAGAAGAAGGTTGGTTAGTGTTTTG	13 (0.000249%)
AGTTATCATGAATCATCAGAGCAACGGGCAGAGCCCGCGTCGACCTTTTATCTAATAAATGCGTCCCTTCCATAAGTCGGGGTTTGTTGCACGTATTAGC	6 (0.000115%)
AGTTCACCACCGCATGTCGGTACGCTCCAGGCGTCCTTGGCTCGGATTTAGGCCAACCGCGTGCGGTAACACACGGGAGACCAGCTTCCGTCCCGCATCA	7 (0.000134%)
AGTTCTTATACTCAATCATACACATGACATCAAGTCATATTCGACTCCAAAACACTAACCAACCTTCTTCTTGCTTCTCAAAGCTTTCATGGTGTAGCCA	26 (0.000498%)
AGTTGTTATACTCAATCATACACATGACATCAAGTCATAT	83 (0.000636%)
AGTTTGGCTGGGGCGGCACATCTGTTAAAAGATAACGCAGGTGTCCTAAGATGAGCTCAACGAGAACAGAAATCTCGTGTGGAACAAAAGGGTAAAAGCT	5 (0.000096%)
ATAACAGGTCTGTGATGCCCTTAGATGTTCTGGGCCGCACGCGCGCTACACTGATGTATTCAACGAGTTCACACCTTGGCCGACAGGCCCGGGTAATCTT	15 (0.000287%)
ATAAGGATACTAAATCCTATTTTCTGGTAAATTTTCATAATTTTTTGACACCTCTAGCTAGGTCATTTGACCTGATACAACATCGGATTTTCATGGTCTA	20 (0.000383%)
ATAATTTTTTGACACCTCTAGCTAGGTCATTTGACCTGATACAACATCGGATTTTCATGGTCTAGTTGGGGCTCCGTGGGCATATTTGATGCAAACTTGA	8 (0.000153%)
ATACACATGACATCAAGTCATATTCGACTCCAAAACACTAACCAACCTTCTTCTTGCTTCTCAAAGCTTTCATGGTGTAGCCAAAGTCCATATGAGTCTT	293 (0.005615%)
ATACCCGGCCGTCGGGGCAAGAGCCAGGCCTCGATGAGTAGGAGGGCGCGGCGGTCGCTGCAAAACCTAGGGCGCGAGCCCGGGCGGAGCGGCCGTCGGT	8 (0.000153%)
ATACTAAATCCTATTTTCTGGTAAATTTTCATAATTTTTTGACACCTCTAGCTAGGTCATTTGACCTGATACAACATCGGATTTTCATGGTCTAGTTGGG	5 (0.000096%)
ATACTCAATCATACACATGACAACAAGTCATATTCGACTCCAAAACACTAACCAACCTTCTTCTTGCTTCTCAAAGCTTTCATGGTGTAGCCAAAGTCCA	13 (0.000249%)
ATACTCAATCATACACATGACATCAAGTCATATTCGACTCCAAAACACTAACCAACCTTCTTCTTGCTTCTCAAAGCTTTCATGGTGTAGCCAAAGTCCA	22 (0.000422%)
ATAGGCCACGCTTTCACGGTTCGTATTCGTACTGAAAATCAGAATCAAACGAGCTTTTACCCTTTTGTTCCACACGAGATTTCTGTTCTCGTTGAGCTCA	79 (0.001514%)
ATAGTCAGAGGTGAAATTCTTGGATTTATGAAAGACGAACAACTGCGAAAGCATTTGCCAAGGATGTTTTCATTAATCAAGAACGAAAGTTGGGGGCTCG	13 (0.000249%)

ATAGTGGCCTACCATGGTGGTAACGGGTGACGGAGAATTAGGGTTCGATTCCGGAGAGGGAGCCTGAGAAA<br>CGGCTACCACATCCAAGGAAGGCAGCAGG	16 (0.000307%)	
ATAGTTTGTTTGATGGTAACTACTACTCGGATAACCGTAGTAATTCTAGAGCTAATACGTGCAACAAACCC<br>CGACTTATGGAAGGGACGCATTTATTAGA	3 (0.000057%)	
ATATGACTTGATGTCATGTGTATGATTGAGTATAAGAACT	118 (0.000905%)	
ATATGCTTGTCTCAAAGATTAAGCCATGCATGTGTAAGTATGAACGAATTCAGACTGTGAAACTGCGAATG<br>GCTCATTAAATCAGTTATAGTTTGTTTGA	59 (0.001131%)	
ATATGGACTTTGGCTACACCATGAAAGCTTTGAGAAGCAAGAAGAAGGTTGGTTAGTGTTTTGGAGTCGAA<br>TATGACTTGATGTCATGTGTATGATTGAG	19 (0.000364%)	
ATATTCGACTCCAAAACACTAACCAACCTTCTTCTTGCTTCTCAAAGCTTTCATGGTGTAGCCAAAGTCCA<br>TATGAGTCTTTGGCTTTGTGTCTTCTAAC	145 (0.002779%)	
ATCAAACGAGCTTTTACCCTTTTGTTCCACACGAGATTTCTGTTCTCGTTGAGCTCATCTTAGGACACCTG<br>CGTTATCTTTTAACAGATGTGCCGCCCCA	5 (0.000096%)	
ATCAAGTCATATTCGACTCCAAAACACTAACCAACCTTCTTCTTGCTTCTCAAAGCTTTCATGGTGTAGCC<br>AAAGTCCATATGAGTCTTTGGCTTTGTGT	1466 (0.028095%)	
ATCAAGTTCACCACCGCATGTCGGTACGCTCCAGGCGTCCTTGGCTCGGATTTAGGCCAACCGCGTGCGGT<br>AACACACGGGAGACCAGCTTCCGTCCCGC	76 (0.001457%)	
ATCAGAATCAAACGAGCTTTTACCCTTTTGTTCCACACGAGATTTCTGTTCTCGTTGAGCTCATCTTAGGA<br>CACCTGCGTTATCTTTTAACAGATGTGCC	10 (0.000192%)	
ATCAGAGCAACGGGCAGAGCCCGCGTCGACCTTTTATCTAATAAATGCGTCCCTTCCATAAGTCGGGGTTT<br>GTTGCACGTATTAGCTCTAGAATTACTAC	26 (0.000498%)	
ATCAGCGTGCTCCGGGCGTCGGCCTGTGGGCTCCCCATTCGACCCGTCTTGAAACACGGACCAAGGAGTCT<br>GACATGTGTGCGAGTCAACGGGTGAGTAA	6 (0.000115%)	
ATCAGTTATAGTTTGTTTGATGGTAACTACTACTCGGATAACCGTAGTAATTCTAGAGCTAATACGTGCAA<br>CAAACCCCGACTTATGGAAGGGACGCATT	8 (0.000153%)	
ATCATAAGGATACTAAATCCTATTTTCTGGTAAATTTTCATAATTTTTTGACACCTCTAGCTAGGTCATTT<br>GACCTGATACAACATCGGATTTTCATGGT	4 (0.000077%)	
ATCATACACATGACAACAAGTCATATTCGACTCCAAAACACTAACCAACCTTCTTCTTGCTTCTCAAAGCT<br>TTCATGGTGTAGCCAAAGTCCATATGAGT	3 (0.000057%)	
ATCATACACATGACATCAAGTCATATTCGACTCCAAAACACTAACCAACCTTCTTCTTGCTTCTCAAAGCT<br>TTCATGGTGTAGCCAAAGTCCATATGAGT	35 (0.000671%)	
ATCATCAGAGCAACGGGCAGAGCCCGCGTCGACCTTTTATCTAATAAATGCGTCCCTTCCATAAGTCGGGG<br>TTTGTTGCACGTATTAGCTCTAGAATTAC	14 (0.000268%)	
ATCATGAATCATCAGAGCAACGGGCAGAGCCCGCGTCGACCTTTTATCTAATAAATGCGTCCCTTCCATAA<br>GTCGGGGTTTGTTGCACGTATTAGCTCTA	61 (0.001169%)	
ATCATTCAATCGGTAGGAGCGACGGGCGGTGTGTACAAAGGGCAGGGACGTAGTCAACGCGAGCTGATGAC<br>TCGCGCTTACTAGGAATTCCTCGTTGAAG	15 (0.000287%)	
ATCCAACCTAGGCGAGACAAGGGTTCACATTTCGTTCATCACCCTTGGCCGGCTATCGAACAGCCGGACTC<br>CCATCAAAAGATGGTTGCCAAGAACATCT	21 (0.000402%)	
ATCCAACCTAGGCGAGACAAGGGTTCACATTTCGTTCATCACCCTTGGCCGGCTTTCGAACAGCCGGACTC<br>CCATCAAAAGATGGTTGCCAAGAACATCT	27 (0.000517%)	
ATCCAGAGCGTAGGCTTGCTTTGAGCACTCTAATTTCTTCAAAGTAACAGCGCCGGAGGCACGACCCGGCC<br>AATTAAGACCAGGAGCGTATCGCCGACCG	12 (0.000230%)	
ATCCATGCTTTCCAACGAAGCACGCCCATCCAACCTAGGCGAGACAAGGGTTCACATTTCGTTCATCACCC<br>TTGGCCGGCTATCGAACAGCCGGACTCCC	5 (0.000096%)	
ATCCATTGGAGGGCAAGTCTGGTGCCAGCAGCCGCGGTAATTCCAGCTCCAATAGCGTATATTTAAGTTGT<br>TGCAGTTAAAAAGCTCGTAGTTGAACCTT	20 (0.000383%)	
ATCCCATGCTAATGTATCCAGAGCGTAGGCTTGCTTTGAGCACTCTAATTTCTTCAAAGTAACAGCGCCGG<br>AGGCACGACCCGGCCAATTAAGACCAGGA	19 (0.000364%)	
ATCCGCCTAGGCTGTCCCGAGTGTGAGCGAGGTGTGAGTGTCGCCCATGGGCATCGACACCTTGCGGCTAG<br>GAACTGGAACGAGACGGGTAGCAAAGATT	90 (0.001725%)	
ATCCGCCTAGGCTGTCCCGAGTGTGAGCGAGGTGTGAGTGTCGCCCATGGGCATCGACACCTTGCGGCTAG<br>GAACTGGAACGAGACGGGTGGCAAAGATT	100 (0.001916%)	
ATCCGCTAAGGAGTGTGTAACAACTCACCTGCCGAATCAACTAGCCCCGAAAATGGATGGCGCTTAAGCGC<br>GCGACCTATACCCGGCCGTCGGGGCAAGA	4 (0.000077%)	
ATCCGGTTAAAATTCCGGAACCGGGACGTGGCGGTTGACGGCAACGTTAGGGAGTCCGGAGACGTCGGCGG<br>GGGCCTCGGGAAGAGTTATCTTTTCTGTT	19 (0.000364%)	
ATCCGTAACTTCGGGAAAAGGATTGGCTCTGAGGGCTGGGCTCGGGGGTCCCAGTTCCGAACCCGTCGGCT<br>GTCAGCGGACTGCTCGAGCTGCTTCCGCG	3 (0.000057%)	
ATCCGTCGAGTTATCATGAATCATCAGAGCAACGGGCAGAGCCCGCGTCGACCTTTTATCTAATAAATGCG<br>TCCCTTCCATAAGTCGGGGTTTGTTGCAC	4 (0.000077%)	
ATCCTATGATGTTATCCCATGCTAATGTATCCAGAGCGTAGGCTTGCTTTGAGCACTCTAATTTCTTCAAA<br>GTAACAGCGCCGGAGGCACGACCCGGCCA	52 (0.000997%)	
ATCCTATTTTCTGGTAAATTTTCATAATTTTTTGACACCTCTAGCTAGGTCATTTGACCTGATACAACATC<br>GGATTTTCATGGTCTAGTTGGGGCTCCGT	3 (0.000057%)	
ATCCTCGTTAAGGGATTTAGATTGTACTCATTCCAATTACCAGACTCGAAAGAGCCCGGTATTGTTATTTA<br>TTGTCACTACCTCCCCGTGTCAGGATTGG	17 (0.000326%)	
ATCCTTGTTAGAAGACACAAAGCCAAAGACTCATATGGACTTTGGCTACACCATGAAAGCTTTGAGAAGCA<br>AGAAGAAGGTTGGTTAGTGTTTTGGAGTC	10 (0.000192%)	
ATCCTTGTTAGTTTCTTTTCCTCCGCTTATTGATATGCTTAAACTCAGCGGGTAATCCCGCCTGACCTGGG<br>GTCGCTATATGGACTTTGGGTCATCTACA	20 (0.000383%)	
ATCCTTTGCTGATGCGGGACGGAAGCTGGTCTCCCGTGTGTTACCGCACGCGGTTGGCCTAAATCCGAGCC<br>AAGGACGCCTGGAGCGTACCGACATGCGG	8 (0.000153%)	
ATCGAAATCCTATGATGTTATCCCATGCTAATGTATCCAGAGCGTAGGCTTGCTTTGAGCACTCTAATTTC<br>TTCAAAGTAACAGCGCCGGAGGCACGACC	11 (0.000211%)	
ATCGACACCTTGCGGCTAGGAACTGGAACGAGACGGGTGGCAAAGATTTCGAGTAGCACTTCATACTACCG<br>TGGGTTTTTTAAACCTTCCGAGTTTTGTT	10 (0.000192%)	
ATCGACCAGAGGCTGTTCACCTTGGAGACCTGATGCGGTTATGAGTACGACCGGGCGTGAGCGGCACTCGG<br>TCCTCCGGATTTTCAAGGGCCGCCGGGGG	24 (0.000460%)	
ATCGATCAAGTTCACCACCGCATGTCGGTACGCTCCAGGCGTCCTTGGCTCGGATTTAGGCCAACCGCGTG<br>CGGTAACACACGGGAGACCAGCTTCCGTC	7 (0.000134%)	
ATCGATCACGGCAATTCCCCGCCACATCCTCTCAAACGCAATGGAAAGAGAGAAAGGACGAGGTCTTGACC<br>GTCATCTTTTGCCCGAAGGACGGATGAGC	105 (0.002012%)	
ATCGATCCATGCTTTCCAACGAAGCACGCCCATCCAACCTAGGCGAGACAAGGGTTCACATTTCGTTCATC<br>ACCCTTGGCCGGCTATCGAACAGCCGGAC	25 (0.000479%)	

ATCGATCCGCCTAGGCTGTCCCGAGTGTGAGCGAGGTGTGAGTGTCGCCCATGGGCATCGACACCTTGCGG CTAGGAACTGGAACGAGACGGGTAGCAAA	13 (0.000249%)
ATCGATCCGCCTAGGCTGTCCCGAGTGTGAGCGAGGTGTGAGTGTCGCCCATGGGCATCGACACCTTGCGG CTAGGAACTGGAACGAGACGGGTGGCAAA	15 (0.000287%)
ATCGATCCGGTTAAAATTCCGGAACCGGGACGTGGCGGTTGACGGCAACGTTAGGGAGTCCGGAGACGTCG GCGGGGGCCTCGGGAAGAGTTATCTTTTC	15 (0.000287%)
ATCGATCCGTAACTTCGGGAAAAGGATTGGCTCTGAGGGCTGGGCTCGGGGGTCCCAGTTCCGAACCCGTC GGCTGTCAGCGGACTGCTCGAGCTGCTTC	33 (0.000632%)
ATCGATCCGTCGAGTTATCATGAATCATCAGAGCAACGGGCAGAGCCCGCGTCGACCTTTTATCTAATAAA TGCGTCCCTTCCATAAGTCGGGGTTTGTT	17 (0.000326%)
ATCGATCCTCGTTAAGGGATTTAGATTGTACTCATTCCAATTACCAGACTCGAAAGAGCCCGGTATTGTTA TTTATTGTCACTACCTCCCCGTGTCAGGA	41 (0.000786%)
ATCGATCGAAATCCTATGATGTTATCCCATGCTAATGTATCCAGAGCGTAGGCTTGCTTTGAGCACTCTAA TTTCTTCAAAGTAACAGCGCCGGAGGCAC	21 (0.000402%)
ATCGATCGGCGGACCGGATTGCTCCGTTCCGCATCCGACCAGGACGCATCGCCGGCCCCCATCCGCTTCCC TCCCGACAATTTCAAGCACTCTTTGACTC	14 (0.000268%)
ATCGATCGGGTTGCGGTTTAAGTTGTTATACTCAATCATA	19 (0.000146%)
ATCGGGTTGCGGTTTAAGTTGTTATACTCAATCATACACA	120 (0.000920%)
ATCGGTAGGAGCGACGGGCGGTGTGTACAAAGGGCAGGGACGTAGTCAACGCGAGCTGATGACTCGCGCTT ACTAGGAATTCCTCGTTGAAGACCAACAA	64 (0.001227%)
ATCTAATAAATGCGTCCCTTCCATAAGTCGGGGTTTGTTGCACGTATTAGCTCTAGAATTACTACGGTTAT CCGAGTAGTAGTTACCATCAAACAAACTA	7 (0.000134%)
ATCTTAAAAGCCTAAGTAGTGTTTCCTTGTTAGAAGACACAAAGCCAAAGACTCATATGGACTTTGGCTAC ACCATGAAAGCTTTGAGAAGCAAGAAGAA	610 (0.011690%)
ATCTTAAAGGCGTAAGAATTGTATCCTTGTTAAAAGACACAAAGCCAAAGACTCATATGGACTTTGGCTAC ACCATGAAAGCTTTGAGAAGCAAGAAGAA	38 (0.000728%)
ATCTTAAAGGCGTAAGAATTGTATCCTTGTTAGAAGACACAAAGCCAAAGACTCATATGGACTTTGGCTAC ACCATGAAAGCTTTGAGAAGCAAGAAGAA	27 (0.000517%)
ATGAATCATCAGAGCAACGGGCAGAGCCCGCGTCGACCTTTTATCTAATAAATGCGTCCCTTCCATAAGTC GGGGTTTGTTGCACGTATTAGCTCTAGAA	86 (0.001648%)
ATGACATCAAGTCATATTCGACTCCAAAACACTAACCAACCTTCTTCTTGCTTCTCAAAGCTTTCATGGTG TAGCCAAAGTCCATATGAGTCTTTGGCTT	110 (0.002108%)
ATGACTTGATGTCATGTGTATGATTGAGTATAAGAACTTA	268 (0.002054%)
ATGATGTTATCCCATGCTAATGTATCCAGAGCGTAGGCTTGCTTTGAGCACTCTAATTTCTTCAAAGTAAC AGCGCCGGAGGCACGACCCGGCCAATTAA	65 (0.001246%)
ATGATTAACAGGGACAGTCGGGGGCATTCGTATTTCATAGTCAGAGGTGAAATTCTTGGATTTATGAAAGA CGAACAACTGCGAAAGCATTTGCCAAGGA	112 (0.002146%)
ATGCATCATAAGGATACTAAATCCTATTTTCTGGTAAATTTTCATAATTTTTTGACACCTCTAGCTAGGTC ATTTGACCTGATACAACATCGGATTTTCA	3 (0.000057%)
ATGCATGTGTAAGTATGAACGAATTCAGACTGTGAAACTGCGAATGGCTCATTAAATCAGTTATAGTTTGT TTGATGGTAACTACTACTCGGATAACCGT	4 (0.000077%)
ATGCCAGCCGTTCGTTTGCATGTTCCTTGACACTTTTCGTGCCGGGGTTTTGTGATATCCGGAAGCAACGC GCACGACAAGACCGAGATAAAAGCTCCCG	8 (0.000153%)
ATGCCCTTAGATGTTCTGGGCCGCACGCGCGCTACACTGATGTATTCAACGAGTTCACACCTTGGCCGACA GGCCCGGGTAATCTTTGAAATTTCATCGT	10 (0.000192%)
ATGCTAATGTATCCAGAGCGTAGGCTTGCTTTGAGCACTCTAATTTCTTCAAAGTAACAGCGCCGGAGGCA CGACCCGGCCAATTAAGACCAGGAGCGTA	6 (0.000115%)
ATGCTTGTCTCAAAGATTAAGCCATGCATGTGTAAGTATGAACGAATTCAGACTGTGAAACTGCGAATGGC TCATTAAATCAGTTATAGTTTGTTTGATG	20 (0.000383%)
ATGCTTTCCAACGAAGCACGCCCATCCAACCTAGGCGAGACAAGGGTTCACATTTCGTTCATCACCCTTGG CCGGCTATCGAACAGCCGGACTCCCATCA	290 (0.005558%)
ATGGACTTTGGCTACACCATGAAAGCTTTGAGAAGCAAGAAGAAGGTTGGTTAGTGTTTTGGAGTCGAATA TGACTTGATGTCATGTGTATGATTGAGTA	74 (0.001418%)
ATGGCTCATTAAATCAGTTATAGTTTGTTTGATGGTAACTACTACTCGGATAACCGTAGTAATTCTAGAGC TAATACGTGCAACAAACCCCGACTTATGG	3 (0.000057%)
ATGGGCATCGACACCTTGCGGCTAGGAACTGGAACGAGACGGGTGGCAAAGATTTCGAGTAGCACTTCATA CTACCGTGGGTTTTTTAAACCTTCCGAGT	5 (0.000096%)
ATGTATCCAGAGCGTAGGCTTGCTTTGAGCACTCTAATTTCTTCAAAGTAACAGCGCCGGAGGCACGACCC GGCCAATTAAGACCAGGAGCGTATCGCCG	4 (0.000077%)
ATGTCATGTGTATGATTGAGTATAAGAACTTAAACCGCAA	135 (0.001035%)
ATGTCGGTACGCTCCAGGCGTCCTTGGCTCGGATTTAGGCCAACCGCGTGCGGTAACACACGGGAGACCAG CTTCCGTCCCGCATCAGCAAAGGATGGTG	7 (0.000134%)
ATGTGTAAGTATGAACGAATTCAGACTGTGAAACTGCGAATGGCTCATTAAATCAGTTATAGTTTGTTTGA TGGTAACTACTACTCGGATAACCGTAGTA	6 (0.000115%)
ATGTTATCCCATGCTAATGTATCCAGAGCGTAGGCTTGCTTTGAGCACTCTAATTTCTTCAAAGTAACAGC GCCGGAGGCACGACCCGGCCAATTAAGAC	17 (0.000326%)
ATGTTCTGGGCCGCACGCGCGCTACACTGATGTATTCAACGAGTTCACACCTTGGCCGACAGGCCCGGGTA ATCTTTGAAATTTCATCGTGATGGGGATA	7 (0.000134%)
ATTAAATCAGTTATAGTTTGTTTGATGGTAACTACTACTCGGATAACCGTAGTAATTCTAGAGCTAATACG TGCAACAAACCCCGACTTATGGAAGGGAC	33 (0.000632%)
ATTAACAGGGACAGTCGGGGGCATTCGTATTTCATAGTCAGAGGTGAAATTCTTGGATTTATGAAAGACGA ACAACTGCGAAAGCATTTGCCAAGGATGT	110 (0.002108%)
ATTAAGCCATGCATGTGTAAGTATGAACGAATTCAGACTGTGAAACTGCGAATGGCTCATTAAATCAGTTA TAGTTTGTTTGATGGTAACTACTACTCGG	18 (0.000345%)
ATTACCAGACTCGAAAGAGCCCGGTATTGTTATTTATTGTCACTACCTCCCCGTGTCAGGATTGGGTAATT TGCGCGCCTGCTGCCTTCCTTGGATGTGG	7 (0.000134%)
ATTAGGGTTCGATTCCGGAGAGGGAGCCTGAGAAACGGCTACCACATCCAAGGAAGGCAGCAGGCGCGCAA ATTACCCAATCCTGACACGGGGAGGTAGT	5 (0.000096%)
ATTCAATCGGTAGGAGCGACGGGCGGTGTGTACAAAGGGCAGGGACGTAGTCAACGCGAGCTGATGACTCG CGCTTACTAGGAATTCCTCGTTGAAGACC	318 (0.006094%)
ATTCCAATTACCAGACTCGAAAGAGCCCGGTATTGTTATTTATTGTCACTACCTCCCCGTGTCAGGATTGG GTAATTTGCGCGCCTGCTGCCTTCCTTGG	22 (0.000422%)

ATTCCAGCTCCAATAGCGTATATTTAAGTTGTTGCAGTTAAAAAGCTCGTAGTTGAACCTTGGGATGGGTCGGCCGGTCCGCCTTTGGTGTGCATTGGTC	12 (0.000230%)
ATTCCCCGCCACATCCTCTCAAACGCAATGGAAAGAGAGAAAGGACGAGGTCTTGACCGTCATCTTTTGCCCGAAGGACGGATGAGCTTTGGCGGGACTG	16 (0.000307%)
ATTCGACTCCAAAACACTAACCAACCTTCTTCTTGCTTCTCAAAGCTTTCATGGTGTAGCCAAAGTCCATATGAGTCTTTGGCTTTGTGTCTTCTAACAA	126 (0.002415%)
ATTCGTACTGAAAATCAGAATCAAACGAGCTTTTACCCTTTTGTTCCACACGAGATTTCTGTTCTCGTTGAGCTCATCTTAGGACACCTGCGTTATCTTT	5 (0.000096%)
ATTCGTATTTCATAGTCAGAGGTGAAATTCTTGGATTTATGAAAGACGAACAACTGCGAAAGCATTTGCCAAGGATGTTTTCATTAATCAAGAACGAAAG	9 (0.000172%)
ATTGGAGGGCAAGTCTGGTGCCAGCAGCCGCGGTAATTCCAGCTCCAATAGCGTATATTTAAGTTGTTGCAGTTAAAAAGCTCGTAGTTGAACCTTGGGA	205 (0.003929%)
ATTGTACTCATTCCAATTACCAGACTCGAAAGAGCCCGGTATTGTTATTTATTGTCACTACCTCCCCGTGTCAGGATTGGGTAATTTGCGCGCCTGCTGC	17 (0.000326%)
ATTGTATCCTTGTTAGAAGACACAAAGCCAAAGACTCATATGGACTTTGGCTACACCATGAAAGCTTTGAGAAGCAAGAAGAAGGTTGGTTAGTGTTTTG	38 (0.000728%)
ATTGTCAGGTGGGGAGTTTGGCTGGGGCGGCACATCTGTTAAAAGATAACGCAGGTGTCCTAAGATGAGCTCAACGAGAACAGAAATCTCGTGTGGAACA	27 (0.000517%)
ATTGTTCCATCGACCAGAGGCTGTTCACCTTGGAGACCTGATGCGGTTATGAGTACGACCGGGCGTGAGCGGCACTCGGTCCTCCGGATTTTCAAGGGCC	13 (0.000249%)
ATTGTTGGTCTTCAACGAGGAATTCCTAGTAAGCGCGAGTCATCAGCTCGCGTTGACTACGTCCCTGCCCTTTGTACACACCGCCCGTCGCTCCTACCGA	27 (0.000517%)
ATTTAGATTGTACTCATTCCAATTACCAGACTCGAAAGAGCCCGGTATTGTTATTTATTGTCACTACCTCCCCGTGTCAGGATTGGGTAATTTGCGCGCC	9 (0.000172%)
ATTTCATAGTCAGAGGTGAAATTCTTGGATTTATGAAAGACGAACAACTGCGAAAGCATTTGCCAAGGATGTTTTCATTAATCAAGAACGAAAGTTGGGG	9 (0.000172%)
ATTTCGTTCATCACCCTTGGCCGGCTATCGAACAGCCGGACTCCCATCAAAAGATGGTTGCCAAGAACATCTTCGTTACGGTTTGCTAATTCTCGGAATA	18 (0.000345%)
ATTTTCATAATTTTTTGACACCTCTAGCTAGGTCATTTGACCTGATACAACATCGGATTTTCATGGTCTAGTTGGGGCTCCGTGGGCATATTTGATGCAA	5 (0.000096%)
ATTTTCTGGTAAATTTTCATAATTTTTTGACACCTCTAGCTAGGTCATTTGACCTGATACAACATCGGATTTTCATGGTCTAGTTGGGGCTCCGTGGGCA	10 (0.000192%)
CAAAAAGCAACGTCGCTATGAACGCTTGGCTGCCACAAGCCAGTTATCCCTGTGGTAACTTTTCTGACACCTCTAGCTTCAAATTCCGAAGGTCTAAAGG	1216 (0.023304%)
CAAACGAGCTTTTACCCTTTTGTTCCACACGAGATTTCTGTTCTCGTTGAGCTCATCTTAGGACACCTGCGTTATCTTTTAACAGATGTGCCGCCCCAGC	5 (0.000096%)
CAAAGACTCATATGGACTTTGGCTACACCATGAAAGCTTTGAGAAGCAAGAAGAAGGTTGGTTAGTGTTTTGGAGTCGAATATGACTTGATGTCATGTGT	117 (0.002242%)
CAAAGATTAAGCCATGCATGTGTAAGTATGAACGAATTCAGACTGTGAAACTGCGAATGGCTCATTAAATCAGTTATAGTTTGTTTGATGGTAACTACTA	54 (0.001035%)
CAAAGCCAAAGACTCATATGGACTTTGGCTACACCATGAAAGCTTTGAGAAGCAAGAAGAAGGTTGGTTAGTGTTTTGGAGTCGAATATGACTTGATGTC	109 (0.002089%)
CAAAGCTTTCATGGTGTAGCCAAAGTCCATATGAGTCTTTGGCTTTGTGTCTTCTAACAAGGAAACACTACTTAGGCTTATAAGATGCGGTTGCGGTTTA	5 (0.000096%)
CAACCGGATCTTAAAGGCGTAAGAATTGTATCCTTGTTAGAAGACACAAAGCCAAAGACTCATATGGACTTTGGCTACACCATGAAAGCTTTGAGAAGCA	4 (0.000077%)
CAACCTAGGCGAGACAAGGGTTCACATTTCGTTCATCACCCTTGGCCGGCTATCGAACAGCCGGACTCCCATCAAAAGATGGTTGCCAAGAACATCTTCG	5 (0.000096%)
CAACCTAGGCGAGACAAGGGTTCACATTTCGTTCATCACCCTTGGCCGGCTTTCGAACAGCCGGACTCCCATCAAAAGATGGTTGCCAAGAACATCTTCG	10 (0.000192%)
CAACGAAGCACGCCCATCCAACCTAGGCGAGACAAGGGTTCACATTTCGTTCATCACCCTTGGCCGGCTATCGAACAGCCGGACTCCCATCAAAAGATGG	69 (0.001322%)
CAACGAAGCACGCCCATCCAACCTAGGCGAGACAAGGGTTCACATTTCGTTCATCACCCTTGGCCGGCTTTCGAACAGCCGGACTCCCATCAAAAGATGG	75 (0.001437%)
CAACGAGGAATTCCTAGTAAGCGCGAGTCATCAGCTCGCGTTGACTACGTCCCTGCCCTTTGTACACACCGCCCGTCGCTCCTACCGATTGAATGATCGA	7 (0.000134%)
CAACGGGCAGAGCCCGCGTCGACCTTTTATCTAATAAATGCGTCCCTTCCATAAGTCGGGGTTTGTTGCACGTATTAGCTCTAGAATTACTACGGTTATC	31 (0.000594%)
CAACGTCGCTATGAACGCTTGGCTGCCACAAGCCAGTTATCCCTGTGGTAACTTTTCTGACACCTCTAGCTTCAAATTCCGAAGGTCTAAAGGATCGATC	32 (0.000613%)
CAAGGGTTCACATTTCGTTCATCACCCTTGGCCGGCTATCGAACAGCCGGACTCCCATCAAAAGATGGTTGCCAAGAACATCTTCGTTACGGTTTGCTAA	21 (0.000402%)
CAAGGGTTCACATTTCGTTCATCACCCTTGGCCGGCTTTCGAACAGCCGGACTCCCATCAAAAGATGGTTGCCAAGAACATCTTCGTTACGGTTTGCTAA	35 (0.000671%)
CAAGTCATATTCGACTCCAAAACACTAACCAACCTTCTTCTTGCTTCTCAAAGCTTTCATGGTGTAGCCAAAGTCCATATGAGTCTTTGGCTTTGTGTCT	123 (0.002357%)
CAAGTCTGGTGCCAGCAGCCGCGGTAATTCCAGCTCCAATAGCGTATATTTAAGTTGTTGCAGTTAAAAAGCTCGTAGTTGAACCTTGGGATGGGTCGGC	21 (0.000402%)
CAAGTTCACCACCGCATGTCGGTACGCTCCAGGCGTCCTTGGCTCGGATTTAGGCCAACCGCGTGCGGTAACACACGGGAGACCAGCTTCCGTCCCGCAT	19 (0.000364%)
CAATAACAGGTCTGTGATGCCCTTAGATGTTCTGGGCCGCACGCGCGCTACACTGATGTATTCAACGAGTTCACACCTTGGCCGACAGGCCCGGGTAATC	61 (0.001169%)
CAATCATACACATGACAACAAGTCATATTCGACTCCAAAACACTAACCAACCTTCTTCTTGCTTCTCAAAGCTTTCATGGTGTAGCCAAAGTCCATATGA	34 (0.000652%)
CAATCATACACATGACATCAAGTCATATTCGACTCCAAAACACTAACCAACCTTCTTCTTGCTTCTCAAAGCTTTCATGGTGTAGCCAAAGTCCATATGA	163 (0.003124%)
CAATCGGTAGGAGCGACGGGCGGTGTGTACAAAGGGCAGGGACGTAGTCAACGCGAGCTGATGACTCGCGCTTACTAGGAATTCCTCGTTGAAGACCAAC	88 (0.001686%)
CAATTACCAGACTCGAAAGAGCCCGGTATTGTTATTTATTGTCACTACCTCCCCGTGTCAGGATTGGGTAATTTGCGCGCCTGCTGCCTTCCTTGGATGT	19 (0.000364%)
CAATTCCCCGCCACATCCTCTCAAACGCAATGGAAAGAGAGAAAGGACGAGGTCTTGACCGTCATCTTTTGCCCGAAGGACGGATGAGCTTTGGCGGGAC	27 (0.000517%)
CACAAAGCCAAAGACTCATATGGACTTTGGCTACACCATGAAAGCTTTGAGAAGCAAGAAGAAGGTTGGTTAGTGTTTTGGAGTCGAATATGACTTGATC	9 (0.000172%)
CACAAAGCCAAAGACTCATATGGACTTTGGCTACACCATGAAAGCTTTGAGAAGCAAGAAGAAGGTTGGTTAGTGTTTTGGAGTCGAATATGACTTGATG	36 (0.000690%)

CACATCCTCTCAAACGCAATGGAAAGAGAGAAAGGACGAGGTCTTGACCGTCATCTTTTGCCCGAAGGACGGATGAGCTTTGGCGGGACTGAATCACTTC	4 (0.000077%)	
CACATCTGTTAAAAGATAACGCAGGTGTCCTAAGATGAGCTCAACGAGAACAGAAATCTCGTGTGGAACAAAAGGGTAAAAGCTCGTTTGATTCTGATTT	9 (0.000172%)	
CACATGACATCAAGTCATATTCGACTCCAAAACACTAACCAACCTTCTTCTTGCTTCTCAAAGCTTTCATGGTGTAGCCAAAGTCCATATGAGTCTTTGG	41 (0.000786%)	
CACATTTCGTTCATCACCCTTGGCCGGCTATCGAACAGCCGGACTCCCATCAAAAGATGGTTGCCAAGAACATCTTCGTTACGGTTTGCTAATTCTCGGA	5 (0.000096%)	
CACATTTCGTTCATCACCCTTGGCCGGCTTTCGAACAGCCGGACTCCCATCAAAAGATGGTTGCCAAGAACATCTTCGTTACGGTTTGCTAATTCTCGGA	11 (0.000211%)	
CACCACCGCATGTCGGTACGCTCCAGGCGTCCTTGGCTCGGATTTAGGCCAACCGCGTGCGGTAACACACGGGAGACCAGCTTCCGTCCCGCATCAGCAA	21 (0.000402%)	
CACCATCCTTTGCTGATGCGGGACGGAAGCTGGTCTCCCGTGTGTTACCGCACGCGGTTGGCCTAAATCCGAGCCAAGGACGCCTGGAGCGTACCGACAT	7 (0.000134%)	
CACCCGTTGACTCGCACACATGTCAGACTCCTTGGTCCGTGTTTCAAGACGGGTCGAATGGGGAGCCCACAGGCCGACGCCCGGAGCACGCTGATGCCGA	6 (0.000115%)	
CACCCTAACGCCTCGAAGAACTAATGGCAGCCCACGCAAGGCAAGCCCATTCTCCTCGACGATTCAGCAGTTTTTGTCCGAGAACTGCTGAGAAAACTCG	11 (0.000211%)	
CACCGCATGTCGGTACGCTCCAGGCGTCCTTGGCTCGGATTTAGGCCAACCGCGTGCGGTAACACACGGGAGACCAGCTTCCGTCCCGCATCAGCAAAGG	7 (0.000134%)	
CACGCCCATCCAACCTAGGCGAGACAAGGGTTCACATTTCGTTCATCACCCTTGGCCGGCTATCGAACAGCCGGACTCCCATCAAAAGATGGTTGCCAAG	26 (0.000498%)	
CACGCCCATCCAACCTAGGCGAGACAAGGGTTCACATTTCGTTCATCACCCTTGGCCGGCTTTCGAACAGCCGGACTCCCATCAAAAGATGGTTGCCAAG	33 (0.000632%)	
CACGCGCCTAACGGCGTGCCTCGGCATCAGCGTGCTCCGGGCGTCGGCCTGTGGGCTCCCCATTCGACCCGTCTTGAAACACGGACCAAGGAGTCTGACA	76 (0.001457%)	
CACGCTTTCACGGTTCGTATTCGTACTGAAAATCAGAATCAAACGAGCTTTTACCCTTTTGTTCCACACGAGATTTCTGTTCTCGTTGAGCTCATCTTAG	111 (0.002127%)	
CACGGCAATTCCCCGCCACATCCTCTCAAACGCAATGGAAAGAGAGAAAGGACGAGGTCTTGACCGTCATCTTTTGCCCGAAGGACGGATGAGCTTTGGC	7 (0.000134%)	
CACGGTTCGTATTCGTACTGAAAATCAGAATCAAACGAGCTTTTACCCTTTTGTTCCACACGAGATTTCTGTTCTCGTTGAGCTCATCTTAGGACACCTG	10 (0.000192%)	
CAGAATCAAACGAGCTTTTACCCTTTTGTTCCACACGAGATTTCTGTTCTCGTTGAGCTCATCTTAGGACACCTGCGTTATCTTTTAACAGATGTGCCGC	18 (0.000345%)	
CAGACTCGAAAGAGCCCGGTATTGTTATTTATTGTCACTACCTCCCCGTGTCAGGATTGGGTAATTTGCGCGCCTGCTGCCTTCCTTGGATGTGGTAGCC	19 (0.000364%)	
CAGAGCAACGGGCAGAGCCCGCGTCGACCTTTTATCTAATAAATGCGTCCCTTCCATAAGTCGGGGTTTGTTGCACGTATTAGCTCTAGAATTACTACGG	16 (0.000307%)	
CAGAGCCCGCGTCGACCTTTTATCTAATAAATGCGTCCCTTCCATAAGTCGGGGTTTGTTGCACGTATTAGCTCTAGAATTACTACGGTTATCCGAGTAG	7 (0.000134%)	
CAGAGCGTAGGCTTGCTTTGAGCACTCTAATTTCTTCAAAGTAACAGCGCCGGAGGCACGACCCGGCCAATTAAGACCAGGAGCGTATCGCCGACCGAAG	39 (0.000747%)	
CAGAGGCTGTTCACCTTGGAGACCTGATGCGGTTATGAGTACGACCGGGCGTGAGCGGCACTCGGTCCTCCGGATTTTCAAGGGCCGCCGGGGGCGCACC	12 (0.000230%)	
CAGAGGTGAAATTCTTGGATTTATGAAAGACGAACAACTGCGAAAGCATTTGCCAAGGATGTTTTCATTAATCAAGAACGAAAGTTGGGGGCTCGAAGAC	18 (0.000345%)	
CAGCACGCGCCTAACGGCGTGCCTCGGCATCAGCGTGCTCCGGGCGTCGGCCTGTGGGCTCCCCATTCGACCCGTCTTGAAACACGGACCAAGGAGTCTG	75 (0.001437%)	
CAGCAGCCGCGGTAATTCCAGCTCCAATAGCGTATATTTAAGTTGTTGCAGTTAAAAAGCTCGTAGTTGAACCTTGGGATGGGTCGGCCGGTCCGCCTTT	27 (0.000517%)	
CAGCCGCGGTAATTCCAGCTCCAATAGCGTATATTTAAGTTGTTGCAGTTAAAAAGCTCGTAGTTGAACCTTGGGATGGGTCGGCCGGTCCGCCTTTGGT	10 (0.000192%)	
CAGCGTGCTCCGGGCGTCGGCCTGTGGGCTCCCCATTCGACCCGTCTTGAAACACGGACCAAGGAGTCTGACATGTGTGCGAGTCAACGGGTGAGTAAAC	9 (0.000172%)	
CAGGGACAGTCGGGGGCATTCGTATTTCATAGTCAGAGGTGAAATTCTTGGATTTATGAAAGACGAACAACTGCGAAAGCATTTGCCAAGGATGTTTTCA	29 (0.000556%)	
CAGGTCTGTGATGCCCTTAGATGTTCTGGGCCGCACGCGCGCTACACTGATGTATTCAACGAGTTCACACCTTGGCCGACAGGCCCGGGTAATCTTTGAA	11 (0.000211%)	
CAGGTGGGGAGTTTGGCTGGGGCGGCACATCTGTTAAAAGATAACGCAGGTGTCCTAAGATGAGCTCAACGAGAACAGAAATCTCGTGTGGAACAAAAGG	36 (0.000690%)	
CAGTAGTCATATGCTTGTCTCAAAGATTAAGCCATGCATGTGTAAGTATGAACGAATTCAGACTGTGAAACTGCGAATGGCTCATTAAATCAGTTATAGT	136 (0.002606%)	
CAGTCGGGGGCATTCGTATTTCATAGTCAGAGGTGAAATTCTTGGATTTATGAAAGACGAACAACTGCGAAAGCATTTGCCAAGGATGTTTTCATTAATC	4 (0.000077%)	
CATAAGGATACTAAATCCTATTTTCTGGTAAATTTTCATAATTTTTTGACACCTCTAGCTAGGTCATTTGACCTGATACAACATCGGATTTTCATGGTCT	24 (0.000460%)	
CATACACATGACATCAAGTCATATTCGACTCCAAAACACTAACCAACCTTCTTCTTGCTTCTCAAAGCTTTCATGGTGTAGCCAAAGTCCATATGAGTCT	47 (0.000901%)	
CATAGTCAGAGGTGAAATTCTTGGATTTATGAAAGACGAACAACTGCGAAAGCATTTGCCAAGGATGTTTTCATTAATCAAGAACGAAAGTTGGGGGCTC	17 (0.000326%)	
CATATGCTTGTCTCAAAGATTAAGCCATGCATGTGTAAGTATGAACGAATTCAGACTGTGAAACTGCGAATGGCTCATTAAATCAGTTATAGTTTGTTTG	52 (0.000997%)	
CATATGGACTTTGGCTACACCATGAAAGCTTTGAGAAGCAAGAAGAAGGTTGGTTAGTGTTTTGGAGTCGAATATGACTTGATGTCATGTGTATGATTGA	12 (0.000230%)	
CATATTCGACTCCAAAACACTAACCAACCTTCTTCTTGCTTCTCAAAGCTTTCATGGTGTAGCCAAAGTCCATATGAGTCTTTGGCTTTGTGTCTTCTAA	80 (0.001533%)	
CATCAAGTCATATTCGACTCCAAAACACTAACCAACCTTCTTCTTGCTTCTCAAAGCTTTCATGGTGTAGCCAAAGTCCATATGAGTCTTTGGCTTTGTG	50 (0.000958%)	
CATCAGAGCAACGGGCAGAGCCCGCGTCGACCTTTTATCTAATAAATGCGTCCCTTCCATAAGTCGGGGTTTGTTGCACGTATTAGCTCTAGAATTACTA	44 (0.000843%)	
CATCAGCGTGCTCCGGGCGTCGGCCTGTGGGCTCCCCATTCGACCCGTCTTGAAACACGGACCAAGGAGTCTGACATGTGTGCGAGTCAACGGGTGAGTA	10 (0.000192%)	
CATCCAACCTAGGCGAGACAAGGGTTCACATTTCGTTCATCACCCTTGGCCGGCTATCGAACAGCCGGACTCCCATCAAAAGATGGTTGCCAAGAACATC	6 (0.000115%)	
CATCCAACCTAGGCGAGACAAGGGTTCACATTTCGTTCATCACCCTTGGCCGGCTTTCGAACAGCCGGACTCCCATCAAAAGATGGTTGCCAAGAACATC	6 (0.000115%)	

CATCCTTTGCTGATGCGGGACGGAAGCTGGTCTCCCGTGTGTTACCGCACGCGGTTGGCCTAAATCCGAGCCAAGGACGCCTGGAGCGTACCGACATGCG	126 (0.002415%)	
CATCGACACCTTGCGGCTAGGAACTGGAACGAGACGGGTGGCAAAGATTTCGAGTAGCACTTCATACTACCGTGGGTTTTTTAAACCTTCCGAGTTTTGT	5 (0.000096%)	
CATCGACCAGAGGCTGTTCACCTTGGAGACCTGATGCGGTTATGAGTACGACCGGGCGTGAGCGGCACTCGGTCCTCCGGATTTTCAAGGGCCGCCGGGG	4 (0.000077%)	
CATGAATCATCAGAGCAACGGGCAGAGCCCGCGTCGACCTTTTATCTAATAAATGCGTCCCTTCCATAAGTCGGGGTTTGTTGCACGTATTAGCTCTAGA	163 (0.003124%)	
CATGACATCAAGTCATATTCGACTCCAAAACACTAACCAACCTTCTTCTTGCTTCTCAAAGCTTTCATGGTGTAGCCAAAGTCCATATGAGTCTTTGGCT	33 (0.000632%)	
CATGCATCATAAGGATACTAAATCCTATTTTCTGGTAAATTTTCATAATTTTTTGACACCTCTAGCTAGGTCATTTGACCTGATACAACATCGGATTTTC	3 (0.000057%)	
CATGCATGTGTAAGTATGAACGAATTCAGACTGTGAAACTGCGAATGGCTCATTAAATCAGTTATAGTTTGTTTGATGGTAACTACTACTCGGATAACCG	7 (0.000134%)	
CATGCTAATGTATCCAGAGCGTAGGCTTGCTTTGAGCACTCTAATTTCTTCAAAGTAACAGCGCCGGAGGCACGACCCGGCCAATTAAGACCAGGAGCGT	8 (0.000153%)	
CATGCTTTCCAACGAAGCACGCCCATCCAACCTAGGCGAGACAAGGGTTCACATTTCGTTCATCACCCTTGGCCGGCTATCGAACAGCCGGACTCCCATC	15 (0.000287%)	
CATGGGCATCGACACCTTGCGGCTAGGAACTGGAACGAGACGGGTGGCAAAGATTTCGAGTAGCACTTCATACTACCGTGGGTTTTTTAAACCTTCCGAG	7 (0.000134%)	
CATGTCGGTACGCTCCAGGCGTCCTTGGCTCGGATTTAGGCCAACCGCGTGCGGTAACACACGGGAGACCAGCTTCCGTCCCGCATCAGCAAAGGATGGT	10 (0.000192%)	
CATGTGTAAGTATGAACGAATTCAGACTGTGAAACTGCGAATGGCTCATTAAATCAGTTATAGTTTGTTTGATGGTAACTACTACTCGGATAACCGTAGT	3 (0.000057%)	
CATTAAATCAGTTATAGTTTGTTTGATGGTAACTACTACTCGGATAACCGTAGTAATTCTAGAGCTAATACGTGCAACAAACCCCGACTTATGGAAGGGA	12 (0.000230%)	
CATTCAATCGGTAGGAGCGACGGGCGGTGTGTACAAAGGGCAGGGACGTAGTCAACGCGAGCTGATGACTCGCGCTTACTAGGAATTCCTCGTTGAAGAC	13 (0.000249%)	
CATTCCAATTACCAGACTCGAAAGAGCCCGGTATTGTTATTTATTGTCACTACCTCCCCGTGTCAGGATTGGGTAATTTGCGCGCCTGCTGCCTTCCTTG	3 (0.000057%)	
CATTCGTATTTCATAGTCAGAGGTGAAATTCTTGGATTTATGAAAGACGAACAACTGCGAAAGCATTTGCCAAGGATGTTTTCATTAATCAAGAACGAAA	3 (0.000057%)	
CATTGGAGGGCAAGTCTGGTGCCAGCAGCCGCGGTAATTCCAGCTCCAATAGCGTATATTTAAGTTGTTGCAGTTAAAAAGCTCGTAGTTGAACCTTGGG	28 (0.000537%)	
CATTGTCAGGTGGGGAGTTTGGCTGGGGCGGCACATCTGTTAAAAGATAACGCAGGTGTCCTAAGATGAGCTCAACGAGAACAGAAATCTCGTGTGGAAC	14 (0.000268%)	
CATTGTTCCATCGACCAGAGGCTGTTCACCTTGGAGACCTGATGCGGTTATGAGTACGACCGGGCGTGAGCGGCACTCGGTCCTCCGGATTTTCAAGGGC	25 (0.000479%)	
CCAAAGACTCATATGGACTTTGGCTACACCATGAAAGCTTTGAGAAGCAAGAAGAAGGTTGGTTAGTGTTTTGGAGTCGAATATGACTTGATGTCATGTG	161 (0.003085%)	
CCAACCTAGGCGAGACAAGGGTTCACATTTCGTTCATCACCCTTGGCCGGCTATCGAACAGCCGGACTCCCATCAAAAGATGGTTGCCAAGAACATCTTC	24 (0.000460%)	
CCAACCTAGGCGAGACAAGGGTTCACATTTCGTTCATCACCCTTGGCCGGCTTTCGAACAGCCGGACTCCCATCAAAAGATGGTTGCCAAGAACATCTTC	18 (0.000345%)	
CCAACGAAGCACGCCCATCCAACCTAGGCGAGACAAGGGTTCACATTTCGTTCATCACCCTTGGCCGGCTATCGAACAGCCGGACTCCCATCAAAAGATG	13 (0.000249%)	
CCAACGAAGCACGCCCATCCAACCTAGGCGAGACAAGGGTTCACATTTCGTTCATCACCCTTGGCCGGCTTTCGAACAGCCGGACTCCCATCAAAAGATG	11 (0.000211%)	
CCAATTACCAGACTCGAAAGAGCCCGGTATTGTTATTTATTGTCACTACCTCCCCGTGTCAGGATTGGGTAATTTGCGCGCCTGCTGCCTTCCTTGGATG	7 (0.000134%)	
CCACATCCTCTCAAACGCAATGGAAAGAGAGAAAGGACGAGGTCTTGACCGTCATCTTTTGCCCGAAGGACGGATGAGCTTTGGCGGGACTGAATCACTT	3 (0.000057%)	
CCACCCTAACGCCTCGAAGAACTAATGGCAGCCCACGCAAGGCAAGCCCATTCTCCTCGACGATTCAGCAGTTTTTGTCCGAGAACTGCTGAGAAAACTC	5 (0.000096%)	
CCACCGCATGTCGGTACGCTCCAGGCGTCCTTGGCTCGGATTTAGGCCAACCGCGTGCGGTAACACACGGGAGACCAGCTTCCGTCCCGCATCAGCAAAG	7 (0.000134%)	
CCACGCTTTCACGGTTCGTATTCGTACTGAAAATCAGAATCAAACGAGCTTTTACCCTTTTGTTCCACACGAGATTTCTGTTCTCGTTGAGCTCATCTTA	56 (0.001073%)	
CCAGACTCGAAAGAGCCCGGTATTGTTATTTATTGTCACTACCTCCCCGTGTCAGGATTGGGTAATTTGCGCGCCTGCTGCCTTCCTTGGATGTGGTAGC	13 (0.000249%)	
CCAGAGCGTAGGCTTGCTTTGAGCACTCTAATTTCTTCAAAGTAACAGCGCCGGAGGCACGACCCGGCCAATTAAGACCAGGAGCGTATCGCCGACCGAA	19 (0.000364%)	
CCAGAGGCTGTTCACCTTGGAGACCTGATGCGGTTATGAGTACGACCGGGCGTGAGCGGCACTCGGTCCTCCGGATTTTCAAGGGCCGCCGGGGGCGCAC	13 (0.000249%)	
CCAGCAGCCGCGGTAATTCCAGCTCCAATAGCGTATATTTAAGTTGTTGCAGTTAAAAAGCTCGTAGTTGAACCTTGGGATGGGTCGGCCGGTCCGCCTT	18 (0.000345%)	
CCAGCCGTTCGTTTGCATGTTCCTTGACACTTTTCGTGCCGGGGTTTTGTGATATCCGGAAGCAACGCGCACGACAAGACCGAGATAAAAGCTCCCGATC	5 (0.000096%)	
CCAGTAGTCATATGCTTGTCTCAAAGATTAAGCCATGCATGTGTAAGTATGAACGAATTCAGACTGTGAAACTGCGAATGGCTCATTAAATCAGTTATAG	92 (0.001763%)	
CCATCCTTTGCTGATGCGGGACGGAAGCTGGTCTCCCGTGTGTTACCGCACGCGGTTGGCCTAAATCCGAGCCAAGGACGCCTGGAGCGTACCGACATGC	8 (0.000153%)	
CCATGCTAATGTATCCAGAGCGTAGGCTTGCTTTGAGCACTCTAATTTCTTCAAAGTAACAGCGCCGGAGGCACGACCCGGCCAATTAAGACCAGGAGCG	5 (0.000096%)	
CCATGCTTTCCAACGAAGCACGCCCATCCAACCTAGGCGAGACAAGGGTTCACATTTCGTTCATCACCCTTGGCCGGCTATCGAACAGCCGGACTCCCAT	27 (0.000517%)	
CCATGGGCATCGACACCTTGCGGCTAGGAACTGGAACGAGACGGGTGGCAAAGATTTCGAGTAGCACTTCATACTACCGTGGGTTTTTTAAACCTTCCGA	8 (0.000153%)	
CCATTGGAGGGCAAGTCTGGTGCCAGCAGCCGCGGTAATTCCAGCTCCAATAGCGTATATTTAAGTTGTTGCAGTTAAAAAGCTCGTAGTTGAACCTTGG	12 (0.000230%)	
CCCATCCAACCTAGGCGAGACAAGGGTTCACATTTCGTTCATCACCCTTGGCCGGCTATCGAACAGCCGGACTCCCATCAAAAGATGGTTGCCAAGAACA	15 (0.000287%)	
CCCATCCAACCTAGGCGAGACAAGGGTTCACATTTCGTTCATCACCCTTGGCCGGCTTTCGAACAGCCGGACTCCCATCAAAAGATGGTTGCCAAGAACA	21 (0.000402%)	
CCCATGCTAATGTATCCAGAGCGTAGGCTTGCTTTGAGCACTCTAATTTCTTCAAAGTAACAGCGCCGGAGGCACGACCCGGCCAATTAAGACCAGGAGC	13 (0.000249%)	

CCCATGGGCATCGACACCTTGCGGCTAGGAACTGGAACGAGACGGGTGGCAAAGATTTCGAGTAGCACTTCATACTACCGTGGGTTTTTTAAACCTTCCG	11 (0.000211%)	
CCCCGCCACATCCTCTCAAACGCAATGGAAAGAGAGAAAGGACGAGGTCTTGACCGTCATCTTTTGCCCGAAGGACGGATGAGCTTTGGCGGGACTGAAT	6 (0.000115%)	
CCCGAGTGTGAGCGAGGTGTGAGTGTCGCCCATGGGCATCGACACCTTGCGGCTAGGAACTGGAACGAGACGGGTAGCAAAGATTTCGAGTAGCACTTCA	10 (0.000192%)	
CCCGAGTGTGAGCGAGGTGTGAGTGTCGCCCATGGGCATCGACACCTTGCGGCTAGGAACTGGAACGAGACGGGTGGCAAAGATTTCGAGTAGCACTTCA	24 (0.000460%)	
CCCGCCACATCCTCTCAAACGCAATGGAAAGAGAGAAAGGACGAGGTCTTGACCGTCATCTTTTGCCCGAAGGACGGATGAGCTTTGGCGGGACTGAATC	6 (0.000115%)	
CCCGCGTCGACCTTTTATCTAATAAATGCGTCCCTTCCATAAGTCGGGGTTTGTTGCACGTATTAGCTCTAGAATTACTACGGTTATCCGAGTAGTAGTT	40 (0.000767%)	
CCCGGCCGTCGGGGCAAGAGCCAGGCCTCGATGAGTAGGAGGGCGCGGCGGTCGCTGCAAAACCTAGGGCGCGAGCCCGGGCGGAGCGGCCGTCGGTGCA	19 (0.000364%)	
CCCTAACGCCTCGAAGAACTAATGGCAGCCCACGCAAGGCAAGCCCATTCTCCTCGACGATTCAGCAGTTTTTGTCCGAGAACTGCTGAGAAAACTCGGA	10 (0.000192%)	
CCCTCACCATCCTTTGCTGATGCGGGACGGAAGCTGGTCTCCCGTGTGTTACCGCACGCGGTTGGCCTAAATCCGAGCCAAGGACGCCTGGAGCGTACCG	20 (0.000383%)	
CCCTGTTGAGCTTGACTCTAGTCCGACTTTGTGAAATGACTTGAGAGGTGTAGGATAAGTGGGAGCTTCGGCGCAAGTGAAATACCACTACTTTTAACGT	13 (0.000249%)	
CCCTTAGATGTTCTGGGCCGCACGCGCGCTACACTGATGTATTCAACGAGTTCACACCTTGGCCGACAGGCCCGGGTAATCTTTGAAATTTCATCGTGAT	5 (0.000096%)	
CCCTTGCCTACATTGTTCCATCGACCAGAGGCTGTTCACCTTGGAGACCTGATGCGGTTATGAGTACGACCGGGCGTGAGCGGCACTCGGTCCTCCGGAT	35 (0.000671%)	
CCGACTTCCCTTGCCTACATTGTTCCATCGACCAGAGGCTGTTCACCTTGGAGACCTGATGCGGTTATGAGTACGACCGGGCGTGAGCGGCACTCGGTCC	48 (0.000920%)	
CCGACTTTGTGAAATGACTTGAGAGGTGTAGGATAAGTGGGAGCTTCGGCGCAAGTGAAATACCACTACTTTTAACGTTATTTTACTTACTCCGTGAATC	19 (0.000364%)	
CCGAGTGTGAGCGAGGTGTGAGTGTCGCCCATGGGCATCGACACCTTGCGGCTAGGAACTGGAACGAGACGGGTAGCAAAGATTTCGAGTAGCACTTCAT	12 (0.000230%)	
CCGAGTGTGAGCGAGGTGTGAGTGTCGCCCATGGGCATCGACACCTTGCGGCTAGGAACTGGAACGAGACGGGTGGCAAAGATTTCGAGTAGCACTTCAT	25 (0.000479%)	
CCGCCACATCCTCTCAAACGCAATGGAAAGAGAGAAAGGACGAGGTCTTGACCGTCATCTTTTGCCCGAAGGACGGATGAGCTTTGGCGGGACTGAATCA	11 (0.000211%)	
CCGCCTAGGCTGTCCCGAGTGTGAGCGAGGTGTGAGTGTCGCCCATGGGCATCGACACCTTGCGGCTAGGAACTGGAACGAGACGGGTAGCAAAGATTTC	31 (0.000594%)	
CCGCCTAGGCTGTCCCGAGTGTGAGCGAGGTGTGAGTGTCGCCCATGGGCATCGACACCTTGCGGCTAGGAACTGGAACGAGACGGGTGGCAAAGATTTC	62 (0.001188%)	
CCGCGGTAATTCCAGCTCCAATAGCGTATATTTAAGTTGTTGCAGTTAAAAAGCTCGTAGTTGAACCTTGGGATGGGTCGGCCGGTCCGCCTTTGGTGTG	6 (0.000115%)	
CCGCGTCGACCTTTTATCTAATAAATGCGTCCCTTCCATAAGTCGGGGTTTGTTGCACGTATTAGCTCTAGAATTACTACGGTTATCCGAGTAGTAGTTA	12 (0.000230%)	
CCGCTAAGGAGTGTGTAACAACTCACCTGCCGAATCAACTAGCCCCGAAAATGGATGGCGCTTAAGCGCGCGACCTATACCCGGCCGTCGGGGCAAGAGC	32 (0.000613%)	
CCGGATTGCTCCGTTCCGCATCCGACCAGGACGCATCGCCGGCCCCCATCCGCTTCCCTCCCGACAATTTCAAGCACTCTTTGACTCTCTTTTCAAAGTC	34 (0.000652%)	
CCGGCCGTCGGGGCAAGAGCCAGGCCTCGATGAGTAGGAGGGCGCGGCGGTCGCTGCAAAACCTAGGGCGCGAGCCCGGGCGGAGCGGCCGTCGGTGCAG	18 (0.000345%)	
CCGGGCGTCGGCCTGTGGGCTCCCCATTCGACCCGTCTTGAAACACGGACCAAGGAGTCTGACATGTGTGCGAGTCAACGGGTGAGTAAACCCGTAAGGC	13 (0.000249%)	
CCGGGGAGGCGAATGCCAGCCGTTCGTTTGCATGTTCCTTGACACTTTTCGTGCCGGGGTTTTGTGATATCCGGAAGCAACGCGCACGACAAGACCGAGA	14 (0.000268%)	
CCGTCGAGTTATCATGAATCATCAGAGCAACGGGCAGAGCCCGCGTCGACCTTTTATCTAATAAATGCGTCCCTTCCATAAGTCGGGGTTTGTTGCACGT	28 (0.000537%)	
CCGTCTCCGGGGAGGCGAATGCCAGCCGTTCGTTTGCATGTTCCTTGACACTTTTCGTGCCGGGGTTTTGTGATATCCGGAAGCAACGCGCACGACAAGA	10 (0.000192%)	
CCTAACGCCTCGAAGAACTAATGGCAGCCCACGCAAGGCAAGCCCATTCTCCTCGACGATTCAGCAGTTTTTGTCCGAGAACTGCTGAGAAAACTCGGAA	18 (0.000345%)	
CCTAACGGCGTGCCTCGGCATCAGCGTGCTCCGGGCGTCGGCCTGTGGGCTCCCCATTCGACCCGTCTTGAAACACGGACCAAGGAGTCTGACATGTGTG	4 (0.000077%)	
CCTAAGTAGTGTTTCCTTGTTAGAAGACACAAAGCCAAAGACTCATATGGACTTTGGCTACACCATGAAAGCTTTGAGAAGCAAGAAGAAGGTTGGTTAG	53 (0.001016%)	
CCTACATTGTTCCATCGACCAGAGGCTGTTCACCTTGGAGACCTGATGCGGTTATGAGTACGACCGGGCGTGAGCGGCACTCGGTCCTCCGGATTTTCAA	13 (0.000249%)	
CCTAGGCGAGACAAGGGTTCACATTTCGTTCATCACCCTTGGCCGGCTATCGAACAGCCGGACTCCCATCAAAAGATGGTTGCCAAGAACATCTTCGTTA	11 (0.000211%)	
CCTAGGCGAGACAAGGGTTCACATTTCGTTCATCACCCTTGGCCGGCTTTCGAACAGCCGGACTCCCATCAAAAGATGGTTGCCAAGAACATCTTCGTTA	12 (0.000230%)	
CCTAGGCTGTCCCGAGTGTGAGCGAGGTGTGAGTGTCGCCCATGGGCATCGACACCTTGCGGCTAGGAACTGGAACGAGACGGGTAGCAAAGATTTCGAG	45 (0.000862%)	
CCTAGGCTGTCCCGAGTGTGAGCGAGGTGTGAGTGTCGCCCATGGGCATCGACACCTTGCGGCTAGGAACTGGAACGAGACGGGTGGCAAAGATTTCGAG	59 (0.001131%)	
CCTATACCCGGCCGTCGGGGCAAGAGCCAGGCCTCGATGAGTAGGAGGGCGCGGCGGTCGCTGCAAAACCTAGGGCGCGAGCCCGGGCGGAGCGGCCGTC	19 (0.000364%)	
CCTATGATGTTATCCCATGCTAATGTATCCAGAGCGTAGGCTTGCTTTGAGCACTCTAATTTCTTCAAAGTAACAGCGCCGGAGGCACGACCCGGCCAAT	24 (0.000460%)	
CCTATTTTCTGGTAAATTTTCATAATTTTTTGACACCTCTAGCTAGGTCATTTGACCTGATACAACATCGGATTTTCATGGTCTAGTTGGGGCTCCGTGG	4 (0.000077%)	
CCTCACCATCCTTTGCTGATGCGGGACGGAAGCTGGTCTCCCGTGTGTTACCGCACGCGGTTGGCCTAAATCCGAGCCAAGGACGCCTGGAGCGTACCGA	81 (0.001552%)	
CCTCCGCTTATTGATATGCTTAAACTCAGCGGGTAATCCCGCCTGACCTGGGGTCGCTATATGGACTTTGGGTCATCTACAGCTTCCGGACAAGAGCGAC	3 (0.000057%)	
CCTCGAAGAACTAATGGCAGCCCACGCAAGGCAAGCCCATTCTCCTCGACGATTCAGCAGTTTTTGTCCGAGAACTGCTGAGAAAACTCGGAAAAAGGCA	12 (0.000230%)	
CCTCGGCATCAGCGTGCTCCGGGCGTCGGCCTGTGGGCTCCCCATTCGACCCGTCTTGAAACACGGACCAAGGAGTCTGACATGTGTGCGAGTCAACGGG	7 (0.000134%)	

CCTCGTTAAGGGATTTAGATTGTACTCATTCCAATTACCAGACTCGAAAGAGCCCGGTATTGTTATTTATTGTCACTACCTCCCCGTGTCAGGATTGGGT	209 (0.004005%)	
CCTGTTGAGCTTGACTCTAGTCCGACTTTGTGAAATGACTTGAGAGGTGTAGGATAAGTGGGAGCTTCGGCGCAAGTGAAATACCACTACTTTTAACGTT	7 (0.000134%)	
CCTTAGATGTTCTGGGCCGCACGCGCGCTACACTGATGTATTCAACGAGTTCACACCTTGGCCGACAGGCCCGGGTAATCTTTGAAATTTCATCGTGATG	7 (0.000134%)	
CCTTGCCTACATTGTTCCATCGACCAGAGGCTGTTCACCTTGGAGACCTGATGCGGTTATGAGTACGACCGGGCGTGAGCGGCACTCGGTCCTCCGGATT	12 (0.000230%)	
CCTTGTTAGAAGACACAAAGCCAAAGACTCATATGGACTTTGGCTACACCATGAAAGCTTTGAGAAGCAAGAAGAAGGTTGGTTAGTGTTTTGGAGTCGA	49 (0.000939%)	
CCTTGTTAGTTTCTTTTCCTCCGCTTATTGATATGCTTAAACTCAGCGGGTAATCCCGCCTGACCTGGGGTCGCTATATGGACTTTGGGTCATCTACAGC	11 (0.000211%)	
CCTTTGCTGATGCGGGACGGAAGCTGGTCTCCCGTGTGTTACCGCACGCGGTTGGCCTAAATCCGAGCCAAGGACGCCTGGAGCGTACCGACATGCGGTG	7 (0.000134%)	
CGAAAGAGCCCGGTATTGTTATTTATTGTCACTACCTCCCCGTGTCAGGATTGGGTAATTTGCGCGCCTGCTGCCTTCCTTGGATGTGGTAGCCGTTTCT	19 (0.000364%)	
CGAAATCCGCTAAGGAGTGTGTAACAACTCACCTGCCGAATCAACTAGCCCCGAAAATGGATGGCGCTTAAGCGCGCGACCTATACCCGGCCGTCGGGGC	9 (0.000172%)	
CGAAATCCTATGATGTTATCCCATGCTAATGTATCCAGAGCGTAGGCTTGCTTTGAGCACTCTAATTTCTTCAAAGTAACAGCGCCGGAGGCACGACCCG	477 (0.009141%)	
CGAAGCACGCCCATCCAACCTAGGCGAGACAAGGGTTCACATTTCGTTCATCACCCTTGGCCGGCTATCGAACAGCCGGACTCCCATCAAAAGATGGTTG	23 (0.000441%)	
CGAAGCACGCCCATCCAACCTAGGCGAGACAAGGGTTCACATTTCGTTCATCACCCTTGGCCGGCTTTCGAACAGCCGGACTCCCATCAAAAGATGGTTG	22 (0.000422%)	
CGAATATGACTTGATGTCATGTGTATGATTGAGTATAAGA	518 (0.003971%)	
CGAATGCCAGCCGTTCGTTTGCATGTTCCTTGACACTTTTCGTGCCGGGGTTTTGTGATATCCGGAAGCAACGCGCACGACAAGACCGAGATAAAAGCTC	23 (0.000441%)	
CGAATGGCTCATTAAATCAGTTATAGTTTGTTTGATGGTAACTACTACTCGGATAACCGTAGTAATTCTAGAGCTAATACGTGCAACAAACCCCGACTTA	9 (0.000172%)	
CGACCAGAGGCTGTTCACCTTGGAGACCTGATGCGGTTATGAGTACGACCGGGCGTGAGCGGCACTCGGTCCTCCGGATTTTCAAGGGCCGCCGGGGGCG	17 (0.000326%)	
CGACCTTTTATCTAATAAATGCGTCCCTTCCATAAGTCGGGGTTTGTTGCACGTATTAGCTCTAGAATTACTACGGTTATCCGAGTAGTAGTTACCATCA	25 (0.000479%)	
CGACGGGCGGTGTGTACAAAGGGCAGGGACGTAGTCAACGCGAGCTGATGACTCGCGCTTACTAGGAATTCCTCGTTGAAGACCAACAATTGCAATGATC	27 (0.000517%)	
CGACGTGGGTGGTTCGCCGCCCGCGACGTCGCGAGAAGTCCACTAAACCTTATCATTTAGAGGAAGGAGAAGTCGTAACAAGGTTTCCGTAGGTGAACCT	97 (0.001859%)	
CGACTCCAAAACACTAACCAACCTTCTTCTTGCTTCTCAAAGCTTTCATGGTGTAGCCAAAGTCCATATGAGTCTTTGGCTTTGTGTCTTCTAACAAGGA	58 (0.001112%)	
CGACTTCCCTTGCCTACATTGTTCCATCGACCAGAGGCTGTTCACCTTGGAGACCTGATGCGGTTATGAGTACGACCGGGCGTGAGCGGCACTCGGTCCT	34 (0.000652%)	
CGACTTTGTGAAATGACTTGAGAGGTGTAGGATAAGTGGGAGCTTCGGCGCAAGTGAAATACCACTACTTTTAACGTTATTTTACTTACTCCGTGAATCG	15 (0.000287%)	
CGAGACAAGGGTTCACATTTCGTTCATCACCCTTGGCCGGCTATCGAACAGCCGGACTCCCATCAAAAGATGGTTGCCAAGAACATCTTCGTTACGGTTT	7 (0.000134%)	
CGAGACAAGGGTTCACATTTCGTTCATCACCCTTGGCCGGCTTTCGAACAGCCGGACTCCCATCAAAAGATGGTTGCCAAGAACATCTTCGTTACGGTTT	6 (0.000115%)	
CGAGGTGTGAGTGTCGCCCATGGGCATCGACACCTTGCGGCTAGGAACTGGAACGAGACGGGTAGCAAAGATTTCGAGTAGCACTTCATACTACCGTGGG	10 (0.000192%)	
CGAGGTGTGAGTGTCGCCCATGGGCATCGACACCTTGCGGCTAGGAACTGGAACGAGACGGGTGGCAAAGATTTCGAGTAGCACTTCATACTACCGTGGG	22 (0.000422%)	
CGAGTGTGAGCGAGGTGTGAGTGTCGCCCATGGGCATCGACACCTTGCGGCTAGGAACTGGAACGAGACGGGTAGCAAAGATTTCGAGTAGCACTTCATA	29 (0.000556%)	
CGAGTGTGAGCGAGGTGTGAGTGTCGCCCATGGGCATCGACACCTTGCGGCTAGGAACTGGAACGAGACGGGTGGCAAAGATTTCGAGTAGCACTTCATA	31 (0.000594%)	
CGAGTTATCATGAATCATCAGAGCAACGGGCAGAGCCCGCGTCGACCTTTTATCTAATAAATGCGTCCCTTCCATAAGTCGGGGTTTGTTGCACGTATTA	158 (0.003028%)	
CGATCCGCCTAGGCTGTCCCGAGTGTGAGCGAGGTGTGAGTGTCGCCCATGGGCATCGACACCTTGCGGCTAGGAACTGGAACGAGACGGGTAGCAAAGA	4 (0.000077%)	
CGATCCGCCTAGGCTGTCCCGAGTGTGAGCGAGGTGTGAGTGTCGCCCATGGGCATCGACACCTTGCGGCTAGGAACTGGAACGAGACGGGTGGCAAAGA	9 (0.000172%)	
CGATCCGGTTAAAATTCCGGAACCGGGACGTGGCGGTTGACGGCAACGTTAGGGAGTCCGGAGACGTCGGCGGGGGCCTCGGGAAGAGTTATCTTTTCTG	3 (0.000057%)	
CGATCCGTAACTTCGGGAAAAGGATTGGCTCTGAGGGCTGGGCTCGGGGGTCCCAGTTCCGAACCCGTCGGCTGTCAGCGGACTGCTCGAGCTGCTTCCG	3 (0.000057%)	
CGATCCGTCGAGTTATCATGAATCATCAGAGCAACGGGCAGAGCCCGCGTCGACCTTTTATCTAATAAATGCGTCCCTTCCATAAGTCGGGGTTTGTTGC	183 (0.003507%)	
CGATCCTCGTTAAGGGATTTAGATTGTACTCATTCCAATTACCAGACTCGAAAGAGCCCGGTATTGTTATTTATTGTCACTACCTCCCCGTGTCAGGATT	7 (0.000134%)	
CGATCGAAATCCTATGATGTTATCCCATGCTAATGTATCCAGAGCGTAGGCTTGCTTTGAGCACTCTAATTTCTTCAAAGTAACAGCGCCGGAGGCACGA	5 (0.000096%)	
CGATCGGCGGACCGGATTGCTCCGTTCCGCATCCGACCAGGACGCATCGCCGGCCCCCATCCGCTTCCCTCCCGACAATTTCAAGCACTCTTTGACTCTC	3 (0.000057%)	
CGATCGGGTTGCGGTTTAAGTTGTTATACTCAATCATACA	75 (0.000575%)	
CGCAACAAGATCTTAAAGGCGTAAGAATTGTATCCTTGTTAAAAGACACAAAGCCAAAGACTCATATGGACTTTGGCTACACCATGAAAGCTTTGAGAAG	52 (0.000997%)	
CGCAACCGGATCTTAAAGGCGTAAGAATTGTATCCTTGTTAGAAGACACAAAGCCAAAGACTCATATGGACTTTGGCTACACCATGAAAGCTTTGAGAAG	5 (0.000096%)	
CGCATGTCGGTACGCTCCAGGCGTCCTTGGCTCGGATTTAGGCCAACCGCGTGCGGTAACACACGGGAGACCAGCTTCCGTCCCGCATCAGCAAAGGATG	11 (0.000211%)	
CGCCCATCCAACCTAGGCGAGACAAGGGTTCACATTTCGTTCATCACCCTTGGCCGGCTATCGAACAGCCGGACTCCCATCAAAAGATGGTTGCCAAGAA	72 (0.001380%)	
CGCCCATCCAACCTAGGCGAGACAAGGGTTCACATTTCGTTCATCACCCTTGGCCGGCTTTCGAACAGCCGGACTCCCATCAAAAGATGGTTGCCAAGAA	82 (0.001571%)	

CGCCCATGGGCATCGACACCTTGCGGCTAGGAACTGGAACGAGACGGGTGGCAAAGATTTCGAGTAGCACTTCATACTACCGTGGGTTTTTTAAACCTTC	21 (0.000402%)	
CGCCGCCCGCGACGTCGCGAGAAGTCCACTAAACCTTATCATTTAGAGGAAGGAGAAGTCGTAACAAGGTTTCCGTAGGTGAACCTGCGGAAGGATCGAT	11 (0.000211%)	
CGCCGTTACTAAGGGAATCCTTGTTAGTTTCTTTTCCTCCGCTTATTGATATGCTTAAACTCAGCGGGTAATCCCGCCTGACCTGGGGTCGCTATATGGA	6 (0.000115%)	
CGCCTAACGGCGTGCCTCGGCATCAGCGTGCTCCGGGCGTCGGCCTGTGGGCTCCCCATTCGACCCGTCTTGAAACACGGACCAAGGAGTCTGACATGTG	37 (0.000709%)	
CGCCTAGGCTGTCCCGAGTGTGAGCGAGGTGTGAGTGTCGCCCATGGGCATCGACACCTTGCGGCTAGGAACTGGAACGAGACGGGTAGCAAAGATTTCG	111 (0.002127%)	
CGCCTAGGCTGTCCCGAGTGTGAGCGAGGTGTGAGTGTCGCCCATGGGCATCGACACCTTGCGGCTAGGAACTGGAACGAGACGGGTGGCAAAGATTTCG	170 (0.003258%)	
CGCCTCGAAGAACTAATGGCAGCCCACGCAAGGCAAGCCCATTCTCCTCGACGATTCAGCAGTTTTTGTCCGAGAACTGCTGAGAAAACTCGGAAAAAGG	6 (0.000115%)	
CGCGACCTATACCCGGCCGTCGGGGCAAGAGCCAGGCCTCGATGAGTAGGAGGGCGCGGCGGTCGCTGCAAAACCTAGGGCGCGAGCCCGGGCGGAGCGG	12 (0.000230%)	
CGCGCCTAACGGCGTGCCTCGGCATCAGCGTGCTCCGGGCGTCGGCCTGTGGGCTCCCCATTCGACCCGTCTTGAAACACGGACCAAGGAGTCTGACATG	46 (0.000882%)	
CGCGCGACCTATACCCGGCCGTCGGGGCAAGAGCCAGGCCTCGATGAGTAGGAGGGCGCGGCGGTCGCTGCAAAACCTAGGGCGCGAGCCCGGGCGGAGC	13 (0.000249%)	
CGCGGTAATTCCAGCTCCAATAGCGTATATTTAAGTTGTTGCAGTTAAAAAGCTCGTAGTTGAACCTTGGGATGGGTCGGCCGGTCCGCCTTTGGTGTGC	17 (0.000326%)	
CGCGTCGACCTTTTATCTAATAAATGCGTCCCTTCCATAAGTCGGGGTTTGTTGCACGTATTAGCTCTAGAATTACTACGGTTATCCGAGTAGTAGTTAC	8 (0.000153%)	
CGCTAAGGAGTGTGTAACAACTCACCTGCCGAATCAACTAGCCCCGAAAATGGATGGCGCTTAAGCGCGCGACCTATACCCGGCCGTCGGGGCAAGAGCC	15 (0.000287%)	
CGCTTATTGATATGCTTAAACTCAGCGGGTAATCCCGCCTGACCTGGGGTCGCTATATGGACTTTGGGTCATCTACAGCTTCCGGACAAGAGCGACCGAT	6 (0.000115%)	
CGCTTTCACGGTTCGTATTCGTACTGAAAATCAGAATCAAACGAGCTTTTACCCTTTTGTTCCACACGAGATTTCTGTTCTCGTTGAGCTCATCTTAGGA	8 (0.000153%)	
CGGACCGGATTGCTCCGTTCCGCATCCGACCAGGACGCATCGCCGGCCCCCATCCGCTTCCCTCCCGACAATTTCAAGCACTCTTTGACTCTCTTTTCAA	3 (0.000057%)	
CGGAGAATTAGGGTTCGATTCCGGAGAGGGAGCCTGAGAAACGGCTACCACATCCAAGGAAGGCAGCAGGCGCGCAAATTACCCAATCCTGACACGGGGA	19 (0.000364%)	
CGGATCTTAAAGGCGTAAGAATTGTATCCTTGTTAGAAGACACAAAGCCAAAGACTCATATGGACTTTGGCTACACCATGAAAGCTTTGAGAAGCAAGAA	7 (0.000134%)	
CGGATTGCTCCGTTCCGCATCCGACCAGGACGCATCGCCGGCCCCCATCCGCTTCCCTCCCGACAATTTCAAGCACTCTTTGACTCTCTTTTCAAAGTCC	24 (0.000460%)	
CGGCAATTCCCCGCCACATCCTCTCAAACGCAATGGAAAGAGAGAAAGGACGAGGTCTTGACCGTCATCTTTTGCCCGAAGGACGGATGAGCTTTGGCGG	188 (0.003603%)	
CGGCACATCTGTTAAAAGATAACGCAGGTGTCCTAAGATGAGCTCAACGAGAACAGAAATCTCGTGTGGAACAAAAGGGTAAAAGCTCGTTTGATTCTGA	12 (0.000230%)	
CGGCATCAGCGTGCTCCGGGCGTCGGCCTGTGGGCTCCCCATTCGACCCGTCTTGAAACACGGACCAAGGAGTCTGACATGTGTGCGAGTCAACGGGTGA	15 (0.000287%)	
CGGCCGTCGGGGCAAGAGCCAGGCCTCGATGAGTAGGAGGGCGCGGCGGTCGCTGCAAAACCTAGGGCGCGAGCCCGGGCGGAGCGGCCGTCGGTGCAGA	46 (0.000882%)	
CGGCGGACCGGATTGCTCCGTTCCGCATCCGACCAGGACGCATCGCCGGCCCCCATCCGCTTCCCTCCCGACAATTTCAAGCACTCTTTGACTCTCTTTT	15 (0.000287%)	
CGGCGTGCCTCGGCATCAGCGTGCTCCGGGCGTCGGCCTGTGGGCTCCCCATTCGACCCGTCTTGAAACACGGACCAAGGAGTCTGACATGTGTGCGAGT	9 (0.000172%)	
CGGGCAGAGCCCGCGTCGACCTTTTATCTAATAAATGCGTCCCTTCCATAAGTCGGGGTTTGTTGCACGTATTAGCTCTAGAATTACTACGGTTATCCGA	47 (0.000901%)	
CGGGCGGTGTGTACAAAGGGCAGGGACGTAGTCAACGCGAGCTGATGACTCGCGCTTACTAGGAATTCCTCGTTGAAGACCAACAATTGCAATGATCGAT	43 (0.000824%)	
CGGGCGTCGGCCTGTGGGCTCCCCATTCGACCCGTCTTGAAACACGGACCAAGGAGTCTGACATGTGTGCGAGTCAACGGGTGAGTAAACCCGTAAGGCG	46 (0.000882%)	
CGGGGAGGCGAATGCCAGCCGTTCGTTTGCATGTTCCTTGACACTTTTCGTGCCGGGGTTTTGTGATATCCGGAAGCAACGCGCACGACAAGACCGAGAT	14 (0.000268%)	
CGGGGGCATTCGTATTTCATAGTCAGAGGTGAAATTCTTGGATTTATGAAAGACGAACAACTGCGAAAGCATTTGCCAAGGATGTTTTCATTAATCAAGA	22 (0.000422%)	
CGGGTGACGGAGAATTAGGGTTCGATTCCGGAGAGGGAGCCTGAGAAACGGCTACCACATCCAAGGAAGGCAGCAGGCGCGCAAATTACCCAATCCTGAC	3 (0.000057%)	
CGGGTTGCGGTTTAAGTTGTTATACTCAATCATACACATG	497 (0.003810%)	
CGGGTTTACTCACCCGTTGACTCGCACACATGTCAGACTCCTTGGTCCGTGTTTCAAGACGGGTCGAATGGGGAGCCCACAGGCCGACGCCCGGAGCACG	18 (0.000345%)	
CGGTAATTCCAGCTCCAATAGCGTATATTTAAGTTGTTGCAGTTAAAAAGCTCGTAGTTGAACCTTGGGATGGGTCGGCCGGTCCGCCTTTGGTGTGCAT	19 (0.000364%)	
CGGTACGCTCCAGGCGTCCTTGGCTCGGATTTAGGCCAACCGCGTGCGGTAACACACGGGAGACCAGCTTCCGTCCCGCATCAGCAAAGGATGGTGAGGG	4 (0.000077%)	
CGGTAGGAGCGACGGGCGGTGTGTACAAAGGGCAGGGACGTAGTCAACGCGAGCTGATGACTCGCGCTTACTAGGAATTCCTCGTTGAAGACCAACAATT	54 (0.001035%)	
CGGTTTAAGTTCTTATACTCAATCATACACATGACATCAA	555 (0.004255%)	
CGGTTTAAGTTGTTATACTCAATCATACACATGACAACAAGTCATATTCGACTCCAAAACACTAACCAACCTTCTTCTTGCTTCTCAAAGCTTTCATGGT	896 (0.017171%)	
CGTAACTTCGGGAAAAGGATTGGCTCTGAGGGCTGGGCTCGGGGGTCCCAGTTCCGAACCCGTCGGCTGTCAGCGGACTGCTCGAGCTGCTTCCGCGGCG	3 (0.000057%)	
CGTAAGAATTGTATCCTTGTTAGAAGACACAAAGCCAAAGACTCATATGGACTTTGGCTACACCATGAAAGCTTTGAGAAGCAAGAAGAAGGTTGGTTAG	58 (0.001112%)	
CGTAGGCTTGCTTTGAGCACTCTAATTTCTTCAAAGTAACAGCGCCGGAGGCACGACCCGGCCAATTAAGACCAGGAGCGTATCGCCGACCGAAGGGACA	17 (0.000326%)	
CGTATTCGTACTGAAAATCAGAATCAAACGAGCTTTTACCCTTTTGTTCCACACGAGATTTCTGTTCTCGTTGAGCTCATCTTAGGACACCTGCGTTATC	27 (0.000517%)	
CGTATTTCATAGTCAGAGGTGAAATTCTTGGATTTATGAAAGACGAACAACTGCGAAAGCATTTGCCAAGGATGTTTTCATTAATCAAGAACGAAAGTTG	7 (0.000134%)	

CGTCCCTCACCATCCTTTGCTGATGCGGGACGGAAGCTGGTCTCCCGTGTGTTACCGCACGCGGTTGGCCTAAATCCGAGCCAAGGACGCCTGGAGCGTA	11 (0.000211%)
CGTCGAGTTATCATGAATCATCAGAGCAACGGGCAGAGCCCGCGTCGACCTTTTATCTAATAAATGCGTCCCTTCCATAAGTCGGGGTTTGTTGCACGTA	104 (0.001993%)
CGTCGGCCTGTGGGCTCCCCATTCGACCCGTCTTGAAACACGGACCAAGGAGTCTGACATGTGTGCGAGTCAACGGGTGAGTAAACCCGTAAGGCGCAAG	6 (0.000115%)
CGTCTCCGGGGAGGCGAATGCCAGCCGTTCGTTTGCATGTTCCTTGACACTTTTCGTGCCGGGGTTTTGTGATATCCGGAAGCAACGCGCACGACAAGAC	3 (0.000057%)
CGTGCCTCGGCATCAGCGTGCTCCGGGCGTCGGCCTGTGGGCTCCCCATTCGACCCGTCTTGAAACACGGACCAAGGAGTCTGACATGTGTGCGAGTCAA	21 (0.000402%)
CGTGCTCCGGGCGTCGGCCTGTGGGCTCCCCATTCGACCCGTCTTGAAACACGGACCAAGGAGTCTGACATGTGTGCGAGTCAACGGGTGAGTAAACCCG	9 (0.000172%)
CGTGGGTGGTTCGCCGCCCGCGACGTCGCGAGAAGTCCACTAAACCTTATCATTTAGAGGAAGGAGAAGTCGTAACAAGGTTTCCGTAGGTGAACCTGCG	27 (0.000517%)
CGTTAAGGGATTTAGATTGTACTCATTCCAATTACCAGACTCGAAAGAGCCCGGTATTGTTATTTATTGTCACTACCTCCCCGTGTCAGGATTGGGTAAT	61 (0.001169%)
CTAAATCCTATTTTCTGGTAAATTTTCATAATTTTTTGACACCTCTAGCTAGGTCATTTGACCTGATACAACATCGGATTTTCATGGTCTAGTTGGGGCT	39 (0.000747%)
CTAACGCCTCGAAGAACTAATGGCAGCCCACGCAAGGCAAGCCCATTCTCCTCGACGATTCAGCAGTTTTTGTCCGAGAACTGCTGAGAAAACTCGGAAA	28 (0.000537%)
CTAACGGCGTGCCTCGGCATCAGCGTGCTCCGGGCGTCGGCCTGTGGGCTCCCCATTCGACCCGTCTTGAAACACGGACCAAGGAGTCTGACATGTGTGC	94 (0.001801%)
CTAAGTAGTGTTTCCTTGTTAGAAGACACAAAGCCAAAGACTCATATGGACTTTGGCTACACCATGAAAGCTTTGAGAAGCAAGAAGAAGGTTGGTTAGT	77 (0.001476%)
CTAATGTATCCAGAGCGTAGGCTTGCTTTGAGCACTCTAATTTCTTCAAAGTAACAGCGCCGGAGGCACGACCCGGCCAATTAAGACCAGGAGCGTATCG	37 (0.000709%)
CTACATTGTTCCATCGACCAGAGGCTGTTCACCTTGGAGACCTGATGCGGTTATGAGTACGACCGGGCGTGAGCGGCACTCGGTCCTCCGGATTTTCAAG	48 (0.000920%)
CTAGGCGAGACAAGGGTTCACATTTCGTTCATCACCCTTGGCCGGCTATCGAACAGCCGGACTCCCATCAAAAGATGGTTGCCAAGAACATCTTCGTTAC	25 (0.000479%)
CTAGGCGAGACAAGGGTTCACATTTCGTTCATCACCCTTGGCCGGCTTTCGAACAGCCGGACTCCCATCAAAAGATGGTTGCCAAGAACATCTTCGTTAC	25 (0.000479%)
CTAGGCTGTCCCGAGTGTGAGCGAGGTGTGAGTGTCGCCCATGGGCATCGACACCTTGCGGCTAGGAACTGGAACGAGACGGGTAGCAAAGATTTCGAGT	274 (0.005251%)
CTAGGCTGTCCCGAGTGTGAGCGAGGTGTGAGTGTCGCCCATGGGCATCGACACCTTGCGGCTAGGAACTGGAACGAGACGGGTGGCAAAGATTTCGAGT	414 (0.007934%)
CTAGTCCGACTTTGTGAAATGACTTGAGAGGTGTAGGATAAGTGGGAGCTTCGGCGCAAGTGAAATACCACTACTTTTAACGTTATTTTACTTACTCCGT	17 (0.000326%)
CTATACCCGGCCGTCGGGGCAAGAGCCAGGCCTCGATGAGTAGGAGGGCGCGGCGGTCGCTGCAAAACCTAGGGCGCGAGCCCGGGCGGAGCGGCCGTCG	64 (0.001227%)
CTATGATGTTATCCCATGCTAATGTATCCAGAGCGTAGGCTTGCTTTGAGCACTCTAATTTCTTCAAAGTAACAGCGCCGGAGGCACGACCCGGCCAATT	220 (0.004216%)
CTATTTTCTGGTAAATTTTCATAATTTTTTGACACCTCTAGCTAGGTCATTTGACCTGATACAACATCGGATTTTCATGGTCTAGTTGGGGCTCCGTGGG	13 (0.000249%)
CTCAAACGCAATGGAAAGAGAGAAAGGACGAGGTCTTGACCGTCATCTTTTGCCCGAAGGACGGATGAGCTTTGGCGGGACTGAATCACTTCGAGTCACC	4 (0.000077%)
CTCAAAGATTAAGCCATGCATGTGTAAGTATGAACGAATTCAGACTGTGAAACTGCGAATGGCTCATTAAATCAGTTATAGTTTGTTTGATGGTAACTAC	24 (0.000460%)
CTCAAAGCTTTCATGGTGTAGCCAAAGTCCATATGAGTCTTTGGCTTTGTGTCTTCTAACAAGGAAACACTACTTAGGCTTATAAGATGCGGTTGCGGTT	9 (0.000172%)
CTCAATCATACACATGACAACAAGTCATATTCGACTCCAAAACACTAACCAACCTTCTTCTTGCTTCTCAAAGCTTTCATGGTGTAGCCAAAGTCCATAT	13 (0.000249%)
CTCAATCATACACATGACATCAAGTCATATTCGACTCCAAAACACTAACCAACCTTCTTCTTGCTTCTCAAAGCTTTCATGGTGTAGCCAAAGTCCATAT	100 (0.001916%)
CTCACCATCCTTTGCTGATGCGGGACGGAAGCTGGTCTCCCGTGTGTTACCGCACGCGGTTGGCCTAAATCCGAGCCAAGGACGCCTGGAGCGTACCGAC	9 (0.000172%)
CTCACCCGTTGACTCGCACACATGTCAGACTCCTTGGTCCGTGTTTCAAGACGGGTCGAATGGGGAGCCCACAGGCCGACGCCCGGAGCACGCTGATGCC	15 (0.000287%)
CTCATATGGACTTTGGCTACACCATGAAAGCTTTGAGAAGCAAGAAGAAGGTTGGTTAGTGTTTTGGAGTCGAATATGACTTGATGTCATGTGTATGATT	83 (0.001591%)
CTCATTAAATCAGTTATAGTTTGTTTGATGGTAACTACTACTCGGATAACCGTAGTAATTCTAGAGCTAATACGTGCAACAAACCCCGACTTATGGAAGG	6 (0.000115%)
CTCATTCCAATTACCAGACTCGAAAGAGCCCGGTATTGTTATTTATTGTCACTACCTCCCCGTGTCAGGATTGGGTAATTTGCGCGCCTGCTGCCTTCCT	18 (0.000345%)
CTCCGCTTATTGATATGCTTAAACTCAGCGGGTAATCCCGCCTGACCTGGGGTCGCTATATGGACTTTGGGTCATCTACAGCTTCCGGACAAGAGCGACC	5 (0.000096%)
CTCCGGGCGTCGGCCTGTGGGCTCCCCATTCGACCCGTCTTGAAACACGGACCAAGGAGTCTGACATGTGTGCGAGTCAACGGGTGAGTAAACCCGTAAG	15 (0.000287%)
CTCCGGGGAGGCGAATGCCAGCCGTTCGTTTGCATGTTCCTTGACACTTTTCGTGCCGGGGTTTTGTGATATCCGGAAGCAACGCGCACGACAAGACCGA	38 (0.000728%)
CTCGAAAGAGCCCGGTATTGTTATTTATTGTCACTACCTCCCCGTGTCAGGATTGGGTAATTTGCGCGCCTGCTGCCTTCCTTGGATGTGGTAGCCGTTT	41 (0.000786%)
CTCGAAGAACTAATGGCAGCCCACGCAAGGCAAGCCCATTCTCCTCGACGATTCAGCAGTTTTTGTCCGAGAACTGCTGAGAAAACTCGGAAAAAGGCAA	17 (0.000326%)
CTCGCCGTTACTAAGGGAATCCTTGTTAGTTTCTTTTCCTCCGCTTATTGATATGCTTAAACTCAGCGGGTAATCCCGCCTGACCTGGGGTCGCTATATG	34 (0.000652%)
CTCGGCATCAGCGTGCTCCGGGCGTCGGCCTGTGGGCTCCCCATTCGACCCGTCTTGAAACACGGACCAAGGAGTCTGACATGTGTGCGAGTCAACGGGT	22 (0.000422%)
CTCGTTAAGGGATTTAGATTGTACTCATTCCAATTACCAGACTCGAAAGAGCCCGGTATTGTTATTTATTGTCACTACCTCCCCGTGTCAGGATTGGGTA	151 (0.002894%)
CTCTAGTCCGACTTTGTGAAATGACTTGAGAGGTGTAGGATAAGTGGGAGCTTCGGCGCAAGTGAAATACCACTACTTTTAACGTTATTTTACTTACTCC	30 (0.000575%)
CTCTCTCTCTCTCTCTCTCTCTCTCTCTCTCTCTCTCT	47 (0.000360%)
CTGAAAATCAGAATCAAACGAGCTTTTACCCTTTTGTTCCACACGAGATTTCTGTTCTCGTTGAGCTCATCTTAGGACACCTGCGTTATCTTTTAACAGA	21 (0.000402%)

CTGATGCGGGACGGAAGCTGGTCTCCCGTGTGTTACCGCACGCGGTTGGCCTAAATCCGAGCCAAGGACGCCTGGAGCGTACCGACATGCGGTGGTGAAC	22 (0.000422%)
CTGCAGCACGCGCCTAACGGCGTGCCTCGGCATCAGCGTGCTCCGGGCGTCGGCCTGTGGGCTCCCCATTCGACCCGTCTTGAAACACGGACCAAGGAGT	2112 (0.040475%)
CTGCCAGTAGTCATATGCTTGTCTCAAAGATTAAGCCATGCATGTGTAAGTATGAACGAATTCAGACTGTGAAACTGCGAATGGCTCATTAAATCAGTTA	2028 (0.038866%)
CTGCGAATGGCTCATTAAATCAGTTATAGTTTGTTTGATGGTAACTACTACTCGGATAACCGTAGTAATTCTAGAGCTAATACGTGCAACAAACCCCGAC	30 (0.000575%)
CTGGGCCGCACGCGCGCTACACTGATGTATTCAACGAGTTCACACCTTGGCCGACAGGCCCGGGTAATCTTTGAAATTTCATCGTGATGGGGATAGATCG	75 (0.001437%)
CTGGGGCGGCACATCTGTTAAAAGATAACGCAGGTGTCCTAAGATGAGCTCAACGAGAACAGAAATCTCGTGTGGAACAAAAGGGTAAAAGCTCGTTTGA	13 (0.000249%)
CTGGTGCCAGCAGCCGCGGTAATTCCAGCTCCAATAGCGTATATTTAAGTTGTTGCAGTTAAAAAGCTCGTAGTTGAACCTTGGGATGGGTCGGCCGGTC	49 (0.000939%)
CTGTCCCGAGTGTGAGCGAGGTGTGAGTGTCGCCCATGGGCATCGACACCTTGCGGCTAGGAACTGGAACGAGACGGGTAGCAAAGATTTCGAGTAGCAC	10 (0.000192%)
CTGTCCCGAGTGTGAGCGAGGTGTGAGTGTCGCCCATGGGCATCGACACCTTGCGGCTAGGAACTGGAACGAGACGGGTGGCAAAGATTTCGAGTAGCAC	48 (0.000920%)
CTGTGATGCCCTTAGATGTTCTGGGCCGCACGCGCGCTACACTGATGTATTCAACGAGTTCACACCTTGGCCGACAGGCCCGGGTAATCTTTGAAATTTC	114 (0.002185%)
CTGTTAAAAGATAACGCAGGTGTCCTAAGATGAGCTCAACGAGAACAGAAATCTCGTGTGGAACAAAAGGGTAAAAGCTCGTTTGATTCTGATTTTCAGT	5 (0.000096%)
CTGTTGAGCTTGACTCTAGTCCGACTTTGTGAAATGACTTGAGAGGTGTAGGATAAGTGGGAGCTTCGGCGCAAGTGAAATACCACTACTTTTAACGTTA	12 (0.000230%)
CTTAAAAGCCTAAGTAGTGTTTCCTTGTTAGAAGACACAAAGCCAAAGACTCATATGGACTTTGGCTACACCATGAAAGCTTTGAGAAGCAAGAAGAAGG	196 (0.003756%)
CTTAAAGGCGTAAGAATTGTATCCTTGTTAAAAGACACAAAGCCAAAGACTCATATGGACTTTGGCTACACCATGAAAGCTTTGAGAAGCAAGAAGAAGG	219 (0.004197%)
CTTAAAGGCGTAAGAATTGTATCCTTGTTAGAAGACACAAAGCCAAAGACTCATATGGACTTTGGCTACACCATGAAAGCTTTGAGAAGCAAGAAGAAGG	225 (0.004312%)
CTTAAGCGCGCGACCTATACCCGGCCGTCGGGGCAAGAGCCAGGCCTCGATGAGTAGGAGGGCGCGGCGGTCGCTGCAAAACCTAGGGCGCGAGCCCGGG	65 (0.001246%)
CTTAGATGTTCTGGGCCGCACGCGCGCTACACTGATGTATTCAACGAGTTCACACCTTGGCCGACAGGCCCGGGTAATCTTTGAAATTTCATCGTGATGG	132 (0.002530%)
CTTATACTCAATCATACACATGACATCAAGTCATATTCGACTCCAAAACACTAACCAACCTTCTTCTTGCTTCTCAAAGCTTTCATGGTGTAGCCAAAGT	127 (0.002434%)
CTTATTGATATGCTTAAACTCAGCGGGTAATCCCGCCTGACCTGGGGTCGCTATATGGACTTTGGGTCATCTACAGCTTCCGGACAAGAGCGACCGATAA	37 (0.000709%)
CTTCAACGAGGAATTCCTAGTAAGCGCGAGTCATCAGCTCGCGTTGACTACGTCCCTGCCCTTTGTACACACCGCCCGTCGCTCCTACCGATTGAATGAT	63 (0.001207%)
CTTCCCTTGCCTACATTGTTCCATCGACCAGAGGCTGTTCACCTTGGAGACCTGATGCGGTTATGAGTACGACCGGGCGTGAGCGGCACTCGGTCCTCCG	57 (0.001092%)
CTTCTCAAAGCTTTCATGGTGTAGCCAAAGTCCATATGAGTCTTTGGCTTTGTGTCTTCTAACAAGGAAACACTACTTAGGCTTATAAGATGCGGTTGCG	9 (0.000172%)
CTTGACTCTAGTCCGACTTTGTGAAATGACTTGAGAGGTGTAGGATAAGTGGGAGCTTCGGCGCAAGTGAAATACCACTACTTTTAACGTTATTTTACTT	47 (0.000901%)
CTTGATGTCATGTGTATGATTGAGTATAAGAACTTAAACC	258 (0.001978%)
CTTGCCTACATTGTTCCATCGACCAGAGGCTGTTCACCTTGGAGACCTGATGCGGTTATGAGTACGACCGGGCGTGAGCGGCACTCGGTCCTCCGGATTT	55 (0.001054%)
CTTGCTTCTCAAAGCTTTCATGGTGTAGCCAAAGTCCATATGAGTCTTTGGCTTTGTGTCTTCTAACAAGGAAACACTACTTAGGCTTATAAGATGCGGT	45 (0.000862%)
CTTGCTTTGAGCACTCTAATTTCTTCAAAGTAACAGCGCCGGAGGCACGACCCGGCCAATTAAGACCAGGAGCGTATCGCCGACCGAAGGGACAAGCCGA	59 (0.001131%)
CTTGTCTCAAAGATTAAGCCATGCATGTGTAAGTATGAACGAATTCAGACTGTGAAACTGCGAATGGCTCATTAAATCAGTTATAGTTTGTTTGATGGTA	52 (0.000997%)
CTTGTTAGAAGACACAAAGCCAAAGACTCATATGGACTTTGGCTACACCATGAAAGCTTTGAGAAGCAAGAAGAAGGTTGGTTAGTGTTTTGGAGTCGAA	116 (0.002223%)
CTTGTTAGTTTCTTTTCCTCCGCTTATTGATATGCTTAAACTCAGCGGGTAATCCCGCCTGACCTGGGGTCGCTATATGGACTTTGGGTCATCTACAGCT	24 (0.000460%)
CTTTCACGGTTCGTATTCGTACTGAAAATCAGAATCAAACGAGCTTTTACCCTTTTGTTCCACACGAGATTTCTGTTCTCGTTGAGCTCATCTTAGGACA	40 (0.000767%)
CTTTCCAACGAAGCACGCCCATCCAACCTAGGCGAGACAAGGGTTCACATTTCGTTCATCACCCTTGGCCGGCTATCGAACAGCCGGACTCCCATCAAAA	15 (0.000287%)
CTTTGCTGATGCGGGACGGAAGCTGGTCTCCCGTGTGTTACCGCACGCGGTTGGCCTAAATCCGAGCCAAGGACGCCTGGAGCGTACCGACATGCGGTGG	69 (0.001322%)
CTTTTATCTAATAAATGCGTCCCTTCCATAAGTCGGGGTTTGTTGCACGTATTAGCTCTAGAATTACTACGGTTATCCGAGTAGTAGTTACCATCAAACA	76 (0.001457%)
CTTTTCCTCCGCTTATTGATATGCTTAAACTCAGCGGGTAATCCCGCCTGACCTGGGGTCGCTATATGGACTTTGGGTCATCTACAGCTTCCGGACAAGA	48 (0.000920%)
GAAAATCAGAATCAAACGAGCTTTTACCCTTTTGTTCCACACGAGATTTCTGTTCTCGTTGAGCTCATCTTAGGACACCTGCGTTATCTTTTAACAGATG	13 (0.000249%)
GAAACTGCGAATGGCTCATTAAATCAGTTATAGTTTGTTTGATGGTAACTACTACTCGGATAACCGTAGTAATTCTAGAGCTAATACGTGCAACAAACCC	176 (0.003373%)
GAAAGAAGACCCTGTTGAGCTTGACTCTAGTCCGACTTTGTGAAATGACTTGAGAGGTGTAGGATAAGTGGGAGCTTCGGCGCAAGTGAAATACCACTAC	33 (0.000632%)
GAAAGAGCCCGGTATTGTTATTTATTGTCACTACCTCCCCGTGTCAGGATTGGGTAATTTGCGCGCCTGCTGCCTTCCTTGGATGTGGTAGCCGTTTCTC	29 (0.000556%)
GAAATCCGCTAAGGAGTGTGTAACAACTCACCTGCCGAATCAACTAGCCCCGAAAATGGATGGCGCTTAAGCGCGCGACCTATACCCGGCCGTCGGGGCA	25 (0.000479%)
GAAATCCTATGATGTTATCCCATGCTAATGTATCCAGAGCGTAGGCTTGCTTTGAGCACTCTAATTTCTTCAAAGTAACAGCGCCGGAGGCACGACCCGG	216 (0.004140%)
GAAGACACAAAGCCAAAGACTCATATGGACTTTGGCTACACCATGAAAGCTTTGAGAAGCAAGAAGAAGGTTGGTTAGTGTTTTGGAGTCGAATATGACT	36 (0.000690%)
GAAGACCCTGTTGAGCTTGACTCTAGTCCGACTTTGTGAAATGACTTGAGAGGTGTAGGATAAGTGGGAGCTTCGGCGCAAGTGAAATACCACTACTTTT	14 (0.000268%)

GAAGCACGCCCATCCAACCTAGGCGAGACAAGGGTTCACATTTCGTTCATCACCCTTGGCCGGCTATCGAACAGCCGGACTCCCATCAAAAGATGGTTGC	12 (0.000230%)	
GAAGCACGCCCATCCAACCTAGGCGAGACAAGGGTTCACATTTCGTTCATCACCCTTGGCCGGCTTTCGAACAGCCGGACTCCCATCAAAAGATGGTTGC	12 (0.000230%)	
GAAGTCGAAATCCGCTAAGGAGTGTGTAACAACTCACCTGCCGAATCAACTAGCCCCGAAAATGGATGGCGCTTAAGCGCGCGACCTATACCCGGCCGTC	3 (0.000057%)	
GAATATGACTTGATGTCATGTGTATGATTGAGTATAAGAA	432 (0.003312%)	
GAATCAAACGAGCTTTTACCCTTTTGTTCCACACGAGATTTCTGTTCTCGTTGAGCTCATCTTAGGACACCTGCGTTATCTTTTAACAGATGTGCCGCCC	3 (0.000057%)	
GAATCATCAGAGCAACGGGCAGAGCCCGCGTCGACCTTTTATCTAATAAATGCGTCCCTTCCATAAGTCGGGGTTTGTTGCACGTATTAGCTCTAGAATT	106 (0.002031%)	
GAATCCTTGTTAGTTTCTTTTCCTCCGCTTATTGATATGCTTAAACTCAGCGGGTAATCCCGCCTGACCTGGGGTCGCTATATGGACTTTGGGTCATCTA	12 (0.000230%)	
GAATGCCAGCCGTTCGTTTGCATGTTCCTTGACACTTTTCGTGCCGGGGTTTTGTGATATCCGGAAGCAACGCGCACGACAAGACCGAGATAAAAGCTCC	13 (0.000249%)	
GAATGGCTCATTAAATCAGTTATAGTTTGTTTGATGGTAACTACTACTCGGATAACCGTAGTAATTCTAGAGCTAATACGTGCAACAAACCCCGACTTAT	6 (0.000115%)	
GAATTAGGGTTCGATTCCGGAGAGGGAGCCTGAGAAACGGCTACCACATCCAAGGAAGGCAGCAGGCGCGCAAATTACCCAATCCTGACACGGGGAGGTA	7 (0.000134%)	
GAATTGTATCCTTGTTAGAAGACACAAAGCCAAAGACTCATATGGACTTTGGCTACACCATGAAAGCTTTGAGAAGCAAGAAGAAGGTTGGTTAGTGTTT	17 (0.000326%)	
GACAAGGGTTCACATTTCGTTCATCACCCTTGGCCGGCTATCGAACAGCCGGACTCCCATCAAAAGATGGTTGCCAAGAACATCTTCGTTACGGTTTGCT	12 (0.000230%)	
GACAAGGGTTCACATTTCGTTCATCACCCTTGGCCGGCTTTCGAACAGCCGGACTCCCATCAAAAGATGGTTGCCAAGAACATCTTCGTTACGGTTTGCT	7 (0.000134%)	
GACACAAAGCCAAAGACTCATATGGACTTTGGCTACACCATGAAAGCTTTGAGAAGCAAGAAGAAGGTTGGTTAGTGTTTTGGAGTCGAATATGACTTGA	59 (0.001131%)	
GACAGTCGGGGGCATTCGTATTTCATAGTCAGAGGTGAAATTCTTGGATTTATGAAAGACGAACAACTGCGAAAGCATTTGCCAAGGATGTTTTCATTAA	4 (0.000077%)	
GACATCAAGTCATATTCGACTCCAAAACACTAACCAACCTTCTTCTTGCTTCTCAAAGCTTTCATGGTGTAGCCAAAGTCCATATGAGTCTTTGGCTTTG	53 (0.001016%)	
GACATTGTCAGGTGGGGAGTTTGGCTGGGGCGGCACATCTGTTAAAAGATAACGCAGGTGTCCTAAGATGAGCTCAACGAGAACAGAAATCTCGTGTGGA	38 (0.000728%)	
GACCAGAGGCTGTTCACCTTGGAGACCTGATGCGGTTATGAGTACGACCGGGCGTGAGCGGCACTCGGTCCTCCGGATTTTCAAGGGCCGCCGGGGGCGC	5 (0.000096%)	
GACCGGATTGCTCCGTTCCGCATCCGACCAGGACGCATCGCCGGCCCCCATCCGCTTCCCTCCCGACAATTTCAAGCACTCTTTGACTCTCTTTTCAAAG	12 (0.000230%)	
GACCTATACCCGGCCGTCGGGGCAAGAGCCAGGCCTCGATGAGTAGGAGGGCGCGGCGGTCGCTGCAAAACCTAGGGCGCGAGCCCGGGCGGAGCGGCCG	11 (0.000211%)	
GACCTTTTATCTAATAAATGCGTCCCTTCCATAAGTCGGGGTTTGTTGCACGTATTAGCTCTAGAATTACTACGGTTATCCGAGTAGTAGTTACCATCAA	8 (0.000153%)	
GACGGAGAATTAGGGTTCGATTCCGGAGAGGGAGCCTGAGAAACGGCTACCACATCCAAGGAAGGCAGCAGGCGCGCAAATTACCCAATCCTGACACGGG	4 (0.000077%)	
GACGGGCGGTGTGTACAAAGGGCAGGGACGTAGTCAACGCGAGCTGATGACTCGCGCTTACTAGGAATTCCTCGTTGAAGACCAACAATTGCAATGATCG	13 (0.000249%)	
GACGTGGGTGGTTCGCCGCCCGCGACGTCGCGAGAAGTCCACTAAACCTTATCATTTAGAGGAAGGAGAAGTCGTAACAAGGTTTCCGTAGGTGAACCTG	53 (0.001016%)	
GACTCATATGGACTTTGGCTACACCATGAAAGCTTTGAGAAGCAAGAAGAAGGTTGGTTAGTGTTTTGGAGTCGAATATGACTTGATGTCATGTGTATGA	7 (0.000134%)	
GACTCCAAAACACTAACCAACCTTCTTCTTGCTTCTCAAAGCTTTCATGGTGTAGCCAAAGTCCATATGAGTCTTTGGCTTTGTGTCTTCTAACAAGGAA	245 (0.004695%)	
GACTTCCCTTGCCTACATTGTTCCATCGACCAGAGGCTGTTCACCTTGGAGACCTGATGCGGTTATGAGTACGACCGGGCGTGAGCGGCACTCGGTCCTC	14 (0.000268%)	
GACTTGATGTCATGTGTATGATTGAGTATAAGAACTTAAA	130 (0.000997%)	
GACTTTGGCTACACCATGAAAGCTTTGAGAAGCAAGAAGAAGGTTGGTTAGTGTTTTGGAGTCGAATATGACTTGATGTCATGTGTATGATTGAGTATAA	47 (0.000901%)	
GAGAATTAGGGTTCGATTCCGGAGAGGGAGCCTGAGAAACGGCTACCACATCCAAGGAAGGCAGCAGGCGCGCAAATTACCCAATCCTGACACGGGGAGG	4 (0.000077%)	
GAGACAAGGGTTCACATTTCGTTCATCACCCTTGGCCGGCTATCGAACAGCCGGACTCCCATCAAAAGATGGTTGCCAAGAACATCTTCGTTACGGTTTG	12 (0.000230%)	
GAGACAAGGGTTCACATTTCGTTCATCACCCTTGGCCGGCTTTCGAACAGCCGGACTCCCATCAAAAGATGGTTGCCAAGAACATCTTCGTTACGGTTTG	3 (0.000057%)	
GAGCAACGGGCAGAGCCCGCGTCGACCTTTTATCTAATAAATGCGTCCCTTCCATAAGTCGGGGTTTGTTGCACGTATTAGCTCTAGAATTACTACGGTT	18 (0.000345%)	
GAGCCCGCGTCGACCTTTTATCTAATAAATGCGTCCCTTCCATAAGTCGGGGTTTGTTGCACGTATTAGCTCTAGAATTACTACGGTTATCCGAGTAGTA	7 (0.000134%)	
GAGCCCGGTATTGTTATTTATTGTCACTACCTCCCCGTGTCAGGATTGGGTAATTTGCGCGCCTGCTGCCTTCCTTGGATGTGGTAGCCGTTTCTCAGGC	3 (0.000057%)	
GAGCGACGGGCGGTGTGTACAAAGGGCAGGGACGTAGTCAACGCGAGCTGATGACTCGCGCTTACTAGGAATTCCTCGTTGAAGACCAACAATTGCAATG	11 (0.000211%)	
GAGCGAGGTGTGAGTGTCGCCCATGGGCATCGACACCTTGCGGCTAGGAACTGGAACGAGACGGGTAGCAAAGATTTCGAGTAGCACTTCATACTACCGT	6 (0.000115%)	
GAGCGAGGTGTGAGTGTCGCCCATGGGCATCGACACCTTGCGGCTAGGAACTGGAACGAGACGGGTGGCAAAGATTTCGAGTAGCACTTCATACTACCGT	7 (0.000134%)	
GAGGACATTGTCAGGTGGGGAGTTTGGCTGGGGCGGCACATCTGTTAAAAGATAACGCAGGTGTCCTAAGATGAGCTCAACGAGAACAGAAATCTCGTGT	76 (0.001457%)	
GAGGCGAATGCCAGCCGTTCGTTTGCATGTTCCTTGACACTTTTCGTGCCGGGGTTTTGTGATATCCGGAAGCAACGCGCACGACAAGACCGAGATAAAA	8 (0.000153%)	
GAGGGCAAGTCTGGTGCCAGCAGCCGCGGTAATTCCAGCTCCAATAGCGTATATTTAAGTTGTTGCAGTTAAAAAGCTCGTAGTTGAACCTTGGGATGGG	14 (0.000268%)	
GAGGTGTGAGTGTCGCCCATGGGCATCGACACCTTGCGGCTAGGAACTGGAACGAGACGGGTAGCAAAGATTTCGAGTAGCACTTCATACTACCGTGGGT	8 (0.000153%)	
GAGGTGTGAGTGTCGCCCATGGGCATCGACACCTTGCGGCTAGGAACTGGAACGAGACGGGTGGCAAAGATTTCGAGTAGCACTTCATACTACCGTGGGT	7 (0.000134%)	

GAGTGTCGCCCATGGGCATCGACACCTTGCGGCTAGGAACTGGAACGAGACGGGTAGCAAAGATTTCGAGTAGCACTTCATACTACCGTGGGTTTTTTAA	4 (0.000077%)
GAGTGTCGCCCATGGGCATCGACACCTTGCGGCTAGGAACTGGAACGAGACGGGTGGCAAAGATTTCGAGTAGCACTTCATACTACCGTGGGTTTTTTAA	4 (0.000077%)
GAGTGTGAGCGAGGTGTGAGTGTCGCCCATGGGCATCGACACCTTGCGGCTAGGAACTGGAACGAGACGGGTGGCAAAGATTTCGAGTAGCACTTCATAC	4 (0.000077%)
GAGTTATCATGAATCATCAGAGCAACGGGCAGAGCCCGCGTCGACCTTTTATCTAATAAATGCGTCCCTTCCATAAGTCGGGGTTTGTTGCACGTATTAG	78 (0.001495%)
GAGTTTGGCTGGGGCGGCACATCTGTTAAAAGATAACGCAGGTGTCCTAAGATGAGCTCAACGAGAACAGAAATCTCGTGTGGAACAAAAGGGTAAAAGC	6 (0.000115%)
GATAGGCCACGCTTTCACGGTTCGTATTCGTACTGAAAATCAGAATCAAACGAGCTTTTACCCTTTTGTTCCACACGAGATTTCTGTTCTCGTTGAGCTC	1713 (0.032829%)
GATAGTGGCCTACCATGGTGGTAACGGGTGACGGAGAATTAGGGTTCGATTCCGGAGAGGGAGCCTGAGAAACGGCTACCACATCCAAGGAAGGCAGCAG	3 (0.000057%)
GATCAAGTTCACCACCGCATGTCGGTACGCTCCAGGCGTCCTTGGCTCGGATTTAGGCCAACCGCGTGCGGTAACACACGGGAGACCAGCTTCCGTCCCG	47 (0.000901%)
GATCACGGCAATTCCCCGCCACATCCTCTCAAACGCAATGGAAAGAGAGAAAGGACGAGGTCTTGACCGTCATCTTTTGCCCGAAGGACGGATGAGCTTT	31 (0.000594%)
GATCATTCAATCGGTAGGAGCGACGGGCGGTGTGTACAAAGGGCAGGGACGTAGTCAACGCGAGCTGATGACTCGCGCTTACTAGGAATTCCTCGTTGAA	762 (0.014603%)
GATCCATGCTTTCCAACGAAGCACGCCCATCCAACCTAGGCGAGACAAGGGTTCACATTTCGTTCATCACCCTTGGCCGGCTATCGAACAGCCGGACTCC	78 (0.001495%)
GATCCATTGGAGGGCAAGTCTGGTGCCAGCAGCCGCGGTAATTCCAGCTCCAATAGCGTATATTTAAGTTGTTGCAGTTAAAAAGCTCGTAGTTGAACCT	1486 (0.028478%)
GATCCGCCTAGGCTGTCCCGAGTGTGAGCGAGGTGTGAGTGTCGCCCATGGGCATCGACACCTTGCGGCTAGGAACTGGAACGAGACGGGTAGCAAAGAT	96 (0.001840%)
GATCCGCCTAGGCTGTCCCGAGTGTGAGCGAGGTGTGAGTGTCGCCCATGGGCATCGACACCTTGCGGCTAGGAACTGGAACGAGACGGGTGGCAAAGAT	142 (0.002721%)
GATCCGGTTAAAATTCCGGAACCGGGACGTGGCGGTTGACGGCAACGTTAGGGAGTCCGGAGACGTCGGCGGGGGCCTCGGGAAGAGTTATCTTTTCTGT	145 (0.002779%)
GATCCGTAACTTCGGGAAAAGGATTGGCTCTGAGGGCTGGGCTCGGGGGTCCCAGTTCCGAACCCGTCGGCTGTCAGCGGACTGCTCGAGCTGCTTCCGC	21 (0.000402%)
GATCCGTCGAGTTATCATGAATCATCAGAGCAACGGGCAGAGCCCGCGTCGACCTTTTATCTAATAAATGCGTCCCTTCCATAAGTCGGGGTTTGTTGCA	123 (0.002357%)
GATCCTCGTTAAGGGATTTAGATTGTACTCATTCCAATTACCAGACTCGAAAGAGCCCGGTATTGTTATTTATTGTCACTACCTCCCCGTGTCAGGATTG	88 (0.001686%)
GATCGAAATCCTATGATGTTATCCCATGCTAATGTATCCAGAGCGTAGGCTTGCTTTGAGCACTCTAATTTCTTCAAAGTAACAGCGCCGGAGGCACGAC	354 (0.006784%)
GATCGATCAA	145668 (0.279165%)
GATCGATCAAGTTCACCACCGCATGTCGGTACGCTCCAGGCGTCCTTGGCTCGGATTTAGGCCAACCGCGTGCGGTAACACACGGGAGACCAGCTTCCGT	1310 (0.025105%)
GATCGATCACGGCAATTCCCCGCCACATCCTCTCAAACGCAATGGAAAGAGAGAAAGGACGAGGTCTTGACCGTCATCTTTTGCCCGAAGGACGGATGAG	933 (0.017880%)
GATCGATCCATGCTTTCCAACGAAGCACGCCCATCCAACCTAGGCGAGACAAGGGTTCACATTTCGTTCATCACCCTTGGCCGGCTATCGAACAGCCGGA	1366 (0.026179%)
GATCGATCCGCCTAGGCTGTCCCGAGTGTGAGCGAGGTGTGAGTGTCGCCCATGGGCATCGACACCTTGCGGCTAGGAACTGGAACGAGACGGGTAGCAA	1052 (0.020161%)
GATCGATCCGCCTAGGCTGTCCCGAGTGTGAGCGAGGTGTGAGTGTCGCCCATGGGCATCGACACCTTGCGGCTAGGAACTGGAACGAGACGGGTGGCAA	1547 (0.029647%)
GATCGATCCGGTTAAAATTCCGGAACCGGGACGTGGCGGTTGACGGCAACGTTAGGGAGTCCGGAGACGTCGGCGGGGGCCTCGGGAAGAGTTATCTTTT	1624 (0.031123%)
GATCGATCCGTAACTTCGGGAAAAGGATTGGCTCTGAGGGCTGGGCTCGGGGGTCCCAGTTCCGAACCCGTCGGCTGTCAGCGGACTGCTCGAGCTGCTT	1344 (0.025757%)
GATCGATCCGTCGAGTTATCATGAATCATCAGAGCAACGGGCAGAGCCCGCGTCGACCTTTTATCTAATAAATGCGTCCCTTCCATAAGTCGGGGTTTGT	1141 (0.021867%)
GATCGATCCTCGTTAAGGGATTTAGATTGTACTCATTCCAATTACCAGACTCGAAAGAGCCCGGTATTGTTATTTATTGTCACTACCTCCCCGTGTCAGG	1505 (0.028843%)
GATCGATCGAAATCCTATGATGTTATCCCATGCTAATGTATCCAGAGCGTAGGCTTGCTTTGAGCACTCTAATTTCTTCAAAGTAACAGCGCCGGAGGCA	1296 (0.024837%)
GATCGATCGGCGGACCGGATTGCTCCGTTCCGCATCCGACCAGGACGCATCGCCGGCCCCCATCCGCTTCCCTCCCGACAATTTCAAGCACTCTTTGACT	907 (0.017382%)
GATCGATCGGGTTGCGGTTTAAGTTGTTATACTCAATCAT	4328 (0.033178%)
GATCGATCTT	165258 (0.316708%)
GATCGGCGGACCGGATTGCTCCGTTCCGCATCCGACCAGGACGCATCGCCGGCCCCCATCCGCTTCCCTCCCGACAATTTCAAGCACTCTTTGACTCTCT	9 (0.000172%)
GATCGGGTTGCGGTTTAAGTTGTTATACTCAATCATACAC	1247 (0.009559%)
GATCTTAAAGGCGTAAGAATTGTATCCTTGTTAAAAGACACAAAGCCAAAGACTCATATGGACTTTGGCTACACCATGAAAGCTTTGAGAAGCAAGAAGA	696 (0.013338%)
GATCTTAAAGGCGTAAGAATTGTATCCTTGTTAGAAGACACAAAGCCAAAGACTCATATGGACTTTGGCTACACCATGAAAGCTTTGAGAAGCAAGAAGA	521 (0.009985%)
GATGCCCTTAGATGTTCTGGGCCGCACGCGCGCTACACTGATGTATTCAACGAGTTCACACCTTGGCCGACAGGCCCGGGTAATCTTTGAAATTTCATCG	9 (0.000172%)
GATGTCATGTGTATGATTGAGTATAAGAACTTAAACCGCA	128 (0.000981%)
GATGTTATCCCATGCTAATGTATCCAGAGCGTAGGCTTGCTTTGAGCACTCTAATTTCTTCAAAGTAACAGCGCCGGAGGCACGACCCGGCCAATTAAGA	12 (0.000230%)
GATGTTCTGGGCCGCACGCGCGCTACACTGATGTATTCAACGAGTTCACACCTTGGCCGACAGGCCCGGGTAATCTTTGAAATTTCATCGTGATGGGGAT	12 (0.000230%)
GATTAACAGGGACAGTCGGGGGCATTCGTATTTCATAGTCAGAGGTGAAATTCTTGGATTTATGAAAGACGAACAACTGCGAAAGCATTTGCCAAGGATG	183 (0.003507%)
GATTAAGCCATGCATGTGTAAGTATGAACGAATTCAGACTGTGAAACTGCGAATGGCTCATTAAATCAGTTATAGTTTGTTTGATGGTAACTACTACTCG	22 (0.000422%)

GATTGTACTCATTCCAATTACCAGACTCGAAAGAGCCCGGTATTGTTATTTATTGTCACTACCTCCCCGTGTCAGGATTGGGTAATTTGCGCGCCTGCTG	7 (0.000134%)	
GATTTAGATTGTACTCATTCCAATTACCAGACTCGAAAGAGCCCGGTATTGTTATTTATTGTCACTACCTCCCCGTGTCAGGATTGGGTAATTTGCGCGC	19 (0.000364%)	
GCAACCGGATCTTAAAGGCGTAAGAATTGTATCCTTGTTAGAAGACACAAAGCCAAAGACTCATATGGACTTTGGCTACACCATGAAAGCTTTGAGAAGC	5 (0.000096%)	
GCAACGGGCAGAGCCCGCGTCGACCTTTTATCTAATAAATGCGTCCCTTCCATAAGTCGGGGTTTGTTGCACGTATTAGCTCTAGAATTACTACGGTTAT	45 (0.000862%)	
GCAACGTCGCTATGAACGCTTGGCTGCCACAAGCCAGTTATCCCTGTGGTAACTTTTCTGACACCTCTAGCTTCAAATTCCGAAGGTCTAAAGGATCGAT	73 (0.001399%)	
GCAAGTCTGGTGCCAGCAGCCGCGGTAATTCCAGCTCCAATAGCGTATATTTAAGTTGTTGCAGTTAAAAAGCTCGTAGTTGAACCTTGGGATGGGTCGG	18 (0.000345%)	
GCAATAACAGGTCTGTGATGCCCTTAGATGTTCTGGGCCGCACGCGCGCTACACTGATGTATTCAACGAGTTCACACCTTGGCCGACAGGCCCGGGTAAT	1018 (0.019509%)	
GCAATTCCCCGCCACATCCTCTCAAACGCAATGGAAAGAGAGAAAGGACGAGGTCTTGACCGTCATCTTTTGCCCGAAGGACGGATGAGCTTTGGCGGGA	30 (0.000575%)	
GCACATCTGTTAAAAGATAACGCAGGTGTCCTAAGATGAGCTCAACGAGAACAGAAATCTCGTGTGGAACAAAAGGGTAAAAGCTCGTTTGATTCTGATT	6 (0.000115%)	
GCACGCCCATCCAACCTAGGCGAGACAAGGGTTCACATTTCGTTCATCACCCTTGGCCGGCTATCGAACAGCCGGACTCCCATCAAAAGATGGTTGCCAA	39 (0.000747%)	
GCACGCCCATCCAACCTAGGCGAGACAAGGGTTCACATTTCGTTCATCACCCTTGGCCGGCTTTCGAACAGCCGGACTCCCATCAAAAGATGGTTGCCAA	27 (0.000517%)	
GCACGCGCCTAACGGCGTGCCTCGGCATCAGCGTGCTCCGGGCGTCGGCCTGTGGGCTCCCCATTCGACCCGTCTTGAAACACGGACCAAGGAGTCTGAC	19 (0.000364%)	
GCAGAGCCCGCGTCGACCTTTTATCTAATAAATGCGTCCCTTCCATAAGTCGGGGTTTGTTGCACGTATTAGCTCTAGAATTACTACGGTTATCCGAGTA	26 (0.000498%)	
GCAGCACGCGCCTAACGGCGTGCCTCGGCATCAGCGTGCTCCGGGCGTCGGCCTGTGGGCTCCCCATTCGACCCGTCTTGAAACACGGACCAAGGAGTCT	425 (0.008145%)	
GCAGCCGCGGTAATTCCAGCTCCAATAGCGTATATTTAAGTTGTTGCAGTTAAAAAGCTCGTAGTTGAACCTTGGGATGGGTCGGCCGGTCCGCCTTTGG	15 (0.000287%)	
GCATCAGCGTGCTCCGGGCGTCGGCCTGTGGGCTCCCCATTCGACCCGTCTTGAAACACGGACCAAGGAGTCTGACATGTGTGCGAGTCAACGGGTGAGT	10 (0.000192%)	
GCATCATAAGGATACTAAATCCTATTTTCTGGTAAATTTTCATAATTTTTTGACACCTCTAGCTAGGTCATTTGACCTGATACAACATCGGATTTTCATG	9 (0.000172%)	
GCATGTGTAAGTATGAACGAATTCAGACTGTGAAACTGCGAATGGCTCATTAAATCAGTTATAGTTTGTTTGATGGTAACTACTACTCGGATAACCGTAG	7 (0.000134%)	
GCCAAAGACTCATATGGACTTTGGCTACACCATGAAAGCTTTGAGAAGCAAGAAGAAGGTTGGTTAGTGTTTTGGAGTCGAATATGACTTGATGTCATGT	57 (0.001092%)	
GCCACATCCTCTCAAACGCAATGGAAAGAGAGAAAGGACGAGGTCTTGACCGTCATCTTTTGCCCGAAGGACGGATGAGCTTTGGCGGGACTGAATCACT	25 (0.000479%)	
GCCACCCTAACGCCTCGAAGAACTAATGGCAGCCCACGCAAGGCAAGCCCATTCTCCTCGACGATTCAGCAGTTTTTGTCCGAGAACTGCTGAGAAAACT	881 (0.016884%)	
GCCACGCTTTCACGGTTCGTATTCGTACTGAAAATCAGAATCAAACGAGCTTTTACCCTTTTGTTCCACACGAGATTTCTGTTCTCGTTGAGCTCATCTT	127 (0.002434%)	
GCCAGCAGCCGCGGTAATTCCAGCTCCAATAGCGTATATTTAAGTTGTTGCAGTTAAAAAGCTCGTAGTTGAACCTTGGGATGGGTCGGCCGGTCCGCCT	27 (0.000517%)	
GCCAGCCGTTCGTTTGCATGTTCCTTGACACTTTTCGTGCCGGGGTTTTGTGATATCCGGAAGCAACGCGCACGACAAGACCGAGATAAAAGCTCCCGAT	28 (0.000537%)	
GCCAGTAGTCATATGCTTGTCTCAAAGATTAAGCCATGCATGTGTAAGTATGAACGAATTCAGACTGTGAAACTGCGAATGGCTCATTAAATCAGTTATA	263 (0.005040%)	
GCCATGCATGTGTAAGTATGAACGAATTCAGACTGTGAAACTGCGAATGGCTCATTAAATCAGTTATAGTTTGTTTGATGGTAACTACTACTCGGATAAC	14 (0.000268%)	
GCCCATCCAACCTAGGCGAGACAAGGGTTCACATTTCGTTCATCACCCTTGGCCGGCTATCGAACAGCCGGACTCCCATCAAAAGATGGTTGCCAAGAAC	53 (0.001016%)	
GCCCATCCAACCTAGGCGAGACAAGGGTTCACATTTCGTTCATCACCCTTGGCCGGCTTTCGAACAGCCGGACTCCCATCAAAAGATGGTTGCCAAGAAC	36 (0.000690%)	
GCCCATGGGCATCGACACCTTGCGGCTAGGAACTGGAACGAGACGGGTGGCAAAGATTTCGAGTAGCACTTCATACTACCGTGGGTTTTTTAAACCTTCC	16 (0.000307%)	
GCCCGCGTCGACCTTTTATCTAATAAATGCGTCCCTTCCATAAGTCGGGGTTTGTTGCACGTATTAGCTCTAGAATTACTACGGTTATCCGAGTAGTAGT	5 (0.000096%)	
GCCCTTAGATGTTCTGGGCCGCACGCGCGCTACACTGATGTATTCAACGAGTTCACACCTTGGCCGACAGGCCCGGGTAATCTTTGAAATTTCATCGTGA	16 (0.000307%)	
GCCGACTTCCCTTGCCTACATTGTTCCATCGACCAGAGGCTGTTCACCTTGGAGACCTGATGCGGTTATGAGTACGACCGGGCGTGAGCGGCACTCGGTC	218 (0.004178%)	
GCCGCCCGCGACGTCGCGAGAAGTCCACTAAACCTTATCATTTAGAGGAAGGAGAAGTCGTAACAAGGTTTCCGTAGGTGAACCTGCGGAAGGATCGATC	14 (0.000268%)	
GCCGCGGTAATTCCAGCTCCAATAGCGTATATTTAAGTTGTTGCAGTTAAAAAGCTCGTAGTTGAACCTTGGGATGGGTCGGCCGGTCCGCCTTTGGTGT	7 (0.000134%)	
GCCGTCGGGGCAAGAGCCAGGCCTCGATGAGTAGGAGGGCGCGGCGGTCGCTGCAAAACCTAGGGCGCGAGCCCGGGCGGAGCGGCCGTCGGTGCAGATC	7 (0.000134%)	
GCCGTTACTAAGGGAATCCTTGTTAGTTTCTTTTCCTCCGCTTATTGATATGCTTAAACTCAGCGGGTAATCCCGCCTGACCTGGGGTCGCTATATGGAC	16 (0.000307%)	
GCCTAACGGCGTGCCTCGGCATCAGCGTGCTCCGGGCGTCGGCCTGTGGGCTCCCCATTCGACCCGTCTTGAAACACGGACCAAGGAGTCTGACATGTGT	48 (0.000920%)	
GCCTAAGTAGTGTTTCCTTGTTAGAAGACACAAAGCCAAAGACTCATATGGACTTTGGCTACACCATGAAAGCTTTGAGAAGCAAGAAGAAGGTTGGTTA	38 (0.000728%)	
GCCTAGGCTGTCCCGAGTGTGAGCGAGGTGTGAGTGTCGCCCATGGGCATCGACACCTTGCGGCTAGGAACTGGAACGAGACGGGTAGCAAAGATTTCGA	257 (0.004925%)	
GCCTAGGCTGTCCCGAGTGTGAGCGAGGTGTGAGTGTCGCCCATGGGCATCGACACCTTGCGGCTAGGAACTGGAACGAGACGGGTGGCAAAGATTTCGA	378 (0.007244%)	
GCCTCGAAGAACTAATGGCAGCCCACGCAAGGCAAGCCCATTCTCCTCGACGATTCAGCAGTTTTTGTCCGAGAACTGCTGAGAAAACTCGGAAAAAGGC	14 (0.000268%)	
GCCTCGGCATCAGCGTGCTCCGGGCGTCGGCCTGTGGGCTCCCCATTCGACCCGTCTTGAAACACGGACCAAGGAGTCTGACATGTGTGCGAGTCAACGG	10 (0.000192%)	
GCGAATGCCAGCCGTTCGTTTGCATGTTCCTTGACACTTTTCGTGCCGGGGTTTTGTGATATCCGGAAGCAACGCGCACGACAAGACCGAGATAAAAGCT	30 (0.000575%)	

GCGAATGGCTCATTAAATCAGTTATAGTTTGTTTGATGGTAACTACTACTCGGATAACCGTAGTAATTCTAGAGCTAATACGTGCAACAAACCCCGACTT	20 (0.000383%)
GCGACCTATACCCGGCCGTCGGGGCAAGAGCCAGGCCTCGATGAGTAGGAGGGCGCGGCGGTCGCTGCAAAACCTAGGGCGCGAGCCCGGGCGGAGCGGC	84 (0.001610%)
GCGACGGGCGGTGTGTACAAAGGGCAGGGACGTAGTCAACGCGAGCTGATGACTCGCGCTTACTAGGAATTCCTCGTTGAAGACCAACAATTGCAATGAT	44 (0.000843%)
GCGACGTGGGTGGTTCGCCGCCCGCGACGTCGCGAGAAGTCCACTAAACCTTATCATTTAGAGGAAGGAGAAGTCGTAACAAGGTTTCCGTAGGTGAACC	2275 (0.043599%)
GCGAGACAAGGGTTCACATTTCGTTCATCACCCTTGGCCGGCTATCGAACAGCCGGACTCCCATCAAAAGATGGTTGCCAAGAACATCTTCGTTACGGTT	41 (0.000786%)
GCGAGACAAGGGTTCACATTTCGTTCATCACCCTTGGCCGGCTTTCGAACAGCCGGACTCCCATCAAAAGATGGTTGCCAAGAACATCTTCGTTACGGTT	32 (0.000613%)
GCGAGGTGTGAGTGTCGCCCATGGGCATCGACACCTTGCGGCTAGGAACTGGAACGAGACGGGTAGCAAAGATTTCGAGTAGCACTTCATACTACCGTGG	31 (0.000594%)
GCGAGGTGTGAGTGTCGCCCATGGGCATCGACACCTTGCGGCTAGGAACTGGAACGAGACGGGTGGCAAAGATTTCGAGTAGCACTTCATACTACCGTGG	53 (0.001016%)
GCGCCTAACGGCGTGCCTCGGCATCAGCGTGCTCCGGGCGTCGGCCTGTGGGCTCCCCATTCGACCCGTCTTGAAACACGGACCAAGGAGTCTGACATGT	25 (0.000479%)
GCGCGACCTATACCCGGCCGTCGGGGCAAGAGCCAGGCCTCGATGAGTAGGAGGGCGCGGCGGTCGCTGCAAAACCTAGGGCGCGAGCCCGGGCGGAGCG	34 (0.000652%)
GCGCGCGACCTATACCCGGCCGTCGGGGCAAGAGCCAGGCCTCGATGAGTAGGAGGGCGCGGCGGTCGCTGCAAAACCTAGGGCGCGAGCCCGGGCGGAG	5 (0.000096%)
GCGGACCGGATTGCTCCGTTCCGCATCCGACCAGGACGCATCGCCGGCCCCCATCCGCTTCCCTCCCGACAATTTCAAGCACTCTTTGACTCTCTTTTCA	19 (0.000364%)
GCGGAGGACATTGTCAGGTGGGGAGTTTGGCTGGGGCGGCACATCTGTTAAAAGATAACGCAGGTGTCCTAAGATGAGCTCAACGAGAACAGAAATCTCG	1582 (0.030318%)
GCGGCACATCTGTTAAAAGATAACGCAGGTGTCCTAAGATGAGCTCAACGAGAACAGAAATCTCGTGTGGAACAAAAGGGTAAAAGCTCGTTTGATTCTG	9 (0.000172%)
GCGGTAATTCCAGCTCCAATAGCGTATATTTAAGTTGTTGCAGTTAAAAAGCTCGTAGTTGAACCTTGGGATGGGTCGGCCGGTCCGCCTTTGGTGTGCA	22 (0.000422%)
GCGGTTTAAGTTCTTATACTCAATCATACACATGACATCA	651 (0.004990%)
GCGTAAGAATTGTATCCTTGTTAGAAGACACAAAGCCAAAGACTCATATGGACTTTGGCTACACCATGAAAGCTTTGAGAAGCAAGAAGAAGGTTGGTTA	19 (0.000364%)
GCGTAGGCTTGCTTTGAGCACTCTAATTTCTTCAAAGTAACAGCGCCGGAGGCACGACCCGGCCAATTAAGACCAGGAGCGTATCGCCGACCGAAGGGAC	52 (0.000997%)
GCGTCGACCTTTTATCTAATAAATGCGTCCCTTCCATAAGTCGGGGTTTGTTGCACGTATTAGCTCTAGAATTACTACGGTTATCCGAGTAGTAGTTACC	12 (0.000230%)
GCGTCGGCCTGTGGGCTCCCCATTCGACCCGTCTTGAAACACGGACCAAGGAGTCTGACATGTGTGCGAGTCAACGGGTGAGTAAACCCGTAAGGCGCAA	8 (0.000153%)
GCGTGCCTCGGCATCAGCGTGCTCCGGGCGTCGGCCTGTGGGCTCCCCATTCGACCCGTCTTGAAACACGGACCAAGGAGTCTGACATGTGTGCGAGTCA	9 (0.000172%)
GCGTGCTCCGGGCGTCGGCCTGTGGGCTCCCCATTCGACCCGTCTTGAAACACGGACCAAGGAGTCTGACATGTGTGCGAGTCAACGGGTGAGTAAACCC	12 (0.000230%)
GCTAAGGAGTGTGTAACAACTCACCTGCCGAATCAACTAGCCCCGAAAATGGATGGCGCTTAAGCGCGCGACCTATACCCGGCCGTCGGGGCAAGAGCCA	56 (0.001073%)
GCTAATGTATCCAGAGCGTAGGCTTGCTTTGAGCACTCTAATTTCTTCAAAGTAACAGCGCCGGAGGCACGACCCGGCCAATTAAGACCAGGAGCGTATC	22 (0.000422%)
GCTCATTAAATCAGTTATAGTTTGTTTGATGGTAACTACTACTCGGATAACCGTAGTAATTCTAGAGCTAATACGTGCAACAAACCCCGACTTATGGAAG	12 (0.000230%)
GCTCCGGGCGTCGGCCTGTGGGCTCCCCATTCGACCCGTCTTGAAACACGGACCAAGGAGTCTGACATGTGTGCGAGTCAACGGGTGAGTAAACCCGTAA	3 (0.000057%)
GCTCCGTTCCGCATCCGACCAGGACGCATCGCCGGCCCCCATCCGCTTCCCTCCCGACAATTTCAAGCACTCTTTGACTCTCTTTTCAAAGTCCTTTTCA	5 (0.000096%)
GCTCGCCGTTACTAAGGGAATCCTTGTTAGTTTCTTTTCCTCCGCTTATTGATATGCTTAAACTCAGCGGGTAATCCCGCCTGACCTGGGGTCGCTATAT	49 (0.000939%)
GCTGATGCGGGACGGAAGCTGGTCTCCCGTGTGTTACCGCACGCGGTTGGCCTAAATCCGAGCCAAGGACGCCTGGAGCGTACCGACATGCGGTGGTGAA	10 (0.000192%)
GCTGGGGCGGCACATCTGTTAAAAGATAACGCAGGTGTCCTAAGATGAGCTCAACGAGAACAGAAATCTCGTGTGGAACAAAAGGGTAAAAGCTCGTTTG	11 (0.000211%)
GCTGTCCCGAGTGTGAGCGAGGTGTGAGTGTCGCCCATGGGCATCGACACCTTGCGGCTAGGAACTGGAACGAGACGGGTAGCAAAGATTTCGAGTAGCA	69 (0.001322%)
GCTGTCCCGAGTGTGAGCGAGGTGTGAGTGTCGCCCATGGGCATCGACACCTTGCGGCTAGGAACTGGAACGAGACGGGTGGCAAAGATTTCGAGTAGCA	104 (0.001993%)
GCTTAAGCGCGCGACCTATACCCGGCCGTCGGGGCAAGAGCCAGGCCTCGATGAGTAGGAGGGCGCGGCGGTCGCTGCAAAACCTAGGGCGCGAGCCCGG	1050 (0.020123%)
GCTTATTGATATGCTTAAACTCAGCGGGTAATCCCGCCTGACCTGGGGTCGCTATATGGACTTTGGGTCATCTACAGCTTCCGGACAAGAGCGACCGATA	31 (0.000594%)
GCTTCTCAAAGCTTTCATGGTGTAGCCAAAGTCCATATGAGTCTTTGGCTTTGTGTCTTCTAACAAGGAAACACTACTTAGGCTTATAAGATGCGGTTGC	12 (0.000230%)
GCTTGACTCTAGTCCGACTTTGTGAAATGACTTGAGAGGTGTAGGATAAGTGGGAGCTTCGGCGCAAGTGAAATACCACTACTTTTAACGTTATTTTACT	22 (0.000422%)
GCTTGCTTTGAGCACTCTAATTTCTTCAAAGTAACAGCGCCGGAGGCACGACCCGGCCAATTAAGACCAGGAGCGTATCGCCGACCGAAGGGACAAGCCG	10 (0.000192%)
GCTTGTCTCAAAGATTAAGCCATGCATGTGTAAGTATGAACGAATTCAGACTGTGAAACTGCGAATGGCTCATTAAATCAGTTATAGTTTGTTTGATGGT	18 (0.000345%)
GCTTTCACGGTTCGTATTCGTACTGAAAATCAGAATCAAACGAGCTTTTACCCTTTTGTTCCACACGAGATTTCTGTTCTCGTTGAGCTCATCTTAGGAC	19 (0.000364%)
GCTTTCCAACGAAGCACGCCCATCCAACCTAGGCGAGACAAGGGTTCACATTTCGTTCATCACCCTTGGCCGGCTATCGAACAGCCGGACTCCCATCAAA	65 (0.001246%)
GGAAAGAAGACCCTGTTGAGCTTGACTCTAGTCCGACTTTGTGAAATGACTTGAGAGGTGTAGGATAAGTGGGAGCTTCGGCGCAAGTGAAATACCACTA	747 (0.014316%)
GGAAAGAGAGAAAGGACGAGGTCTTGACCGTCATCTTTTGCCCGAAGGACGGATGAGCTTTGGCGGGACTGAATCACTTCGAGTCACCGTCGACAACTTT	11 (0.000211%)
GGAAGTCGAAATCCGCTAAGGAGTGTGTAACAACTCACCTGCCGAATCAACTAGCCCCGAAAATGGATGGCGCTTAAGCGCGCGACCTATACCCGGCCGT	25 (0.000479%)

GGAATCCTTGTTAGTTTCTTTTCCTCCGCTTATTGATATGCTTAAACTCAGCGGGTAATCCCGCCTGACCTGGGGTCGCTATATGGACTTTGGGTCATCT	30 (0.000575%)
GGACAGTCGGGGGCATTCGTATTTCATAGTCAGAGGTGAAATTCTTGGATTTATGAAAGACGAACAACTGCGAAAGCATTTGCCAAGGATGTTTTCATTA	75 (0.001437%)
GGACATTGTCAGGTGGGGAGTTTGGCTGGGGCGGCACATCTGTTAAAAGATAACGCAGGTGTCCTAAGATGAGCTCAACGAGAACAGAAATCTCGTGTGG	176 (0.003373%)
GGACCGGATTGCTCCGTTCCGCATCCGACCAGGACGCATCGCCGGCCCCCATCCGCTTCCCTCCCGACAATTTCAAGCACTCTTTGACTCTCTTTTCAAA	23 (0.000441%)
GGACTTTGGCTACACCATGAAAGCTTTGAGAAGCAAGAAGAAGGTTGGTTAGTGTTTTGGAGTCGAATATGACTTGATGTCATGTGTATGATTGAGTATA	80 (0.001533%)
GGAGAATTAGGGTTCGATTCCGGAGAGGGAGCCTGAGAAACGGCTACCACATCCAAGGAAGGCAGCAGGCGCGCAAATTACCCAATCCTGACACGGGGAG	88 (0.001686%)
GGAGCGACGGGCGGTGTGTACAAAGGGCAGGGACGTAGTCAACGCGAGCTGATGACTCGCGCTTACTAGGAATTCCTCGTTGAAGACCAACAATTGCAAT	54 (0.001035%)
GGAGGACATTGTCAGGTGGGGAGTTTGGCTGGGGCGGCACATCTGTTAAAAGATAACGCAGGTGTCCTAAGATGAGCTCAACGAGAACAGAAATCTCGTG	450 (0.008624%)
GGAGGCGAATGCCAGCCGTTCGTTTGCATGTTCCTTGACACTTTTCGTGCCGGGGTTTTGTGATATCCGGAAGCAACGCGCACGACAAGACCGAGATAAA	15 (0.000287%)
GGAGGGCAAGTCTGGTGCCAGCAGCCGCGGTAATTCCAGCTCCAATAGCGTATATTTAAGTTGTTGCAGTTAAAAAGCTCGTAGTTGAACCTTGGGATGG	58 (0.001112%)
GGAGTTTGGCTGGGGCGGCACATCTGTTAAAAGATAACGCAGGTGTCCTAAGATGAGCTCAACGAGAACAGAAATCTCGTGTGGAACAAAAGGGTAAAAG	6 (0.000115%)
GGATACTAAATCCTATTTTCTGGTAAATTTTCATAATTTTTTGACACCTCTAGCTAGGTCATTTGACCTGATACAACATCGGATTTTCATGGTCTAGTTG	14 (0.000268%)
GGATAGTGGCCTACCATGGTGGTAACGGGTGACGGAGAATTAGGGTTCGATTCCGGAGAGGGAGCCTGAGAAACGGCTACCACATCCAAGGAAGGCAGCA	728 (0.013952%)
GGATCTTAAAGGCGTAAGAATTGTATCCTTGTTAGAAGACACAAAGCCAAAGACTCATATGGACTTTGGCTACACCATGAAAGCTTTGAGAAGCAAGAAG	17 (0.000326%)
GGATTGCTCCGTTCCGCATCCGACCAGGACGCATCGCCGGCCCCCATCCGCTTCCCTCCCGACAATTTCAAGCACTCTTTGACTCTCTTTTCAAAGTCCT	10 (0.000192%)
GGATTTAGATTGTACTCATTCCAATTACCAGACTCGAAAGAGCCCGGTATTGTTATTTATTGTCACTACCTCCCCGTGTCAGGATTGGGTAATTTGCGCG	5 (0.000096%)
GGCAAGTCTGGTGCCAGCAGCCGCGGTAATTCCAGCTCCAATAGCGTATATTTAAGTTGTTGCAGTTAAAAAGCTCGTAGTTGAACCTTGGGATGGGTCG	66 (0.001265%)
GGCAATTCCCCGCCACATCCTCTCAAACGCAATGGAAAGAGAGAAAGGACGAGGTCTTGACCGTCATCTTTTGCCCGAAGGACGGATGAGCTTTGGCGGG	83 (0.001591%)
GGCACATCTGTTAAAAGATAACGCAGGTGTCCTAAGATGAGCTCAACGAGAACAGAAATCTCGTGTGGAACAAAAGGGTAAAAGCTCGTTTGATTCTGAT	25 (0.000479%)
GGCAGAGCCCGCGTCGACCTTTTATCTAATAAATGCGTCCCTTCCATAAGTCGGGGTTTGTTGCACGTATTAGCTCTAGAATTACTACGGTTATCCGAGT	21 (0.000402%)
GGCATCAGCGTGCTCCGGGCGTCGGCCTGTGGGCTCCCCATTCGACCCGTCTTGAAACACGGACCAAGGAGTCTGACATGTGTGCGAGTCAACGGGTGAG	11 (0.000211%)
GGCATCGACACCTTGCGGCTAGGAACTGGAACGAGACGGGTGGCAAAGATTTCGAGTAGCACTTCATACTACCGTGGGTTTTTTAAACCTTCCGAGTTTT	9 (0.000172%)
GGCATGCATCATAAGGATACTAAATCCTATTTTCTGGTAAATTTTCATAATTTTTTGACACCTCTAGCTAGGTCATTTGACCTGATACAACATCGGATTT	3 (0.000057%)
GGCATTCGTATTTCATAGTCAGAGGTGAAATTCTTGGATTTATGAAAGACGAACAACTGCGAAAGCATTTGCCAAGGATGTTTTCATTAATCAAGAACGA	4 (0.000077%)
GGCCACGCTTTCACGGTTCGTATTCGTACTGAAAATCAGAATCAAACGAGCTTTTACCCTTTTGTTCCACACGAGATTTCTGTTCTCGTTGAGCTCATCT	30 (0.000575%)
GGCCGCACGCGCGCTACACTGATGTATTCAACGAGTTCACACCTTGGCCGACAGGCCCGGGTAATCTTTGAAATTTCATCGTGATGGGGATAGATCGATC	3 (0.000057%)
GGCCGTCGGGGCAAGAGCCAGGCCTCGATGAGTAGGAGGGCGCGGCGGTCGCTGCAAAACCTAGGGCGCGAGCCCGGGCGGAGCGGCCGTCGGTGCAGAT	22 (0.000422%)
GGCCTACCATGGTGGTAACGGGTGACGGAGAATTAGGGTTCGATTCCGGAGAGGGAGCCTGAGAAACGGCTACCACATCCAAGGAAGGCAGCAGGCGCGC	17 (0.000326%)
GGCGAATGCCAGCCGTTCGTTTGCATGTTCCTTGACACTTTTCGTGCCGGGGTTTTGTGATATCCGGAAGCAACGCGCACGACAAGACCGAGATAAAAGC	43 (0.000824%)
GGCGAGACAAGGGTTCACATTTCGTTCATCACCCTTGGCCGGCTATCGAACAGCCGGACTCCCATCAAAAGATGGTTGCCAAGAACATCTTCGTTACGGT	19 (0.000364%)
GGCGAGACAAGGGTTCACATTTCGTTCATCACCCTTGGCCGGCTTTCGAACAGCCGGACTCCCATCAAAAGATGGTTGCCAAGAACATCTTCGTTACGGT	20 (0.000383%)
GGCGGACCGGATTGCTCCGTTCCGCATCCGACCAGGACGCATCGCCGGCCCCCATCCGCTTCCCTCCCGACAATTTCAAGCACTCTTTGACTCTCTTTTC	12 (0.000230%)
GGCGGCACATCTGTTAAAAGATAACGCAGGTGTCCTAAGATGAGCTCAACGAGAACAGAAATCTCGTGTGGAACAAAAGGGTAAAAGCTCGTTTGATTCT	10 (0.000192%)
GGCGTAAGAATTGTATCCTTGTTAGAAGACACAAAGCCAAAGACTCATATGGACTTTGGCTACACCATGAAAGCTTTGAGAAGCAAGAAGAAGGTTGGTT	10 (0.000192%)
GGCGTGCCTCGGCATCAGCGTGCTCCGGGCGTCGGCCTGTGGGCTCCCCATTCGACCCGTCTTGAAACACGGACCAAGGAGTCTGACATGTGTGCGAGTC	14 (0.000268%)
GGCTCATTAAATCAGTTATAGTTTGTTTGATGGTAACTACTACTCGGATAACCGTAGTAATTCTAGAGCTAATACGTGCAACAAACCCCGACTTATGGAA	9 (0.000172%)
GGCTGGGGCGGCACATCTGTTAAAAGATAACGCAGGTGTCCTAAGATGAGCTCAACGAGAACAGAAATCTCGTGTGGAACAAAAGGGTAAAAGCTCGTTT	3 (0.000057%)
GGCTGTCCCGAGTGTGAGCGAGGTGTGAGTGTCGCCCATGGGCATCGACACCTTGCGGCTAGGAACTGGAACGAGACGGGTAGCAAAGATTTCGAGTAGC	12 (0.000230%)
GGCTGTCCCGAGTGTGAGCGAGGTGTGAGTGTCGCCCATGGGCATCGACACCTTGCGGCTAGGAACTGGAACGAGACGGGTGGCAAAGATTTCGAGTAGC	17 (0.000326%)
GGCTTGCTTTGAGCACTCTAATTTCTTCAAAGTAACAGCGCCGGAGGCACGACCCGGCCAATTAAGACCAGGAGCGTATCGCCGACCGAAGGGACAAGCC	7 (0.000134%)
GGGAATCCTTGTTAGTTTCTTTTCCTCCGCTTATTGATATGCTTAAACTCAGCGGGTAATCCCGCCTGACCTGGGGTCGCTATATGGACTTTGGGTCATC	53 (0.001016%)
GGGACAGTCGGGGGCATTCGTATTTCATAGTCAGAGGTGAAATTCTTGGATTTATGAAAGACGAACAACTGCGAAAGCATTTGCCAAGGATGTTTTCATT	24 (0.000460%)
GGGAGGCGAATGCCAGCCGTTCGTTTGCATGTTCCTTGACACTTTTCGTGCCGGGGTTTTGTGATATCCGGAAGCAACGCGCACGACAAGACCGAGATAA	16 (0.000307%)

GGGAGTTTGGCTGGGGCGGCACATCTGTTAAAAGATAACGCAGGTGTCCTAAGATGAGCTCAACGAGAACAGAAATCTCGTGTGGAACAAAAGGGTAAAA	29 (0.000556%)
GGGATTTAGATTGTACTCATTCCAATTACCAGACTCGAAAGAGCCCGGTATTGTTATTTATTGTCACTACCTCCCCGTGTCAGGATTGGGTAATTTGCGC	42 (0.000805%)
GGGCAAGTCTGGTGCCAGCAGCCGCGGTAATTCCAGCTCCAATAGCGTATATTTAAGTTGTTGCAGTTAAAAAGCTCGTAGTTGAACCTTGGGATGGGTC	156 (0.002990%)
GGGCAGAGCCCGCGTCGACCTTTTATCTAATAAATGCGTCCCTTCCATAAGTCGGGGTTTGTTGCACGTATTAGCTCTAGAATTACTACGGTTATCCGAG	45 (0.000862%)
GGGCATCGACACCTTGCGGCTAGGAACTGGAACGAGACGGGTGGCAAAGATTTCGAGTAGCACTTCATACTACCGTGGGTTTTTTAAACCTTCCGAGTTT	12 (0.000230%)
GGGCATTCGTATTTCATAGTCAGAGGTGAAATTCTTGGATTTATGAAAGACGAACAACTGCGAAAGCATTTGCCAAGGATGTTTTCATTAATCAAGAACG	44 (0.000843%)
GGGCCGCACGCGCGCTACACTGATGTATTCAACGAGTTCACACCTTGGCCGACAGGCCCGGGTAATCTTTGAAATTTCATCGTGATGGGGATAGATCGAT	7 (0.000134%)
GGGCGGCACATCTGTTAAAAGATAACGCAGGTGTCCTAAGATGAGCTCAACGAGAACAGAAATCTCGTGTGGAACAAAAGGGTAAAAGCTCGTTTGATTC	11 (0.000211%)
GGGCGGTGTGTACAAAGGGCAGGGACGTAGTCAACGCGAGCTGATGACTCGCGCTTACTAGGAATTCCTCGTTGAAGACCAACAATTGCAATGATCGATC	25 (0.000479%)
GGGCGTCGGCCTGTGGGCTCCCCATTCGACCCGTCTTGAAACACGGACCAAGGAGTCTGACATGTGTGCGAGTCAACGGGTGAGTAAACCCGTAAGGCGC	5 (0.000096%)
GGGGAGGCGAATGCCAGCCGTTCGTTTGCATGTTCCTTGACACTTTTCGTGCCGGGGTTTTGTGATATCCGGAAGCAACGCGCACGACAAGACCGAGATA	36 (0.000690%)
GGGGAGTTTGGCTGGGGCGGCACATCTGTTAAAAGATAACGCAGGTGTCCTAAGATGAGCTCAACGAGAACAGAAATCTCGTGTGGAACAAAAGGGTAAA	34 (0.000652%)
GGGGCATTCGTATTTCATAGTCAGAGGTGAAATTCTTGGATTTATGAAAGACGAACAACTGCGAAAGCATTTGCCAAGGATGTTTTCATTAATCAAGAAC	37 (0.000709%)
GGGGGCATTCGTATTTCATAGTCAGAGGTGAAATTCTTGGATTTATGAAAGACGAACAACTGCGAAAGCATTTGCCAAGGATGTTTTCATTAATCAAGAA	18 (0.000345%)
GGGTGACGGAGAATTAGGGTTCGATTCCGGAGAGGGAGCCTGAGAAACGGCTACCACATCCAAGGAAGGCAGCAGGCGCGCAAATTACCCAATCCTGACA	13 (0.000249%)
GGGTGGTTCGCCGCCCGCGACGTCGCGAGAAGTCCACTAAACCTTATCATTTAGAGGAAGGAGAAGTCGTAACAAGGTTTCCGTAGGTGAACCTGCGGAA	162 (0.003105%)
GGGTTCACATTTCGTTCATCACCCTTGGCCGGCTTTCGAACAGCCGGACTCCCATCAAAAGATGGTTGCCAAGAACATCTTCGTTACGGTTTGCTAATTC	3 (0.000057%)
GGGTTGCGGTTTAAGTTGTTATACTCAATCATACACATGA	417 (0.003197%)
GGGTTTACTCACCCGTTGACTCGCACACATGTCAGACTCCTTGGTCCGTGTTTCAAGACGGGTCGAATGGGGAGCCCACAGGCCGACGCCCGGAGCACGC	5 (0.000096%)
GGTAAATTTTCATAATTTTTTGACACCTCTAGCTAGGTCATTTGACCTGATACAACATCGGATTTTCATGGTCTAGTTGGGGCTCCGTGGGCATATTTGA	5 (0.000096%)
GGTAACGGGTGACGGAGAATTAGGGTTCGATTCCGGAGAGGGAGCCTGAGAAACGGCTACCACATCCAAGGAAGGCAGCAGGCGCGCAAATTACCCAATC	90 (0.001725%)
GGTAATTCCAGCTCCAATAGCGTATATTTAAGTTGTTGCAGTTAAAAAGCTCGTAGTTGAACCTTGGGATGGGTCGGCCGGTCCGCCTTTGGTGTGCATT	126 (0.002415%)
GGTAGGAGCGACGGGCGGTGTGTACAAAGGGCAGGGACGTAGTCAACGCGAGCTGATGACTCGCGCTTACTAGGAATTCCTCGTTGAAGACCAACAATTG	43 (0.000824%)
GGTCTGTGATGCCCTTAGATGTTCTGGGCCGCACGCGCGCTACACTGATGTATTCAACGAGTTCACACCTTGGCCGACAGGCCCGGGTAATCTTTGAAAT	11 (0.000211%)
GGTCTTCAACGAGGAATTCCTAGTAAGCGCGAGTCATCAGCTCGCGTTGACTACGTCCCTGCCCTTTGTACACACCGCCCGTCGCTCCTACCGATTGAAT	10 (0.000192%)
GGTGACGGAGAATTAGGGTTCGATTCCGGAGAGGGAGCCTGAGAAACGGCTACCACATCCAAGGAAGGCAGCAGGCGCGCAAATTACCCAATCCTGACAC	3 (0.000057%)
GGTGCCAGCAGCCGCGGTAATTCCAGCTCCAATAGCGTATATTTAAGTTGTTGCAGTTAAAAAGCTCGTAGTTGAACCTTGGGATGGGTCGGCCGGTCCG	14 (0.000268%)
GGTGGTTCGCCGCCCGCGACGTCGCGAGAAGTCCACTAAACCTTATCATTTAGAGGAAGGAGAAGTCGTAACAAGGTTTCCGTAGGTGAACCTGCGGAAG	21 (0.000402%)
GGTGTGAGTGTCGCCCATGGGCATCGACACCTTGCGGCTAGGAACTGGAACGAGACGGGTAGCAAAGATTTCGAGTAGCACTTCATACTACCGTGGGTTT	6 (0.000115%)
GGTGTGAGTGTCGCCCATGGGCATCGACACCTTGCGGCTAGGAACTGGAACGAGACGGGTGGCAAAGATTTCGAGTAGCACTTCATACTACCGTGGGTTT	7 (0.000134%)
GGTTCACATTTCGTTCATCACCCTTGGCCGGCTATCGAACAGCCGGACTCCCATCAAAAGATGGTTGCCAAGAACATCTTCGTTACGGTTTGCTAATTCT	9 (0.000172%)
GGTTCACATTTCGTTCATCACCCTTGGCCGGCTTTCGAACAGCCGGACTCCCATCAAAAGATGGTTGCCAAGAACATCTTCGTTACGGTTTGCTAATTCT	5 (0.000096%)
GGTTCGCCGCCCGCGACGTCGCGAGAAGTCCACTAAACCTTATCATTTAGAGGAAGGAGAAGTCGTAACAAGGTTTCCGTAGGTGAACCTGCGGAAGGAT	14 (0.000268%)
GGTTCGTATTCGTACTGAAAATCAGAATCAAACGAGCTTTTACCCTTTTGTTCCACACGAGATTTCTGTTCTCGTTGAGCTCATCTTAGGACACCTGCGT	3 (0.000057%)
GGTTGCGGTTTAAGTTCTTATACTCAATCATACACATGAC	9307 (0.071346%)
GGTTGCGGTTTAAGTTGTTATACTCAATCATACACATGAC	901 (0.006907%)
GGTTTAAGTTCTTATACTCAATCATACACATGACATCAAG	1481 (0.011353%)
GGTTTAAGTTGTTATACTCAATCATACACATGACAACAAGTCATATTCGACTCCAAAACACTAACCAACCTTCTTCTTGCTTCTCAAAGCTTTCATGGTG	123 (0.002357%)
GGTTTAAGTTGTTATACTCAATCATACACATGACATCAAG	2143 (0.016428%)
GGTTTACTCACCCGTTGACTCGCACACATGTCAGACTCCTTGGTCCGTGTTTCAAGACGGGTCGAATGGGGAGCCCACAGGCCGACGCCCGGAGCACGCT	20 (0.000383%)
GTAAATTTTCATAATTTTTTGACACCTCTAGCTAGGTCATTTGACCTGATACAACATCGGATTTTCATGGTCTAGTTGGGGCTCCGTGGGCATATTTGAT	17 (0.000326%)
GTAACGGGTGACGGAGAATTAGGGTTCGATTCCGGAGAGGGAGCCTGAGAAACGGCTACCACATCCAAGGAAGGCAGCAGGCGCGCAAATTACCCAATCC	19 (0.000364%)
GTAACTTCGGGAAAAGGATTGGCTCTGAGGGCTGGGCTCGGGGGTCCCAGTTCCGAACCCGTCGGCTGTCAGCGGACTGCTCGAGCTGCTTCCGCGGCGA	10 (0.000192%)

GTAAGAATTGTATCCTTGTTAGAAGACACAAAGCCAAAGACTCATATGGACTTTGGCTACACCATGAAAGCTTTGAGAAGCAAGAAGAAGGTTGGTTAGT	58 (0.001112%)	
GTAAGTATGAACGAATTCAGACTGTGAAACTGCGAATGGCTCATTAAATCAGTTATAGTTTGTTTGATGGTAACTACTACTCGGATAACCGTAGTAATTC	30 (0.000575%)	
GTAATTCCAGCTCCAATAGCGTATATTTAAGTTGTTGCAGTTAAAAAGCTCGTAGTTGAACCTTGGGATGGGTCGGCCGGTCCGCCTTTGGTGTGCATTG	19 (0.000364%)	
GTACGCTCCAGGCGTCCTTGGCTCGGATTTAGGCCAACCGCGTGCGGTAACACACGGGAGACCAGCTTCCGTCCCGCATCAGCAAAGGATGGTGAGGGAC	12 (0.000230%)	
GTACTCATTCCAATTACCAGACTCGAAAGAGCCCGGTATTGTTATTTATTGTCACTACCTCCCCGTGTCAGGATTGGGTAATTTGCGCGCCTGCTGCCTT	3 (0.000057%)	
GTACTGAAAATCAGAATCAAACGAGCTTTTACCCTTTTGTTCCACACGAGATTTCTGTTCTCGTTGAGCTCATCTTAGGACACCTGCGTTATCTTTTAAC	4 (0.000077%)	
GTAGGAGCGACGGGCGGTGTGTACAAAGGGCAGGGACGTAGTCAACGCGAGCTGATGACTCGCGCTTACTAGGAATTCCTCGTTGAAGACCAACAATTGC	114 (0.002185%)	
GTAGGCTTGCTTTGAGCACTCTAATTTCTTCAAAGTAACAGCGCCGGAGGCACGACCCGGCCAATTAAGACCAGGAGCGTATCGCCGACCGAAGGGACAA	33 (0.000632%)	
GTAGTCATATGCTTGTCTCAAAGATTAAGCCATGCATGTGTAAGTATGAACGAATTCAGACTGTGAAACTGCGAATGGCTCATTAAATCAGTTATAGTTT	98 (0.001878%)	
GTAGTGTTTCCTTGTTAGAAGACACAAAGCCAAAGACTCATATGGACTTTGGCTACACCATGAAAGCTTTGAGAAGCAAGAAGAAGGTTGGTTAGTGTTT	18 (0.000345%)	
GTATCCAGAGCGTAGGCTTGCTTTGAGCACTCTAATTTCTTCAAAGTAACAGCGCCGGAGGCACGACCCGGCCAATTAAGACCAGGAGCGTATCGCCGAC	16 (0.000307%)	
GTATCCTTGTTAGAAGACACAAAGCCAAAGACTCATATGGACTTTGGCTACACCATGAAAGCTTTGAGAAGCAAGAAGAAGGTTGGTTAGTGTTTTGGAG	22 (0.000422%)	
GTATGAACGAATTCAGACTGTGAAACTGCGAATGGCTCATTAAATCAGTTATAGTTTGTTTGATGGTAACTACTACTCGGATAACCGTAGTAATTCTAGA	10 (0.000192%)	
GTATTCGTACTGAAAATCAGAATCAAACGAGCTTTTACCCTTTTGTTCCACACGAGATTTCTGTTCTCGTTGAGCTCATCTTAGGACACCTGCGTTATCT	4 (0.000077%)	
GTATTTCATAGTCAGAGGTGAAATTCTTGGATTTATGAAAGACGAACAACTGCGAAAGCATTTGCCAAGGATGTTTTCATTAATCAAGAACGAAAGTTGG	5 (0.000096%)	
GTCAGAGGTGAAATTCTTGGATTTATGAAAGACGAACAACTGCGAAAGCATTTGCCAAGGATGTTTTCATTAATCAAGAACGAAAGTTGGGGGCTCGAAG	20 (0.000383%)	
GTCAGGTGGGGAGTTTGGCTGGGGCGGCACATCTGTTAAAAGATAACGCAGGTGTCCTAAGATGAGCTCAACGAGAACAGAAATCTCGTGTGGAACAAAA	12 (0.000230%)	
GTCATATGCTTGTCTCAAAGATTAAGCCATGCATGTGTAAGTATGAACGAATTCAGACTGTGAAACTGCGAATGGCTCATTAAATCAGTTATAGTTTGTT	57 (0.001092%)	
GTCATATTCGACTCCAAAACACTAACCAACCTTCTTCTTGCTTCTCAAAGCTTTCATGGTGTAGCCAAAGTCCATATGAGTCTTTGGCTTTGTGTCTTCT	260 (0.004983%)	
GTCCATATGAGTCTTTGGCTTTGTGTCTTCTAACAAGGAT	7093 (0.054373%)	
GTCCCGAGTGTGAGCGAGGTGTGAGTGTCGCCCATGGGCATCGACACCTTGCGGCTAGGAACTGGAACGAGACGGGTAGCAAAGATTTCGAGTAGCACTT	16 (0.000307%)	
GTCCCGAGTGTGAGCGAGGTGTGAGTGTCGCCCATGGGCATCGACACCTTGCGGCTAGGAACTGGAACGAGACGGGTGGCAAAGATTTCGAGTAGCACTT	28 (0.000537%)	
GTCCCTCACCATCCTTTGCTGATGCGGGACGGAAGCTGGTCTCCCGTGTGTTACCGCACGCGGTTGGCCTAAATCCGAGCCAAGGACGCCTGGAGCGTAC	9 (0.000172%)	
GTCCGACTTTGTGAAATGACTTGAGAGGTGTAGGATAAGTGGGAGCTTCGGCGCAAGTGAAATACCACTACTTTTAACGTTATTTTACTTACTCCGTGAA	12 (0.000230%)	
GTCGAAATCCGCTAAGGAGTGTGTAACAACTCACCTGCCGAATCAACTAGCCCCGAAAATGGATGGCGCTTAAGCGCGCGACCTATACCCGGCCGTCGGG	73 (0.001399%)	
GTCGACCTTTTATCTAATAAATGCGTCCCTTCCATAAGTCGGGGTTTGTTGCACGTATTAGCTCTAGAATTACTACGGTTATCCGAGTAGTAGTTACCAT	23 (0.000441%)	
GTCGAGTTATCATGAATCATCAGAGCAACGGGCAGAGCCCGCGTCGACCTTTTATCTAATAAATGCGTCCCTTCCATAAGTCGGGGTTTGTTGCACGTAT	462 (0.008854%)	
GTCGCCCATGGGCATCGACACCTTGCGGCTAGGAACTGGAACGAGACGGGTGGCAAAGATTTCGAGTAGCACTTCATACTACCGTGGGTTTTTTAAACCT	30 (0.000575%)	
GTCGGCCTGTGGGCTCCCCATTCGACCCGTCTTGAAACACGGACCAAGGAGTCTGACATGTGTGCGAGTCAACGGGTGAGTAAACCCGTAAGGCGCAAGG	28 (0.000537%)	
GTCGGGGGCATTCGTATTTCATAGTCAGAGGTGAAATTCTTGGATTTATGAAAGACGAACAACTGCGAAAGCATTTGCCAAGGATGTTTTCATTAATCAA	6 (0.000115%)	
GTCGGTACGCTCCAGGCGTCCTTGGCTCGGATTTAGGCCAACCGCGTGCGGTAACACACGGGAGACCAGCTTCCGTCCCGCATCAGCAAAGGATGGTGAG	8 (0.000153%)	
GTCGTCCCTCACCATCCTTTGCTGATGCGGGACGGAAGCTGGTCTCCCGTGTGTTACCGCACGCGGTTGGCCTAAATCCGAGCCAAGGACGCCTGGAGCG	683 (0.013089%)	
GTCTCCGGGGAGGCGAATGCCAGCCGTTCGTTTGCATGTTCCTTGACACTTTTCGTGCCGGGGTTTTGTGATATCCGGAAGCAACGCGCACGACAAGACC	8 (0.000153%)	
GTCTGGTGCCAGCAGCCGCGGTAATTCCAGCTCCAATAGCGTATATTTAAGTTGTTGCAGTTAAAAAGCTCGTAGTTGAACCTTGGGATGGGTCGGCCGG	18 (0.000345%)	
GTCTGTGATGCCCTTAGATGTTCTGGGCCGCACGCGCGCTACACTGATGTATTCAACGAGTTCACACCTTGGCCGACAGGCCCGGGTAATCTTTGAAATT	21 (0.000402%)	
GTCTTCAACGAGGAATTCCTAGTAAGCGCGAGTCATCAGCTCGCGTTGACTACGTCCCTGCCCTTTGTACACACCGCCCGTCGCTCCTACCGATTGAATG	13 (0.000249%)	
GTGACGGAGAATTAGGGTTCGATTCCGGAGAGGGAGCCTGAGAAACGGCTACCACATCCAAGGAAGGCAGCAGGCGCGCAAATTACCCAATCCTGACACG	8 (0.000153%)	
GTGAGCGAGGTGTGAGTGTCGCCCATGGGCATCGACACCTTGCGGCTAGGAACTGGAACGAGACGGGTAGCAAAGATTTCGAGTAGCACTTCATACTACC	22 (0.000422%)	
GTGAGCGAGGTGTGAGTGTCGCCCATGGGCATCGACACCTTGCGGCTAGGAACTGGAACGAGACGGGTGGCAAAGATTTCGAGTAGCACTTCATACTACC	23 (0.000441%)	
GTGAGTGTCGCCCATGGGCATCGACACCTTGCGGCTAGGAACTGGAACGAGACGGGTAGCAAAGATTTCGAGTAGCACTTCATACTACCGTGGGTTTTTT	25 (0.000479%)	
GTGAGTGTCGCCCATGGGCATCGACACCTTGCGGCTAGGAACTGGAACGAGACGGGTGGCAAAGATTTCGAGTAGCACTTCATACTACCGTGGGTTTTTT	50 (0.000958%)	
GTGATGCCCTTAGATGTTCTGGGCCGCACGCGCGCTACACTGATGTATTCAACGAGTTCACACCTTGGCCGACAGGCCCGGGTAATCTTTGAAATTTCAT	22 (0.000422%)	
GTGCCAGCAGCCGCGGTAATTCCAGCTCCAATAGCGTATATTTAAGTTGTTGCAGTTAAAAAGCTCGTAGTTGAACCTTGGGATGGGTCGGCCGGTCCGC	26 (0.000498%)	

GTGCCTCGGCATCAGCGTGCTCCGGGCGTCGGCCTGTGGGCTCCCCATTCGACCCGTCTTGAAACACGGACCAAGGAGTCTGACATGTGTGCGAGTCAAC	15 (0.000287%)	
GTGCTCCGGGCGTCGGCCTGTGGGCTCCCCATTCGACCCGTCTTGAAACACGGACCAAGGAGTCTGACATGTGTGCGAGTCAACGGGTGAGTAAACCCGT	17 (0.000326%)	
GTGGCCTACCATGGTGGTAACGGGTGACGGAGAATTAGGGTTCGATTCCGGAGAGGGAGCCTGAGAAACGGCTACCACATCCAAGGAAGGCAGCAGGCGC	9 (0.000172%)	
GTGGGGAGTTTGGCTGGGGCGGCACATCTGTTAAAAGATAACGCAGGTGTCCTAAGATGAGCTCAACGAGAACAGAAATCTCGTGTGGAACAAAAGGGTA	8 (0.000153%)	
GTGGGTGGTTCGCCGCCCGCGACGTCGCGAGAAGTCCACTAAACCTTATCATTTAGAGGAAGGAGAAGTCGTAACAAGGTTTCCGTAGGTGAACCTGCGG	76 (0.001457%)	
GTGGTTCGCCGCCCGCGACGTCGCGAGAAGTCCACTAAACCTTATCATTTAGAGGAAGGAGAAGTCGTAACAAGGTTTCCGTAGGTGAACCTGCGGAAGG	17 (0.000326%)	
GTGTAAGTATGAACGAATTCAGACTGTGAAACTGCGAATGGCTCATTAAATCAGTTATAGTTTGTTTGATGGTAACTACTACTCGGATAACCGTAGTAAT	8 (0.000153%)	
GTGTATCCTTGTTAGAAGACACAAAGCCAAAGACTCATATGGACTTTGGCTACACCATGAAAGCTTTGAGAAGCAAGAAGAAGGTTGGTTAGTGTTTTGG	5 (0.000096%)	
GTGTCGCCCATGGGCATCGACACCTTGCGGCTAGGAACTGGAACGAGACGGGTGGCAAAGATTTCGAGTAGCACTTCATACTACCGTGGGTTTTTTAAAC	10 (0.000192%)	
GTGTGAGCGAGGTGTGAGTGTCGCCCATGGGCATCGACACCTTGCGGCTAGGAACTGGAACGAGACGGGTAGCAAAGATTTCGAGTAGCACTTCATACTA	34 (0.000652%)	
GTGTGAGCGAGGTGTGAGTGTCGCCCATGGGCATCGACACCTTGCGGCTAGGAACTGGAACGAGACGGGTGGCAAAGATTTCGAGTAGCACTTCATACTA	35 (0.000671%)	
GTGTGAGTGTCGCCCATGGGCATCGACACCTTGCGGCTAGGAACTGGAACGAGACGGGTAGCAAAGATTTCGAGTAGCACTTCATACTACCGTGGGTTTT	14 (0.000268%)	
GTGTGAGTGTCGCCCATGGGCATCGACACCTTGCGGCTAGGAACTGGAACGAGACGGGTGGCAAAGATTTCGAGTAGCACTTCATACTACCGTGGGTTTT	24 (0.000460%)	
GTGTTTCCTTGTTAGAAGACACAAAGCCAAAGACTCATATGGACTTTGGCTACACCATGAAAGCTTTGAGAAGCAAGAAGAAGGTTGGTTAGTGTTTTGG	35 (0.000671%)	
GTTAAAAGATAACGCAGGTGTCCTAAGATGAGCTCAACGAGAACAGAAATCTCGTGTGGAACAAAAGGGTAAAAGCTCGTTTGATTCTGATTTTCAGTAC	24 (0.000460%)	
GTTAAGGGATTTAGATTGTACTCATTCCAATTACCAGACTCGAAAGAGCCCGGTATTGTTATTTATTGTCACTACCTCCCCGTGTCAGGATTGGGTAATT	104 (0.001993%)	
GTTAGAAGACACAAAGCCAAAGACTCATATGGACTTTGGCTACACCATGAAAGCTTTGAGAAGCAAGAAGAAGGTTGGTTAGTGTTTTGGAGTCGAATAT	81 (0.001552%)	
GTTAGTTTCTTTTCCTCCGCTTATTGATATGCTTAAACTCAGCGGGTAATCCCGCCTGACCTGGGGTCGCTATATGGACTTTGGGTCATCTACAGCTTCC	25 (0.000479%)	
GTTATACTCAATCATACACATGACAACAAGTCATATTCGACTCCAAAACACTAACCAACCTTCTTCTTGCTTCTCAAAGCTTTCATGGTGTAGCCAAAGT	122 (0.002338%)	
GTTATAGTTTGTTTGATGGTAACTACTACTCGGATAACCGTAGTAATTCTAGAGCTAATACGTGCAACAAACCCCGACTTATGGAAGGGACGCATTTATT	11 (0.000211%)	
GTTATCATGAATCATCAGAGCAACGGGCAGAGCCCGCGTCGACCTTTTATCTAATAAATGCGTCCCTTCCATAAGTCGGGGTTTGTTGCACGTATTAGCT	298 (0.005711%)	
GTTATCCCATGCTAATGTATCCAGAGCGTAGGCTTGCTTTGAGCACTCTAATTTCTTCAAAGTAACAGCGCCGGAGGCACGACCCGGCCAATTAAGACCA	58 (0.001112%)	
GTTCACATTTCGTTCATCACCCTTGGCCGGCTATCGAACAGCCGGACTCCCATCAAAAGATGGTTGCCAAGAACATCTTCGTTACGGTTTGCTAATTCTC	13 (0.000249%)	
GTTCACATTTCGTTCATCACCCTTGGCCGGCTTTCGAACAGCCGGACTCCCATCAAAAGATGGTTGCCAAGAACATCTTCGTTACGGTTTGCTAATTCTC	15 (0.000287%)	
GTTCACCACCGCATGTCGGTACGCTCCAGGCGTCCTTGGCTCGGATTTAGGCCAACCGCGTGCGGTAACACACGGGAGACCAGCTTCCGTCCCGCATCAG	95 (0.001821%)	
GTTCCATCGACCAGAGGCTGTTCACCTTGGAGACCTGATGCGGTTATGAGTACGACCGGGCGTGAGCGGCACTCGGTCCTCCGGATTTTCAAGGGCCGCC	41 (0.000786%)	
GTTCGCCGCCCGCGACGTCGCGAGAAGTCCACTAAACCTTATCATTTAGAGGAAGGAGAAGTCGTAACAAGGTTTCCGTAGGTGAACCTGCGGAAGGATC	21 (0.000402%)	
GTTCGCTCGCCGTTACTAAGGGAATCCTTGTTAGTTTCTTTTCCTCCGCTTATTGATATGCTTAAACTCAGCGGGTAATCCCGCCTGACCTGGGGTCGCT	1057 (0.020257%)	
GTTCGTATTCGTACTGAAAATCAGAATCAAACGAGCTTTTACCCTTTTGTTCCACACGAGATTTCTGTTCTCGTTGAGCTCATCTTAGGACACCTGCGTT	12 (0.000230%)	
GTTCTGGGCCGCACGCGCGCTACACTGATGTATTCAACGAGTTCACACCTTGGCCGACAGGCCCGGGTAATCTTTGAAATTTCATCGTGATGGGGATAGA	42 (0.000805%)	
GTTCTTATACTCAATCATACACATGACATCAAGTCATATTCGACTCCAAAACACTAACCAACCTTCTTCTTGCTTCTCAAAGCTTTCATGGTGTAGCCAA	107 (0.002051%)	
GTTGAGCTTGACTCTAGTCCGACTTTGTGAAATGACTTGAGAGGTGTAGGATAAGTGGGAGCTTCGGCGCAAGTGAAATACCACTACTTTTAACGTTATT	46 (0.000882%)	
GTTGCGGTTTAAGTTCTTATACTCAATCATACACATGACA	2282 (0.017493%)	
GTTGCGGTTTAAGTTGTTATACTCAATCATACACATGACA	2448 (0.018766%)	
GTTGGTCTTCAACGAGGAATTCCTAGTAAGCGCGAGTCATCAGCTCGCGTTGACTACGTCCCTGCCCTTTGTACACACCGCCCGTCGCTCCTACCGATTG	184 (0.003526%)	
GTTGTTATACTCAATCATACACATGACATCAAGTCATATT	221 (0.001694%)	
GTTTAAGTTCTTATACTCAATCATACACATGACATCAAGT	2186 (0.016757%)	
GTTTAAGTTGTTATACTCAATCATACACATGACAACAAGTCATATTCGACTCCAAAACACTAACCAACCTTCTTCTTGCTTCTCAAAGCTTTCATGGTGT	147 (0.002817%)	
GTTTAAGTTGTTATACTCAATCATACACATGACATCAAGT	1025 (0.007857%)	
GTTTACTCACCCGTTGACTCGCACACATGTCAGACTCCTTGGTCCGTGTTTCAAGACGGGTCGAATGGGGAGCCCACAGGCCGACGCCCGGAGCACGCTG	27 (0.000517%)	
GTTTCCTTGTTAGAAGACACAAAGCCAAAGACTCATATGGACTTTGGCTACACCATGAAAGCTTTGAGAAGCAAGAAGAAGGTTGGTTAGTGTTTTGGAG	48 (0.000920%)	
GTTTCTTTTCCTCCGCTTATTGATATGCTTAAACTCAGCGGGTAATCCCGCCTGACCTGGGGTCGCTATATGGACTTTGGGTCATCTACAGCTTCCGGAC	26 (0.000498%)	
GTTTGATGGTAACTACTACTCGGATAACCGTAGTAATTCTAGAGCTAATACGTGCAACAAACCCCGACTTATGGAAGGGACGCATTTATTAGATAAAAGG	24 (0.000460%)	

GTTTGGCTGGGGCGGCACATCTGTTAAAAGATAACGCAGGTGTCCTAAGATGAGCTCAACGAGAACAGAAA<br>TCTCGTGTGGAACAAAAGGGTAAAAGCTC	35 (0.000671%)	
GTTTGTTTGATGGTAACTACTACTCGGATAACCGTAGTAATTCTAGAGCTAATACGTGCAACAAACCCCGA<br>CTTATGGAAGGGACGCATTTATTAGATAA	35 (0.000671%)	
TAAAAGATAACGCAGGTGTCCTAAGATGAGCTCAACGAGAACAGAAATCTCGTGTGGAACAAAAGGGTAAA<br>AGCTCGTTTGATTCTGATTTTCAGTACGA	17 (0.000326%)	
TAAAAGCCTAAGTAGTGTTTCCTTGTTAGAAGACACAAAGCCAAAGACTCATATGGACTTTGGCTACACCA<br>TGAAAGCTTTGAGAAGCAAGAAGAAGGTT	37 (0.000709%)	
TAAAGGCGTAAGAATTGTATCCTTGTTAAAAGACACAAAGCCAAAGACTCATATGGACTTTGGCTACACCA<br>TGAAAGCTTTGAGAAGCAAGAAGAAGGTT	62 (0.001188%)	
TAAAGGCGTAAGAATTGTATCCTTGTTAGAAGACACAAAGCCAAAGACTCATATGGACTTTGGCTACACCA<br>TGAAAGCTTTGAGAAGCAAGAAGAAGGTT	138 (0.002645%)	
TAAATCAGTTATAGTTTGTTTGATGGTAACTACTACTCGGATAACCGTAGTAATTCTAGAGCTAATACGTG<br>CAACAAACCCCGACTTATGGAAGGGACGC	9 (0.000172%)	
TAAATTTTCATAATTTTTTGACACCTCTAGCTAGGTCATTTGACCTGATACAACATCGGATTTTCATGGTC<br>TAGTTGGGGCTCCGTGGGCATATTTGATG	9 (0.000172%)	
TAACAGGGACAGTCGGGGGCATTCGTATTTCATAGTCAGAGGTGAAATTCTTGGATTTATGAAAGACGAAC<br>AACTGCGAAAGCATTTGCCAAGGATGTTT	45 (0.000862%)	
TAACAGGTCTGTGATGCCCTTAGATGTTCTGGGCCGCACGCGCGCTACACTGATGTATTCAACGAGTTCAC<br>ACCTTGGCCGACAGGCCCGGGTAATCTTT	37 (0.000709%)	
TAACGCCTCGAAGAACTAATGGCAGCCCACGCAAGGCAAGCCCATTCTCCTCGACGATTCAGCAGTTTTTG<br>TCCGAGAACTGCTGAGAAAACTCGGAAAA	11 (0.000211%)	
TAACGGCGTGCCTCGGCATCAGCGTGCTCCGGGCGTCGGCCTGTGGGCTCCCCATTCGACCCGTCTTGAAA<br>CACGGACCAAGGAGTCTGACATGTGTGCG	34 (0.000652%)	
TAACGGGTGACGGAGAATTAGGGTTCGATTCCGGAGAGGGAGCCTGAGAAACGGCTACCACATCCAAGGAA<br>GGCAGCAGGCGCGCAAATTACCCAATCCT	13 (0.000249%)	
TAAGAATTGTATCCTTGTTAGAAGACACAAAGCCAAAGACTCATATGGACTTTGGCTACACCATGAAAGCT<br>TTGAGAAGCAAGAAGAAGGTTGGTTAGTG	101 (0.001936%)	
TAAGCCATGCATGTGTAAGTATGAACGAATTCAGACTGTGAAACTGCGAATGGCTCATTAAATCAGTTATA<br>GTTTGTTTGATGGTAACTACTACTCGGAT	5 (0.000096%)	
TAAGCGCGCGACCTATACCCGGCCGTCGGGGCAAGAGCCAGGCCTCGATGAGTAGGAGGGCGCGGCGGTCG<br>CTGCAAAACCTAGGGCGCGAGCCCGGGCG	10 (0.000192%)	
TAAGGATACTAAATCCTATTTTCTGGTAAATTTTCATAATTTTTTGACACCTCTAGCTAGGTCATTTGACC<br>TGATACAACATCGGATTTTCATGGTCTAG	12 (0.000230%)	
TAAGGGATTTAGATTGTACTCATTCCAATTACCAGACTCGAAAGAGCCCGGTATTGTTATTTATTGTCACT<br>ACCTCCCCGTGTCAGGATTGGGTAATTTG	36 (0.000690%)	
TAAGTAGTGTTTCCTTGTTAGAAGACACAAAGCCAAAGACTCATATGGACTTTGGCTACACCATGAAAGCT<br>TTGAGAAGCAAGAAGAAGGTTGGTTAGTG	52 (0.000997%)	
TAAGTATGAACGAATTCAGACTGTGAAACTGCGAATGGCTCATTAAATCAGTTATAGTTTGTTTGATGGTA<br>ACTACTACTCGGATAACCGTAGTAATTCT	5 (0.000096%)	
TAAGTTCTTATACTCAATCATACACATGACATCAAGTCATATTCGACTCCAAAACACTAACCAACCTTCTT<br>CTTGCTTCTCAAAGCTTTCATGGTGTAGC	17 (0.000326%)	
TAAGTTGTTATACTCAATCATACACATGACATCAAGTCAT	91 (0.000698%)	
TAATGATTAACAGGGACAGTCGGGGGCATTCGTATTTCATAGTCAGAGGTGAAATTCTTGGATTTATGAAA<br>GACGAACAACTGCGAAAGCATTTGCCAAG	1509 (0.028919%)	
TAATTCCAGCTCCAATAGCGTATATTTAAGTTGTTGCAGTTAAAAAGCTCGTAGTTGAACCTTGGGATGGG<br>TCGGCCGGTCCGCCTTTGGTGTGCATTGG	15 (0.000287%)	
TAATTTTTTGACACCTCTAGCTAGGTCATTTGACCTGATACAACATCGGATTTTCATGGTCTAGTTGGGGC<br>TCCGTGGGCATATTTGATGCAAACTTGAC	6 (0.000115%)	
TACACATGACATCAAGTCATATTCGACTCCAAAACACTAACCAACCTTCTTCTTGCTTCTCAAAGCTTTCA<br>TGGTGTAGCCAAAGTCCATATGAGTCTTT	11 (0.000211%)	
TACACCATGAAAGCTTTGAGAAGCAAGAAGAAGGTTGGTTAGTGTTTTGGAGTCGAATATGACTTGATGTC<br>ATGTGTATGATTGAGTATAACAACTTAAA	132 (0.002530%)	
TACATTGTTCCATCGACCAGAGGCTGTTCACCTTGGAGACCTGATGCGGTTATGAGTACGACCGGGCGTGA<br>GCGGCACTCGGTCCTCCGGATTTTCAAGG	3 (0.000057%)	
TACCAGACTCGAAAGAGCCCGGTATTGTTATTTATTGTCACTACCTCCCCGTGTCAGGATTGGGTAATTTG<br>CGCGCCTGCTGCCTTCCTTGGATGTGGTA	6 (0.000115%)	
TACCCGGCCGTCGGGGCAAGAGCCAGGCCTCGATGAGTAGGAGGGCGCGGCGGTCGCTGCAAAACCTAGGG<br>CGCGAGCCCGGGCGGAGCGGCCGTCGGTG	3 (0.000057%)	
TACGGGTTTACTCACCCGTTGACTCGCACACATGTCAGACTCCTTGGTCCGTGTTTCAAGACGGGTCGAAT<br>GGGGAGCCCACAGGCCGACGCCCGGAGCA	3 (0.000057%)	
TACTCAATCATACACATGACAACAAGTCATATTCGACTCCAAAACACTAACCAACCTTCTTCTTGCTTCTC<br>AAAGCTTTCATGGTGTAGCCAAAGTCCAT	12 (0.000230%)	
TACTCAATCATACACATGACATCAAGTCATATTCGACTCCAAAACACTAACCAACCTTCTTCTTGCTTCTC<br>AAAGCTTTCATGGTGTAGCCAAAGTCCAT	17 (0.000326%)	
TAGAAGACACAAAGCCAAAGACTCATATGGACTTTGGCTACACCATGAAAGCTTTGAGAAGCAAGAAGAAG<br>GTTGGTTAGTGTTTTGGAGTCGAATATGA	25 (0.000479%)	
TAGATGTTCTGGGCCGCACGCGCGCTACACTGATGTATTCAACGAGTTCACACCTTGGCCGACAGGCCCGG<br>GTAATCTTTGAAATTTCATCGTGATGGGG	18 (0.000345%)	
TAGGAGCGACGGGCGGTGTGTACAAAGGGCAGGGACGTAGTCAACGCGAGCTGATGACTCGCGCTTACTAG<br>GAATTCCTCGTTGAAGACCAACAATTGCA	14 (0.000268%)	
TAGGCCACGCTTTCACGGTTCGTATTCGTACTGAAAATCAGAATCAAACGAGCTTTTACCCTTTTGTTCCA<br>CACGAGATTTCTGTTCTCGTTGAGCTCAT	62 (0.001188%)	
TAGGCGAGACAAGGGTTCACATTTCGTTCATCACCCTTGGCCGGCTATCGAACAGCCGGACTCCCATCAAA<br>AGATGGTTGCCAAGAACATCTTCGTTACG	8 (0.000153%)	
TAGGCGAGACAAGGGTTCACATTTCGTTCATCACCCTTGGCCGGCTTTCGAACAGCCGGACTCCCATCAAA<br>AGATGGTTGCCAAGAACATCTTCGTTACG	6 (0.000115%)	
TAGGCTGTCCCGAGTGTGAGCGAGGTGTGAGTGTCGCCCATGGGCATCGACACCTTGCGGCTAGGAACTGG<br>AACGAGACGGGTAGCAAAGATTTCGAGTA	16 (0.000307%)	
TAGGCTGTCCCGAGTGTGAGCGAGGTGTGAGTGTCGCCCATGGGCATCGACACCTTGCGGCTAGGAACTGG<br>AACGAGACGGGTGGCAAAGATTTCGAGTA	21 (0.000402%)	
TAGTCAGAGGTGAAATTCTTGGATTTATGAAAGACGAACAACTGCGAAAGCATTTGCCAAGGATGTTTTCA<br>TTAATCAAGAACGAAAGTTGGGGGCTCGA	3 (0.000057%)	
TAGTCATATGCTTGTCTCAAAGATTAAGCCATGCATGTGTAAGTATGAACGAATTCAGACTGTGAAACTGC<br>GAATGGCTCATTAAATCAGTTATAGTTTG	54 (0.001035%)	

TAGTGGCCTACCATGGTGGTAACGGGTGACGGAGAATTAGGGTTCGATTCCGGAGAGGGAGCCTGAGAAACGGCTACCACATCCAAGGAAGGCAGCAGGC	6 (0.000115%)	
TAGTGTTTCCTTGTTAGAAGACACAAAGCCAAAGACTCATATGGACTTTGGCTACACCATGAAAGCTTTGAGAAGCAAGAAGAAGGTTGGTTAGTGTTTT	10 (0.000192%)	
TAGTTTCTTTTCCTCCGCTTATTGATATGCTTAAACTCAGCGGGTAATCCCGCCTGACCTGGGGTCGCTATATGGACTTTGGGTCATCTACAGCTTCCGG	5 (0.000096%)	
TATACCCGGCCGTCGGGGCAAGAGCCAGGCCTCGATGAGTAGGAGGGCGCGGCGGTCGCTGCAAAACCTAGGGCGCGAGCCCGGGCGGAGCGGCCGTCGG	12 (0.000230%)	
TATACTCAATCATACACATGACAACAAGTCATATTCGACTCCAAAACACTAACCAACCTTCTTCTTGCTTCTCAAAGCTTTCATGGTGTAGCCAAAGTCC	10 (0.000192%)	
TATACTCAATCATACACATGACATCAAGTCATATTCGACTCCAAAACACTAACCAACCTTCTTCTTGCTTCTCAAAGCTTTCATGGTGTAGCCAAAGTCC	44 (0.000843%)	
TATAGTTTGTTTGATGGTAACTACTACTCGGATAACCGTAGTAATTCTAGAGCTAATACGTGCAACAAACCCCGACTTATGGAAGGGACGCATTTATTAG	4 (0.000077%)	
TATCATGAATCATCAGAGCAACGGGCAGAGCCCGCGTCGACCTTTTATCTAATAAATGCGTCCCTTCCATAAGTCGGGGTTTGTTGCACGTATTAGCTCT	70 (0.001342%)	
TATCCAGAGCGTAGGCTTGCTTTGAGCACTCTAATTTCTTCAAAGTAACAGCGCCGGAGGCACGACCCGGCCAATTAAGACCAGGAGCGTATCGCCGACC	3 (0.000057%)	
TATCCCATGCTAATGTATCCAGAGCGTAGGCTTGCTTTGAGCACTCTAATTTCTTCAAAGTAACAGCGCCGGAGGCACGACCCGGCCAATTAAGACCAGG	6 (0.000115%)	
TATCCTTGTTAGAAGACACAAAGCCAAAGACTCATATGGACTTTGGCTACACCATGAAAGCTTTGAGAAGCAAGAAGAAGGTTGGTTAGTGTTTTGGAGT	33 (0.000632%)	
TATGACTTGATGTCATGTGTATGATTGAGTATAAGAACTT	292 (0.002238%)	
TATGATGTTATCCCATGCTAATGTATCCAGAGCGTAGGCTTGCTTTGAGCACTCTAATTTCTTCAAAGTAACAGCGCCGGAGGCACGACCCGGCCAATTA	28 (0.000537%)	
TATGCTTGTCTCAAAGATTAAGCCATGCATGTGTAAGTATGAACGAATTCAGACTGTGAAACTGCGAATGGCTCATTAAATCAGTTATAGTTTGTTTGAT	10 (0.000192%)	
TATGGACTTTGGCTACACCATGAAAGCTTTGAGAAGCAAGAAGAAGGTTGGTTAGTGTTTTGGAGTCGAATATGACTTGATGTCATGTGTATGATTGAGT	23 (0.000441%)	
TATTCGACTCCAAAACACTAACCAACCTTCTTCTTGCTTCTCAAAGCTTTCATGGTGTAGCCAAAGTCCATATGAGTCTTTGGCTTTGTGTCTTCTAACA	41 (0.000786%)	
TATTCGTACTGAAAATCAGAATCAAACGAGCTTTTACCCTTTTGTTCCACACGAGATTTCTGTTCTCGTTGAGCTCATCTTAGGACACCTGCGTTATCTT	5 (0.000096%)	
TCAAAGATTAAGCCATGCATGTGTAAGTATGAACGAATTCAGACTGTGAAACTGCGAATGGCTCATTAAATCAGTTATAGTTTGTTTGATGGTAACTACT	4 (0.000077%)	
TCAAAGCTTTCATGGTGTAGCCAAAGTCCATATGAGTCTTTGGCTTTGTGTCTTCTAACAAGGAAACACTACTTAGGCTTATAAGATGCGGTTGCGGTTT	7 (0.000134%)	
TCAAGTCATATTCGACTCCAAAACACTAACCAACCTTCTTCTTGCTTCTCAAAGCTTTCATGGTGTAGCCAAAGTCCATATGAGTCTTTGGCTTTGTGTC	48 (0.000920%)	
TCAAGTTCACCACCGCATGTCGGTACGCTCCAGGCGTCCTTGGCTCGGATTTAGGCCAACCGCGTGCGGTAACACACGGGAGACCAGCTTCCGTCCCGCA	3 (0.000057%)	
TCAATCATACACATGACAACAAGTCATATTCGACTCCAAAACACTAACCAACCTTCTTCTTGCTTCTCAAAGCTTTCATGGTGTAGCCAAAGTCCATATG	18 (0.000345%)	
TCAATCATACACATGACATCAAGTCATATTCGACTCCAAAACACTAACCAACCTTCTTCTTGCTTCTCAAAGCTTTCATGGTGTAGCCAAAGTCCATATG	30 (0.000575%)	
TCAATCGGTAGGAGCGACGGGCGGTGTGTACAAAGGGCAGGGACGTAGTCAACGCGAGCTGATGACTCGCGCTTACTAGGAATTCCTCGTTGAAGACCAA	44 (0.000843%)	
TCACATTTCGTTCATCACCCTTGGCCGGCTATCGAACAGCCGGACTCCCATCAAAAGATGGTTGCCAAGAACATCTTCGTTACGGTTTGCTAATTCTCGG	5 (0.000096%)	
TCACATTTCGTTCATCACCCTTGGCCGGCTTTCGAACAGCCGGACTCCCATCAAAAGATGGTTGCCAAGAACATCTTCGTTACGGTTTGCTAATTCTCGG	8 (0.000153%)	
TCACCACCGCATGTCGGTACGCTCCAGGCGTCCTTGGCTCGGATTTAGGCCAACCGCGTGCGGTAACACACGGGAGACCAGCTTCCGTCCCGCATCAGCA	6 (0.000115%)	
TCACCATCCTTTGCTGATGCGGGACGGAAGCTGGTCTCCCGTGTGTTACCGCACGCGGTTGGCCTAAATCCGAGCCAAGGACGCCTGGAGCGTACCGACA	6 (0.000115%)	
TCAGAATCAAACGAGCTTTTACCCTTTTGTTCCACACGAGATTTCTGTTCTCGTTGAGCTCATCTTAGGACACCTGCGTTATCTTTTAACAGATGTGCCG	17 (0.000326%)	
TCAGAGGTGAAATTCTTGGATTTATGAAAGACGAACAACTGCGAAAGCATTTGCCAAGGATGTTTTCATTAATCAAGAACGAAAGTTGGGGGCTCGAAGA	4 (0.000077%)	
TCAGCGTGCTCCGGGCGTCGGCCTGTGGGCTCCCCATTCGACCCGTCTTGAAACACGGACCAAGGAGTCTGACATGTGTGCGAGTCAACGGGTGAGTAAA	4 (0.000077%)	
TCATAGTCAGAGGTGAAATTCTTGGATTTATGAAAGACGAACAACTGCGAAAGCATTTGCCAAGGATGTTTTCATTAATCAAGAACGAAAGTTGGGGGCT	8 (0.000153%)	
TCATATGCTTGTCTCAAAGATTAAGCCATGCATGTGTAAGTATGAACGAATTCAGACTGTGAAACTGCGAATGGCTCATTAAATCAGTTATAGTTTGTTT	4 (0.000077%)	
TCATATGGACTTTGGCTACACCATGAAAGCTTTGAGAAGCAAGAAGAAGGTTGGTTAGTGTTTTGGAGTCGAATATGACTTGATGTCATGTGTATGATTG	36 (0.000690%)	
TCATATTCGACTCCAAAACACTAACCAACCTTCTTCTTGCTTCTCAAAGCTTTCATGGTGTAGCCAAAGTCCATATGAGTCTTTGGCTTTGTGTCTTCTA	68 (0.001303%)	
TCATGAATCATCAGAGCAACGGGCAGAGCCCGCGTCGACCTTTTATCTAATAAATGCGTCCCTTCCATAAGTCGGGGTTTGTTGCACGTATTAGCTCTAG	30 (0.000575%)	
TCATGTGTATGATTGAGTATAAGAACTTAAACCGCAACCG	4325 (0.033155%)	
TCATTCCAATTACCAGACTCGAAAGAGCCCGGTATTGTTATTTATTGTCACTACCTCCCCGTGTCAGGATTGGGTAATTTGCGCGCCTGCTGCCTTCCTT	3 (0.000057%)	
TCCAACCTAGGCGAGACAAGGGTTCACATTTCGTTCATCACCCTTGGCCGGCTATCGAACAGCCGGACTCCCATCAAAAGATGGTTGCCAAGAACATCTT	4 (0.000077%)	
TCCAACCTAGGCGAGACAAGGGTTCACATTTCGTTCATCACCCTTGGCCGGCTTTCGAACAGCCGGACTCCCATCAAAAGATGGTTGCCAAGAACATCTT	3 (0.000057%)	
TCCAACGAAGCACGCCCATCCAACCTAGGCGAGACAAGGGTTCACATTTCGTTCATCACCCTTGGCCGGCTATCGAACAGCCGGACTCCCATCAAAAGAT	25 (0.000479%)	
TCCAACGAAGCACGCCCATCCAACCTAGGCGAGACAAGGGTTCACATTTCGTTCATCACCCTTGGCCGGCTTTCGAACAGCCGGACTCCCATCAAAAGAT	18 (0.000345%)	
TCCAATTACCAGACTCGAAAGAGCCCGGTATTGTTATTTATTGTCACTACCTCCCCGTGTCAGGATTGGGTAATTTGCGCGCCTGCTGCCTTCCTTGGAT	7 (0.000134%)	

TCCAGAGCGTAGGCTTGCTTTGAGCACTCTAATTTCTTCAAAGTAACAGCGCCGGAGGCACGACCCGGCCAATTAAGACCAGGAGCGTATCGCCGACCGA	4 (0.000077%)	
TCCATATGAGTCTTTGGCTTTGTGTCTTCTAACAAGGATA	256 (0.001962%)	
TCCATGCTTTCCAACGAAGCACGCCCATCCAACCTAGGCGAGACAAGGGTTCACATTTCGTTCATCACCCTTGGCCGGCTATCGAACAGCCGGACTCCCA	12 (0.000230%)	
TCCATTGGAGGGCAAGTCTGGTGCCAGCAGCCGCGGTAATTCCAGCTCCAATAGCGTATATTTAAGTTGTTGCAGTTAAAAAGCTCGTAGTTGAACCTTG	11 (0.000211%)	
TCCCATGCTAATGTATCCAGAGCGTAGGCTTGCTTTGAGCACTCTAATTTCTTCAAAGTAACAGCGCCGGAGGCACGACCCGGCCAATTAAGACCAGGAG	5 (0.000096%)	
TCCCTTGCCTACATTGTTCCATCGACCAGAGGCTGTTCACCTTGGAGACCTGATGCGGTTATGAGTACGACCGGGCGTGAGCGGCACTCGGTCCTCCGGA	6 (0.000115%)	
TCCGACTTTGTGAAATGACTTGAGAGGTGTAGGATAAGTGGGAGCTTCGGCGCAAGTGAAATACCACTACTTTTAACGTTATTTTACTTACTCCGTGAAT	6 (0.000115%)	
TCCGCCTAGGCTGTCCCGAGTGTGAGCGAGGTGTGAGTGTCGCCCATGGGCATCGACACCTTGCGGCTAGGAACTGGAACGAGACGGGTAGCAAAGATTT	170 (0.003258%)	
TCCGCCTAGGCTGTCCCGAGTGTGAGCGAGGTGTGAGTGTCGCCCATGGGCATCGACACCTTGCGGCTAGGAACTGGAACGAGACGGGTGGCAAAGATTT	283 (0.005424%)	
TCCGCTTATTGATATGCTTAAACTCAGCGGGTAATCCCGCCTGACCTGGGGTCGCTATATGGACTTTGGGTCATCTACAGCTTCCGGACAAGAGCGACCG	5 (0.000096%)	
TCCGGGCGTCGGCCTGTGGGCTCCCCATTCGACCCGTCTTGAAACACGGACCAAGGAGTCTGACATGTGTGCGAGTCAACGGGTGAGTAAACCCGTAAGG	4 (0.000077%)	
TCCGGGGAGGCGAATGCCAGCCGTTCGTTTGCATGTTCCTTGACACTTTTCGTGCCGGGGTTTTGTGATATCCGGAAGCAACGCGCACGACAAGACCGAG	7 (0.000134%)	
TCCGGTTAAAATTCCGGAACCGGGACGTGGCGGTTGACGGCAACGTTAGGGAGTCCGGAGACGTCGGCGGGGGCCTCGGGAAGAGTTATCTTTTCTGTTT	30 (0.000575%)	
TCCGTCGAGTTATCATGAATCATCAGAGCAACGGGCAGAGCCCGCGTCGACCTTTTATCTAATAAATGCGTCCCTTCCATAAGTCGGGGTTTGTTGCACG	17 (0.000326%)	
TCCGTCTCCGGGGAGGCGAATGCCAGCCGTTCGTTTGCATGTTCCTTGACACTTTTCGTGCCGGGGTTTTGTGATATCCGGAAGCAACGCGCACGACAAG	791 (0.015159%)	
TCCTATGATGTTATCCCATGCTAATGTATCCAGAGCGTAGGCTTGCTTTGAGCACTCTAATTTCTTCAAAGTAACAGCGCCGGAGGCACGACCCGGCCAA	7 (0.000134%)	
TCCTCGTTAAGGGATTTAGATTGTACTCATTCCAATTACCAGACTCGAAAGAGCCCGGTATTGTTATTTATTGTCACTACCTCCCCGTGTCAGGATTGGG	25 (0.000479%)	
TCCTTGTTAGAAGACACAAAGCCAAAGACTCATATGGACTTTGGCTACACCATGAAAGCTTTGAGAAGCAAGAAGAAGGTTGGTTAGTGTTTTGGAGTCG	9 (0.000172%)	
TCGAAAGAGCCCGGTATTGTTATTTATTGTCACTACCTCCCCGTGTCAGGATTGGGTAATTTGCGCGCCTGCTGCCTTCCTTGGATGTGGTAGCCGTTTC	8 (0.000153%)	
TCGAAATCCGCTAAGGAGTGTGTAACAACTCACCTGCCGAATCAACTAGCCCCGAAAATGGATGGCGCTTAAGCGCGCGACCTATACCCGGCCGTCGGGG	50 (0.000958%)	
TCGAAATCCTATGATGTTATCCCATGCTAATGTATCCAGAGCGTAGGCTTGCTTTGAGCACTCTAATTTCTTCAAAGTAACAGCGCCGGAGGCACGACCC	257 (0.004925%)	
TCGAATATGACTTGATGTCATGTGTATGATTGAGTATAAG	8791 (0.067390%)	
TCGACCTTTTATCTAATAAATGCGTCCCTTCCATAAGTCGGGGTTTGTTGCACGTATTAGCTCTAGAATTACTACGGTTATCCGAGTAGTAGTTACCATC	12 (0.000230%)	
TCGACTCCAAAACACTAACCAACCTTCTTCTTGCTTCTCAAAGCTTTCATGGTGTAGCCAAAGTCCATATGAGTCTTTGGCTTTGTGTCTTCTAACAAGG	22 (0.000422%)	
TCGAGTTATCATGAATCATCAGAGCAACGGGCAGAGCCCGCGTCGACCTTTTATCTAATAAATGCGTCCCTTCCATAAGTCGGGGTTTGTTGCACGTATT	62 (0.001188%)	
TCGATCAAGTTCACCACCGCATGTCGGTACGCTCCAGGCGTCCTTGGCTCGGATTTAGGCCAACCGCGTGCGGTAACACACGGGAGACCAGCTTCCGTCC	3 (0.000057%)	
TCGATCCATGCTTTCCAACGAAGCACGCCCATCCAACCTAGGCGAGACAAGGGTTCACATTTCGTTCATCACCCTTGGCCGGCTATCGAACAGCCGGACT	9 (0.000172%)	
TCGATCCGTAACTTCGGGAAAAGGATTGGCTCTGAGGGCTGGGCTCGGGGGTCCCAGTTCCGAACCCGTCGGCTGTCAGCGGACTGCTCGAGCTGCTTCC	12 (0.000230%)	
TCGATCCTCGTTAAGGGATTTAGATTGTACTCATTCCAATTACCAGACTCGAAAGAGCCCGGTATTGTTATTTATTGTCACTACCTCCCCGTGTCAGGAT	3 (0.000057%)	
TCGATCGAAATCCTATGATGTTATCCCATGCTAATGTATCCAGAGCGTAGGCTTGCTTTGAGCACTCTAATTTCTTCAAAGTAACAGCGCCGGAGGCACG	6 (0.000115%)	
TCGATCGGGTTGCGGTTTAAGTTGTTATACTCAATCATAC	10 (0.000077%)	
TCGCCCATGGGCATCGACACCTTGCGGCTAGGAACTGGAACGAGACGGGTGGCAAAGATTTCGAGTAGCACTTCATACTACCGTGGGTTTTTTAAACCTT	4 (0.000077%)	
TCGCCGCCCGCGACGTCGCGAGAAGTCCACTAAACCTTATCATTTAGAGGAAGGAGAAGTCGTAACAAGGTTTCCGTAGGTGAACCTGCGGAAGGATCGA	5 (0.000096%)	
TCGCCGTTACTAAGGGAATCCTTGTTAGTTTCTTTTCCTCCGCTTATTGATATGCTTAAACTCAGCGGGTAATCCCGCCTGACCTGGGGTCGCTATATGG	3 (0.000057%)	
TCGGCATCAGCGTGCTCCGGGCGTCGGCCTGTGGGCTCCCCATTCGACCCGTCTTGAAACACGGACCAAGGAGTCTGACATGTGTGCGAGTCAACGGGTG	28 (0.000537%)	
TCGGCCTGTGGGCTCCCCATTCGACCCGTCTTGAAACACGGACCAAGGAGTCTGACATGTGTGCGAGTCAACGGGTGAGTAAACCCGTAAGGCGCAAGGA	7 (0.000134%)	
TCGGGTTGCGGTTTAAGTTGTTATACTCAATCATACACAT	117 (0.000897%)	
TCGGTACGCTCCAGGCGTCCTTGGCTCGGATTTAGGCCAACCGCGTGCGGTAACACACGGGAGACCAGCTTCCGTCCCGCATCAGCAAAGGATGGTGAGG	4 (0.000077%)	
TCGGTAGGAGCGACGGGCGGTGTGTACAAAGGGCAGGGACGTAGTCAACGCGAGCTGATGACTCGCGCTTACTAGGAATTCCTCGTTGAAGACCAACAAT	33 (0.000632%)	
TCGTACTGAAAATCAGAATCAAACGAGCTTTTACCCTTTTGTTCCACACGAGATTTCTGTTCTCGTTGAGCTCATCTTAGGACACCTGCGTTATCTTTTA	5 (0.000096%)	
TCGTATTCGTACTGAAAATCAGAATCAAACGAGCTTTTACCCTTTTGTTCCACACGAGATTTCTGTTCTCGTTGAGCTCATCTTAGGACACCTGCGTTAT	6 (0.000115%)	
TCGTATTTCATAGTCAGAGGTGAAATTCTTGGATTTATGAAAGACGAACAACTGCGAAAGCATTTGCCAAGGATGTTTTCATTAATCAAGAACGAAAGTT	7 (0.000134%)	
TCGTCCCTCACCATCCTTTGCTGATGCGGGACGGAAGCTGGTCTCCCGTGTGTTACCGCACGCGGTTGGCCTAAATCCGAGCCAAGGACGCCTGGAGCGT	5 (0.000096%)	

TCGTTAAGGGATTTAGATTGTACTCATTCCAATTACCAGACTCGAAAGAGCCCGGTATTGTTATTTATTGTCACTACCTCCCCGTGTCAGGATTGGGTAA	8 (0.000153%)	
TCTAGTCCGACTTTGTGAAATGACTTGAGAGGTGTAGGATAAGTGGGAGCTTCGGCGCAAGTGAAATACCACTACTTTTAACGTTATTTTACTTACTCCG	19 (0.000364%)	
TCTCAAAGCTTTCATGGTGTAGCCAAAGTCCATATGAGTCTTTGGCTTTGTGTCTTCTAACAAGGAAACACTACTTAGGCTTATAAGATGCGGTTGCGGT	6 (0.000115%)	
TCTCTCTCTCTCTCTCTCTCTCTCTCTCTCTCTCTCTC	66 (0.000506%)	
TCTGGGCCGCACGCGCGCTACACTGATGTATTCAACGAGTTCACACCTTGGCCGACAGGCCCGGGTAATCTTTGAAATTTCATCGTGATGGGGATAGATC	3 (0.000057%)	
TCTGGTGCCAGCAGCCGCGGTAATTCCAGCTCCAATAGCGTATATTTAAGTTGTTGCAGTTAAAAAGCTCGTAGTTGAACCTTGGGATGGGTCGGCCGGT	14 (0.000268%)	
TCTGTGATGCCCTTAGATGTTCTGGGCCGCACGCGCGCTACACTGATGTATTCAACGAGTTCACACCTTGGCCGACAGGCCCGGGTAATCTTTGAAATTT	10 (0.000192%)	
TCTTAAAAGCCTAAGTAGTGTTTCCTTGTTAGAAGACACAAAGCCAAAGACTCATATGGACTTTGGCTACACCATGAAAGCTTTGAGAAGCAAGAAGAAG	24 (0.000460%)	
TCTTAAAGGCGTAAGAATTGTATCCTTGTTAAAAGACACAAAGCCAAAGACTCATATGGACTTTGGCTACACCATGAAAGCTTTGAGAAGCAAGAAGAAG	86 (0.001648%)	
TCTTAAAGGCGTAAGAATTGTATCCTTGTTAGAAGACACAAAGCCAAAGACTCATATGGACTTTGGCTACACCATGAAAGCTTTGAGAAGCAAGAAGAAG	88 (0.001686%)	
TCTTATACTCAATCATACACATGACATCAAGTCATATTCGACTCCAAAACACTAACCAACCTTCTTCTTGCTTCTCAAAGCTTTCATGGTGTAGCCAAAG	221 (0.004235%)	
TCTTCAACGAGGAATTCCTAGTAAGCGCGAGTCATCAGCTCGCGTTGACTACGTCCCTGCCCTTTGTACACACCGCCCGTCGCTCCTACCGATTGAATGA	12 (0.000230%)	
TCTTTTCCTCCGCTTATTGATATGCTTAAACTCAGCGGGTAATCCCGCCTGACCTGGGGTCGCTATATGGACTTTGGGTCATCTACAGCTTCCGGACAAG	4 (0.000077%)	
TGAAAATCAGAATCAAACGAGCTTTTACCCTTTTGTTCCACACGAGATTTCTGTTCTCGTTGAGCTCATCTTAGGACACCTGCGTTATCTTTTAACAGAT	46 (0.000882%)	
TGAATCATCAGAGCAACGGGCAGAGCCCGCGTCGACCTTTTATCTAATAAATGCGTCCCTTCCATAAGTCGGGGTTTGTTGCACGTATTAGCTCTAGAAT	10 (0.000192%)	
TGACATCAAGTCATATTCGACTCCAAAACACTAACCAACCTTCTTCTTGCTTCTCAAAGCTTTCATGGTGTAGCCAAAGTCCATATGAGTCTTTGGCTTT	31 (0.000594%)	
TGACTCTAGTCCGACTTTGTGAAATGACTTGAGAGGTGTAGGATAAGTGGGAGCTTCGGCGCAAGTGAAATACCACTACTTTTAACGTTATTTTACTTAC	11 (0.000211%)	
TGACTTGATGTCATGTGTATGATTGAGTATAAGAACTTAA	87 (0.000667%)	
TGAGCGAGGTGTGAGTGTCGCCCATGGGCATCGACACCTTGCGGCTAGGAACTGGAACGAGACGGGTAGCAAAGATTTCGAGTAGCACTTCATACTACCG	3 (0.000057%)	
TGAGCGAGGTGTGAGTGTCGCCCATGGGCATCGACACCTTGCGGCTAGGAACTGGAACGAGACGGGTGGCAAAGATTTCGAGTAGCACTTCATACTACCG	4 (0.000077%)	
TGAGCTTGACTCTAGTCCGACTTTGTGAAATGACTTGAGAGGTGTAGGATAAGTGGGAGCTTCGGCGCAAGTGAAATACCACTACTTTTAACGTTATTTT	9 (0.000172%)	
TGAGTGTCGCCCATGGGCATCGACACCTTGCGGCTAGGAACTGGAACGAGACGGGTGGCAAAGATTTCGAGTAGCACTTCATACTACCGTGGGTTTTTTA	7 (0.000134%)	
TGATGCCCTTAGATGTTCTGGGCCGCACGCGCGCTACACTGATGTATTCAACGAGTTCACACCTTGGCCGACAGGCCCGGGTAATCTTTGAAATTTCATC	11 (0.000211%)	
TGATGCGGGACGGAAGCTGGTCTCCCGTGTGTTACCGCACGCGGTTGGCCTAAATCCGAGCCAAGGACGCCTGGAGCGTACCGACATGCGGTGGTGAACT	6 (0.000115%)	
TGATGTCATGTGTATGATTGAGTATAAGAACTTAAACCGC	104 (0.000797%)	
TGATGTTATCCCATGCTAATGTATCCAGAGCGTAGGCTTGCTTTGAGCACTCTAATTTCTTCAAAGTAACAGCGCCGGAGGCACGACCCGGCCAATTAAG	31 (0.000594%)	
TGATTAACAGGGACAGTCGGGGGCATTCGTATTTCATAGTCAGAGGTGAAATTCTTGGATTTATGAAAGACGAACAACTGCGAAAGCATTTGCCAAGGAT	112 (0.002146%)	
TGCAGCACGCGCCTAACGGCGTGCCTCGGCATCAGCGTGCTCCGGGCGTCGGCCTGTGGGCTCCCCATTCGACCCGTCTTGAAACACGGACCAAGGAGTC	88 (0.001686%)	
TGCATGTGTAAGTATGAACGAATTCAGACTGTGAAACTGCGAATGGCTCATTAAATCAGTTATAGTTTGTTTGATGGTAACTACTACTCGGATAACCGTA	11 (0.000211%)	
TGCCAGCAGCCGCGGTAATTCCAGCTCCAATAGCGTATATTTAAGTTGTTGCAGTTAAAAAGCTCGTAGTTGAACCTTGGGATGGGTCGGCCGGTCCGCC	4 (0.000077%)	
TGCCAGTAGTCATATGCTTGTCTCAAAGATTAAGCCATGCATGTGTAAGTATGAACGAATTCAGACTGTGAAACTGCGAATGGCTCATTAAATCAGTTAT	125 (0.002396%)	
TGCCCTTAGATGTTCTGGGCCGCACGCGCGCTACACTGATGTATTCAACGAGTTCACACCTTGGCCGACAGGCCCGGGTAATCTTTGAAATTTCATCGTG	5 (0.000096%)	
TGCCGACTTCCCTTGCCTACATTGTTCCATCGACCAGAGGCTGTTCACCTTGGAGACCTGATGCGGTTATGAGTACGACCGGGCGTGAGCGGCACTCGGT	135 (0.002587%)	
TGCCTACATTGTTCCATCGACCAGAGGCTGTTCACCTTGGAGACCTGATGCGGTTATGAGTACGACCGGGCGTGAGCGGCACTCGGTCCTCCGGATTTTC	9 (0.000172%)	
TGCGAATGGCTCATTAAATCAGTTATAGTTTGTTTGATGGTAACTACTACTCGGATAACCGTAGTAATTCTAGAGCTAATACGTGCAACAAACCCCGACT	8 (0.000153%)	
TGCGGTTTAAGTTCTTATACTCAATCATACACATGACATC	3708 (0.028425%)	
TGCTAATGTATCCAGAGCGTAGGCTTGCTTTGAGCACTCTAATTTCTTCAAAGTAACAGCGCCGGAGGCACGACCCGGCCAATTAAGACCAGGAGCGTAT	21 (0.000402%)	
TGCTGATGCGGGACGGAAGCTGGTCTCCCGTGTGTTACCGCACGCGGTTGGCCTAAATCCGAGCCAAGGACGCCTGGAGCGTACCGACATGCGGTGGTGA	9 (0.000172%)	
TGCTTCTCAAAGCTTTCATGGTGTAGCCAAAGTCCATATGAGTCTTTGGCTTTGTGTCTTCTAACAAGGAAACACTACTTAGGCTTATAAGATGCGGTTG	6 (0.000115%)	
TGCTTGTCTCAAAGATTAAGCCATGCATGTGTAAGTATGAACGAATTCAGACTGTGAAACTGCGAATGGCTCATTAAATCAGTTATAGTTTGTTTGATGG	7 (0.000134%)	
TGCTTTCCAACGAAGCACGCCCATCCAACCTAGGCGAGACAAGGGTTCACATTTCGTTCATCACCCTTGGCCGGCTATCGAACAGCCGGACTCCCATCAA	26 (0.000498%)	
TGGAAAGAGAGAAAGGACGAGGTCTTGACCGTCATCTTTTGCCCGAAGGACGGATGAGCTTTGGCGGGACTGAATCACTTCGAGTCACCGTCGACAACTT	10 (0.000192%)	
TGGAAGTCGAAATCCGCTAAGGAGTGTGTAACAACTCACCTGCCGAATCAACTAGCCCCGAAAATGGATGGCGCTTAAGCGCGCGACCTATACCCGGCCG	957 (0.018340%)	

TGGACTTTGGCTACACCATGAAAGCTTTGAGAAGCAAGAAGAAGGTTGGTTAGTGTTTTGGAGTCGAATATGACTTGATGTCATGTGTATGATTGAGTAT	41 (0.000786%)	
TGGAGGGCAAGTCTGGTGCCAGCAGCCGCGGTAATTCCAGCTCCAATAGCGTATATTTAAGTTGTTGCAGTTAAAAAGCTCGTAGTTGAACCTTGGGATG	322 (0.006171%)	
TGGCTGGGGCGGCACATCTGTTAAAAGATAACGCAGGTGTCCTAAGATGAGCTCAACGAGAACAGAAATCTCGTGTGGAACAAAAGGGTAAAAGCTCGTT	3 (0.000057%)	
TGGGCATCGACACCTTGCGGCTAGGAACTGGAACGAGACGGGTGGCAAAGATTTCGAGTAGCACTTCATACTACCGTGGGTTTTTTAAACCTTCCGAGTT	28 (0.000537%)	
TGGGCCGCACGCGCGCTACACTGATGTATTCAACGAGTTCACACCTTGGCCGACAGGCCCGGGTAATCTTTGAAATTTCATCGTGATGGGGATAGATCGA	14 (0.000268%)	
TGGGGCGGCACATCTGTTAAAAGATAACGCAGGTGTCCTAAGATGAGCTCAACGAGAACAGAAATCTCGTGTGGAACAAAAGGGTAAAAGCTCGTTTGAT	12 (0.000230%)	
TGGGTGGTTCGCCGCCCGCGACGTCGCGAGAAGTCCACTAAACCTTATCATTTAGAGGAAGGAGAAGTCGTAACAAGGTTTCCGTAGGTGAACCTGCGGA	10 (0.000192%)	
TGGTCTTCAACGAGGAATTCCTAGTAAGCGCGAGTCATCAGCTCGCGTTGACTACGTCCCTGCCCTTTGTACACACCGCCCGTCGCTCCTACCGATTGAA	65 (0.001246%)	
TGGTGCCAGCAGCCGCGGTAATTCCAGCTCCAATAGCGTATATTTAAGTTGTTGCAGTTAAAAAGCTCGTAGTTGAACCTTGGGATGGGTCGGCCGGTCC	5 (0.000096%)	
TGGTTCGCCGCCCGCGACGTCGCGAGAAGTCCACTAAACCTTATCATTTAGAGGAAGGAGAAGTCGTAACAAGGTTTCCGTAGGTGAACCTGCGGAAGGA	6 (0.000115%)	
TGTACTCATTCCAATTACCAGACTCGAAAGAGCCCGGTATTGTTATTTATTGTCACTACCTCCCCGTGTCAGGATTGGGTAATTTGCGCGCCTGCTGCCT	5 (0.000096%)	
TGTAGCCAAAGTCCATATGAGTCTTTGGCTTTGTGTCTTT	6689 (0.051276%)	
TGTATCCAGAGCGTAGGCTTGCTTTGAGCACTCTAATTTCTTCAAAGTAACAGCGCCGGAGGCACGACCCGGCCAATTAAGACCAGGAGCGTATCGCCGA	17 (0.000326%)	
TGTATCCTTGTTAGAAGACACAAAGCCAAAGACTCATATGGACTTTGGCTACACCATGAAAGCTTTGAGAAGCAAGAAGAAGGTTGGTTAGTGTTTTGGA	28 (0.000537%)	
TGTCAGGTGGGGAGTTTGGCTGGGGCGGCACATCTGTTAAAAGATAACGCAGGTGTCCTAAGATGAGCTCAACGAGAACAGAAATCTCGTGTGGAACAAA	42 (0.000805%)	
TGTCATGTGTATGATTGAGTATAAGAACTTAAACCGCAAC	635 (0.004868%)	
TGTCCCGAGTGTGAGCGAGGTGTGAGTGTCGCCCATGGGCATCGACACCTTGCGGCTAGGAACTGGAACGAGACGGGTAGCAAAGATTTCGAGTAGCACT	5 (0.000096%)	
TGTCCCGAGTGTGAGCGAGGTGTGAGTGTCGCCCATGGGCATCGACACCTTGCGGCTAGGAACTGGAACGAGACGGGTGGCAAAGATTTCGAGTAGCACT	4 (0.000077%)	
TGTCGCCCATGGGCATCGACACCTTGCGGCTAGGAACTGGAACGAGACGGGTGGCAAAGATTTCGAGTAGCACTTCATACTACCGTGGGTTTTTTAAACC	4 (0.000077%)	
TGTCGGTACGCTCCAGGCGTCCTTGGCTCGGATTTAGGCCAACCGCGTGCGGTAACACACGGGAGACCAGCTTCCGTCCCGCATCAGCAAAGGATGGTGA	3 (0.000057%)	
TGTGAGCGAGGTGTGAGTGTCGCCCATGGGCATCGACACCTTGCGGCTAGGAACTGGAACGAGACGGGTGGCAAAGATTTCGAGTAGCACTTCATACTAC	3 (0.000057%)	
TGTGAGTGTCGCCCATGGGCATCGACACCTTGCGGCTAGGAACTGGAACGAGACGGGTAGCAAAGATTTCGAGTAGCACTTCATACTACCGTGGGTTTTT	9 (0.000172%)	
TGTGAGTGTCGCCCATGGGCATCGACACCTTGCGGCTAGGAACTGGAACGAGACGGGTGGCAAAGATTTCGAGTAGCACTTCATACTACCGTGGGTTTTT	12 (0.000230%)	
TGTGATGCCCTTAGATGTTCTGGGCCGCACGCGCGCTACACTGATGTATTCAACGAGTTCACACCTTGGCCGACAGGCCCGGGTAATCTTTGAAATTTCA	6 (0.000115%)	
TGTTAGAAGACACAAAGCCAAAGACTCATATGGACTTTGGCTACACCATGAAAGCTTTGAGAAGCAAGAAGAAGGTTGGTTAGTGTTTTGGAGTCGAATA	27 (0.000517%)	
TGTTAGTTTCTTTTCCTCCGCTTATTGATATGCTTAAACTCAGCGGGTAATCCCGCCTGACCTGGGGTCGCTATATGGACTTTGGGTCATCTACAGCTTC	7 (0.000134%)	
TGTTATCCCATGCTAATGTATCCAGAGCGTAGGCTTGCTTTGAGCACTCTAATTTCTTCAAAGTAACAGCGCCGGAGGCACGACCCGGCCAATTAAGACC	9 (0.000172%)	
TGTTGAGCTTGACTCTAGTCCGACTTTGTGAAATGACTTGAGAGGTGTAGGATAAGTGGGAGCTTCGGCGCAAGTGAAATACCACTACTTTTAACGTTAT	3 (0.000057%)	
TGTTGGTCTTCAACGAGGAATTCCTAGTAAGCGCGAGTCATCAGCTCGCGTTGACTACGTCCCTGCCCTTTGTACACACCGCCCGTCGCTCCTACCGATT	184 (0.003526%)	
TGTTTCCTTGTTAGAAGACACAAAGCCAAAGACTCATATGGACTTTGGCTACACCATGAAAGCTTTGAGAAGCAAGAAGAAGGTTGGTTAGTGTTTTGGA	24 (0.000460%)	
TTAAAAGATAACGCAGGTGTCCTAAGATGAGCTCAACGAGAACAGAAATCTCGTGTGGAACAAAAGGGTAAAAGCTCGTTTGATTCTGATTTTCAGTACG	25 (0.000479%)	
TTAAAAGCCTAAGTAGTGTTTCCTTGTTAGAAGACACAAAGCCAAAGACTCATATGGACTTTGGCTACACCATGAAAGCTTTGAGAAGCAAGAAGAAGGT	147 (0.002817%)	
TTAAAGGCGTAAGAATTGTATCCTTGTTAAAAGACACAAAGCCAAAGACTCATATGGACTTTGGCTACACCATGAAAGCTTTGAGAAGCAAGAAGAAGGT	126 (0.002415%)	
TTAAAGGCGTAAGAATTGTATCCTTGTTAGAAGACACAAAGCCAAAGACTCATATGGACTTTGGCTACACCATGAAAGCTTTGAGAAGCAAGAAGAAGGT	102 (0.001955%)	
TTAAATCAGTTATAGTTTGTTTGATGGTAACTACTACTCGGATAACCGTAGTAATTCTAGAGCTAATACGTGCAACAAACCCCGACTTATGGAAGGGACG	4 (0.000077%)	
TTAACAGGGACAGTCGGGGGCATTCGTATTTCATAGTCAGAGGTGAAATTCTTGGATTTATGAAAGACGAACAACTGCGAAAGCATTTGCCAAGGATGTT	202 (0.003871%)	
TTAAGCCATGCATGTGTAAGTATGAACGAATTCAGACTGTGAAACTGCGAATGGCTCATTAAATCAGTTATAGTTTGTTTGATGGTAACTACTACTCGGA	15 (0.000287%)	
TTAAGCGCGCGACCTATACCCGGCCGTCGGGGCAAGAGCCAGGCCTCGATGAGTAGGAGGGCGCGGCGGTCGCTGCAAAACCTAGGGCGCGAGCCCGGGC	13 (0.000249%)	
TTAAGGGATTTAGATTGTACTCATTCCAATTACCAGACTCGAAAGAGCCCGGTATTGTTATTTATTGTCACTACCTCCCCGTGTCAGGATTGGGTAATTT	19 (0.000364%)	
TTAAGTTCTTATACTCAATCATACACATGACATCAAGTCATATTCGACTCCAAAACACTAACCAACCTTCTTCTTGCTTCTCAAAGCTTTCATGGTGTAG	2935 (0.056248%)	
TTAAGTTGTTATACTCAATCATACACATGACAACAAGTCATATTCGACTCCAAAACACTAACCAACCTTCTTCTTGCTTCTCAAAGCTTTCATGGTGTAG	70 (0.001342%)	
TTAAGTTGTTATACTCAATCATACACATGACATCAAGTCA	474 (0.003634%)	
TTACCAGACTCGAAAGAGCCCGGTATTGTTATTTATTGTCACTACCTCCCCGTGTCAGGATTGGGTAATTTGCGCGCCTGCTGCCTTCCTTGGATGTGGT	30 (0.000575%)	

TTACGGGTTTACTCACCCGTTGACTCGCACACATGTCAGACTCCTTGGTCCGTGTTTCAAGACGGGTCGAATGGGGAGCCCACAGGCCGACGCCCGGAGC	3313 (0.063492%)	
TTACTCACCCGTTGACTCGCACACATGTCAGACTCCTTGGTCCGTGTTTCAAGACGGGTCGAATGGGGAGCCCACAGGCCGACGCCCGGAGCACGCTGAT	11 (0.000211%)	
TTAGAAGACACAAAGCCAAAGACTCATATGGACTTTGGCTACACCATGAAAGCTTTGAGAAGCAAGAAGAAGGTTGGTTAGTGTTTTGGAGTCGAATATG	83 (0.001591%)	
TTAGATGTTCTGGGCCGCACGCGCGCTACACTGATGTATTCAACGAGTTCACACCTTGGCCGACAGGCCCGGGTAATCTTTGAAATTTCATCGTGATGGG	15 (0.000287%)	
TTAGATTGTACTCATTCCAATTACCAGACTCGAAAGAGCCCGGTATTGTTATTTATTGTCACTACCTCCCCGTGTCAGGATTGGGTAATTTGCGCGCCTG	26 (0.000498%)	
TTAGTTTCTTTTCCTCCGCTTATTGATATGCTTAAACTCAGCGGGTAATCCCGCCTGACCTGGGGTCGCTATATGGACTTTGGGTCATCTACAGCTTCCG	4 (0.000077%)	
TTATACTCAATCATACACATGACAACAAGTCATATTCGACTCCAAAACACTAACCAACCTTCTTCTTGCTTCTCAAAGCTTTCATGGTGTAGCCAAAGTC	239 (0.004580%)	
TTATACTCAATCATACACATGACATCAAGTCATATTCGACTCCAAAACACTAACCAACCTTCTTCTTGCTTCTCAAAGCTTTCATGGTGTAGCCAAAGTC	774 (0.014833%)	
TTATAGTTTGTTTGATGGTAACTACTACTCGGATAACCGTAGTAATTCTAGAGCTAATACGTGCAACAAACCCCGACTTATGGAAGGGACGCATTTATTA	6 (0.000115%)	
TTATCATGAATCATCAGAGCAACGGGCAGAGCCCGCGTCGACCTTTTATCTAATAAATGCGTCCCTTCCATAAGTCGGGGTTTGTTGCACGTATTAGCTC	66 (0.001265%)	
TTATCCCATGCTAATGTATCCAGAGCGTAGGCTTGCTTTGAGCACTCTAATTTCTTCAAAGTAACAGCGCCGGAGGCACGACCCGGCCAATTAAGACCAG	5 (0.000096%)	
TTATCTAATAAATGCGTCCCTTCCATAAGTCGGGGTTTGTTGCACGTATTAGCTCTAGAATTACTACGGTTATCCGAGTAGTAGTTACCATCAAACAAAC	4 (0.000077%)	
TTATTGATATGCTTAAACTCAGCGGGTAATCCCGCCTGACCTGGGGTCGCTATATGGACTTTGGGTCATCTACAGCTTCCGGACAAGAGCGACCGATAAA	16 (0.000307%)	
TTCAACGAGGAATTCCTAGTAAGCGCGAGTCATCAGCTCGCGTTGACTACGTCCCTGCCCTTTGTACACACCGCCCGTCGCTCCTACCGATTGAATGATC	14 (0.000268%)	
TTCAATCGGTAGGAGCGACGGGCGGTGTGTACAAAGGGCAGGGACGTAGTCAACGCGAGCTGATGACTCGCGCTTACTAGGAATTCCTCGTTGAAGACCA	72 (0.001380%)	
TTCACATTTCGTTCATCACCCTTGGCCGGCTATCGAACAGCCGGACTCCCATCAAAAGATGGTTGCCAAGAACATCTTCGTTACGGTTTGCTAATTCTCG	24 (0.000460%)	
TTCACATTTCGTTCATCACCCTTGGCCGGCTTTCGAACAGCCGGACTCCCATCAAAAGATGGTTGCCAAGAACATCTTCGTTACGGTTTGCTAATTCTCG	25 (0.000479%)	
TTCACCACCGCATGTCGGTACGCTCCAGGCGTCCTTGGCTCGGATTTAGGCCAACCGCGTGCGGTAACACACGGGAGACCAGCTTCCGTCCCGCATCAGC	8 (0.000153%)	
TTCACGGTTCGTATTCGTACTGAAAATCAGAATCAAACGAGCTTTTACCCTTTTGTTCCACACGAGATTTCTGTTCTCGTTGAGCTCATCTTAGGACACC	9 (0.000172%)	
TTCATAATTTTTTGACACCTCTAGCTAGGTCATTTGACCTGATACAACATCGGATTTTCATGGTCTAGTTGGGGCTCCGTGGGCATATTTGATGCAAACT	3 (0.000057%)	
TTCATAGTCAGAGGTGAAATTCTTGGATTTATGAAAGACGAACAACTGCGAAAGCATTTGCCAAGGATGTTTTCATTAATCAAGAACGAAAGTTGGGGGC	33 (0.000632%)	
TTCCAACGAAGCACGCCCATCCAACCTAGGCGAGACAAGGGTTCACATTTCGTTCATCACCCTTGGCCGGCTATCGAACAGCCGGACTCCCATCAAAAGA	110 (0.002108%)	
TTCCAACGAAGCACGCCCATCCAACCTAGGCGAGACAAGGGTTCACATTTCGTTCATCACCCTTGGCCGGCTTTCGAACAGCCGGACTCCCATCAAAAGA	2303 (0.044136%)	
TTCCAATTACCAGACTCGAAAGAGCCCGGTATTGTTATTTATTGTCACTACCTCCCCGTGTCAGGATTGGGTAATTTGCGCGCCTGCTGCCTTCCTTGGA	10 (0.000192%)	
TTCCCCGCCACATCCTCTCAAACGCAATGGAAAGAGAGAAAGGACGAGGTCTTGACCGTCATCTTTTGCCCGAAGGACGGATGAGCTTTGGCGGGACTGA	8 (0.000153%)	
TTCCCTTGCCTACATTGTTCCATCGACCAGAGGCTGTTCACCTTGGAGACCTGATGCGGTTATGAGTACGACCGGGCGTGAGCGGCACTCGGTCCTCCGG	10 (0.000192%)	
TTCCTTGTTAGAAGACACAAAGCCAAAGACTCATATGGACTTTGGCTACACCATGAAAGCTTTGAGAAGCAAGAAGAAGGTTGGTTAGTGTTTTGGAGTC	55 (0.001054%)	
TTCGACTCCAAAACACTAACCAACCTTCTTCTTGCTTCTCAAAGCTTTCATGGTGTAGCCAAAGTCCATATGAGTCTTTGGCTTTGTGTCTTCTAACAAG	116 (0.002223%)	
TTCGTACTGAAAATCAGAATCAAACGAGCTTTTACCCTTTTGTTCCACACGAGATTTCTGTTCTCGTTGAGCTCATCTTAGGACACCTGCGTTATCTTTT	24 (0.000460%)	
TTCGTATTCGTACTGAAAATCAGAATCAAACGAGCTTTTACCCTTTTGTTCCACACGAGATTTCTGTTCTCGTTGAGCTCATCTTAGGACACCTGCGTTA	16 (0.000307%)	
TTCGTATTTCATAGTCAGAGGTGAAATTCTTGGATTTATGAAAGACGAACAACTGCGAAAGCATTTGCCAAGGATGTTTTCATTAATCAAGAACGAAAGT	18 (0.000345%)	
TTCTCAAAGCTTTCATGGTGTAGCCAAAGTCCATATGAGTCTTTGGCTTTGTGTCTTCTAACAAGGAAACACTACTTAGGCTTATAAGATGCGGTTGCGG	11 (0.000211%)	
TTCTGGGCCGCACGCGCGCTACACTGATGTATTCAACGAGTTCACACCTTGGCCGACAGGCCCGGGTAATCTTTGAAATTTCATCGTGATGGGGATAGAT	3 (0.000057%)	
TTCTTATACTCAATCATACACATGACATCAAGTCATATTCGACTCCAAAACACTAACCAACCTTCTTCTTGCTTCTCAAAGCTTTCATGGTGTAGCCAAA	105 (0.002012%)	
TTCTTGCTTCTCAAAGCTTTCATGGTGTAGCCAAAGTCCATATGAGTCTTTGGCTTTGTGTCTTCTAACAAGGAAACACTACTTAGGCTTATAAGATGCG	271 (0.005194%)	
TTCTTTTCCTCCGCTTATTGATATGCTTAAACTCAGCGGGTAATCCCGCCTGACCTGGGGTCGCTATATGGACTTTGGGTCATCTACAGCTTCCGGACAA	38 (0.000728%)	
TTGACTCTAGTCCGACTTTGTGAAATGACTTGAGAGGTGTAGGATAAGTGGGAGCTTCGGCGCAAGTGAAATACCACTACTTTTAACGTTATTTTACTTA	16 (0.000307%)	
TTGAGCTTGACTCTAGTCCGACTTTGTGAAATGACTTGAGAGGTGTAGGATAAGTGGGAGCTTCGGCGCAAGTGAAATACCACTACTTTTAACGTTATTT	30 (0.000575%)	
TTGATCGATC	180377 (0.345683%)	
TTGATGGTAACTACTACTCGGATAACCGTAGTAATTCTAGAGCTAATACGTGCAACAAACCCCGACTTATGGAAGGGACGCATTTATTAGATAAAAGGTC	21 (0.000402%)	
TTGATGTCATGTGTATGATTGAGTATAAGAACTTAAACCG	368 (0.002821%)	
TTGCCGACTTCCCTTGCCTACATTGTTCCATCGACCAGAGGCTGTTCACCTTGGAGACCTGATGCGGTTATGAGTACGACCGGGCGTGAGCGGCACTCGG	125 (0.002396%)	
TTGCCTACATTGTTCCATCGACCAGAGGCTGTTCACCTTGGAGACCTGATGCGGTTATGAGTACGACCGGGCGTGAGCGGCACTCGGTCCTCCGGATTTT	7 (0.000134%)	

TTGCGGTTTAAGTTCTTATACTCAATCATACACATGACAT	2025 (0.015523%)
TTGCGGTTTAAGTTGTTATACTCAATCATACACATGACAT	710 (0.005443%)
TTGCTCCGTTCCGCATCCGACCAGGACGCATCGCCGGCCCCCATCCGCTTCCCTCCCGACAATTTCAAGCACTCTTTGACTCTCTTTTCAAAGTCCTTTT	6 (0.000115%)
TTGCTGATGCGGGACGGAAGCTGGTCTCCCGTGTGTTACCGCACGCGGTTGGCCTAAATCCGAGCCAAGGACGCCTGGAGCGTACCGACATGCGGTGGTG	4 (0.000077%)
TTGCTTCTCAAAGCTTTCATGGTGTAGCCAAAGTCCATATGAGTCTTTGGCTTTGTGTCTTCTAACAAGGAAACACTACTTAGGCTTATAAGATGCGGTT	4 (0.000077%)
TTGCTTTGAGCACTCTAATTTCTTCAAAGTAACAGCGCCGGAGGCACGACCCGGCCAATTAAGACCAGGAGCGTATCGCCGACCGAAGGGACAAGCCGAC	7 (0.000134%)
TTGGAGGGCAAGTCTGGTGCCAGCAGCCGCGGTAATTCCAGCTCCAATAGCGTATATTTAAGTTGTTGCAGTTAAAAAGCTCGTAGTTGAACCTTGGGAT	339 (0.006497%)
TTGGCATGCATCATAAGGATACTAAATCCTATTTTCTGGTAAATTTTCATAATTTTTTGACACCTCTAGCTAGGTCATTTGACCTGATACAACATCGGAT	442 (0.008471%)
TTGGCTGGGGCGGCACATCTGTTAAAAGATAACGCAGGTGTCCTAAGATGAGCTCAACGAGAACAGAAATCTCGTGTGGAACAAAAGGGTAAAAGCTCGT	19 (0.000364%)
TTGGTCTTCAACGAGGAATTCCTAGTAAGCGCGAGTCATCAGCTCGCGTTGACTACGTCCCTGCCCTTTGTACACACCGCCCGTCGCTCCTACCGATTGA	89 (0.001706%)
TTGTACTCATTCCAATTACCAGACTCGAAAGAGCCCGGTATTGTTATTTATTGTCACTACCTCCCCGTGTCAGGATTGGGTAATTTGCGCGCCTGCTGCC	12 (0.000230%)
TTGTATCCTTGTTAGAAGACACAAAGCCAAAGACTCATATGGACTTTGGCTACACCATGAAAGCTTTGAGAAGCAAGAAGAAGGTTGGTTAGTGTTTTGG	20 (0.000383%)
TTGTCAGGTGGGGAGTTTGGCTGGGGCGGCACATCTGTTAAAAGATAACGCAGGTGTCCTAAGATGAGCTCAACGAGAACAGAAATCTCGTGTGGAACAA	3 (0.000057%)
TTGTCTCAAAGATTAAGCCATGCATGTGTAAGTATGAACGAATTCAGACTGTGAAACTGCGAATGGCTCATTAAATCAGTTATAGTTTGTTTGATGGTAA	13 (0.000249%)
TTGTTAGAAGACACAAAGCCAAAGACTCATATGGACTTTGGCTACACCATGAAAGCTTTGAGAAGCAAGAAGAAGGTTGGTTAGTGTTTTGGAGTCGAAT	59 (0.001131%)
TTGTTAGTTTCTTTTCCTCCGCTTATTGATATGCTTAAACTCAGCGGGTAATCCCGCCTGACCTGGGGTCGCTATATGGACTTTGGGTCATCTACAGCTT	12 (0.000230%)
TTGTTGGTCTTCAACGAGGAATTCCTAGTAAGCGCGAGTCATCAGCTCGCGTTGACTACGTCCCTGCCCTTTGTACACACCGCCCGTCGCTCCTACCGAT	195 (0.003737%)
TTGTTTGATGGTAACTACTACTCGGATAACCGTAGTAATTCTAGAGCTAATACGTGCAACAAACCCCGACTTATGGAAGGGACGCATTTATTAGATAAAA	11 (0.000211%)
TTTAAGTTCTTATACTCAATCATACACATGACATCAAGTC	755 (0.005788%)
TTTAAGTTGTTATACTCAATCATACACATGACAACAAGTCATATTCGACTCCAAAACACTAACCAACCTTCTTCTTGCTTCTCAAAGCTTTCATGGTGTA	37 (0.000709%)
TTTAAGTTGTTATACTCAATCATACACATGACATCAAGTC	325 (0.002491%)
TTTACTCACCCGTTGACTCGCACACATGTCAGACTCCTTGGTCCGTGTTTCAAGACGGGTCGAATGGGGAGCCCACAGGCCGACGCCCGGAGCACGCTGA	8 (0.000153%)
TTTAGATTGTACTCATTCCAATTACCAGACTCGAAAGAGCCCGGTATTGTTATTTATTGTCACTACCTCCCCGTGTCAGGATTGGGTAATTTGCGCGCCT	129 (0.002472%)
TTTATCTAATAAATGCGTCCCTTCCATAAGTCGGGGTTTGTTGCACGTATTAGCTCTAGAATTACTACGGTTATCCGAGTAGTAGTTACCATCAAACAAA	4 (0.000077%)
TTTCACGGTTCGTATTCGTACTGAAAATCAGAATCAAACGAGCTTTTACCCTTTTGTTCCACACGAGATTTCTGTTCTCGTTGAGCTCATCTTAGGACAC	66 (0.001265%)
TTTCATAATTTTTTGACACCTCTAGCTAGGTCATTTGACCTGATACAACATCGGATTTTCATGGTCTAGTTGGGGCTCCGTGGGCATATTTGATGCAAAC	10 (0.000192%)
TTTCATAGTCAGAGGTGAAATTCTTGGATTTATGAAAGACGAACAACTGCGAAAGCATTTGCCAAGGATGTTTTCATTAATCAAGAACGAAAGTTGGGGG	9 (0.000172%)
TTTCCAACGAAGCACGCCCATCCAACCTAGGCGAGACAAGGGTTCACATTTCGTTCATCACCCTTGGCCGGCTATCGAACAGCCGGACTCCCATCAAAAG	304 (0.005826%)
TTTCCTCCGCTTATTGATATGCTTAAACTCAGCGGGTAATCCCGCCTGACCTGGGGTCGCTATATGGACTTTGGGTCATCTACAGCTTCCGGACAAGAGC	5 (0.000096%)
TTTCCTTGTTAGAAGACACAAAGCCAAAGACTCATATGGACTTTGGCTACACCATGAAAGCTTTGAGAAGCAAGAAGAAGGTTGGTTAGTGTTTTGGAGT	609 (0.011671%)
TTTCGTTCATCACCCTTGGCCGGCTATCGAACAGCCGGACTCCCATCAAAAGATGGTTGCCAAGAACATCTTCGTTACGGTTTGCTAATTCTCGGAATAA	12 (0.000230%)
TTTCTGGTAAATTTTCATAATTTTTTGACACCTCTAGCTAGGTCATTTGACCTGATACAACATCGGATTTTCATGGTCTAGTTGGGGCTCCGTGGGCATA	14 (0.000268%)
TTTCTTTTCCTCCGCTTATTGATATGCTTAAACTCAGCGGGTAATCCCGCCTGACCTGGGGTCGCTATATGGACTTTGGGTCATCTACAGCTTCCGGACA	12 (0.000230%)
TTTGATGGTAACTACTACTCGGATAACCGTAGTAATTCTAGAGCTAATACGTGCAACAAACCCCGACTTATGGAAGGGACGCATTTATTAGATAAAAGGT	8 (0.000153%)
TTTGCCGACTTCCCTTGCCTACATTGTTCCATCGACCAGAGGCTGTTCACCTTGGAGACCTGATGCGGTTATGAGTACGACCGGGCGTGAGCGGCACTCG	2344 (0.044922%)
TTTGCTGATGCGGGACGGAAGCTGGTCTCCCGTGTGTTACCGCACGCGGTTGGCCTAAATCCGAGCCAAGGACGCCTGGAGCGTACCGACATGCGGTGGT	30 (0.000575%)
TTTGGCTGGGGCGGCACATCTGTTAAAAGATAACGCAGGTGTCCTAAGATGAGCTCAACGAGAACAGAAATCTCGTGTGGAACAAAAGGGTAAAAGCTCG	20 (0.000383%)
TTTGTTTGATGGTAACTACTACTCGGATAACCGTAGTAATTCTAGAGCTAATACGTGCAACAAACCCCGACTTATGGAAGGGACGCATTTATTAGATAAA	16 (0.000307%)
TTTTATCTAATAAATGCGTCCCTTCCATAAGTCGGGGTTTGTTGCACGTATTAGCTCTAGAATTACTACGGTTATCCGAGTAGTAGTTACCATCAAACAA	13 (0.000249%)
TTTTCATAATTTTTTGACACCTCTAGCTAGGTCATTTGACCTGATACAACATCGGATTTTCATGGTCTAGTTGGGGCTCCGTGGGCATATTTGATGCAAA	15 (0.000287%)
TTTTCCTCCGCTTATTGATATGCTTAAACTCAGCGGGTAATCCCGCCTGACCTGGGGTCGCTATATGGACTTTGGGTCATCTACAGCTTCCGGACAAGAG	47 (0.000901%)
TTTTCTGGTAAATTTTCATAATTTTTTGACACCTCTAGCTAGGTCATTTGACCTGATACAACATCGGATTTTCATGGTCTAGTTGGGGCTCCGTGGGCAT	5 (0.000096%)
TTTTTTTTTT	53205 (0.101965%)

After filtering: read2: quality

Value of each position will be shown on mouse over.

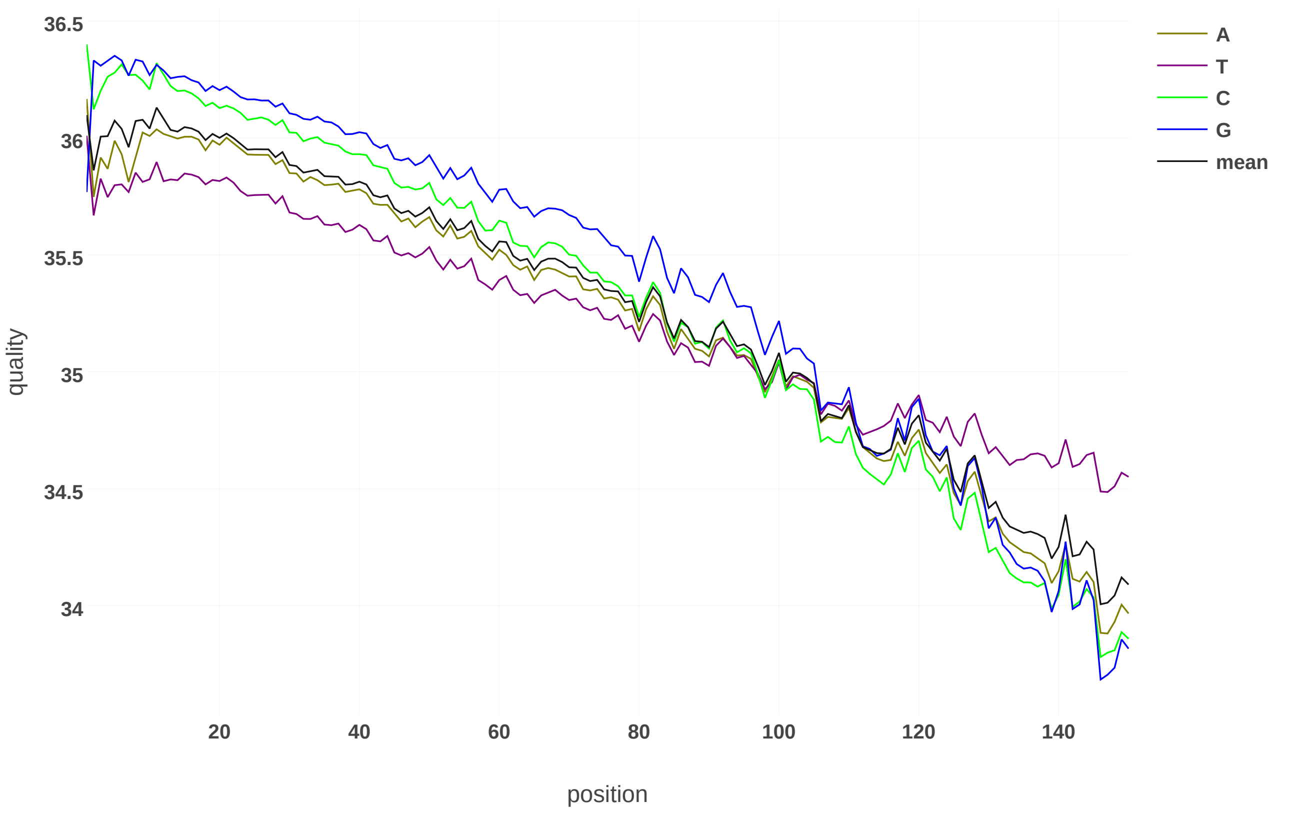

After filtering: read2: base contents

Value of each position will be shown on mouse over.

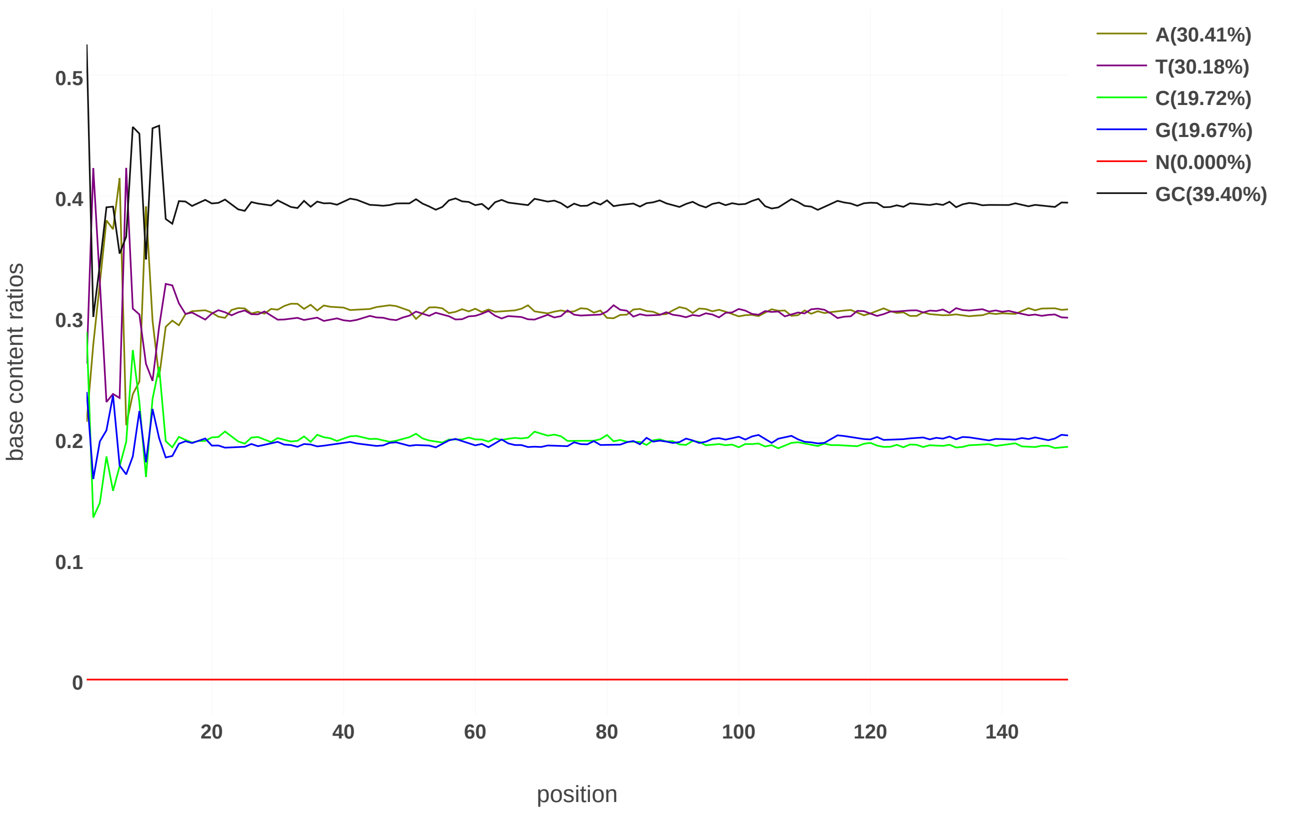

After filtering: read2: KMER counting

Darker background means larger counts. The count will be shown on mouse over.

	AA	AT	AC	AG	TA	TT	TC	TG	CA	CT	CC	CG	GA	GT	GC	GG
AAA	AAAAA	AAAAT	AAAAC	AAAAG	AAATA	AAATT	AAATC	AAATG	AAACA	AAACT	AAACC	AAACG	AAAGA	AAAGT	AAAGC	AAAGG
AAT	AATAA	AATAT	AATAC	AATAG	AATTA	AATTT	AATTC	AATTG	AATCA	AATCT	AATCC	AATCG	AATGA	AATGT	AATGC	AATGG
AAC	AACAA	AACAT	AACAC	AACAG	AACTA	AACTT	AACTC	AACTG	AACCA	AACCT	AACCC	AACCG	AACGA	AACGT	AACGC	AACGG
AAG	AAGAA	AAGAT	AAGAC	AAGAG	AAGTA	AAGTT	AAGTC	AAGTG	AAGCA	AAGCT	AAGCC	AAGCG	AAGGA	AAGGT	AAGGC	AAGGG
ATA	ATAAA	ATAAT	ATAAC	ATAAG	ATATA	ATATT	ATATC	ATATG	ATACA	ATACT	ATACC	ATACG	ATAGA	ATAGT	ATAGC	ATAGG
ATT	ATTAA	ATTAT	ATTAC	ATTAG	ATTTA	ATTTT	ATTTC	ATTTG	ATTCA	ATTCT	ATTCC	ATTCG	ATTGA	ATTGT	ATTGC	ATTGG
ATC	ATCAA	ATCAT	ATCAC	ATCAG	ATCTA	ATCTT	ATCTC	ATCTG	ATCCA	ATCCT	ATCCC	ATCCG	ATCGA	ATCGT	ATCGC	ATCGG
ATG	ATGAA	ATGAT	ATGAC	ATGAG	ATGTA	ATGTT	ATGTC	ATGTG	ATGCA	ATGCT	ATGCC	ATGCG	ATGGA	ATGGT	ATGGC	ATGGG
ACA	ACAAA	ACAAT	ACAAC	ACAAG	ACATA	ACATT	ACATC	ACATG	ACACA	ACACT	ACACC	ACACG	ACAGA	ACAGT	ACAGC	ACAGG
ACT	ACTAA	ACTAT	ACTAC	ACTAG	ACTTA	ACTTT	ACTTC	ACTTG	ACTCA	ACTCT	ACTCC	ACTCG	ACTGA	ACTGT	ACTGC	ACTGG
ACC	ACCAA	ACCAT	ACCAC	ACCAG	ACCTA	ACCTT	ACCTC	ACCTG	ACCCA	ACCCT	ACCCC	ACCCG	ACCGA	ACCGT	ACCGC	ACCGG
ACG	ACGAA	ACGAT	ACGAC	ACGAG	ACGTA	ACGTT	ACGTC	ACGTG	ACGCA	ACGCT	ACGCC	ACGCG	ACGGA	ACGGT	ACGGC	ACGGG
AGA	AGAAA	AGAAT	AGAAC	AGAAG	AGATA	AGATT	AGATC	AGATG	AGACA	AGACT	AGACC	AGACG	AGAGA	AGAGT	AGAGC	AGAGG
AGT	AGTAA	AGTAT	AGTAC	AGTAG	AGTTA	AGTTT	AGTTC	AGTTG	AGTCA	AGTCT	AGTCC	AGTCG	AGTGA	AGTGT	AGTGC	AGTGG
AGC	AGCAA	AGCAT	AGCAC	AGCAG	AGCTA	AGCTT	AGCTC	AGCTG	AGCCA	AGCCT	AGCCC	AGCCG	AGCGA	AGCGT	AGCGC	AGCGG
AGG	AGGAA	AGGAT	AGGAC	AGGAG	AGGTA	AGGTT	AGGTC	AGGTG	AGGCA	AGGCT	AGGCC	AGGCG	AGGGA	AGGGT	AGGGC	AGGGG
TAA	TAAAA	TAAAT	TAAAC	TAAAG	TAATA	TAATT	TAATC	TAATG	TAACA	TAACT	TAACC	TAACG	TAAGA	TAAGT	TAAGC	TAAGG
TAT	TATAA	TATAT	TATAC	TATAG	TATTA	TATTT	TATTC	TATTG	TATCA	TATCT	TATCC	TATCG	TATGA	TATGT	TATGC	TATGG
TAC	TACAA	TACAT	TACAC	TACAG	TACTA	TACTT	TACTC	TACTG	TACCA	TACCT	TACCC	TACCG	TACGA	TACGT	TACGC	TACGG
TAG	TAGAA	TAGAT	TAGAC	TAGAG	TAGTA	TAGTT	TAGTC	TAGTG	TAGCA	TAGCT	TAGCC	TAGCG	TAGGA	TAGGT	TAGGC	TAGGG
TTA	TTAAA	TTAAT	TTAAC	TTAAG	TTATA	TTATT	TTATC	TTATG	TTACA	TTACT	TTACC	TTACG	TTAGA	TTAGT	TTAGC	TTAGG
TTT	TTTAA	TTTAT	TTTAC	TTTAG	TTTTA	TTTTT	TTTTC	TTTTG	TTTCA	TTTCT	TTTCC	TTTCG	TTTGA	TTTGT	TTTGC	TTTGG
TTC	TTCAA	TTCAT	TTCAC	TTCAG	TTCTA	TTCTT	TTCTC	TTCTG	TTCCA	TTCCT	TTCCC	TTCCG	TTCGA	TTCGT	TTCGC	TTCGG
TTG	TTGAA	TTGAT	TTGAC	TTGAG	TTGTA	TTGTT	TTGTC	TTGTG	TTGCA	TTGCT	TTGCC	TTGCG	TTGGA	TTGGT	TTGGC	TTGGG
TCA	TCAAA	TCAAT	TCAAC	TCAAG	TCATA	TCATT	TCATC	TCATG	TCACA	TCACT	TCACC	TCACG	TCAGA	TCAGT	TCAGC	TCAGG
TCT	TCTAA	TCTAT	TCTAC	TCTAG	TCTTA	TCTTT	TCTTC	TCTTG	TCTCA	TCTCT	TCTCC	TCTCG	TCTGA	TCTGT	TCTGC	TCTGG
TCC	TCCAA	TCCAT	TCCAC	TCCAG	TCCTA	TCCTT	TCCTC	TCCTG	TCCCA	TCCCT	TCCCC	TCCCG	TCCGA	TCCGT	TCCGC	TCCGG
TCG	TCGAA	TCGAT	TCGAC	TCGAG	TCGTA	TCGTT	TCGTC	TCGTG	TCGCA	TCGCT	TCGCC	TCGCG	TCGGA	TCGGT	TCGGC	TCGGG
TGA	TGAAA	TGAAT	TGAAC	TGAAG	TGATA	TGATT	TGATC	TGATG	TGACA	TGACT	TGACC	TGACG	TGAGA	TGAGT	TGAGC	TGAGG
TGT	TGTAA	TGTAT	TGTAC	TGTAG	TGTTA	TGTTT	TGTTC	TGTTG	TGTCA	TGTCT	TGTCC	TGTCG	TGTGA	TGTGT	TGTGC	TGTGG
TGC	TGCAA	TGCAT	TGCAC	TGCAG	TGCTA	TGCTT	TGCTC	TGCTG	TGCCA	TGCCT	TGCCC	TGCCG	TGCGA	TGCGT	TGCGC	TGCGG
TGG	TGGAA	TGGAT	TGGAC	TGGAG	TGGTA	TGGTT	TGGTC	TGGTG	TGGCA	TGGCT	TGGCC	TGGCG	TGGGA	TGGGT	TGGGC	TGGGG
CAA	CAAAA	CAAAT	CAAAC	CAAAG	CAATA	CAATT	CAATC	CAATG	CAACA	CAACT	CAACC	CAACG	CAAGA	CAAGT	CAAGC	CAAGG
CAT	CATAA	CATAT	CATAC	CATAG	CATTA	CATTT	CATTC	CATTG	CATCA	CATCT	CATCC	CATCG	CATGA	CATGT	CATGC	CATGG
CAC	CACAA	CACAT	CACAC	CACAG	CACTA	CACTT	CACTC	CACTG	CACCA	CACCT	CACCC	CACCG	CACGA	CACGT	CACGC	CACGG
CAG	CAGAA	CAGAT	CAGAC	CAGAG	CAGTA	CAGTT	CAGTC	CAGTG	CAGCA	CAGCT	CAGCC	CAGCG	CAGGA	CAGGT	CAGGC	CAGGG
CTA	CTAAA	CTAAT	CTAAC	CTAAG	CTATA	CTATT	CTATC	CTATG	CTACA	CTACT	CTACC	CTACG	CTAGA	CTAGT	CTAGC	CTAGG
CTT	CTTAA	CTTAT	CTTAC	CTTAG	CTTTA	CTTTT	CTTTC	CTTTG	CTTCA	CTTCT	CTTCC	CTTCG	CTTGA	CTTGT	CTTGC	CTTGG
CTC	CTCAA	CTCAT	CTCAC	CTCAG	CTCTA	CTCTT	CTCTC	CTCTG	CTCCA	CTCCT	CTCCC	CTCCG	CTCGA	CTCGT	CTCGC	CTCGG
CTG	CTGAA	CTGAT	CTGAC	CTGAG	CTGTA	CTGTT	CTGTC	CTGTG	CTGCA	CTGCT	CTGCC	CTGCG	CTGGA	CTGGT	CTGGC	CTGGG
CCA	CCAAA	CCAAT	CCAAC	CCAAG	CCATA	CCATT	CCATC	CCATG	CCACA	CCACT	CCACC	CCACG	CCAGA	CCAGT	CCAGC	CCAGG
CCT	CCTAA	CCTAT	CCTAC	CCTAG	CCTTA	CCTTT	CCTTC	CCTTG	CCTCA	CCTCT	CCTCC	CCTCG	CCTGA	CCTGT	CCTGC	CCTGG
CCC	CCCAA	CCCAT	CCCAC	CCCAG	CCCTA	CCCTT	CCCTC	CCCTG	CCCCA	CCCCT	CCCCC	CCCCG	CCCGA	CCCGT	CCCGC	CCCGG
CCG	CCGAA	CCGAT	CCGAC	CCGAG	CCGTA	CCGTT	CCGTC	CCGTG	CCGCA	CCGCT	CCGCC	CCGCG	CCGGA	CCGGT	CCGGC	CCGGG
CGA	CGAAA	CGAAT	CGAAC	CGAAG	CGATA	CGATT	CGATC	CGATG	CGACA	CGACT	CGACC	CGACG	CGAGA	CGAGT	CGAGC	CGAGG
CGT	CGTAA	CGTAT	CGTAC	CGTAG	CGTTA	CGTTT	CGTTC	CGTTG	CGTCA	CGTCT	CGTCC	CGTCG	CGTGA	CGTGT	CGTGC	CGTGG
CGC	CGCAA	CGCAT	CGCAC	CGCAG	CGCTA	CGCTT	CGCTC	CGCTG	CGCCA	CGCCT	CGCCC	CGCCG	CGCGA	CGCGT	CGCGC	CGCGG
CGG	CGGAA	CGGAT	CGGAC	CGGAG	CGGTA	CGGTT	CGGTC	CGGTG	CGGCA	CGGCT	CGGCC	CGGCG	CGGGA	CGGGT	CGGGC	CGGGG
GAA	GAAAA	GAAAT	GAAAC	GAAAG	GAATA	GAATT	GAATC	GAATG	GAACA	GAACT	GAACC	GAACG	GAAGA	GAAGT	GAAGC	GAAGG
GAT	GATAA	GATAT	GATAC	GATAG	GATTA	GATTT	GATTC	GATTG	GATCA	GATCT	GATCC	GATCG	GATGA	GATGT	GATGC	GATGG
GAC	GACAA	GACAT	GACAC	GACAG	GACTA	GACTT	GACTC	GACTG	GACCA	GACCT	GACCC	GACCG	GACGA	GACGT	GACGC	GACGG
GAG	GAGAA	GAGAT	GAGAC	GAGAG	GAGTA	GAGTT	GAGTC	GAGTG	GAGCA	GAGCT	GAGCC	GAGCG	GAGGA	GAGGT	GAGGC	GAGGG
GTA	GTAAA	GTAAT	GTAAC	GTAAG	GTATA	GTATT	GTATC	GTATG	GTACA	GTACT	GTACC	GTACG	GTAGA	GTAGT	GTAGC	GTAGG
GTT	GTTAA	GTTAT	GTTAC	GTTAG	GTTTA	GTTTT	GTTTC	GTTTG	GTTCA	GTTCT	GTTCC	GTTCG	GTTGA	GTTGT	GTTGC	GTTGG
GTC	GTCAA	GTCAT	GTCAC	GTCAG	GTCTA	GTCTT	GTCTC	GTCTG	GTCCA	GTCCT	GTCCC	GTCCG	GTCGA	GTCGT	GTCGC	GTCGG
GTG	GTGAA	GTGAT	GTGAC	GTGAG	GTGTA	GTGTT	GTGTC	GTGTG	GTGCA	GTGCT	GTGCC	GTGCG	GTGGA	GTGGT	GTGGC	GTGGG
GCA	GCAAA	GCAAT	GCAAC	GCAAG	GCATA	GCATT	GCATC	GCATG	GCACA	GCACT	GCACC	GCACG	GCAGA	GCAGT	GCAGC	GCAGG
GCT	GCTAA	GCTAT	GCTAC	GCTAG	GCTTA	GCTTT	GCTTC	GCTTG	GCTCA	GCTCT	GCTCC	GCTCG	GCTGA	GCTGT	GCTGC	GCTGG
GCC	GCCAA	GCCAT	GCCAC	GCCAG	GCCTA	GCCTT	GCCTC	GCCTG	GCCCA	GCCCT	GCCCC	GCCCG	GCCGA	GCCGT	GCCGC	GCCGG
GCG	GCGAA	GCGAT	GCGAC	GCGAG	GCGTA	GCGTT	GCGTC	GCGTG	GCGCA	GCGCT	GCGCC	GCGCG	GCGGA	GCGGT	GCGGC	GCGGG
GGA	GGAAA	GGAAT	GGAAC	GGAAG	GGATA	GGATT	GGATC	GGATG	GGACA	GGACT	GGACC	GGACG	GGAGA	GGAGT	GGAGC	GGAGG
GGT	GGTAA	GGTAT	GGTAC	GGTAG	GGTTA	GGTTT	GGTTC	GGTTG	GGTCA	GGTCT	GGTCC	GGTCG	GGTGA	GGTGT	GGTGC	GGTGG
GGC	GGCAA	GGCAT	GGCAC	GGCAG	GGCTA	GGCTT	GGCTC	GGCTG	GGCCA	GGCCT	GGCCC	GGCCG	GGCGA	GGCGT	GGCGC	GGCGG
GGG	GGGAA	GGGAT	GGGAC	GGGAG	GGGTA	GGGTT	GGGTC	GGGTG	GGGCA	GGGCT	GGGCC	GGGCG	GGGGA	GGGGT	GGGGC	GGGGG

After filtering: read2: overrepresented sequences

Sampling rate: 1 / 20

overrepresented sequence	count (% of bases)	distribution: cycle 1 ~ cycle 150
AAAAAAAAAAAAAAAAAAAA	5181 (0.019858%)	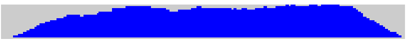
AAAAGATAACGCAGGTGTCCTAAGATGAGCTCAACGAGAACAGAAATCTCGTGTGGAACAAAAGGGTAAAA GCTCGTTTGATTCTGATTTTCAGTACGAA	13 (0.000249%)	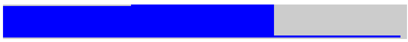
AAAAGTGTCGGAGTTTTTTCAGCAGTTCTCGGACAAAAATTGCTGAGTGGCCGAGAAGAATGGGCGTGTCA TGCGTGGGCTGACATGGATTCTTCGAGGC	3 (0.000057%)	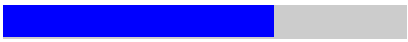
AAAATCATCAATCGTTCCAACTAATCTACCGAAGTACTCGGCTAAGAAGAAAGAAGACGGACGAATCCGAG CCAAAGCCGTACAAGCGCGAGATACCTTC	11 (0.000211%)	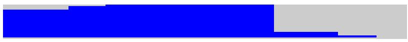
AAACACAGGCCCCGGAACTCATCATCGAGCGTAACATCGCCCGTGAATTAACTGAGAAGGATAGGTGGTAG GTAGTTCGATGCGCGAGCATGGAGCCTAC	20 (0.000383%)	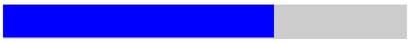
AAACACTTGGTGATATGAACACAAACGTTCAATATGACAAACCCATGCCAAGTAAAGAGAAAATGAAAACT GGTGATTGTTGCGGAAATCGTCCAGGATT	312 (0.005979%)	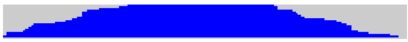
AAACGTTCAATATGACAAACCCATGCCAAGTAAAGAGAAAATGAAAACTGGTGATTGTTGCGGAAATCGTC CAGGATTCCTCGACCAGGACTTGAAATCG	7 (0.000134%)	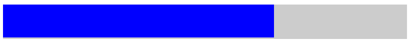
AAAGCCAAAGACTCATATGGACTTTGGCTACACCATGAAAGCTTTGAGAAGCAAGAAGAAGGTTGGTTAGT GTTTTGGAGTCGAATATGACTTGATGTCA	283 (0.005424%)	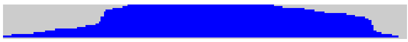
AAAGGCGTAAGAATTGTATCCTTGTTAGAAGACACAAAGC	197 (0.001510%)	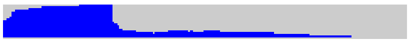
AAAGGGTGTTGGTCGATTAAGACAGCAGGACGGTGGTCATGGAAGTCGAAATCCGCTAAGGAGTGTGTAAC AACTCACCTGCCGAATCAACTAGCCCCGA	10 (0.000192%)	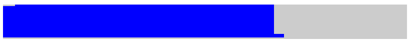
AAAGTGTCGGAGTTTTTTCAGCAGTTCTCGGACAAAAATTGCTGAGTGGCCGAGAAGAATGGGCGTGTCAT GCGTGGGCTGACATGGATTCTTCGAGGCC	3 (0.000057%)	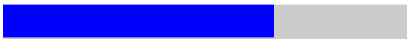
AAAGTTGGGAATTCGTTAAGGAGCTGTTGCTTTGTTAGTGTAGAAACACTTGTGTAGAATTGGGGATTGTT TTTTTTGGAGTGATTTAGGGGAGGGTCGA	10 (0.000192%)	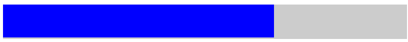
AAATACGGGCGAGAGACCGATAGCGAACAAGTACCGCGAGGTAAAGATGAAAAGGACTTTGAAAAGAGAGT CAAAGAGTGCTTGAAATTGTCGGGAGGGA	5 (0.000096%)	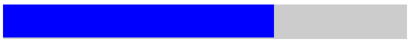
AAATCATCAATCGTTCCAACTAATCTACCGAAGTACTCGGCTAAGAAGAAAGAAGACGGACGAATCCGAGC CAAAGCCGTACAAGCGCGAGATACCTTCG	6 (0.000115%)	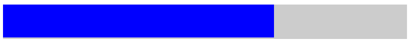
AAATCCTATGATGTTATCCCATGCTAATGTATCCAGAGCGTAGGCTTGCTTTGAGCACTCTAATTTCTTCA AAGTAACAGCGCCGGAGGCACGACCCGGC	21 (0.000402%)	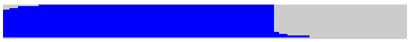
AAATCGTCGTCCCTCACCATCCTTTGCTGATGCGGGACGGAAGCTGGTCTCCCGTGTGTTACCGCACGCGG TTGGCCTAAATCCGAGCCAAGGACGCCTG	40 (0.000767%)	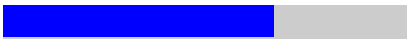
AAATTAGGTACGAAACACAGGCCCCGGAACTCATCATCGAGCGTAACATCGCCCGTGAATTAACTGAGAAG GATAGGTGGTAGGTAGTTCGATGCGCGAG	9 (0.000172%)	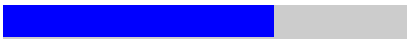
AAATTTCTGCCCTATCAACTTTCGATGGTAGGATAGTGGCCTACCATGGTGGTAACGGGTGACGGAGAATT AGGGTTCGATTCCGGAGAGGGAGCCTGAG	53 (0.001016%)	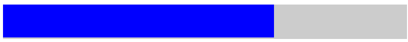
AACACAGGCCCCGGAACTCATCATCGAGCGTAACATCGCCCGTGAATTAACTGAGAAGGATAGGTGGTAGG TAGTTCGATGCGCGAGCATGGAGCCTACG	14 (0.000268%)	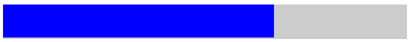

AACACTTGGTGATATGAACACAAACGTTCAATATGACAAACCCATGCCAAGTAAAGAGAAAATGAAAACTGGTGATTGTTGCGGAAATCGTCCAGGATTC	3 (0.000057%)	
AACAGGGACAGTCGGGGGCATTCGTATTTCATAGTCAGAGGTGAAATTCTTGGATTTATGAAAGACGAACAACTGCGAAAGCATTTGCCAAGGATGTTTT	24 (0.000460%)	
AACGAAGCACGCCCATCCAACCTAGGCGAGACAAGGGTTCACATTTCGTTCATCACCCTTGGCCGGCTATCGAACAGCCGGACTCCCATCAAAAGATGGT	18 (0.000345%)	
AACGGCGTGCCTCGGCATCAGCGTGCTCCGGGCGTCGGCCTGTGGGCTCCCCATTCGACCCGTCTTGAAACACGGACCAAGGAGTCTGACATGTGTGCGA	5 (0.000096%)	
AACGGGCAGAGCCCGCGTCGACCTTTTATCTAATAAATGCGTCCCTTCCATAAGTCGGGGTTTGTTGCACGTATTAGCTCTAGAATTACTACGGTTATCC	11 (0.000211%)	
AACTAGCTACGTGGAGGCATCCCTTCACGGCCGGCTTCTTAGAGGGACTATGGCCGTTTAGGCCAAGGAAGTTTGAGGCAATAACAGGTCTGTGATGCCC	6 (0.000115%)	
AACTTTCGATGGTAGGATAGTGGCCTACCATGGTGGTAACGGGTGACGGAGAATTAGGGTTCGATTCCGGAGAGGGAGCCTGAGAAACGGCTACCACATC	5 (0.000096%)	
AAGAATTGTATCCTTGTTAGAAGACACAAAGCCAAAGACTCATATGGACTTTGGCTACACCATGAAAGCTTTGAGAAGCAAGAAGAAGGTTGGTTAGTGT	19 (0.000364%)	
AAGACACAAAGCCAAAGACTCATATGGACTTTGGCTACACCATGAAAGCTTTGAGAAGCAAGAAGAAGGTTGGTTAGTGTTTTGGAGTCGAATATGACTT	487 (0.009333%)	
AAGACAGCAGGACGGTGGTCATGGAAGTCGAAATCCGCTAAGGAGTGTGTAACAACTCACCTGCCGAATCAACTAGCCCCGAAAATGGATGGCGCTTAAG	23 (0.000441%)	
AAGATAACGCAGGTGTCCTAAGATGAGCTCAACGAGAACAGAAATCTCGTGTGGAACAAAAGGGTAAAAGCTCGTTTGATTCTGATTTTCAGTACGAATA	5 (0.000096%)	
AAGATCGATC	201342 (0.385864%)	
AAGCACGCCCATCCAACCTAGGCGAGACAAGGGTTCACATTTCGTTCATCACCCTTGGCCGGCTATCGAACAGCCGGACTCCCATCAAAAGATGGTTGCC	6 (0.000115%)	
AAGCCAAAGACTCATATGGACTTTGGCTACACCATGAAAGCTTTGAGAAGCAAGAAGAAGGTTGGTTAGTGTTTTGGAGTCGAATATGACTTGATGTCAT	10 (0.000192%)	
AAGCCTAAGTAGTGTTTCCTTGTTAGAACATACAAAGCCAAAGACTCATACGGACTTTGGCTACACCATGAAAGCTTTGAGAAGCTAGAAGAAGGTTGGT	1075 (0.020602%)	
AAGCCTAAGTAGTGTTTCCTTGTTAGAAGACACAAAGCCAAAGACTCATATGGACTTTGGCTACACCATGAAAGCTTTGAGAAGCAAGAAGAAGGTTGGT	1653 (0.031679%)	
AAGGCGTAAGAATTGTATCCTTGTTAGAAGACACAAAGCC	67 (0.000514%)	
AAGGGTTCACATTTCGTTCATCACCCTTGGCCGGCTTTCGAACAGCCGGACTCCCATCAAAAGATGGTTGCCAAGAACATCTTCGTTACGGTTTGCTAAT	4 (0.000077%)	
AAGGTATCTCGCGCTTGTACGGCTTTGGCTCGGATTCGTCCGTCTTCTTTCTTCTTAGCCGAGTACTTCGGTAGATTAGTTGGAACGATTGATGATTTTG	9 (0.000172%)	
AAGTAGTGTTTCCTTGTTAGAAGACACAAAGCCAAAGACTCATATGGACTTTGGCTACACCATGAAAGCTTTGAGAAGCAAGAAGAAGGTTGGTTAGTGT	23 (0.000441%)	
AAGTCATATTCGACTCCAAAACACTAACCAACCTTCTTCTTGCTTCTCAAAGCTTTCATGGTGTAGCCAAAGTCCATATGAGTCTTTGGCTTTGTGTCTT	122 (0.002338%)	
AAGTCTGGTGCCAGCAGCCGCGGTAATTCCAGCTCCAATAGCGTATATTTAAGTTGTTGCAGTTAAAAAGCTCGTAGTTGAACCTTGGGATGGGTCGGCC	4 (0.000077%)	
AAGTTCTTATACTCAATCATACACATGACATCAAGTCATATTCGACTCCAAAACACTAACCAACCTTCTTCTTGCTTCTCAAAGCTTTCATGGTGTAGCC	32 (0.000613%)	
AAGTTGTTATACTCAATCATACACATGACAACAAGTCATATTCGACTCCAAAACACTAACCAACCTTCTTCTTGCTTCTCAAAGCTTTCATGGTGTAGCC	25 (0.000479%)	
AAGTTGTTATACTCAATCATACACATGACATCAAGTCATA	126 (0.000966%)	
AATACGGGCGAGAGACCGATAGCGAACAAGTACCGCGAGGTAAAGATGAAAAGGACTTTGAAAAGAGAGTCAAAGAGTGCTTGAAATTGTCGGGAGGGAA	8 (0.000153%)	
AATAGCGTATATTTAAGTTGTTGCAGTTAAAAAGCTCGTAGTTGAACCTTGGGATGGGTCGGCCGGTCCGCCTTTGGTGTGCATTGGTCGGCTTGTCCCT	6 (0.000115%)	
AATCAGCTTCCTTGCGCCTTACGGGTTTACTCACCCGTTGACTCGCACACATGTCAGACTCCTTGGTCCGTGTTTCAAGACGGGTCGAATGGGGAGCCCA	174 (0.003335%)	
AATCATACACATGACATCAAGTCATATTCGACTCCAAAACACTAACCAACCTTCTTCTTGCTTCTCAAAGCTTTCATGGTGTAGCCAAAGTCCATATGAG	56 (0.001073%)	
AATCATCAATCGTTCCAACTAATCTACCGAAGTACTCGGCTAAGAAGAAAGAAGACGGACGAATCCGAGCCAAAGCCGTACAAGCGCGAGATACCTTCGG	5 (0.000096%)	
AATCATCAGAGCAACGGGCAGAGCCCGCGTCGACCTTTTATCTAATAAATGCGTCCCTTCCATAAGTCGGGGTTTGTTGCACGTATTAGCTCTAGAATTA	48 (0.000920%)	
AATCCTATGATGTTATCCCATGCTAATGTATCCAGAGCGTAGGCTTGCTTTGAGCACTCTAATTTCTTCAAAGTAACAGCGCCGGAGGCACGACCCGGCC	41 (0.000786%)	
AATCGGTAGGAGCGACGGGCGGTGTGTACAAAGGGCAGGGACGTAGTCAACGCGAGCTGATGACTCGCGCTTACTAGGAATTCCTCGTTGAAGACCAACA	156 (0.002990%)	
AATCGTCGTCCCTCACCATCCTTTGCTGATGCGGGACGGAAGCTGGTCTCCCGTGTGTTACCGCACGCGGTTGGCCTAAATCCGAGCCAAGGACGCCTGG	14 (0.000268%)	
AATCGTTCCAACTAATCTACCGAAGTACTCGGCTAAGAAGAAAGAAGACGGACGAATCCGAGCCAAAGCCGTACAAGCGCGAGATACCTTCGGGACAGCC	17 (0.000326%)	
AATGATTAACAGGGACAGTCGGGGGCATTCGTATTTCATAGTCAGAGGTGAAATTCTTGGATTTATGAAAGACGAACAACTGCGAAAGCATTTGCCAAGG	515 (0.009870%)	
AATGCTTTGTTTTAATTAAACAGTCGGATTCCCCTTGTCCGTACCAGTTCTGAGCTGACTGTTCGACGCCCGGGGAAAGCTCCCGAGAGAGCCGTTCCCA	701 (0.013434%)	
AATTCCAGCTCCAATAGCGTATATTTAAGTTGTTGCAGTTAAAAAGCTCGTAGTTGAACCTTGGGATGGGTCGGCCGGTCCGCCTTTGGTGTGCATTGGT	12 (0.000230%)	
AATTGTATCCTTGTTAGAAGACACAAAGCCAAAGACTCATATGGACTTTGGCTACACCATGAAAGCTTTGAGAAGCAAGAAGAAGGTTGGTTAGTGTTTT	7 (0.000134%)	
AATTGTTGGTCTTCAACGAGGAATTCCTAGTAAGCGCGAGTCATCAGCTCGCGTTGACTACGTCCCTGCCCTTTGTACACACCGCCCGTCGCTCCTACCG	1428 (0.027367%)	
AATTTCTGCCCTATCAACTTTCGATGGTAGGATAGTGGCCTACCATGGTGGTAACGGGTGACGGAGAATTAGGGTTCGATTCCGGAGAGGGAGCCTGAGA	8 (0.000153%)	
ACAAAGCCAAAGACTCATATGGACTTTGGCTACACCATGAAAGCTTTGAGAAGCAAGAAGAAGGTTGGTTAGTGTTTTGGAGTCGAATATGACTTGATGT	161 (0.003085%)	
ACAAAGGGTGTTGGTCGATTAAGACAGCAGGACGGTGGTCATGGAAGTCGAAATCCGCTAAGGAGTGTGTAACAACTCACCTGCCGAATCAACTAGCCCC	13 (0.000249%)	

ACAAATCGTCGTCCCTCACCATCCTTTGCTGATGCGGGACGGAAGCTGGTCTCCCGTGTGTTACCGCACGCGGTTGGCCTAAATCCGAGCCAAGGACGCC	10 (0.000192%)
ACAAGGGTTCACATTTCGTTCATCACCCTTGGCCGGCTATCGAACAGCCGGACTCCCATCAAAAGATGGTTGCCAAGAACATCTTCGTTACGGTTTGCTA	4 (0.000077%)
ACACAAAGCCAAAGACTCATATGGACTTTGGCTACACCATGAAAGCTTTGAGAAGCAAGAAGAAGGTTGGTTAGTGTTTTGGAGTCGAATATGACTTGAT	153 (0.002932%)
ACACAGGCCCCGGAACTCATCATCGAGCGTAACATCGCCCGTGAATTAACTGAGAAGGATAGGTGGTAGGTAGTTCGATGCGCGAGCATGGAGCCTACGA	8 (0.000153%)
ACACATGACATCAAGTCATATTCGACTCCAAAACACTAACCAACCTTCTTCTTGCTTCTCAAAGCTTTCATGGTGTAGCCAAAGTCCATATGAGTCTTTG	22 (0.000422%)
ACAGGGACAGTCGGGGGCATTCGTATTTCATAGTCAGAGGTGAAATTCTTGGATTTATGAAAGACGAACAACTGCGAAAGCATTTGCCAAGGATGTTTTC	3 (0.000057%)
ACATCAAGTCATATTCGACTCCAAAACACTAACCAACCTTCTTCTTGCTTCTCAAAGCTTTCATGGTGTAGCCAAAGTCCATATGAGTCTTTGGCTTTGT	40 (0.000767%)
ACATGACATCAAGTCATATTCGACTCCAAAACACTAACCAACCTTCTTCTTGCTTCTCAAAGCTTTCATGGTGTAGCCAAAGTCCATATGAGTCTTTGGC	57 (0.001092%)
ACATTGTCAGGTGGGGAGTTTGGCTGGGGCGGCACATCTGTTAAAAGATAACGCAGGTGTCCTAAGATGAGCTCAACGAGAACAGAAATCTCGTGTGGAA	13 (0.000249%)
ACATTGTTCCATCGACCAGAGGCTGTTCACCTTGGAGACCTGATGCGGTTATGAGTACGACCGGGCGTGAGCGGCACTCGGTCCTCCGGATTTTCAAGGG	3 (0.000057%)
ACATTTCGTTCATCACCCTTGGCCGGCTTTCGAACAGCCGGACTCCCATCAAAAGATGGTTGCCAAGAACATCTTCGTTACGGTTTGCTAATTCTCGGAA	3 (0.000057%)
ACATTTTATCGGTCGCTCTTGTCCGGAAGCTGTAGATGACCCAAAGTCCATATAGCGACCCCAGGTCAGGCGGGATTACCCGCTGAGTTTAAGCATATCA	3 (0.000057%)
ACCAGGGGTTGAAATCGTCGACCAGGTCCGAGACTTCATCGACCGGGTCCGAGGATTCGTCGACCAGGACGGCCGGATGTCCGAGAAAAAAAAATGTTGC	6 (0.000115%)
ACCATCCTTTGCTGATGCGGGACGGAAGCTGGTCTCCCGTGTGTTACCGCACGCGGTTGGCCTAAATCCGAGCCAAGGACGCCTGGAGCGTACCGACATG	9 (0.000172%)
ACCGGACACCACGCGACGTGCGGTGCTCTTCCAGCCGCTGGACCCTACCTCCGGCTGAGCCGTTTCCAGGGTGGGCAGGCTGTTAAACAGAAAAGATAAC	5 (0.000096%)
ACCGGGACGTGGCGGTTGACGGCAACGTTAGGGAGTCCGGAGACGTCGGCGGGGGCCTCGGGAAGAGTTATCTTTTCTGTTTAACAGCCTGCCCACCCTG	5 (0.000096%)
ACCTAGGCGAGACAAGGGTTCACATTTCGTTCATCACCCTTGGCCGGCTATCGAACAGCCGGACTCCCATCAAAAGATGGTTGCCAAGAACATCTTCGTT	4 (0.000077%)
ACCTCAGCCTGCTAACTAGCTACGTGGAGGCATCCCTTCACGGCCGGCTTCTTAGAGGGACTATGGCCGTTTAGGCCAAGGAAGTTTGAGGCAATAACAG	10 (0.000192%)
ACGAAACACAGGCCCCGGAACTCATCATCGAGCGTAACATCGCCCGTGAATTAACTGAGAAGGATAGGTGGTAGGTAGTTCGATGCGCGAGCATGGAGCC	33 (0.000632%)
ACGAAGCACGCCCATCCAACCTAGGCGAGACAAGGGTTCACATTTCGTTCATCACCCTTGGCCGGCTATCGAACAGCCGGACTCCCATCAAAAGATGGTT	4 (0.000077%)
ACGCATCATTCAAATTTCTGCCCTATCAACTTTCGATGGTAGGATAGTGGCCTACCATGGTGGTAACGGGTGACGGAGAATTAGGGTTCGATTCCGGAGA	3 (0.000057%)
ACGCCCATCCAACCTAGGCGAGACAAGGGTTCACATTTCGTTCATCACCCTTGGCCGGCTTTCGAACAGCCGGACTCCCATCAAAAGATGGTTGCCAAGA	2059 (0.039460%)
ACGCGCCTAACGGCGTGCCTCGGCATCAGCGTGCTCCGGGCGTCGGCCTGTGGGCTCCCCATTCGACCCGTCTTGAAACACGGACCAAGGAGTCTGACAT	14 (0.000268%)
ACGCTTTCACGGTTCGTATTCGTACTGAAAATCAGAATCAAACGAGCTTTTACCCTTTTGTTCCACACGAGATTTCTGTTCTCGTTGAGCTCATCTTAGG	11 (0.000211%)
ACGGCTTTGGCTCGGATTCGTCCGTCTTCTTTCTTCTTAGCCGAGTACTTCGGTAGATTAGTTGGAACGATTGATGATTTTGAGTTAATTGAACGTTCGG	7 (0.000134%)
ACGGGCAGAGCCCGCGTCGACCTTTTATCTAATAAATGCGTCCCTTCCATAAGTCGGGGTTTGTTGCACGTATTAGCTCTAGAATTACTACGGTTATCCG	3 (0.000057%)
ACGGGTTTACTCACCCGTTGACTCGCACACATGTCAGACTCCTTGGTCCGTGTTTCAAGACGGGTCGAATGGGGAGCCCACAGGCCGACGCCCGGAGCAC	5 (0.000096%)
ACGGTGGTCATGGAAGTCGAAATCCGCTAAGGAGTGTGTAACAACTCACCTGCCGAATCAACTAGCCCCGAAAATGGATGGCGCTTAAGCGCGCGACCTA	10 (0.000192%)
ACGTGGGTGGTTCGCCGCCCGCGACGTCGCGAGAAGTCCACTAAACCTTATCATTTAGAGGAAGGAGAAGTCGTAACAAGGTTTCCGTAGGTGAACCTGC	5 (0.000096%)
ACTACCTACCACCTATCCTTCTCAGTTAATTCACGGGCGATGTTACGCTCGATGATGAGTTCCGGGGCCTGTGTTTCGTACCTAATTTGAAGGAATTGTT	4 (0.000077%)
ACTAGCTACGTGGAGGCATCCCTTCACGGCCGGCTTCTTAGAGGGACTATGGCCGTTTAGGCCAAGGAAGTTTGAGGCAATAACAGGTCTGTGATGCCCT	4 (0.000077%)
ACTCAATCATACACATGACATCAAGTCATATTCGACTCCAAAACACTAACCAACCTTCTTCTTGCTTCTCAAAGCTTTCATGGTGTAGCCAAAGTCCATA	31 (0.000594%)
ACTCCAAAACACTAACCAACCTTCTTCTTGCTTCTCAAAGCTTTCATGGTGTAGCCAAAGTCCATATGAGTCTTTGGCTTTGTGTCTTCTAACAAGGAAA	388 (0.007436%)
ACTCCAAAACACTAACCAACCTTCTTCTTGCTTCTCAAAGCTTTCATGGTGTAGCCAAAGTCCATATGAGTCTTTGGCTTTGTGTCTTCTAACAAGGATA	3 (0.000057%)
ACTCGGTCCTCCGGATTTTCAAGGGCCGCCGGGGGCGCACCGGACACCACGCGACGTGCGGTGCTCTTCCAGCCGCTGGACCCTACCTCCGGCTGAGCCG	11 (0.000211%)
ACTTCCCTTGCCTACATTGTTCCATCGACCAGAGGCTGTTCACCTTGGAGACCTGATGCGGTTATGAGTACGACCGGGCGTGAGCGGCACTCGGTCCTCC	18 (0.000345%)
AGAAGACACAAAGCCAAAGACTCATATGGACTTTGGCTACACCATGAAAGCTTTGAGAAGCAAGAAGAAGGTTGGTTAGTGTTTTGGAGTCGAATATGAC	64 (0.001227%)
AGAATTGTATCCTTGTTAGAAGACACAAAGCCAAAGACTCATATGGACTTTGGCTACACCATGAAAGCTTTGAGAAGCAAGAAGAAGGTTGGTTAGTGTT	3 (0.000057%)
AGACAAGGGTTCACATTTCGTTCATCACCCTTGGCCGGCTATCGAACAGCCGGACTCCCATCAAAAGATGGTTGCCAAGAACATCTTCGTTACGGTTTGC	6 (0.000115%)
AGACACAAAGCCAAAGACTCATATGGACTTTGGCTACACCATGAAAGCTTTGAGAAGCAAGAAGAAGGTTGGTTAGTGTTTTGGAGTCGAATATGACTTG	33 (0.000632%)
AGACAGCAGGACGGTGGTCATGGAAGTCGAAATCCGCTAAGGAGTGTGTAACAACTCACCTGCCGAATCAACTAGCCCCGAAAATGGATGGCGCTTAAGC	7 (0.000134%)
AGAGCAACGGGCAGAGCCCGCGTCGACCTTTTATCTAATAAATGCGTCCCTTCCATAAGTCGGGGTTTGTTGCACGTATTAGCTCTAGAATTACTACGGT	31 (0.000594%)
AGAGCCCGCGTCGACCTTTTATCTAATAAATGCGTCCCTTCCATAAGTCGGGGTTTGTTGCACGTATTAGCTCTAGAATTACTACGGTTATCCGAGTAGT	7 (0.000134%)

AGAGCGTAGGCTTGCTTTGAGCACTCTAATTTCTTCAAAGTAACAGCGCCGGAGGCACGACCCGGCCAATTAAGACCAGGAGCGTATCGCCGACCGAAGG	15 (0.000287%)
AGAGGCTGTTCACCTTGGAGACCTGATGCGGTTATGAGTACGACCGGGCGTGAGCGGCACTCGGTCCTCCGGATTTTCAAGGGCCGCCGGGGGCGCACCG	150 (0.002875%)
AGATCGGAAGAGCGTCGTGTAGGGAAAGAGTGTTCAGAGC	47 (0.000360%)
AGCAACGGGCAGAGCCCGCGTCGACCTTTTATCTAATAAATGCGTCCCTTCCATAAGTCGGGGTTTGTTGCACGTATTAGCTCTAGAATTACTACGGTTA	7 (0.000134%)
AGCACGCCCATCCAACCTAGGCGAGACAAGGGTTCACATTTCGTTCATCACCCTTGGCCGGCTATCGAACAGCCGGACTCCCATCAAAAGATGGTTGCCA	12 (0.000230%)
AGCACGCGCCTAACGGCGTGCCTCGGCATCAGCGTGCTCCGGGCGTCGGCCTGTGGGCTCCCCATTCGACCCGTCTTGAAACACGGACCAAGGAGTCTGA	31 (0.000594%)
AGCAGCCGCGGTAATTCCAGCTCCAATAGCGTATATTTAAGTTGTTGCAGTTAAAAAGCTCGTAGTTGAACCTTGGGATGGGTCGGCCGGTCCGCCTTTG	6 (0.000115%)
AGCAGGACGGTGGTCATGGAAGTCGAAATCCGCTAAGGAGTGTGTAACAACTCACCTGCCGAATCAACTAGCCCCGAAAATGGATGGCGCTTAAGCGCGC	6 (0.000115%)
AGCAGTTCTCGGACAAAAATTGCTGAGTGGCCGAGAAGAATGGGCGTGTCATGCGTGGGCTGACATGGATTCTTCGAGGCCTAGGGGTGGCGGTATATAA	6 (0.000115%)
AGCCAAAGACTCATATGGACTTTGGCTACACCATGAAAGCTTTGAGAAGCAAGAAGAAGGTTGGTTAGTGTTTTGGAGTCGAATATGACTTGATGTCATG	8 (0.000153%)
AGCCGCGGTAATTCCAGCTCCAATAGCGTATATTTAAGTTGTTGCAGTTAAAAAGCTCGTAGTTGAACCTTGGGATGGGTCGGCCGGTCCGCCTTTGGTG	8 (0.000153%)
AGCCTAAGTAGTGTTTCCTTGTTAGAAGACACAAAGCCAAAGACTCATATGGACTTTGGCTACACCATGAAAGCTTTGAGAAGCAAGAAGAAGGTTGGTT	33 (0.000632%)
AGCCTGCTAACTAGCTACGTGGAGGCATCCCTTCACGGCCGGCTTCTTAGAGGGACTATGGCCGTTTAGGCCAAGGAAGTTTGAGGCAATAACAGGTCTG	6 (0.000115%)
AGCGAGGTGTGAGTGTCGCCCATGGGCATCGACACCTTGCGGCTAGGAACTGGAACGAGACGGGTGGCAAAGATTTCGAGTAGCACTTCATACTACCGTG	7 (0.000134%)
AGCGGCACTCGGTCCTCCGGATTTTCAAGGGCCGCCGGGGGCGCACCGGACACCACGCGACGTGCGGTGCTCTTCCAGCCGCTGGACCCTACCTCCGGCT	3 (0.000057%)
AGCGTAGGCTTGCTTTGAGCACTCTAATTTCTTCAAAGTAACAGCGCCGGAGGCACGACCCGGCCAATTAAGACCAGGAGCGTATCGCCGACCGAAGGGA	3 (0.000057%)
AGCGTGCTCCGGGCGTCGGCCTGTGGGCTCCCCATTCGACCCGTCTTGAAACACGGACCAAGGAGTCTGACATGTGTGCGAGTCAACGGGTGAGTAAACC	4 (0.000077%)
AGCTAGTGTTCGTAGGCTCCATGCTCGCGCATCGAACTACCTACCACCTATCCTTCTCAGTTAATTCACGGGCGATGTTACGCTCGATGATGAGTTCCGG	12 (0.000230%)
AGCTCCAATAGCGTATATTTAAGTTGTTGCAGTTAAAAAGCTCGTAGTTGAACCTTGGGATGGGTCGGCCGGTCCGCCTTTGGTGTGCATTGGTCGGCTT	3 (0.000057%)
AGCTTCCTTGCGCCTTACGGGTTTACTCACCCGTTGACTCGCACACATGTCAGACTCCTTGGTCCGTGTTTCAAGACGGGTCGAATGGGGAGCCCACAGG	24 (0.000460%)
AGGACATTGTCAGGTGGGGAGTTTGGCTGGGGCGGCACATCTGTTAAAAGATAACGCAGGTGTCCTAAGATGAGCTCAACGAGAACAGAAATCTCGTGTG	150 (0.002875%)
AGGACGGTGGTCATGGAAGTCGAAATCCGCTAAGGAGTGTGTAACAACTCACCTGCCGAATCAACTAGCCCCGAAAATGGATGGCGCTTAAGCGCGCGAC	35 (0.000671%)
AGGAGCGACGGGCGGTGTGTACAAAGGGCAGGGACGTAGTCAACGCGAGCTGATGACTCGCGCTTACTAGGAATTCCTCGTTGAAGACCAACAATTGCAA	19 (0.000364%)
AGGAGCTGTTGCTTTGTTAGTGTAGAAACACTTGTGTAGAATTGGGGATTGTTTTTTTTGGAGTGATTTAGGGGAGGGTCGAATCTTAGCGACAAAGGGC	12 (0.000230%)
AGGCGAGACAAGGGTTCACATTTCGTTCATCACCCTTGGCCGGCTATCGAACAGCCGGACTCCCATCAAAAGATGGTTGCCAAGAACATCTTCGTTACGG	3 (0.000057%)
AGGCGAGACAAGGGTTCACATTTCGTTCATCACCCTTGGCCGGCTTTCGAACAGCCGGACTCCCATCAAAAGATGGTTGCCAAGAACATCTTCGTTACGG	4 (0.000077%)
AGGCGTAAGAATTGTATCCTTGTTAGAAGACACAAAGCCA	111 (0.000851%)
AGGCTGTCCCGAGTGTGAGCGAGGTGTGAGTGTCGCCCATGGGCATCGACACCTTGCGGCTAGGAACTGGAACGAGACGGGTAGCAAAGATTTCGAGTAG	1874 (0.035914%)
AGGCTGTCCCGAGTGTGAGCGAGGTGTGAGTGTCGCCCATGGGCATCGACACCTTGCGGCTAGGAACTGGAACGAGACGGGTGGCAAAGATTTCGAGTAG	13 (0.000249%)
AGGCTGTTCACCTTGGAGACCTGATGCGGTTATGAGTACGACCGGGCGTGAGCGGCACTCGGTCCTCCGGATTTTCAAGGGCCGCCGGGGGCGCACCGGA	33 (0.000632%)
AGGGACAGTCGGGGGCATTCGTATTTCATAGTCAGAGGTGAAATTCTTGGATTTATGAAAGACGAACAACTGCGAAAGCATTTGCCAAGGATGTTTTCAT	6 (0.000115%)
AGGGAGTCCGGAGACGTCGGCGGGGGCCTCGGGAAGAGTTATCTTTTCTGTTTAACAGCCTGCCCACCCTGGAAACGGCTCAGCCGGAGGTAGGGTCCAG	7 (0.000134%)
AGGGCAAGTCTGGTGCCAGCAGCCGCGGTAATTCCAGCTCCAATAGCGTATATTTAAGTTGTTGCAGTTAAAAAGCTCGTAGTTGAACCTTGGGATGGGT	27 (0.000517%)
AGGGGTTGAAATCGTCGACCAGGTCCGAGACTTCATCGACCGGGTCCGAGGATTCGTCGACCAGGACGGCCGGATGTCCGAGAAAAAAAAATGTTGCCGA	4 (0.000077%)
AGGGTGTTGGTCGATTAAGACAGCAGGACGGTGGTCATGGAAGTCGAAATCCGCTAAGGAGTGTGTAACAACTCACCTGCCGAATCAACTAGCCCCGAAA	13 (0.000249%)
AGGTATCTCGCGCTTGTACGGCTTTGGCTCGGATTCGTCCGTCTTCTTTCTTCTTAGCCGAGTACTTCGGTAGATTAGTTGGAACGATTGATGATTTTGA	6 (0.000115%)
AGTAGTCATATGCTTGTCTCAAAGATTAAGCCATGCATGTGTAAGTATGAACGAATTCAGACTGTGAAACTGCGAATGGCTCATTAAATCAGTTATAGTT	2299 (0.044059%)
AGTAGTGTTTCCTTGTTAGAAGACACAAAGCCAAAGACTCATATGGACTTTGGCTACACCATGAAAGCTTTGAGAAGCAAGAAGAAGGTTGGTTAGTGTT	14 (0.000268%)
AGTATAAGAACTTAAACCGCAACCGCATCTTATAAGCCTAAGTAGTGTTTCCTTGTTAGAAGACACAAAGCCAAAGACTCATATGGACTTTGGCTACACC	3 (0.000057%)
AGTCATATGCTTGTCTCAAAGATTAAGCCATGCATGTGTAAGTATGAACGAATTCAGACTGTGAAACTGCGAATGGCTCATTAAATCAGTTATAGTTTGT	13 (0.000249%)
AGTCATATTCGACTCCAAAACACTAACCAACCTTCTTCTTGCTTCTCAAAGCTTTCATGGTGTAGCCAAAGTCCATATGAGTCTTTGGCTTTGTGTCTTC	6 (0.000115%)
AGTCGAATATGACTTGATGTCATGTGTATGATTGAGTATA	1268 (0.009720%)
AGTCGGGGGCATTCGTATTTCATAGTCAGAGGTGAAATTCTTGGATTTATGAAAGACGAACAACTGCGAAAGCATTTGCCAAGGATGTTTTCATTAATCA	4 (0.000077%)

AGTGTATCCTTGTTAGAAGACACAAAGCCAAAGACTCATA	8146 (0.062446%)
AGTGTTCGTAGGCTCCATGCTCGCGCATCGAACTACCTACCACCTATCCTTCTCAGTTAATTCACGGGCGATGTTACGCTCGATGATGAGTTCCGGGGCC	210 (0.004025%)
AGTGTTTCCTTGTTAGAAGACACAAAGCCAAAGACTCATATGGACTTTGGCTACACCATGAAAGCTTTGAGAAGCAAGAAGAAGGTTGGTTAGTGTTTTG	10 (0.000192%)
AGTGTTTTGGAGTCGAATATGACTTGATGTCATGTGTATG	181 (0.001388%)
AGTTATCATGAATCATCAGAGCAACGGGCAGAGCCCGCGTCGACCTTTTATCTAATAAATGCGTCCCTTCCATAAGTCGGGGTTTGTTGCACGTATTAGC	12 (0.000230%)
AGTTCTTATACTCAATCATACACATGACATCAAGTCATATTCGACTCCAAAACACTAACCAACCTTCTTCTTGCTTCTCAAAGCTTTCATGGTGTAGCCA	25 (0.000479%)
AGTTGTTATACTCAATCATACACATGACAACAAGTCATATTCGACTCCAAAACACTAACCAACCTTCTTCTTGCTTCTCAAAGCTTTCATGGTGTAGCCA	19 (0.000364%)
AGTTGTTATACTCAATCATACACATGACATCAAGTCATAT	97 (0.000744%)
AGTTTGGCTGGGGCGGCACATCTGTTAAAAGATAACGCAGGTGTCCTAAGATGAGCTCAACGAGAACAGAAATCTCGTGTGGAACAAAAGGGTAAAAGCT	5 (0.000096%)
AGTTTTTTCAGCAGTTCTCGGACAAAAATTGCTGAGTGGCCGAGAAGAATGGGCGTGTCATGCGTGGGCTGACATGGATTCTTCGAGGCCTAGGGGTGGC	4 (0.000077%)
ATAAAAGTGTCGGAGTTTTTTCAGCAGTTCTCGGACAAAAATTGCTGAGTGGCCGAGAAGAATGGGCGTGTCATGCGTGGGCTGACATGGATTCTTCGAG	155 (0.002971%)
ATAAGAACTTAAACCGCAACCGCATCTTATAAGCCTAAGTAGTGTTTCCTTGTTAGAAGACACAAAGCCAAAGACTCATATGGACTTTGGCTACACCATG	24 (0.000460%)
ATACACATGACATCAAGTCATATTCGACTCCAAAACACTAACCAACCTTCTTCTTGCTTCTCAAAGCTTTCATGGTGTAGCCAAAGTCCATATGAGTCTT	286 (0.005481%)
ATACGGGCGAGAGACCGATAGCGAACAAGTACCGCGAGGTAAAGATGAAAAGGACTTTGAAAAGAGAGTCAAAGAGTGCTTGAAATTGTCGGGAGGGAAG	14 (0.000268%)
ATACTCAATCATACACATGACATCAAGTCATATTCGACTCCAAAACACTAACCAACCTTCTTCTTGCTTCTCAAAGCTTTCATGGTGTAGCCAAAGTCCA	29 (0.000556%)
ATAGCTAGTGTTCGTAGGCTCCATGCTCGCGCATCGAACTACCTACCACCTATCCTTCTCAGTTAATTCACGGGCGATGTTACGCTCGATGATGAGTTCC	4 (0.000077%)
ATATGCTTGTCTCAAAGATTAAGCCATGCATGTGTAAGTATGAACGAATTCAGACTGTGAAACTGCGAATGGCTCATTAAATCAGTTATAGTTTGTTTGA	47 (0.000901%)
ATATTCGACTCCAAAACACTAACCAACCTTCTTCTTGCTTCTCAAAGCTTTCATGGTGTAGCCAAAGTCCATATGAGTCTTTGGCTTTGTGTCTTCTAAC	150 (0.002875%)
ATATTCGACTCCAAAACACTAACCAACCTTCTTCTTGCTTCTCAAAGCTTTCATGGTGTAGCCAAAGTCCATATGAGTCTTTGGCTTTGTGTCTTTTAAC	61 (0.001169%)
ATATTTAAGTTGTTGCAGTTAAAAAGCTCGTAGTTGAACCTTGGGATGGGTCGGCCGGTCCGCCTTTGGTGTGCATTGGTCGGCTTGTCCCTTCGGTCGG	7 (0.000134%)
ATCAACTTTCGATGGTAGGATAGTGGCCTACCATGGTGGTAACGGGTGACGGAGAATTAGGGTTCGATTCCGGAGAGGGAGCCTGAGAAACGGCTACCAC	9 (0.000172%)
ATCAAGTCATATTCGACTCCAAAACACTAACCAACCTTCTTCTTGCTTCTCAAAGCTTTCATGGTGTAGCCAAAGTCCATATGAGTCTTTGGCTTTGTGT	1348 (0.025834%)
ATCAATCGTTCCAACTAATCTACCGAAGTACTCGGCTAAGAAGAAAGAAGACGGACGAATCCGAGCCAAAGCCGTACAAGCGCGAGATACCTTCGGGACA	5 (0.000096%)
ATCAGAGCAACGGGCAGAGCCCGCGTCGACCTTTTATCTAATAAATGCGTCCCTTCCATAAGTCGGGGTTTGTTGCACGTATTAGCTCTAGAATTACTAC	36 (0.000690%)
ATCAGCGTGCTCCGGGCGTCGGCCTGTGGGCTCCCCATTCGACCCGTCTTGAAACACGGACCAAGGAGTCTGACATGTGTGCGAGTCAACGGGTGAGTAA	4 (0.000077%)
ATCAGCTTCCTTGCGCCTTACGGGTTTACTCACCCGTTGACTCGCACACATGTCAGACTCCTTGGTCCGTGTTTCAAGACGGGTCGAATGGGGAGCCCAC	65 (0.001246%)
ATCATACACATGACATCAAGTCATATTCGACTCCAAAACACTAACCAACCTTCTTCTTGCTTCTCAAAGCTTTCATGGTGTAGCCAAAGTCCATATGAGT	24 (0.000460%)
ATCATCAGAGCAACGGGCAGAGCCCGCGTCGACCTTTTATCTAATAAATGCGTCCCTTCCATAAGTCGGGGTTTGTTGCACGTATTAGCTCTAGAATTAC	12 (0.000230%)
ATCATGAATCATCAGAGCAACGGGCAGAGCCCGCGTCGACCTTTTATCTAATAAATGCGTCCCTTCCATAAGTCGGGGTTTGTTGCACGTATTAGCTCTA	53 (0.001016%)
ATCCAACCTAGGCGAGACAAGGGTTCACATTTCGTTCATCACCCTTGGCCGGCTATCGAACAGCCGGACTCCCATCAAAAGATGGTTGCCAAGAACATCT	25 (0.000479%)
ATCCAACCTAGGCGAGACAAGGGTTCACATTTCGTTCATCACCCTTGGCCGGCTTTCGAACAGCCGGACTCCCATCAAAAGATGGTTGCCAAGAACATCT	27 (0.000517%)
ATCCAGAGCGTAGGCTTGCTTTGAGCACTCTAATTTCTTCAAAGTAACAGCGCCGGAGGCACGACCCGGCCAATTAAGACCAGGAGCGTATCGCCGACCG	17 (0.000326%)
ATCCCATGCTAATGTATCCAGAGCGTAGGCTTGCTTTGAGCACTCTAATTTCTTCAAAGTAACAGCGCCGGAGGCACGACCCGGCCAATTAAGACCAGGA	23 (0.000441%)
ATCCCGCCAATCAGCTTCCTTGCGCCTTACGGGTTTACTCACCCGTTGACTCGCACACATGTCAGACTCCTTGGTCCGTGTTTCAAGACGGGTCGAATGG	7 (0.000134%)
ATCCGTCGAGTTATCATGAATCATCAGAGCAACGGGCAGAGCCCGCGTCGACCTTTTATCTAATAAATGCGTCCCTTCCATAAGTCGGGGTTTGTTGCAC	10 (0.000192%)
ATCCTATGATGTTATCCCATGCTAATGTATCCAGAGCGTAGGCTTGCTTTGAGCACTCTAATTTCTTCAAAGTAACAGCGCCGGAGGCACGACCCGGCCA	44 (0.000843%)
ATCCTTGTTAGAAGACACAAAGCCAAAGACTCATATGGACTTTGGCTACACCATGAAAGCTTTGAGAAGCAAGAAGAAGGTTGGTTAGTGTTTTGGAGTC	13 (0.000249%)
ATCGAAATCCTATGATGTTATCCCATGCTAATGTATCCAGAGCGTAGGCTTGCTTTGAGCACTCTAATTTCTTCAAAGTAACAGCGCCGGAGGCACGACC	15 (0.000287%)
ATCGACACCTTGCGGCTAGGAACTGGAACGAGACGGGTGGCAAAGATTTCGAGTAGCACTTCATACTACCGTGGGTTTTTTAAACCTTCCGAGTTTTGTT	10 (0.000192%)
ATCGACCCGCCGAAGCGAGCCTTGGGACCAAAAACAGGGGTTGTACCCCGCCTCCGATTCACGGAGTAAGTAAAATAACGTTAAAAGTAGTGGTATTTCA	6 (0.000115%)
ATCGATCCATTACATTTTATCGGTCGCTCTTGTCCGGAAGCTGTAGATGACCCAAAGTCCATATAGCGACCCCAGGTCAGGCGGGATTACCCGCTGAGTT	19 (0.000364%)
ATCGATCCCGCCAATCAGCTTCCTTGCGCCTTACGGGTTTACTCACCCGTTGACTCGCACACATGTCAGACTCCTTGGTCCGTGTTTCAAGACGGGTCGA	1139 (0.021828%)
ATCGATCCGTAACTTCGGGAAAAGGATTGGCTCTGAGGGCTGGGCTCGGGGGTCCCAGTTCCGAACCCGTCGGCTGTCAGCGGACTGCTCGAGCTGCTTC	30 (0.000575%)

ATCGATCCGTCGAGTTATCATGAATCATCAGAGCAACGGGCAGAGCCCGCGTCGACCTTTTATCTAATAAATGCGTCCCTTCCATAAGTCGGGGTTTGTT	19 (0.000364%)
ATCGATCGACCCGCCGAAGCGAGCCTTGGGACCAAAAACAGGGGTTGTACCCCGCCTCCGATTCACGGAGTAAGTAAAATAACGTTAAAAGTAGTGGTAT	14 (0.000268%)
ATCGATCTTAAAGGCGTAAGAATTGTATCCTTGTTAAAAGACACAAAGCCAAAGACTCATATGGACTTTGGCTACACCATGAAAGCTTTGAGAAGCAAGA	11 (0.000211%)
ATCGATCTTCTGAGAAGGGTTCGAGTGTGAGCATGCCTGTCGGGACCCGAAAGATGGTGAACTATGCCTGAGCGGGGTAAAGCCAGAGGAAACTCTGGTG	20 (0.000383%)
ATCGGTAGGAGCGACGGGCGGTGTGTACAAAGGGCAGGGACGTAGTCAACGCGAGCTGATGACTCGCGCTTACTAGGAATTCCTCGTTGAAGACCAACAA	65 (0.001246%)
ATCGTCGTCCCTCACCATCCTTTGCTGATGCGGGACGGAAGCTGGTCTCCCGTGTGTTACCGCACGCGGTTGGCCTAAATCCGAGCCAAGGACGCCTGGA	9 (0.000172%)
ATCTCGCGCTTGTACGGCTTTGGCTCGGATTCGTCCGTCTTCTTTCTTCTTAGCCGAGTACTTCGGTAGATTAGTTGGAACGATTGATGATTTTGAGTTA	3 (0.000057%)
ATCTTAAAGGCGTAAGAATTGTATCCTTGTTAAAAGACACAAAGCCAAAGACTCATATGGACTTTGGCTACACCATGAAAGCTTTGAGAAGCAAGAAGAA	36 (0.000690%)
ATCTTAAAGGCGTAAGAATTGTATCCTTGTTAGAAGACAC	1341 (0.010280%)
ATCTTCTGAGAAGGGTTCGAGTGTGAGCATGCCTGTCGGGACCCGAAAGATGGTGAACTATGCCTGAGCGGGGTAAAGCCAGAGGAAACTCTGGTGGAAG	3 (0.000057%)
ATGAACACAAACGTTCAATATGACAAACCCATGCCAAGTAAAGAGAAAATGAAAACTGGTGATTGTTGCGGAAATCGTCCAGGATTCCTCGACCAGGACT	12 (0.000230%)
ATGAATCATCAGAGCAACGGGCAGAGCCCGCGTCGACCTTTTATCTAATAAATGCGTCCCTTCCATAAGTCGGGGTTTGTTGCACGTATTAGCTCTAGAA	68 (0.001303%)
ATGACATCAAGTCATATTCGACTCCAAAACACTAACCAACCTTCTTCTTGCTTCTCAAAGCTTTCATGGTGTAGCCAAAGTCCATATGAGTCTTTGGCTT	116 (0.002223%)
ATGATGTTATCCCATGCTAATGTATCCAGAGCGTAGGCTTGCTTTGAGCACTCTAATTTCTTCAAAGTAACAGCGCCGGAGGCACGACCCGGCCAATTAA	68 (0.001303%)
ATGATTAACAGGGACAGTCGGGGGCATTCGTATTTCATAGTCAGAGGTGAAATTCTTGGATTTATGAAAGACGAACAACTGCGAAAGCATTTGCCAAGGA	98 (0.001878%)
ATGATTGAGTATAAGAACTTAAACCGCAACCGCATCTTATAAGCCTAAGTAGTGTTTCCTTGTTAGAAGACACAAAGCCAAAGACTCATATGGACTTTGG	27 (0.000517%)
ATGCTAATGTATCCAGAGCGTAGGCTTGCTTTGAGCACTCTAATTTCTTCAAAGTAACAGCGCCGGAGGCACGACCCGGCCAATTAAGACCAGGAGCGTA	10 (0.000192%)
ATGCTCGCGCATCGAACTACCTACCACCTATCCTTCTCAGTTAATTCACGGGCGATGTTACGCTCGATGATGAGTTCCGGGGCCTGTGTTTCGTACCTAA	5 (0.000096%)
ATGCTTGTCTCAAAGATTAAGCCATGCATGTGTAAGTATGAACGAATTCAGACTGTGAAACTGCGAATGGCTCATTAAATCAGTTATAGTTTGTTTGATG	20 (0.000383%)
ATGGAAGTCGAAATCCGCTAAGGAGTGTGTAACAACTCACCTGCCGAATCAACTAGCCCCGAAAATGGATGGCGCTTAAGCGCGCGACCTATACCCGGCC	36 (0.000690%)
ATGGCCTCTGTGCTGGCGACGCATCATTCAAATTTCTGCCCTATCAACTTTCGATGGTAGGATAGTGGCCTACCATGGTGGTAACGGGTGACGGAGAATT	392 (0.007513%)
ATGGGCATCGACACCTTGCGGCTAGGAACTGGAACGAGACGGGTGGCAAAGATTTCGAGTAGCACTTCATACTACCGTGGGTTTTTTAAACCTTCCGAGT	5 (0.000096%)
ATGTATCCAGAGCGTAGGCTTGCTTTGAGCACTCTAATTTCTTCAAAGTAACAGCGCCGGAGGCACGACCCGGCCAATTAAGACCAGGAGCGTATCGCCG	6 (0.000115%)
ATGTGTAAGTATGAACGAATTCAGACTGTGAAACTGCGAATGGCTCATTAAATCAGTTATAGTTTGTTTGATGGTAACTACTACTCGGATAACCGTAGTA	184 (0.003526%)
ATGTGTATGATTGAGTATAAGAACTTAAACCGCAACCGCATCTTATAAGCCTAAGTAGTGTTTCCTTGTTAGAAGACACAAAGCCAAAGACTCATATGGA	16 (0.000307%)
ATGTTATCCCATGCTAATGTATCCAGAGCGTAGGCTTGCTTTGAGCACTCTAATTTCTTCAAAGTAACAGCGCCGGAGGCACGACCCGGCCAATTAAGAC	9 (0.000172%)
ATTAACAGGGACAGTCGGGGGCATTCGTATTTCATAGTCAGAGGTGAAATTCTTGGATTTATGAAAGACGAACAACTGCGAAAGCATTTGCCAAGGATGT	85 (0.001629%)
ATTAAGACAGCAGGACGGTGGTCATGGAAGTCGAAATCCGCTAAGGAGTGTGTAACAACTCACCTGCCGAATCAACTAGCCCCGAAAATGGATGGCGCTT	18 (0.000345%)
ATTACATTTTATCGGTCGCTCTTGTCCGGAAGCTGTAGATGACCCAAAGTCCATATAGCGACCCCAGGTCAGGCGGGATTACCCGCTGAGTTTAAGCATA	7 (0.000134%)
ATTAGGTACGAAACACAGGCCCCGGAACTCATCATCGAGCGTAACATCGCCCGTGAATTAACTGAGAAGGATAGGTGGTAGGTAGTTCGATGCGCGAGCA	16 (0.000307%)
ATTAGTCTTTCGCCCCTATACCCAAGTCAGACGAACGATTTGCACGTCAGTATCGCTGCGGGCTTCCACCAGAGTTTCCTCTGGCTTTACCCCGCTCAGG	24 (0.000460%)
ATTCAAATTTCTGCCCTATCAACTTTCGATGGTAGGATAGTGGCCTACCATGGTGGTAACGGGTGACGGAGAATTAGGGTTCGATTCCGGAGAGGGAGCC	17 (0.000326%)
ATTCAATCGGTAGGAGCGACGGGCGGTGTGTACAAAGGGCAGGGACGTAGTCAACGCGAGCTGATGACTCGCGCTTACTAGGAATTCCTCGTTGAAGACC	1022 (0.019586%)
ATTCCAGCTCCAATAGCGTATATTTAAGTTGTTGCAGTTAAAAAGCTCGTAGTTGAACCTTGGGATGGGTCGGCCGGTCCGCCTTTGGTGTGCATTGGTC	10 (0.000192%)
ATTCGACTCCAAAACACTAACCAACCTTCTTCTTGCTTCTCAAAGCTTTCATGGTGTAGCCAAAGTCCATATGAGTCTTTGGCTTTGTGTCTTCTAACAA	108 (0.002070%)
ATTCGACTCCAAAACACTAACCAACCTTCTTCTTGCTTCTCAAAGCTTTCATGGTGTAGCCAAAGTCCATATGAGTCTTTGGCTTTGTGTCTTTTAACAA	221 (0.004235%)
ATTCGTACTGAAAATCAGAATCAAACGAGCTTTTACCCTTTTGTTCCACACGAGATTTCTGTTCTCGTTGAGCTCATCTTAGGACACCTGCGTTATCTTT	5 (0.000096%)
ATTCGTATTTCATAGTCAGAGGTGAAATTCTTGGATTTATGAAAGACGAACAACTGCGAAAGCATTTGCCAAGGATGTTTTCATTAATCAAGAACGAAAG	6 (0.000115%)
ATTGAGTATAAGAACTTAAACCGCAACCGCATCTTATAAGCCTAAGTAGTGTTTCCTTGTTAGAAGACACAAAGCCAAAGACTCATATGGACTTTGGCTA	26 (0.000498%)
ATTGGAGGGCAAGTCTGGTGCCAGCAGCCGCGGTAATTCCAGCTCCAATAGCGTATATTTAAGTTGTTGCAGTTAAAAAGCTCGTAGTTGAACCTTGGGA	1512 (0.028977%)
ATTGTATCCTTGTTAGAAGACACAAAGCCAAAGACTCATATGGACTTTGGCTACACCATGAAAGCTTTGAGAAGCAAGAAGAAGGTTGGTTAGTGTTTTG	22 (0.000422%)
ATTGTCAGGTGGGGAGTTTGGCTGGGGCGGCACATCTGTTAAAAGATAACGCAGGTGTCCTAAGATGAGCTCAACGAGAACAGAAATCTCGTGTGGAACA	34 (0.000652%)
ATTGTTCCATCGACCAGAGGCTGTTCACCTTGGAGACCTGATGCGGTTATGAGTACGACCGGGCGTGAGCGGCACTCGGTCCTCCGGATTTTCAAGGGCC	13 (0.000249%)

ATTGTTGGTCTTCAACGAGGAATTCCTAGTAAGCGCGAGTCATCAGCTCGCGTTGACTACGTCCCTGCCCTTTGTACACACCGCCCGTCGCTCCTACCGA	31 (0.000594%)	
ATTTCATAGTCAGAGGTGAAATTCTTGGATTTATGAAAGACGAACAACTGCGAAAGCATTTGCCAAGGATGTTTTCATTAATCAAGAACGAAAGTTGGGG	5 (0.000096%)	
ATTTCGTTCATCACCCTTGGCCGGCTATCGAACAGCCGGACTCCCATCAAAAGATGGTTGCCAAGAACATCTTCGTTACGGTTTGCTAATTCTCGGAATA	18 (0.000345%)	
ATTTCGTTCATCACCCTTGGCCGGCTTTCGAACAGCCGGACTCCCATCAAAAGATGGTTGCCAAGAACATCTTCGTTACGGTTTGCTAATTCTCGGAATA	11 (0.000211%)	
ATTTCTGCCCTATCAACTTTCGATGGTAGGATAGTGGCCTACCATGGTGGTAACGGGTGACGGAGAATTAGGGTTCGATTCCGGAGAGGGAGCCTGAGAA	5 (0.000096%)	
ATTTGCACGTCAGTATCGCTGCGGGCTTCCACCAGAGTTTCCTCTGGCTTTACCCCGCTCAGGCATAGTTCACCATCTTTCGGGTCCCGACAGGCATGCT	35 (0.000671%)	
ATTTTATCGGTCGCTCTTGTCCGGAAGCTGTAGATGACCCAAAGTCCATATAGCGACCCCAGGTCAGGCGGGATTACCCGCTGAGTTTAAGCATATCAAT	18 (0.000345%)	
ATTTTCAAGGGCCGCCGGGGGCGCACCGGACACCACGCGACGTGCGGTGCTCTTCCAGCCGCTGGACCCTACCTCCGGCTGAGCCGTTTCCAGGGTGGGC	15 (0.000287%)	
CAAAATCATCAATCGTTCCAACTAATCTACCGAAGTACTCGGCTAAGAAGAAAGAAGACGGACGAATCCGAGCCAAAGCCGTACAAGCGCGAGATACCTT	21 (0.000402%)	
CAAACGTTCAATATGACAAACCCATGCCAAGTAAAGAGAAAATGAAAACTGGTGATTGTTGCGGAAATCGTCCAGGATTCCTCGACCAGGACTTGAAATC	21 (0.000402%)	
CAAAGCCAAAGACTCATATGGACTTTGGCTACACCATGAAAGCTTTGAGAAGCAAGAAGAAGGTTGGTTAGTGTTTTGGAGTCGAATATGACTTGATGTC	92 (0.001763%)	
CAAAGGGTGTTGGTCGATTAAGACAGCAGGACGGTGGTCATGGAAGTCGAAATCCGCTAAGGAGTGTGTAACAACTCACCTGCCGAATCAACTAGCCCCG	25 (0.000479%)	
CAAATCGTCGTCCCTCACCATCCTTTGCTGATGCGGGACGGAAGCTGGTCTCCCGTGTGTTACCGCACGCGGTTGGCCTAAATCCGAGCCAAGGACGCCT	7 (0.000134%)	
CAAATTAGGTACGAAACACAGGCCCCGGAACTCATCATCGAGCGTAACATCGCCCGTGAATTAACTGAGAAGGATAGGTGGTAGGTAGTTCGATGCGCGA	15 (0.000287%)	
CAAATTTCTGCCCTATCAACTTTCGATGGTAGGATAGTGGCCTACCATGGTGGTAACGGGTGACGGAGAATTAGGGTTCGATTCCGGAGAGGGAGCCTGA	154 (0.002951%)	
CAACCTAGGCGAGACAAGGGTTCACATTTCGTTCATCACCCTTGGCCGGCTATCGAACAGCCGGACTCCCATCAAAAGATGGTTGCCAAGAACATCTTCG	3 (0.000057%)	
CAACGAAGCACGCCCATCCAACCTAGGCGAGACAAGGGTTCACATTTCGTTCATCACCCTTGGCCGGCTATCGAACAGCCGGACTCCCATCAAAAGATGG	76 (0.001457%)	
CAACGGGCAGAGCCCGCGTCGACCTTTTATCTAATAAATGCGTCCCTTCCATAAGTCGGGGTTTGTTGCACGTATTAGCTCTAGAATTACTACGGTTATC	31 (0.000594%)	
CAACGTTAGGGAGTCCGGAGACGTCGGCGGGGGCCTCGGGAAGAGTTATCTTTTCTGTTTAACAGCCTGCCCACCCTGGAAACGGCTCAGCCGGAGGTAG	9 (0.000172%)	
CAACTTTCGATGGTAGGATAGTGGCCTACCATGGTGGTAACGGGTGACGGAGAATTAGGGTTCGATTCCGGAGAGGGAGCCTGAGAAACGGCTACCACAT	14 (0.000268%)	
CAAGGGCCGCCGGGGGCGCACCGGACACCACGCGACGTGCGGTGCTCTTCCAGCCGCTGGACCCTACCTCCGGCTGAGCCGTTTCCAGGGTGGGCAGGCT	12 (0.000230%)	
CAAGGGTTCACATTTCGTTCATCACCCTTGGCCGGCTATCGAACAGCCGGACTCCCATCAAAAGATGGTTGCCAAGAACATCTTCGTTACGGTTTGCTAA	30 (0.000575%)	
CAAGGGTTCACATTTCGTTCATCACCCTTGGCCGGCTTTCGAACAGCCGGACTCCCATCAAAAGATGGTTGCCAAGAACATCTTCGTTACGGTTTGCTAA	31 (0.000594%)	
CAAGTCATATTCGACTCCAAAACACTAACCAACCTTCTTCTTGCTTCTCAAAGCTTTCATGGTGTAGCCAAAGTCCATATGAGTCTTTGGCTTTGTGTCT	141 (0.002702%)	
CAAGTCTGGTGCCAGCAGCCGCGGTAATTCCAGCTCCAATAGCGTATATTTAAGTTGTTGCAGTTAAAAAGCTCGTAGTTGAACCTTGGGATGGGTCGGC	27 (0.000517%)	
CAATAGCGTATATTTAAGTTGTTGCAGTTAAAAAGCTCGTAGTTGAACCTTGGGATGGGTCGGCCGGTCCGCCTTTGGTGTGCATTGGTCGGCTTGTCCC	20 (0.000383%)	
CAATCAGCTTCCTTGCGCCTTACGGGTTTACTCACCCGTTGACTCGCACACATGTCAGACTCCTTGGTCCGTGTTTCAAGACGGGTCGAATGGGGAGCCC	157 (0.003009%)	
CAATCATACACATGACATCAAGTCATATTCGACTCCAAAACACTAACCAACCTTCTTCTTGCTTCTCAAAGCTTTCATGGTGTAGCCAAAGTCCATATGA	134 (0.002568%)	
CAATCGTTCCAACTAATCTACCGAAGTACTCGGCTAAGAAGAAAGAAGACGGACGAATCCGAGCCAAAGCCGTACAAGCGCGAGATACCTTCGGGACAGC	4 (0.000077%)	
CACAAACGTTCAATATGACAAACCCATGCCAAGTAAAGAGAAAATGAAAACTGGTGATTGTTGCGGAAATCGTCCAGGATTCCTCGACCAGGACTTGAAA	21 (0.000402%)	
CACAAAGCCAAAGACTCATATGGACTTTGGCTACACCATGAAAGCTTTGAGAAGCAAGAAGAAGGTTGGTTAGTGTTTTGGAGTCGAATATGACTTGATG	37 (0.000709%)	
CACAAAGGGTGTTGGTCGATTAAGACAGCAGGACGGTGGTCATGGAAGTCGAAATCCGCTAAGGAGTGTGTAACAACTCACCTGCCGAATCAACTAGCCC	47 (0.000901%)	
CACAAATCGTCGTCCCTCACCATCCTTTGCTGATGCGGGACGGAAGCTGGTCTCCCGTGTGTTACCGCACGCGGTTGGCCTAAATCCGAGCCAAGGACGC	38 (0.000728%)	
CACACATGTCAGACTCCTTGGTCCGTGTTTCAAGACGGGTCGAATGGGGAGCCCACAGGCCGACGCCCGGAGCACGCTGATGCCGAGGCACGCCGTTAGG	10 (0.000192%)	
CACAGGCCCCGGAACTCATCATCGAGCGTAACATCGCCCGTGAATTAACTGAGAAGGATAGGTGGTAGGTAGTTCGATGCGCGAGCATGGAGCCTACGAA	29 (0.000556%)	
CACATCTGTTAAAAGATAACGCAGGTGTCCTAAGATGAGCTCAACGAGAACAGAAATCTCGTGTGGAACAAAAGGGTAAAAGCTCGTTTGATTCTGATTT	6 (0.000115%)	
CACATGACATCAAGTCATATTCGACTCCAAAACACTAACCAACCTTCTTCTTGCTTCTCAAAGCTTTCATGGTGTAGCCAAAGTCCATATGAGTCTTTGG	33 (0.000632%)	
CACATTTCGTTCATCACCCTTGGCCGGCTATCGAACAGCCGGACTCCCATCAAAAGATGGTTGCCAAGAACATCTTCGTTACGGTTTGCTAATTCTCGGA	7 (0.000134%)	
CACATTTCGTTCATCACCCTTGGCCGGCTTTCGAACAGCCGGACTCCCATCAAAAGATGGTTGCCAAGAACATCTTCGTTACGGTTTGCTAATTCTCGGA	7 (0.000134%)	
CACCATCCTTTGCTGATGCGGGACGGAAGCTGGTCTCCCGTGTGTTACCGCACGCGGTTGGCCTAAATCCGAGCCAAGGACGCCTGGAGCGTACCGACAT	5 (0.000096%)	
CACCCGTTGACTCGCACACATGTCAGACTCCTTGGTCCGTGTTTCAAGACGGGTCGAATGGGGAGCCCACAGGCCGACGCCCGGAGCACGCTGATGCCGA	11 (0.000211%)	
CACCGGACACCACGCGACGTGCGGTGCTCTTCCAGCCGCTGGACCCTACCTCCGGCTGAGCCGTTTCCAGGGTGGGCAGGCTGTTAAACAGAAAAGATAA	7 (0.000134%)	
CACGCCCATCCAACCTAGGCGAGACAAGGGTTCACATTTCGTTCATCACCCTTGGCCGGCTATCGAACAGCCGGACTCCCATCAAAAGATGGTTGCCAAG	20 (0.000383%)	

CACGCGCCTAACGGCGTGCCTCGGCATCAGCGTGCTCCGGGCGTCGGCCTGTGGGCTCCCCATTCGACCCGTCTTGAAACACGGACCAAGGAGTCTGACA	82 (0.001571%)	
CACGCTTTCACGGTTCGTATTCGTACTGAAAATCAGAATCAAACGAGCTTTTACCCTTTTGTTCCACACGAGATTTCTGTTCTCGTTGAGCTCATCTTAG	2071 (0.039690%)	
CACGGTTCGTATTCGTACTGAAAATCAGAATCAAACGAGCTTTTACCCTTTTGTTCCACACGAGATTTCTGTTCTCGTTGAGCTCATCTTAGGACACCTG	5 (0.000096%)	
CACTCGGTCCTCCGGATTTTCAAGGGCCGCCGGGGGCGCACCGGACACCACGCGACGTGCGGTGCTCTTCCAGCCGCTGGACCCTACCTCCGGCTGAGCC	5 (0.000096%)	
CAGAGCAACGGGCAGAGCCCGCGTCGACCTTTTATCTAATAAATGCGTCCCTTCCATAAGTCGGGGTTTGTTGCACGTATTAGCTCTAGAATTACTACGG	17 (0.000326%)	
CAGAGCCCGCGTCGACCTTTTATCTAATAAATGCGTCCCTTCCATAAGTCGGGGTTTGTTGCACGTATTAGCTCTAGAATTACTACGGTTATCCGAGTAG	9 (0.000172%)	
CAGAGCGTAGGCTTGCTTTGAGCACTCTAATTTCTTCAAAGTAACAGCGCCGGAGGCACGACCCGGCCAATTAAGACCAGGAGCGTATCGCCGACCGAAG	38 (0.000728%)	
CAGCACGCGCCTAACGGCGTGCCTCGGCATCAGCGTGCTCCGGGCGTCGGCCTGTGGGCTCCCCATTCGACCCGTCTTGAAACACGGACCAAGGAGTCTG	62 (0.001188%)	
CAGCAGCCGCGGTAATTCCAGCTCCAATAGCGTATATTTAAGTTGTTGCAGTTAAAAAGCTCGTAGTTGAACCTTGGGATGGGTCGGCCGGTCCGCCTTT	19 (0.000364%)	
CAGCAGGACGGTGGTCATGGAAGTCGAAATCCGCTAAGGAGTGTGTAACAACTCACCTGCCGAATCAACTAGCCCCGAAAATGGATGGCGCTTAAGCGCG	8 (0.000153%)	
CAGCAGTTCTCGGACAAAAATTGCTGAGTGGCCGAGAAGAATGGGCGTGTCATGCGTGGGCTGACATGGATTCTTCGAGGCCTAGGGGTGGCGGTATATA	5 (0.000096%)	
CAGCCGCGGTAATTCCAGCTCCAATAGCGTATATTTAAGTTGTTGCAGTTAAAAAGCTCGTAGTTGAACCTTGGGATGGGTCGGCCGGTCCGCCTTTGGT	10 (0.000192%)	
CAGCCTGCTAACTAGCTACGTGGAGGCATCCCTTCACGGCCGGCTTCTTAGAGGGACTATGGCCGTTTAGGCCAAGGAAGTTTGAGGCAATAACAGGTCT	27 (0.000517%)	
CAGCGTGCTCCGGGCGTCGGCCTGTGGGCTCCCCATTCGACCCGTCTTGAAACACGGACCAAGGAGTCTGACATGTGTGCGAGTCAACGGGTGAGTAAAC	5 (0.000096%)	
CAGCTCCAATAGCGTATATTTAAGTTGTTGCAGTTAAAAAGCTCGTAGTTGAACCTTGGGATGGGTCGGCCGGTCCGCCTTTGGTGTGCATTGGTCGGCT	10 (0.000192%)	
CAGCTTCCTTGCGCCTTACGGGTTTACTCACCCGTTGACTCGCACACATGTCAGACTCCTTGGTCCGTGTTTCAAGACGGGTCGAATGGGGAGCCCACAG	114 (0.002185%)	
CAGGACGGTGGTCATGGAAGTCGAAATCCGCTAAGGAGTGTGTAACAACTCACCTGCCGAATCAACTAGCCCCGAAAATGGATGGCGCTTAAGCGCGCGA	28 (0.000537%)	
CAGGGACAGTCGGGGGCATTCGTATTTCATAGTCAGAGGTGAAATTCTTGGATTTATGAAAGACGAACAACTGCGAAAGCATTTGCCAAGGATGTTTTCA	36 (0.000690%)	
CAGGGGTTGAAATCGTCGACCAGGTCCGAGACTTCATCGACCGGGTCCGAGGATTCGTCGACCAGGACGGCCGGATGTCCGAGAAAAAAAAATGTTGCCG	21 (0.000402%)	
CAGGTGGGGAGTTTGGCTGGGGCGGCACATCTGTTAAAAGATAACGCAGGTGTCCTAAGATGAGCTCAACGAGAACAGAAATCTCGTGTGGAACAAAAGG	27 (0.000517%)	
CAGTCGGGGGCATTCGTATTTCATAGTCAGAGGTGAAATTCTTGGATTTATGAAAGACGAACAACTGCGAAAGCATTTGCCAAGGATGTTTTCATTAATC	7 (0.000134%)	
CATACACATGACATCAAGTCATATTCGACTCCAAAACACTAACCAACCTTCTTCTTGCTTCTCAAAGCTTTCATGGTGTAGCCAAAGTCCATATGAGTCT	47 (0.000901%)	
CATATGAGTCTTTGGCTTTGTGTCTTCTAACAAGGATACA	6428 (0.049276%)	
CATATGCTTGTCTCAAAGATTAAGCCATGCATGTGTAAGTATGAACGAATTCAGACTGTGAAACTGCGAATGGCTCATTAAATCAGTTATAGTTTGTTTG	51 (0.000977%)	
CATATTCGACTCCAAAACACTAACCAACCTTCTTCTTGCTTCTCAAAGCTTTCATGGTGTAGCCAAAGTCCATATGAGTCTTTGGCTTTGTGTCTTCTAA	78 (0.001495%)	
CATATTCGACTCCAAAACACTAACCAACCTTCTTCTTGCTTCTCAAAGCTTTCATGGTGTAGCCAAAGTCCATATGAGTCTTTGGCTTTGTGTCTTTTAA	308 (0.005903%)	
CATCAAGTCATATTCGACTCCAAAACACTAACCAACCTTCTTCTTGCTTCTCAAAGCTTTCATGGTGTAGCCAAAGTCCATATGAGTCTTTGGCTTTGTG	62 (0.001188%)	
CATCAATCGTTCCAACTAATCTACCGAAGTACTCGGCTAAGAAGAAAGAAGACGGACGAATCCGAGCCAAAGCCGTACAAGCGCGAGATACCTTCGGGAC	4 (0.000077%)	
CATCAGAGCAACGGGCAGAGCCCGCGTCGACCTTTTATCTAATAAATGCGTCCCTTCCATAAGTCGGGGTTTGTTGCACGTATTAGCTCTAGAATTACTA	48 (0.000920%)	
CATCAGCGTGCTCCGGGCGTCGGCCTGTGGGCTCCCCATTCGACCCGTCTTGAAACACGGACCAAGGAGTCTGACATGTGTGCGAGTCAACGGGTGAGTA	19 (0.000364%)	
CATCATCGAGCGTAACATCGCCCGTGAATTAACTGAGAAGGATAGGTGGTAGGTAGTTCGATGCGCGAGCATGGAGCCTACGAACACTAGCTATCCGATC	13 (0.000249%)	
CATCATTCAAATTTCTGCCCTATCAACTTTCGATGGTAGGATAGTGGCCTACCATGGTGGTAACGGGTGACGGAGAATTAGGGTTCGATTCCGGAGAGGG	11 (0.000211%)	
CATCCAACCTAGGCGAGACAAGGGTTCACATTTCGTTCATCACCCTTGGCCGGCTTTCGAACAGCCGGACTCCCATCAAAAGATGGTTGCCAAGAACATC	4 (0.000077%)	
CATCCTTTGCTGATGCGGGACGGAAGCTGGTCTCCCGTGTGTTACCGCACGCGGTTGGCCTAAATCCGAGCCAAGGACGCCTGGAGCGTACCGACATGCG	131 (0.002511%)	
CATCGAACTACCTACCACCTATCCTTCTCAGTTAATTCACGGGCGATGTTACGCTCGATGATGAGTTCCGGGGCCTGTGTTTCGTACCTAATTTGAAGGA	7 (0.000134%)	
CATCTGTTAAAAGATAACGCAGGTGTCCTAAGATGAGCTCAACGAGAACAGAAATCTCGTGTGGAACAAAAGGGTAAAAGCTCGTTTGATTCTGATTTTC	4 (0.000077%)	
CATGAATCATCAGAGCAACGGGCAGAGCCCGCGTCGACCTTTTATCTAATAAATGCGTCCCTTCCATAAGTCGGGGTTTGTTGCACGTATTAGCTCTAGA	173 (0.003315%)	
CATGACATCAAGTCATATTCGACTCCAAAACACTAACCAACCTTCTTCTTGCTTCTCAAAGCTTTCATGGTGTAGCCAAAGTCCATATGAGTCTTTGGCT	21 (0.000402%)	
CATGGAAGTCGAAATCCGCTAAGGAGTGTGTAACAACTCACCTGCCGAATCAACTAGCCCCGAAAATGGATGGCGCTTAAGCGCGCGACCTATACCCGGC	58 (0.001112%)	
CATGGCCTCTGTGCTGGCGACGCATCATTCAAATTTCTGCCCTATCAACTTTCGATGGTAGGATAGTGGCCTACCATGGTGGTAACGGGTGACGGAGAAT	258 (0.004944%)	
CATGGGCATCGACACCTTGCGGCTAGGAACTGGAACGAGACGGGTGGCAAAGATTTCGAGTAGCACTTCATACTACCGTGGGTTTTTTAAACCTTCCGAG	9 (0.000172%)	
CATGTGTATGATTGAGTATAAGAACTTAAACCGCAACCGCATCTTATAAGCCTAAGTAGTGTTTCCTTGTTAGAAGACACAAAGCCAAAGACTCATATGG	12 (0.000230%)	
CATTGTCAGGTGGGGAGTTTGGCTGGGGCGGCACATCTGTTAAAAGATAACGCAGGTGTCCTAAGATGAGCTCAACGAGAACAGAAATCTCGTGTGGAAC	22 (0.000422%)	

CATTGTTCCATCGACCAGAGGCTGTTCACCTTGGAGACCTGATGCGGTTATGAGTACGACCGGGCGTGAGC<br>GGCACTCGGTCCTCCGGATTTTCAAGGGC	20 (0.000383%)	
CATTTTATCGGTCGCTCTTGTCCGGAAGCTGTAGATGACCCAAAGTCCATATAGCGACCCCAGGTCAGGCG<br>GGATTACCCGCTGAGTTTAAGCATATCAA	4 (0.000077%)	
CCAAAGACTCATATGGACTTTGGCTACACCATGAAAGCTTTGAGAAGCAAGAAGAAGGTTGGTTAGTGTTT<br>TGGAGTCGAATATGACTTGATGTCATGTG	143 (0.002741%)	
CCAACCTAGGCGAGACAAGGGTTCACATTTCGTTCATCACCCTTGGCCGGCTATCGAACAGCCGGACTCCC<br>ATCAAAAGATGGTTGCCAAGAACATCTTC	18 (0.000345%)	
CCAACGAAGCACGCCCATCCAACCTAGGCGAGACAAGGGTTCACATTTCGTTCATCACCCTTGGCCGGCTA<br>TCGAACAGCCGGACTCCCATCAAAAGATG	11 (0.000211%)	
CCAATAGCGTATATTTAAGTTGTTGCAGTTAAAAAGCTCGTAGTTGAACCTTGGGATGGGTCGGCCGGTCC<br>GCCTTTGGTGTGCATTGGTCGGCTTGTCC	3 (0.000057%)	
CCAATCAGCTTCCTTGCGCCTTACGGGTTTACTCACCCGTTGACTCGCACACATGTCAGACTCCTTGGTCC<br>GTGTTTCAAGACGGGTCGAATGGGGAGCC	18 (0.000345%)	
CCACAAAGGGTGTTGGTCGATTAAGACAGCAGGACGGTGGTCATGGAAGTCGAAATCCGCTAAGGAGTGTG<br>TAACAACTCACCTGCCGAATCAACTAGCC	7 (0.000134%)	
CCAGAGCGTAGGCTTGCTTTGAGCACTCTAATTTCTTCAAAGTAACAGCGCCGGAGGCACGACCCGGCCAA<br>TTAAGACCAGGAGCGTATCGCCGACCGAA	28 (0.000537%)	
CCAGCAGCCGCGGTAATTCCAGCTCCAATAGCGTATATTTAAGTTGTTGCAGTTAAAAAGCTCGTAGTTGA<br>ACCTTGGGATGGGTCGGCCGGTCCGCCTT	10 (0.000192%)	
CCAGCTCCAATAGCGTATATTTAAGTTGTTGCAGTTAAAAAGCTCGTAGTTGAACCTTGGGATGGGTCGGC<br>CGGTCCGCCTTTGGTGTGCATTGGTCGGC	8 (0.000153%)	
CCATCCAACCTAGGCGAGACAAGGGTTCACATTTCGTTCATCACCCTTGGCCGGCTTTCGAACAGCCGGAC<br>TCCCATCAAAAGATGGTTGCCAAGAACAT	6 (0.000115%)	
CCATCCTTTGCTGATGCGGGACGGAAGCTGGTCTCCCGTGTGTTACCGCACGCGGTTGGCCTAAATCCGAG<br>CCAAGGACGCCTGGAGCGTACCGACATGC	8 (0.000153%)	
CCATGCTAATGTATCCAGAGCGTAGGCTTGCTTTGAGCACTCTAATTTCTTCAAAGTAACAGCGCCGGAGG<br>CACGACCCGGCCAATTAAGACCAGGAGCG	8 (0.000153%)	
CCATGCTCGCGCATCGAACTACCTACCACCTATCCTTCTCAGTTAATTCACGGGCGATGTTACGCTCGATG<br>ATGAGTTCCGGGGCCTGTGTTTCGTACCT	4 (0.000077%)	
CCCACAAAGGGTGTTGGTCGATTAAGACAGCAGGACGGTGGTCATGGAAGTCGAAATCCGCTAAGGAGTGT<br>GTAACAACTCACCTGCCGAATCAACTAGC	9 (0.000172%)	
CCCATCCAACCTAGGCGAGACAAGGGTTCACATTTCGTTCATCACCCTTGGCCGGCTATCGAACAGCCGGA<br>CTCCCATCAAAAGATGGTTGCCAAGAACA	8 (0.000153%)	
CCCATCCAACCTAGGCGAGACAAGGGTTCACATTTCGTTCATCACCCTTGGCCGGCTTTCGAACAGCCGGA<br>CTCCCATCAAAAGATGGTTGCCAAGAACA	11 (0.000211%)	
CCCATGCTAATGTATCCAGAGCGTAGGCTTGCTTTGAGCACTCTAATTTCTTCAAAGTAACAGCGCCGGAG<br>GCACGACCCGGCCAATTAAGACCAGGAGC	10 (0.000192%)	
CCCATGGGCATCGACACCTTGCGGCTAGGAACTGGAACGAGACGGGTGGCAAAGATTTCGAGTAGCACTTC<br>ATACTACCGTGGGTTTTTTAAACCTTCCG	13 (0.000249%)	
CCCGAAGGTATCTCGCGCTTGTACGGCTTTGGCTCGGATTCGTCCGTCTTCTTTCTTCTTAGCCGAGTACT<br>TCGGTAGATTAGTTGGAACGATTGATGAT	53 (0.001016%)	
CCCGAGTGTGAGCGAGGTGTGAGTGTCGCCCATGGGCATCGACACCTTGCGGCTAGGAACTGGAACGAGAC<br>GGGTAGCAAAGATTTCGAGTAGCACTTCA	9 (0.000172%)	
CCCGAGTGTGAGCGAGGTGTGAGTGTCGCCCATGGGCATCGACACCTTGCGGCTAGGAACTGGAACGAGAC<br>GGGTGGCAAAGATTTCGAGTAGCACTTCA	21 (0.000402%)	
CCCGCCAATCAGCTTCCTTGCGCCTTACGGGTTTACTCACCCGTTGACTCGCACACATGTCAGACTCCTTG<br>GTCCGTGTTTCAAGACGGGTCGAATGGGG	8 (0.000153%)	
CCCGCCGAAGCGAGCCTTGGGACCAAAAACAGGGGTTGTACCCCGCCTCCGATTCACGGAGTAAGTAAAAT<br>AACGTTAAAAGTAGTGGTATTTCACTTGC	8 (0.000153%)	
CCCGCGTCGACCTTTTATCTAATAAATGCGTCCCTTCCATAAGTCGGGGTTTGTTGCACGTATTAGCTCTA<br>GAATTACTACGGTTATCCGAGTAGTAGTT	38 (0.000728%)	
CCCGTTGACTCGCACACATGTCAGACTCCTTGGTCCGTGTTTCAAGACGGGTCGAATGGGGAGCCCACAGG<br>CCGACGCCCGGAGCACGCTGATGCCGAGG	3 (0.000057%)	
CCCTCACCATCCTTTGCTGATGCGGGACGGAAGCTGGTCTCCCGTGTGTTACCGCACGCGGTTGGCCTAAA<br>TCCGAGCCAAGGACGCCTGGAGCGTACCG	14 (0.000268%)	
CCCTTGCCTACATTGTTCCATCGACCAGAGGCTGTTCACCTTGGAGACCTGATGCGGTTATGAGTACGACC<br>GGGCGTGAGCGGCACTCGGTCCTCCGGAT	21 (0.000402%)	
CCGAAGCGAGCCTTGGGACCAAAAACAGGGGTTGTACCCCGCCTCCGATTCACGGAGTAAGTAAAATAACG<br>TTAAAAGTAGTGGTATTTCACTTGCGCCG	8 (0.000153%)	
CCGAAGGTATCTCGCGCTTGTACGGCTTTGGCTCGGATTCGTCCGTCTTCTTTCTTCTTAGCCGAGTACTT<br>CGGTAGATTAGTTGGAACGATTGATGATT	7 (0.000134%)	
CCGACTTCCCTTGCCTACATTGTTCCATCGACCAGAGGCTGTTCACCTTGGAGACCTGATGCGGTTATGAG<br>TACGACCGGGCGTGAGCGGCACTCGGTCC	50 (0.000958%)	
CCGAGTGTGAGCGAGGTGTGAGTGTCGCCCATGGGCATCGACACCTTGCGGCTAGGAACTGGAACGAGACG<br>GGTAGCAAAGATTTCGAGTAGCACTTCAT	11 (0.000211%)	
CCGAGTGTGAGCGAGGTGTGAGTGTCGCCCATGGGCATCGACACCTTGCGGCTAGGAACTGGAACGAGACG<br>GGTGGCAAAGATTTCGAGTAGCACTTCAT	19 (0.000364%)	
CCGCCAATCAGCTTCCTTGCGCCTTACGGGTTTACTCACCCGTTGACTCGCACACATGTCAGACTCCTTGG<br>TCCGTGTTTCAAGACGGGTCGAATGGGGA	49 (0.000939%)	
CCGCCGGGGGCGCACCGGACACCACGCGACGTGCGGTGCTCTTCCAGCCGCTGGACCCTACCTCCGGCTGA<br>GCCGTTTCCAGGGTGGGCAGGCTGTTAAA	6 (0.000115%)	
CCGCCTAGGCTGTCCCGAGTGTGAGCGAGGTGTGAGTGTCGCCCATGGGCATCGACACCTTGCGGCTAGGA<br>ACTGGAACGAGACGGGTGGCAAAGATTTC	92 (0.001763%)	
CCGCGGTAATTCCAGCTCCAATAGCGTATATTTAAGTTGTTGCAGTTAAAAAGCTCGTAGTTGAACCTTGG<br>GATGGGTCGGCCGGTCCGCCTTTGGTGTG	6 (0.000115%)	
CCGGAACCGGGACGTGGCGGTTGACGGCAACGTTAGGGAGTCCGGAGACGTCGGCGGGGGCCTCGGGAAGA<br>GTTATCTTTTCTGTTTAACAGCCTGCCCA	107 (0.002051%)	
CCGGACACCACGCGACGTGCGGTGCTCTTCCAGCCGCTGGACCCTACCTCCGGCTGAGCCGTTTCCAGGGT<br>GGGCAGGCTGTTAAACAGAAAAGATAACT	22 (0.000422%)	
CCGGAGACGTCGGCGGGGGCCTCGGGAAGAGTTATCTTTTCTGTTTAACAGCCTGCCCACCCTGGAAACGG<br>CTCAGCCGGAGGTAGGGTCCAGCGGCTGG	8 (0.000153%)	
CCGGATTTTCAAGGGCCGCCGGGGGCGCACCGGACACCACGCGACGTGCGGTGCTCTTCCAGCCGCTGGAC<br>CCTACCTCCGGCTGAGCCGTTTCCAGGGT	8 (0.000153%)	
CCGGGACGTGGCGGTTGACGGCAACGTTAGGGAGTCCGGAGACGTCGGCGGGGGCCTCGGGAAGAGTTATC<br>TTTTCTGTTTAACAGCCTGCCCACCCTGG	21 (0.000402%)	

CCGGGCGTCGGCCTGTGGGCTCCCCATTCGACCCGTCTTGAAACACGGACCAAGGAGTCTGACATGTGTGCGAGTCAACGGGTGAGTAAACCCGTAAGGC	7 (0.000134%)	
CCGGGGGCGCACCGGACACCACGCGACGTGCGGTGCTCTTCCAGCCGCTGGACCCTACCTCCGGCTGAGCCGTTTCCAGGGTGGGCAGGCTGTTAAACAG	7 (0.000134%)	
CCGTCGAGTTATCATGAATCATCAGAGCAACGGGCAGAGCCCGCGTCGACCTTTTATCTAATAAATGCGTCCCTTCCATAAGTCGGGGTTTGTTGCACGT	18 (0.000345%)	
CCGTTGACTCGCACACATGTCAGACTCCTTGGTCCGTGTTTCAAGACGGGTCGAATGGGGAGCCCACAGGCCGACGCCCGGAGCACGCTGATGCCGAGGC	4 (0.000077%)	
CCTAACGGCGTGCCTCGGCATCAGCGTGCTCCGGGCGTCGGCCTGTGGGCTCCCCATTCGACCCGTCTTGAAACACGGACCAAGGAGTCTGACATGTGTG	7 (0.000134%)	
CCTAAGTAGTGTTTCCTTGTTAGAAGACACAAAGCCAAAGACTCATATGGACTTTGGCTACACCATGAAAGCTTTGAGAAGCAAGAAGAAGGTTGGTTAG	67 (0.001284%)	
CCTACATTGTTCCATCGACCAGAGGCTGTTCACCTTGGAGACCTGATGCGGTTATGAGTACGACCGGGCGTGAGCGGCACTCGGTCCTCCGGATTTTCAA	8 (0.000153%)	
CCTAGGCGAGACAAGGGTTCACATTTCGTTCATCACCCTTGGCCGGCTATCGAACAGCCGGACTCCCATCAAAAGATGGTTGCCAAGAACATCTTCGTTA	10 (0.000192%)	
CCTAGGCTGTCCCGAGTGTGAGCGAGGTGTGAGTGTCGCCCATGGGCATCGACACCTTGCGGCTAGGAACTGGAACGAGACGGGTGGCAAAGATTTCGAG	58 (0.001112%)	
CCTATCAACTTTCGATGGTAGGATAGTGGCCTACCATGGTGGTAACGGGTGACGGAGAATTAGGGTTCGATTCCGGAGAGGGAGCCTGAGAAACGGCTAC	4 (0.000077%)	
CCTATGATGTTATCCCATGCTAATGTATCCAGAGCGTAGGCTTGCTTTGAGCACTCTAATTTCTTCAAAGTAACAGCGCCGGAGGCACGACCCGGCCAAT	24 (0.000460%)	
CCTCACCATCCTTTGCTGATGCGGGACGGAAGCTGGTCTCCCGTGTGTTACCGCACGCGGTTGGCCTAAATCCGAGCCAAGGACGCCTGGAGCGTACCGA	66 (0.001265%)	
CCTCAGCCTGCTAACTAGCTACGTGGAGGCATCCCTTCACGGCCGGCTTCTTAGAGGGACTATGGCCGTTTAGGCCAAGGAAGTTTGAGGCAATAACAGG	107 (0.002051%)	
CCTCGGCATCAGCGTGCTCCGGGCGTCGGCCTGTGGGCTCCCCATTCGACCCGTCTTGAAACACGGACCAAGGAGTCTGACATGTGTGCGAGTCAACGGG	5 (0.000096%)	
CCTCTGTGCTGGCGACGCATCATTCAAATTTCTGCCCTATCAACTTTCGATGGTAGGATAGTGGCCTACCATGGTGGTAACGGGTGACGGAGAATTAGGG	93 (0.001782%)	
CCTGCTAACTAGCTACGTGGAGGCATCCCTTCACGGCCGGCTTCTTAGAGGGACTATGGCCGTTTAGGCCAAGGAAGTTTGAGGCAATAACAGGTCTGTG	6 (0.000115%)	
CCTTGCCTACATTGTTCCATCGACCAGAGGCTGTTCACCTTGGAGACCTGATGCGGTTATGAGTACGACCGGGCGTGAGCGGCACTCGGTCCTCCGGATT	14 (0.000268%)	
CCTTGCGCCTTACGGGTTTACTCACCCGTTGACTCGCACACATGTCAGACTCCTTGGTCCGTGTTTCAAGACGGGTCGAATGGGGAGCCCACAGGCCGAC	3 (0.000057%)	
CCTTGTTAGAAGACACAAAGCCAAAGACTCATATGGACTTTGGCTACACCATGAAAGCTTTGAGAAGCAAGAAGAAGGTTGGTTAGTGTTTTGGAGTCGA	50 (0.000958%)	
CGAAACACAGGCCCCGGAACTCATCATCGAGCGTAACATCGCCCGTGAATTAACTGAGAAGGATAGGTGGTAGGTAGTTCGATGCGCGAGCATGGAGCCT	29 (0.000556%)	
CGAAATCCTATGATGTTATCCCATGCTAATGTATCCAGAGCGTAGGCTTGCTTTGAGCACTCTAATTTCTTCAAAGTAACAGCGCCGGAGGCACGACCCG	457 (0.008758%)	
CGAACTACCTACCACCTATCCTTCTCAGTTAATTCACGGGCGATGTTACGCTCGATGATGAGTTCCGGGGCCTGTGTTTCGTACCTAATTTGAAGGAATT	6 (0.000115%)	
CGAAGCACGCCCATCCAACCTAGGCGAGACAAGGGTTCACATTTCGTTCATCACCCTTGGCCGGCTATCGAACAGCCGGACTCCCATCAAAAGATGGTTG	18 (0.000345%)	
CGAAGGTATCTCGCGCTTGTACGGCTTTGGCTCGGATTCGTCCGTCTTCTTTCTTCTTAGCCGAGTACTTCGGTAGATTAGTTGGAACGATTGATGATTT	19 (0.000364%)	
CGACCAGGGGTTGAAATCGTCGACCAGGTCCGAGACTTCATCGACCGGGTCCGAGGATTCGTCGACCAGGACGGCCGGATGTCCGAGAAAAAAAAATGTT	28 (0.000537%)	
CGACCCGCCGAAGCGAGCCTTGGGACCAAAAACAGGGGTTGTACCCCGCCTCCGATTCACGGAGTAAGTAAAATAACGTTAAAAGTAGTGGTATTTCACT	5 (0.000096%)	
CGACGCATCATTCAAATTTCTGCCCTATCAACTTTCGATGGTAGGATAGTGGCCTACCATGGTGGTAACGGGTGACGGAGAATTAGGGTTCGATTCCGGA	8 (0.000153%)	
CGACGGGCGGTGTGTACAAAGGGCAGGGACGTAGTCAACGCGAGCTGATGACTCGCGCTTACTAGGAATTCCTCGTTGAAGACCAACAATTGCAATGATC	27 (0.000517%)	
CGACGTGGGTGGTTCGCCGCCCGCGACGTCGCGAGAAGTCCACTAAACCTTATCATTTAGAGGAAGGAGAAGTCGTAACAAGGTTTCCGTAGGTGAACCT	115 (0.002204%)	
CGACTCCAAAACACTAACCAACCTTCTTCTTGCTTCTCAAAGCTTTCATGGTGTAGCCAAAGTCCATATGAGTCTTTGGCTTTGTGTCTTCTAACAAGGA	57 (0.001092%)	
CGACTCCAAAACACTAACCAACCTTCTTCTTGCTTCTCAAAGCTTTCATGGTGTAGCCAAAGTCCATATGAGTCTTTGGCTTTGTGTCTTTTAACAAGGA	38 (0.000728%)	
CGACTTCCCTTGCCTACATTGTTCCATCGACCAGAGGCTGTTCACCTTGGAGACCTGATGCGGTTATGAGTACGACCGGGCGTGAGCGGCACTCGGTCCT	25 (0.000479%)	
CGAGACAAGGGTTCACATTTCGTTCATCACCCTTGGCCGGCTATCGAACAGCCGGACTCCCATCAAAAGATGGTTGCCAAGAACATCTTCGTTACGGTTT	3 (0.000057%)	
CGAGACAAGGGTTCACATTTCGTTCATCACCCTTGGCCGGCTTTCGAACAGCCGGACTCCCATCAAAAGATGGTTGCCAAGAACATCTTCGTTACGGTTT	9 (0.000172%)	
CGAGCTCGTGTAAAGTTGGGAATTCGTTAAGGAGCTGTTGCTTTGTTAGTGTAGAAACACTTGTGTAGAATTGGGGATTGTTTTTTTTGGAGTGATTTAG	753 (0.014431%)	
CGAGGTGTGAGTGTCGCCCATGGGCATCGACACCTTGCGGCTAGGAACTGGAACGAGACGGGTGGCAAAGATTTCGAGTAGCACTTCATACTACCGTGGG	24 (0.000460%)	
CGAGTGTGAGCGAGGTGTGAGTGTCGCCCATGGGCATCGACACCTTGCGGCTAGGAACTGGAACGAGACGGGTAGCAAAGATTTCGAGTAGCACTTCATA	20 (0.000383%)	
CGAGTGTGAGCGAGGTGTGAGTGTCGCCCATGGGCATCGACACCTTGCGGCTAGGAACTGGAACGAGACGGGTGGCAAAGATTTCGAGTAGCACTTCATA	32 (0.000613%)	
CGAGTTATCATGAATCATCAGAGCAACGGGCAGAGCCCGCGTCGACCTTTTATCTAATAAATGCGTCCCTTCCATAAGTCGGGGTTTGTTGCACGTATTA	143 (0.002741%)	
CGATCCATTACATTTTATCGGTCGCTCTTGTCCGGAAGCTGTAGATGACCCAAAGTCCATATAGCGACCCCAGGTCAGGCGGGATTACCCGCTGAGTTTA	5 (0.000096%)	
CGATCCCGCCAATCAGCTTCCTTGCGCCTTACGGGTTTACTCACCCGTTGACTCGCACACATGTCAGACTCCTTGGTCCGTGTTTCAAGACGGGTCGAAT	6 (0.000115%)	
CGATCCGTCGAGTTATCATGAATCATCAGAGCAACGGGCAGAGCCCGCGTCGACCTTTTATCTAATAAATGCGTCCCTTCCATAAGTCGGGGTTTGTTGC	177 (0.003392%)	
CGATCGACCCGCCGAAGCGAGCCTTGGGACCAAAAACAGGGGTTGTACCCCGCCTCCGATTCACGGAGTAAGTAAAATAACGTTAAAAGTAGTGGTATTT	7 (0.000134%)	

CGATTAAGACAGCAGGACGGTGGTCATGGAAGTCGAAATCCGCTAAGGAGTGTGTAACAACTCACCTGCCGAATCAACTAGCCCCGAAAATGGATGGCGC	12 (0.000230%)
CGATTAGTCTTTCGCCCCTATACCCAAGTCAGACGAACGATTTGCACGTCAGTATCGCTGCGGGCTTCCACCAGAGTTTCCTCTGGCTTTACCCCGCTCA	12 (0.000230%)
CGATTTGCACGTCAGTATCGCTGCGGGCTTCCACCAGAGTTTCCTCTGGCTTTACCCCGCTCAGGCATAGTTCACCATCTTTCGGGTCCCGACAGGCATG	485 (0.009295%)
CGCACACATGTCAGACTCCTTGGTCCGTGTTTCAAGACGGGTCGAATGGGGAGCCCACAGGCCGACGCCCGGAGCACGCTGATGCCGAGGCACGCCGTTA	19 (0.000364%)
CGCACCGGACACCACGCGACGTGCGGTGCTCTTCCAGCCGCTGGACCCTACCTCCGGCTGAGCCGTTTCCAGGGTGGGCAGGCTGTTAAACAGAAAAGAT	8 (0.000153%)
CGCATCATTCAAATTTCTGCCCTATCAACTTTCGATGGTAGGATAGTGGCCTACCATGGTGGTAACGGGTGACGGAGAATTAGGGTTCGATTCCGGAGAG	5 (0.000096%)
CGCCAATCAGCTTCCTTGCGCCTTACGGGTTTACTCACCCGTTGACTCGCACACATGTCAGACTCCTTGGTCCGTGTTTCAAGACGGGTCGAATGGGGAG	1227 (0.023515%)
CGCCCATCCAACCTAGGCGAGACAAGGGTTCACATTTCGTTCATCACCCTTGGCCGGCTATCGAACAGCCGGACTCCCATCAAAAGATGGTTGCCAAGAA	85 (0.001629%)
CGCCCATCCAACCTAGGCGAGACAAGGGTTCACATTTCGTTCATCACCCTTGGCCGGCTTTCGAACAGCCGGACTCCCATCAAAAGATGGTTGCCAAGAA	83 (0.001591%)
CGCCCATGGGCATCGACACCTTGCGGCTAGGAACTGGAACGAGACGGGTGGCAAAGATTTCGAGTAGCACTTCATACTACCGTGGGTTTTTTAAACCTTC	31 (0.000594%)
CGCCGAAGCGAGCCTTGGGACCAAAAACAGGGGTTGTACCCCGCCTCCGATTCACGGAGTAAGTAAAATAACGTTAAAAGTAGTGGTATTTCACTTGCGC	6 (0.000115%)
CGCCGCCCGCGACGTCGCGAGAAGTCCACTAAACCTTATCATTTAGAGGAAGGAGAAGTCGTAACAAGGTTTCCGTAGGTGAACCTGCGGAAGGATCGAT	12 (0.000230%)
CGCCGGGGGCGCACCGGACACCACGCGACGTGCGGTGCTCTTCCAGCCGCTGGACCCTACCTCCGGCTGAGCCGTTTCCAGGGTGGGCAGGCTGTTAAAC	9 (0.000172%)
CGCCTAACGGCGTGCCTCGGCATCAGCGTGCTCCGGGCGTCGGCCTGTGGGCTCCCCATTCGACCCGTCTTGAAACACGGACCAAGGAGTCTGACATGTG	39 (0.000747%)
CGCCTAGGCTGTCCCGAGTGTGAGCGAGGTGTGAGTGTCGCCCATGGGCATCGACACCTTGCGGCTAGGAACTGGAACGAGACGGGTGGCAAAGATTTCG	148 (0.002836%)
CGCCTTACGGGTTTACTCACCCGTTGACTCGCACACATGTCAGACTCCTTGGTCCGTGTTTCAAGACGGGTCGAATGGGGAGCCCACAGGCCGACGCCCG	9 (0.000172%)
CGCGCATCGAACTACCTACCACCTATCCTTCTCAGTTAATTCACGGGCGATGTTACGCTCGATGATGAGTTCCGGGGCCTGTGTTTCGTACCTAATTTGA	14 (0.000268%)
CGCGCCTAACGGCGTGCCTCGGCATCAGCGTGCTCCGGGCGTCGGCCTGTGGGCTCCCCATTCGACCCGTCTTGAAACACGGACCAAGGAGTCTGACATG	27 (0.000517%)
CGCGCTTGTACGGCTTTGGCTCGGATTCGTCCGTCTTCTTTCTTCTTAGCCGAGTACTTCGGTAGATTAGTTGGAACGATTGATGATTTTGAGTTAATTG	25 (0.000479%)
CGCGGTAATTCCAGCTCCAATAGCGTATATTTAAGTTGTTGCAGTTAAAAAGCTCGTAGTTGAACCTTGGGATGGGTCGGCCGGTCCGCCTTTGGTGTGC	5 (0.000096%)
CGCTTGTACGGCTTTGGCTCGGATTCGTCCGTCTTCTTTCTTCTTAGCCGAGTACTTCGGTAGATTAGTTGGAACGATTGATGATTTTGAGTTAATTGAA	6 (0.000115%)
CGCTTTCACGGTTCGTATTCGTACTGAAAATCAGAATCAAACGAGCTTTTACCCTTTTGTTCCACACGAGATTTCTGTTCTCGTTGAGCTCATCTTAGGA	7 (0.000134%)
CGGAACCGGGACGTGGCGGTTGACGGCAACGTTAGGGAGTCCGGAGACGTCGGCGGGGGCCTCGGGAAGAGTTATCTTTTCTGTTTAACAGCCTGCCCAC	83 (0.001591%)
CGGACACCACGCGACGTGCGGTGCTCTTCCAGCCGCTGGACCCTACCTCCGGCTGAGCCGTTTCCAGGGTGGGCAGGCTGTTAAACAGAAAAGATAACTC	58 (0.001112%)
CGGAGACGTCGGCGGGGGCCTCGGGAAGAGTTATCTTTTCTGTTTAACAGCCTGCCCACCCTGGAAACGGCTCAGCCGGAGGTAGGGTCCAGCGGCTGGA	41 (0.000786%)
CGGAGGACATTGTCAGGTGGGGAGTTTGGCTGGGGCGGCACATCTGTTAAAAGATAACGCAGGTGTCCTAAGATGAGCTCAACGAGAACAGAAATCTCGT	45 (0.000862%)
CGGAGTTTTTTCAGCAGTTCTCGGACAAAAATTGCTGAGTGGCCGAGAAGAATGGGCGTGTCATGCGTGGGCTGACATGGATTCTTCGAGGCCTAGGGGT	3 (0.000057%)
CGGATAGCTAGTGTTCGTAGGCTCCATGCTCGCGCATCGAACTACCTACCACCTATCCTTCTCAGTTAATTCACGGGCGATGTTACGCTCGATGATGAGT	695 (0.013319%)
CGGATTTTCAAGGGCCGCCGGGGGCGCACCGGACACCACGCGACGTGCGGTGCTCTTCCAGCCGCTGGACCCTACCTCCGGCTGAGCCGTTTCCAGGGTG	40 (0.000767%)
CGGCAACGTTAGGGAGTCCGGAGACGTCGGCGGGGGCCTCGGGAAGAGTTATCTTTTCTGTTTAACAGCCTGCCCACCCTGGAAACGGCTCAGCCGGAGG	24 (0.000460%)
CGGCAATTCCCCGCCACATCCTCTCAAACGCAATGGAAAGAGAGAAAGGACGAGGTCTTGACCGTCATCTTTTGCCCGAAGGACGGATGAGCTTTGGCGG	1185 (0.022710%)
CGGCACATCTGTTAAAAGATAACGCAGGTGTCCTAAGATGAGCTCAACGAGAACAGAAATCTCGTGTGGAACAAAAGGGTAAAAGCTCGTTTGATTCTGA	10 (0.000192%)
CGGCACTCGGTCCTCCGGATTTTCAAGGGCCGCCGGGGGCGCACCGGACACCACGCGACGTGCGGTGCTCTTCCAGCCGCTGGACCCTACCTCCGGCTGA	50 (0.000958%)
CGGCATCAGCGTGCTCCGGGCGTCGGCCTGTGGGCTCCCCATTCGACCCGTCTTGAAACACGGACCAAGGAGTCTGACATGTGTGCGAGTCAACGGGTGA	10 (0.000192%)
CGGCCTGTGGGCTCCCCATTCGACCCGTCTTGAAACACGGACCAAGGAGTCTGACATGTGTGCGAGTCAACGGGTGAGTAAACCCGTAAGGCGCAAGGAA	8 (0.000153%)
CGGCGACGTGGGTGGTTCGCCGCCCGCGACGTCGCGAGAAGTCCACTAAACCTTATCATTTAGAGGAAGGAGAAGTCGTAACAAGGTTTCCGTAGGTGAA	502 (0.009621%)
CGGCGTGCCTCGGCATCAGCGTGCTCCGGGCGTCGGCCTGTGGGCTCCCCATTCGACCCGTCTTGAAACACGGACCAAGGAGTCTGACATGTGTGCGAGT	5 (0.000096%)
CGGCTTTGGCTCGGATTCGTCCGTCTTCTTTCTTCTTAGCCGAGTACTTCGGTAGATTAGTTGGAACGATTGATGATTTTGAGTTAATTGAACGTTCGGC	3 (0.000057%)
CGGGACGTGGCGGTTGACGGCAACGTTAGGGAGTCCGGAGACGTCGGCGGGGGCCTCGGGAAGAGTTATCTTTTCTGTTTAACAGCCTGCCCACCCTGGA	43 (0.000824%)
CGGGCAGAGCCCGCGTCGACCTTTTATCTAATAAATGCGTCCCTTCCATAAGTCGGGGTTTGTTGCACGTATTAGCTCTAGAATTACTACGGTTATCCGA	50 (0.000958%)
CGGGCGAGAGACCGATAGCGAACAAGTACCGCGAGGTAAAGATGAAAAGGACTTTGAAAAGAGAGTCAAAGAGTGCTTGAAATTGTCGGGAGGGAAGCGG	3 (0.000057%)
CGGGCGGTGTGTACAAAGGGCAGGGACGTAGTCAACGCGAGCTGATGACTCGCGCTTACTAGGAATTCCTCGTTGAAGACCAACAATTGCAATGATCGAT	37 (0.000709%)
CGGGCGTCGGCCTGTGGGCTCCCCATTCGACCCGTCTTGAAACACGGACCAAGGAGTCTGACATGTGTGCGAGTCAACGGGTGAGTAAACCCGTAAGGCG	46 (0.000882%)

CGGGGGCATTCGTATTTCATAGTCAGAGGTGAAATTCTTGGATTTATGAAAGACGAACAACTGCGAAAGCA<br>TTTGCCAAGGATGTTTTCATTAATCAAGA	29 (0.000556%)	
CGGGGGCGCACCGGACACCACGCGACGTGCGGTGCTCTTCCAGCCGCTGGACCCTACCTCCGGCTGAGCCG<br>TTTCCAGGGTGGGCAGGCTGTTAAACAGA	31 (0.000594%)	
CGGGTTTACTCACCCGTTGACTCGCACACATGTCAGACTCCTTGGTCCGTGTTTCAAGACGGGTCGAATGG<br>GGAGCCCACAGGCCGACGCCCGGAGCACG	17 (0.000326%)	
CGGTAATTCCAGCTCCAATAGCGTATATTTAAGTTGTTGCAGTTAAAAAGCTCGTAGTTGAACCTTGGGAT<br>GGGTCGGCCGGTCCGCCTTTGGTGTGCAT	15 (0.000287%)	
CGGTAGGAGCGACGGGCGGTGTGTACAAAGGGCAGGGACGTAGTCAACGCGAGCTGATGACTCGCGCTTAC<br>TAGGAATTCCTCGTTGAAGACCAACAATT	52 (0.000997%)	
CGGTCCTCCGGATTTTCAAGGGCCGCCGGGGGCGCACCGGACACCACGCGACGTGCGGTGCTCTTCCAGCC<br>GCTGGACCCTACCTCCGGCTGAGCCGTTT	8 (0.000153%)	
CGGTGGTCATGGAAGTCGAAATCCGCTAAGGAGTGTGTAACAACTCACCTGCCGAATCAACTAGCCCCGAA<br>AATGGATGGCGCTTAAGCGCGCGACCTAT	33 (0.000632%)	
CGGTTGACGGCAACGTTAGGGAGTCCGGAGACGTCGGCGGGGGCCTCGGGAAGAGTTATCTTTTCTGTTTA<br>ACAGCCTGCCCACCCTGGAAACGGCTCAG	5 (0.000096%)	
CGGTTTAAGTTCTTATACTCAATCATACACATGACATCAAGTCATATTCGACTCCAAAACACTAACCAACC<br>TTCTTCTTGCTTCTCAAAGCTTTCATGGT	87 (0.001667%)	
CGGTTTAAGTTGTTATACTCAATCATACACATGACAACAAGTCATATTCGACTCCAAAACACTAACCAACC<br>TTCTTCTTGCTTCTCAAAGCTTTCATGGT	97 (0.001859%)	
CGTAAGAATTGTATCCTTGTTAAAAGACACAAAGCCAAAGACTCATATGGACTTTGGCTACACCATGAAAG<br>CTTTGAGAAGCAAGAAGAAGGTTGGTTAG	519 (0.009946%)	
CGTAAGAATTGTATCCTTGTTAGAAGACACAAAGCCAAAG	235 (0.001801%)	
CGTAGGCTCCATGCTCGCGCATCGAACTACCTACCACCTATCCTTCTCAGTTAATTCACGGGCGATGTTAC<br>GCTCGATGATGAGTTCCGGGGCCTGTGTT	3 (0.000057%)	
CGTATATTTAAGTTGTTGCAGTTAAAAAGCTCGTAGTTGAACCTTGGGATGGGTCGGCCGGTCCGCCTTTG<br>GTGTGCATTGGTCGGCTTGTCCCTTCGGT	3 (0.000057%)	
CGTATTCGTACTGAAAATCAGAATCAAACGAGCTTTTACCCTTTTGTTCCACACGAGATTTCTGTTCTCGT<br>TGAGCTCATCTTAGGACACCTGCGTTATC	15 (0.000287%)	
CGTATTTCATAGTCAGAGGTGAAATTCTTGGATTTATGAAAGACGAACAACTGCGAAAGCATTTGCCAAGG<br>ATGTTTTCATTAATCAAGAACGAAAGTTG	7 (0.000134%)	
CGTCCCTCACCATCCTTTGCTGATGCGGGACGGAAGCTGGTCTCCCGTGTGTTACCGCACGCGGTTGGCCT<br>AAATCCGAGCCAAGGACGCCTGGAGCGTA	11 (0.000211%)	
CGTCGAGTTATCATGAATCATCAGAGCAACGGGCAGAGCCCGCGTCGACCTTTTATCTAATAAATGCGTCC<br>CTTCCATAAGTCGGGGTTTGTTGCACGTA	104 (0.001993%)	
CGTCGTCCCTCACCATCCTTTGCTGATGCGGGACGGAAGCTGGTCTCCCGTGTGTTACCGCACGCGGTTGG<br>CCTAAATCCGAGCCAAGGACGCCTGGAGC	15 (0.000287%)	
CGTGCCTCGGCATCAGCGTGCTCCGGGCGTCGGCCTGTGGGCTCCCCATTCGACCCGTCTTGAAACACGGA<br>CCAAGGAGTCTGACATGTGTGCGAGTCAA	20 (0.000383%)	
CGTGCTCCGGGCGTCGGCCTGTGGGCTCCCCATTCGACCCGTCTTGAAACACGGACCAAGGAGTCTGACAT<br>GTGTGCGAGTCAACGGGTGAGTAAACCCG	9 (0.000172%)	
CGTGGGTGGTTCGCCGCCCGCGACGTCGCGAGAAGTCCACTAAACCTTATCATTTAGAGGAAGGAGAAGTC<br>GTAACAAGGTTTCCGTAGGTGAACCTGCG	35 (0.000671%)	
CGTTAAGGAGCTGTTGCTTTGTTAGTGTAGAAACACTTGTGTAGAATTGGGGATTGTTTTTTTTGGAGTGA<br>TTTAGGGGAGGGTCGAATCTTAGCGACAA	233 (0.004465%)	
CGTTAGGGAGTCCGGAGACGTCGGCGGGGGCCTCGGGAAGAGTTATCTTTTCTGTTTAACAGCCTGCCCAC<br>CCTGGAAACGGCTCAGCCGGAGGTAGGGT	3 (0.000057%)	
CGTTCATCACCCTTGGCCGGCTTTCGAACAGCCGGACTCCCATCAAAAGATGGTTGCCAAGAACATCTTCG<br>TTACGGTTTGCTAATTCTCGGAATAACAT	18 (0.000345%)	
CGTTGACTCGCACACATGTCAGACTCCTTGGTCCGTGTTTCAAGACGGGTCGAATGGGGAGCCCACAGGCC<br>GACGCCCGGAGCACGCTGATGCCGAGGCA	9 (0.000172%)	
CTAAATACGGGCGAGAGACCGATAGCGAACAAGTACCGCGAGGTAAAGATGAAAAGGACTTTGAAAAGAGA<br>GTCAAAGAGTGCTTGAAATTGTCGGGAGG	766 (0.014680%)	
CTAACGGCGTGCCTCGGCATCAGCGTGCTCCGGGCGTCGGCCTGTGGGCTCCCCATTCGACCCGTCTTGAA<br>ACACGGACCAAGGAGTCTGACATGTGTGC	107 (0.002051%)	
CTAACTAGCTACGTGGAGGCATCCCTTCACGGCCGGCTTCTTAGAGGGACTATGGCCGTTTAGGCCAAGGA<br>AGTTTGAGGCAATAACAGGTCTGTGATGC	38 (0.000728%)	
CTAAGTAGTGTTTCCTTGTTAGAAGACACAAAGCCAAAGACTCATATGGACTTTGGCTACACCATGAAAGC<br>TTTGAGAAGCAAGAAGAAGGTTGGTTAGT	72 (0.001380%)	
CTAATGTATCCAGAGCGTAGGCTTGCTTTGAGCACTCTAATTTCTTCAAAGTAACAGCGCCGGAGGCACGA<br>CCCGGCCAATTAAGACCAGGAGCGTATCG	28 (0.000537%)	
CTACATTGTTCCATCGACCAGAGGCTGTTCACCTTGGAGACCTGATGCGGTTATGAGTACGACCGGGCGTG<br>AGCGGCACTCGGTCCTCCGGATTTTCAAG	49 (0.000939%)	
CTACCTACCACCTATCCTTCTCAGTTAATTCACGGGCGATGTTACGCTCGATGATGAGTTCCGGGGCCTGT<br>GTTTCGTACCTAATTTGAAGGAATTGTTG	20 (0.000383%)	
CTAGCTACGTGGAGGCATCCCTTCACGGCCGGCTTCTTAGAGGGACTATGGCCGTTTAGGCCAAGGAAGTT<br>TGAGGCAATAACAGGTCTGTGATGCCCTT	23 (0.000441%)	
CTAGGCGAGACAAGGGTTCACATTTCGTTCATCACCCTTGGCCGGCTATCGAACAGCCGGACTCCCATCAA<br>AAGATGGTTGCCAAGAACATCTTCGTTAC	28 (0.000537%)	
CTAGGCGAGACAAGGGTTCACATTTCGTTCATCACCCTTGGCCGGCTTTCGAACAGCCGGACTCCCATCAA<br>AAGATGGTTGCCAAGAACATCTTCGTTAC	66 (0.001265%)	
CTAGGCTGTCCCGAGTGTGAGCGAGGTGTGAGTGTCGCCCATGGGCATCGACACCTTGCGGCTAGGAACTG<br>GAACGAGACGGGTGGCAAAGATTTCGAGT	402 (0.007704%)	
CTAGTGTTCGTAGGCTCCATGCTCGCGCATCGAACTACCTACCACCTATCCTTCTCAGTTAATTCACGGGC<br>GATGTTACGCTCGATGATGAGTTCCGGGG	19 (0.000364%)	
CTATCAACTTTCGATGGTAGGATAGTGGCCTACCATGGTGGTAACGGGTGACGGAGAATTAGGGTTCGATT<br>CCGGAGAGGGAGCCTGAGAAACGGCTACC	3 (0.000057%)	
CTATGATGTTATCCCATGCTAATGTATCCAGAGCGTAGGCTTGCTTTGAGCACTCTAATTTCTTCAAAGTA<br>ACAGCGCCGGAGGCACGACCCGGCCAATT	218 (0.004178%)	
CTCAAAATCATCAATCGTTCCAACTAATCTACCGAAGTACTCGGCTAAGAAGAAAGAAGACGGACGAATCC<br>GAGCCAAAGCCGTACAAGCGCGAGATACC	1983 (0.038003%)	
CTCAAAGATTAAGCCATGCATGTGTAAGTATGAACGAATTCAGACTGTGAAACTGCGAATGGCTCATTAAA<br>TCAGTTATAGTTTGTTTGATGGTAACTAC	19 (0.000364%)	
CTCAATCATACACATGACATCAAGTCATATTCGACTCCAAAACACTAACCAACCTTCTTCTTGCTTCTCAA<br>AGCTTTCATGGTGTAGCCAAAGTCCATAT	61 (0.001169%)	

CTCACCATCCTTTGCTGATGCGGGACGGAAGCTGGTCTCCCGTGTGTTACCGCACGCGGTTGGCCTAAATCCGAGCCAAGGACGCCTGGAGCGTACCGAC	16 (0.000307%)	
CTCACCCGTTGACTCGCACACATGTCAGACTCCTTGGTCCGTGTTTCAAGACGGGTCGAATGGGGAGCCCACAGGCCGACGCCCGGAGCACGCTGATGCC	8 (0.000153%)	
CTCAGCCTGCTAACTAGCTACGTGGAGGCATCCCTTCACGGCCGGCTTCTTAGAGGGACTATGGCCGTTTAGGCCAAGGAAGTTTGAGGCAATAACAGGT	26 (0.000498%)	
CTCATGTGTATGATTGAGTATAAGAACTTAAACCGCAACCGCATCTTATAAGCCTAAGTAGTGTTTCCTTGTTAGAAGACACAAAGCCAAAGACTCATAT	110 (0.002108%)	
CTCCAAAACACTAACCAACCTTCTTCTTGCTTCTCAAAGCTTTCATGGTGTAGCCAAAGTCCATATGAGTCTTTGGCTTTGTGTCTTCTAACAAGGATAC	29 (0.000556%)	
CTCCAATAGCGTATATTTAAGTTGTTGCAGTTAAAAAGCTCGTAGTTGAACCTTGGGATGGGTCGGCCGGTCCGCCTTTGGTGTGCATTGGTCGGCTTGT	32 (0.000613%)	
CTCCATGCTCGCGCATCGAACTACCTACCACCTATCCTTCTCAGTTAATTCACGGGCGATGTTACGCTCGATGATGAGTTCCGGGGCCTGTGTTTCGTAC	9 (0.000172%)	
CTCCGGGCGTCGGCCTGTGGGCTCCCCATTCGACCCGTCTTGAAACACGGACCAAGGAGTCTGACATGTGTGCGAGTCAACGGGTGAGTAAACCCGTAAG	17 (0.000326%)	
CTCGCACACATGTCAGACTCCTTGGTCCGTGTTTCAAGACGGGTCGAATGGGGAGCCCACAGGCCGACGCCCGGAGCACGCTGATGCCGAGGCACGCCGT	19 (0.000364%)	
CTCGCGCATCGAACTACCTACCACCTATCCTTCTCAGTTAATTCACGGGCGATGTTACGCTCGATGATGAGTTCCGGGGCCTGTGTTTCGTACCTAATTT	7 (0.000134%)	
CTCGCGCTTGTACGGCTTTGGCTCGGATTCGTCCGTCTTCTTTCTTCTTAGCCGAGTACTTCGGTAGATTAGTTGGAACGATTGATGATTTTGAGTTAAT	10 (0.000192%)	
CTCGGCATCAGCGTGCTCCGGGCGTCGGCCTGTGGGCTCCCCATTCGACCCGTCTTGAAACACGGACCAAGGAGTCTGACATGTGTGCGAGTCAACGGGT	24 (0.000460%)	
CTCGGTCCTCCGGATTTTCAAGGGCCGCCGGGGGCGCACCGGACACCACGCGACGTGCGGTGCTCTTCCAGCCGCTGGACCCTACCTCCGGCTGAGCCGT	32 (0.000613%)	
CTCGTGTAAAGTTGGGAATTCGTTAAGGAGCTGTTGCTTTGTTAGTGTAGAAACACTTGTGTAGAATTGGGGATTGTTTTTTTTGGAGTGATTTAGGGGA	33 (0.000632%)	
CTCTGTGCTGGCGACGCATCATTCAAATTTCTGCCCTATCAACTTTCGATGGTAGGATAGTGGCCTACCATGGTGGTAACGGGTGACGGAGAATTAGGGT	57 (0.001092%)	
CTGAGAAGGGTTCGAGTGTGAGCATGCCTGTCGGGACCCGAAAGATGGTGAACTATGCCTGAGCGGGGTAAAGCCAGAGGAAACTCTGGTGGAAGCCCGC	12 (0.000230%)	
CTGATGCGGGACGGAAGCTGGTCTCCCGTGTGTTACCGCACGCGGTTGGCCTAAATCCGAGCCAAGGACGCCTGGAGCGTACCGACATGCGGTGGTGAAC	29 (0.000556%)	
CTGCAGCACGCGCCTAACGGCGTGCCTCGGCATCAGCGTGCTCCGGGCGTCGGCCTGTGGGCTCCCCATTCGACCCGTCTTGAAACACGGACCAAGGAGT	2045 (0.039192%)	
CTGCCCTATCAACTTTCGATGGTAGGATAGTGGCCTACCATGGTGGTAACGGGTGACGGAGAATTAGGGTTCGATTCCGGAGAGGGAGCCTGAGAAACGG	11 (0.000211%)	
CTGCTAACTAGCTACGTGGAGGCATCCCTTCACGGCCGGCTTCTTAGAGGGACTATGGCCGTTTAGGCCAAGGAAGTTTGAGGCAATAACAGGTCTGTGA	52 (0.000997%)	
CTGGCGACGCATCATTCAAATTTCTGCCCTATCAACTTTCGATGGTAGGATAGTGGCCTACCATGGTGGTAACGGGTGACGGAGAATTAGGGTTCGATTC	24 (0.000460%)	
CTGGGGCGGCACATCTGTTAAAAGATAACGCAGGTGTCCTAAGATGAGCTCAACGAGAACAGAAATCTCGTGTGGAACAAAAGGGTAAAAGCTCGTTTGA	13 (0.000249%)	
CTGGGTGTCACAAATCGTCGTCCCTCACCATCCTTTGCTGATGCGGGACGGAAGCTGGTCTCCCGTGTGTTACCGCACGCGGTTGGCCTAAATCCGAGCC	870 (0.016673%)	
CTGGTGCCAGCAGCCGCGGTAATTCCAGCTCCAATAGCGTATATTTAAGTTGTTGCAGTTAAAAAGCTCGTAGTTGAACCTTGGGATGGGTCGGCCGGTC	66 (0.001265%)	
CTGTCCCGAAGGTATCTCGCGCTTGTACGGCTTTGGCTCGGATTCGTCCGTCTTCTTTCTTCTTAGCCGAGTACTTCGGTAGATTAGTTGGAACGATTGA	60 (0.001150%)	
CTGTCCCGAGTGTGAGCGAGGTGTGAGTGTCGCCCATGGGCATCGACACCTTGCGGCTAGGAACTGGAACGAGACGGGTAGCAAAGATTTCGAGTAGCAC	21 (0.000402%)	
CTGTCCCGAGTGTGAGCGAGGTGTGAGTGTCGCCCATGGGCATCGACACCTTGCGGCTAGGAACTGGAACGAGACGGGTGGCAAAGATTTCGAGTAGCAC	29 (0.000556%)	
CTGTGCTGGCGACGCATCATTCAAATTTCTGCCCTATCAACTTTCGATGGTAGGATAGTGGCCTACCATGGTGGTAACGGGTGACGGAGAATTAGGGTTC	23 (0.000441%)	
CTGTTGCTTTGTTAGTGTAGAAACACTTGTGTAGAATTGGGGATTGTTTTTTTTGGAGTGATTTAGGGGAGGGTCGAATCTTAGCGACAAAGGGCTGAAT	11 (0.000211%)	
CTTAAAGGCGTAAGAATTGTATCCTTGTTAGAAGACACAA	943 (0.007229%)	
CTTACGGGTTTACTCACCCGTTGACTCGCACACATGTCAGACTCCTTGGTCCGTGTTTCAAGACGGGTCGAATGGGGAGCCCACAGGCCGACGCCCGGAG	14 (0.000268%)	
CTTATACTCAATCATACACATGACATCAAGTCATATTCGACTCCAAAACACTAACCAACCTTCTTCTTGCTTCTCAAAGCTTTCATGGTGTAGCCAAAGT	117 (0.002242%)	
CTTCAAATTAGGTACGAAACACAGGCCCCGGAACTCATCATCGAGCGTAACATCGCCCGTGAATTAACTGAGAAGGATAGGTGGTAGGTAGTTCGATGCG	778 (0.014910%)	
CTTCAACGAGGAATTCCTAGTAAGCGCGAGTCATCAGCTCGCGTTGACTACGTCCCTGCCCTTTGTACACACCGCCCGTCGCTCCTACCGATTGAATGAT	69 (0.001322%)	
CTTCCCTTGCCTACATTGTTCCATCGACCAGAGGCTGTTCACCTTGGAGACCTGATGCGGTTATGAGTACGACCGGGCGTGAGCGGCACTCGGTCCTCCG	57 (0.001092%)	
CTTCCTTGCGCCTTACGGGTTTACTCACCCGTTGACTCGCACACATGTCAGACTCCTTGGTCCGTGTTTCAAGACGGGTCGAATGGGGAGCCCACAGGCC	103 (0.001974%)	
CTTCTGAGAAGGGTTCGAGTGTGAGCATGCCTGTCGGGACCCGAAAGATGGTGAACTATGCCTGAGCGGGGTAAAGCCAGAGGAAACTCTGGTGGAAGCC	17 (0.000326%)	
CTTGCCTACATTGTTCCATCGACCAGAGGCTGTTCACCTTGGAGACCTGATGCGGTTATGAGTACGACCGGGCGTGAGCGGCACTCGGTCCTCCGGATTT	51 (0.000977%)	
CTTGCGCCTTACGGGTTTACTCACCCGTTGACTCGCACACATGTCAGACTCCTTGGTCCGTGTTTCAAGACGGGTCGAATGGGGAGCCCACAGGCCGACG	26 (0.000498%)	
CTTGGAGACCTGATGCGGTTATGAGTACGACCGGGCGTGAGCGGCACTCGGTCCTCCGGATTTTCAAGGGCCGCCGGGGGCGCACCGGACACCACGCGAC	118 (0.002261%)	
CTTGGTGATATGAACACAAACGTTCAATATGACAAACCCATGCCAAGTAAAGAGAAAATGAAAACTGGTGATTGTTGCGGAAATCGTCCAGGATTCCTCG	13 (0.000249%)	
CTTGTACGGCTTTGGCTCGGATTCGTCCGTCTTCTTTCTTCTTAGCCGAGTACTTCGGTAGATTAGTTGGAACGATTGATGATTTTGAGTTAATTGAACG	12 (0.000230%)	
CTTGTCTCAAAGATTAAGCCATGCATGTGTAAGTATGAACGAATTCAGACTGTGAAACTGCGAATGGCTCATTAAATCAGTTATAGTTTGTTTGATGGTA	49 (0.000939%)	

CTTGTTAGAAGACACAAAGCCAAAGACTCATATGGACTTTGGCTACACCATGAAAGCTTTGAGAAGCAAGAAGAAGGTTGGTTAGTGTTTTGGAGTCGAA	111 (0.002127%)	
CTTTCACGGTTCGTATTCGTACTGAAAATCAGAATCAAACGAGCTTTTACCCTTTTGTTCCACACGAGATTTCTGTTCTCGTTGAGCTCATCTTAGGACA	35 (0.000671%)	
CTTTCGATGGTAGGATAGTGGCCTACCATGGTGGTAACGGGTGACGGAGAATTAGGGTTCGATTCCGGAGAGGGAGCCTGAGAAACGGCTACCACATCCA	8 (0.000153%)	
CTTTGCTGATGCGGGACGGAAGCTGGTCTCCCGTGTGTTACCGCACGCGGTTGGCCTAAATCCGAGCCAAGGACGCCTGGAGCGTACCGACATGCGGTGG	73 (0.001399%)	
CTTTGGCTCGGATTCGTCCGTCTTCTTTCTTCTTAGCCGAGTACTTCGGTAGATTAGTTGGAACGATTGATGATTTTGAGTTAATTGAACGTTCGGCGTA	9 (0.000172%)	
CTTTGTTTTAATTAAACAGTCGGATTCCCCTTGTCCGTACCAGTTCTGAGCTGACTGTTCGACGCCCGGGGAAAGCTCCCGAGAGAGCCGTTCCCAGTCC	81 (0.001552%)	
GAAACACAGGCCCCGGAACTCATCATCGAGCGTAACATCGCCCGTGAATTAACTGAGAAGGATAGGTGGTAGGTAGTTCGATGCGCGAGCATGGAGCCTA	76 (0.001457%)	
GAAATCCTATGATGTTATCCCATGCTAATGTATCCAGAGCGTAGGCTTGCTTTGAGCACTCTAATTTCTTCAAAGTAACAGCGCCGGAGGCACGACCCGG	191 (0.003660%)	
GAACACAAACGTTCAATATGACAAACCCATGCCAAGTAAAGAGAAAATGAAAACTGGTGATTGTTGCGGAAATCGTCCAGGATTCCTCGACCAGGACTTG	6 (0.000115%)	
GAACCCACAAAGGGTGTTGGTCGATTAAGACAGCAGGACGGTGGTCATGGAAGTCGAAATCCGCTAAGGAGTGTGTAACAACTCACCTGCCGAATCAACT	898 (0.017210%)	
GAACCGGGACGTGGCGGTTGACGGCAACGTTAGGGAGTCCGGAGACGTCGGCGGGGGCCTCGGGAAGAGTTATCTTTTCTGTTTAACAGCCTGCCCACCC	12 (0.000230%)	
GAACTACCTACCACCTATCCTTCTCAGTTAATTCACGGGCGATGTTACGCTCGATGATGAGTTCCGGGGCCTGTGTTTCGTACCTAATTTGAAGGAATTG	5 (0.000096%)	
GAAGACACAAAGCCAAAGACTCATATGGACTTTGGCTACACCATGAAAGCTTTGAGAAGCAAGAAGAAGGTTGGTTAGTGTTTTGGAGTCGAATATGACT	35 (0.000671%)	
GAAGCACGCCCATCCAACCTAGGCGAGACAAGGGTTCACATTTCGTTCATCACCCTTGGCCGGCTATCGAACAGCCGGACTCCCATCAAAAGATGGTTGC	15 (0.000287%)	
GAAGGGTTCGAGTGTGAGCATGCCTGTCGGGACCCGAAAGATGGTGAACTATGCCTGAGCGGGGTAAAGCCAGAGGAAACTCTGGTGGAAGCCCGCAGCG	5 (0.000096%)	
GAAGGTATCTCGCGCTTGTACGGCTTTGGCTCGGATTCGTCCGTCTTCTTTCTTCTTAGCCGAGTACTTCGGTAGATTAGTTGGAACGATTGATGATTTT	11 (0.000211%)	
GAAGTCGAAATCCGCTAAGGAGTGTGTAACAACTCACCTGCCGAATCAACTAGCCCCGAAAATGGATGGCGCTTAAGCGCGCGACCTATACCCGGCCGTC	3 (0.000057%)	
GAATCATCAGAGCAACGGGCAGAGCCCGCGTCGACCTTTTATCTAATAAATGCGTCCCTTCCATAAGTCGGGGTTTGTTGCACGTATTAGCTCTAGAATT	97 (0.001859%)	
GAATTGTATCCTTGTTAGAAGACACAAAGCCAAAGACTCATATGGACTTTGGCTACACCATGAAAGCTTTGAGAAGCAAGAAGAAGGTTGGTTAGTGTTT	16 (0.000307%)	
GACAAGGGTTCACATTTCGTTCATCACCCTTGGCCGGCTATCGAACAGCCGGACTCCCATCAAAAGATGGTTGCCAAGAACATCTTCGTTACGGTTTGCT	6 (0.000115%)	
GACAAGGGTTCACATTTCGTTCATCACCCTTGGCCGGCTTTCGAACAGCCGGACTCCCATCAAAAGATGGTTGCCAAGAACATCTTCGTTACGGTTTGCT	14 (0.000268%)	
GACACAAAGCCAAAGACTCATATGGACTTTGGCTACACCATGAAAGCTTTGAGAAGCAAGAAGAAGGTTGGTTAGTGTTTTGGAGTCGAATATGACTTGA	51 (0.000977%)	
GACAGCAGGACGGTGGTCATGGAAGTCGAAATCCGCTAAGGAGTGTGTAACAACTCACCTGCCGAATCAACTAGCCCCGAAAATGGATGGCGCTTAAGCG	6 (0.000115%)	
GACATCAAGTCATATTCGACTCCAAAACACTAACCAACCTTCTTCTTGCTTCTCAAAGCTTTCATGGTGTAGCCAAAGTCCATATGAGTCTTTGGCTTTG	45 (0.000862%)	
GACATTGTCAGGTGGGGAGTTTGGCTGGGGCGGCACATCTGTTAAAAGATAACGCAGGTGTCCTAAGATGAGCTCAACGAGAACAGAAATCTCGTGTGGA	32 (0.000613%)	
GACCAGGGGTTGAAATCGTCGACCAGGTCCGAGACTTCATCGACCGGGTCCGAGGATTCGTCGACCAGGACGGCCGGATGTCCGAGAAAAAAAAATGTTG	10 (0.000192%)	
GACCCGCCGAAGCGAGCCTTGGGACCAAAAACAGGGGTTGTACCCCGCCTCCGATTCACGGAGTAAGTAAAATAACGTTAAAAGTAGTGGTATTTCACTT	6 (0.000115%)	
GACCTCAGCCTGCTAACTAGCTACGTGGAGGCATCCCTTCACGGCCGGCTTCTTAGAGGGACTATGGCCGTTTAGGCCAAGGAAGTTTGAGGCAATAACA	8 (0.000153%)	
GACGCATCATTCAAATTTCTGCCCTATCAACTTTCGATGGTAGGATAGTGGCCTACCATGGTGGTAACGGGTGACGGAGAATTAGGGTTCGATTCCGGAG	15 (0.000287%)	
GACGGGCGGTGTGTACAAAGGGCAGGGACGTAGTCAACGCGAGCTGATGACTCGCGCTTACTAGGAATTCCTCGTTGAAGACCAACAATTGCAATGATCG	12 (0.000230%)	
GACGGTGGTCATGGAAGTCGAAATCCGCTAAGGAGTGTGTAACAACTCACCTGCCGAATCAACTAGCCCCGAAAATGGATGGCGCTTAAGCGCGCGACCT	3 (0.000057%)	
GACGTCGGCGGGGGCCTCGGGAAGAGTTATCTTTTCTGTTTAACAGCCTGCCCACCCTGGAAACGGCTCAGCCGGAGGTAGGGTCCAGCGGCTGGAAGAG	4 (0.000077%)	
GACGTGGGTGGTTCGCCGCCCGCGACGTCGCGAGAAGTCCACTAAACCTTATCATTTAGAGGAAGGAGAAGTCGTAACAAGGTTTCCGTAGGTGAACCTG	42 (0.000805%)	
GACTCCAAAACACTAACCAACCTTCTTCTTGCTTCTCAAAGCTTTCATGGTGTAGCCAAAGTCCATATGAGTCTTTGGCTTTGTGTCTTCTAACAAGGAT	25 (0.000479%)	
GACTTCCCTTGCCTACATTGTTCCATCGACCAGAGGCTGTTCACCTTGGAGACCTGATGCGGTTATGAGTACGACCGGGCGTGAGCGGCACTCGGTCCTC	24 (0.000460%)	
GAGAAGGGTTCGAGTGTGAGCATGCCTGTCGGGACCCGAAAGATGGTGAACTATGCCTGAGCGGGGTAAAGCCAGAGGAAACTCTGGTGGAAGCCCGCAG	7 (0.000134%)	
GAGACAAGGGTTCACATTTCGTTCATCACCCTTGGCCGGCTTTCGAACAGCCGGACTCCCATCAAAAGATGGTTGCCAAGAACATCTTCGTTACGGTTTG	6 (0.000115%)	
GAGACCTCAGCCTGCTAACTAGCTACGTGGAGGCATCCCTTCACGGCCGGCTTCTTAGAGGGACTATGGCCGTTTAGGCCAAGGAAGTTTGAGGCAATAA	934 (0.017900%)	
GAGACGTCGGCGGGGGCCTCGGGAAGAGTTATCTTTTCTGTTTAACAGCCTGCCCACCCTGGAAACGGCTCAGCCGGAGGTAGGGTCCAGCGGCTGGAAG	15 (0.000287%)	
GAGCAACGGGCAGAGCCCGCGTCGACCTTTTATCTAATAAATGCGTCCCTTCCATAAGTCGGGGTTTGTTGCACGTATTAGCTCTAGAATTACTACGGTT	14 (0.000268%)	
GAGCCCGCGTCGACCTTTTATCTAATAAATGCGTCCCTTCCATAAGTCGGGGTTTGTTGCACGTATTAGCTCTAGAATTACTACGGTTATCCGAGTAGTA	8 (0.000153%)	
GAGCGACGGGCGGTGTGTACAAAGGGCAGGGACGTAGTCAACGCGAGCTGATGACTCGCGCTTACTAGGAATTCCTCGTTGAAGACCAACAATTGCAATG	12 (0.000230%)	
GAGCGAGGTGTGAGTGTCGCCCATGGGCATCGACACCTTGCGGCTAGGAACTGGAACGAGACGGGTGGCAAAGATTTCGAGTAGCACTTCATACTACCGT	9 (0.000172%)	

GAGCGGCACTCGGTCCTCCGGATTTTCAAGGGCCGCCGGGGGCGCACCGGACACCACGCGACGTGCGGTGCTCTTCCAGCCGCTGGACCCTACCTCCGGC	14 (0.000268%)	
GAGCTCGTGTAAAGTTGGGAATTCGTTAAGGAGCTGTTGCTTTGTTAGTGTAGAAACACTTGTGTAGAATTGGGGATTGTTTTTTTTGGAGTGATTTAGG	10 (0.000192%)	
GAGCTGTTGCTTTGTTAGTGTAGAAACACTTGTGTAGAATTGGGGATTGTTTTTTTTGGAGTGATTTAGGGGAGGGTCGAATCTTAGCGACAAAGGGCTG	5 (0.000096%)	
GAGGACATTGTCAGGTGGGGAGTTTGGCTGGGGCGGCACATCTGTTAAAAGATAACGCAGGTGTCCTAAGATGAGCTCAACGAGAACAGAAATCTCGTGT	58 (0.001112%)	
GAGGCTGTTCACCTTGGAGACCTGATGCGGTTATGAGTACGACCGGGCGTGAGCGGCACTCGGTCCTCCGGATTTTCAAGGGCCGCCGGGGGCGCACCGG	10 (0.000192%)	
GAGGGCAAGTCTGGTGCCAGCAGCCGCGGTAATTCCAGCTCCAATAGCGTATATTTAAGTTGTTGCAGTTAAAAAGCTCGTAGTTGAACCTTGGGATGGG	22 (0.000422%)	
GAGGTGTGAGTGTCGCCCATGGGCATCGACACCTTGCGGCTAGGAACTGGAACGAGACGGGTAGCAAAGATTTCGAGTAGCACTTCATACTACCGTGGGT	66 (0.001265%)	
GAGGTGTGAGTGTCGCCCATGGGCATCGACACCTTGCGGCTAGGAACTGGAACGAGACGGGTGGCAAAGATTTCGAGTAGCACTTCATACTACCGTGGGT	11 (0.000211%)	
GAGTATAAGAACTTAAACCGCAACCGCATCTTATAAGCCTAAGTAGTGTTTCCTTGTTAGAAGACACAAAGCCAAAGACTCATATGGACTTTGGCTACAC	6 (0.000115%)	
GAGTCGAATATGACTTGATGTCATGTGTATGATTGAGTAT	1382 (0.010594%)	
GAGTGTCGCCCATGGGCATCGACACCTTGCGGCTAGGAACTGGAACGAGACGGGTGGCAAAGATTTCGAGTAGCACTTCATACTACCGTGGGTTTTTTAA	4 (0.000077%)	
GAGTGTGAGCGAGGTGTGAGTGTCGCCCATGGGCATCGACACCTTGCGGCTAGGAACTGGAACGAGACGGGTAGCAAAGATTTCGAGTAGCACTTCATAC	3 (0.000057%)	
GAGTTATCATGAATCATCAGAGCAACGGGCAGAGCCCGCGTCGACCTTTTATCTAATAAATGCGTCCCTTCCATAAGTCGGGGTTTGTTGCACGTATTAG	84 (0.001610%)	
GAGTTTGGCTGGGGCGGCACATCTGTTAAAAGATAACGCAGGTGTCCTAAGATGAGCTCAACGAGAACAGAAATCTCGTGTGGAACAAAAGGGTAAAAGC	4 (0.000077%)	
GATAGCTAGTGTTCGTAGGCTCCATGCTCGCGCATCGAACTACCTACCACCTATCCTTCTCAGTTAATTCACGGGCGATGTTACGCTCGATGATGAGTTC	13 (0.000249%)	
GATCCATTACATTTTATCGGTCGCTCTTGTCCGGAAGCTGTAGATGACCCAAAGTCCATATAGCGACCCCAGGTCAGGCGGGATTACCCGCTGAGTTTAA	12 (0.000230%)	
GATCCCGCCAATCAGCTTCCTTGCGCCTTACGGGTTTACTCACCCGTTGACTCGCACACATGTCAGACTCCTTGGTCCGTGTTTCAAGACGGGTCGAATG	523 (0.010023%)	
GATCCGTCGAGTTATCATGAATCATCAGAGCAACGGGCAGAGCCCGCGTCGACCTTTTATCTAATAAATGCGTCCCTTCCATAAGTCGGGGTTTGTTGCA	131 (0.002511%)	
GATCGAAATCCTATGATGTTATCCCATGCTAATGTATCCAGAGCGTAGGCTTGCTTTGAGCACTCTAATTTCTTCAAAGTAACAGCGCCGGAGGCACGAC	1612 (0.030893%)	
GATCGACCCGCCGAAGCGAGCCTTGGGACCAAAAACAGGGGTTGTACCCCGCCTCCGATTCACGGAGTAAGTAAAATAACGTTAAAAGTAGTGGTATTTC	41 (0.000786%)	
GATCGATCAA	168754 (0.323410%)	
GATCGATCCATTACATTTTATCGGTCGCTCTTGTCCGGAAGCTGTAGATGACCCAAAGTCCATATAGCGACCCCAGGTCAGGCGGGATTACCCGCTGAGT	1202 (0.023036%)	
GATCGATCCGTAACTTCGGGAAAAGGATTGGCTCTGAGGGCTGGGCTCGGGGGTCCCAGTTCCGAACCCGTCGGCTGTCAGCGGACTGCTCGAGCTGCTT	1224 (0.023457%)	
GATCGATCCGTCGAGTTATCATGAATCATCAGAGCAACGGGCAGAGCCCGCGTCGACCTTTTATCTAATAAATGCGTCCCTTCCATAAGTCGGGGTTTGT	1215 (0.023285%)	
GATCGATCGACCCGCCGAAGCGAGCCTTGGGACCAAAAACAGGGGTTGTACCCCGCCTCCGATTCACGGAGTAAGTAAAATAACGTTAAAAGTAGTGGTA	989 (0.018954%)	
GATCGATCTCATGTGTATGATTGAGTATAAGAACTTAAACCGCAACCGCATCTTATAAGCCTAAGTAGTGTTTCCTTGTTAGAAGACACAAAGCCAAAGA	233 (0.004465%)	
GATCGATCTT	187116 (0.358600%)	
GATCGATCTTAAAGGCGTAAGAATTGTATCCTTGTTAAAAGACACAAAGCCAAAGACTCATATGGACTTTGGCTACACCATGAAAGCTTTGAGAAGCAAG	467 (0.008950%)	
GATCGATCTTAAAGGCGTAAGAATTGTATCCTTGTTAGAA	4228 (0.032411%)	
GATCGATCTTCTGAGAAGGGTTCGAGTGTGAGCATGCCTGTCGGGACCCGAAAGATGGTGAACTATGCCTGAGCGGGGTAAAGCCAGAGGAAACTCTGGT	1125 (0.021560%)	
GATCTCATGTGTATGATTGAGTATAAGAACTTAAACCGCAACCGCATCTTATAAGCCTAAGTAGTGTTTCCTTGTTAGAAGACACAAAGCCAAAGACTCA	49 (0.000939%)	
GATCTTAAAGGCGTAAGAATTGTATCCTTGTTAAAAGACACAAAGCCAAAGACTCATATGGACTTTGGCTACACCATGAAAGCTTTGAGAAGCAAGAAGA	253 (0.004849%)	
GATCTTCTGAGAAGGGTTCGAGTGTGAGCATGCCTGTCGGGACCCGAAAGATGGTGAACTATGCCTGAGCGGGGTAAAGCCAGAGGAAACTCTGGTGGAA	134 (0.002568%)	
GATGTTATCCCATGCTAATGTATCCAGAGCGTAGGCTTGCTTTGAGCACTCTAATTTCTTCAAAGTAACAGCGCCGGAGGCACGACCCGGCCAATTAAGA	17 (0.000326%)	
GATTAACAGGGACAGTCGGGGGCATTCGTATTTCATAGTCAGAGGTGAAATTCTTGGATTTATGAAAGACGAACAACTGCGAAAGCATTTGCCAAGGATG	150 (0.002875%)	
GATTAAGACAGCAGGACGGTGGTCATGGAAGTCGAAATCCGCTAAGGAGTGTGTAACAACTCACCTGCCGAATCAACTAGCCCCGAAAATGGATGGCGCT	37 (0.000709%)	
GATTAGTCTTTCGCCCCTATACCCAAGTCAGACGAACGATTTGCACGTCAGTATCGCTGCGGGCTTCCACCAGAGTTTCCTCTGGCTTTACCCCGCTCAG	21 (0.000402%)	
GATTGAGTATAAGAACTTAAACCGCAACCGCATCTTATAAGCCTAAGTAGTGTTTCCTTGTTAGAAGACACAAAGCCAAAGACTCATATGGACTTTGGCT	35 (0.000671%)	
GATTTGCACGTCAGTATCGCTGCGGGCTTCCACCAGAGTTTCCTCTGGCTTTACCCCGCTCAGGCATAGTTCACCATCTTTCGGGTCCCGACAGGCATGC	29 (0.000556%)	
GATTTTCAAGGGCCGCCGGGGGCGCACCGGACACCACGCGACGTGCGGTGCTCTTCCAGCCGCTGGACCCTACCTCCGGCTGAGCCGTTTCCAGGGTGGG	9 (0.000172%)	
GCAACGGGCAGAGCCCGCGTCGACCTTTTATCTAATAAATGCGTCCCTTCCATAAGTCGGGGTTTGTTGCACGTATTAGCTCTAGAATTACTACGGTTAT	34 (0.000652%)	
GCAACGTTAGGGAGTCCGGAGACGTCGGCGGGGGCCTCGGGAAGAGTTATCTTTTCTGTTTAACAGCCTGCCCACCCTGGAAACGGCTCAGCCGGAGGTA	18 (0.000345%)	
GCAAGTCTGGTGCCAGCAGCCGCGGTAATTCCAGCTCCAATAGCGTATATTTAAGTTGTTGCAGTTAAAAAGCTCGTAGTTGAACCTTGGGATGGGTCGG	26 (0.000498%)	

GCACCGGACACCACGCGACGTGCGGTGCTCTTCCAGCCGCTGGACCCTACCTCCGGCTGAGCCGTTTCCAGGGTGGGCAGGCTGTTAAACAGAAAAGATA	22 (0.000422%)	
GCACGCCCATCCAACCTAGGCGAGACAAGGGTTCACATTTCGTTCATCACCCTTGGCCGGCTATCGAACAGCCGGACTCCCATCAAAAGATGGTTGCCAA	38 (0.000728%)	
GCACGCGCCTAACGGCGTGCCTCGGCATCAGCGTGCTCCGGGCGTCGGCCTGTGGGCTCCCCATTCGACCCGTCTTGAAACACGGACCAAGGAGTCTGAC	22 (0.000422%)	
GCACTCGGTCCTCCGGATTTTCAAGGGCCGCCGGGGGCGCACCGGACACCACGCGACGTGCGGTGCTCTTCCAGCCGCTGGACCCTACCTCCGGCTGAGC	6 (0.000115%)	
GCAGAGCCCGCGTCGACCTTTTATCTAATAAATGCGTCCCTTCCATAAGTCGGGGTTTGTTGCACGTATTAGCTCTAGAATTACTACGGTTATCCGAGTA	20 (0.000383%)	
GCAGCACGCGCCTAACGGCGTGCCTCGGCATCAGCGTGCTCCGGGCGTCGGCCTGTGGGCTCCCCATTCGACCCGTCTTGAAACACGGACCAAGGAGTCT	419 (0.008030%)	
GCAGCCGCGGTAATTCCAGCTCCAATAGCGTATATTTAAGTTGTTGCAGTTAAAAAGCTCGTAGTTGAACCTTGGGATGGGTCGGCCGGTCCGCCTTTGG	5 (0.000096%)	
GCAGGACGGTGGTCATGGAAGTCGAAATCCGCTAAGGAGTGTGTAACAACTCACCTGCCGAATCAACTAGCCCCGAAAATGGATGGCGCTTAAGCGCGCG	46 (0.000882%)	
GCATCAGCGTGCTCCGGGCGTCGGCCTGTGGGCTCCCCATTCGACCCGTCTTGAAACACGGACCAAGGAGTCTGACATGTGTGCGAGTCAACGGGTGAGT	7 (0.000134%)	
GCATCATTCAAATTTCTGCCCTATCAACTTTCGATGGTAGGATAGTGGCCTACCATGGTGGTAACGGGTGACGGAGAATTAGGGTTCGATTCCGGAGAGG	45 (0.000862%)	
GCATCGACACCTTGCGGCTAGGAACTGGAACGAGACGGGTGGCAAAGATTTCGAGTAGCACTTCATACTACCGTGGGTTTTTTAAACCTTCCGAGTTTTG	7 (0.000134%)	
GCATGGCCTCTGTGCTGGCGACGCATCATTCAAATTTCTGCCCTATCAACTTTCGATGGTAGGATAGTGGCCTACCATGGTGGTAACGGGTGACGGAGAA	1617 (0.030989%)	
GCATTCGTATTTCATAGTCAGAGGTGAAATTCTTGGATTTATGAAAGACGAACAACTGCGAAAGCATTTGCCAAGGATGTTTTCATTAATCAAGAACGAA	3 (0.000057%)	
GCCAAAGACTCATATGGACTTTGGCTACACCATGAAAGCTTTGAGAAGCAAGAAGAAGGTTGGTTAGTGTTTTGGAGTCGAATATGACTTGATGTCATGT	51 (0.000977%)	
GCCAATCAGCTTCCTTGCGCCTTACGGGTTTACTCACCCGTTGACTCGCACACATGTCAGACTCCTTGGTCCGTGTTTCAAGACGGGTCGAATGGGGAGC	128 (0.002453%)	
GCCAGCAGCCGCGGTAATTCCAGCTCCAATAGCGTATATTTAAGTTGTTGCAGTTAAAAAGCTCGTAGTTGAACCTTGGGATGGGTCGGCCGGTCCGCCT	25 (0.000479%)	
GCCCATCCAACCTAGGCGAGACAAGGGTTCACATTTCGTTCATCACCCTTGGCCGGCTATCGAACAGCCGGACTCCCATCAAAAGATGGTTGCCAAGAAC	60 (0.001150%)	
GCCCATCCAACCTAGGCGAGACAAGGGTTCACATTTCGTTCATCACCCTTGGCCGGCTTTCGAACAGCCGGACTCCCATCAAAAGATGGTTGCCAAGAAC	34 (0.000652%)	
GCCCATGGGCATCGACACCTTGCGGCTAGGAACTGGAACGAGACGGGTGGCAAAGATTTCGAGTAGCACTTCATACTACCGTGGGTTTTTTAAACCTTCC	8 (0.000153%)	
GCCCTATCAACTTTCGATGGTAGGATAGTGGCCTACCATGGTGGTAACGGGTGACGGAGAATTAGGGTTCGATTCCGGAGAGGGAGCCTGAGAAACGGCT	23 (0.000441%)	
GCCGAAGCGAGCCTTGGGACCAAAAACAGGGGTTGTACCCCGCCTCCGATTCACGGAGTAAGTAAAATAACGTTAAAAGTAGTGGTATTTCACTTGCGCC	29 (0.000556%)	
GCCGACTTCCCTTGCCTACATTGTTCCATCGACCAGAGGCTGTTCACCTTGGAGACCTGATGCGGTTATGAGTACGACCGGGCGTGAGCGGCACTCGGTC	204 (0.003910%)	
GCCGCCCGCGACGTCGCGAGAAGTCCACTAAACCTTATCATTTAGAGGAAGGAGAAGTCGTAACAAGGTTTCCGTAGGTGAACCTGCGGAAGGATCGATC	23 (0.000441%)	
GCCGCCGGGGGCGCACCGGACACCACGCGACGTGCGGTGCTCTTCCAGCCGCTGGACCCTACCTCCGGCTGAGCCGTTTCCAGGGTGGGCAGGCTGTTAA	9 (0.000172%)	
GCCGCGGTAATTCCAGCTCCAATAGCGTATATTTAAGTTGTTGCAGTTAAAAAGCTCGTAGTTGAACCTTGGGATGGGTCGGCCGGTCCGCCTTTGGTGT	11 (0.000211%)	
GCCGGGGGCGCACCGGACACCACGCGACGTGCGGTGCTCTTCCAGCCGCTGGACCCTACCTCCGGCTGAGCCGTTTCCAGGGTGGGCAGGCTGTTAAACA	17 (0.000326%)	
GCCTAACGGCGTGCCTCGGCATCAGCGTGCTCCGGGCGTCGGCCTGTGGGCTCCCCATTCGACCCGTCTTGAAACACGGACCAAGGAGTCTGACATGTGT	34 (0.000652%)	
GCCTAAGTAGTGTTTCCTTGTTAGAACATACAAAGCCAAAGACTCATACGGACTTTGGCTACACCATGAAAGCTTTGAGAAGCTAGAAGAAGGTTGGTTA	54 (0.001035%)	
GCCTAAGTAGTGTTTCCTTGTTAGAAGACACAAAGCCAAAGACTCATATGGACTTTGGCTACACCATGAAAGCTTTGAGAAGCAAGAAGAAGGTTGGTTA	34 (0.000652%)	
GCCTACATTGTTCCATCGACCAGAGGCTGTTCACCTTGGAGACCTGATGCGGTTATGAGTACGACCGGGCGTGAGCGGCACTCGGTCCTCCGGATTTTCA	3 (0.000057%)	
GCCTAGGCTGTCCCGAGTGTGAGCGAGGTGTGAGTGTCGCCCATGGGCATCGACACCTTGCGGCTAGGAACTGGAACGAGACGGGTGGCAAAGATTTCGA	358 (0.006861%)	
GCCTCGGCATCAGCGTGCTCCGGGCGTCGGCCTGTGGGCTCCCCATTCGACCCGTCTTGAAACACGGACCAAGGAGTCTGACATGTGTGCGAGTCAACGG	17 (0.000326%)	
GCCTCTGTGCTGGCGACGCATCATTCAAATTTCTGCCCTATCAACTTTCGATGGTAGGATAGTGGCCTACCATGGTGGTAACGGGTGACGGAGAATTAGG	13 (0.000249%)	
GCCTGCTAACTAGCTACGTGGAGGCATCCCTTCACGGCCGGCTTCTTAGAGGGACTATGGCCGTTTAGGCCAAGGAAGTTTGAGGCAATAACAGGTCTGT	5 (0.000096%)	
GCCTTACGGGTTTACTCACCCGTTGACTCGCACACATGTCAGACTCCTTGGTCCGTGTTTCAAGACGGGTCGAATGGGGAGCCCACAGGCCGACGCCCGG	31 (0.000594%)	
GCGACGCATCATTCAAATTTCTGCCCTATCAACTTTCGATGGTAGGATAGTGGCCTACCATGGTGGTAACGGGTGACGGAGAATTAGGGTTCGATTCCGG	35 (0.000671%)	
GCGACGGGCGGTGTGTACAAAGGGCAGGGACGTAGTCAACGCGAGCTGATGACTCGCGCTTACTAGGAATTCCTCGTTGAAGACCAACAATTGCAATGAT	46 (0.000882%)	
GCGACGTGGGTGGTTCGCCGCCCGCGACGTCGCGAGAAGTCCACTAAACCTTATCATTTAGAGGAAGGAGAAGTCGTAACAAGGTTTCCGTAGGTGAACC	347 (0.006650%)	
GCGAGACAAGGGTTCACATTTCGTTCATCACCCTTGGCCGGCTATCGAACAGCCGGACTCCCATCAAAAGATGGTTGCCAAGAACATCTTCGTTACGGTT	29 (0.000556%)	
GCGAGACAAGGGTTCACATTTCGTTCATCACCCTTGGCCGGCTTTCGAACAGCCGGACTCCCATCAAAAGATGGTTGCCAAGAACATCTTCGTTACGGTT	39 (0.000747%)	
GCGAGGTGTGAGTGTCGCCCATGGGCATCGACACCTTGCGGCTAGGAACTGGAACGAGACGGGTGGCAAAGATTTCGAGTAGCACTTCATACTACCGTGG	48 (0.000920%)	
GCGCACCGGACACCACGCGACGTGCGGTGCTCTTCCAGCCGCTGGACCCTACCTCCGGCTGAGCCGTTTCCAGGGTGGGCAGGCTGTTAAACAGAAAAGA	27 (0.000517%)	
GCGCATCGAACTACCTACCACCTATCCTTCTCAGTTAATTCACGGGCGATGTTACGCTCGATGATGAGTTCCGGGGCCTGTGTTTCGTACCTAATTTGAA	5 (0.000096%)	

GCGCCTAACGGCGTGCCTCGGCATCAGCGTGCTCCGGGCGTCGGCCTGTGGGCTCCCCATTCGACCCGTCTTGAAACACGGACCAAGGAGTCTGACATGT	15 (0.000287%)
GCGCCTTACGGGTTTACTCACCCGTTGACTCGCACACATGTCAGACTCCTTGGTCCGTGTTTCAAGACGGGTCGAATGGGGAGCCCACAGGCCGACGCCC	20 (0.000383%)
GCGCTTGTACGGCTTTGGCTCGGATTCGTCCGTCTTCTTTCTTCTTAGCCGAGTACTTCGGTAGATTAGTTGGAACGATTGATGATTTTGAGTTAATTGA	10 (0.000192%)
GCGGAGGACATTGTCAGGTGGGGAGTTTGGCTGGGGCGGCACATCTGTTAAAAGATAACGCAGGTGTCCTAAGATGAGCTCAACGAGAACAGAAATCTCG	1475 (0.028268%)
GCGGCACATCTGTTAAAAGATAACGCAGGTGTCCTAAGATGAGCTCAACGAGAACAGAAATCTCGTGTGGAACAAAAGGGTAAAAGCTCGTTTGATTCTG	8 (0.000153%)
GCGGCACTCGGTCCTCCGGATTTTCAAGGGCCGCCGGGGGCGCACCGGACACCACGCGACGTGCGGTGCTCTTCCAGCCGCTGGACCCTACCTCCGGCTG	60 (0.001150%)
GCGGCGACGTGGGTGGTTCGCCGCCCGCGACGTCGCGAGAAGTCCACTAAACCTTATCATTTAGAGGAAGGAGAAGTCGTAACAAGGTTTCCGTAGGTGA	1261 (0.024167%)
GCGGTAATTCCAGCTCCAATAGCGTATATTTAAGTTGTTGCAGTTAAAAAGCTCGTAGTTGAACCTTGGGATGGGTCGGCCGGTCCGCCTTTGGTGTGCA	18 (0.000345%)
GCGGTTGACGGCAACGTTAGGGAGTCCGGAGACGTCGGCGGGGGCCTCGGGAAGAGTTATCTTTTCTGTTTAACAGCCTGCCCACCCTGGAAACGGCTCA	10 (0.000192%)
GCGGTTTAAGTTCTTATACTCAATCATACACATGACATCAAGTCATATTCGACTCCAAAACACTAACCAACCTTCTTCTTGCTTCTCAAAGCTTTCATGG	139 (0.002664%)
GCGGTTTAAGTTGTTATACTCAATCATACACATGACAACAAGTCATATTCGACTCCAAAACACTAACCAACCTTCTTCTTGCTTCTCAAAGCTTTCATGG	45 (0.000862%)
GCGTAAGAATTGTATCCTTGTTAGAAGACACAAAGCCAAA	170 (0.001303%)
GCGTGCCTCGGCATCAGCGTGCTCCGGGCGTCGGCCTGTGGGCTCCCCATTCGACCCGTCTTGAAACACGGACCAAGGAGTCTGACATGTGTGCGAGTCA	10 (0.000192%)
GCGTGCTCCGGGCGTCGGCCTGTGGGCTCCCCATTCGACCCGTCTTGAAACACGGACCAAGGAGTCTGACATGTGTGCGAGTCAACGGGTGAGTAAACCC	11 (0.000211%)
GCTAACTAGCTACGTGGAGGCATCCCTTCACGGCCGGCTTCTTAGAGGGACTATGGCCGTTTAGGCCAAGGAAGTTTGAGGCAATAACAGGTCTGTGATG	31 (0.000594%)
GCTAATGTATCCAGAGCGTAGGCTTGCTTTGAGCACTCTAATTTCTTCAAAGTAACAGCGCCGGAGGCACGACCCGGCCAATTAAGACCAGGAGCGTATC	19 (0.000364%)
GCTAGTGTTCGTAGGCTCCATGCTCGCGCATCGAACTACCTACCACCTATCCTTCTCAGTTAATTCACGGGCGATGTTACGCTCGATGATGAGTTCCGGG	20 (0.000383%)
GCTCCAATAGCGTATATTTAAGTTGTTGCAGTTAAAAAGCTCGTAGTTGAACCTTGGGATGGGTCGGCCGGTCCGCCTTTGGTGTGCATTGGTCGGCTTG	13 (0.000249%)
GCTCCATGCTCGCGCATCGAACTACCTACCACCTATCCTTCTCAGTTAATTCACGGGCGATGTTACGCTCGATGATGAGTTCCGGGGCCTGTGTTTCGTA	4 (0.000077%)
GCTCCGGGCGTCGGCCTGTGGGCTCCCCATTCGACCCGTCTTGAAACACGGACCAAGGAGTCTGACATGTGTGCGAGTCAACGGGTGAGTAAACCCGTAA	10 (0.000192%)
GCTCGCGCATCGAACTACCTACCACCTATCCTTCTCAGTTAATTCACGGGCGATGTTACGCTCGATGATGAGTTCCGGGGCCTGTGTTTCGTACCTAATT	6 (0.000115%)
GCTCGTGTAAAGTTGGGAATTCGTTAAGGAGCTGTTGCTTTGTTAGTGTAGAAACACTTGTGTAGAATTGGGGATTGTTTTTTTTGGAGTGATTTAGGGG	5 (0.000096%)
GCTGATGCGGGACGGAAGCTGGTCTCCCGTGTGTTACCGCACGCGGTTGGCCTAAATCCGAGCCAAGGACGCCTGGAGCGTACCGACATGCGGTGGTGAA	6 (0.000115%)
GCTGGCGACGCATCATTCAAATTTCTGCCCTATCAACTTTCGATGGTAGGATAGTGGCCTACCATGGTGGTAACGGGTGACGGAGAATTAGGGTTCGATT	29 (0.000556%)
GCTGGGGCGGCACATCTGTTAAAAGATAACGCAGGTGTCCTAAGATGAGCTCAACGAGAACAGAAATCTCGTGTGGAACAAAAGGGTAAAAGCTCGTTTG	9 (0.000172%)
GCTGTCCCGAAGGTATCTCGCGCTTGTACGGCTTTGGCTCGGATTCGTCCGTCTTCTTTCTTCTTAGCCGAGTACTTCGGTAGATTAGTTGGAACGATTG	55 (0.001054%)
GCTGTCCCGAGTGTGAGCGAGGTGTGAGTGTCGCCCATGGGCATCGACACCTTGCGGCTAGGAACTGGAACGAGACGGGTAGCAAAGATTTCGAGTAGCA	66 (0.001265%)
GCTGTCCCGAGTGTGAGCGAGGTGTGAGTGTCGCCCATGGGCATCGACACCTTGCGGCTAGGAACTGGAACGAGACGGGTGGCAAAGATTTCGAGTAGCA	99 (0.001897%)
GCTGTTGCTTTGTTAGTGTAGAAACACTTGTGTAGAATTGGGGATTGTTTTTTTTGGAGTGATTTAGGGGAGGGTCGAATCTTAGCGACAAAGGGCTGAA	5 (0.000096%)
GCTTCCTTGCGCCTTACGGGTTTACTCACCCGTTGACTCGCACACATGTCAGACTCCTTGGTCCGTGTTTCAAGACGGGTCGAATGGGGAGCCCACAGGC	44 (0.000843%)
GCTTGTACGGCTTTGGCTCGGATTCGTCCGTCTTCTTTCTTCTTAGCCGAGTACTTCGGTAGATTAGTTGGAACGATTGATGATTTTGAGTTAATTGAAC	18 (0.000345%)
GCTTGTCTCAAAGATTAAGCCATGCATGTGTAAGTATGAACGAATTCAGACTGTGAAACTGCGAATGGCTCATTAAATCAGTTATAGTTTGTTTGATGGT	20 (0.000383%)
GCTTTCACGGTTCGTATTCGTACTGAAAATCAGAATCAAACGAGCTTTTACCCTTTTGTTCCACACGAGATTTCTGTTCTCGTTGAGCTCATCTTAGGAC	10 (0.000192%)
GCTTTGGCTCGGATTCGTCCGTCTTCTTTCTTCTTAGCCGAGTACTTCGGTAGATTAGTTGGAACGATTGATGATTTTGAGTTAATTGAACGTTCGGCGT	5 (0.000096%)
GCTTTGTTTTAATTAAACAGTCGGATTCCCCTTGTCCGTACCAGTTCTGAGCTGACTGTTCGACGCCCGGGGAAAGCTCCCGAGAGAGCCGTTCCCAGTC	16 (0.000307%)
GGAACCGGGACGTGGCGGTTGACGGCAACGTTAGGGAGTCCGGAGACGTCGGCGGGGGCCTCGGGAAGAGTTATCTTTTCTGTTTAACAGCCTGCCCACC	72 (0.001380%)
GGAAGTCGAAATCCGCTAAGGAGTGTGTAACAACTCACCTGCCGAATCAACTAGCCCCGAAAATGGATGGCGCTTAAGCGCGCGACCTATACCCGGCCGT	24 (0.000460%)
GGACAGTCGGGGGCATTCGTATTTCATAGTCAGAGGTGAAATTCTTGGATTTATGAAAGACGAACAACTGCGAAAGCATTTGCCAAGGATGTTTTCATTA	63 (0.001207%)
GGACATTGTCAGGTGGGGAGTTTGGCTGGGGCGGCACATCTGTTAAAAGATAACGCAGGTGTCCTAAGATGAGCTCAACGAGAACAGAAATCTCGTGTGG	183 (0.003507%)
GGACGGTGGTCATGGAAGTCGAAATCCGCTAAGGAGTGTGTAACAACTCACCTGCCGAATCAACTAGCCCCGAAAATGGATGGCGCTTAAGCGCGCGACC	50 (0.000958%)
GGACGTGGCGGTTGACGGCAACGTTAGGGAGTCCGGAGACGTCGGCGGGGGCCTCGGGAAGAGTTATCTTTTCTGTTTAACAGCCTGCCCACCCTGGAAA	3 (0.000057%)
GGAGACGTCGGCGGGGGCCTCGGGAAGAGTTATCTTTTCTGTTTAACAGCCTGCCCACCCTGGAAACGGCTCAGCCGGAGGTAGGGTCCAGCGGCTGGAA	19 (0.000364%)
GGAGCGACGGGCGGTGTGTACAAAGGGCAGGGACGTAGTCAACGCGAGCTGATGACTCGCGCTTACTAGGAATTCCTCGTTGAAGACCAACAATTGCAAT	54 (0.001035%)

GGAGCTGTTGCTTTGTTAGTGTAGAAACACTTGTGTAGAATTGGGGATTGTTTTTTTTGGAGTGATTTAGGGGAGGGTCGAATCTTAGCGACAAAGGGCT	7 (0.000134%)
GGAGGACATTGTCAGGTGGGGAGTTTGGCTGGGGCGGCACATCTGTTAAAAGATAACGCAGGTGTCCTAAGATGAGCTCAACGAGAACAGAAATCTCGTG	368 (0.007053%)
GGAGGGCAAGTCTGGTGCCAGCAGCCGCGGTAATTCCAGCTCCAATAGCGTATATTTAAGTTGTTGCAGTTAAAAAGCTCGTAGTTGAACCTTGGGATGG	85 (0.001629%)
GGAGTAATGATTAACAGGGACAGTCGGGGGCATTCGTATTTCATAGTCAGAGGTGAAATTCTTGGATTTATGAAAGACGAACAACTGCGAAAGCATTTGC	960 (0.018398%)
GGAGTCCGGAGACGTCGGCGGGGGCCTCGGGAAGAGTTATCTTTTCTGTTTAACAGCCTGCCCACCCTGGAAACGGCTCAGCCGGAGGTAGGGTCCAGCG	5 (0.000096%)
GGAGTTTGGCTGGGGCGGCACATCTGTTAAAAGATAACGCAGGTGTCCTAAGATGAGCTCAACGAGAACAGAAATCTCGTGTGGAACAAAAGGGTAAAAG	5 (0.000096%)
GGAGTTTTTTCAGCAGTTCTCGGACAAAAATTGCTGAGTGGCCGAGAAGAATGGGCGTGTCATGCGTGGGCTGACATGGATTCTTCGAGGCCTAGGGGTG	5 (0.000096%)
GGATAGCTAGTGTTCGTAGGCTCCATGCTCGCGCATCGAACTACCTACCACCTATCCTTCTCAGTTAATTCACGGGCGATGTTACGCTCGATGATGAGTT	33 (0.000632%)
GGATTTTCAAGGGCCGCCGGGGGCGCACCGGACACCACGCGACGTGCGGTGCTCTTCCAGCCGCTGGACCCTACCTCCGGCTGAGCCGTTTCCAGGGTGG	43 (0.000824%)
GGCAACGTTAGGGAGTCCGGAGACGTCGGCGGGGGCCTCGGGAAGAGTTATCTTTTCTGTTTAACAGCCTGCCCACCCTGGAAACGGCTCAGCCGGAGGT	8 (0.000153%)
GGCAAGTCTGGTGCCAGCAGCCGCGGTAATTCCAGCTCCAATAGCGTATATTTAAGTTGTTGCAGTTAAAAAGCTCGTAGTTGAACCTTGGGATGGGTCG	44 (0.000843%)
GGCAATTCCCCGCCACATCCTCTCAAACGCAATGGAAAGAGAGAAAGGACGAGGTCTTGACCGTCATCTTTTGCCCGAAGGACGGATGAGCTTTGGCGGG	64 (0.001227%)
GGCACATCTGTTAAAAGATAACGCAGGTGTCCTAAGATGAGCTCAACGAGAACAGAAATCTCGTGTGGAACAAAAGGGTAAAAGCTCGTTTGATTCTGAT	16 (0.000307%)
GGCACTCGGTCCTCCGGATTTTCAAGGGCCGCCGGGGGCGCACCGGACACCACGCGACGTGCGGTGCTCTTCCAGCCGCTGGACCCTACCTCCGGCTGAG	21 (0.000402%)
GGCAGAGCCCGCGTCGACCTTTTATCTAATAAATGCGTCCCTTCCATAAGTCGGGGTTTGTTGCACGTATTAGCTCTAGAATTACTACGGTTATCCGAGT	29 (0.000556%)
GGCATCAGCGTGCTCCGGGCGTCGGCCTGTGGGCTCCCCATTCGACCCGTCTTGAAACACGGACCAAGGAGTCTGACATGTGTGCGAGTCAACGGGTGAG	12 (0.000230%)
GGCATCGACACCTTGCGGCTAGGAACTGGAACGAGACGGGTGGCAAAGATTTCGAGTAGCACTTCATACTACCGTGGGTTTTTTAAACCTTCCGAGTTTT	6 (0.000115%)
GGCATTCGTATTTCATAGTCAGAGGTGAAATTCTTGGATTTATGAAAGACGAACAACTGCGAAAGCATTTGCCAAGGATGTTTTCATTAATCAAGAACGA	4 (0.000077%)
GGCCGCCGGGGGCGCACCGGACACCACGCGACGTGCGGTGCTCTTCCAGCCGCTGGACCCTACCTCCGGCTGAGCCGTTTCCAGGGTGGGCAGGCTGTTA	7 (0.000134%)
GGCCTCTGTGCTGGCGACGCATCATTCAAATTTCTGCCCTATCAACTTTCGATGGTAGGATAGTGGCCTACCATGGTGGTAACGGGTGACGGAGAATTAG	147 (0.002817%)
GGCCTGTGGGCTCCCCATTCGACCCGTCTTGAAACACGGACCAAGGAGTCTGACATGTGTGCGAGTCAACGGGTGAGTAAACCCGTAAGGCGCAAGGAAG	8 (0.000153%)
GGCGACGCATCATTCAAATTTCTGCCCTATCAACTTTCGATGGTAGGATAGTGGCCTACCATGGTGGTAACGGGTGACGGAGAATTAGGGTTCGATTCCG	29 (0.000556%)
GGCGACGTGGGTGGTTCGCCGCCCGCGACGTCGCGAGAAGTCCACTAAACCTTATCATTTAGAGGAAGGAGAAGTCGTAACAAGGTTTCCGTAGGTGAAC	348 (0.006669%)
GGCGAGACAAGGGTTCACATTTCGTTCATCACCCTTGGCCGGCTATCGAACAGCCGGACTCCCATCAAAAGATGGTTGCCAAGAACATCTTCGTTACGGT	12 (0.000230%)
GGCGAGACAAGGGTTCACATTTCGTTCATCACCCTTGGCCGGCTTTCGAACAGCCGGACTCCCATCAAAAGATGGTTGCCAAGAACATCTTCGTTACGGT	25 (0.000479%)
GGCGCACCGGACACCACGCGACGTGCGGTGCTCTTCCAGCCGCTGGACCCTACCTCCGGCTGAGCCGTTTCCAGGGTGGGCAGGCTGTTAAACAGAAAAG	11 (0.000211%)
GGCGGCACATCTGTTAAAAGATAACGCAGGTGTCCTAAGATGAGCTCAACGAGAACAGAAATCTCGTGTGGAACAAAAGGGTAAAAGCTCGTTTGATTCT	6 (0.000115%)
GGCGGTTGACGGCAACGTTAGGGAGTCCGGAGACGTCGGCGGGGGCCTCGGGAAGAGTTATCTTTTCTGTTTAACAGCCTGCCCACCCTGGAAACGGCTC	14 (0.000268%)
GGCGTAAGAATTGTATCCTTGTTAGAAGACACAAAGCCAA	55 (0.000422%)
GGCGTGCCTCGGCATCAGCGTGCTCCGGGCGTCGGCCTGTGGGCTCCCCATTCGACCCGTCTTGAAACACGGACCAAGGAGTCTGACATGTGTGCGAGTC	18 (0.000345%)
GGCTGGGGCGGCACATCTGTTAAAAGATAACGCAGGTGTCCTAAGATGAGCTCAACGAGAACAGAAATCTCGTGTGGAACAAAAGGGTAAAAGCTCGTTT	10 (0.000192%)
GGCTGTCCCGAGTGTGAGCGAGGTGTGAGTGTCGCCCATGGGCATCGACACCTTGCGGCTAGGAACTGGAACGAGACGGGTAGCAAAGATTTCGAGTAGC	19 (0.000364%)
GGCTGTCCCGAGTGTGAGCGAGGTGTGAGTGTCGCCCATGGGCATCGACACCTTGCGGCTAGGAACTGGAACGAGACGGGTGGCAAAGATTTCGAGTAGC	16 (0.000307%)
GGCTTTGGCTCGGATTCGTCCGTCTTCTTTCTTCTTAGCCGAGTACTTCGGTAGATTAGTTGGAACGATTGATGATTTTGAGTTAATTGAACGTTCGGCG	8 (0.000153%)
GGGACAGTCGGGGGCATTCGTATTTCATAGTCAGAGGTGAAATTCTTGGATTTATGAAAGACGAACAACTGCGAAAGCATTTGCCAAGGATGTTTTCATT	17 (0.000326%)
GGGACGTGGCGGTTGACGGCAACGTTAGGGAGTCCGGAGACGTCGGCGGGGGCCTCGGGAAGAGTTATCTTTTCTGTTTAACAGCCTGCCCACCCTGGAA	20 (0.000383%)
GGGAGTCCGGAGACGTCGGCGGGGGCCTCGGGAAGAGTTATCTTTTCTGTTTAACAGCCTGCCCACCCTGGAAACGGCTCAGCCGGAGGTAGGGTCCAGC	4 (0.000077%)
GGGAGTTTGGCTGGGGCGGCACATCTGTTAAAAGATAACGCAGGTGTCCTAAGATGAGCTCAACGAGAACAGAAATCTCGTGTGGAACAAAAGGGTAAAA	32 (0.000613%)
GGGCAAGTCTGGTGCCAGCAGCCGCGGTAATTCCAGCTCCAATAGCGTATATTTAAGTTGTTGCAGTTAAAAAGCTCGTAGTTGAACCTTGGGATGGGTC	148 (0.002836%)
GGGCAGAGCCCGCGTCGACCTTTTATCTAATAAATGCGTCCCTTCCATAAGTCGGGGTTTGTTGCACGTATTAGCTCTAGAATTACTACGGTTATCCGAG	53 (0.001016%)
GGGCATCGACACCTTGCGGCTAGGAACTGGAACGAGACGGGTGGCAAAGATTTCGAGTAGCACTTCATACTACCGTGGGTTTTTTAAACCTTCCGAGTTT	13 (0.000249%)
GGGCATTCGTATTTCATAGTCAGAGGTGAAATTCTTGGATTTATGAAAGACGAACAACTGCGAAAGCATTTGCCAAGGATGTTTTCATTAATCAAGAACG	29 (0.000556%)
GGGCCGCCGGGGGCGCACCGGACACCACGCGACGTGCGGTGCTCTTCCAGCCGCTGGACCCTACCTCCGGCTGAGCCGTTTCCAGGGTGGGCAGGCTGTT	9 (0.000172%)

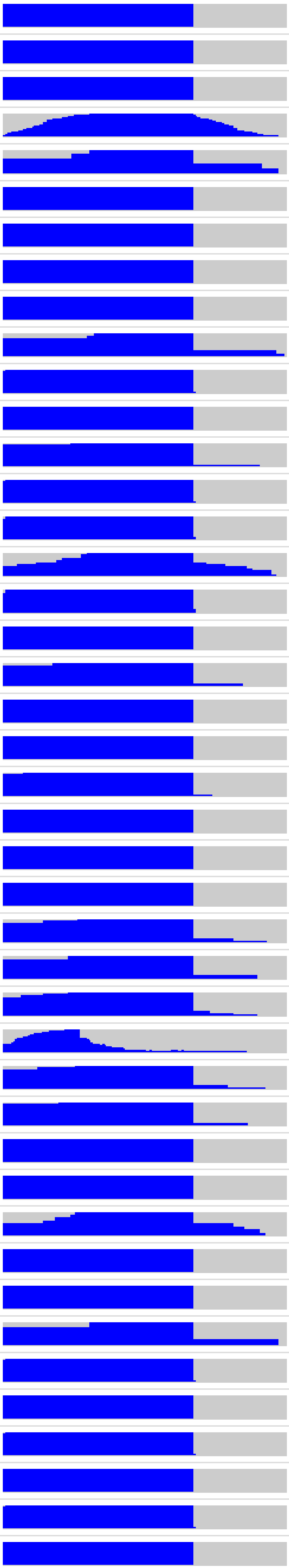

GGGCGCACCGGACACCACGCGACGTGCGGTGCTCTTCCAGCCGCTGGACCCTACCTCCGGCTGAGCCGTTTCCAGGGTGGGCAGGCTGTTAAACAGAAAA	4 (0.000077%)	
GGGCGGCACATCTGTTAAAAGATAACGCAGGTGTCCTAAGATGAGCTCAACGAGAACAGAAATCTCGTGTGGAACAAAAGGGTAAAAGCTCGTTTGATTC	7 (0.000134%)	
GGGCGGTGTGTACAAAGGGCAGGGACGTAGTCAACGCGAGCTGATGACTCGCGCTTACTAGGAATTCCTCGTTGAAGACCAACAATTGCAATGATCGATC	22 (0.000422%)	
GGGGAGTTTGGCTGGGGCGGCACATCTGTTAAAAGATAACGCAGGTGTCCTAAGATGAGCTCAACGAGAACAGAAATCTCGTGTGGAACAAAAGGGTAAA	31 (0.000594%)	
GGGGCATTCGTATTTCATAGTCAGAGGTGAAATTCTTGGATTTATGAAAGACGAACAACTGCGAAAGCATTTGCCAAGGATGTTTTCATTAATCAAGAAC	24 (0.000460%)	
GGGGCGCACCGGACACCACGCGACGTGCGGTGCTCTTCCAGCCGCTGGACCCTACCTCCGGCTGAGCCGTTTCCAGGGTGGGCAGGCTGTTAAACAGAAA	9 (0.000172%)	
GGGGCGGCACATCTGTTAAAAGATAACGCAGGTGTCCTAAGATGAGCTCAACGAGAACAGAAATCTCGTGTGGAACAAAAGGGTAAAAGCTCGTTTGATT	5 (0.000096%)	
GGGGGCATTCGTATTTCATAGTCAGAGGTGAAATTCTTGGATTTATGAAAGACGAACAACTGCGAAAGCATTTGCCAAGGATGTTTTCATTAATCAAGAA	17 (0.000326%)	
GGGGGCGCACCGGACACCACGCGACGTGCGGTGCTCTTCCAGCCGCTGGACCCTACCTCCGGCTGAGCCGTTTCCAGGGTGGGCAGGCTGTTAAACAGAA	21 (0.000402%)	
GGGGTTGAAATCGTCGACCAGGTCCGAGACTTCATCGACCGGGTCCGAGGATTCGTCGACCAGGACGGCCGGATGTCCGAGAAAAAAAAATGTTGCCGAA	5 (0.000096%)	
GGGTGGTTCGCCGCCCGCGACGTCGCGAGAAGTCCACTAAACCTTATCATTTAGAGGAAGGAGAAGTCGTAACAAGGTTTCCGTAGGTGAACCTGCGGAA	146 (0.002798%)	
GGGTGTCACAAATCGTCGTCCCTCACCATCCTTTGCTGATGCGGGACGGAAGCTGGTCTCCCGTGTGTTACCGCACGCGGTTGGCCTAAATCCGAGCCAA	3 (0.000057%)	
GGGTGTTGGTCGATTAAGACAGCAGGACGGTGGTCATGGAAGTCGAAATCCGCTAAGGAGTGTGTAACAACTCACCTGCCGAATCAACTAGCCCCGAAAA	17 (0.000326%)	
GGGTTGCGGTTTAAGTTGTTATACTCAATCATACACATGACAACAAGTCATATTCGACTCCAAAACACTAACCAACCTTCTTCTTGCTTCTCAAAGCTTT	453 (0.008682%)	
GGGTTTACTCACCCGTTGACTCGCACACATGTCAGACTCCTTGGTCCGTGTTTCAAGACGGGTCGAATGGGGAGCCCACAGGCCGACGCCCGGAGCACGC	8 (0.000153%)	
GGTAATTCCAGCTCCAATAGCGTATATTTAAGTTGTTGCAGTTAAAAAGCTCGTAGTTGAACCTTGGGATGGGTCGGCCGGTCCGCCTTTGGTGTGCATT	96 (0.001840%)	
GGTACGAAACACAGGCCCCGGAACTCATCATCGAGCGTAACATCGCCCGTGAATTAACTGAGAAGGATAGGTGGTAGGTAGTTCGATGCGCGAGCATGGA	6 (0.000115%)	
GGTAGGAGCGACGGGCGGTGTGTACAAAGGGCAGGGACGTAGTCAACGCGAGCTGATGACTCGCGCTTACTAGGAATTCCTCGTTGAAGACCAACAATTG	39 (0.000747%)	
GGTATCTCGCGCTTGTACGGCTTTGGCTCGGATTCGTCCGTCTTCTTTCTTCTTAGCCGAGTACTTCGGTAGATTAGTTGGAACGATTGATGATTTTGAG	18 (0.000345%)	
GGTCCTCCGGATTTTCAAGGGCCGCCGGGGGCGCACCGGACACCACGCGACGTGCGGTGCTCTTCCAGCCGCTGGACCCTACCTCCGGCTGAGCCGTTTC	6 (0.000115%)	
GGTCGACCAGGGGTTGAAATCGTCGACCAGGTCCGAGACTTCATCGACCGGGTCCGAGGATTCGTCGACCAGGACGGCCGGATGTCCGAGAAAAAAAAAT	800 (0.015332%)	
GGTCGATTAAGACAGCAGGACGGTGGTCATGGAAGTCGAAATCCGCTAAGGAGTGTGTAACAACTCACCTGCCGAATCAACTAGCCCCGAAAATGGATGG	43 (0.000824%)	
GGTCTTCAACGAGGAATTCCTAGTAAGCGCGAGTCATCAGCTCGCGTTGACTACGTCCCTGCCCTTTGTACACACCGCCCGTCGCTCCTACCGATTGAAT	22 (0.000422%)	
GGTGCCAGCAGCCGCGGTAATTCCAGCTCCAATAGCGTATATTTAAGTTGTTGCAGTTAAAAAGCTCGTAGTTGAACCTTGGGATGGGTCGGCCGGTCCG	9 (0.000172%)	
GGTGGTCATGGAAGTCGAAATCCGCTAAGGAGTGTGTAACAACTCACCTGCCGAATCAACTAGCCCCGAAAATGGATGGCGCTTAAGCGCGCGACCTATA	13 (0.000249%)	
GGTGGTTCGCCGCCCGCGACGTCGCGAGAAGTCCACTAAACCTTATCATTTAGAGGAAGGAGAAGTCGTAACAAGGTTTCCGTAGGTGAACCTGCGGAAG	22 (0.000422%)	
GGTGTCACAAATCGTCGTCCCTCACCATCCTTTGCTGATGCGGGACGGAAGCTGGTCTCCCGTGTGTTACCGCACGCGGTTGGCCTAAATCCGAGCCAAG	6 (0.000115%)	
GGTGTGAGTGTCGCCCATGGGCATCGACACCTTGCGGCTAGGAACTGGAACGAGACGGGTAGCAAAGATTTCGAGTAGCACTTCATACTACCGTGGGTTT	9 (0.000172%)	
GGTGTGAGTGTCGCCCATGGGCATCGACACCTTGCGGCTAGGAACTGGAACGAGACGGGTGGCAAAGATTTCGAGTAGCACTTCATACTACCGTGGGTTT	5 (0.000096%)	
GGTGTTGGTCGATTAAGACAGCAGGACGGTGGTCATGGAAGTCGAAATCCGCTAAGGAGTGTGTAACAACTCACCTGCCGAATCAACTAGCCCCGAAAAT	3 (0.000057%)	
GGTTCACATTTCGTTCATCACCCTTGGCCGGCTTTCGAACAGCCGGACTCCCATCAAAAGATGGTTGCCAAGAACATCTTCGTTACGGTTTGCTAATTCT	3 (0.000057%)	
GGTTCGAGTGTGAGCATGCCTGTCGGGACCCGAAAGATGGTGAACTATGCCTGAGCGGGGTAAAGCCAGAGGAAACTCTGGTGGAAGCCCGCAGCGATAC	3 (0.000057%)	
GGTTCGCCGCCCGCGACGTCGCGAGAAGTCCACTAAACCTTATCATTTAGAGGAAGGAGAAGTCGTAACAAGGTTTCCGTAGGTGAACCTGCGGAAGGAT	18 (0.000345%)	
GGTTCGTATTCGTACTGAAAATCAGAATCAAACGAGCTTTTACCCTTTTGTTCCACACGAGATTTCTGTTCTCGTTGAGCTCATCTTAGGACACCTGCGT	11 (0.000211%)	
GGTTGAAATCGTCGACCAGGTCCGAGACTTCATCGACCGGGTCCGAGGATTCGTCGACCAGGACGGCCGGATGTCCGAGAAAAAAAAATGTTGCCGAATA	17 (0.000326%)	
GGTTGACGGCAACGTTAGGGAGTCCGGAGACGTCGGCGGGGGCCTCGGGAAGAGTTATCTTTTCTGTTTAACAGCCTGCCCACCCTGGAAACGGCTCAGC	18 (0.000345%)	
GGTTGCGGTTTAAGTTCTTATACTCAATCATACACATGACATCAAGTCATATTCGACTCCAAAACACTAACCAACCTTCTTCTTGCTTCTCAAAGCTTTC	1203 (0.023055%)	
GGTTGCGGTTTAAGTTGTTATACTCAATCATACACATGACAACAAGTCATATTCGACTCCAAAACACTAACCAACCTTCTTCTTGCTTCTCAAAGCTTTC	21 (0.000402%)	
GGTTTAAGTTCTTATACTCAATCATACACATGACATCAAGTCATATTCGACTCCAAAACACTAACCAACCTTCTTCTTGCTTCTCAAAGCTTTCATGGTG	253 (0.004849%)	
GGTTTAAGTTGTTATACTCAATCATACACATGACAACAAGTCATATTCGACTCCAAAACACTAACCAACCTTCTTCTTGCTTCTCAAAGCTTTCATGGTG	116 (0.002223%)	
GGTTTACTCACCCGTTGACTCGCACACATGTCAGACTCCTTGGTCCGTGTTTCAAGACGGGTCGAATGGGGAGCCCACAGGCCGACGCCCGGAGCACGCT	24 (0.000460%)	
GTAAAGTTGGGAATTCGTTAAGGAGCTGTTGCTTTGTTAGTGTAGAAACACTTGTGTAGAATTGGGGATTGTTTTTTTTGGAGTGATTTAGGGGAGGGTC	46 (0.000882%)	
GTAAGAATTGTATCCTTGTTAGAAGACACAAAGCCAAAGA	320 (0.002453%)	

GTAATTCCAGCTCCAATAGCGTATATTTAAGTTGTTGCAGTTAAAAAGCTCGTAGTTGAACCTTGGGATGG<br>GTCGGCCGGTCCGCCTTTGGTGTGCATTG	14 (0.000268%)	
GTACGAAACACAGGCCCCGGAACTCATCATCGAGCGTAACATCGCCCGTGAATTAACTGAGAAGGATAGGT<br>GGTAGGTAGTTCGATGCGCGAGCATGGAG	16 (0.000307%)	
GTACGGCTTTGGCTCGGATTCGTCCGTCTTCTTTCTTCTTAGCCGAGTACTTCGGTAGATTAGTTGGAACG<br>ATTGATGATTTTGAGTTAATTGAACGTTC	6 (0.000115%)	
GTAGGAGCGACGGGCGGTGTGTACAAAGGGCAGGGACGTAGTCAACGCGAGCTGATGACTCGCGCTTACTA<br>GGAATTCCTCGTTGAAGACCAACAATTGC	100 (0.001916%)	
GTAGGCTCCATGCTCGCGCATCGAACTACCTACCACCTATCCTTCTCAGTTAATTCACGGGCGATGTTACG<br>CTCGATGATGAGTTCCGGGGCCTGTGTTT	13 (0.000249%)	
GTAGTCATATGCTTGTCTCAAAGATTAAGCCATGCATGTGTAAGTATGAACGAATTCAGACTGTGAAACTG<br>CGAATGGCTCATTAAATCAGTTATAGTTT	79 (0.001514%)	
GTATAAGAACTTAAACCGCAACCGCATCTTATAAGCCTAAGTAGTGTTTCCTTGTTAGAAGACACAAAGCC<br>AAAGACTCATATGGACTTTGGCTACACCA	6 (0.000115%)	
GTATATTTAAGTTGTTGCAGTTAAAAAGCTCGTAGTTGAACCTTGGGATGGGTCGGCCGGTCCGCCTTTGG<br>TGTGCATTGGTCGGCTTGTCCCTTCGGTC	23 (0.000441%)	
GTATCCAGAGCGTAGGCTTGCTTTGAGCACTCTAATTTCTTCAAAGTAACAGCGCCGGAGGCACGACCCGG<br>CCAATTAAGACCAGGAGCGTATCGCCGAC	24 (0.000460%)	
GTATCCTTGTTAGAAGACACAAAGCCAAAGACTCATATGGACTTTGGCTACACCATGAAAGCTTTGAGAAG<br>CAAGAAGAAGGTTGGTTAGTGTTTTGGAG	17 (0.000326%)	
GTATGATTGAGTATAAGAACTTAAACCGCAACCGCATCTTATAAGCCTAAGTAGTGTTTCCTTGTTAGAAG<br>ACACAAAGCCAAAGACTCATATGGACTTT	46 (0.000882%)	
GTATTCGTACTGAAAATCAGAATCAAACGAGCTTTTACCCTTTTGTTCCACACGAGATTTCTGTTCTCGTT<br>GAGCTCATCTTAGGACACCTGCGTTATCT	5 (0.000096%)	
GTCACAAATCGTCGTCCCTCACCATCCTTTGCTGATGCGGGACGGAAGCTGGTCTCCCGTGTGTTACCGCA<br>CGCGGTTGGCCTAAATCCGAGCCAAGGAC	13 (0.000249%)	
GTCAGGTGGGGAGTTTGGCTGGGGCGGCACATCTGTTAAAAGATAACGCAGGTGTCCTAAGATGAGCTCAA<br>CGAGAACAGAAATCTCGTGTGGAACAAAA	8 (0.000153%)	
GTCATATGCTTGTCTCAAAGATTAAGCCATGCATGTGTAAGTATGAACGAATTCAGACTGTGAAACTGCGA<br>ATGGCTCATTAAATCAGTTATAGTTTGTT	38 (0.000728%)	
GTCATATTCGACTCCAAAACACTAACCAACCTTCTTCTTGCTTCTCAAAGCTTTCATGGTGTAGCCAAAGT<br>CCATATGAGTCTTTGGCTTTGTGTCTTCT	188 (0.003603%)	
GTCCCGAAGGTATCTCGCGCTTGTACGGCTTTGGCTCGGATTCGTCCGTCTTCTTTCTTCTTAGCCGAGTA<br>CTTCGGTAGATTAGTTGGAACGATTGATG	10 (0.000192%)	
GTCCCGAGTGTGAGCGAGGTGTGAGTGTCGCCCATGGGCATCGACACCTTGCGGCTAGGAACTGGAACGAG<br>ACGGGTAGCAAAGATTTCGAGTAGCACTT	8 (0.000153%)	
GTCCCGAGTGTGAGCGAGGTGTGAGTGTCGCCCATGGGCATCGACACCTTGCGGCTAGGAACTGGAACGAG<br>ACGGGTGGCAAAGATTTCGAGTAGCACTT	12 (0.000230%)	
GTCCCTCACCATCCTTTGCTGATGCGGGACGGAAGCTGGTCTCCCGTGTGTTACCGCACGCGGTTGGCCTA<br>AATCCGAGCCAAGGACGCCTGGAGCGTAC	11 (0.000211%)	
GTCCGGAGACGTCGGCGGGGGCCTCGGGAAGAGTTATCTTTTCTGTTTAACAGCCTGCCCACCCTGGAAAC<br>GGCTCAGCCGGAGGTAGGGTCCAGCGGCT	4 (0.000077%)	
GTCCTCCGGATTTTCAAGGGCCGCCGGGGGCGCACCGGACACCACGCGACGTGCGGTGCTCTTCCAGCCGC<br>TGGACCCTACCTCCGGCTGAGCCGTTTCC	4 (0.000077%)	
GTCGAATATGACTTGATGTCATGTGTATGATTGAGTATAA	466 (0.003572%)	
GTCGACCAGGGGTTGAAATCGTCGACCAGGTCCGAGACTTCATCGACCGGGTCCGAGGATTCGTCGACCAG<br>GACGGCCGGATGTCCGAGAAAAAAAAATG	58 (0.001112%)	
GTCGAGTTATCATGAATCATCAGAGCAACGGGCAGAGCCCGCGTCGACCTTTTATCTAATAAATGCGTCCC<br>TTCCATAAGTCGGGGTTTGTTGCACGTAT	443 (0.008490%)	
GTCGATTAAGACAGCAGGACGGTGGTCATGGAAGTCGAAATCCGCTAAGGAGTGTGTAACAACTCACCTGC<br>CGAATCAACTAGCCCCGAAAATGGATGGC	27 (0.000517%)	
GTCGCCCATGGGCATCGACACCTTGCGGCTAGGAACTGGAACGAGACGGGTGGCAAAGATTTCGAGTAGCA<br>CTTCATACTACCGTGGGTTTTTTAAACCT	18 (0.000345%)	
GTCGGGGGCATTCGTATTTCATAGTCAGAGGTGAAATTCTTGGATTTATGAAAGACGAACAACTGCGAAAG<br>CATTTGCCAAGGATGTTTTCATTAATCAA	7 (0.000134%)	
GTCGTCCCTCACCATCCTTTGCTGATGCGGGACGGAAGCTGGTCTCCCGTGTGTTACCGCACGCGGTTGGC<br>CTAAATCCGAGCCAAGGACGCCTGGAGCG	33 (0.000632%)	
GTCTCAAAGATTAAGCCATGCATGTGTAAGTATGAACGAATTCAGACTGTGAAACTGCGAATGGCTCATTA<br>AATCAGTTATAGTTTGTTTGATGGTAACT	5 (0.000096%)	
GTCTGGTGCCAGCAGCCGCGGTAATTCCAGCTCCAATAGCGTATATTTAAGTTGTTGCAGTTAAAAAGCTC<br>GTAGTTGAACCTTGGGATGGGTCGGCCGG	20 (0.000383%)	
GTCTTCAACGAGGAATTCCTAGTAAGCGCGAGTCATCAGCTCGCGTTGACTACGTCCCTGCCCTTTGTACA<br>CACCGCCCGTCGCTCCTACCGATTGAATG	9 (0.000172%)	
GTGAGCGAGGTGTGAGTGTCGCCCATGGGCATCGACACCTTGCGGCTAGGAACTGGAACGAGACGGGTGGC<br>AAAGATTTCGAGTAGCACTTCATACTACC	22 (0.000422%)	
GTGAGCGGCACTCGGTCCTCCGGATTTTCAAGGGCCGCCGGGGGCGCACCGGACACCACGCGACGTGCGGT<br>GCTCTTCCAGCCGCTGGACCCTACCTCCG	377 (0.007225%)	
GTGAGTGTCGCCCATGGGCATCGACACCTTGCGGCTAGGAACTGGAACGAGACGGGTGGCAAAGATTTCGA<br>GTAGCACTTCATACTACCGTGGGTTTTTT	33 (0.000632%)	
GTGCCAGCAGCCGCGGTAATTCCAGCTCCAATAGCGTATATTTAAGTTGTTGCAGTTAAAAAGCTCGTAGT<br>TGAACCTTGGGATGGGTCGGCCGGTCCGC	31 (0.000594%)	
GTGCCTCGGCATCAGCGTGCTCCGGGCGTCGGCCTGTGGGCTCCCCATTCGACCCGTCTTGAAACACGGAC<br>CAAGGAGTCTGACATGTGTGCGAGTCAAC	15 (0.000287%)	
GTGCTCCGGGCGTCGGCCTGTGGGCTCCCCATTCGACCCGTCTTGAAACACGGACCAAGGAGTCTGACATG<br>TGTGCGAGTCAACGGGTGAGTAAACCCGT	6 (0.000115%)	
GTGCTGGCGACGCATCATTCAAATTTCTGCCCTATCAACTTTCGATGGTAGGATAGTGGCCTACCATGGTG<br>GTAACGGGTGACGGAGAATTAGGGTTCGA	28 (0.000537%)	
GTGGCGGTTGACGGCAACGTTAGGGAGTCCGGAGACGTCGGCGGGGGCCTCGGGAAGAGTTATCTTTTCTG<br>TTTAACAGCCTGCCCACCCTGGAAACGGC	18 (0.000345%)	
GTGGGGAGTTTGGCTGGGGCGGCACATCTGTTAAAAGATAACGCAGGTGTCCTAAGATGAGCTCAACGAGA<br>ACAGAAATCTCGTGTGGAACAAAAGGGTA	13 (0.000249%)	
GTGGGTGGTTCGCCGCCCGCGACGTCGCGAGAAGTCCACTAAACCTTATCATTTAGAGGAAGGAGAAGTCG<br>TAACAAGGTTTCCGTAGGTGAACCTGCGG	77 (0.001476%)	
GTGGTCATGGAAGTCGAAATCCGCTAAGGAGTGTGTAACAACTCACCTGCCGAATCAACTAGCCCCGAAAA<br>TGGATGGCGCTTAAGCGCGCGACCTATAC	13 (0.000249%)	

GTGGTTCGCCGCCCGCGACGTCGCGAGAAGTCCACTAAACCTTATCATTTAGAGGAAGGAGAAGTCGTAACAAGGTTTCCGTAGGTGAACCTGCGGAAGG	12 (0.000230%)
GTGTAAAGTTGGGAATTCGTTAAGGAGCTGTTGCTTTGTTAGTGTAGAAACACTTGTGTAGAATTGGGGATTGTTTTTTTTGGAGTGATTTAGGGGAGGG	38 (0.000728%)
GTGTATCCTTGTTAGAAGACACAAAGCCAAAGACTCATAT	66 (0.000506%)
GTGTATGATTGAGTATAAGAACTTAAACCGCAACCGCATCTTATAAGCCTAAGTAGTGTTTCCTTGTTAGAAGACACAAAGCCAAAGACTCATATGGACT	6 (0.000115%)
GTGTCACAAATCGTCGTCCCTCACCATCCTTTGCTGATGCGGGACGGAAGCTGGTCTCCCGTGTGTTACCGCACGCGGTTGGCCTAAATCCGAGCCAAGG	6 (0.000115%)
GTGTCGCCCATGGGCATCGACACCTTGCGGCTAGGAACTGGAACGAGACGGGTGGCAAAGATTTCGAGTAGCACTTCATACTACCGTGGGTTTTTTAAAC	10 (0.000192%)
GTGTGAGCATGCCTGTCGGGACCCGAAAGATGGTGAACTATGCCTGAGCGGGGTAAAGCCAGAGGAAACTCTGGTGGAAGCCCGCAGCGATACTGACGTG	4 (0.000077%)
GTGTGAGCGAGGTGTGAGTGTCGCCCATGGGCATCGACACCTTGCGGCTAGGAACTGGAACGAGACGGGTAGCAAAGATTTCGAGTAGCACTTCATACTA	19 (0.000364%)
GTGTGAGCGAGGTGTGAGTGTCGCCCATGGGCATCGACACCTTGCGGCTAGGAACTGGAACGAGACGGGTGGCAAAGATTTCGAGTAGCACTTCATACTA	29 (0.000556%)
GTGTGAGTGTCGCCCATGGGCATCGACACCTTGCGGCTAGGAACTGGAACGAGACGGGTAGCAAAGATTTCGAGTAGCACTTCATACTACCGTGGGTTTT	11 (0.000211%)
GTGTGAGTGTCGCCCATGGGCATCGACACCTTGCGGCTAGGAACTGGAACGAGACGGGTGGCAAAGATTTCGAGTAGCACTTCATACTACCGTGGGTTTT	15 (0.000287%)
GTGTTCGTAGGCTCCATGCTCGCGCATCGAACTACCTACCACCTATCCTTCTCAGTTAATTCACGGGCGATGTTACGCTCGATGATGAGTTCCGGGGCCT	8 (0.000153%)
GTGTTGGTCGATTAAGACAGCAGGACGGTGGTCATGGAAGTCGAAATCCGCTAAGGAGTGTGTAACAACTCACCTGCCGAATCAACTAGCCCCGAAAATG	6 (0.000115%)
GTGTTTCCTTGTTAGAAGACACAAAGCCAAAGACTCATATGGACTTTGGCTACACCATGAAAGCTTTGAGAAGCAAGAAGAAGGTTGGTTAGTGTTTTGG	25 (0.000479%)
GTGTTTTGGAGTCGAATATGACTTGATGTCATGTGTATGA	209 (0.001602%)
GTTAAAAGATAACGCAGGTGTCCTAAGATGAGCTCAACGAGAACAGAAATCTCGTGTGGAACAAAAGGGTAAAAGCTCGTTTGATTCTGATTTTCAGTAC	30 (0.000575%)
GTTAAGGAGCTGTTGCTTTGTTAGTGTAGAAACACTTGTGTAGAATTGGGGATTGTTTTTTTTGGAGTGATTTAGGGGAGGGTCGAATCTTAGCGACAAA	47 (0.000901%)
GTTAGAAGACACAAAGCCAAAGACTCATATGGACTTTGGCTACACCATGAAAGCTTTGAGAAGCAAGAAGAAGGTTGGTTAGTGTTTTGGAGTCGAATAT	73 (0.001399%)
GTTAGGGAGTCCGGAGACGTCGGCGGGGGCCTCGGGAAGAGTTATCTTTTCTGTTTAACAGCCTGCCCACCCTGGAAACGGCTCAGCCGGAGGTAGGGTC	13 (0.000249%)
GTTAGTGTTTTGGAGTCGAATATGACTTGATGTCATGTGT	15393 (0.118000%)
GTTATCATGAATCATCAGAGCAACGGGCAGAGCCCGCGTCGACCTTTTATCTAATAAATGCGTCCCTTCCATAAGTCGGGGTTTGTTGCACGTATTAGCT	264 (0.005059%)
GTTATCCCATGCTAATGTATCCAGAGCGTAGGCTTGCTTTGAGCACTCTAATTTCTTCAAAGTAACAGCGCCGGAGGCACGACCCGGCCAATTAAGACCA	49 (0.000939%)
GTTCAATATGACAAACCCATGCCAAGTAAAGAGAAAATGAAAACTGGTGATTGTTGCGGAAATCGTCCAGGATTCCTCGACCAGGACTTGAAATCGTCGA	23 (0.000441%)
GTTCACATTTCGTTCATCACCCTTGGCCGGCTATCGAACAGCCGGACTCCCATCAAAAGATGGTTGCCAAGAACATCTTCGTTACGGTTTGCTAATTCTC	12 (0.000230%)
GTTCACATTTCGTTCATCACCCTTGGCCGGCTTTCGAACAGCCGGACTCCCATCAAAAGATGGTTGCCAAGAACATCTTCGTTACGGTTTGCTAATTCTC	19 (0.000364%)
GTTCATCACCCTTGGCCGGCTTTCGAACAGCCGGACTCCCATCAAAAGATGGTTGCCAAGAACATCTTCGTTACGGTTTGCTAATTCTCGGAATAACATC	6 (0.000115%)
GTTCGATTAGTCTTTCGCCCCTATACCCAAGTCAGACGAACGATTTGCACGTCAGTATCGCTGCGGGCTTCCACCAGAGTTTCCTCTGGCTTTACCCCGC	775 (0.014853%)
GTTCGCCGCCCGCGACGTCGCGAGAAGTCCACTAAACCTTATCATTTAGAGGAAGGAGAAGTCGTAACAAGGTTTCCGTAGGTGAACCTGCGGAAGGATC	33 (0.000632%)
GTTCGTAGGCTCCATGCTCGCGCATCGAACTACCTACCACCTATCCTTCTCAGTTAATTCACGGGCGATGTTACGCTCGATGATGAGTTCCGGGGCCTGT	8 (0.000153%)
GTTCGTATTCGTACTGAAAATCAGAATCAAACGAGCTTTTACCCTTTTGTTCCACACGAGATTTCTGTTCTCGTTGAGCTCATCTTAGGACACCTGCGTT	12 (0.000230%)
GTTCTCGGACAAAAATTGCTGAGTGGCCGAGAAGAATGGGCGTGTCATGCGTGGGCTGACATGGATTCTTCGAGGCCTAGGGGTGGCGGTATATAACTTG	3 (0.000057%)
GTTCTTATACTCAATCATACACATGACATCAAGTCATATTCGACTCCAAAACACTAACCAACCTTCTTCTTGCTTCTCAAAGCTTTCATGGTGTAGCCAA	92 (0.001763%)
GTTGAAATCGTCGACCAGGTCCGAGACTTCATCGACCGGGTCCGAGGATTCGTCGACCAGGACGGCCGGATGTCCGAGAAAAAAAAATGTTGCCGAATAA	41 (0.000786%)
GTTGACGGCAACGTTAGGGAGTCCGGAGACGTCGGCGGGGGCCTCGGGAAGAGTTATCTTTTCTGTTTAACAGCCTGCCCACCCTGGAAACGGCTCAGCC	41 (0.000786%)
GTTGACTCGCACACATGTCAGACTCCTTGGTCCGTGTTTCAAGACGGGTCGAATGGGGAGCCCACAGGCCGACGCCCGGAGCACGCTGATGCCGAGGCAC	54 (0.001035%)
GTTGCGGTTTAAGTTCTTATACTCAATCATACACATGACATCAAGTCATATTCGACTCCAAAACACTAACCAACCTTCTTCTTGCTTCTCAAAGCTTTCA	292 (0.005596%)
GTTGCGGTTTAAGTTGTTATACTCAATCATACACATGACAACAAGTCATATTCGACTCCAAAACACTAACCAACCTTCTTCTTGCTTCTCAAAGCTTTCA	146 (0.002798%)
GTTGGTCGATTAAGACAGCAGGACGGTGGTCATGGAAGTCGAAATCCGCTAAGGAGTGTGTAACAACTCACCTGCCGAATCAACTAGCCCCGAAAATGGA	15 (0.000287%)
GTTGGTCTTCAACGAGGAATTCCTAGTAAGCGCGAGTCATCAGCTCGCGTTGACTACGTCCCTGCCCTTTGTACACACCGCCCGTCGCTCCTACCGATTG	175 (0.003354%)
GTTGTTATACTCAATCATACACATGACAACAAGTCATATTCGACTCCAAAACACTAACCAACCTTCTTCTTGCTTCTCAAAGCTTTCATGGTGTAGCCAA	14 (0.000268%)
GTTGTTATACTCAATCATACACATGACATCAAGTCATATT	240 (0.001840%)
GTTTAAGTTCTTATACTCAATCATACACATGACATCAAGTCATATTCGACTCCAAAACACTAACCAACCTTCTTCTTGCTTCTCAAAGCTTTCATGGTGT	348 (0.006669%)
GTTTAAGTTGTTATACTCAATCATACACATGACAACAAGTCATATTCGACTCCAAAACACTAACCAACCTTCTTCTTGCTTCTCAAAGCTTTCATGGTGT	127 (0.002434%)

GTTTACTCACCCGTTGACTCGCACACATGTCAGACTCCTTGGTCCGTGTTTCAAGACGGGTCGAATGGGGAGCCCACAGGCCGACGCCCGGAGCACGCTG	19 (0.000364%)
GTTTGGCTGGGGCGGCACATCTGTTAAAAGATAACGCAGGTGTCCTAAGATGAGCTCAACGAGAACAGAAATCTCGTGTGGAACAAAAGGGTAAAAGCTC	33 (0.000632%)
GTTTTAATTAAACAGTCGGATTCCCCTTGTCCGTACCAGTTCTGAGCTGACTGTTCGACGCCCGGGGAAAGCTCCCGAGAGAGCCGTTCCCAGTCCGTCC	14 (0.000268%)
GTTTTGGAGTCGAATATGACTTGATGTCATGTGTATGATT	459 (0.003519%)
GTTTTTTCAGCAGTTCTCGGACAAAAATTGCTGAGTGGCCGAGAAGAATGGGCGTGTCATGCGTGGGCTGACATGGATTCTTCGAGGCCTAGGGGTGGCG	16 (0.000307%)
TAAAAGATAACGCAGGTGTCCTAAGATGAGCTCAACGAGAACAGAAATCTCGTGTGGAACAAAAGGGTAAAAGCTCGTTTGATTCTGATTTTCAGTACGA	19 (0.000364%)
TAAAAGTGTCGGAGTTTTTTCAGCAGTTCTCGGACAAAAATTGCTGAGTGGCCGAGAAGAATGGGCGTGTCATGCGTGGGCTGACATGGATTCTTCGAGG	8 (0.000153%)
TAAAGGCGTAAGAATTGTATCCTTGTTAGAAGACACAAAG	572 (0.004385%)
TAAAGTTGGGAATTCGTTAAGGAGCTGTTGCTTTGTTAGTGTAGAAACACTTGTGTAGAATTGGGGATTGTTTTTTTTGGAGTGATTTAGGGGAGGGTCG	3 (0.000057%)
TAAATACGGGCGAGAGACCGATAGCGAACAAGTACCGCGAGGTAAAGATGAAAAGGACTTTGAAAAGAGAGTCAAAGAGTGCTTGAAATTGTCGGGAGGG	24 (0.000460%)
TAACAGGGACAGTCGGGGGCATTCGTATTTCATAGTCAGAGGTGAAATTCTTGGATTTATGAAAGACGAACAACTGCGAAAGCATTTGCCAAGGATGTTT	61 (0.001169%)
TAACGGCGTGCCTCGGCATCAGCGTGCTCCGGGCGTCGGCCTGTGGGCTCCCCATTCGACCCGTCTTGAAACACGGACCAAGGAGTCTGACATGTGTGCG	41 (0.000786%)
TAACTAGCTACGTGGAGGCATCCCTTCACGGCCGGCTTCTTAGAGGGACTATGGCCGTTTAGGCCAAGGAAGTTTGAGGCAATAACAGGTCTGTGATGCC	33 (0.000632%)
TAAGAACTTAAACCGCAACCGCATCTTATAAGCCTAAGTAGTGTTTCCTTGTTAGAAGACACAAAGCCAAAGACTCATATGGACTTTGGCTACACCATGA	38 (0.000728%)
TAAGAATTGTATCCTTGTTAGAAGACACAAAGCCAAAGACTCATATGGACTTTGGCTACACCATGAAAGCTTTGAGAAGCAAGAAGAAGGTTGGTTAGTG	1020 (0.019548%)
TAAGACAGCAGGACGGTGGTCATGGAAGTCGAAATCCGCTAAGGAGTGTGTAACAACTCACCTGCCGAATCAACTAGCCCCGAAAATGGATGGCGCTTAA	45 (0.000862%)
TAAGGAGCTGTTGCTTTGTTAGTGTAGAAACACTTGTGTAGAATTGGGGATTGTTTTTTTTGGAGTGATTTAGGGGAGGGTCGAATCTTAGCGACAAAGG	26 (0.000498%)
TAAGTAGTGTTTCCTTGTTAGAAGACACAAAGCCAAAGACTCATATGGACTTTGGCTACACCATGAAAGCTTTGAGAAGCAAGAAGAAGGTTGGTTAGTG	41 (0.000786%)
TAAGTTCTTATACTCAATCATACACATGACATCAAGTCATATTCGACTCCAAAACACTAACCAACCTTCTTCTTGCTTCTCAAAGCTTTCATGGTGTAGC	23 (0.000441%)
TAAGTTGTTATACTCAATCATACACATGACAACAAGTCATATTCGACTCCAAAACACTAACCAACCTTCTTCTTGCTTCTCAAAGCTTTCATGGTGTAGC	15 (0.000287%)
TAAGTTGTTATACTCAATCATACACATGACATCAAGTCAT	9364 (0.071783%)
TAATGTATCCAGAGCGTAGGCTTGCTTTGAGCACTCTAATTTCTTCAAAGTAACAGCGCCGGAGGCACGACCCGGCCAATTAAGACCAGGAGCGTATCGC	5 (0.000096%)
TAATTCCAGCTCCAATAGCGTATATTTAAGTTGTTGCAGTTAAAAAGCTCGTAGTTGAACCTTGGGATGGGTCGGCCGGTCCGCCTTTGGTGTGCATTGG	10 (0.000192%)
TACACATGACATCAAGTCATATTCGACTCCAAAACACTAACCAACCTTCTTCTTGCTTCTCAAAGCTTTCATGGTGTAGCCAAAGTCCATATGAGTCTTT	17 (0.000326%)
TACATTTTATCGGTCGCTCTTGTCCGGAAGCTGTAGATGACCCAAAGTCCATATAGCGACCCCAGGTCAGGCGGGATTACCCGCTGAGTTTAAGCATATC	5 (0.000096%)
TACCTACCACCTATCCTTCTCAGTTAATTCACGGGCGATGTTACGCTCGATGATGAGTTCCGGGGCCTGTGTTTCGTACCTAATTTGAAGGAATTGTTGA	4 (0.000077%)
TACGAAACACAGGCCCCGGAACTCATCATCGAGCGTAACATCGCCCGTGAATTAACTGAGAAGGATAGGTGGTAGGTAGTTCGATGCGCGAGCATGGAGC	10 (0.000192%)
TACGGGTTTACTCACCCGTTGACTCGCACACATGTCAGACTCCTTGGTCCGTGTTTCAAGACGGGTCGAATGGGGAGCCCACAGGCCGACGCCCGGAGCA	4 (0.000077%)
TACTCAATCATACACATGACATCAAGTCATATTCGACTCCAAAACACTAACCAACCTTCTTCTTGCTTCTCAAAGCTTTCATGGTGTAGCCAAAGTCCAT	27 (0.000517%)
TACTCACCCGTTGACTCGCACACATGTCAGACTCCTTGGTCCGTGTTTCAAGACGGGTCGAATGGGGAGCCCACAGGCCGACGCCCGGAGCACGCTGATG	3 (0.000057%)
TAGAAGACACAAAGCCAAAGACTCATATGGACTTTGGCTACACCATGAAAGCTTTGAGAAGCAAGAAGAAGGTTGGTTAGTGTTTTGGAGTCGAATATGA	38 (0.000728%)
TAGCTAGTGTTCGTAGGCTCCATGCTCGCGCATCGAACTACCTACCACCTATCCTTCTCAGTTAATTCACGGGCGATGTTACGCTCGATGATGAGTTCCG	6 (0.000115%)
TAGGAGCGACGGGCGGTGTGTACAAAGGGCAGGGACGTAGTCAACGCGAGCTGATGACTCGCGCTTACTAGGAATTCCTCGTTGAAGACCAACAATTGCA	11 (0.000211%)
TAGGCGAGACAAGGGTTCACATTTCGTTCATCACCCTTGGCCGGCTTTCGAACAGCCGGACTCCCATCAAAAGATGGTTGCCAAGAACATCTTCGTTACG	4 (0.000077%)
TAGGCTCCATGCTCGCGCATCGAACTACCTACCACCTATCCTTCTCAGTTAATTCACGGGCGATGTTACGCTCGATGATGAGTTCCGGGGCCTGTGTTTC	4 (0.000077%)
TAGGCTGTCCCGAAGGTATCTCGCGCTTGTACGGCTTTGGCTCGGATTCGTCCGTCTTCTTTCTTCTTAGCCGAGTACTTCGGTAGATTAGTTGGAACGA	1761 (0.033749%)
TAGGCTGTCCCGAGTGTGAGCGAGGTGTGAGTGTCGCCCATGGGCATCGACACCTTGCGGCTAGGAACTGGAACGAGACGGGTGGCAAAGATTTCGAGTA	40 (0.000767%)
TAGGGAGTCCGGAGACGTCGGCGGGGGCCTCGGGAAGAGTTATCTTTTCTGTTTAACAGCCTGCCCACCCTGGAAACGGCTCAGCCGGAGGTAGGGTCCA	14 (0.000268%)
TAGGTACGAAACACAGGCCCCGGAACTCATCATCGAGCGTAACATCGCCCGTGAATTAACTGAGAAGGATAGGTGGTAGGTAGTTCGATGCGCGAGCATG	21 (0.000402%)
TAGTCATATGCTTGTCTCAAAGATTAAGCCATGCATGTGTAAGTATGAACGAATTCAGACTGTGAAACTGCGAATGGCTCATTAAATCAGTTATAGTTTG	46 (0.000882%)
TAGTGTTCGTAGGCTCCATGCTCGCGCATCGAACTACCTACCACCTATCCTTCTCAGTTAATTCACGGGCGATGTTACGCTCGATGATGAGTTCCGGGGC	11 (0.000211%)
TAGTGTTTCCTTGTTAGAAGACACAAAGCCAAAGACTCATATGGACTTTGGCTACACCATGAAAGCTTTGAGAAGCAAGAAGAAGGTTGGTTAGTGTTTT	19 (0.000364%)
TAGTGTTTTGGAGTCGAATATGACTTGATGTCATGTGTAT	277 (0.002123%)

TATAAGAACTTAAACCGCAACCGCATCTTATAAGCCTAAGTAGTGTTTCCTTGTTAGAAGACACAAAGCCAAAGACTCATATGGACTTTGGCTACACCAT	28 (0.000537%)
TATACTCAATCATACACATGACATCAAGTCATATTCGACTCCAAAACACTAACCAACCTTCTTCTTGCTTCTCAAAGCTTTCATGGTGTAGCCAAAGTCC	36 (0.000690%)
TATCAACTTTCGATGGTAGGATAGTGGCCTACCATGGTGGTAACGGGTGACGGAGAATTAGGGTTCGATTCCGGAGAGGGAGCCTGAGAAACGGCTACCA	12 (0.000230%)
TATCATGAATCATCAGAGCAACGGGCAGAGCCCGCGTCGACCTTTTATCTAATAAATGCGTCCCTTCCATAAGTCGGGGTTTGTTGCACGTATTAGCTCT	66 (0.001265%)
TATCCAGAGCGTAGGCTTGCTTTGAGCACTCTAATTTCTTCAAAGTAACAGCGCCGGAGGCACGACCCGGCCAATTAAGACCAGGAGCGTATCGCCGACC	4 (0.000077%)
TATCCCATGCTAATGTATCCAGAGCGTAGGCTTGCTTTGAGCACTCTAATTTCTTCAAAGTAACAGCGCCGGAGGCACGACCCGGCCAATTAAGACCAGG	3 (0.000057%)
TATCCTTGTTAGAAGACACAAAGCCAAAGACTCATATGGACTTTGGCTACACCATGAAAGCTTTGAGAAGCAAGAAGAAGGTTGGTTAGTGTTTTGGAGT	24 (0.000460%)
TATGAACACAAACGTTCAATATGACAAACCCATGCCAAGTAAAGAGAAAATGAAAACTGGTGATTGTTGCGGAAATCGTCCAGGATTCCTCGACCAGGAC	5 (0.000096%)
TATGATGTTATCCCATGCTAATGTATCCAGAGCGTAGGCTTGCTTTGAGCACTCTAATTTCTTCAAAGTAACAGCGCCGGAGGCACGACCCGGCCAATTA	23 (0.000441%)
TATGATTGAGTATAAGAACTTAAACCGCAACCGCATCTTATAAGCCTAAGTAGTGTTTCCTTGTTAGAAGACACAAAGCCAAAGACTCATATGGACTTTG	16 (0.000307%)
TATGCTTGTCTCAAAGATTAAGCCATGCATGTGTAAGTATGAACGAATTCAGACTGTGAAACTGCGAATGGCTCATTAAATCAGTTATAGTTTGTTTGAT	14 (0.000268%)
TATTCGACTCCAAAACACTAACCAACCTTCTTCTTGCTTCTCAAAGCTTTCATGGTGTAGCCAAAGTCCATATGAGTCTTTGGCTTTGTGTCTTCTAACA	38 (0.000728%)
TATTCGACTCCAAAACACTAACCAACCTTCTTCTTGCTTCTCAAAGCTTTCATGGTGTAGCCAAAGTCCATATGAGTCTTTGGCTTTGTGTCTTTTAACA	6 (0.000115%)
TATTTAAGTTGTTGCAGTTAAAAAGCTCGTAGTTGAACCTTGGGATGGGTCGGCCGGTCCGCCTTTGGTGTGCATTGGTCGGCTTGTCCCTTCGGTCGGC	4 (0.000077%)
TCAAAATCATCAATCGTTCCAACTAATCTACCGAAGTACTCGGCTAAGAAGAAAGAAGACGGACGAATCCGAGCCAAAGCCGTACAAGCGCGAGATACCT	14 (0.000268%)
TCAAAGATTAAGCCATGCATGTGTAAGTATGAACGAATTCAGACTGTGAAACTGCGAATGGCTCATTAAATCAGTTATAGTTTGTTTGATGGTAACTACT	3 (0.000057%)
TCAAATTAGGTACGAAACACAGGCCCCGGAACTCATCATCGAGCGTAACATCGCCCGTGAATTAACTGAGAAGGATAGGTGGTAGGTAGTTCGATGCGCG	7 (0.000134%)
TCAAATTTCTGCCCTATCAACTTTCGATGGTAGGATAGTGGCCTACCATGGTGGTAACGGGTGACGGAGAATTAGGGTTCGATTCCGGAGAGGGAGCCTG	12 (0.000230%)
TCAAGGGCCGCCGGGGGCGCACCGGACACCACGCGACGTGCGGTGCTCTTCCAGCCGCTGGACCCTACCTCCGGCTGAGCCGTTTCCAGGGTGGGCAGGC	15 (0.000287%)
TCAAGTCATATTCGACTCCAAAACACTAACCAACCTTCTTCTTGCTTCTCAAAGCTTTCATGGTGTAGCCAAAGTCCATATGAGTCTTTGGCTTTGTGTC	44 (0.000843%)
TCAATCATACACATGACATCAAGTCATATTCGACTCCAAAACACTAACCAACCTTCTTCTTGCTTCTCAAAGCTTTCATGGTGTAGCCAAAGTCCATATG	30 (0.000575%)
TCAATCGGTAGGAGCGACGGGCGGTGTGTACAAAGGGCAGGGACGTAGTCAACGCGAGCTGATGACTCGCGCTTACTAGGAATTCCTCGTTGAAGACCAA	63 (0.001207%)
TCACAAATCGTCGTCCCTCACCATCCTTTGCTGATGCGGGACGGAAGCTGGTCTCCCGTGTGTTACCGCACGCGGTTGGCCTAAATCCGAGCCAAGGACG	4 (0.000077%)
TCACATTTCGTTCATCACCCTTGGCCGGCTATCGAACAGCCGGACTCCCATCAAAAGATGGTTGCCAAGAACATCTTCGTTACGGTTTGCTAATTCTCGG	4 (0.000077%)
TCACATTTCGTTCATCACCCTTGGCCGGCTTTCGAACAGCCGGACTCCCATCAAAAGATGGTTGCCAAGAACATCTTCGTTACGGTTTGCTAATTCTCGG	8 (0.000153%)
TCACCATCCTTTGCTGATGCGGGACGGAAGCTGGTCTCCCGTGTGTTACCGCACGCGGTTGGCCTAAATCCGAGCCAAGGACGCCTGGAGCGTACCGACA	7 (0.000134%)
TCACGGTTCGTATTCGTACTGAAAATCAGAATCAAACGAGCTTTTACCCTTTTGTTCCACACGAGATTTCTGTTCTCGTTGAGCTCATCTTAGGACACCT	4 (0.000077%)
TCAGCCTGCTAACTAGCTACGTGGAGGCATCCCTTCACGGCCGGCTTCTTAGAGGGACTATGGCCGTTTAGGCCAAGGAAGTTTGAGGCAATAACAGGTC	7 (0.000134%)
TCAGCGTGCTCCGGGCGTCGGCCTGTGGGCTCCCCATTCGACCCGTCTTGAAACACGGACCAAGGAGTCTGACATGTGTGCGAGTCAACGGGTGAGTAAA	5 (0.000096%)
TCAGCTTCCTTGCGCCTTACGGGTTTACTCACCCGTTGACTCGCACACATGTCAGACTCCTTGGTCCGTGTTTCAAGACGGGTCGAATGGGGAGCCCACA	35 (0.000671%)
TCATACACATGACATCAAGTCATATTCGACTCCAAAACACTAACCAACCTTCTTCTTGCTTCTCAAAGCTTTCATGGTGTAGCCAAAGTCCATATGAGTC	10 (0.000192%)
TCATATTCGACTCCAAAACACTAACCAACCTTCTTCTTGCTTCTCAAAGCTTTCATGGTGTAGCCAAAGTCCATATGAGTCTTTGGCTTTGTGTCTTCTA	60 (0.001150%)
TCATCAGAGCAACGGGCAGAGCCCGCGTCGACCTTTTATCTAATAAATGCGTCCCTTCCATAAGTCGGGGTTTGTTGCACGTATTAGCTCTAGAATTACT	3 (0.000057%)
TCATCATCGAGCGTAACATCGCCCGTGAATTAACTGAGAAGGATAGGTGGTAGGTAGTTCGATGCGCGAGCATGGAGCCTACGAACACTAGCTATCCGAT	246 (0.004714%)
TCATGAATCATCAGAGCAACGGGCAGAGCCCGCGTCGACCTTTTATCTAATAAATGCGTCCCTTCCATAAGTCGGGGTTTGTTGCACGTATTAGCTCTAG	26 (0.000498%)
TCATGTGTATGATTGAGTATAAGAACTTAAACCGCAACCGCATCTTATAAGCCTAAGTAGTGTTTCCTTGTTAGAAGACACAAAGCCAAAGACTCATATG	11 (0.000211%)
TCCAACCTAGGCGAGACAAGGGTTCACATTTCGTTCATCACCCTTGGCCGGCTTTCGAACAGCCGGACTCCCATCAAAAGATGGTTGCCAAGAACATCTT	4 (0.000077%)
TCCAACGAAGCACGCCCATCCAACCTAGGCGAGACAAGGGTTCACATTTCGTTCATCACCCTTGGCCGGCTATCGAACAGCCGGACTCCCATCAAAAGAT	18 (0.000345%)
TCCAGAGCGTAGGCTTGCTTTGAGCACTCTAATTTCTTCAAAGTAACAGCGCCGGAGGCACGACCCGGCCAATTAAGACCAGGAGCGTATCGCCGACCGA	5 (0.000096%)
TCCAGCTCCAATAGCGTATATTTAAGTTGTTGCAGTTAAAAAGCTCGTAGTTGAACCTTGGGATGGGTCGGCCGGTCCGCCTTTGGTGTGCATTGGTCGG	53 (0.001016%)
TCCATGCTCGCGCATCGAACTACCTACCACCTATCCTTCTCAGTTAATTCACGGGCGATGTTACGCTCGATGATGAGTTCCGGGGCCTGTGTTTCGTACC	6 (0.000115%)
TCCCATGCTAATGTATCCAGAGCGTAGGCTTGCTTTGAGCACTCTAATTTCTTCAAAGTAACAGCGCCGGAGGCACGACCCGGCCAATTAAGACCAGGAG	4 (0.000077%)
TCCCGAAGGTATCTCGCGCTTGTACGGCTTTGGCTCGGATTCGTCCGTCTTCTTTCTTCTTAGCCGAGTACTTCGGTAGATTAGTTGGAACGATTGATGA	5 (0.000096%)

TCCCGCCAATCAGCTTCCTTGCGCCTTACGGGTTTACTCACCCGTTGACTCGCACACATGTCAGACTCCTTGGTCCGTGTTTCAAGACGGGTCGAATGGG	3 (0.000057%)
TCCCTTGCCTACATTGTTCCATCGACCAGAGGCTGTTCACCTTGGAGACCTGATGCGGTTATGAGTACGACCGGGCGTGAGCGGCACTCGGTCCTCCGGA	5 (0.000096%)
TCCGCCTAGGCTGTCCCGAGTGTGAGCGAGGTGTGAGTGTCGCCCATGGGCATCGACACCTTGCGGCTAGGAACTGGAACGAGACGGGTGGCAAAGATTT	2024 (0.038789%)
TCCGGAACCGGGACGTGGCGGTTGACGGCAACGTTAGGGAGTCCGGAGACGTCGGCGGGGGCCTCGGGAAGAGTTATCTTTTCTGTTTAACAGCCTGCCC	31 (0.000594%)
TCCGGATTTTCAAGGGCCGCCGGGGGCGCACCGGACACCACGCGACGTGCGGTGCTCTTCCAGCCGCTGGACCCTACCTCCGGCTGAGCCGTTTCCAGGG	3 (0.000057%)
TCCGGGCGTCGGCCTGTGGGCTCCCCATTCGACCCGTCTTGAAACACGGACCAAGGAGTCTGACATGTGTGCGAGTCAACGGGTGAGTAAACCCGTAAGG	5 (0.000096%)
TCCGTCGAGTTATCATGAATCATCAGAGCAACGGGCAGAGCCCGCGTCGACCTTTTATCTAATAAATGCGTCCCTTCCATAAGTCGGGGTTTGTTGCACG	13 (0.000249%)
TCCTATGATGTTATCCCATGCTAATGTATCCAGAGCGTAGGCTTGCTTTGAGCACTCTAATTTCTTCAAAGTAACAGCGCCGGAGGCACGACCCGGCCAA	4 (0.000077%)
TCCTTGCGCCTTACGGGTTTACTCACCCGTTGACTCGCACACATGTCAGACTCCTTGGTCCGTGTTTCAAGACGGGTCGAATGGGGAGCCCACAGGCCGA	7 (0.000134%)
TCCTTGTTAGAAGACACAAAGCCAAAGACTCATATGGACTTTGGCTACACCATGAAAGCTTTGAGAAGCAAGAAGAAGGTTGGTTAGTGTTTTGGAGTCG	250 (0.004791%)
TCGAAATCCTATGATGTTATCCCATGCTAATGTATCCAGAGCGTAGGCTTGCTTTGAGCACTCTAATTTCTTCAAAGTAACAGCGCCGGAGGCACGACCC	203 (0.003890%)
TCGACCCGCCGAAGCGAGCCTTGGGACCAAAAACAGGGGTTGTACCCCGCCTCCGATTCACGGAGTAAGTAAAATAACGTTAAAAGTAGTGGTATTTCAC	3 (0.000057%)
TCGACTCCAAAACACTAACCAACCTTCTTCTTGCTTCTCAAAGCTTTCATGGTGTAGCCAAAGTCCATATGAGTCTTTGGCTTTGTGTCTTCTAACAAGG	25 (0.000479%)
TCGACTCCAAAACACTAACCAACCTTCTTCTTGCTTCTCAAAGCTTTCATGGTGTAGCCAAAGTCCATATGAGTCTTTGGCTTTGTGTCTTTTAACAAGG	11 (0.000211%)
TCGAGTTATCATGAATCATCAGAGCAACGGGCAGAGCCCGCGTCGACCTTTTATCTAATAAATGCGTCCCTTCCATAAGTCGGGGTTTGTTGCACGTATT	58 (0.001112%)
TCGATCCATTACATTTTATCGGTCGCTCTTGTCCGGAAGCTGTAGATGACCCAAAGTCCATATAGCGACCCCAGGTCAGGCGGGATTACCCGCTGAGTTT	7 (0.000134%)
TCGATCCGTCGAGTTATCATGAATCATCAGAGCAACGGGCAGAGCCCGCGTCGACCTTTTATCTAATAAATGCGTCCCTTCCATAAGTCGGGGTTTGTTG	4 (0.000077%)
TCGATCGACCCGCCGAAGCGAGCCTTGGGACCAAAAACAGGGGTTGTACCCCGCCTCCGATTCACGGAGTAAGTAAAATAACGTTAAAAGTAGTGGTATT	3 (0.000057%)
TCGATCTTAAAGGCGTAAGAATTGTATCCTTGTTAAAAGACACAAAGCCAAAGACTCATATGGACTTTGGCTACACCATGAAAGCTTTGAGAAGCAAGAA	4 (0.000077%)
TCGATTAAGACAGCAGGACGGTGGTCATGGAAGTCGAAATCCGCTAAGGAGTGTGTAACAACTCACCTGCCGAATCAACTAGCCCCGAAAATGGATGGCG	3 (0.000057%)
TCGATTAGTCTTTCGCCCCTATACCCAAGTCAGACGAACGATTTGCACGTCAGTATCGCTGCGGGCTTCCACCAGAGTTTCCTCTGGCTTTACCCCGCTC	8 (0.000153%)
TCGCACACATGTCAGACTCCTTGGTCCGTGTTTCAAGACGGGTCGAATGGGGAGCCCACAGGCCGACGCCCGGAGCACGCTGATGCCGAGGCACGCCGTT	3 (0.000057%)
TCGCCCATGGGCATCGACACCTTGCGGCTAGGAACTGGAACGAGACGGGTGGCAAAGATTTCGAGTAGCACTTCATACTACCGTGGGTTTTTTAAACCTT	3 (0.000057%)
TCGCCGCCCGCGACGTCGCGAGAAGTCCACTAAACCTTATCATTTAGAGGAAGGAGAAGTCGTAACAAGGTTTCCGTAGGTGAACCTGCGGAAGGATCGA	8 (0.000153%)
TCGGCATCAGCGTGCTCCGGGCGTCGGCCTGTGGGCTCCCCATTCGACCCGTCTTGAAACACGGACCAAGGAGTCTGACATGTGTGCGAGTCAACGGGTG	17 (0.000326%)
TCGGCCTGTGGGCTCCCCATTCGACCCGTCTTGAAACACGGACCAAGGAGTCTGACATGTGTGCGAGTCAACGGGTGAGTAAACCCGTAAGGCGCAAGGA	65 (0.001246%)
TCGGTAGGAGCGACGGGCGGTGTGTACAAAGGGCAGGGACGTAGTCAACGCGAGCTGATGACTCGCGCTTACTAGGAATTCCTCGTTGAAGACCAACAAT	30 (0.000575%)
TCGGTCCTCCGGATTTTCAAGGGCCGCCGGGGGCGCACCGGACACCACGCGACGTGCGGTGCTCTTCCAGCCGCTGGACCCTACCTCCGGCTGAGCCGTT	11 (0.000211%)
TCGTACTGAAAATCAGAATCAAACGAGCTTTTACCCTTTTGTTCCACACGAGATTTCTGTTCTCGTTGAGCTCATCTTAGGACACCTGCGTTATCTTTTA	4 (0.000077%)
TCGTAGGCTCCATGCTCGCGCATCGAACTACCTACCACCTATCCTTCTCAGTTAATTCACGGGCGATGTTACGCTCGATGATGAGTTCCGGGGCCTGTGT	7 (0.000134%)
TCGTATTCGTACTGAAAATCAGAATCAAACGAGCTTTTACCCTTTTGTTCCACACGAGATTTCTGTTCTCGTTGAGCTCATCTTAGGACACCTGCGTTAT	4 (0.000077%)
TCGTATTTCATAGTCAGAGGTGAAATTCTTGGATTTATGAAAGACGAACAACTGCGAAAGCATTTGCCAAGGATGTTTTCATTAATCAAGAACGAAAGTT	9 (0.000172%)
TCGTCCCTCACCATCCTTTGCTGATGCGGGACGGAAGCTGGTCTCCCGTGTGTTACCGCACGCGGTTGGCCTAAATCCGAGCCAAGGACGCCTGGAGCGT	5 (0.000096%)
TCGTCGTCCCTCACCATCCTTTGCTGATGCGGGACGGAAGCTGGTCTCCCGTGTGTTACCGCACGCGGTTGGCCTAAATCCGAGCCAAGGACGCCTGGAG	5 (0.000096%)
TCTCATGTGTATGATTGAGTATAAGAACTTAAACCGCAACCGCATCTTATAAGCCTAAGTAGTGTTTCCTTGTTAGAAGACACAAAGCCAAAGACTCATA	7 (0.000134%)
TCTCGCGCTTGTACGGCTTTGGCTCGGATTCGTCCGTCTTCTTTCTTCTTAGCCGAGTACTTCGGTAGATTAGTTGGAACGATTGATGATTTTGAGTTAA	15 (0.000287%)
TCTGAGAAGGGTTCGAGTGTGAGCATGCCTGTCGGGACCCGAAAGATGGTGAACTATGCCTGAGCGGGGTAAAGCCAGAGGAAACTCTGGTGGAAGCCCG	8 (0.000153%)
TCTGCCCTATCAACTTTCGATGGTAGGATAGTGGCCTACCATGGTGGTAACGGGTGACGGAGAATTAGGGTTCGATTCCGGAGAGGGAGCCTGAGAAACG	10 (0.000192%)
TCTGGTGCCAGCAGCCGCGGTAATTCCAGCTCCAATAGCGTATATTTAAGTTGTTGCAGTTAAAAAGCTCGTAGTTGAACCTTGGGATGGGTCGGCCGGT	11 (0.000211%)
TCTGTGCTGGCGACGCATCATTCAAATTTCTGCCCTATCAACTTTCGATGGTAGGATAGTGGCCTACCATGGTGGTAACGGGTGACGGAGAATTAGGGTT	24 (0.000460%)
TCTGTTAAAAGATAACGCAGGTGTCCTAAGATGAGCTCAACGAGAACAGAAATCTCGTGTGGAACAAAAGGGTAAAAGCTCGTTTGATTCTGATTTTCAG	4 (0.000077%)
TCTTAAAGGCGTAAGAATTGTATCCTTGTTAGAAGACACA	426 (0.003266%)
TCTTATACTCAATCATACACATGACATCAAGTCATATTCGACTCCAAAACACTAACCAACCTTCTTCTTGCTTCTCAAAGCTTTCATGGTGTAGCCAAAG	199 (0.003814%)

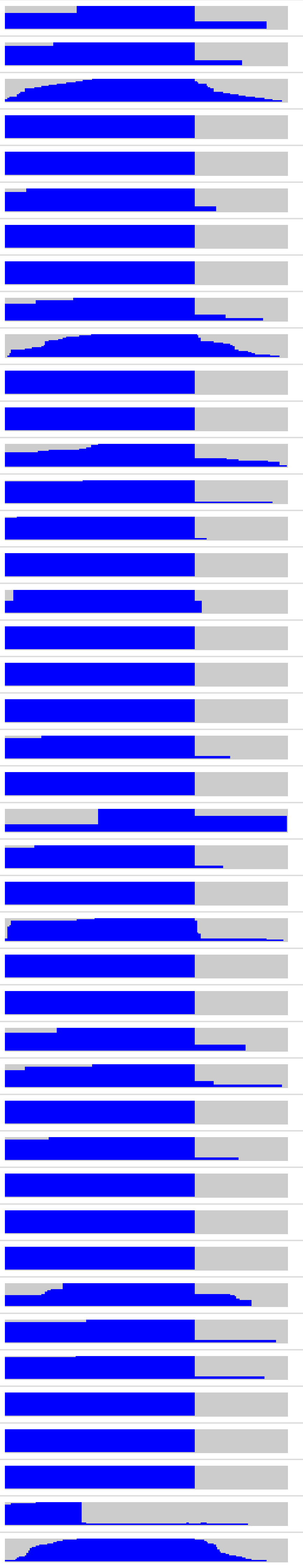

TCTTCAACGAGGAATTCCTAGTAAGCGCGAGTCATCAGCTCGCGTTGACTACGTCCCTGCCCTTTGTACACACCGCCCGTCGCTCCTACCGATTGAATGA	12 (0.000230%)
TGAACACAAACGTTCAATATGACAAACCCATGCCAAGTAAAGAGAAAATGAAAACTGGTGATTGTTGCGGAAATCGTCCAGGATTCCTCGACCAGGACTT	19 (0.000364%)
TGAATCATCAGAGCAACGGGCAGAGCCCGCGTCGACCTTTTATCTAATAAATGCGTCCCTTCCATAAGTCGGGGTTTGTTGCACGTATTAGCTCTAGAAT	7 (0.000134%)
TGACATCAAGTCATATTCGACTCCAAAACACTAACCAACCTTCTTCTTGCTTCTCAAAGCTTTCATGGTGTAGCCAAAGTCCATATGAGTCTTTGGCTTT	32 (0.000613%)
TGACGGCAACGTTAGGGAGTCCGGAGACGTCGGCGGGGGCCTCGGGAAGAGTTATCTTTTCTGTTTAACAGCCTGCCCACCCTGGAAACGGCTCAGCCGG	22 (0.000422%)
TGACTCGCACACATGTCAGACTCCTTGGTCCGTGTTTCAAGACGGGTCGAATGGGGAGCCCACAGGCCGACGCCCGGAGCACGCTGATGCCGAGGCACGC	5 (0.000096%)
TGAGCGAGGTGTGAGTGTCGCCCATGGGCATCGACACCTTGCGGCTAGGAACTGGAACGAGACGGGTGGCAAAGATTTCGAGTAGCACTTCATACTACCG	6 (0.000115%)
TGAGTATAAGAACTTAAACCGCAACCGCATCTTATAAGCCTAAGTAGTGTTTCCTTGTTAGAAGACACAAAGCCAAAGACTCATATGGACTTTGGCTACA	23 (0.000441%)
TGAGTGTCGCCCATGGGCATCGACACCTTGCGGCTAGGAACTGGAACGAGACGGGTGGCAAAGATTTCGAGTAGCACTTCATACTACCGTGGGTTTTTTA	7 (0.000134%)
TGATGTTATCCCATGCTAATGTATCCAGAGCGTAGGCTTGCTTTGAGCACTCTAATTTCTTCAAAGTAACAGCGCCGGAGGCACGACCCGGCCAATTAAG	21 (0.000402%)
TGATTAACAGGGACAGTCGGGGGCATTCGTATTTCATAGTCAGAGGTGAAATTCTTGGATTTATGAAAGACGAACAACTGCGAAAGCATTTGCCAAGGAT	126 (0.002415%)
TGATTGAGTATAAGAACTTAAACCGCAACCGCATCTTATAAGCCTAAGTAGTGTTTCCTTGTTAGAAGACACAAAGCCAAAGACTCATATGGACTTTGGC	5 (0.000096%)
TGCAGCACGCGCCTAACGGCGTGCCTCGGCATCAGCGTGCTCCGGGCGTCGGCCTGTGGGCTCCCCATTCGACCCGTCTTGAAACACGGACCAAGGAGTC	58 (0.001112%)
TGCCAGCAGCCGCGGTAATTCCAGCTCCAATAGCGTATATTTAAGTTGTTGCAGTTAAAAAGCTCGTAGTTGAACCTTGGGATGGGTCGGCCGGTCCGCC	5 (0.000096%)
TGCCGACTTCCCTTGCCTACATTGTTCCATCGACCAGAGGCTGTTCACCTTGGAGACCTGATGCGGTTATGAGTACGACCGGGCGTGAGCGGCACTCGGT	2358 (0.045190%)
TGCCTACATTGTTCCATCGACCAGAGGCTGTTCACCTTGGAGACCTGATGCGGTTATGAGTACGACCGGGCGTGAGCGGCACTCGGTCCTCCGGATTTTC	8 (0.000153%)
TGCGCCTTACGGGTTTACTCACCCGTTGACTCGCACACATGTCAGACTCCTTGGTCCGTGTTTCAAGACGGGTCGAATGGGGAGCCCACAGGCCGACGCC	6 (0.000115%)
TGCGGTTTAAGTTCTTATACTCAATCATACACATGACATCAAGTCATATTCGACTCCAAAACACTAACCAACCTTCTTCTTGCTTCTCAAAGCTTTCATG	392 (0.007513%)
TGCGGTTTAAGTTGTTATACTCAATCATACACATGACAACAAGTCATATTCGACTCCAAAACACTAACCAACCTTCTTCTTGCTTCTCAAAGCTTTCATG	92 (0.001763%)
TGCTAACTAGCTACGTGGAGGCATCCCTTCACGGCCGGCTTCTTAGAGGGACTATGGCCGTTTAGGCCAAGGAAGTTTGAGGCAATAACAGGTCTGTGAT	26 (0.000498%)
TGCTAATGTATCCAGAGCGTAGGCTTGCTTTGAGCACTCTAATTTCTTCAAAGTAACAGCGCCGGAGGCACGACCCGGCCAATTAAGACCAGGAGCGTAT	13 (0.000249%)
TGCTCCGGGCGTCGGCCTGTGGGCTCCCCATTCGACCCGTCTTGAAACACGGACCAAGGAGTCTGACATGTGTGCGAGTCAACGGGTGAGTAAACCCGTA	5 (0.000096%)
TGCTGATGCGGGACGGAAGCTGGTCTCCCGTGTGTTACCGCACGCGGTTGGCCTAAATCCGAGCCAAGGACGCCTGGAGCGTACCGACATGCGGTGGTGA	6 (0.000115%)
TGCTTGTCTCAAAGATTAAGCCATGCATGTGTAAGTATGAACGAATTCAGACTGTGAAACTGCGAATGGCTCATTAAATCAGTTATAGTTTGTTTGATGG	5 (0.000096%)
TGCTTTGTTTTAATTAAACAGTCGGATTCCCCTTGTCCGTACCAGTTCTGAGCTGACTGTTCGACGCCCGGGGAAAGCTCCCGAGAGAGCCGTTCCCAGT	4 (0.000077%)
TGGAAGTCGAAATCCGCTAAGGAGTGTGTAACAACTCACCTGCCGAATCAACTAGCCCCGAAAATGGATGGCGCTTAAGCGCGCGACCTATACCCGGCCG	62 (0.001188%)
TGGAGGGCAAGTCTGGTGCCAGCAGCCGCGGTAATTCCAGCTCCAATAGCGTATATTTAAGTTGTTGCAGTTAAAAAGCTCGTAGTTGAACCTTGGGATG	311 (0.005960%)
TGGCCTCTGTGCTGGCGACGCATCATTCAAATTTCTGCCCTATCAACTTTCGATGGTAGGATAGTGGCCTACCATGGTGGTAACGGGTGACGGAGAATTA	32 (0.000613%)
TGGCGACGCATCATTCAAATTTCTGCCCTATCAACTTTCGATGGTAGGATAGTGGCCTACCATGGTGGTAACGGGTGACGGAGAATTAGGGTTCGATTCC	33 (0.000632%)
TGGCGGTTGACGGCAACGTTAGGGAGTCCGGAGACGTCGGCGGGGGCCTCGGGAAGAGTTATCTTTTCTGTTTAACAGCCTGCCCACCCTGGAAACGGCT	7 (0.000134%)
TGGCTGGGGCGGCACATCTGTTAAAAGATAACGCAGGTGTCCTAAGATGAGCTCAACGAGAACAGAAATCTCGTGTGGAACAAAAGGGTAAAAGCTCGTT	4 (0.000077%)
TGGGCATCGACACCTTGCGGCTAGGAACTGGAACGAGACGGGTGGCAAAGATTTCGAGTAGCACTTCATACTACCGTGGGTTTTTTAAACCTTCCGAGTT	18 (0.000345%)
TGGGGAGTTTGGCTGGGGCGGCACATCTGTTAAAAGATAACGCAGGTGTCCTAAGATGAGCTCAACGAGAACAGAAATCTCGTGTGGAACAAAAGGGTAA	8 (0.000153%)
TGGGGCGGCACATCTGTTAAAAGATAACGCAGGTGTCCTAAGATGAGCTCAACGAGAACAGAAATCTCGTGTGGAACAAAAGGGTAAAAGCTCGTTTGAT	17 (0.000326%)
TGGGTGGTTCGCCGCCCGCGACGTCGCGAGAAGTCCACTAAACCTTATCATTTAGAGGAAGGAGAAGTCGTAACAAGGTTTCCGTAGGTGAACCTGCGGA	8 (0.000153%)
TGGGTGTCACAAATCGTCGTCCCTCACCATCCTTTGCTGATGCGGGACGGAAGCTGGTCTCCCGTGTGTTACCGCACGCGGTTGGCCTAAATCCGAGCCA	3 (0.000057%)
TGGTCATGGAAGTCGAAATCCGCTAAGGAGTGTGTAACAACTCACCTGCCGAATCAACTAGCCCCGAAAATGGATGGCGCTTAAGCGCGCGACCTATACC	17 (0.000326%)
TGGTCGATTAAGACAGCAGGACGGTGGTCATGGAAGTCGAAATCCGCTAAGGAGTGTGTAACAACTCACCTGCCGAATCAACTAGCCCCGAAAATGGATG	7 (0.000134%)
TGGTCTTCAACGAGGAATTCCTAGTAAGCGCGAGTCATCAGCTCGCGTTGACTACGTCCCTGCCCTTTGTACACACCGCCCGTCGCTCCTACCGATTGAA	55 (0.001054%)
TGGTGCCAGCAGCCGCGGTAATTCCAGCTCCAATAGCGTATATTTAAGTTGTTGCAGTTAAAAAGCTCGTAGTTGAACCTTGGGATGGGTCGGCCGGTCC	8 (0.000153%)
TGGTTCGCCGCCCGCGACGTCGCGAGAAGTCCACTAAACCTTATCATTTAGAGGAAGGAGAAGTCGTAACAAGGTTTCCGTAGGTGAACCTGCGGAAGGA	3 (0.000057%)
TGTAAAGTTGGGAATTCGTTAAGGAGCTGTTGCTTTGTTAGTGTAGAAACACTTGTGTAGAATTGGGGATTGTTTTTTTTGGAGTGATTTAGGGGAGGGT	10 (0.000192%)
TGTATCCAGAGCGTAGGCTTGCTTTGAGCACTCTAATTTCTTCAAAGTAACAGCGCCGGAGGCACGACCCGGCCAATTAAGACCAGGAGCGTATCGCCGA	22 (0.000422%)

TGTATCCTTGTTAGAAGACACAAAGCCAAAGACTCATATGGACTTTGGCTACACCATGAAAGCTTTGAGAAGCAAGAAGAAGGTTGGTTAGTGTTTTGGA	702 (0.013454%)
TGTATGATTGAGTATAAGAACTTAAACCGCAACCGCATCTTATAAGCCTAAGTAGTGTTTCCTTGTTAGAAGACACAAAGCCAAAGACTCATATGGACTT	3 (0.000057%)
TGTCAGGTGGGGAGTTTGGCTGGGGCGGCACATCTGTTAAAAGATAACGCAGGTGTCCTAAGATGAGCTCAACGAGAACAGAAATCTCGTGTGGAACAAA	49 (0.000939%)
TGTCCCGAAGGTATCTCGCGCTTGTACGGCTTTGGCTCGGATTCGTCCGTCTTCTTTCTTCTTAGCCGAGTACTTCGGTAGATTAGTTGGAACGATTGAT	3 (0.000057%)
TGTCCCGAGTGTGAGCGAGGTGTGAGTGTCGCCCATGGGCATCGACACCTTGCGGCTAGGAACTGGAACGAGACGGGTAGCAAAGATTTCGAGTAGCACT	3 (0.000057%)
TGTCCCGAGTGTGAGCGAGGTGTGAGTGTCGCCCATGGGCATCGACACCTTGCGGCTAGGAACTGGAACGAGACGGGTGGCAAAGATTTCGAGTAGCACT	5 (0.000096%)
TGTCGCCCATGGGCATCGACACCTTGCGGCTAGGAACTGGAACGAGACGGGTGGCAAAGATTTCGAGTAGCACTTCATACTACCGTGGGTTTTTTAAACC	4 (0.000077%)
TGTCTCAAAGATTAAGCCATGCATGTGTAAGTATGAACGAATTCAGACTGTGAAACTGCGAATGGCTCATTAAATCAGTTATAGTTTGTTTGATGGTAAC	3 (0.000057%)
TGTGAGCGAGGTGTGAGTGTCGCCCATGGGCATCGACACCTTGCGGCTAGGAACTGGAACGAGACGGGTGGCAAAGATTTCGAGTAGCACTTCATACTAC	3 (0.000057%)
TGTGAGTGTCGCCCATGGGCATCGACACCTTGCGGCTAGGAACTGGAACGAGACGGGTAGCAAAGATTTCGAGTAGCACTTCATACTACCGTGGGTTTTT	7 (0.000134%)
TGTGAGTGTCGCCCATGGGCATCGACACCTTGCGGCTAGGAACTGGAACGAGACGGGTGGCAAAGATTTCGAGTAGCACTTCATACTACCGTGGGTTTTT	7 (0.000134%)
TGTGCTGGCGACGCATCATTCAAATTTCTGCCCTATCAACTTTCGATGGTAGGATAGTGGCCTACCATGGTGGTAACGGGTGACGGAGAATTAGGGTTCG	8 (0.000153%)
TGTTAGAAGACACAAAGCCAAAGACTCATATGGACTTTGGCTACACCATGAAAGCTTTGAGAAGCAAGAAGAAGGTTGGTTAGTGTTTTGGAGTCGAATA	24 (0.000460%)
TGTTATACTCAATCATACACATGACAACAAGTCATATTCGACTCCAAAACACTAACCAACCTTCTTCTTGCTTCTCAAAGCTTTCATGGTGTAGCCAAAG	3 (0.000057%)
TGTTATCCCATGCTAATGTATCCAGAGCGTAGGCTTGCTTTGAGCACTCTAATTTCTTCAAAGTAACAGCGCCGGAGGCACGACCCGGCCAATTAAGACC	12 (0.000230%)
TGTTGCTTTGTTAGTGTAGAAACACTTGTGTAGAATTGGGGATTGTTTTTTTTGGAGTGATTTAGGGGAGGGTCGAATCTTAGCGACAAAGGGCTGAATC	15 (0.000287%)
TGTTGGTCGATTAAGACAGCAGGACGGTGGTCATGGAAGTCGAAATCCGCTAAGGAGTGTGTAACAACTCACCTGCCGAATCAACTAGCCCCGAAAATGG	8 (0.000153%)
TGTTGGTCTTCAACGAGGAATTCCTAGTAAGCGCGAGTCATCAGCTCGCGTTGACTACGTCCCTGCCCTTTGTACACACCGCCCGTCGCTCCTACCGATT	196 (0.003756%)
TGTTTTAATTAAACAGTCGGATTCCCCTTGTCCGTACCAGTTCTGAGCTGACTGTTCGACGCCCGGGGAAAGCTCCCGAGAGAGCCGTTCCCAGTCCGTC	3 (0.000057%)
TGTTTTGGAGTCGAATATGACTTGATGTCATGTGTATGAT	252 (0.001932%)
TTAAAAGATAACGCAGGTGTCCTAAGATGAGCTCAACGAGAACAGAAATCTCGTGTGGAACAAAAGGGTAAAAGCTCGTTTGATTCTGATTTTCAGTACG	24 (0.000460%)
TTAAAGGCGTAAGAATTGTATCCTTGTTAGAAGACACAAA	519 (0.003979%)
TTAACAGGGACAGTCGGGGGCATTCGTATTTCATAGTCAGAGGTGAAATTCTTGGATTTATGAAAGACGAACAACTGCGAAAGCATTTGCCAAGGATGTT	191 (0.003660%)
TTAAGACAGCAGGACGGTGGTCATGGAAGTCGAAATCCGCTAAGGAGTGTGTAACAACTCACCTGCCGAATCAACTAGCCCCGAAAATGGATGGCGCTTA	86 (0.001648%)
TTAAGGAGCTGTTGCTTTGTTAGTGTAGAAACACTTGTGTAGAATTGGGGATTGTTTTTTTTGGAGTGATTTAGGGGAGGGTCGAATCTTAGCGACAAAG	15 (0.000287%)
TTAAGTTCTTATACTCAATCATACACATGACATCAAGTCATATTCGACTCCAAAACACTAACCAACCTTCTTCTTGCTTCTCAAAGCTTTCATGGTGTAG	166 (0.003181%)
TTAAGTTGTTATACTCAATCATACACATGACAACAAGTCATATTCGACTCCAAAACACTAACCAACCTTCTTCTTGCTTCTCAAAGCTTTCATGGTGTAG	63 (0.001207%)
TTACATTTTATCGGTCGCTCTTGTCCGGAAGCTGTAGATGACCCAAAGTCCATATAGCGACCCCAGGTCAGGCGGGATTACCCGCTGAGTTTAAGCATAT	12 (0.000230%)
TTACGGGTTTACTCACCCGTTGACTCGCACACATGTCAGACTCCTTGGTCCGTGTTTCAAGACGGGTCGAATGGGGAGCCCACAGGCCGACGCCCGGAGC	24 (0.000460%)
TTACTCACCCGTTGACTCGCACACATGTCAGACTCCTTGGTCCGTGTTTCAAGACGGGTCGAATGGGGAGCCCACAGGCCGACGCCCGGAGCACGCTGAT	9 (0.000172%)
TTAGAAGACACAAAGCCAAAGACTCATATGGACTTTGGCTACACCATGAAAGCTTTGAGAAGCAAGAAGAAGGTTGGTTAGTGTTTTGGAGTCGAATATG	73 (0.001399%)
TTAGGGAGTCCGGAGACGTCGGCGGGGGCCTCGGGAAGAGTTATCTTTTCTGTTTAACAGCCTGCCCACCCTGGAAACGGCTCAGCCGGAGGTAGGGTCC	3 (0.000057%)
TTAGGTACGAAACACAGGCCCCGGAACTCATCATCGAGCGTAACATCGCCCGTGAATTAACTGAGAAGGATAGGTGGTAGGTAGTTCGATGCGCGAGCAT	14 (0.000268%)
TTAGTGTTTTGGAGTCGAATATGACTTGATGTCATGTGTA	340 (0.002606%)
TTATACTCAATCATACACATGACAACAAGTCATATTCGACTCCAAAACACTAACCAACCTTCTTCTTGCTTCTCAAAGCTTTCATGGTGTAGCCAAAGTC	276 (0.005289%)
TTATACTCAATCATACACATGACATCAAGTCATATTCGACTCCAAAACACTAACCAACCTTCTTCTTGCTTCTCAAAGCTTTCATGGTGTAGCCAAAGTC	697 (0.013358%)
TTATCATGAATCATCAGAGCAACGGGCAGAGCCCGCGTCGACCTTTTATCTAATAAATGCGTCCCTTCCATAAGTCGGGGTTTGTTGCACGTATTAGCTC	55 (0.001054%)
TTATCCCATGCTAATGTATCCAGAGCGTAGGCTTGCTTTGAGCACTCTAATTTCTTCAAAGTAACAGCGCCGGAGGCACGACCCGGCCAATTAAGACCAG	6 (0.000115%)
TTATCGGTCGCTCTTGTCCGGAAGCTGTAGATGACCCAAAGTCCATATAGCGACCCCAGGTCAGGCGGGATTACCCGCTGAGTTTAAGCATATCAATAAG	6 (0.000115%)
TTCAAATTAGGTACGAAACACAGGCCCCGGAACTCATCATCGAGCGTAACATCGCCCGTGAATTAACTGAGAAGGATAGGTGGTAGGTAGTTCGATGCGC	36 (0.000690%)
TTCAAATTTCTGCCCTATCAACTTTCGATGGTAGGATAGTGGCCTACCATGGTGGTAACGGGTGACGGAGAATTAGGGTTCGATTCCGGAGAGGGAGCCT	20 (0.000383%)
TTCAACGAGGAATTCCTAGTAAGCGCGAGTCATCAGCTCGCGTTGACTACGTCCCTGCCCTTTGTACACACCGCCCGTCGCTCCTACCGATTGAATGATC	7 (0.000134%)
TTCAAGGGCCGCCGGGGGCGCACCGGACACCACGCGACGTGCGGTGCTCTTCCAGCCGCTGGACCCTACCTCCGGCTGAGCCGTTTCCAGGGTGGGCAGG	14 (0.000268%)

TTCAATCGGTAGGAGCGACGGGCGGTGTGTACAAAGGGCAGGGACGTAGTCAACGCGAGCTGATGACTCGCGCTTACTAGGAATTCCTCGTTGAAGACCA	84 (0.001610%)
TTCAATGTCTGTTGGTGCCAAGAGGGAAAAGGGCTATTAAGCTATATAGGGGGGTGGGTGTTGAGGGAGTCTGGGCAGTCCGTGGGGAACCCCCTTTTTC	469 (0.008988%)
TTCACATTTCGTTCATCACCCTTGGCCGGCTATCGAACAGCCGGACTCCCATCAAAAGATGGTTGCCAAGAACATCTTCGTTACGGTTTGCTAATTCTCG	17 (0.000326%)
TTCACATTTCGTTCATCACCCTTGGCCGGCTTTCGAACAGCCGGACTCCCATCAAAAGATGGTTGCCAAGAACATCTTCGTTACGGTTTGCTAATTCTCG	17 (0.000326%)
TTCACGGTTCGTATTCGTACTGAAAATCAGAATCAAACGAGCTTTTACCCTTTTGTTCCACACGAGATTTCTGTTCTCGTTGAGCTCATCTTAGGACACC	14 (0.000268%)
TTCAGCAGTTCTCGGACAAAAATTGCTGAGTGGCCGAGAAGAATGGGCGTGTCATGCGTGGGCTGACATGGATTCTTCGAGGCCTAGGGGTGGCGGTATA	8 (0.000153%)
TTCATCACCCTTGGCCGGCTTTCGAACAGCCGGACTCCCATCAAAAGATGGTTGCCAAGAACATCTTCGTTACGGTTTGCTAATTCTCGGAATAACATCA	3 (0.000057%)
TTCCAACGAAGCACGCCCATCCAACCTAGGCGAGACAAGGGTTCACATTTCGTTCATCACCCTTGGCCGGCTATCGAACAGCCGGACTCCCATCAAAAGA	105 (0.002012%)
TTCCCTTGCCTACATTGTTCCATCGACCAGAGGCTGTTCACCTTGGAGACCTGATGCGGTTATGAGTACGACCGGGCGTGAGCGGCACTCGGTCCTCCGG	5 (0.000096%)
TTCCGGAACCGGGACGTGGCGGTTGACGGCAACGTTAGGGAGTCCGGAGACGTCGGCGGGGGCCTCGGGAAGAGTTATCTTTTCTGTTTAACAGCCTGCC	2365 (0.045324%)
TTCCTTGCGCCTTACGGGTTTACTCACCCGTTGACTCGCACACATGTCAGACTCCTTGGTCCGTGTTTCAAGACGGGTCGAATGGGGAGCCCACAGGCCG	8 (0.000153%)
TTCGACTCCAAAACACTAACCAACCTTCTTCTTGCTTCTCAAAGCTTTCATGGTGTAGCCAAAGTCCATATGAGTCTTTGGCTTTGTGTCTTCTAACAAG	99 (0.001897%)
TTCGACTCCAAAACACTAACCAACCTTCTTCTTGCTTCTCAAAGCTTTCATGGTGTAGCCAAAGTCCATATGAGTCTTTGGCTTTGTGTCTTTTAACAAG	85 (0.001629%)
TTCGAGTGTGAGCATGCCTGTCGGGACCCGAAAGATGGTGAACTATGCCTGAGCGGGGTAAAGCCAGAGGAAACTCTGGTGGAAGCCCGCAGCGATACTG	3 (0.000057%)
TTCGATTAGTCTTTCGCCCCTATACCCAAGTCAGACGAACGATTTGCACGTCAGTATCGCTGCGGGCTTCCACCAGAGTTTCCTCTGGCTTTACCCCGCT	21 (0.000402%)
TTCGCCGCCCGCGACGTCGCGAGAAGTCCACTAAACCTTATCATTTAGAGGAAGGAGAAGTCGTAACAAGGTTTCCGTAGGTGAACCTGCGGAAGGATCG	3 (0.000057%)
TTCGTACTGAAAATCAGAATCAAACGAGCTTTTACCCTTTTGTTCCACACGAGATTTCTGTTCTCGTTGAGCTCATCTTAGGACACCTGCGTTATCTTTT	18 (0.000345%)
TTCGTAGGCTCCATGCTCGCGCATCGAACTACCTACCACCTATCCTTCTCAGTTAATTCACGGGCGATGTTACGCTCGATGATGAGTTCCGGGGCCTGTG	9 (0.000172%)
TTCGTATTCGTACTGAAAATCAGAATCAAACGAGCTTTTACCCTTTTGTTCCACACGAGATTTCTGTTCTCGTTGAGCTCATCTTAGGACACCTGCGTTA	16 (0.000307%)
TTCGTATTTCATAGTCAGAGGTGAAATTCTTGGATTTATGAAAGACGAACAACTGCGAAAGCATTTGCCAAGGATGTTTTCATTAATCAAGAACGAAAGT	22 (0.000422%)
TTCGTTCATCACCCTTGGCCGGCTATCGAACAGCCGGACTCCCATCAAAAGATGGTTGCCAAGAACATCTTCGTTACGGTTTGCTAATTCTCGGAATAAC	12 (0.000230%)
TTCGTTCATCACCCTTGGCCGGCTTTCGAACAGCCGGACTCCCATCAAAAGATGGTTGCCAAGAACATCTTCGTTACGGTTTGCTAATTCTCGGAATAAC	11 (0.000211%)
TTCTGAGAAGGGTTCGAGTGTGAGCATGCCTGTCGGGACCCGAAAGATGGTGAACTATGCCTGAGCGGGGTAAAGCCAGAGGAAACTCTGGTGGAAGCCC	10 (0.000192%)
TTCTGCCCTATCAACTTTCGATGGTAGGATAGTGGCCTACCATGGTGGTAACGGGTGACGGAGAATTAGGGTTCGATTCCGGAGAGGGAGCCTGAGAAAC	24 (0.000460%)
TTCTTATACTCAATCATACACATGACATCAAGTCATATTCGACTCCAAAACACTAACCAACCTTCTTCTTGCTTCTCAAAGCTTTCATGGTGTAGCCAAA	76 (0.001457%)
TTGAAATCGTCGACCAGGTCCGAGACTTCATCGACCGGGTCCGAGGATTCGTCGACCAGGACGGCCGGATGTCCGAGAAAAAAAAATGTTGCCGAATAAC	46 (0.000882%)
TTGACGGCAACGTTAGGGAGTCCGGAGACGTCGGCGGGGGCCTCGGGAAGAGTTATCTTTTCTGTTTAACAGCCTGCCCACCCTGGAAACGGCTCAGCCG	10 (0.000192%)
TTGACTCGCACACATGTCAGACTCCTTGGTCCGTGTTTCAAGACGGGTCGAATGGGGAGCCCACAGGCCGACGCCCGGAGCACGCTGATGCCGAGGCACG	15 (0.000287%)
TTGAGTATAAGAACTTAAACCGCAACCGCATCTTATAAGCCTAAGTAGTGTTTCCTTGTTAGAAGACACAAAGCCAAAGACTCATATGGACTTTGGCTAC	8 (0.000153%)
TTGCGCCTTACGGGTTTACTCACCCGTTGACTCGCACACATGTCAGACTCCTTGGTCCGTGTTTCAAGACGGGTCGAATGGGGAGCCCACAGGCCGACGC	20 (0.000383%)
TTGCGGTTTAAGTTCTTATACTCAATCATACACATGACATCAAGTCATATTCGACTCCAAAACACTAACCAACCTTCTTCTTGCTTCTCAAAGCTTTCAT	444 (0.008509%)
TTGCGGTTTAAGTTGTTATACTCAATCATACACATGACAACAAGTCATATTCGACTCCAAAACACTAACCAACCTTCTTCTTGCTTCTCAAAGCTTTCAT	85 (0.001629%)
TTGCTGATGCGGGACGGAAGCTGGTCTCCCGTGTGTTACCGCACGCGGTTGGCCTAAATCCGAGCCAAGGACGCCTGGAGCGTACCGACATGCGGTGGTG	5 (0.000096%)
TTGGAGACCTGATGCGGTTATGAGTACGACCGGGCGTGAGCGGCACTCGGTCCTCCGGATTTTCAAGGGCCGCCGGGGGCGCACCGGACACCACGCGACG	22 (0.000422%)
TTGGAGGGCAAGTCTGGTGCCAGCAGCCGCGGTAATTCCAGCTCCAATAGCGTATATTTAAGTTGTTGCAGTTAAAAAGCTCGTAGTTGAACCTTGGGAT	314 (0.006018%)
TTGGAGTCGAATATGACTTGATGTCATGTGTATGATTGAG	274 (0.002100%)
TTGGCTCGGATTCGTCCGTCTTCTTTCTTCTTAGCCGAGTACTTCGGTAGATTAGTTGGAACGATTGATGATTTTGAGTTAATTGAACGTTCGGCGTATG	6 (0.000115%)
TTGGCTGGGGCGGCACATCTGTTAAAAGATAACGCAGGTGTCCTAAGATGAGCTCAACGAGAACAGAAATCTCGTGTGGAACAAAAGGGTAAAAGCTCGT	19 (0.000364%)
TTGGTCGATTAAGACAGCAGGACGGTGGTCATGGAAGTCGAAATCCGCTAAGGAGTGTGTAACAACTCACCTGCCGAATCAACTAGCCCCGAAAATGGAT	7 (0.000134%)
TTGGTCTTCAACGAGGAATTCCTAGTAAGCGCGAGTCATCAGCTCGCGTTGACTACGTCCCTGCCCTTTGTACACACCGCCCGTCGCTCCTACCGATTGA	110 (0.002108%)
TTGGTGATATGAACACAAACGTTCAATATGACAAACCCATGCCAAGTAAAGAGAAAATGAAAACTGGTGATTGTTGCGGAAATCGTCCAGGATTCCTCGA	6 (0.000115%)
TTGTACGGCTTTGGCTCGGATTCGTCCGTCTTCTTTCTTCTTAGCCGAGTACTTCGGTAGATTAGTTGGAACGATTGATGATTTTGAGTTAATTGAACGT	6 (0.000115%)
TTGTATCCTTGTTAGAAGACACAAAGCCAAAGACTCATATGGACTTTGGCTACACCATGAAAGCTTTGAGAAGCAAGAAGAAGGTTGGTTAGTGTTTTGG	11 (0.000211%)

TTGTCAGGTGGGGAGTTTGGCTGGGGCGGCACATCTGTTAAAAGATAACGCAGGTGTCCTAAGATGAGCTCAACGAGAACAGAAATCTCGTGTGGAACAA	17 (0.000326%)
TTGTCTCAAAGATTAAGCCATGCATGTGTAAGTATGAACGAATTCAGACTGTGAAACTGCGAATGGCTCATTAAATCAGTTATAGTTTGTTTGATGGTAA	15 (0.000287%)
TTGTTAGAAGACACAAAGCCAAAGACTCATATGGACTTTGGCTACACCATGAAAGCTTTGAGAAGCAAGAAGAAGGTTGGTTAGTGTTTTGGAGTCGAAT	53 (0.001016%)
TTGTTATACTCAATCATACACATGACAACAAGTCATATTCGACTCCAAAACACTAACCAACCTTCTTCTTGCTTCTCAAAGCTTTCATGGTGTAGCCAAA	58 (0.001112%)
TTGTTGGTCTTCAACGAGGAATTCCTAGTAAGCGCGAGTCATCAGCTCGCGTTGACTACGTCCCTGCCCTTTGTACACACCGCCCGTCGCTCCTACCGAT	187 (0.003584%)
TTGTTTTAATTAAACAGTCGGATTCCCCTTGTCCGTACCAGTTCTGAGCTGACTGTTCGACGCCCGGGGAAAGCTCCCGAGAGAGCCGTTCCCAGTCCGT	12 (0.000230%)
TTTAAGTTCTTATACTCAATCATACACATGACATCAAGTCATATTCGACTCCAAAACACTAACCAACCTTCTTCTTGCTTCTCAAAGCTTTCATGGTGTA	105 (0.002012%)
TTTAAGTTGTTATACTCAATCATACACATGACAACAAGTCATATTCGACTCCAAAACACTAACCAACCTTCTTCTTGCTTCTCAAAGCTTTCATGGTGTA	26 (0.000498%)
TTTAATTAAACAGTCGGATTCCCCTTGTCCGTACCAGTTCTGAGCTGACTGTTCGACGCCCGGGGAAAGCTCCCGAGAGAGCCGTTCCCAGTCCGTCCCC	14 (0.000268%)
TTTACTCACCCGTTGACTCGCACACATGTCAGACTCCTTGGTCCGTGTTTCAAGACGGGTCGAATGGGGAGCCCACAGGCCGACGCCCGGAGCACGCTGA	11 (0.000211%)
TTTATCGGTCGCTCTTGTCCGGAAGCTGTAGATGACCCAAAGTCCATATAGCGACCCCAGGTCAGGCGGGATTACCCGCTGAGTTTAAGCATATCAATAA	12 (0.000230%)
TTTCAAGGGCCGCCGGGGGCGCACCGGACACCACGCGACGTGCGGTGCTCTTCCAGCCGCTGGACCCTACCTCCGGCTGAGCCGTTTCCAGGGTGGGCAG	22 (0.000422%)
TTTCACGGTTCGTATTCGTACTGAAAATCAGAATCAAACGAGCTTTTACCCTTTTGTTCCACACGAGATTTCTGTTCTCGTTGAGCTCATCTTAGGACAC	51 (0.000977%)
TTTCAGCAGTTCTCGGACAAAAATTGCTGAGTGGCCGAGAAGAATGGGCGTGTCATGCGTGGGCTGACATGGATTCTTCGAGGCCTAGGGGTGGCGGTAT	5 (0.000096%)
TTTCCAACGAAGCACGCCCATCCAACCTAGGCGAGACAAGGGTTCACATTTCGTTCATCACCCTTGGCCGGCTATCGAACAGCCGGACTCCCATCAAAAG	1817 (0.034822%)
TTTCGTTCATCACCCTTGGCCGGCTATCGAACAGCCGGACTCCCATCAAAAGATGGTTGCCAAGAACATCTTCGTTACGGTTTGCTAATTCTCGGAATAA	12 (0.000230%)
TTTCGTTCATCACCCTTGGCCGGCTTTCGAACAGCCGGACTCCCATCAAAAGATGGTTGCCAAGAACATCTTCGTTACGGTTTGCTAATTCTCGGAATAA	9 (0.000172%)
TTTCTGCCCTATCAACTTTCGATGGTAGGATAGTGGCCTACCATGGTGGTAACGGGTGACGGAGAATTAGGGTTCGATTCCGGAGAGGGAGCCTGAGAAA	20 (0.000383%)
TTTGCTGATGCGGGACGGAAGCTGGTCTCCCGTGTGTTACCGCACGCGGTTGGCCTAAATCCGAGCCAAGGACGCCTGGAGCGTACCGACATGCGGTGGT	32 (0.000613%)
TTTGGAGTCGAATATGACTTGATGTCATGTGTATGATTGA	327 (0.002507%)
TTTGGCTCGGATTCGTCCGTCTTCTTTCTTCTTAGCCGAGTACTTCGGTAGATTAGTTGGAACGATTGATGATTTTGAGTTAATTGAACGTTCGGCGTAT	26 (0.000498%)
TTTGGCTGGGGCGGCACATCTGTTAAAAGATAACGCAGGTGTCCTAAGATGAGCTCAACGAGAACAGAAATCTCGTGTGGAACAAAAGGGTAAAAGCTCG	16 (0.000307%)
TTTGGCTTTGTGTCTTCTAACAAGGAAACACTACTTAGGC	6775 (0.051936%)
TTTGTTTTAATTAAACAGTCGGATTCCCCTTGTCCGTACCAGTTCTGAGCTGACTGTTCGACGCCCGGGGAAAGCTCCCGAGAGAGCCGTTCCCAGTCCG	30 (0.000575%)
TTTTAATTAAACAGTCGGATTCCCCTTGTCCGTACCAGTTCTGAGCTGACTGTTCGACGCCCGGGGAAAGCTCCCGAGAGAGCCGTTCCCAGTCCGTCCC	13 (0.000249%)
TTTTATCGGTCGCTCTTGTCCGGAAGCTGTAGATGACCCAAAGTCCATATAGCGACCCCAGGTCAGGCGGGATTACCCGCTGAGTTTAAGCATATCAATA	8 (0.000153%)
TTTTCAAGGGCCGCCGGGGGCGCACCGGACACCACGCGACGTGCGGTGCTCTTCCAGCCGCTGGACCCTACCTCCGGCTGAGCCGTTTCCAGGGTGGGCA	23 (0.000441%)
TTTTCAGCAGTTCTCGGACAAAAATTGCTGAGTGGCCGAGAAGAATGGGCGTGTCATGCGTGGGCTGACATGGATTCTTCGAGGCCTAGGGGTGGCGGTA	4 (0.000077%)
TTTTGGAGTCGAATATGACTTGATGTCATGTGTATGATTG	480 (0.003680%)
TTTTTCAGCAGTTCTCGGACAAAAATTGCTGAGTGGCCGAGAAGAATGGGCGTGTCATGCGTGGGCTGACATGGATTCTTCGAGGCCTAGGGGTGGCGGT	21 (0.000402%)
TTTTTTCAGCAGTTCTCGGACAAAAATTGCTGAGTGGCCGAGAAGAATGGGCGTGTCATGCGTGGGCTGACATGGATTCTTCGAGGCCTAGGGGTGGCGG	3 (0.000057%)
TTTTTTTTTT	52066 (0.099782%)

```
fastp -p -i resources/raw_hic/CRR302669_1.fastq.gz -I resources/raw_hic/CRR302669_2.fastq.gz -o results/fastp/hic_trim_1.fastq.gz -O
results/fastp/hic_trim_2.fastq.gz --detect_adapter_for_pe --json results/fastp/hic_report_fastp.HiC.json --html
results/fastp/hic_report_fastp.HiC.html --thread 20

fastp 0.23.4, at 2024-04-29 23:10:53
```
