# Supplementary material for: Colora: a Snakemake workflow for complete chromosome-scale de novo genome assembly
Source: Bioinformatics. 2025 Apr 16;41(5):btaf175. doi: 10.1093/bioinformatics/btaf175 (PMC12065627; doi:10.1093/bioinformatics/btaf175)
Supplement: btaf175_Supplementary_Data [file btaf175_supplementary_data.zip › Additional_files/S9_fastp_M.domestica.pdf]

fastp report

Summary

General

**fastp version:**	0.23.4 (https://github.com/OpenGene/fastp)
**sequencing:**	paired end (150 cycles + 150 cycles)
**mean length before filtering:**	148bp, 148bp
**mean length after filtering:**	148bp, 148bp
**duplication rate:**	15.932978%
**Insert size peak:**	269

Before filtering

**total reads:**	676.416866 M
**total bases:**	100.522162 G
**Q20 bases:**	97.141880 G (96.637277%)
**Q30 bases:**	91.747105 G (91.270525%)
**GC content:**	38.625093%

After filtering

**total reads:**	670.577270 M
**total bases:**	99.631921 G
**Q20 bases:**	96.542553 G (96.899218%)
**Q30 bases:**	91.267955 G (91.605134%)
**GC content:**	38.614515%

Filtering result

**reads passed filters:**	670.577270 M (99.136687%)
**reads with low quality:**	5.839586 M (0.863312%)
**reads with too many N:**	10 (0.000001%)
**reads too short:**	0 (0.000000%)

Adapters

Adapter or bad ligation of read1

The input has little adapter percentage (~0.021127%), probably it's trimmed before.

Sequence	Occurrences
A	13923
AG	10829
AGA	10090
AGAT	9126
AGATC	7589
AGATCG	6282
AGATCGG	5910
AGATCGGA	5459
AGATCGGAA	4996
AGATCGGAAG	4442
other adapter sequences	350838

Adapter or bad ligation of read2

The input has little adapter percentage (~0.019260%), probably it's trimmed before.

Sequence	Occurrences
A	14491
AG	12850
AGA	12305
AGAT	11612
AGATC	10336
AGATCG	9425
AGATCGG	9149
AGATCGGA	8601
AGATCGGAA	8016
AGATCGGAAG	7382
AGATCGGAAGA	7323
AGATCGGAAGAG	6966
AGATCGGAAGAGC	6487
AGATCGGAAGAGCG	5979
AGATCGGAAGAGCGT	5416
AGATCGGAAGAGCGTC	6472
AGATCGGAAGAGCGTCG	4983
AGATCGGAAGAGCGTCGT	5269
AGATCGGAAGAGCGTCGTGTA	5089
AGATCGGAAGAGCGTCGTGTAGGGAAA	5615
other adapter sequences	266342

Insert size estimation

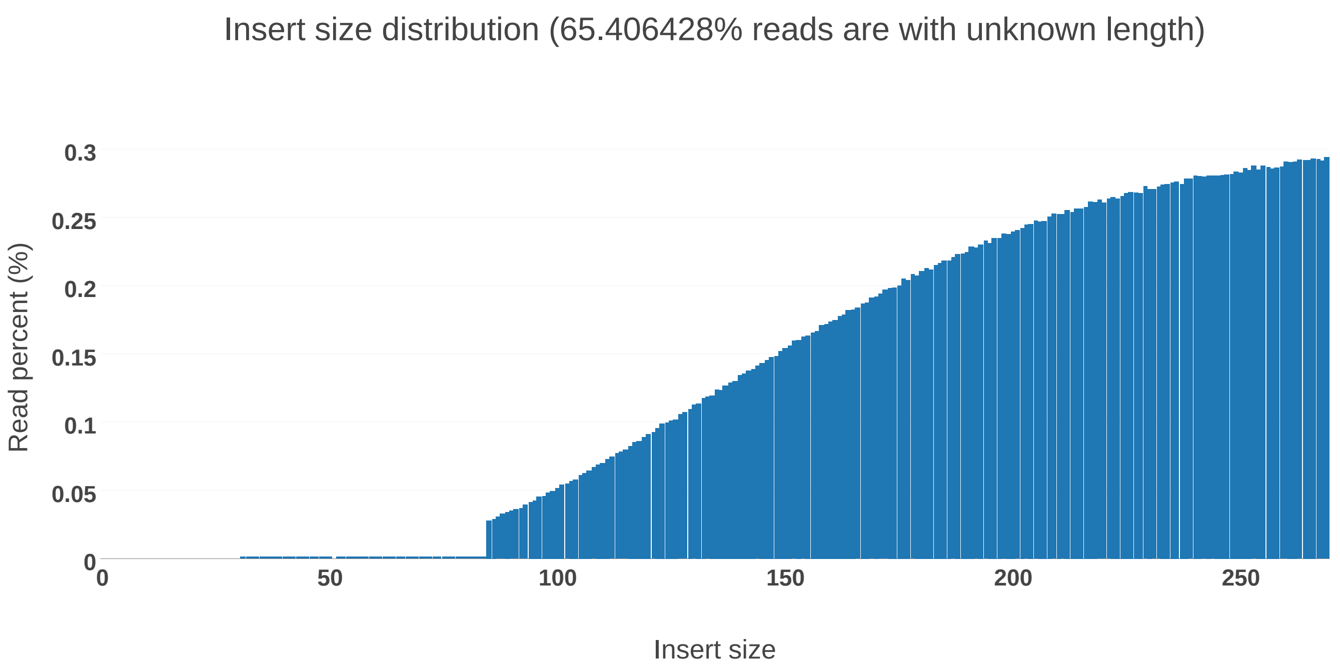

This estimation is based on paired-end overlap analysis, and there are 65.406428% reads found not overlapped.
The nonoverlapped read pairs may have insert size <30 or >270, or contain too much sequencing errors to be detected as overlapped.

Before filtering

Before filtering: read1: quality

Value of each position will be shown on mouse over.

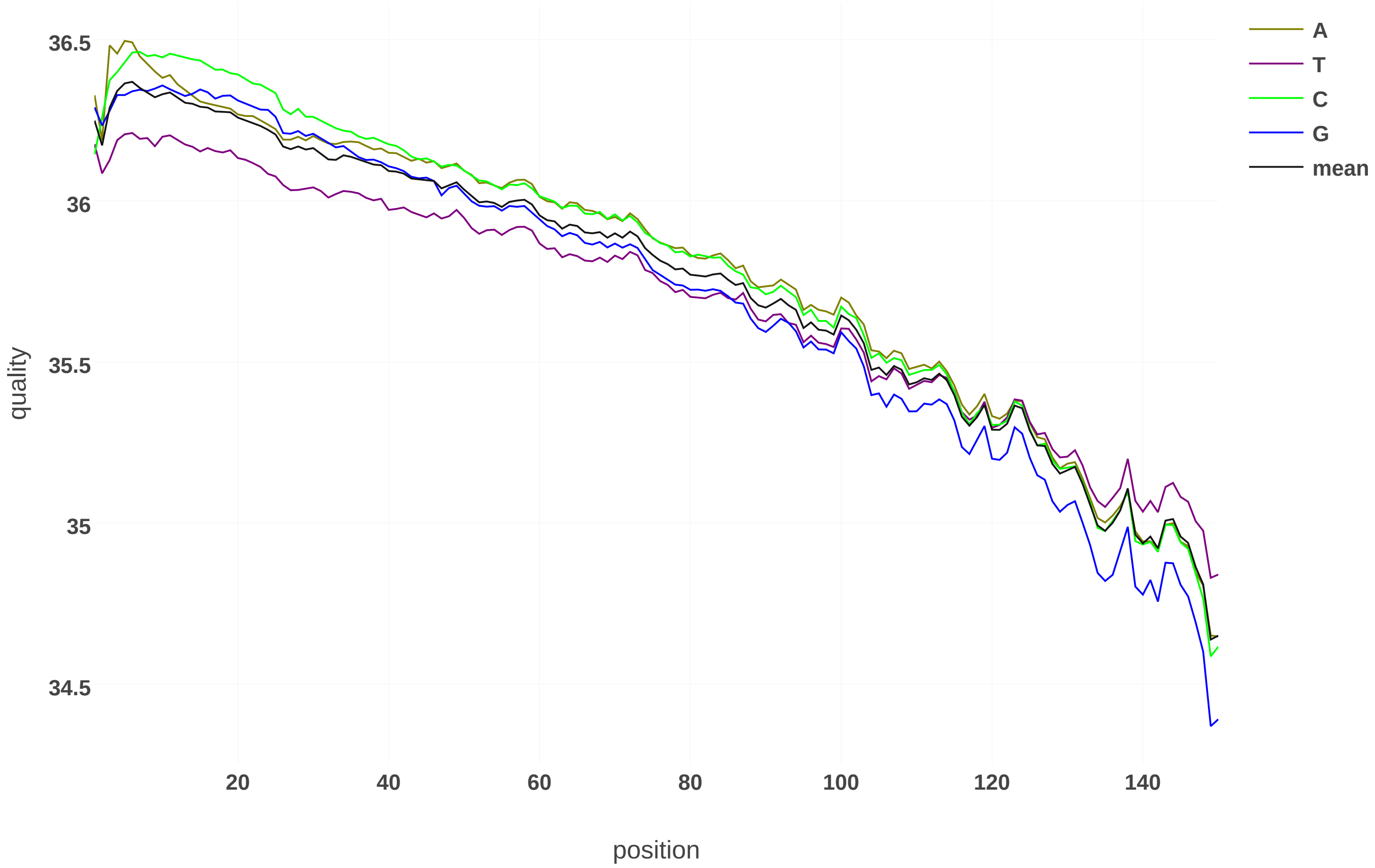

Before filtering: read1: base contents

Value of each position will be shown on mouse over.

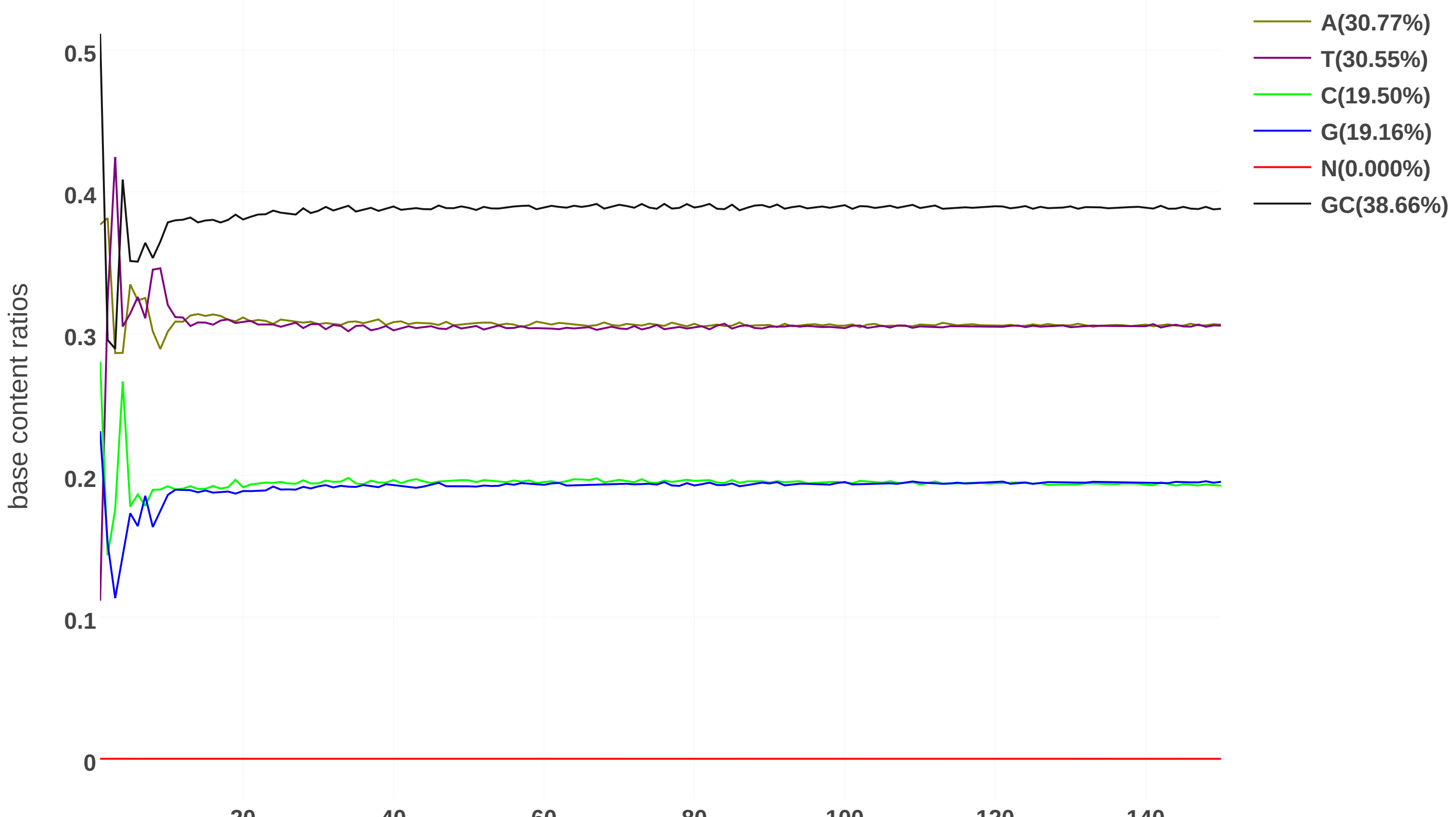

20 40 60 80 100 120 140

position

Before filtering: read1: KMER counting

Darker background means larger counts. The count will be shown on mouse over.

	AA	AT	AC	AG	TA	TT	TC	TG	CA	CT	CC	CG	GA	GT	GC	GG
AAA	AAAAA	AAAAT	AAAAC	AAAAG	AAATA	AAATT	AAATC	AAATG	AAACA	AAACT	AAACC	AAACG	AAAGA	AAAGT	AAAGC	AAAGG
AAT	AATAA	AATAT	AATAC	AATAG	AATTA	AATTT	AATTC	AATTG	AATCA	AATCT	AATCC	AATCG	AATGA	AATGT	AATGC	AATGG
AAC	AACAA	AACAT	AACAC	AACAG	AACTA	AACTT	AACTC	AACTG	AACCA	AACCT	AACCC	AACCG	AACGA	AACGT	AACGC	AACGG
AAG	AAGAA	AAGAT	AAGAC	AAGAG	AAGTA	AAGTT	AAGTC	AAGTG	AAGCA	AAGCT	AAGCC	AAGCG	AAGGA	AAGGT	AAGGC	AAGGG
ATA	ATAAA	ATAAT	ATAAC	ATAAG	ATATA	ATATT	ATATC	ATATG	ATACA	ATACT	ATACC	ATACG	ATAGA	ATAGT	ATAGC	ATAGG
ATT	ATTAA	ATTAT	ATTAC	ATTAG	ATTTA	ATTTT	ATTTC	ATTTG	ATTCA	ATTCT	ATTCC	ATTCG	ATTGA	ATTGT	ATTGC	ATTGG
ATC	ATCAA	ATCAT	ATCAC	ATCAG	ATCTA	ATCTT	ATCTC	ATCTG	ATCCA	ATCCT	ATCCC	ATCCG	ATCGA	ATCGT	ATCGC	ATCGG
ATG	ATGAA	ATGAT	ATGAC	ATGAG	ATGTA	ATGTT	ATGTC	ATGTG	ATGCA	ATGCT	ATGCC	ATGCG	ATGGA	ATGGT	ATGGC	ATGGG
ACA	ACAAA	ACAAT	ACAAC	ACAAG	ACATA	ACATT	ACATC	ACATG	ACACA	ACACT	ACACC	ACACG	ACAGA	ACAGT	ACAGC	ACAGG
ACT	ACTAA	ACTAT	ACTAC	ACTAG	ACTTA	ACTTT	ACTTC	ACTTG	ACTCA	ACTCT	ACTCC	ACTCG	ACTGA	ACTGT	ACTGC	ACTGG
ACC	ACCAA	ACCAT	ACCAC	ACCAG	ACCTA	ACCTT	ACCTC	ACCTG	ACCCA	ACCCT	ACCCC	ACCCG	ACCGA	ACCGT	ACCGC	ACCGG
ACG	ACGAA	ACGAT	ACGAC	ACGAG	ACGTA	ACGTT	ACGTC	ACGTG	ACGCA	ACGCT	ACGCC	ACGCG	ACGGA	ACGGT	ACGGC	ACGGG
AGA	AGAAA	AGAAT	AGAAC	AGAAG	AGATA	AGATT	AGATC	AGATG	AGACA	AGACT	AGACC	AGACG	AGAGA	AGAGT	AGAGC	AGAGG
AGT	AGTAA	AGTAT	AGTAC	AGTAG	AGTTA	AGTTT	AGTTC	AGTTG	AGTCA	AGTCT	AGTCC	AGTCG	AGTGA	AGTGT	AGTGC	AGTGG
AGC	AGCAA	AGCAT	AGCAC	AGCAG	AGCTA	AGCTT	AGCTC	AGCTG	AGCCA	AGCCT	AGCCC	AGCCG	AGCGA	AGCGT	AGCGC	AGCGG
AGG	AGGAA	AGGAT	AGGAC	AGGAG	AGGTA	AGGTT	AGGTC	AGGTG	AGGCA	AGGCT	AGGCC	AGGCG	AGGGA	AGGGT	AGGGC	AGGGG
TAA	TAAAA	TAAAT	TAAAC	TAAAG	TAATA	TAATT	TAATC	TAATG	TAACA	TAACT	TAACC	TAACG	TAAGA	TAAGT	TAAGC	TAAGG
TAT	TATAA	TATAT	TATAC	TATAG	TATTA	TATTT	TATTC	TATTG	TATCA	TATCT	TATCC	TATCG	TATGA	TATGT	TATGC	TATGG
TAC	TACAA	TACAT	TACAC	TACAG	TACTA	TACTT	TACTC	TACTG	TACCA	TACCT	TACCC	TACCG	TACGA	TACGT	TACGC	TACGG
TAG	TAGAA	TAGAT	TAGAC	TAGAG	TAGTA	TAGTT	TAGTC	TAGTG	TAGCA	TAGCT	TAGCC	TAGCG	TAGGA	TAGGT	TAGGC	TAGGG
TTA	TTAAA	TTAAT	TTAAC	TTAAG	TTATA	TTATT	TTATC	TTATG	TTACA	TTACT	TTACC	TTACG	TTAGA	TTAGT	TTAGC	TTAGG
TTT	TTTAA	TTTAT	TTTAC	TTTAG	TTTTA	TTTTT	TTTTC	TTTTG	TTTCA	TTTCT	TTTCC	TTTCG	TTTGA	TTTGT	TTTGC	TTTGG
TTC	TTCAA	TTCAT	TTCAC	TTCAG	TTCTA	TTCTT	TTCTC	TTCTG	TTCCA	TTCCT	TTCCC	TTCCG	TTCGA	TTCGT	TTCGC	TTCGG
TTG	TTGAA	TTGAT	TTGAC	TTGAG	TTGTA	TTGTT	TTGTC	TTGTG	TTGCA	TTGCT	TTGCC	TTGCG	TTGGA	TTGGT	TTGGC	TTGGG
TCA	TCAAA	TCAAT	TCAAC	TCAAG	TCATA	TCATT	TCATC	TCATG	TCACA	TCACT	TCACC	TCACG	TCAGA	TCAGT	TCAGC	TCAGG
TCT	TCTAA	TCTAT	TCTAC	TCTAG	TCTTA	TCTTT	TCTTC	TCTTG	TCTCA	TCTCT	TCTCC	TCTCG	TCTGA	TCTGT	TCTGC	TCTGG
TCC	TCCAA	TCCAT	TCCAC	TCCAG	TCCTA	TCCTT	TCCTC	TCCTG	TCCCA	TCCCT	TCCCC	TCCCG	TCCGA	TCCGT	TCCGC	TCCGG
TCG	TCGAA	TCGAT	TCGAC	TCGAG	TCGTA	TCGTT	TCGTC	TCGTG	TCGCA	TCGCT	TCGCC	TCGCG	TCGGA	TCGGT	TCGGC	TCGGG
TGA	TGAAA	TGAAT	TGAAC	TGAAG	TGATA	TGATT	TGATC	TGATG	TGACA	TGACT	TGACC	TGACG	TGAGA	TGAGT	TGAGC	TGAGG
TGT	TGTAA	TGTAT	TGTAC	TGTAG	TGTTA	TGTTT	TGTTC	TGTTG	TGTCA	TGTCT	TGTCC	TGTCG	TGTGA	TGTGT	TGTGC	TGTGG
TGC	TGCAA	TGCAT	TGCAC	TGCAG	TGCTA	TGCTT	TGCTC	TGCTG	TGCCA	TGCCT	TGCCC	TGCCG	TGCGA	TGCGT	TGCGC	TGCGG
TGG	TGGAA	TGGAT	TGGAC	TGGAG	TGGTA	TGGTT	TGGTC	TGGTG	TGGCA	TGGCT	TGGCC	TGGCG	TGGGA	TGGGT	TGGGC	TGGGG
CAA	CAAAA	CAAAT	CAAAC	CAAAG	CAATA	CAATT	CAATC	CAATG	CAACA	CAACT	CAACC	CAACG	CAAGA	CAAGT	CAAGC	CAAGG
CAT	CATAA	CATAT	CATAC	CATAG	CATTA	CATTT	CATTC	CATTG	CATCA	CATCT	CATCC	CATCG	CATGA	CATGT	CATGC	CATGG
CAC	CACAA	CACAT	CACAC	CACAG	CACTA	CACTT	CACTC	CACTG	CACCA	CACCT	CACCC	CACCG	CACGA	CACGT	CACGC	CACGG
CAG	CAGAA	CAGAT	CAGAC	CAGAG	CAGTA	CAGTT	CAGTC	CAGTG	CAGCA	CAGCT	CAGCC	CAGCG	CAGGA	CAGGT	CAGGC	CAGGG
CTA	CTAAA	CTAAT	CTAAC	CTAAG	CTATA	CTATT	CTATC	CTATG	CTACA	CTACT	CTACC	CTACG	CTAGA	CTAGT	CTAGC	CTAGG
CTT	CTTAA	CTTAT	CTTAC	CTTAG	CTTTA	CTTTT	CTTTC	CTTTG	CTTCA	CTTCT	CTTCC	CTTCG	CTTGA	CTTGT	CTTGC	CTTGG
CTC	CTCAA	CTCAT	CTCAC	CTCAG	CTCTA	CTCTT	CTCTC	CTCTG	CTCCA	CTCCT	CTCCC	CTCCG	CTCGA	CTCGT	CTCGC	CTCGG
CTG	CTGAA	CTGAT	CTGAC	CTGAG	CTGTA	CTGTT	CTGTC	CTGTG	CTGCA	CTGCT	CTGCC	CTGCG	CTGGA	CTGGT	CTGGC	CTGGG
CCA	CCAAA	CCAAT	CCAAC	CCAAG	CCATA	CCATT	CCATC	CCATG	CCACA	CCACT	CCACC	CCACG	CCAGA	CCAGT	CCAGC	CCAGG
CCT	CCTAA	CCTAT	CCTAC	CCTAG	CCTTA	CCTTT	CCTTC	CCTTG	CCTCA	CCTCT	CCTCC	CCTCG	CCTGA	CCTGT	CCTGC	CCTGG
CCC	CCCAA	CCCAT	CCCAC	CCCAG	CCCTA	CCCTT	CCCTC	CCCTG	CCCCA	CCCCT	CCCCC	CCCCG	CCCGA	CCCGT	CCCGC	CCCGG
CCG	CCGAA	CCGAT	CCGAC	CCGAG	CCGTA	CCGTT	CCGTC	CCGTG	CCGCA	CCGCT	CCGCC	CCGCG	CCGGA	CCGGT	CCGGC	CCGGG
CGA	CGAAA	CGAAT	CGAAC	CGAAG	CGATA	CGATT	CGATC	CGATG	CGACA	CGACT	CGACC	CGACG	CGAGA	CGAGT	CGAGC	CGAGG
CGT	CGTAA	CGTAT	CGTAC	CGTAG	CGTTA	CGTTT	CGTTC	CGTTG	CGTCA	CGTCT	CGTCC	CGTCG	CGTGA	CGTGT	CGTGC	CGTGG
CGC	CGCAA	CGCAT	CGCAC	CGCAG	CGCTA	CGCTT	CGCTC	CGCTG	CGCCA	CGCCT	CGCCC	CGCCG	CGCGA	CGCGT	CGCGC	CGCGG
CGG	CGGAA	CGGAT	CGGAC	CGGAG	CGGTA	CGGTT	CGGTC	CGGTG	CGGCA	CGGCT	CGGCC	CGGCG	CGGGA	CGGGT	CGGGC	CGGGG
GAA	GAAAA	GAAAT	GAAAC	GAAAG	GAATA	GAATT	GAATC	GAATG	GAACA	GAACT	GAACC	GAACG	GAAGA	GAAGT	GAAGC	GAAGG
GAT	GATAA	GATAT	GATAC	GATAG	GATTA	GATTT	GATTC	GATTG	GATCA	GATCT	GATCC	GATCG	GATGA	GATGT	GATGC	GATGG
GAC	GACAA	GACAT	GACAC	GACAG	GACTA	GACTT	GACTC	GACTG	GACCA	GACCT	GACCC	GACCG	GACGA	GACGT	GACGC	GACGG
GAG	GAGAA	GAGAT	GAGAC	GAGAG	GAGTA	GAGTT	GAGTC	GAGTG	GAGCA	GAGCT	GAGCC	GAGCG	GAGGA	GAGGT	GAGGC	GAGGG
GTA	GTAAA	GTAAT	GTAAC	GTAAG	GTATA	GTATT	GTATC	GTATG	GTACA	GTACT	GTACC	GTACG	GTAGA	GTAGT	GTAGC	GTAGG
GTT	GTTAA	GTTAT	GTTAC	GTTAG	GTTTA	GTTTT	GTTTC	GTTTG	GTTCA	GTTCT	GTTCC	GTTCG	GTTGA	GTTGT	GTTGC	GTTGG
GTC	GTCAA	GTCAT	GTCAC	GTCAG	GTCTA	GTCTT	GTCTC	GTCTG	GTCCA	GTCCT	GTCCC	GTCCG	GTCGA	GTCGT	GTCGC	GTCGG
GTG	GTGAA	GTGAT	GTGAC	GTGAG	GTGTA	GTGTT	GTGTC	GTGTG	GTGCA	GTGCT	GTGCC	GTGCG	GTGGA	GTGGT	GTGGC	GTGGG
GCA	GCAAA	GCAAT	GCAAC	GCAAG	GCATA	GCATT	GCATC	GCATG	GCACA	GCACT	GCACC	GCACG	GCAGA	GCAGT	GCAGC	GCAGG
GCT	GCTAA	GCTAT	GCTAC	GCTAG	GCTTA	GCTTT	GCTTC	GCTTG	GCTCA	GCTCT	GCTCC	GCTCG	GCTGA	GCTGT	GCTGC	GCTGG
GCC	GCCAA	GCCAT	GCCAC	GCCAG	GCCTA	GCCTT	GCCTC	GCCTG	GCCCA	GCCCT	GCCCC	GCCCG	GCCGA	GCCGT	GCCGC	GCCGG
GCG	GCGAA	GCGAT	GCGAC	GCGAG	GCGTA	GCGTT	GCGTC	GCGTG	GCGCA	GCGCT	GCGCC	GCGCG	GCGGA	GCGGT	GCGGC	GCGGG
GGA	GGAAA	GGAAT	GGAAC	GGAAG	GGATA	GGATT	GGATC	GGATG	GGACA	GGACT	GGACC	GGACG	GGAGA	GGAGT	GGAGC	GGAGG
GGT	GGTAA	GGTAT	GGTAC	GGTAG	GGTTA	GGTTT	GGTTC	GGTTG	GGTCA	GGTCT	GGTCC	GGTCG	GGTGA	GGTGT	GGTGC	GGTGG
GGC	GGCAA	GGCAT	GGCAC	GGCAG	GGCTA	GGCTT	GGCTC	GGCTG	GGCCA	GGCCT	GGCCC	GGCCG	GGCGA	GGCGT	GGCGC	GGCGG
GGG	GGGAA	GGGAT	GGGAC	GGGAG	GGGTA	GGGTT	GGGTC	GGGTG	GGGCA	GGGCT	GGGCC	GGGCG	GGGGA	GGGGT	GGGGC	GGGGG

Before filtering: read1: overrepresented sequences

Sampling rate: 1 / 20

overrepresented sequence	count (% of bases)	distribution: cycle 1 ~ cycle 150
AAAAAAAAAA	274736 (0.109319%)	
AAACTAACCTGTCTCACGACGGTCTAAACCCAGCTCACGTTCCCTATTGGTGGGTGAACAATCCAACACTTGGTGAATTCTGCTTCACAATGATAGGAAG	323 (0.001285%)	
AACCTGTCTCACGACGGTCTAAACCCAGCTCACGTTCCCTATTGGTGGGTGAACAATCCAACACTTGGTGAATTCTGCTTCACAATGATAGGAAGAGCCG	122 (0.000485%)	
AACTAACCTGTCTCACGACGGTCTAAACCCAGCTCACGTTCCCTATTGGTGGGTGAACAATCCAACACTTGGTGAATTCTGCTTCACAATGATAGGAAGA	120 (0.000477%)	
AAGATCGATC	1125372 (0.447793%)	
ACCTGTCTCACGACGGTCTAAACCCAGCTCACGTTCCCTATTGGTGGGTGAACAATCCAACACTTGGTGAATTCTGCTTCACAATGATAGGAAGAGCCGA	97 (0.000386%)	
ACGACGGTCTAAACCCAGCTCACGTTCCCTATTGGTGGGTGAACAATCCAACACTTGGTGAATTCTGCTTCACAATGATAGGAAGAGCCGACATCGAAGG	103 (0.000410%)	
ACGGTCTAAACCCAGCTCACGTTCCCTATTGGTGGGTGAACAATCCAACACTTGGTGAATTCTGCTTCACAATGATAGGAAGAGCCGACATCGAAGGATC	91 (0.000362%)	
ACTAACCTGTCTCACGACGGTCTAAACCCAGCTCACGTTCCCTATTGGTGGGTGAACAATCCAACACTTGGTGAATTCTGCTTCACAATGATAGGAAGAG	106 (0.000422%)	
AGGATCGATC	891180 (0.354606%)	

AGGGTAAAACTAACCTGTCTCACGACGGTCTAAACCCAGCTCACGTTCCCTATTGGTGGGTGAACAATCCAACACTTGGTGAATTCTGCTTCACAATGAT	121 (0.000481%)	
AGTAGGGTAAAACTAACCTGTCTCACGACGGTCTAAACCCAGCTCACGTTCCCTATTGGTGGGTGAACAATCCAACACTTGGTGAATTCTGCTTCACAAT	188 (0.000748%)	
ATCAGTAGGGTAAAACTAACCTGTCTCACGACGGTCTAAACCCAGCTCACGTTCCCTATTGGTGGGTGAACAATCCAACACTTGGTGAATTCTGCTTCAC	4519 (0.017981%)	
ATGATCGATC	1064344 (0.423510%)	
CACGACGGTCTAAACCCAGCTCACGTTCCCTATTGGTGGGTGAACAATCCAACACTTGGTGAATTCTGCTTCACAATGATAGGAAGAGCCGACATCGAAG	163 (0.000649%)	
CAGTAGGGTAAAACTAACCTGTCTCACGACGGTCTAAACCCAGCTCACGTTCCCTATTGGTGGGTGAACAATCCAACACTTGGTGAATTCTGCTTCACAA	80 (0.000318%)	
CCTGTCTCACGACGGTCTAAACCCAGCTCACGTTCCCTATTGGTGGGTGAACAATCCAACACTTGGTGAATTCTGCTTCACAATGATAGGAAGAGCCGAC	201 (0.000800%)	
CGACGGTCTAAACCCAGCTCACGTTCCCTATTGGTGGGTGAACAATCCAACACTTGGTGAATTCTGCTTCACAATGATAGGAAGAGCCGACATCGAAGGA	87 (0.000346%)	
CGGTCTAAACCCAGCTCACGTTCCCTATTGGTGGGTGAACAATCCAACACTTGGTGAATTCTGCTTCACAATGATAGGAAGAGCCGACATCGAAGGATCG	39 (0.000155%)	
CTAACCTGTCTCACGACGGTCTAAACCCAGCTCACGTTCCCTATTGGTGGGTGAACAATCCAACACTTGGTGAATTCTGCTTCACAATGATAGGAAGAGC	207 (0.000824%)	
CTCACGACGGTCTAAACCCAGCTCACGTTCCCTATTGGTGGGTGAACAATCCAACACTTGGTGAATTCTGCTTCACAATGATAGGAAGAGCCGACATCGA	221 (0.000879%)	
CTGTCTCACGACGGTCTAAACCCAGCTCACGTTCCCTATTGGTGGGTGAACAATCCAACACTTGGTGAATTCTGCTTCACAATGATAGGAAGAGCCGACA	140 (0.000557%)	
GACGGTCTAAACCCAGCTCACGTTCCCTATTGGTGGGTGAACAATCCAACACTTGGTGAATTCTGCTTCACAATGATAGGAAGAGCCGACATCGAAGGAT	167 (0.000665%)	
GATCGATCAA	438317 (0.174409%)	
GATCGATCAT	467813 (0.186146%)	
GATCGATCCT	446159 (0.177530%)	
GATCGATCTT	570665 (0.227071%)	
GGGTAAAACTAACCTGTCTCACGACGGTCTAAACCCAGCTCACGTTCCCTATTGGTGGGTGAACAATCCAACACTTGGTGAATTCTGCTTCACAATGATA	40 (0.000159%)	
GGTAAAACTAACCTGTCTCACGACGGTCTAAACCCAGCTCACGTTCCCTATTGGTGGGTGAACAATCCAACACTTGGTGAATTCTGCTTCACAATGATAG	85 (0.000338%)	
GGTCTAAACCCAGCTCACGTTCCCTATTGGTGGGTGAACAATCCAACACTTGGTGAATTCTGCTTCACAATGATAGGAAGAGCCGACATCGAAGGATCGA	144 (0.000573%)	
GTAGGGTAAAACTAACCTGTCTCACGACGGTCTAAACCCAGCTCACGTTCCCTATTGGTGGGTGAACAATCCAACACTTGGTGAATTCTGCTTCACAATG	36 (0.000143%)	
GTCTAAACCCAGCTCACGTTCCCTATTGGTGGGTGAACAATCCAACACTTGGTGAATTCTGCTTCACAATGATAGGAAGAGCCGACATCGAAGGATCGAT	113 (0.000450%)	
GTCTCACGACGGTCTAAACCCAGCTCACGTTCCCTATTGGTGGGTGAACAATCCAACACTTGGTGAATTCTGCTTCACAATGATAGGAAGAGCCGACATC	75 (0.000298%)	
TAACCTGTCTCACGACGGTCTAAACCCAGCTCACGTTCCCTATTGGTGGGTGAACAATCCAACACTTGGTGAATTCTGCTTCACAATGATAGGAAGAGCC	45 (0.000179%)	
TAGGGTAAAACTAACCTGTCTCACGACGGTCTAAACCCAGCTCACGTTCCCTATTGGTGGGTGAACAATCCAACACTTGGTGAATTCTGCTTCACAATGA	7 (0.000028%)	
TCACGACGGTCTAAACCCAGCTCACGTTCCCTATTGGTGGGTGAACAATCCAACACTTGGTGAATTCTGCTTCACAATGATAGGAAGAGCCGACATCGAA	10 (0.000040%)	
TCAGTAGGGTAAAACTAACCTGTCTCACGACGGTCTAAACCCAGCTCACGTTCCCTATTGGTGGGTGAACAATCCAACACTTGGTGAATTCTGCTTCACA	12 (0.000048%)	
TCTAAACCCAGCTCACGTTCCCTATTGGTGGGTGAACAATCCAACACTTGGTGAATTCTGCTTCACAATGATAGGAAGAGCCGACATCGAAGGATCGATC	39 (0.000155%)	
TCTCACGACGGTCTAAACCCAGCTCACGTTCCCTATTGGTGGGTGAACAATCCAACACTTGGTGAATTCTGCTTCACAATGATAGGAAGAGCCGACATCG	27 (0.000107%)	
TGATCGATCA	170240 (0.067740%)	
TGTCTCACGACGGTCTAAACCCAGCTCACGTTCCCTATTGGTGGGTGAACAATCCAACACTTGGTGAATTCTGCTTCACAATGATAGGAAGAGCCGACAT	118 (0.000470%)	
TTGATCGATC	1024205 (0.407538%)	
TTTTTTTTTTTTTTTTTTTT	13665 (0.010875%)	

Before filtering: read2: quality

Value of each position will be shown on mouse over.

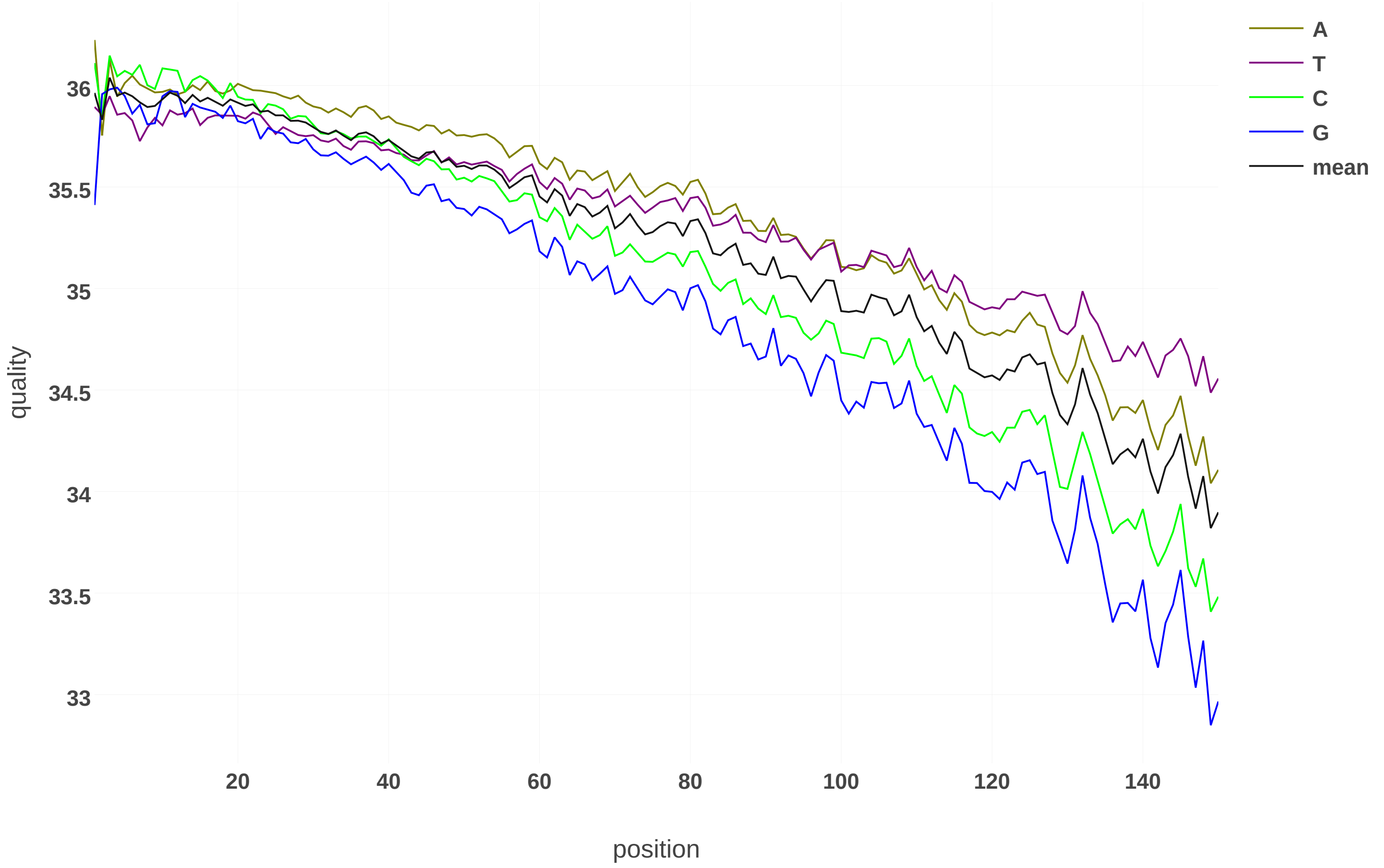

Before filtering: read2: base contents

Value of each position will be shown on mouse over.

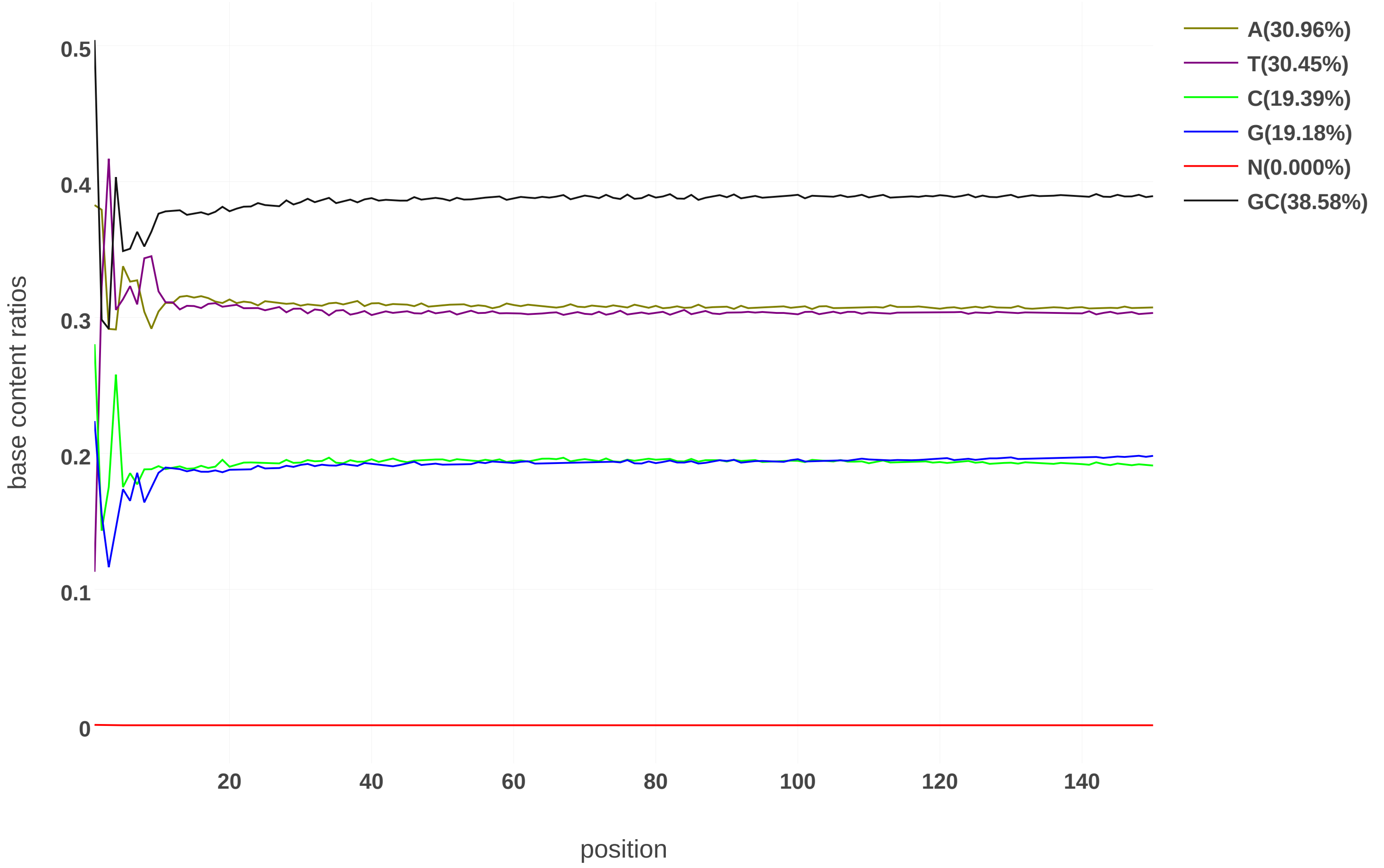

Before filtering: read2: KMER counting

Darker background means larger counts. The count will be shown on mouse over.

	AA	AT	AC	AG	TA	TT	TC	TG	CA	CT	CC	CG	GA	GT	GC	GG
AAA	AAAAA	AAAAT	AAAAC	AAAAG	AAATA	AAATT	AAATC	AAATG	AAACA	AAACT	AAACC	AAACG	AAAGA	AAAGT	AAAGC	AAAGG
AAT	AATAA	AATAT	AATAC	AATAG	AATTA	AATTT	AATTC	AATTG	AATCA	AATCT	AATCC	AATCG	AATGA	AATGT	AATGC	AATGG
AAC	AACAA	AACAT	AACAC	AACAG	AACTA	AACTT	AACTC	AACTG	AACCA	AACCT	AACCC	AACCG	AACGA	AACGT	AACGC	AACGG
AAG	AAGAA	AAGAT	AAGAC	AAGAG	AAGTA	AAGTT	AAGTC	AAGTG	AAGCA	AAGCT	AAGCC	AAGCG	AAGGA	AAGGT	AAGGC	AAGGG
ATA	ATAAA	ATAAT	ATAAC	ATAAG	ATATA	ATATT	ATATC	ATATG	ATACA	ATACT	ATACC	ATACG	ATAGA	ATAGT	ATAGC	ATAGG
ATT	ATTAA	ATTAT	ATTAC	ATTAG	ATTTA	ATTTT	ATTTC	ATTTG	ATTCA	ATTCT	ATTCC	ATTCG	ATTGA	ATTGT	ATTGC	ATTGG
ATC	ATCAA	ATCAT	ATCAC	ATCAG	ATCTA	ATCTT	ATCTC	ATCTG	ATCCA	ATCCT	ATCCC	ATCCG	ATCGA	ATCGT	ATCGC	ATCGG
ATG	ATGAA	ATGAT	ATGAC	ATGAG	ATGTA	ATGTT	ATGTC	ATGTG	ATGCA	ATGCT	ATGCC	ATGCG	ATGGA	ATGGT	ATGGC	ATGGG
ACA	ACAAA	ACAAT	ACAAC	ACAAG	ACATA	ACATT	ACATC	ACATG	ACACA	ACACT	ACACC	ACACG	ACAGA	ACAGT	ACAGC	ACAGG
ACT	ACTAA	ACTAT	ACTAC	ACTAG	ACTTA	ACTTT	ACTTC	ACTTG	ACTCA	ACTCT	ACTCC	ACTCG	ACTGA	ACTGT	ACTGC	ACTGG
ACC	ACCAA	ACCAT	ACCAC	ACCAG	ACCTA	ACCTT	ACCTC	ACCTG	ACCCA	ACCCT	ACCCC	ACCCG	ACCGA	ACCGT	ACCGC	ACCGG
ACG	ACGAA	ACGAT	ACGAC	ACGAG	ACGTA	ACGTT	ACGTC	ACGTG	ACGCA	ACGCT	ACGCC	ACGCG	ACGGA	ACGGT	ACGGC	ACGGG
AGA	AGAAA	AGAAT	AGAAC	AGAAG	AGATA	AGATT	AGATC	AGATG	AGACA	AGACT	AGACC	AGACG	AGAGA	AGAGT	AGAGC	AGAGG
AGT	AGTAA	AGTAT	AGTAC	AGTAG	AGTTA	AGTTT	AGTTC	AGTTG	AGTCA	AGTCT	AGTCC	AGTCG	AGTGA	AGTGT	AGTGC	AGTGG
AGC	AGCAA	AGCAT	AGCAC	AGCAG	AGCTA	AGCTT	AGCTC	AGCTG	AGCCA	AGCCT	AGCCC	AGCCG	AGCGA	AGCGT	AGCGC	AGCGG
AGG	AGGAA	AGGAT	AGGAC	AGGAG	AGGTA	AGGTT	AGGTC	AGGTG	AGGCA	AGGCT	AGGCC	AGGCG	AGGGA	AGGGT	AGGGC	AGGGG
TAA	TAAAA	TAAAT	TAAAC	TAAAG	TAATA	TAATT	TAATC	TAATG	TAACA	TAACT	TAACC	TAACG	TAAGA	TAAGT	TAAGC	TAAGG
TAT	TATAA	TATAT	TATAC	TATAG	TATTA	TATTT	TATTC	TATTG	TATCA	TATCT	TATCC	TATCG	TATGA	TATGT	TATGC	TATGG
TAC	TACAA	TACAT	TACAC	TACAG	TACTA	TACTT	TACTC	TACTG	TACCA	TACCT	TACCC	TACCG	TACGA	TACGT	TACGC	TACGG
TAG	TAGAA	TAGAT	TAGAC	TAGAG	TAGTA	TAGTT	TAGTC	TAGTG	TAGCA	TAGCT	TAGCC	TAGCG	TAGGA	TAGGT	TAGGC	TAGGG
TTA	TTAAA	TTAAT	TTAAC	TTAAG	TTATA	TTATT	TTATC	TTATG	TTACA	TTACT	TTACC	TTACG	TTAGA	TTAGT	TTAGC	TTAGG
TTT	TTTAA	TTTAT	TTTAC	TTTAG	TTTTA	TTTTT	TTTTC	TTTTG	TTTCA	TTTCT	TTTCC	TTTCG	TTTGA	TTTGT	TTTGC	TTTGG
TTC	TTCAA	TTCAT	TTCAC	TTCAG	TTCTA	TTCTT	TTCTC	TTCTG	TTCCA	TTCCT	TTCCC	TTCCG	TTCGA	TTCGT	TTCGC	TTCGG
TTG	TTGAA	TTGAT	TTGAC	TTGAG	TTGTA	TTGTT	TTGTC	TTGTG	TTGCA	TTGCT	TTGCC	TTGCG	TTGGA	TTGGT	TTGGC	TTGGG
TCA	TCAAA	TCAAT	TCAAC	TCAAG	TCATA	TCATT	TCATC	TCATG	TCACA	TCACT	TCACC	TCACG	TCAGA	TCAGT	TCAGC	TCAGG
TCT	TCTAA	TCTAT	TCTAC	TCTAG	TCTTA	TCTTT	TCTTC	TCTTG	TCTCA	TCTCT	TCTCC	TCTCG	TCTGA	TCTGT	TCTGC	TCTGG
TCC	TCCAA	TCCAT	TCCAC	TCCAG	TCCTA	TCCTT	TCCTC	TCCTG	TCCCA	TCCCT	TCCCC	TCCCG	TCCGA	TCCGT	TCCGC	TCCGG
TCG	TCGAA	TCGAT	TCGAC	TCGAG	TCGTA	TCGTT	TCGTC	TCGTG	TCGCA	TCGCT	TCGCC	TCGCG	TCGGA	TCGGT	TCGGC	TCGGG
TGA	TGAAA	TGAAT	TGAAC	TGAAG	TGATA	TGATT	TGATC	TGATG	TGACA	TGACT	TGACC	TGACG	TGAGA	TGAGT	TGAGC	TGAGG
TGT	TGTAA	TGTAT	TGTAC	TGTAG	TGTTA	TGTTT	TGTTC	TGTTG	TGTCA	TGTCT	TGTCC	TGTCG	TGTGA	TGTGT	TGTGC	TGTGG
TGC	TGCAA	TGCAT	TGCAC	TGCAG	TGCTA	TGCTT	TGCTC	TGCTG	TGCCA	TGCCT	TGCCC	TGCCG	TGCGA	TGCGT	TGCGC	TGCGG
TGG	TGGAA	TGGAT	TGGAC	TGGAG	TGGTA	TGGTT	TGGTC	TGGTG	TGGCA	TGGCT	TGGCC	TGGCG	TGGGA	TGGGT	TGGGC	TGGGG
CAA	CAAAA	CAAAT	CAAAC	CAAAG	CAATA	CAATT	CAATC	CAATG	CAACA	CAACT	CAACC	CAACG	CAAGA	CAAGT	CAAGC	CAAGG
CAT	CATAA	CATAT	CATAC	CATAG	CATTA	CATTT	CATTC	CATTG	CATCA	CATCT	CATCC	CATCG	CATGA	CATGT	CATGC	CATGG
CAC	CACAA	CACAT	CACAC	CACAG	CACTA	CACTT	CACTC	CACTG	CACCA	CACCT	CACCC	CACCG	CACGA	CACGT	CACGC	CACGG
CAG	CAGAA	CAGAT	CAGAC	CAGAG	CAGTA	CAGTT	CAGTC	CAGTG	CAGCA	CAGCT	CAGCC	CAGCG	CAGGA	CAGGT	CAGGC	CAGGG
CTA	CTAAA	CTAAT	CTAAC	CTAAG	CTATA	CTATT	CTATC	CTATG	CTACA	CTACT	CTACC	CTACG	CTAGA	CTAGT	CTAGC	CTAGG
CTT	CTTAA	CTTAT	CTTAC	CTTAG	CTTTA	CTTTT	CTTTC	CTTTG	CTTCA	CTTCT	CTTCC	CTTCG	CTTGA	CTTGT	CTTGC	CTTGG
CTC	CTCAA	CTCAT	CTCAC	CTCAG	CTCTA	CTCTT	CTCTC	CTCTG	CTCCA	CTCCT	CTCCC	CTCCG	CTCGA	CTCGT	CTCGC	CTCGG
CTG	CTGAA	CTGAT	CTGAC	CTGAG	CTGTA	CTGTT	CTGTC	CTGTG	CTGCA	CTGCT	CTGCC	CTGCG	CTGGA	CTGGT	CTGGC	CTGGG
CCA	CCAAA	CCAAT	CCAAC	CCAAG	CCATA	CCATT	CCATC	CCATG	CCACA	CCACT	CCACC	CCACG	CCAGA	CCAGT	CCAGC	CCAGG
CCT	CCTAA	CCTAT	CCTAC	CCTAG	CCTTA	CCTTT	CCTTC	CCTTG	CCTCA	CCTCT	CCTCC	CCTCG	CCTGA	CCTGT	CCTGC	CCTGG
CCC	CCCAA	CCCAT	CCCAC	CCCAG	CCCTA	CCCTT	CCCTC	CCCTG	CCCCA	CCCCT	CCCCC	CCCCG	CCCGA	CCCGT	CCCGC	CCCGG
CCG	CCGAA	CCGAT	CCGAC	CCGAG	CCGTA	CCGTT	CCGTC	CCGTG	CCGCA	CCGCT	CCGCC	CCGCG	CCGGA	CCGGT	CCGGC	CCGGG
CGA	CGAAA	CGAAT	CGAAC	CGAAG	CGATA	CGATT	CGATC	CGATG	CGACA	CGACT	CGACC	CGACG	CGAGA	CGAGT	CGAGC	CGAGG
CGT	CGTAA	CGTAT	CGTAC	CGTAG	CGTTA	CGTTT	CGTTC	CGTTG	CGTCA	CGTCT	CGTCC	CGTCG	CGTGA	CGTGT	CGTGC	CGTGG
CGC	CGCAA	CGCAT	CGCAC	CGCAG	CGCTA	CGCTT	CGCTC	CGCTG	CGCCA	CGCCT	CGCCC	CGCCG	CGCGA	CGCGT	CGCGC	CGCGG
CGG	CGGAA	CGGAT	CGGAC	CGGAG	CGGTA	CGGTT	CGGTC	CGGTG	CGGCA	CGGCT	CGGCC	CGGCG	CGGGA	CGGGT	CGGGC	CGGGG
GAA	GAAAA	GAAAT	GAAAC	GAAAG	GAATA	GAATT	GAATC	GAATG	GAACA	GAACT	GAACC	GAACG	GAAGA	GAAGT	GAAGC	GAAGG
GAT	GATAA	GATAT	GATAC	GATAG	GATTA	GATTT	GATTC	GATTG	GATCA	GATCT	GATCC	GATCG	GATGA	GATGT	GATGC	GATGG
GAC	GACAA	GACAT	GACAC	GACAG	GACTA	GACTT	GACTC	GACTG	GACCA	GACCT	GACCC	GACCG	GACGA	GACGT	GACGC	GACGG
GAG	GAGAA	GAGAT	GAGAC	GAGAG	GAGTA	GAGTT	GAGTC	GAGTG	GAGCA	GAGCT	GAGCC	GAGCG	GAGGA	GAGGT	GAGGC	GAGGG
GTA	GTAAA	GTAAT	GTAAC	GTAAG	GTATA	GTATT	GTATC	GTATG	GTACA	GTACT	GTACC	GTACG	GTAGA	GTAGT	GTAGC	GTAGG
GTT	GTTAA	GTTAT	GTTAC	GTTAG	GTTTA	GTTTT	GTTTC	GTTTG	GTTCA	GTTCT	GTTCC	GTTCG	GTTGA	GTTGT	GTTGC	GTTGG
GTC	GTCAA	GTCAT	GTCAC	GTCAG	GTCTA	GTCTT	GTCTC	GTCTG	GTCCA	GTCCT	GTCCC	GTCCG	GTCGA	GTCGT	GTCGC	GTCGG
GTG	GTGAA	GTGAT	GTGAC	GTGAG	GTGTA	GTGTT	GTGTC	GTGTG	GTGCA	GTGCT	GTGCC	GTGCG	GTGGA	GTGGT	GTGGC	GTGGG
GCA	GCAAA	GCAAT	GCAAC	GCAAG	GCATA	GCATT	GCATC	GCATG	GCACA	GCACT	GCACC	GCACG	GCAGA	GCAGT	GCAGC	GCAGG
GCT	GCTAA	GCTAT	GCTAC	GCTAG	GCTTA	GCTTT	GCTTC	GCTTG	GCTCA	GCTCT	GCTCC	GCTCG	GCTGA	GCTGT	GCTGC	GCTGG
GCC	GCCAA	GCCAT	GCCAC	GCCAG	GCCTA	GCCTT	GCCTC	GCCTG	GCCCA	GCCCT	GCCCC	GCCCG	GCCGA	GCCGT	GCCGC	GCCGG
GCG	GCGAA	GCGAT	GCGAC	GCGAG	GCGTA	GCGTT	GCGTC	GCGTG	GCGCA	GCGCT	GCGCC	GCGCG	GCGGA	GCGGT	GCGGC	GCGGG
GGA	GGAAA	GGAAT	GGAAC	GGAAG	GGATA	GGATT	GGATC	GGATG	GGACA	GGACT	GGACC	GGACG	GGAGA	GGAGT	GGAGC	GGAGG
GGT	GGTAA	GGTAT	GGTAC	GGTAG	GGTTA	GGTTT	GGTTC	GGTTG	GGTCA	GGTCT	GGTCC	GGTCG	GGTGA	GGTGT	GGTGC	GGTGG
GGC	GGCAA	GGCAT	GGCAC	GGCAG	GGCTA	GGCTT	GGCTC	GGCTG	GGCCA	GGCCT	GGCCC	GGCCG	GGCGA	GGCGT	GGCGC	GGCGG
GGG	GGGAA	GGGAT	GGGAC	GGGAG	GGGTA	GGGTT	GGGTC	GGGTG	GGGCA	GGGCT	GGGCC	GGGCG	GGGGA	GGGGT	GGGGC	GGGGG

Before filtering: read2: overrepresented sequences

Sampling rate: 1 / 20

overrepresented sequence	count (% of bases)	distribution: cycle 1 ~ cycle 150
AAAAAAAAAAAAAAAAAAAAAAAAAAAAAAAAAAAAAAAAAAAAAAAAAAAAAAAAAAAAAAAAAAAAAAAAAAAAAAAAAAAAAAAAAAAAAAAAAAAAAAAA	773 (0.003076%)	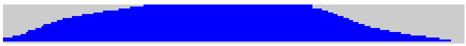
AAGATCGATC	1129958 (0.449653%)	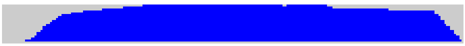
AGGATCGATC	887147 (0.353029%)	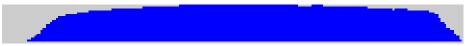
ATGATCGATC	1062878 (0.422959%)	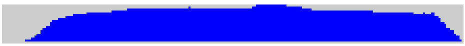
GATCGATCAA	436416 (0.173666%)	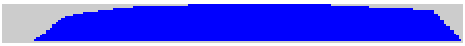
GATCGATCAT	464150 (0.184703%)	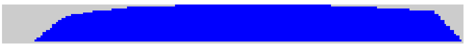
GATCGATCCT	440620 (0.175339%)	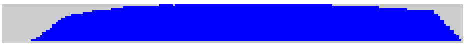
GATCGATCTT	558367 (0.222195%)	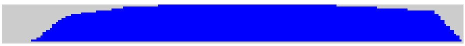
TGATCGATCA	170756 (0.067950%)	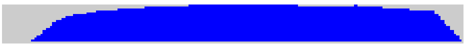
TTGATCGATC	1011707 (0.402596%)	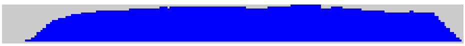
TTTTTTTTTT	245359 (0.097638%)	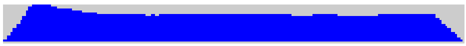

After filtering

After filtering: read1: quality

Value of each position will be shown on mouse over.

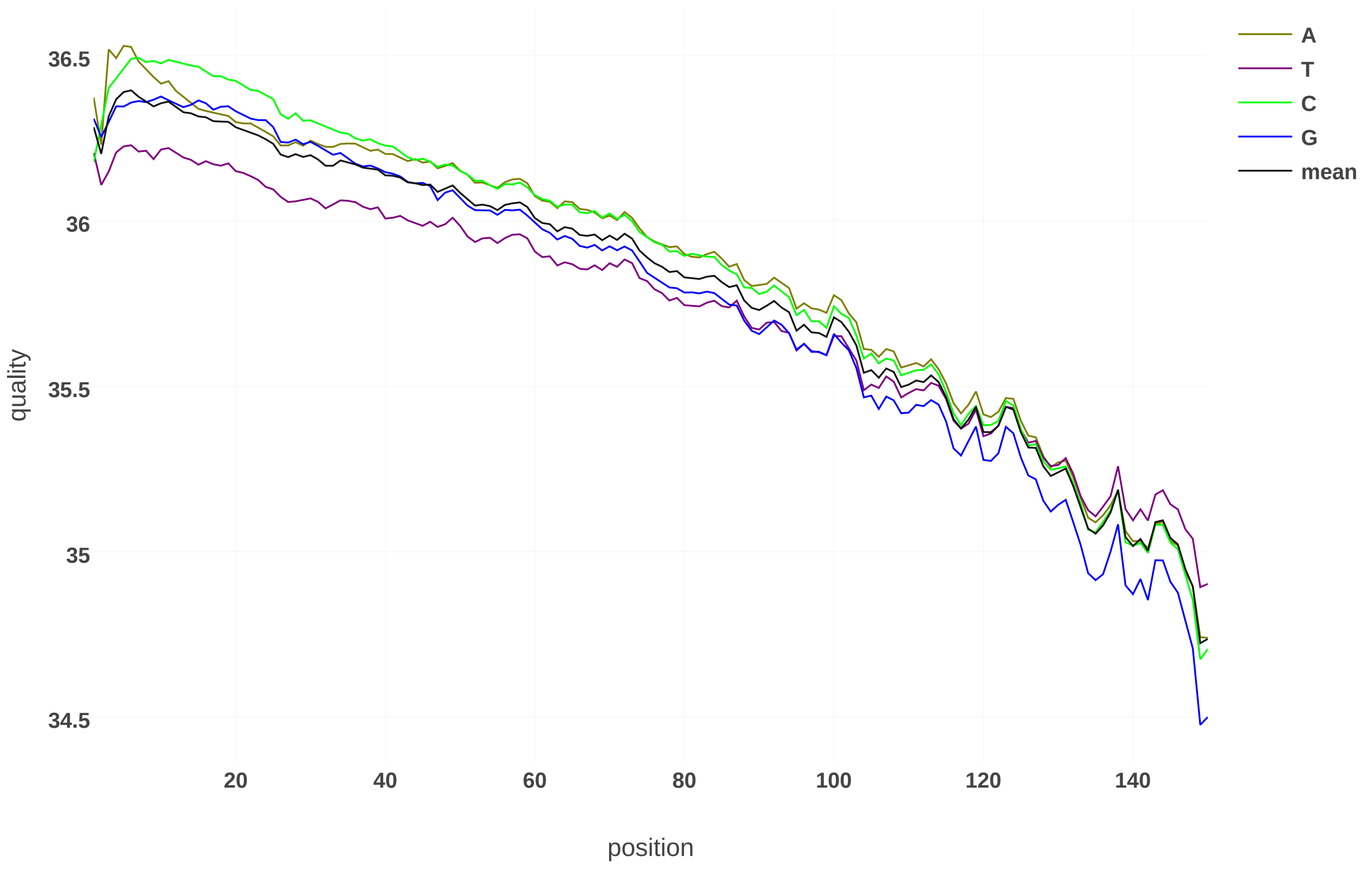

After filtering: read1: base contents

Value of each position will be shown on mouse over.

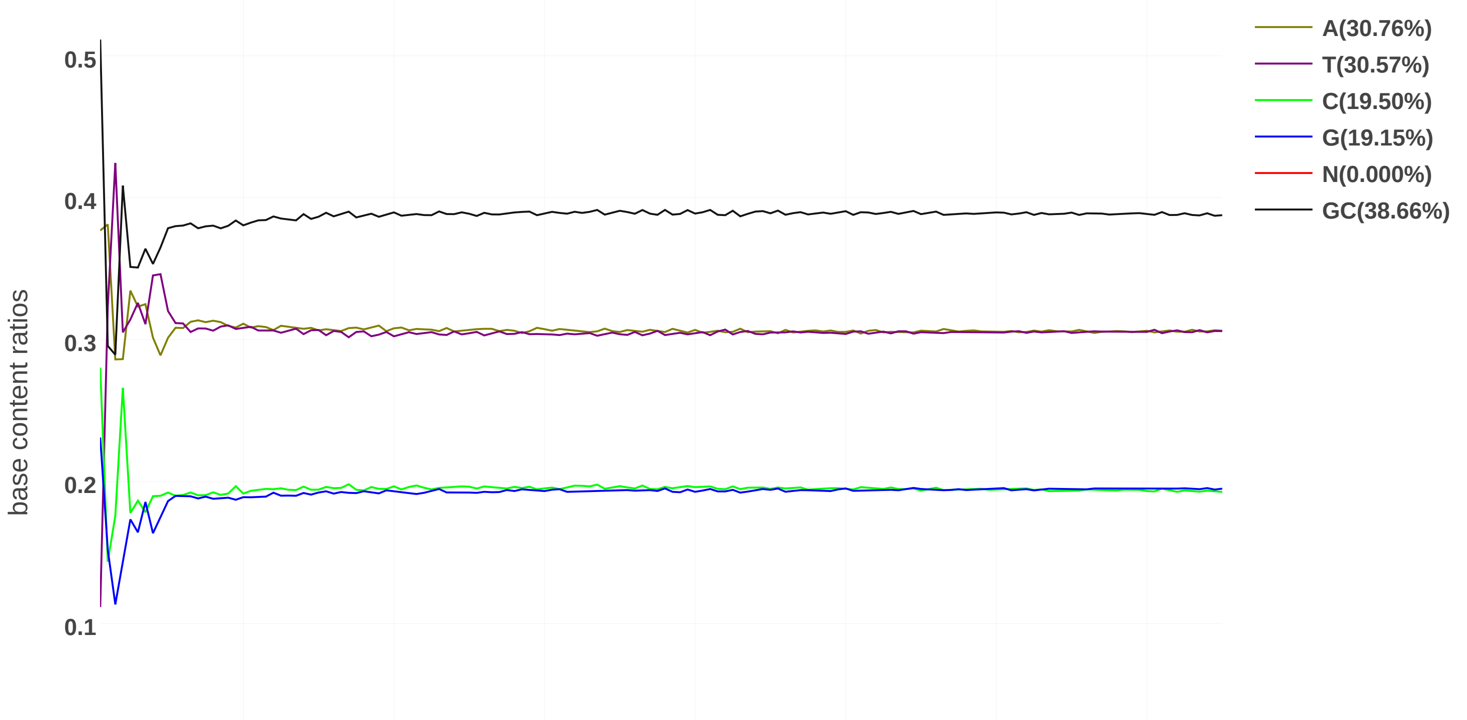

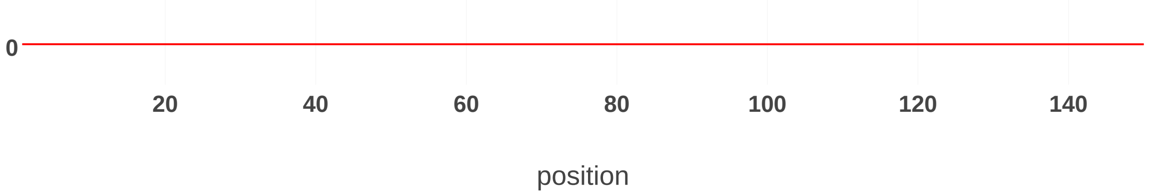

After filtering: read1: KMER counting

Darker background means larger counts. The count will be shown on mouse over.

	AA	AT	AC	AG	TA	TT	TC	TG	CA	CT	CC	CG	GA	GT	GC	GG
AAA	AAAAA	AAAAT	AAAAC	AAAAG	AAATA	AAATT	AAATC	AAATG	AAACA	AAACT	AAACC	AAACG	AAAGA	AAAGT	AAAGC	AAAGG
AAT	AATAA	AATAT	AATAC	AATAG	AATTA	AATTT	AATTC	AATTG	AATCA	AATCT	AATCC	AATCG	AATGA	AATGT	AATGC	AATGG
AAC	AACAA	AACAT	AACAC	AACAG	AACTA	AACTT	AACTC	AACTG	AACCA	AACCT	AACCC	AACCG	AACGA	AACGT	AACGC	AACGG
AAG	AAGAA	AAGAT	AAGAC	AAGAG	AAGTA	AAGTT	AAGTC	AAGTG	AAGCA	AAGCT	AAGCC	AAGCG	AAGGA	AAGGT	AAGGC	AAGGG
ATA	ATAAA	ATAAT	ATAAC	ATAAG	ATATA	ATATT	ATATC	ATATG	ATACA	ATACT	ATACC	ATACG	ATAGA	ATAGT	ATAGC	ATAGG
ATT	ATTAA	ATTAT	ATTAC	ATTAG	ATTTA	ATTTT	ATTTC	ATTTG	ATTCA	ATTCT	ATTCC	ATTCG	ATTGA	ATTGT	ATTGC	ATTGG
ATC	ATCAA	ATCAT	ATCAC	ATCAG	ATCTA	ATCTT	ATCTC	ATCTG	ATCCA	ATCCT	ATCCC	ATCCG	ATCGA	ATCGT	ATCGC	ATCGG
ATG	ATGAA	ATGAT	ATGAC	ATGAG	ATGTA	ATGTT	ATGTC	ATGTG	ATGCA	ATGCT	ATGCC	ATGCG	ATGGA	ATGGT	ATGGC	ATGGG
ACA	ACAAA	ACAAT	ACAAC	ACAAG	ACATA	ACATT	ACATC	ACATG	ACACA	ACACT	ACACC	ACACG	ACAGA	ACAGT	ACAGC	ACAGG
ACT	ACTAA	ACTAT	ACTAC	ACTAG	ACTTA	ACTTT	ACTTC	ACTTG	ACTCA	ACTCT	ACTCC	ACTCG	ACTGA	ACTGT	ACTGC	ACTGG
ACC	ACCAA	ACCAT	ACCAC	ACCAG	ACCTA	ACCTT	ACCTC	ACCTG	ACCCA	ACCCT	ACCCC	ACCCG	ACCGA	ACCGT	ACCGC	ACCGG
ACG	ACGAA	ACGAT	ACGAC	ACGAG	ACGTA	ACGTT	ACGTC	ACGTG	ACGCA	ACGCT	ACGCC	ACGCG	ACGGA	ACGGT	ACGGC	ACGGG
AGA	AGAAA	AGAAT	AGAAC	AGAAG	AGATA	AGATT	AGATC	AGATG	AGACA	AGACT	AGACC	AGACG	AGAGA	AGAGT	AGAGC	AGAGG
AGT	AGTAA	AGTAT	AGTAC	AGTAG	AGTTA	AGTTT	AGTTC	AGTTG	AGTCA	AGTCT	AGTCC	AGTCG	AGTGA	AGTGT	AGTGC	AGTGG
AGC	AGCAA	AGCAT	AGCAC	AGCAG	AGCTA	AGCTT	AGCTC	AGCTG	AGCCA	AGCCT	AGCCC	AGCCG	AGCGA	AGCGT	AGCGC	AGCGG
AGG	AGGAA	AGGAT	AGGAC	AGGAG	AGGTA	AGGTT	AGGTC	AGGTG	AGGCA	AGGCT	AGGCC	AGGCG	AGGGA	AGGGT	AGGGC	AGGGG
TAA	TAAAA	TAAAT	TAAAC	TAAAG	TAATA	TAATT	TAATC	TAATG	TAACA	TAACT	TAACC	TAACG	TAAGA	TAAGT	TAAGC	TAAGG
TAT	TATAA	TATAT	TATAC	TATAG	TATTA	TATTT	TATTC	TATTG	TATCA	TATCT	TATCC	TATCG	TATGA	TATGT	TATGC	TATGG
TAC	TACAA	TACAT	TACAC	TACAG	TACTA	TACTT	TACTC	TACTG	TACCA	TACCT	TACCC	TACCG	TACGA	TACGT	TACGC	TACGG
TAG	TAGAA	TAGAT	TAGAC	TAGAG	TAGTA	TAGTT	TAGTC	TAGTG	TAGCA	TAGCT	TAGCC	TAGCG	TAGGA	TAGGT	TAGGC	TAGGG
TTA	TTAAA	TTAAT	TTAAC	TTAAG	TTATA	TTATT	TTATC	TTATG	TTACA	TTACT	TTACC	TTACG	TTAGA	TTAGT	TTAGC	TTAGG
TTT	TTTAA	TTTAT	TTTAC	TTTAG	TTTTA	TTTTT	TTTTC	TTTTG	TTTCA	TTTCT	TTTCC	TTTCG	TTTGA	TTTGT	TTTGC	TTTGG
TTC	TTCAA	TTCAT	TTCAC	TTCAG	TTCTA	TTCTT	TTCTC	TTCTG	TTCCA	TTCCT	TTCCC	TTCCG	TTCGA	TTCGT	TTCGC	TTCGG
TTG	TTGAA	TTGAT	TTGAC	TTGAG	TTGTA	TTGTT	TTGTC	TTGTG	TTGCA	TTGCT	TTGCC	TTGCG	TTGGA	TTGGT	TTGGC	TTGGG
TCA	TCAAA	TCAAT	TCAAC	TCAAG	TCATA	TCATT	TCATC	TCATG	TCACA	TCACT	TCACC	TCACG	TCAGA	TCAGT	TCAGC	TCAGG
TCT	TCTAA	TCTAT	TCTAC	TCTAG	TCTTA	TCTTT	TCTTC	TCTTG	TCTCA	TCTCT	TCTCC	TCTCG	TCTGA	TCTGT	TCTGC	TCTGG
TCC	TCCAA	TCCAT	TCCAC	TCCAG	TCCTA	TCCTT	TCCTC	TCCTG	TCCCA	TCCCT	TCCCC	TCCCG	TCCGA	TCCGT	TCCGC	TCCGG
TCG	TCGAA	TCGAT	TCGAC	TCGAG	TCGTA	TCGTT	TCGTC	TCGTG	TCGCA	TCGCT	TCGCC	TCGCG	TCGGA	TCGGT	TCGGC	TCGGG
TGA	TGAAA	TGAAT	TGAAC	TGAAG	TGATA	TGATT	TGATC	TGATG	TGACA	TGACT	TGACC	TGACG	TGAGA	TGAGT	TGAGC	TGAGG
TGT	TGTAA	TGTAT	TGTAC	TGTAG	TGTTA	TGTTT	TGTTC	TGTTG	TGTCA	TGTCT	TGTCC	TGTCG	TGTGA	TGTGT	TGTGC	TGTGG
TGC	TGCAA	TGCAT	TGCAC	TGCAG	TGCTA	TGCTT	TGCTC	TGCTG	TGCCA	TGCCT	TGCCC	TGCCG	TGCGA	TGCGT	TGCGC	TGCGG
TGG	TGGAA	TGGAT	TGGAC	TGGAG	TGGTA	TGGTT	TGGTC	TGGTG	TGGCA	TGGCT	TGGCC	TGGCG	TGGGA	TGGGT	TGGGC	TGGGG
CAA	CAAAA	CAAAT	CAAAC	CAAAG	CAATA	CAATT	CAATC	CAATG	CAACA	CAACT	CAACC	CAACG	CAAGA	CAAGT	CAAGC	CAAGG
CAT	CATAA	CATAT	CATAC	CATAG	CATTA	CATTT	CATTC	CATTG	CATCA	CATCT	CATCC	CATCG	CATGA	CATGT	CATGC	CATGG
CAC	CACAA	CACAT	CACAC	CACAG	CACTA	CACTT	CACTC	CACTG	CACCA	CACCT	CACCC	CACCG	CACGA	CACGT	CACGC	CACGG
CAG	CAGAA	CAGAT	CAGAC	CAGAG	CAGTA	CAGTT	CAGTC	CAGTG	CAGCA	CAGCT	CAGCC	CAGCG	CAGGA	CAGGT	CAGGC	CAGGG
CTA	CTAAA	CTAAT	CTAAC	CTAAG	CTATA	CTATT	CTATC	CTATG	CTACA	CTACT	CTACC	CTACG	CTAGA	CTAGT	CTAGC	CTAGG
CTT	CTTAA	CTTAT	CTTAC	CTTAG	CTTTA	CTTTT	CTTTC	CTTTG	CTTCA	CTTCT	CTTCC	CTTCG	CTTGA	CTTGT	CTTGC	CTTGG
CTC	CTCAA	CTCAT	CTCAC	CTCAG	CTCTA	CTCTT	CTCTC	CTCTG	CTCCA	CTCCT	CTCCC	CTCCG	CTCGA	CTCGT	CTCGC	CTCGG
CTG	CTGAA	CTGAT	CTGAC	CTGAG	CTGTA	CTGTT	CTGTC	CTGTG	CTGCA	CTGCT	CTGCC	CTGCG	CTGGA	CTGGT	CTGGC	CTGGG
CCA	CCAAA	CCAAT	CCAAC	CCAAG	CCATA	CCATT	CCATC	CCATG	CCACA	CCACT	CCACC	CCACG	CCAGA	CCAGT	CCAGC	CCAGG
CCT	CCTAA	CCTAT	CCTAC	CCTAG	CCTTA	CCTTT	CCTTC	CCTTG	CCTCA	CCTCT	CCTCC	CCTCG	CCTGA	CCTGT	CCTGC	CCTGG
CCC	CCCAA	CCCAT	CCCAC	CCCAG	CCCTA	CCCTT	CCCTC	CCCTG	CCCCA	CCCCT	CCCCC	CCCCG	CCCGA	CCCGT	CCCGC	CCCGG
CCG	CCGAA	CCGAT	CCGAC	CCGAG	CCGTA	CCGTT	CCGTC	CCGTG	CCGCA	CCGCT	CCGCC	CCGCG	CCGGA	CCGGT	CCGGC	CCGGG
CGA	CGAAA	CGAAT	CGAAC	CGAAG	CGATA	CGATT	CGATC	CGATG	CGACA	CGACT	CGACC	CGACG	CGAGA	CGAGT	CGAGC	CGAGG
CGT	CGTAA	CGTAT	CGTAC	CGTAG	CGTTA	CGTTT	CGTTC	CGTTG	CGTCA	CGTCT	CGTCC	CGTCG	CGTGA	CGTGT	CGTGC	CGTGG
CGC	CGCAA	CGCAT	CGCAC	CGCAG	CGCTA	CGCTT	CGCTC	CGCTG	CGCCA	CGCCT	CGCCC	CGCCG	CGCGA	CGCGT	CGCGC	CGCGG
CGG	CGGAA	CGGAT	CGGAC	CGGAG	CGGTA	CGGTT	CGGTC	CGGTG	CGGCA	CGGCT	CGGCC	CGGCG	CGGGA	CGGGT	CGGGC	CGGGG
GAA	GAAAA	GAAAT	GAAAC	GAAAG	GAATA	GAATT	GAATC	GAATG	GAACA	GAACT	GAACC	GAACG	GAAGA	GAAGT	GAAGC	GAAGG
GAT	GATAA	GATAT	GATAC	GATAG	GATTA	GATTT	GATTC	GATTG	GATCA	GATCT	GATCC	GATCG	GATGA	GATGT	GATGC	GATGG
GAC	GACAA	GACAT	GACAC	GACAG	GACTA	GACTT	GACTC	GACTG	GACCA	GACCT	GACCC	GACCG	GACGA	GACGT	GACGC	GACGG
GAG	GAGAA	GAGAT	GAGAC	GAGAG	GAGTA	GAGTT	GAGTC	GAGTG	GAGCA	GAGCT	GAGCC	GAGCG	GAGGA	GAGGT	GAGGC	GAGGG
GTA	GTAAA	GTAAT	GTAAC	GTAAG	GTATA	GTATT	GTATC	GTATG	GTACA	GTACT	GTACC	GTACG	GTAGA	GTAGT	GTAGC	GTAGG
GTT	GTTAA	GTTAT	GTTAC	GTTAG	GTTTA	GTTTT	GTTTC	GTTTG	GTTCA	GTTCT	GTTCC	GTTCG	GTTGA	GTTGT	GTTGC	GTTGG
GTC	GTCAA	GTCAT	GTCAC	GTCAG	GTCTA	GTCTT	GTCTC	GTCTG	GTCCA	GTCCT	GTCCC	GTCCG	GTCGA	GTCGT	GTCGC	GTCGG
GTG	GTGAA	GTGAT	GTGAC	GTGAG	GTGTA	GTGTT	GTGTC	GTGTG	GTGCA	GTGCT	GTGCC	GTGCG	GTGGA	GTGGT	GTGGC	GTGGG
GCA	GCAAA	GCAAT	GCAAC	GCAAG	GCATA	GCATT	GCATC	GCATG	GCACA	GCACT	GCACC	GCACG	GCAGA	GCAGT	GCAGC	GCAGG
GCT	GCTAA	GCTAT	GCTAC	GCTAG	GCTTA	GCTTT	GCTTC	GCTTG	GCTCA	GCTCT	GCTCC	GCTCG	GCTGA	GCTGT	GCTGC	GCTGG
GCC	GCCAA	GCCAT	GCCAC	GCCAG	GCCTA	GCCTT	GCCTC	GCCTG	GCCCA	GCCCT	GCCCC	GCCCG	GCCGA	GCCGT	GCCGC	GCCGG
GCG	GCGAA	GCGAT	GCGAC	GCGAG	GCGTA	GCGTT	GCGTC	GCGTG	GCGCA	GCGCT	GCGCC	GCGCG	GCGGA	GCGGT	GCGGC	GCGGG
GGA	GGAAA	GGAAT	GGAAC	GGAAG	GGATA	GGATT	GGATC	GGATG	GGACA	GGACT	GGACC	GGACG	GGAGA	GGAGT	GGAGC	GGAGG
GGT	GGTAA	GGTAT	GGTAC	GGTAG	GGTTA	GGTTT	GGTTC	GGTTG	GGTCA	GGTCT	GGTCC	GGTCG	GGTGA	GGTGT	GGTGC	GGTGG
GGC	GGCAA	GGCAT	GGCAC	GGCAG	GGCTA	GGCTT	GGCTC	GGCTG	GGCCA	GGCCT	GGCCC	GGCCG	GGCGA	GGCGT	GGCGC	GGCGG
GGG	GGGAA	GGGAT	GGGAC	GGGAG	GGGTA	GGGTT	GGGTC	GGGTG	GGGCA	GGGCT	GGGCC	GGGCG	GGGGA	GGGGT	GGGGC	GGGGG

After filtering: read1: overrepresented sequences

Sampling rate: 1 / 20

overrepresented sequence	count (% of bases)	distribution: cycle 1 ~ cycle 150
AAAAAAAAAA	263012 (0.105592%)	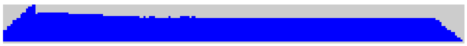
AAACTAACCTGTCTCACGACGGTCTAAACCCAGCTCACGTTCCCTATTGGTGGGTGAACAATCCAACACTTGGTGAATTCTGCTTCACAATGATAGGAAG	299 (0.001200%)	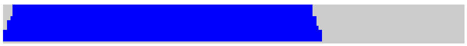
AACCTGTCTCACGACGGTCTAAACCCAGCTCACGTTCCCTATTGGTGGGTGAACAATCCAACACTTGGTGAATTCTGCTTCACAATGATAGGAAGAGCCG	100 (0.000401%)	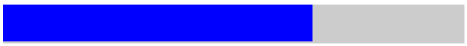
AACTAACCTGTCTCACGACGGTCTAAACCCAGCTCACGTTCCCTATTGGTGGGTGAACAATCCAACACTTGGTGAATTCTGCTTCACAATGATAGGAAGA	127 (0.000510%)	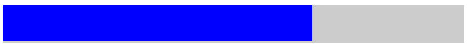
AAGATCGATC	1118795 (0.449167%)	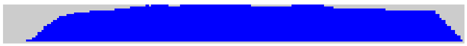
ACCTGTCTCACGACGGTCTAAACCCAGCTCACGTTCCCTATTGGTGGGTGAACAATCCAACACTTGGTGAATTCTGCTTCACAATGATAGGAAGAGCCGA	98 (0.000393%)	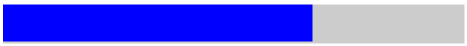
ACGACGGTCTAAACCCAGCTCACGTTCCCTATTGGTGGGTGAACAATCCAACACTTGGTGAATTCTGCTTCACAATGATAGGAAGAGCCGACATCGAAGG	96 (0.000385%)	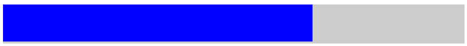
ACGGTCTAAACCCAGCTCACGTTCCCTATTGGTGGGTGAACAATCCAACACTTGGTGAATTCTGCTTCACAATGATAGGAAGAGCCGACATCGAAGGATC	95 (0.000381%)	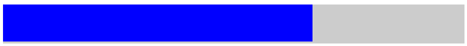

ACTAACCTGTCTCACGACGGTCTAAACCCAGCTCACGTTCCCTATTGGTGGGTGAACAATCCAACACTTGGTGAATTCTGCTTCACAATGATAGGAAGAG	114 (0.000458%)	
AGGATCGATC	887151 (0.356168%)	
AGGGTAAAACTAACCTGTCTCACGACGGTCTAAACCCAGCTCACGTTCCCTATTGGTGGGTGAACAATCCAACACTTGGTGAATTCTGCTTCACAATGAT	121 (0.000486%)	
AGTAGGGTAAAACTAACCTGTCTCACGACGGTCTAAACCCAGCTCACGTTCCCTATTGGTGGGTGAACAATCCAACACTTGGTGAATTCTGCTTCACAAT	181 (0.000727%)	
ATCAGTAGGGTAAAACTAACCTGTCTCACGACGGTCTAAACCCAGCTCACGTTCCCTATTGGTGGGTGAACAATCCAACACTTGGTGAATTCTGCTTCAC	4534 (0.018203%)	
ATGATCGATC	1058833 (0.425093%)	
CACGACGGTCTAAACCCAGCTCACGTTCCCTATTGGTGGGTGAACAATCCAACACTTGGTGAATTCTGCTTCACAATGATAGGAAGAGCCGACATCGAAG	139 (0.000558%)	
CAGTAGGGTAAAACTAACCTGTCTCACGACGGTCTAAACCCAGCTCACGTTCCCTATTGGTGGGTGAACAATCCAACACTTGGTGAATTCTGCTTCACAA	89 (0.000357%)	
CCTGTCTCACGACGGTCTAAACCCAGCTCACGTTCCCTATTGGTGGGTGAACAATCCAACACTTGGTGAATTCTGCTTCACAATGATAGGAAGAGCCGAC	213 (0.000855%)	
CGACGGTCTAAACCCAGCTCACGTTCCCTATTGGTGGGTGAACAATCCAACACTTGGTGAATTCTGCTTCACAATGATAGGAAGAGCCGACATCGAAGGA	77 (0.000309%)	
CGGTCTAAACCCAGCTCACGTTCCCTATTGGTGGGTGAACAATCCAACACTTGGTGAATTCTGCTTCACAATGATAGGAAGAGCCGACATCGAAGGATCG	48 (0.000193%)	
CTAACCTGTCTCACGACGGTCTAAACCCAGCTCACGTTCCCTATTGGTGGGTGAACAATCCAACACTTGGTGAATTCTGCTTCACAATGATAGGAAGAGC	173 (0.000695%)	
CTCACGACGGTCTAAACCCAGCTCACGTTCCCTATTGGTGGGTGAACAATCCAACACTTGGTGAATTCTGCTTCACAATGATAGGAAGAGCCGACATCGA	240 (0.000964%)	
CTGTCTCACGACGGTCTAAACCCAGCTCACGTTCCCTATTGGTGGGTGAACAATCCAACACTTGGTGAATTCTGCTTCACAATGATAGGAAGAGCCGACA	144 (0.000578%)	
GACGGTCTAAACCCAGCTCACGTTCCCTATTGGTGGGTGAACAATCCAACACTTGGTGAATTCTGCTTCACAATGATAGGAAGAGCCGACATCGAAGGAT	171 (0.000687%)	
GATCGATCAA	436700 (0.175324%)	
GATCGATCAT	463213 (0.185968%)	
GATCGATCCT	443416 (0.178020%)	
GATCGATCTT	566315 (0.227360%)	
GGGTAAAACTAACCTGTCTCACGACGGTCTAAACCCAGCTCACGTTCCCTATTGGTGGGTGAACAATCCAACACTTGGTGAATTCTGCTTCACAATGATA	53 (0.000213%)	
GGTAAAACTAACCTGTCTCACGACGGTCTAAACCCAGCTCACGTTCCCTATTGGTGGGTGAACAATCCAACACTTGGTGAATTCTGCTTCACAATGATAG	74 (0.000297%)	
GGTCTAAACCCAGCTCACGTTCCCTATTGGTGGGTGAACAATCCAACACTTGGTGAATTCTGCTTCACAATGATAGGAAGAGCCGACATCGAAGGATCGA	161 (0.000646%)	
GTAGGGTAAAACTAACCTGTCTCACGACGGTCTAAACCCAGCTCACGTTCCCTATTGGTGGGTGAACAATCCAACACTTGGTGAATTCTGCTTCACAATG	42 (0.000169%)	
GTCTAAACCCAGCTCACGTTCCCTATTGGTGGGTGAACAATCCAACACTTGGTGAATTCTGCTTCACAATGATAGGAAGAGCCGACATCGAAGGATCGAT	120 (0.000482%)	
GTCTCACGACGGTCTAAACCCAGCTCACGTTCCCTATTGGTGGGTGAACAATCCAACACTTGGTGAATTCTGCTTCACAATGATAGGAAGAGCCGACATC	75 (0.000301%)	
TAACCTGTCTCACGACGGTCTAAACCCAGCTCACGTTCCCTATTGGTGGGTGAACAATCCAACACTTGGTGAATTCTGCTTCACAATGATAGGAAGAGCC	45 (0.000181%)	
TAGGGTAAAACTAACCTGTCTCACGACGGTCTAAACCCAGCTCACGTTCCCTATTGGTGGGTGAACAATCCAACACTTGGTGAATTCTGCTTCACAATGA	14 (0.000056%)	
TCACGACGGTCTAAACCCAGCTCACGTTCCCTATTGGTGGGTGAACAATCCAACACTTGGTGAATTCTGCTTCACAATGATAGGAAGAGCCGACATCGAA	7 (0.000028%)	
TCAGTAGGGTAAAACTAACCTGTCTCACGACGGTCTAAACCCAGCTCACGTTCCCTATTGGTGGGTGAACAATCCAACACTTGGTGAATTCTGCTTCACA	11 (0.000044%)	
TCTAAACCCAGCTCACGTTCCCTATTGGTGGGTGAACAATCCAACACTTGGTGAATTCTGCTTCACAATGATAGGAAGAGCCGACATCGAAGGATCGATC	48 (0.000193%)	
TCTCACGACGGTCTAAACCCAGCTCACGTTCCCTATTGGTGGGTGAACAATCCAACACTTGGTGAATTCTGCTTCACAATGATAGGAAGAGCCGACATCG	41 (0.000165%)	
TGATCGATCA	168554 (0.067670%)	
TGTCTCACGACGGTCTAAACCCAGCTCACGTTCCCTATTGGTGGGTGAACAATCCAACACTTGGTGAATTCTGCTTCACAATGATAGGAAGAGCCGACAT	115 (0.000462%)	
TTGATCGATC	1018395 (0.408859%)	
TTTTTTTTTTTTTTTTTTTT	12294 (0.009871%)	

After filtering: read2: quality

Value of each position will be shown on mouse over.

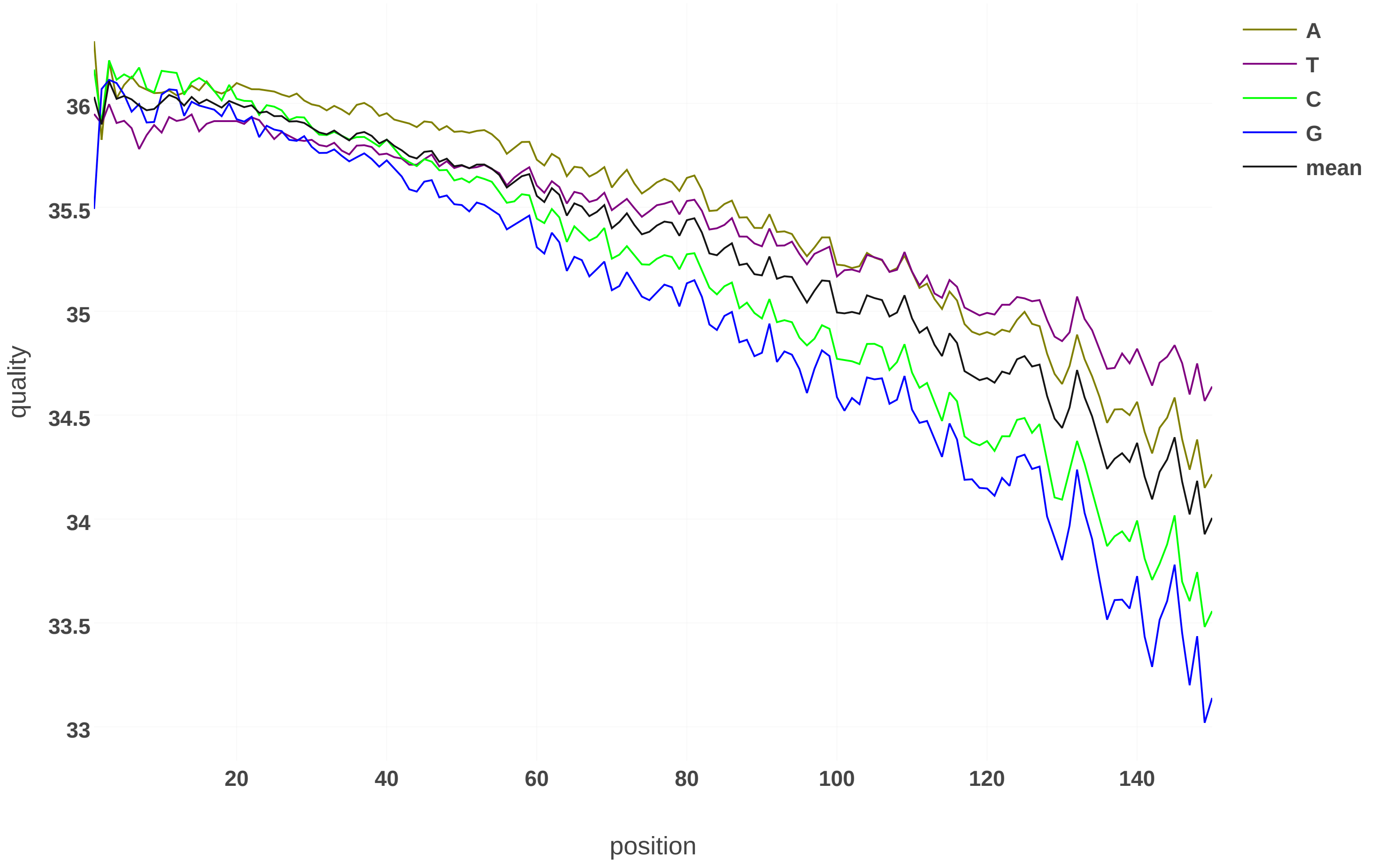

After filtering: read2: base contents

Value of each position will be shown on mouse over.

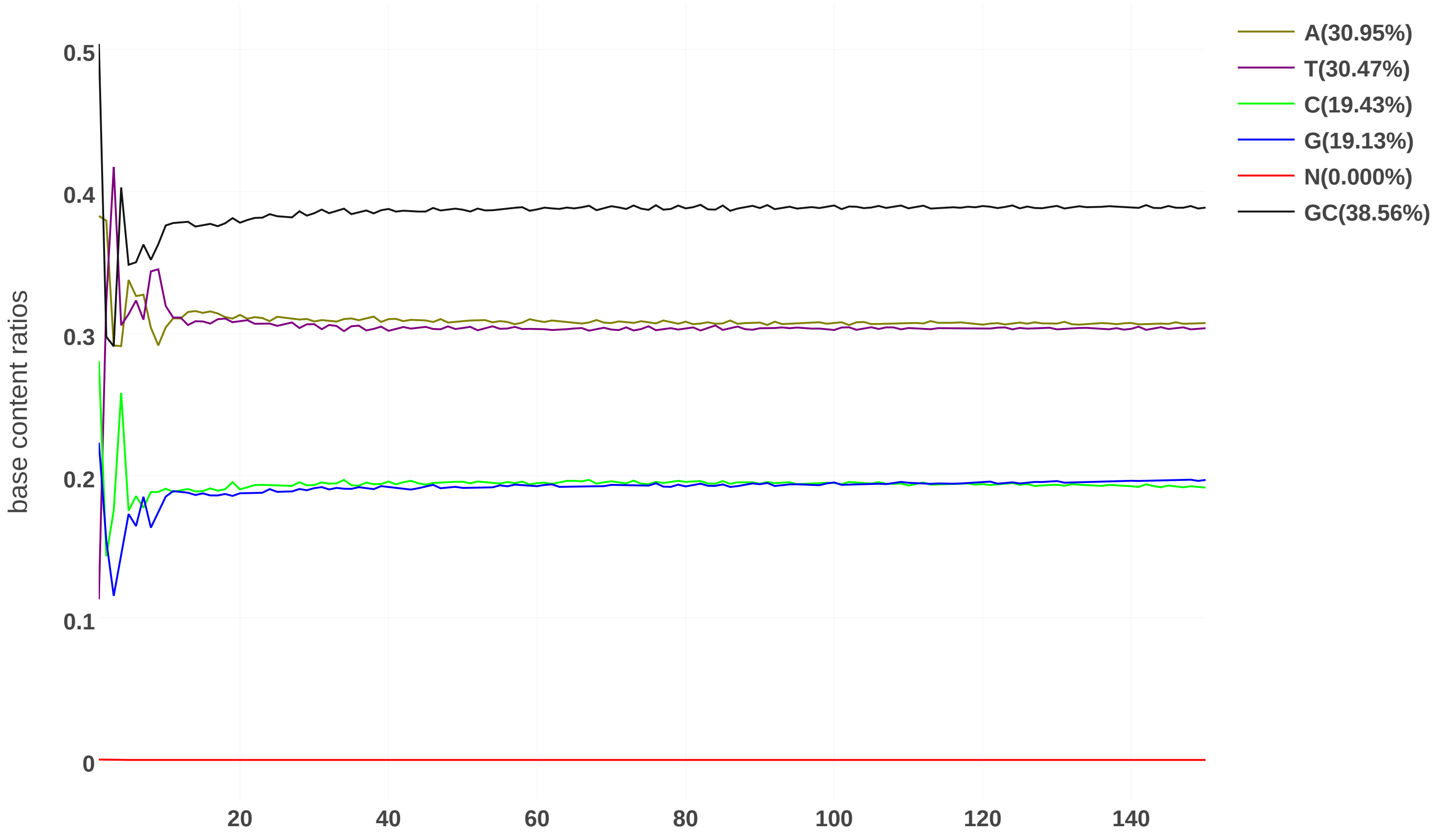

position

After filtering: read2: KMER counting

Darker background means larger counts. The count will be shown on mouse over.

	AA	AT	AC	AG	TA	TT	TC	TG	CA	CT	CC	CG	GA	GT	GC	GG
AAA	AAAAA	AAAAT	AAAAC	AAAAG	AAATA	AAATT	AAATC	AAATG	AAACA	AAACT	AAACC	AAACG	AAAGA	AAAGT	AAAGC	AAAGG
AAT	AATAA	AATAT	AATAC	AATAG	AATTA	AATTT	AATTC	AATTG	AATCA	AATCT	AATCC	AATCG	AATGA	AATGT	AATGC	AATGG
AAC	AACAA	AACAT	AACAC	AACAG	AACTA	AACTT	AACTC	AACTG	AACCA	AACCT	AACCC	AACCG	AACGA	AACGT	AACGC	AACGG
AAG	AAGAA	AAGAT	AAGAC	AAGAG	AAGTA	AAGTT	AAGTC	AAGTG	AAGCA	AAGCT	AAGCC	AAGCG	AAGGA	AAGGT	AAGGC	AAGGG
ATA	ATAAA	ATAAT	ATAAC	ATAAG	ATATA	ATATT	ATATC	ATATG	ATACA	ATACT	ATACC	ATACG	ATAGA	ATAGT	ATAGC	ATAGG
ATT	ATTAA	ATTAT	ATTAC	ATTAG	ATTTA	ATTTT	ATTTC	ATTTG	ATTCA	ATTCT	ATTCC	ATTCG	ATTGA	ATTGT	ATTGC	ATTGG
ATC	ATCAA	ATCAT	ATCAC	ATCAG	ATCTA	ATCTT	ATCTC	ATCTG	ATCCA	ATCCT	ATCCC	ATCCG	ATCGA	ATCGT	ATCGC	ATCGG
ATG	ATGAA	ATGAT	ATGAC	ATGAG	ATGTA	ATGTT	ATGTC	ATGTG	ATGCA	ATGCT	ATGCC	ATGCG	ATGGA	ATGGT	ATGGC	ATGGG
ACA	ACAAA	ACAAT	ACAAC	ACAAG	ACATA	ACATT	ACATC	ACATG	ACACA	ACACT	ACACC	ACACG	ACAGA	ACAGT	ACAGC	ACAGG
ACT	ACTAA	ACTAT	ACTAC	ACTAG	ACTTA	ACTTT	ACTTC	ACTTG	ACTCA	ACTCT	ACTCC	ACTCG	ACTGA	ACTGT	ACTGC	ACTGG
ACC	ACCAA	ACCAT	ACCAC	ACCAG	ACCTA	ACCTT	ACCTC	ACCTG	ACCCA	ACCCT	ACCCC	ACCCG	ACCGA	ACCGT	ACCGC	ACCGG
ACG	ACGAA	ACGAT	ACGAC	ACGAG	ACGTA	ACGTT	ACGTC	ACGTG	ACGCA	ACGCT	ACGCC	ACGCG	ACGGA	ACGGT	ACGGC	ACGGG
AGA	AGAAA	AGAAT	AGAAC	AGAAG	AGATA	AGATT	AGATC	AGATG	AGACA	AGACT	AGACC	AGACG	AGAGA	AGAGT	AGAGC	AGAGG
AGT	AGTAA	AGTAT	AGTAC	AGTAG	AGTTA	AGTTT	AGTTC	AGTTG	AGTCA	AGTCT	AGTCC	AGTCG	AGTGA	AGTGT	AGTGC	AGTGG
AGC	AGCAA	AGCAT	AGCAC	AGCAG	AGCTA	AGCTT	AGCTC	AGCTG	AGCCA	AGCCT	AGCCC	AGCCG	AGCGA	AGCGT	AGCGC	AGCGG
AGG	AGGAA	AGGAT	AGGAC	AGGAG	AGGTA	AGGTT	AGGTC	AGGTG	AGGCA	AGGCT	AGGCC	AGGCG	AGGGA	AGGGT	AGGGC	AGGGG
TAA	TAAAA	TAAAT	TAAAC	TAAAG	TAATA	TAATT	TAATC	TAATG	TAACA	TAACT	TAACC	TAACG	TAAGA	TAAGT	TAAGC	TAAGG
TAT	TATAA	TATAT	TATAC	TATAG	TATTA	TATTT	TATTC	TATTG	TATCA	TATCT	TATCC	TATCG	TATGA	TATGT	TATGC	TATGG
TAC	TACAA	TACAT	TACAC	TACAG	TACTA	TACTT	TACTC	TACTG	TACCA	TACCT	TACCC	TACCG	TACGA	TACGT	TACGC	TACGG
TAG	TAGAA	TAGAT	TAGAC	TAGAG	TAGTA	TAGTT	TAGTC	TAGTG	TAGCA	TAGCT	TAGCC	TAGCG	TAGGA	TAGGT	TAGGC	TAGGG
TTA	TTAAA	TTAAT	TTAAC	TTAAG	TTATA	TTATT	TTATC	TTATG	TTACA	TTACT	TTACC	TTACG	TTAGA	TTAGT	TTAGC	TTAGG
TTT	TTTAA	TTTAT	TTTAC	TTTAG	TTTTA	TTTTT	TTTTC	TTTTG	TTTCA	TTTCT	TTTCC	TTTCG	TTTGA	TTTGT	TTTGC	TTTGG
TTC	TTCAA	TTCAT	TTCAC	TTCAG	TTCTA	TTCTT	TTCTC	TTCTG	TTCCA	TTCCT	TTCCC	TTCCG	TTCGA	TTCGT	TTCGC	TTCGG
TTG	TTGAA	TTGAT	TTGAC	TTGAG	TTGTA	TTGTT	TTGTC	TTGTG	TTGCA	TTGCT	TTGCC	TTGCG	TTGGA	TTGGT	TTGGC	TTGGG
TCA	TCAAA	TCAAT	TCAAC	TCAAG	TCATA	TCATT	TCATC	TCATG	TCACA	TCACT	TCACC	TCACG	TCAGA	TCAGT	TCAGC	TCAGG
TCT	TCTAA	TCTAT	TCTAC	TCTAG	TCTTA	TCTTT	TCTTC	TCTTG	TCTCA	TCTCT	TCTCC	TCTCG	TCTGA	TCTGT	TCTGC	TCTGG
TCC	TCCAA	TCCAT	TCCAC	TCCAG	TCCTA	TCCTT	TCCTC	TCCTG	TCCCA	TCCCT	TCCCC	TCCCG	TCCGA	TCCGT	TCCGC	TCCGG
TCG	TCGAA	TCGAT	TCGAC	TCGAG	TCGTA	TCGTT	TCGTC	TCGTG	TCGCA	TCGCT	TCGCC	TCGCG	TCGGA	TCGGT	TCGGC	TCGGG
TGA	TGAAA	TGAAT	TGAAC	TGAAG	TGATA	TGATT	TGATC	TGATG	TGACA	TGACT	TGACC	TGACG	TGAGA	TGAGT	TGAGC	TGAGG
TGT	TGTAA	TGTAT	TGTAC	TGTAG	TGTTA	TGTTT	TGTTC	TGTTG	TGTCA	TGTCT	TGTCC	TGTCG	TGTGA	TGTGT	TGTGC	TGTGG
TGC	TGCAA	TGCAT	TGCAC	TGCAG	TGCTA	TGCTT	TGCTC	TGCTG	TGCCA	TGCCT	TGCCC	TGCCG	TGCGA	TGCGT	TGCGC	TGCGG
TGG	TGGAA	TGGAT	TGGAC	TGGAG	TGGTA	TGGTT	TGGTC	TGGTG	TGGCA	TGGCT	TGGCC	TGGCG	TGGGA	TGGGT	TGGGC	TGGGG
CAA	CAAAA	CAAAT	CAAAC	CAAAG	CAATA	CAATT	CAATC	CAATG	CAACA	CAACT	CAACC	CAACG	CAAGA	CAAGT	CAAGC	CAAGG
CAT	CATAA	CATAT	CATAC	CATAG	CATTA	CATTT	CATTC	CATTG	CATCA	CATCT	CATCC	CATCG	CATGA	CATGT	CATGC	CATGG
CAC	CACAA	CACAT	CACAC	CACAG	CACTA	CACTT	CACTC	CACTG	CACCA	CACCT	CACCC	CACCG	CACGA	CACGT	CACGC	CACGG
CAG	CAGAA	CAGAT	CAGAC	CAGAG	CAGTA	CAGTT	CAGTC	CAGTG	CAGCA	CAGCT	CAGCC	CAGCG	CAGGA	CAGGT	CAGGC	CAGGG
CTA	CTAAA	CTAAT	CTAAC	CTAAG	CTATA	CTATT	CTATC	CTATG	CTACA	CTACT	CTACC	CTACG	CTAGA	CTAGT	CTAGC	CTAGG
CTT	CTTAA	CTTAT	CTTAC	CTTAG	CTTTA	CTTTT	CTTTC	CTTTG	CTTCA	CTTCT	CTTCC	CTTCG	CTTGA	CTTGT	CTTGC	CTTGG
CTC	CTCAA	CTCAT	CTCAC	CTCAG	CTCTA	CTCTT	CTCTC	CTCTG	CTCCA	CTCCT	CTCCC	CTCCG	CTCGA	CTCGT	CTCGC	CTCGG
CTG	CTGAA	CTGAT	CTGAC	CTGAG	CTGTA	CTGTT	CTGTC	CTGTG	CTGCA	CTGCT	CTGCC	CTGCG	CTGGA	CTGGT	CTGGC	CTGGG
CCA	CCAAA	CCAAT	CCAAC	CCAAG	CCATA	CCATT	CCATC	CCATG	CCACA	CCACT	CCACC	CCACG	CCAGA	CCAGT	CCAGC	CCAGG
CCT	CCTAA	CCTAT	CCTAC	CCTAG	CCTTA	CCTTT	CCTTC	CCTTG	CCTCA	CCTCT	CCTCC	CCTCG	CCTGA	CCTGT	CCTGC	CCTGG
CCC	CCCAA	CCCAT	CCCAC	CCCAG	CCCTA	CCCTT	CCCTC	CCCTG	CCCCA	CCCCT	CCCCC	CCCCG	CCCGA	CCCGT	CCCGC	CCCGG
CCG	CCGAA	CCGAT	CCGAC	CCGAG	CCGTA	CCGTT	CCGTC	CCGTG	CCGCA	CCGCT	CCGCC	CCGCG	CCGGA	CCGGT	CCGGC	CCGGG
CGA	CGAAA	CGAAT	CGAAC	CGAAG	CGATA	CGATT	CGATC	CGATG	CGACA	CGACT	CGACC	CGACG	CGAGA	CGAGT	CGAGC	CGAGG
CGT	CGTAA	CGTAT	CGTAC	CGTAG	CGTTA	CGTTT	CGTTC	CGTTG	CGTCA	CGTCT	CGTCC	CGTCG	CGTGA	CGTGT	CGTGC	CGTGG
CGC	CGCAA	CGCAT	CGCAC	CGCAG	CGCTA	CGCTT	CGCTC	CGCTG	CGCCA	CGCCT	CGCCC	CGCCG	CGCGA	CGCGT	CGCGC	CGCGG
CGG	CGGAA	CGGAT	CGGAC	CGGAG	CGGTA	CGGTT	CGGTC	CGGTG	CGGCA	CGGCT	CGGCC	CGGCG	CGGGA	CGGGT	CGGGC	CGGGG
GAA	GAAAA	GAAAT	GAAAC	GAAAG	GAATA	GAATT	GAATC	GAATG	GAACA	GAACT	GAACC	GAACG	GAAGA	GAAGT	GAAGC	GAAGG
GAT	GATAA	GATAT	GATAC	GATAG	GATTA	GATTT	GATTC	GATTG	GATCA	GATCT	GATCC	GATCG	GATGA	GATGT	GATGC	GATGG
GAC	GACAA	GACAT	GACAC	GACAG	GACTA	GACTT	GACTC	GACTG	GACCA	GACCT	GACCC	GACCG	GACGA	GACGT	GACGC	GACGG
GAG	GAGAA	GAGAT	GAGAC	GAGAG	GAGTA	GAGTT	GAGTC	GAGTG	GAGCA	GAGCT	GAGCC	GAGCG	GAGGA	GAGGT	GAGGC	GAGGG
GTA	GTAAA	GTAAT	GTAAC	GTAAG	GTATA	GTATT	GTATC	GTATG	GTACA	GTACT	GTACC	GTACG	GTAGA	GTAGT	GTAGC	GTAGG
GTT	GTTAA	GTTAT	GTTAC	GTTAG	GTTTA	GTTTT	GTTTC	GTTTG	GTTCA	GTTCT	GTTCC	GTTCG	GTTGA	GTTGT	GTTGC	GTTGG
GTC	GTCAA	GTCAT	GTCAC	GTCAG	GTCTA	GTCTT	GTCTC	GTCTG	GTCCA	GTCCT	GTCCC	GTCCG	GTCGA	GTCGT	GTCGC	GTCGG
GTG	GTGAA	GTGAT	GTGAC	GTGAG	GTGTA	GTGTT	GTGTC	GTGTG	GTGCA	GTGCT	GTGCC	GTGCG	GTGGA	GTGGT	GTGGC	GTGGG
GCA	GCAAA	GCAAT	GCAAC	GCAAG	GCATA	GCATT	GCATC	GCATG	GCACA	GCACT	GCACC	GCACG	GCAGA	GCAGT	GCAGC	GCAGG
GCT	GCTAA	GCTAT	GCTAC	GCTAG	GCTTA	GCTTT	GCTTC	GCTTG	GCTCA	GCTCT	GCTCC	GCTCG	GCTGA	GCTGT	GCTGC	GCTGG
GCC	GCCAA	GCCAT	GCCAC	GCCAG	GCCTA	GCCTT	GCCTC	GCCTG	GCCCA	GCCCT	GCCCC	GCCCG	GCCGA	GCCGT	GCCGC	GCCGG
GCG	GCGAA	GCGAT	GCGAC	GCGAG	GCGTA	GCGTT	GCGTC	GCGTG	GCGCA	GCGCT	GCGCC	GCGCG	GCGGA	GCGGT	GCGGC	GCGGG
GGA	GGAAA	GGAAT	GGAAC	GGAAG	GGATA	GGATT	GGATC	GGATG	GGACA	GGACT	GGACC	GGACG	GGAGA	GGAGT	GGAGC	GGAGG
GGT	GGTAA	GGTAT	GGTAC	GGTAG	GGTTA	GGTTT	GGTTC	GGTTG	GGTCA	GGTCT	GGTCC	GGTCG	GGTGA	GGTGT	GGTGC	GGTGG
GGC	GGCAA	GGCAT	GGCAC	GGCAG	GGCTA	GGCTT	GGCTC	GGCTG	GGCCA	GGCCT	GGCCC	GGCCG	GGCGA	GGCGT	GGCGC	GGCGG
GGG	GGGAA	GGGAT	GGGAC	GGGAG	GGGTA	GGGTT	GGGTC	GGGTG	GGGCA	GGGCT	GGGCC	GGGCG	GGGGA	GGGGT	GGGGC	GGGGG

After filtering: read2: overrepresented sequences

Sampling rate: 1 / 20

overrepresented sequence	count (% of bases)	distribution: cycle 1 ~ cycle 150
AAAAAAAAAAAAAAAAAAAAAAAAAAAAAAAAAAAAAAAAAAAAAAAAAAAAAAAAAAAAAAAAAAAAAAAAAAAAAAAAAAAAAAAAAAAAAAAAAAAAA	749 (0.003007%)	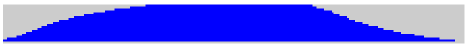
AAGATCGATC	1127304 (0.452592%)	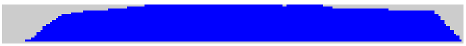
AGGATCGATC	885316 (0.355438%)	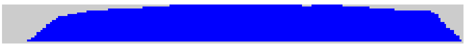
ATGATCGATC	1057436 (0.424541%)	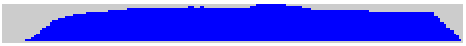
GATCGATCAA	435102 (0.174686%)	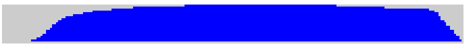
GATCGATCAT	462890 (0.185842%)	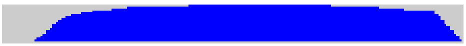
GATCGATCCT	439461 (0.176436%)	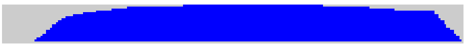
GATCGATCTT	557663 (0.223892%)	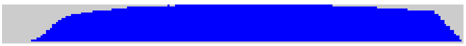
TGATCGATCA	170869 (0.068601%)	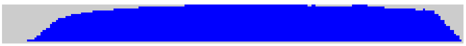
TTGATCGATC	1010176 (0.405567%)	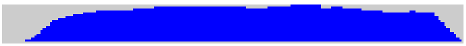

TTTTTTTTTT	235186 (0.094423%)	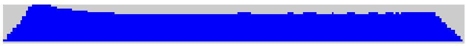

```
fastp -p -i resources/raw_hic/SRR18311512_1.fastq.gz -I resources/raw_hic/SRR18311512_2.fastq.gz -o
results/fastp/hic_trim_1.fastq.gz -O results/fastp/hic_trim_2.fastq.gz --detect_adapter_for_pe --json
results/fastp/hic_report_fastp.HiC.json --html results/fastp/hic_report_fastp.HiC.html --thread 20

fastp 0.23.4, at 2024-05-07 10:48:51
```
