# Supplementary material for: Colora: a Snakemake workflow for complete chromosome-scale de novo genome assembly
Source: Bioinformatics. 2025 Apr 16;41(5):btaf175. doi: 10.1093/bioinformatics/btaf175 (PMC12065627; doi:10.1093/bioinformatics/btaf175)
Supplement: btaf175_Supplementary_Data [file btaf175_supplementary_data.zip › Additional_files/S12_R.irregularis_mitochondrion_gfastats.stats.pdf]

Gfastats results for *R. irregularis* mitochondrion

```
+++Assembly summary+++:
# scaffolds: 1
Total scaffold length: 70272
Average scaffold length: 70272.00
Scaffold N50: 70272
Scaffold auN: 70272.00
Scaffold L50: 1
Largest scaffold: 70272
Smallest scaffold: 70272
# contigs: 1
Total contig length: 70272
Average contig length: 70272.00
Contig N50: 70272
Contig auN: 70272.00
Contig L50: 1
Largest contig: 70272
Smallest contig: 70272
# gaps in scaffolds: 0
Total gap length in scaffolds: 0
Average gap length in scaffolds: 0.00
Gap N50 in scaffolds: 0
Gap auN in scaffolds: 0.00
Gap L50 in scaffolds: 0
Largest gap in scaffolds: 0
Smallest gap in scaffolds: 0
Base composition (A:C:G:T): 22929:13130:13106:21107
GC content %: 37.33
# soft-masked bases: 0
# segments: 1
Total segment length: 70272
Average segment length: 70272.00
# gaps: 0
# paths: 1
```
