# Supplementary material for: Colora: a Snakemake workflow for complete chromosome-scale de novo genome assembly
Source: Bioinformatics. 2025 Apr 16;41(5):btaf175. doi: 10.1093/bioinformatics/btaf175 (PMC12065627; doi:10.1093/bioinformatics/btaf175)
Supplement: btaf175_Supplementary_Data [file btaf175_supplementary_data.zip › Additional_files/S14_M.domestica_mitochondrion_gfastats.stats.pdf]

Gfastats results for *M. domestica* mitochondrion

```
+++Assembly summary+++:
# scaffolds: 1
Total scaffold length: 396948
Average scaffold length: 396948.00
Scaffold N50: 396948
Scaffold auN: 396948.00
Scaffold L50: 1
Largest scaffold: 396948
Smallest scaffold: 396948
# contigs: 1
Total contig length: 396948
Average contig length: 396948.00
Contig N50: 396948
Contig auN: 396948.00
Contig L50: 1
Largest contig: 396948
Smallest contig: 396948
# gaps in scaffolds: 0
Total gap length in scaffolds: 0
Average gap length in scaffolds: 0.00
Gap N50 in scaffolds: 0
Gap auN in scaffolds: 0.00
Gap L50 in scaffolds: 0
Largest gap in scaffolds: 0
Smallest gap in scaffolds: 0
Base composition (A:C:G:T): 108311:89455:90789:108393
GC content %: 45.41
# soft-masked bases: 0
# segments: 1
Total segment length: 396948
Average segment length: 396948.00
# gaps: 0
# paths: 1
```
