# Supplementary material for: Colora: a Snakemake workflow for complete chromosome-scale de novo genome assembly
Source: Bioinformatics. 2025 Apr 16;41(5):btaf175. doi: 10.1093/bioinformatics/btaf175 (PMC12065627; doi:10.1093/bioinformatics/btaf175)
Supplement: btaf175_Supplementary_Data [file btaf175_supplementary_data.zip › Additional_files/S15_A.thaliana_chloroplast_gfastats.stats.pdf]

Gfastats results for *A. thaliana* chloroplast

```
+++Assembly summary+++:
# scaffolds: 1
Total scaffold length: 154479
Average scaffold length: 154479.00
Scaffold N50: 154479
Scaffold auN: 154479.00
Scaffold L50: 1
Largest scaffold: 154479
Smallest scaffold: 154479
# contigs: 1
Total contig length: 154479
Average contig length: 154479.00
Contig N50: 154479
Contig auN: 154479.00
Contig L50: 1
Largest contig: 154479
Smallest contig: 154479
# gaps in scaffolds: 0
Total gap length in scaffolds: 0
Average gap length in scaffolds: 0.00
Gap N50 in scaffolds: 0
Gap auN in scaffolds: 0.00
Gap L50 in scaffolds: 0
Largest gap in scaffolds: 0
Smallest gap in scaffolds: 0
Base composition (A:C:G:T): 48546:28496:27570:49867
GC content %: 36.29
# soft-masked bases: 0
# segments: 1
Total segment length: 154479
Average segment length: 154479.00
# gaps: 0
# paths: 1
```
