# Supplementary material for: Colora: a Snakemake workflow for complete chromosome-scale de novo genome assembly
Source: Bioinformatics. 2025 Apr 16;41(5):btaf175. doi: 10.1093/bioinformatics/btaf175 (PMC12065627; doi:10.1093/bioinformatics/btaf175)
Supplement: btaf175_Supplementary_Data [file btaf175_supplementary_data.zip › Additional_files/S16_M.domestica_chloroplast_gfastats.stats.pdf]

Gfastats results for *M. domestica* chloroplast

```
+++Assembly summary+++:
# scaffolds: 1
Total scaffold length: 160072
Average scaffold length: 160072.00
Scaffold N50: 160072
Scaffold auN: 160072.00
Scaffold L50: 1
Largest scaffold: 160072
Smallest scaffold: 160072
# contigs: 1
Total contig length: 160072
Average contig length: 160072.00
Contig N50: 160072
Contig auN: 160072.00
Contig L50: 1
Largest contig: 160072
Smallest contig: 160072
# gaps in scaffolds: 0
Total gap length in scaffolds: 0
Average gap length in scaffolds: 0.00
Gap N50 in scaffolds: 0
Gap auN in scaffolds: 0.00
Gap L50 in scaffolds: 0
Largest gap in scaffolds: 0
Smallest gap in scaffolds: 0
Base composition (A:C:G:T): 50131:29834:28677:51430
GC content %: 36.55
# soft-masked bases: 0
# segments: 1
Total segment length: 160072
Average segment length: 160072.00
# gaps: 0
# paths: 1
```
