# Supplementary material for: Colora: a Snakemake workflow for complete chromosome-scale de novo genome assembly
Source: Bioinformatics. 2025 Apr 16;41(5):btaf175. doi: 10.1093/bioinformatics/btaf175 (PMC12065627; doi:10.1093/bioinformatics/btaf175)
Supplement: btaf175_Supplementary_Data [file btaf175_supplementary_data.zip › Additional_files/S11_complete_QUAST_reports.pdf]

Complete QUAST report for *R. irregularis*

	Colora results						Reference genome	Original paper results	
Assembly	asm_hap1	asm_hap2	fcsgx_hap1	fcsgx_hap2	yahs_hap1	yahs_hap2	GCF_026210795.1_ASM2621079v1	R_irregularis_G1_haplotype1	R_irregularis_G1_haplotype2
# contigs (>= 0 bp)	183	109	164	106	152	97	32	53	53
# contigs (>= 1000 bp)	183	109	164	106	152	97	32	53	53
# contigs (>= 5000 bp)	183	109	164	106	152	97	32	53	53
# contigs (>= 10000 bp)	183	109	164	106	152	97	32	53	53
# contigs (>= 25000 bp)	149	97	134	94	122	85	32	45	52
# contigs (>= 50000 bp)	60	49	58	46	46	38	32	33	45
Total length (>= 0 bp)	149918426	147032028	149290886	146497146	149292186	146498046	146773001	144127305	147419605
Total length (>= 1000 bp)	149918426	147032028	149290886	146497146	149292186	146498046	146773001	144127305	147419605
Total length (>= 5000 bp)	149918426	147032028	149290886	146497146	149292186	146498046	146773001	144127305	147419605
Total length (>= 10000 bp)	149918426	147032028	149290886	146497146	149292186	146498046	146773001	144127305	147419605
Total length (>= 25000 bp)	149219379	146779350	148685683	146244468	148686983	146245368	146773001	143971042	147403771
Total length (>= 50000 bp)	146242975	145159261	146110621	144624379	146111921	144669293	146773001	143586331	147149411
# contigs	183	109	164	106	152	97	32	53	53
Largest contig	7814124	7795048	7814124	7795048	7814124	7839162	7995222	7810117	7800626
Total length	149918426	147032028	149290886	146497146	149292186	146498046	146773001	144127305	147419605
GC (%)	27.74	27.71	27.69	27.64	27.69	27.64	27.84	27.54	27.51
N50	3588378	4297361	3588378	4297361	4862057	4871874	5085394	4928871	4971883
N90	2006314	2620892	2006314	2620892	2736882	3232781	3440831	3232763	3225433
auN	3883265	4184950.2	3899429.1	4199462.5	4518255.4	4684466.4	4873894.9	4760804	4766283.9
L50	15	14	15	14	14	14	13	13	14
L90	36	32	36	32	30	28	28	28	28
# N's per 100 kbp	0	0	0	0	0.87	0.61	1.36	0.35	0.95

Complete QUAST report for *A. thaliana*

	Colora results				Reference genome*	Original paper results
**Assembly**	**asm_primary**	**fcsgx_primary**	**purged_primary**	**yahs_primary**	**GCA_000001735.2_TAIR10.1**	**GWHBDNP00000000.1**
# contigs (>= 0 bp)	507	507	19	18	7	5
# contigs (>= 1000 bp)	507	507	19	18	7	5
# contigs (>= 5000 bp)	500	500	14	13	7	5
# contigs (>= 10000 bp)	499	499	13	12	7	5
# contigs (>= 25000 bp)	478	478	13	12	7	5
# contigs (>= 50000 bp)	186	186	7	6	7	5
Total length (>= 0 bp)	160397006	160397006	134384931	134385031	119668634	133725193
Total length (>= 1000 bp)	160397006	160397006	134384931	134385031	119668634	133725193
Total length (>= 5000 bp)	160376359	160376359	134369164	134369264	119668634	133725193
Total length (>= 10000 bp)	160366996	160366996	134359801	134359901	119668634	133725193
Total length (>= 25000 bp)	159931865	159931865	134359801	134359901	119668634	133725193
Total length (>= 50000 bp)	148770985	148770985	134140209	134140309	119668634	133725193
# contigs	507	507	19	18	7	5
Largest contig	32656027	32656027	32656027	48425965	30427671	32659241
Total length	160397006	160397006	134384931	134385031	119668634	133725193
Reference length	119668634	119668634	119668634	119668634	-	-
GC (%)	37.02	37.02	36.37	36.37	36.06	36.34
Reference GC (%)	36.06	36.06	36.06	36.06	-	-
N50	26162003	26162003	26162003	32656027	23459830	26161332
NG50	30145414	30145414	30145414	32656027	18585056	22250686
N90	60830	60830	22263862	22674124	24546704	27374990.3
NG90	22674124	22674124	22674124	30145414	3	3
auN	22892065.5	22892065.5	27305490.1	35974174.4	5	5
auNG	30683217.9	30683217.9	30663393.4	40398142.6	155.6	0.15
L50	3	3	3	2	-	-
LG50	2	2	2	2	-	-
L90	107	107	5	4	-	-
LG90	4	4	4	3	-	-
# misassemblies	2328	2328	1681	1676	-	-
# misassembled contigs	127	127	8	7	-	-
Misassembled contigs length	143776289	143776289	134077020	134077120	-	-

# local misassemblies	912	912	626	622	-	-
# scaffold gap ext. mis.	0	0	0	0	-	-
# scaffold gap loc. mis.	0	0	0	0	-	-
# unaligned mis. contigs	0	0	0	0	-	-
# unaligned contigs	4 + 17 part	4 + 17 part	2 + 6 part	2 + 5 part	-	-
Unaligned length	11574156	11574156	11526791	11543684	-	-
Genome fraction (%)	99.615	99.615	99.431	99.416	-	-
Duplication ratio	1.244	1.244	1.03	1.03	-	-
# N's per 100 kbp	0	0	0	0.07	-	-
# mismatches per 100 kbp	71.38	71.38	70.03	69.66	-	-
# indels per 100 kbp	14.96	14.96	8.48	8.42	-	-
# genomic features	709529 + 147 part	709529 + 147 part	708502 + 146 part	708508 + 146 part	-	-
Largest alignment	9639574	9639574	9639574	9639574	-	-
Total aligned length	148079254	148079254	122343224	122358033	-	-
NA50	2431317	2431317	3601484	3601484	-	-
NGA50	3956256	3956256	3956256	3956256	-	-
NA90	8936	8936	4405	4405	-	-
NGA90	842402	842402	842402	842402	-	-
auNA	3261818.6	3261818.6	3885490.4	3886035.4	-	-
auNGA	4371955.5	4371955.5	4363310.1	4363925.4	-	-
LA50	17	17	12	12	-	-
LGA50	10	10	10	10	-	-
LA90	1108	1108	337	333	-	-
LGA90	36	36	36	36	-	-

* it includes mitochondrion and chloroplast

Complete QUAST report for *M. domestica*

	Colora results						Reference genome*	Original paper results		
**Assembly**	**asm_hap1**	**asm_hap2**	**fcsgx_hap1**	**fcsgx_hap2**	**yahs_hap1**	**yahs_hap2**	**GCF_002114115.1_ASM211411v1**	**GCA_033963175.2_Fuji_haploid_consensus**	**GCA_033962815.2_Fuji_haplome_A**	**GCA_033962845.2_Fuji_haplome_B**
# contigs (>= 0 bp)	1137	287	1135	287	1126	278	807	1358	206	164
# contigs (>= 1000 bp)	1137	287	1135	287	1126	278	806	1358	206	164
# contigs (>= 5000 bp)	1137	287	1135	287	1126	278	793	1358	206	164
# contigs (>= 10000 bp)	1136	286	1134	286	1125	277	776	1358	206	164
# contigs (>= 25000 bp)	611	218	611	218	602	209	631	625	177	151
# contigs (>= 50000 bp)	124	108	124	108	115	99	379	102	85	79
Total length (>= 0 bp)	686890689	662610989	686842438	662610989	686843538	662612189	703358299	736926135	692760243	671417745
Total length (>= 1000 bp)	686890689	662610989	686842438	662610989	686843538	662612189	703357371	736926135	692760243	671417745
Total length (>= 5000 bp)	686890689	662610989	686842438	662610989	686843538	662612189	703327981	736926135	692760243	671417745
Total length (>= 10000 bp)	686882013	662603660	686833762	662603660	686834862	662604860	703201884	736926135	692760243	671417745
Total length (>= 25000 bp)	675914062	661226252	675914062	661226252	675915162	661227452	700514125	721318658	692158219	671154676
Total length (>= 50000 bp)	660503316	657533713	660503316	657533713	660504416	657534913	691430802	705436290	688977363	668522060
# contigs	1137	287	1135	287	1126	278	807	1358	206	164
Largest contig	56194835	55977444	56194835	55977444	96327438	96762219	55080361	56797885	56619705	56701660
Total length	686890689	662610989	686842438	662610989	686843538	662612189	703358299	736926135	692760243	671417745
Reference length	702961352	702961352	702961352	702961352	702961352	702961352	-	-	-	-
GC (%)	38.35	38.32	38.35	38.32	38.35	38.32	38.04	38.46	38.46	38.36
Reference GC (%)	38.04	38.04	38.04	38.04	38.04	38.04	-	-	-	-
N50	28328816	32709378	28328816	32709378	37217313	36870539	37631755	36771785	37279547	36869019
NG50	28328816	32709378	28328816	32709378	36260008	36490050	31666303	16528878	31387705	31216434
N90	14291838	10063527	14291838	10063527	31387149	31202618	37130857.6	36447281.9	38113525.4	38299801
NG90	12253221	8785574	12253221	8785574	31375366	30267070	9	9	8	8
auN	28654148.9	30615883.4	28656160.2	30615883.4	43766959.2	45310096.3	17	18	17	16
auNG	27999075.7	28858515.1	27999074	28858515.1	42763450.7	42709349.5	11045.1	0.11	18.12	6.85
L50	10	9	10	9	7	7	-	-	-	-
LG50	10	9	10	9	8	8	-	-	-	-
L90	23	21	23	21	15	15	-	-	-	-
LG90	24	25	24	25	16	16	-	-	-	-
# misassemblies	50676	53675	50676	53675	50670	53658	-	-	-	-
# misassembled contigs	467	237	467	237	458	228	-	-	-	-

Misassembled contigs length	668493352	660673734	668493352	660673734	668494452	660674934	-	-	-	-
# local misassemblies	14248	14881	14248	14881	14247	14897	-	-	-	-
# scaffold gap ext. mis.	0	0	0	0	2	1	-	-	-	-
# scaffold gap loc. mis.	0	0	0	0	3	4	-	-	-	-
# unaligned mis. contigs	654	40	654	40	654	40	-	-	-	-
# unaligned contigs	2 + 1092 part	0 + 263 part	0 + 1092 part	0 + 263 part	0 + 1083 part	0 + 254 part	-	-	-	-
Unaligned length	70549919	63672113	70501668	63672113	70561359	63717879	-	-	-	-
Genome fraction (%)	85.246	83.527	85.246	83.527	85.256	83.527	-	-	-	-
Duplication ratio	1.149	1.14	1.149	1.14	1.15	1.14	-	-	-	-
# N's per 100 kbp	0	0	0	0	0.16	0.18	-	-	-	-
# mismatches per 100 kbp	909.08	1001.97	909.08	1001.97	908.44	1001.44	-	-	-	-
# indels per 100 kbp	161.26	166.35	161.26	166.35	161.17	166.2	-	-	-	-
Largest alignment	2220882	1641104	2220882	1641104	2218997	1641104	-	-	-	-
Total aligned length	612656624	595453294	612656624	595453294	612832964	595614243	-	-	-	-
NA50	30907	26835	30909	26835	30900	26838	-	-	-	-
NGA50	29105	23503	29105	23503	29104	23525	-	-	-	-
NA90	-	-	-	-	-	-	-	-	-	-
NGA90	-	-	-	-	-	-	-	-	-	-
auNA	127932.8	113172.3	127941.7	113172.3	128006.8	113276.3	-	-	-	-
auNGA	125008	106676.1	125008	106676.1	125071.8	106774.4	-	-	-	-
LA50	3803	4455	3802	4455	3801	4451	-	-	-	-
LGA50	4071	5258	4071	5258	4070	5253	-	-	-	-
LA90	-	-	-	-	-	-	-	-	-	-
LGA90	-	-	-	-	-	-	-	-	-	-

* it includes the mitochondrion
