# Supplementary material for: Colora: a Snakemake workflow for complete chromosome-scale de novo genome assembly
Source: Bioinformatics. 2025 Apr 16;41(5):btaf175. doi: 10.1093/bioinformatics/btaf175 (PMC12065627; doi:10.1093/bioinformatics/btaf175)
Supplement: btaf175_Supplementary_Data [file btaf175_supplementary_data.zip › Additional_files/S18_BUSCO_results_for_reference_and_original_assemblies.pdf]

BUSCO Assessment resuls for the reference genome of *R. irregularis* and for

	Reference genome	Original paper results	
	**GCF_026210795.1_ASM2621079v1**	**R_irregularis_G1_haplotype1**	**R_irregularis_G1_haplotype2**
Complete	1587 (98.3%)	1586 (98.3%)	1585 (98.2%)
Complete and single-copy	1582 (98.0%)	1577 (97.7%)	1544 (95.7%)
Complete and duplicated	5 (0.3%)	9 (0.6%)	41 (2.5%)
Fragmented	4 (0.2%)	5 (0.3%)	5 (0.3%)
Missing	23 (1.5%)	23 (1.4%)	24 (1.5%)

database: mucoromycota_odb10

BUSCO Assessment resuls for the reference genome of *A. thaliana* and for the assemblies produced by Wang et al. (2022)

	Reference genome	Original paper results
	GCA_000001735.2_TAIR10.1	GWHBDNP00000000.1
Complete	4593 (99.9%)	4593 (100.0%)
Complete and single-copy	4554 (99.1%)	4553 (99.1%)
Complete and duplicated	39 (0.8%)	40 (0.9%)
Fragmented	3 (0.1%)	3 (0.1%)
Missing	0 (0.0%)	0 (0.0%)

database: brassicales_odb10

BUSCO Assessment resuls for the reference genome of *M. domestica* and for the assemblies produced by Li et al. (2024)

	Reference genome	Original paper results		
	GCF_002114115.1_ASM211411v1	GCA_033963175.2_Fuji_haploid_consensus	GCA_033962815.2_Fuji_haplome_A	GCA_033962845.2_Fuji_haplome_B
Complete	2275 (97.8%)	2291 (98.5%)	2292 (98.5%)	2289 (98.4%)
Complete and single-copy	1322 (56.8%)	1261 (54.2%)	1301 (55.9%)	1298 (55.8%)
Complete and duplicated	953 (41.0%)	1030 (44.3%)	991 (42.6%)	991 (42.6%)
Fragmented	15 (0.6%)	14 (0.6%)	14 (0.6%)	16 (0.7%)
Missing	36 (1.6%)	21 (0.9%)	20 (0.9%)	21 (0.9%)

database: eudicots_odb10
